# Sequence of the Mitochondrial Genome of *Lactuca virosa* Suggests an Unexpected Role in *Lactuca sativa*’s Evolution

**DOI:** 10.3389/fpls.2021.697136

**Published:** 2021-07-26

**Authors:** Arnaud Fertet, Stéfanie Graindorge, Sandrine Koechler, Gert-Jan de Boer, Emilie Guilloteau-Fonteny, José M. Gualberto

**Affiliations:** ^1^Institut de Biologie Moléculaire des Plantes, CNRS, Université de Strasbourg, Strasbourg, France; ^2^Enza Zaden Research and Development B.V., Enkhuizen, Netherlands; ^3^Enza Zaden France Recherche S.A.S., Allonnes, France

**Keywords:** mitochondrial genome, genome evolution, lettuce, *Lactuca virosa*, *Lactuca serriola*, *Lactuca saligna*

## Abstract

The involvement of the different *Lactuca* species in the domestication and diversification of cultivated lettuce is not totally understood. *Lactuca serriola* is considered as the direct ancestor and the closest relative to *Lactuca sativa*, while the other wild species that can be crossed with *L. sativa*, *Lactuca virosa*, and *Lactuca saligna*, would have just contributed to the latter diversification of cultivated typologies. To contribute to the study of *Lactuca* evolution, we assembled the mtDNA genomes of nine *Lactuca* spp. accessions, among them three from *L. virosa*, whose mtDNA had not been studied so far. Our results unveiled little to no intraspecies variation among *Lactuca* species, with the exception of *L. serriola* where the accessions we sequenced diverge significantly from the mtDNA of a *L. serriola* accession already reported. Furthermore, we found a remarkable phylogenetic closeness between the mtDNA of *L. sativa* and the mtDNA of *L. virosa*, contrasting to the *L. serriola* origin of the nuclear and plastidial genomes. These results suggest that a cross between *L. virosa* and the ancestor of cultivated lettuce is at the origin of the actual mitochondrial genome of *L. sativa.*

## Introduction

Within the *Lactuca* genus which contains about 100 species, four species are well-described and characterized: the cultivated one, *Lactuca sativa*, and three wild species, *L. saligna*, *L. serriola*, and *Lactuca virosa*, which can all be crossed with *L. sativa* (Ryder, 1999). *L. saligna*, with its long and narrow leaves (Lindqvist, 1960), is also called “least lettuce,” while *L. serriola* is named “prickly lettuce,” because of the harder prickles in its lobed leaves and stem. *L. virosa*, or “great lettuce,” shows many phenotypic variations according to ecotypes: leaves can be lobed or not, some with prickles others not, but all have broad leaves (Lindqvist, 1960). The differences between *L. sativa*, the domesticated lettuce, and the wild species are morphological, like the formation of the lettuce head at the vegetative stage, and a less bitter taste through the reduction of latex production. On the market, four *L. sativa* typologies are predominant (Davis et al., 1997): Crisphead lettuces (var. *capitata* L. *salinas*), Cos lettuces (var. *longifolia*), leaf lettuces (var. *acephala* Alef.), and Butterhead lettuces (var. *capitata* L. *nidus tenerrima*).

The process of domestication, that led to *L. sativa*, is not clearly understood. It is established that *L. serriola* is one of the direct ancestors and the closest related species (de Vries, 1990; Kesseli et al., 1991; de Vries and van Raamsdonk, 1994) and that hybrids *L. sativa* × *L. serriola* are self-fertile (Thompson et al., 1941; Thompson, 1943). *L. virosa* and *L. saligna* are playing an important role in the development of lettuce cultivars, and especially in the introgression of resistance genes like the ones for *Bremia lactucae* (Parra et al., 2021). However, a study based on RFLP markers suggested that *L. sativa* could have a polyphyletic origin, but without being able to identify other contributing species (Kesseli et al., 1991). More recently, a study investigated the genealogy of North American lettuces and identified *L. virosa* as introgressed in *L. sativa* in certain lineages of cultivars, such as in a group of Crisphead lettuces. *L. virosa* would contribute to a more robust root system and decreased leaf drop (Mikel, 2007). The advances in sequencing technologies and the recent increase in available sequenced genomes helped to clarify the relationship between *Lactuca* spp. Two sets of markers were developed: as nuclear marker, a set of ribosomal internal transcribed spacers (ITS), ITS-1 and ITS-2, which flank the *5.8* rRNA gene sequence, and as chloroplast marker the concatenated sequence of the *ndhF* and *trnL-F* genes (Koopman et al., 1998; Wei et al., 2017). These studies showed that the *Lactuca* spp. can be subdivided into several clades among which the *crop group* containing *L. sativa*, *L. saligna*, *L. serriola*, and *L. virosa* (Wei et al., 2017). This subdivision goes further and the species can be distinguished, with the very exception of *L. sativa* and *L. serriola* that could not be separated from each other (Koopman et al., 2001). Regarding the mitochondrial genome (or mtDNA), the ones of *L. sativa* (363 kb), *L. saligna* (368 kb), and *L. serriola* (363 kb) were recently published, which allowed the development of a new set of markers (Kozik et al., 2019).

It is not surprising that the last *L. sativa* genome released was the mtDNA. Indeed, the large size and complex and dynamic structure of plant mitochondrial genomes makes their sequencing and assembly delicate. The presence of mitochondrial sequences inserted in the nuclear genome, also known as NUMTs (Nuclear MiTochondrial sequences), further complicates their assembly from total genomic data. This is a relatively lesser issue in *Arabidopsis thaliana* and its small nuclear genome of ±157 Mb (The Arabidopsis Genome Initiative, 2000; Bennett et al., 2003), but it is a different situation for plants with much bigger nuclear genomes, such as lettuce (2.9 Gb) (Reyes-Chin-Wo et al., 2017). There are two ways to work around this problem: choose a higher-throughput sequencing strategy to increase sequencing deep, or enrich the mtDNA concentration by extracting it from purified mitochondria. Then, once the sequencing is done the challenge is not finished. Plant mitochondrial genomes have highly dynamic and multipartite structures because of large repeated sequences (≥500 bp) involved in frequent and reciprocal recombination, which makes their assembly tricky. These large repeated regions cannot be crossed by a conventional short-read sequencing strategy. Consequently, during assembly these sequences remain contigs that can be linked to four other contigs (two per side) leading to four possible configurations. This assembly issue can only be overcome using a long-reads strategy (Pacbio SMRT or Illumina mate-pair) in combination with a short-reads one.

The content of plant mtDNAs varies little between species. Indeed, there are rarely any differences in gene content within a genus. Even in *Silene* spp. where there are massive changes in mtDNA genome size and structure, very few differences in gene content can be observed (Sloan et al., 2012). And there are even fewer intraspecific differences, like in *A. thaliana*, where different accessions have the same content in protein and tRNA genes (Davila et al., 2011; Sloan et al., 2018). In fact, the diversity of plant mtDNAs is mainly found in their structure, such as in *A. thaliana* where the different sequences of the mtDNA are differently organized in different accessions, as in Col-0, C24, and Landsberg erecta (Davila et al., 2011). A major source of diversity in plant mtDNA content comes from its interaction with other genomes, by horizontal sequence transfer (Marienfeld et al., 1999; Bergthorsson et al., 2003), and in particular with the plastidial genome (cpDNA). Indeed, the mtDNA of plants is often strewn with plastid DNA insertions. Most plastid DNA sequences transferred to the mtDNA are neutral (Cummings et al., 2003), but several chloroplast-derived tRNA genes have been recruited to be functional in mitochondria (Dietrich et al., 1996).

In this work our goal was to assess the diversity of mtDNA genomes interspecies among different *Lactuca* species, and intraspecies among different accessions of the same species. The final objective is to better understand the contribution of the different wild-type *Lactuca* species to the domestication and diversification of *L. sativa*. Here we determined the complete mtDNA sequences of nine accessions from four related species in the genus *Lactuca*. The nuclear data confirmed previous reports showing that *L. sativa* and *L. serriola* are closely related species and distinct from *L. saligna* and *L. virosa* (Koopman et al., 1998; Wei et al., 2017; Zhang et al., 2017). The phylogeny of the complete chloroplastic genome sequences was in line with the nuclear one. However, we found that from the sequence and structural points of view the mtDNA of *L. sativa* is closer to the one from *L. virosa* than to the ones from *L. saligna* or *L. serriola*. The close phylogenetic relation between the mtDNA of *L. sativa* and *L. virosa* is further supported by a clear divergence in cpDNA insertions in the mtDNA, where *L. sativa* and *L. virosa* share a same specific one, whereas *L. serriola* and *L. saligna* share a distinct cpDNA insertion. Our results highlight a complex origin of the organellar genomes of cultivated lettuce.

## Materials and Methods

### Plant Material and Growth Conditions

A collection of nine accessions (Supplementary Table 1) belonging to four different *Lactuca* species (*L. sativa*, *L. saligna*, *L. serriola*, and *L. virosa*) originating from diverse geographical origins were used in this study and seeds are available upon request. Seeds of all accessions were provided by the company Enza Zaden (Enkhuizen, Netherlands), and propagated to obtain sufficient seeds for mitochondria purification. All accessions are recorded in the company Genbank and were isolated either by Enza Zaden (*L. sativa* accessions LACWENDEL and LAC004500, *L. saligna* accession LAC008020, *L. serriola* accession LAC005780, *L. virosa* accession LAC006941) or originate from other collections (*L. serriola* accession CGN004799, *L. virosa* accessions CGN013357 and CGN019045 are from the Centre for Genetic Resources, Wageningen University and Research and *L. sativa* accession UC12100 is from the University of California, Davis).

Seeds were sterilized prior to growth by incubation for 30 s with 70% ethanol followed by 10 min with 1% NaOH, washed with sterile water and dried on blotting paper in a sterile flow cabinet. Sterilized seeds were placed on T-300 blotting paper (Ø 80 mm; All Paper b.v., Didam, Netherlands) in Pétri dishes and wet with 5 mL of sterile water. The Pétri dishes were sealed with parafilm to keep humidity and stratified for 2 days at 4°C to induce germination, followed by growth in the dark at 21°C up to 7 days.

### Mitochondrial DNA Extraction

Whole 7 days old *Lactuca* etiolated seedlings were used for mitochondria preparation. Briefly, about 1,000 seedlings are ground with a mortar and pestle in grinding buffer (0.6 M sucrose, 50 mM tetrasodium pyrophosphate, 4 mM EDTA, 20 mM KH2PO4, 2% PVP-40, 2% BSA, pH 7.5, 40 mM ascorbate, 10 mM cysteine, 2 mM DTT; 10 mL for 100 seedlings). The suspension is filtered through two layers of nylon mesh (96 μm) and the solid material is ground a second time in 10 mL of grinding buffer and filtered. The suspension is then fractionated by differential centrifugation to eliminate particles with low sedimentation coefficients (10 min at 3,500 *g*, twice 5 min at 3,500 *g*, 5 min at 6,000 *g*) followed by a final sedimentation of the mitochondria enriched fraction (20 min at 17,000 *g*). The pellet is then resuspended in washing buffer (0.3 M sucrose, 10 mM MOPS, 0.1% BSA, pH 7.5) and mitochondria purified on Percoll step gradients (18–25–50% representing 33–50–17% of the total gradient volume of 2.2 mL), at 25,000 *g* for 25 min on an Optima MAX-TL (rotor TLA 100.3) from Beckman Coulter (Brea, CA, United States). The purified mitochondria band is collected at the 25–50% Percoll interphase. After mitochondria purification, contaminant nuclear or plastid DNA bound to the mitochondria surface is removed by DNase treatment. For that mitochondria are resuspended in 100 μL of washing buffer containing 50 μg DNase I and 0.55 μM Mg2+ and incubated 20 min at 25°C. After incubation, 1 mL of washing medium containing EDTA and EGTA (both at 10 mM) is added and mitochondria sedimented at 18,000 *g* for 15 min. DNA extraction from purified mitochondria was performed using the *QIAamp*^®^ *DNA Micro* kit (Qiagen, Venlo, Netherlands) following the protocol “Isolation of Genomic DNA from tissues.”

### DNA Sequencing

For Illumina sequencing, library preparation and sequencing reactions were performed by the IBMP “gene expression analysis” platform. Libraries were prepared with the Nextera XT DNA Library Prep kit (Illumina, San Diego, CA, United States) and their size checked on a 2100 Bioanalyzer (Agilent, Santa Clara, CA, United States). The libraries were then sequenced with paired-end 2 × 151 reads on a MiSeq System (Illumina). The raw Illumina reads were deposited in GenBank under accession numbers ERS5266978 and ERS5267070 to ERS5267078.^1^ Pacbio sequencing of the mtDNA of *L. sativa* var. *capitata* L. *nidus tenerrima* (accession LACWendel) was performed by Keygene (Wageningen, Netherlands) on a Pacbio Sequel II instrument (Pacific Biosciences, Menlo Park, CA, United States). The sequencing generated 19.9 Gb from 3.2 million reads (average read size 3,051 pb). The raw PacBio reads were deposited in GenBank under accession number ERS5266954.^1^

### Sequences Alignments and Phylogenetic Analysis

From GenBank we obtained 88 *Lactuca* ITS1-*5.8S*-ITS2 sequences from eight species: *L. sativa*, *Lactuca dregeana*, *Lactuca indica*, *L. saligna*, *Lactuca scandens*, *L. serriola*, *Lactuca sibirica*, and *Lactuca virosa* (Supplementary Table 2). For the ITS1-*5.8S*-ITS2 sequences of accessions described in Supplementary Table 1 and from Kozik et al. (2019) the Illumina reads were mapped on *L. sativa* references (KT249801.1) with the Burrows-Wheeler Aligner bwa-mem (v07.7.17-r1188) (Li and Durbin, 2009) and mapped reads were assembled with *SPAdes* (v3.13.1) (Bankevich et al., 2012). Finally, assembled sequences were manually trimmed to match starts and ends of reference sequences. Sequence alignments were performed on *MEGA X* (v10.1.7) (Kumar et al., 2018; Stecher et al., 2020) using *MUSCLE* (Edgar, 2004) with default options. Maximum likelihood trees were also built on *MEGA X*, using the bootstrap method (1,000 replications) with default options and final trees were computed on the *Interactive Tree Of Life* (v6) (Letunic and Bork, 2019).

The plastid genomes assembly and comparison was performed using *MacVector* (v18.1.5) (MacVector INC., Apex, NC, United States) Assembler plugin’s. Illumina reads were mapped on the *L. sativa* cultivar Salinas cpDNA reference (AP007232.1) with the aligner Bowtie v1.3.0 (Langmead et al., 2009). Sequences were aligned with ClustalW 1.83 implemented in the MacVector package using default options. Regions of ambiguity that are duplicated in the mitochondrial genome were removed from the alignment. Maximum likelihood trees were built by the neighbor-joining method. The alignment is given as Supplementary Data.

Concatenated protein coding sequences of the mtDNA were manually assembled, from the annotations of the genomes. They were aligned with ClustalW 1.83 and maximum likelihood trees built by the neighbor-joining method, using the software implemented in the MacVector package. The alignment is given as Supplementary Data.

### Assembly of the Mitochondrial Genomes

The assembly of the mtDNA of *L. sativa* var. *capitata* L. *nidus tenerrima* combined Illumina and Pacbio reads. Contigs from Illumina MiSeq paired-end (2 × 150 bases) were built with the organellar *de novo* assembler *Novoplasty* (v2.4) (*k*-mer 22) (Dierckxsens et al., 2016). This led to 48 contigs, among them 22 were confirmed as mtDNA contigs by *BLAST* (Altschul et al., 1990). Repeated regions were identified as the overlap between four different contigs and particularly three large ones: R01 (34,696 bp), R02 (10,430 bp), and R03 (3,552 bp). Raw Pacbio data delivered by Keygene were mapped on the 21 mtDNA contigs from Illumina with bwa-mem (Supplementary Figure 1A). Mapped Pacbio reads were then assembled using Canu (Koren et al., 2017) to obtain four contigs. Among them three large ones were identified as mitochondrial by *BLAST* (v2.10.1) (Altschul et al., 1990): 177,077 bp (PB01), 90,502 bp (PB02), and 73,475 bp (PB03). Interestingly, the start of the PB01 sequence overlaps its end for 5,225 bp. Consequently, we circularized it into a circular chromosome (PBc01) of 171,852 bp. Furthermore, we found that the extremities of contigs PB02 and PB03 mapped to sequences internal to PBc01, corresponding to repeated regions identified among Novoplasty contigs. So we circularized PB02 and PB03 using repeats R01 and R02, respectively, into circular chromosomes PBc02 (112,968 bp) and PBc03 (78,504 bp). The circular chromosomes were then assembled through recombination-like events involving repeated sequences R01 and R02 into a single circular molecule of 363,324 bp. The code used for the assembly is available at github.com/ARNTET/Pacbio_Illumina_mtDNA_assembly.

The assembly of the *L. saligna*, *L*. *sativa*, *L. serriola*, and *L. virosa* mitochondrial genomes was from Illumina reads. We built our pipeline (Supplementary Figure 1B) based on a recent work from Garcia et al. (2019). Illumina reads from *Lactuca* species were trimmed with *Trimmomatic* (v0.39) (Bolger et al., 2014) to remove adapter sequences and low quality ends and joined with *Fastq-join* (v1.3.1)^2^ when paired reads mapped to each other. Then those reads were assembled into contigs with a first assembler: Velvet (v1.2.10) (Zerbino and Birney, 2008). Those contigs were mapped on the *L. sativa* var. *capitata* L. *nidus tenerrima* mtDNA sequence and mapped Velvet contigs were used as trusted contigs for a second round of assembly with SPAdes (v13.3.1) (Bankevich et al., 2012). SPAdes contigs were identified by *BLAST* (v2.10.1) (Altschul et al., 1990) and filtered according to their coverage. Filtered SPAdes contigs were extended with SSPACE (v1.12) into long scaffolds (up 170 kb). Finally, contigs were manually assembled into single circular molecules. The code used for the assembly is available at github.com/ARNTET/Illumina_plant_mtDNA_assembly.

### Annotation of the Genomes

*Lactuca sativa* mtDNA was first automatically annotated using GeSeq (v2.0.3) (Tillich et al., 2017) with default settings for circular mitochondrial genome and with *Diplostephium hartwegii* (KX063855.1) as 3rd party reference. Then the annotation was manually reviewed with *L. sativa* RNA-seq data (accessions SRR080725 and SRR085107). RNA-seq reads were mapped on *L. sativa* mtDNA using *Hisat2* (v2.1.0) (Kim et al., 2015) with reduced mismatch penalties (–mp parameter to “2.0”), allowing the mapping of more reads. SAM format files were then converted to BAM format with *Samtools* (v1.9) (Li et al., 2009). BAM files were sorted with Samtools for visualization on *IGV* (v2.8.12) (Robinson et al., 2011) to review CDS border. SNPs calling was made with *Freebayes* (v1.3.1) (Garrison and Marth, 2012), using default parameters. SNPs were filtered to remove non-C-to-U (or G-to-A) ones. Finally, only SNPs in coding regions were conserved and annotated as RNA editing sites. Code available at github.com/ARNTET/RNAseq_mapping_hisat2.

The *L. saligna*, *L. serriola*, and *L. virosa* mitochondrial genomes were automatically annotated using GeSeq (v2.0.3) (Tillich et al., 2017) with default settings for circular mitochondrial genome and with the *L. sativa* mtDNA and the recently published *L. serriola* US96UC23 and *L. saligna* CGN5271 *mtDNAs* (MK820672.1 and MK759657.1, respectively) as “3rd party references.” The repeated regions among the *Lactuca* mtDNA sequences were identified by using the python script *ROUSfinder* (v1.1) (Wynn and Christensen, 2019). We annotated the sequences larger than 50 bp from the largest (R01) to the smallest (R17). The nomenclature in our dataset is based on the one of *L. sativa*, homologous repeated regions in the others *Lactuca* species mitogenomes are annotated with the same number. Visualization and identification of blocks of synteny between the mtDNA genomes assembled in this work was done with *progressiveMauve* (v2.4.0) (Darling et al., 2010) with default options. The annotated mtDNA sequences have been deposited in GenBank under accession numbers MZ159953 (LsatUC12100), MZ159954 (LsatLACWENDEL), MZ159955 (LsatLAC004500), MZ159956 (LsalLAC008020), MZ159957 (LserCGN004799), MZ159958 (LserLAC005780), MZ159959 (LvirLAC006941), MZ159960 (LvirCGN013357), and MZ159961 (LvirCGN019045).

## Results

### Phenotypic Diversity Within a Collection of Nine Accessions of Cultivated and Wild Lettuce

**FIGURE 1:**
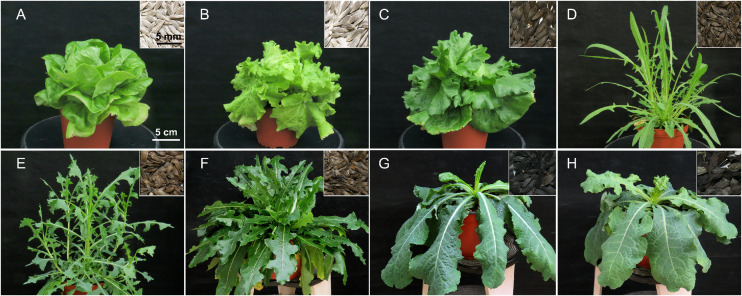
Phenotypes of the selected *Lactuca* at the rosette stage and corresponding seeds. **(A)** *L. sativa* LACWendel; **(B)** *L. sativa* LAC004500; **(C)** *L. sativa* US12100; **(D)** *L. saligna* LAC008020; **(E)** *L. serriola* CGN004799; **(F)** *L. serriola* LAC005780; **(G)** *L. virosa* CGN013357; **(H)** *L. virosa* LAC006941. *L. virosa* CGN019045 is not represented but it is similar to the two others *L. virosa* accessions, in terms of leaves and seeds shape and color.

For this study, we selected nine accessions of *Lactuca* spp. among those available at the Genbank of Enza Zaden (Enkhuizen, Netherlands): three of *L. sativa*, one of *L. saligna*, two of *L. serriola*, and three of *L. virosa*. The three *L. sativa* accessions represent two cultivars: LACWendel is a Butterhead lettuce (*L. sativa* var. *capitata* L. *nidus tenerrima*), and UC12100 and LAC004500 are Crisphead lettuce (var. *capitata* L. *salinas*). The accessions were selected to include the three wild *Lactuca* species believed to be at the origin of domesticated *L. sativa*, and covering a wide diversity of geographical origins (Supplementary Table 1). Accessions showed marked phenotypical variation according to the species, but also interspecies (Figure 1). At the seed level, seeds from *L. virosa* accessions are large and black, while *L. saligna* and *L. serriola* seeds are thinner and brown. The *L. sativa* LAC011481 seeds are also thin and brown, while those of *L. sativa* LACWendel and LAC004500 are white. The phenotypic variations are even stronger at the rosette stage. *L. sativa* accessions show upright spooned leaves while the leaves of the *L. virosa* accessions are long and droopy. The leaves of *L. serriola* plants are lobed shaped, but the ones *L. saligna* LAC00820 are upright needle-like.

### Nuclear Sequence Diversity Among Accessions

**FIGURE 2:**
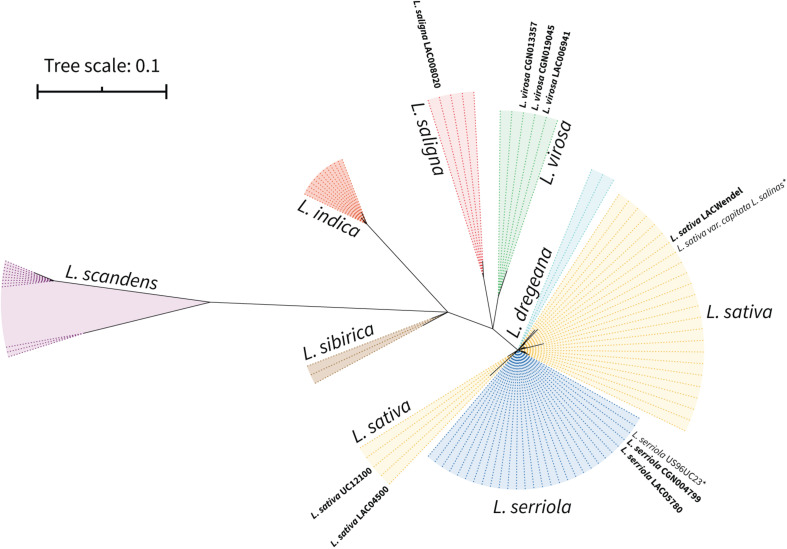
Phylogenetic relation of the selected *Lactuca* accessions according to nuclear marker (*ITS1-rRNA5.8S-ITS2*). Maximum likelihood phylogram of the sequences of internal transcribed spacers 1 and 2 and the *5.8S* rRNA. Bootstrap values are not shown but branches with BS <99 are collapsed. *Sequences assembled from Kozik et al. (2019) raw reads SRX5097892 (*L. serriola* US96UC23) and SRR577192 (*L. sativa* cv. *Salinas*).

We propagated the seeds of the selected accessions and prepared DNA from purified mitochondria, as described in “Materials and Methods.” The enrichment in mitochondrial DNA as compared to nuclear and plastidial DNA was evaluated by qPCR, using primer pairs specific for each genomic compartment: *18S* nuclear rRNA gene, *18S* mitochondrial rRNA gene, *NAD3* mitochondrial gene and *RBCL* plastid gene (Supplementary Table 3). For all samples the mtDNA was enriched more than 100-fold (up to ∼10,000-fold for LACWendel), except for *L. virosa* LAC006941 (∼80-fold), while the cpDNA was poorly enriched (∼2.5 to ∼20-fold). We successfully sequenced the mtDNA enriched samples from the nine selected accessions by Illumina MiSeq (2 × 151 bp). These Illumina reads were evaluated afterward with the corresponding mtDNA built bellow, and showed a proportion of mitochondrial reads from 57% for *L. sativa* UC12100 to 5% for *L. virosa* LAC006941, allowing coverages of 1362X to 86X, respectively (Supplementary Table 4). We also obtained Pacbio long reads from *L. sativa* LACWENDEL. Our aim was to build the sequence and structure of the mtDNA of the corresponding accession, which could be used later as scaffolds to build the structures of the other accessions. Because we lacked information on the accessions from our population, besides the name of the species and from where they had been collected, we first established the nuclear diversity within this population to confirm their identity. To do so we built the set of ribosomal ITS, ITS-1 and ITS-2, flanking the nuclear *5.8* rRNA gene sequence (640 bp), which has been previously used as nuclear marker to build phylogenetic relationships among *Asteraceae* species (Koopman et al., 1998). We did it for our selected accessions, from our Illumina reads, and for the *L. sativa* and *L. serriola* accessions from which the mtDNA was recently published and from which sequence data is available (accessions SRR080725 and SRR085107). We aligned the corresponding sequences to the 89 additional ones of *Asteraceae* species available on the NCBI nucleotide database (Supplementary Table 2). The alignment of these 100 sequences was 723 nucleotides long, comprising 404 conserved positions. The polyphyletic tree built using Maximum Likelihood from this alignment is shown in Figure 2. In this tree, our sequences branched as expected, in the same clusters as others from the same species. It is possible to distinguish every individual species, at the exception of *L. sativa* and *L. serriola*, which are unresolvable with this marker, as previously described.

This result confirmed the species identity of our selected accessions, and that *L. sativa* and *L. serriola* are closely related species that branch together and quite apart from *L. saligna* and *L. virosa*, at least at the nuclear level. On the other hand, our preliminary analysis of the nuclear sequences revealed that two accessions previously selected and annotated as *L. saligna* proved to be *L. sativa* accessions instead, highlighting the importance of previously confirming the identity of accessions obtained from germplasm banks.

### Mitochondrial Genomes of the Selected *Lactuca* spp. Accessions

**FIGURE 3:**
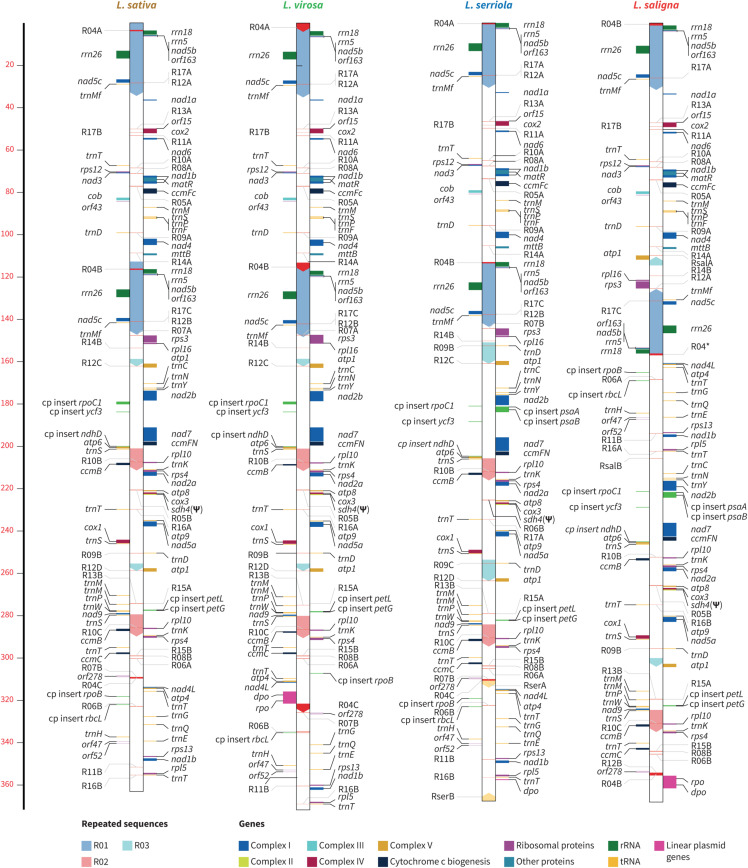
Linear representations of lettuces mitochondrial genomes. The relative positions of genes are indicated by horizontal lines, color coded by function, as indicated in the key at the bottom right. Transcription direction is down for genes on the right of the genome bar and up for those on the left. Large repeats (0.5 kb) are indicated by arrowed boxes within each genome bar, which are color coded according to the key at the bottom left. Plastidial DNA insertions (annotated as “cp insert”) are highlighted in green. Scale is in kilobases.

We assembled the mtDNA of *L. sativa* LACWENDEL by combining Illumina MiSeq (2 × 151 bp) and Pacbio long reads, as described in “Materials and Methods.” The mtDNA of *L. sativa* accessions LAC004500 and UC12100, of *L. saligna* LAC008020, of *L. serriola* accessions CGN004799 and LAC005780 and of *L. virosa* accessions CGN013357, CGN019045, and LAC006941 were assembled by using Illumina MiSeq (2 × 151 bp) data only, from highly purified mtDNA. The assemblies generated, in each case, single circular molecules that are depicted in linearized forms in Figure 3.

The three *L. sativa* mtDNA genomes are 363,324 bp long and are 100% identical, and perfect isoforms of the recently published (Kozik et al., 2019) mtDNA of *L. sativa* cultivar Salinas (MK642355.1). The switch from one isoform to the other possible ones requires homologous recombination events involving the large repeated sequences R02 and R03, which are 10,430 and 3,553 bp long, respectively. A dynamic structure by recombination involving large repeats is a known and expected feature of plant mtDNA genomes. The mtDNA of *L. saligna* LAC008020 is 368,269 bp long. The structure that we obtained using our assembly pipeline is the perfect isoform to the recently published mtDNA of *L. saligna* CGN5271 (MK759657.1; Kozik et al., 2019). The two *L. serriola* mtDNAs that were assembled (accessions CGN004799 and LAC005780) are 367,647 bp long and identical, both in sequence and in structure. However, they diverge significantly from the recently published mtDNA of *L. serriola* accession US96UC23 (MK820672.1) which is 363,328 bp long (Kozik et al., 2019) and that seems to be an isoform of *L. sativa* mtDNA, with no sequence differences, while the mtDNAs of CGN004799 and LAC005780 are significantly different from *L. sativa*.

The mtDNAs of the three *L. virosa* accessions that were assembled (accessions CGN013357, CGN019045, and LAC006941) are 373,019 bp long. CGN013357 and LAC006941 are 100% identical and diverge from CGN019045 by two SNPs at positions 190,612 (C to A) and 190,615 (G to T). These positions are ∼600 bp downstream *nad7* in a non-coding region. Consequently, only one genome per species is represented in Figure 3.

These genomes are not only similar in size but also in gene content (Supplementary Tables 5, 6). Indeed, the mtDNAs of *L. sativa*, *L. saligna, L. serriola*, and *L. virosa* all carry the same mitochondrial protein and rRNAs genes (Supplementary Table 6). The only difference in coding content concerns tRNA genes, more specifically one additional copy of the *trnD*(GUC) gene of plastidial origin which is found in the mtDNA of *L. serriola*. Mapping of available lettuce RNA-seq data (*SRR080725* and *SRR085107*) confirmed expression of all protein and rRNA gene sequences. It also identified seven expressed *orf*s (*orf15*, *orf43, orf47, orf52a, orf52b, orf163*, and *orf278*) in the *L. sativa* mtDNA. Several of them seem to be co-transcribed with conventional mitochondrial genes; *orf15* with *cox2, orf43* with *cob, orf163* with *nad5.* The sequences and flanking regions of these *orf*s are conserved in all *Lactuca* mtDNAs that we built. Therefore, although there is no RNA-seq data for *L. saligna*, *L. serriola*, or *L. virosa*, these *orfs* are probably also transcribed in those species.

The RNA-seq data was also used to identify 738 RNA-editing sites in gene coding sequences. Among these sites, the one at position 245,933 (A**C**G) creates the initiation codon (A**U**G) of *cox1*, in 89% of the reads. The open reading frame of *rpl16* overlaps with the 3′-end of *rps3*, like in all higher plant mtDNAs already sequenced (Newton et al., 1996). It was therefore believed that translation of *rpl16* is initiated internally to the *rps3* sequence, but in a different frame (Takemura et al., 1992). However, it has been recently reported in *Solanum tuberosum* that an editing site that transforms an U**C**A serine codon into an U**U**A leucine codon in *rps3* creates an internal **U**AG stop codon in the overlapping *rpl16* reading frame, and that translation of *rpl16* should initiate downstream of *rps3* (Varré et al., 2019). We could identify the exact same event in *L. sativa* (editing site at position 151,176), thus confirming the strong conservation of this editing site and its essential importance for the correct expression of *rpl16*. The recent determination of the structure of the plant mitochondrial ribosome (Waltz et al., 2020) showed that the mature rpl16 protein apparently starts at codon 37 of the corresponding *orf*, consistent with an initiation of translation at the GTG codon that is just upstream, as it was proposed (Varré et al., 2019).

The same recent publication about the *S. tuberosum* mtDNA suggested methylation of the mitochondrial 18S rRNA (Varré et al., 2019). This hypothesis relies on RNA-seq data showing that in 55% of reads a T is found at base 960, while an A is found in the corresponding genomic sequence. Such an event is diagnostic for the mis-incorporation of a T in front of an m1A nucleotide, during cDNA synthesis (Cognat et al., 2017). We observed the same event in *L. sativa*, where the A at position 961 of the 18S rRNA gene sequence (position 4,757 in the mtDNA) corresponds to a T in 48.5% of the RNA-seq reads. Because the methylation of the 18S rRNA affects a nucleotide in close vicinity of the tRNA anticodon at position P of the ribosome this methylation might be important for proper tRNA positioning and efficient translation. Our results show that methylation of the 18S rRNA at this position is conserved in higher plants, and are in line with the hypothesis of the important role of this post-transcription modification in the regulation of plant mitochondrial translation. Surprisingly, both in *S. tuberosum* and in *L. sativa* the modification is found in roughly half of the 18S molecules, suggesting the existence of two populations of ribosomes.

The mtDNAs of *Lactuca* species also show differences regarding insertions of exogenous sequences. In the genome of *L. saligna* LAC008020, a sequence is found that includes a Type B2 superfamily DNA polymerase and a T3/T7 phage-type RNA polymerase (*dpo*, 3,144 bp, and *rpo*, 2,808 bp, on Figure 3). The same sequence was described in the mtDNA of *L. saligna* CGN5271 (Kozik et al., 2019). These sequences resemble those found in mitochondrial linear plasmids described in other plant species, which at a certain time in evolution physically integrated into the mitochondrial genome (Warren et al., 2016). Interestingly, we identified the same genes in the mtDNA of the three *L. virosa* accessions (*dpo*, 2,831 bp and *rpo*, 2,805 bp). However, only fragments of *rpo* (319 bp) remain in the *L. serriola* mtDNA, indicating that these apparently pseudogenes were lost at a certain time during the evolution of *Lactuca* species. These sequences are no longer found in the mtDNA of *L. sativa* accessions.

**FIGURE 4:**
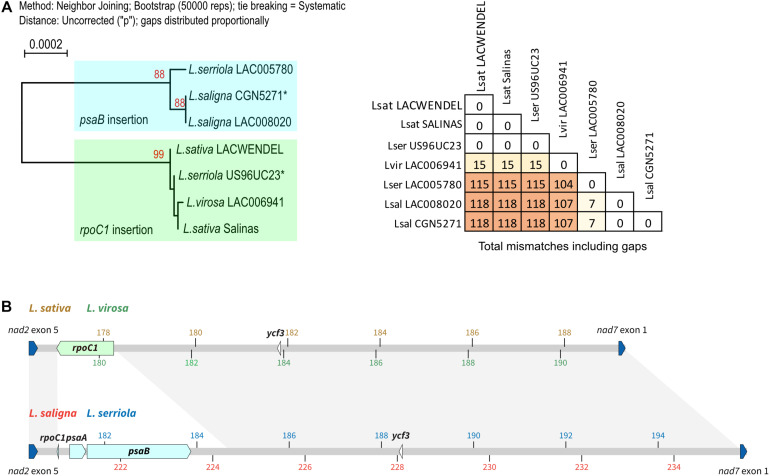
Phylogeny of the mtDNA correlates with different plastidial DNA insertions. **(A)** Maximum likelihood phylogram based on the alignment of the concatenated protein coding sequences. Bootstrap values are indicated, as well as the table with the number of SNPs and gaps. Sequences labeled with an asterisk (*) are from Kozik et al. (2019). **(B)** The region between exon 5 of *nad2* (last exon of the *nad2b* transcript) and exon 1 of *nad7* (blue boxes) in the mtDNA of *L. sativa, L. saligna, L. virosa*, and *L. serriola* are represented with their plastidial DNA insertions (green boxes). Above is shown the context in *L. sativa* and *L. virosa*, with coordinates in brown and green, respectively. Below in *L. serriola and L. saligna*, with coordinates in blue and red, respectively.

A phylogeny tree was built based on the alignment of the concatenated protein coding sequences, presented in Figure 4A that also includes a table with the number of SNPs and gaps. It shows that there are only 15 SNPs/indels between *L. sativa* and *L. virosa* mtDNA coding sequences, eightfold less than between *L. sativa* and *L. serriola* or *L. saligna.* Thus, the mtDNA of *L. sativa* is more closely related to the mtDNA of *L. virosa* than to the mitogenomes of the other *Lactuca* species. As described above, the mtDNA of *L. serriola* accession US96UC23 is surprisingly identical to the mtDNA of *L. sativa*.

A second major divergence between the *Lactuca* mtDNA sequences involves plastid DNA insertions. All genomes share six identical plastid DNA insertions that are from *ndhD* (194 bp), *petG* (116 bp), *petL* (81 bp), *rbcL* (198 bp), *rpoB* (57 bp), and from *ycf3* (72 bp). However, they also have an insertion that is variable between species. The insertion corresponds to a large piece of the plastidial *rpoC1* gene (1,239 bp) in *L. sativ*a and *L. virosa*, while in the *L. saligna* and *L. serriola* accessions a different insertion is found at the same position, just retaining 15 bp of the *rpoC1* gene but now further comprising *psaA* (354 bp) and *psaB* (2,253 bp) gene sequences (Figure 4B). The architecture of these two alternative plastidial insertions suggests two independent insertions events during the evolution of the mtDNA. A first one would be the insertion of the large piece of the *rpoC1* gene, while a second event, apparently involved recombination between the *rpoC1* sequence and another piece of plastidial DNA sharing sequence similarity, resulting in the replacement of most of the *rpoC1* sequence by the new insertion. These two different plastidial DNA insertions are interesting markers of *Lactuca* mtDNA evolution. They further support the closer phylogenetic relationship between the mtDNA genomes of *L. sativa* and of *L. virosa.*

The content in repeated sequences also diverges between the different species (Supplementary Table 7). The mtDNA genomes that we assembled contain 17 pairs of repeated sequences larger than 50 bp, in *L. sativa* and *L. virosa*, and 18 in *L. saligna* and *L. serriola*. All genomes bear a large repeated sequence (30,534–34,705 bp) containing the three rRNA genes. The large pair of repeats R02 (10,430 bp) is also found in all the mtDNAs that we built. It is extremely conserved and diverges only in *L. saligna* LAC008020, where it has an insertion of three nucleotides. The two accessions of *L. serriola* show a larger repeat R03 (10 kb) which contains the smaller repeat R03 found in *L. sativa*, *L. virosa* (3.5 kb) and *L. saligna* (4.0 kb). The case of repeat R04 is particular because it is present as three copies in the genomes, two of which are part of the very large repeat R01. It is variable in size in the different genomes (from 576 bp in *L. sativa* and *L. serriola*, up to 4,116 pb in *L. virosa*), but in all species it comprises 320 bp from the *18S* rRNA gene.

The interpretation of these results is not as clear as the one based on nuclear markers, which show that *L. sativa* and *L. serriola* are the closest species. On one hand, the loss of *dpo* and *rpo* from the mtDNA of both species and the same size of repeated sequence R04 could let us think that the *L. sativa* mtDNA is closer to the one of *L. serriola*. But on the other hand, the mtDNA of *L. sativa* is closer to the one of *L. virosa* in sequence, by the size of the repeated sequence R03, and by the presence of the *rpoC1* plastid insertion.

### *Lactuca* mtDNA Structure Diversity

**FIGURE 5:**
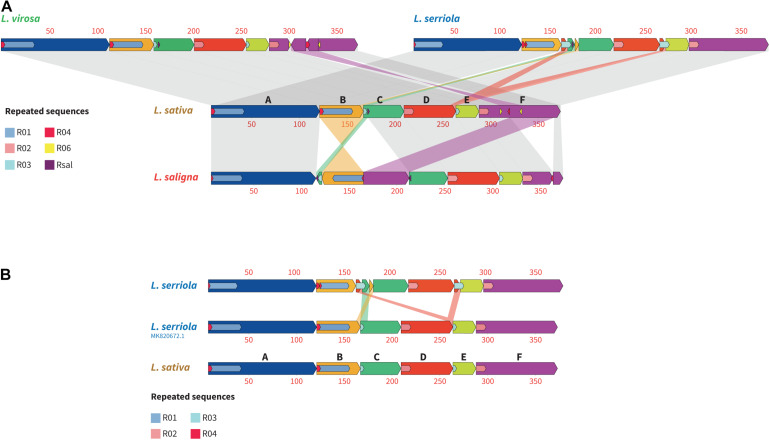
Structural diversity between the mitochondrial genomes *of Lactuca* spp. Alignments between *Lactuca* mtDNAs made with *progressive Mauve*. **(A)** Between *L. sativa, L. saligna, L. virosa* and *L. serriola*. **(B)** Between *L. sativa*, the *L. serriola* mtDNAs built in our study and the mtDNA of *L. serriola* US96UC23 published by Kozik et al. (2019). Homologous regions delimited by repeated regions are shown in the same color according to the six blocks (A–F) that subdivide the *L. sativa* mtDNA. Repeated sequences involved in structural evolution between the genomes are represented, according to the key on the left. Scale is in kilobases.

To study the structural diversity of these *Lactuca* mtDNA genomes and the possible events that resulted in their divergence, we aligned them according to the six blocks that subdivide the *L. sativa* mtDNA (Figure 5A, regions A–F in the representation of the *L. sativa* mtDNA). Through this alignment it is clear that the mtDNA from *L. saligna* has the highest structural diversity to *L. sativa*. The alignment highlighted a recombination event in *L. saligna* involving an intermediate-size repeated sequence, Rsal of 122 bp. The sequence of this repeat can be also found in the mitogenomes of *L. sativa, L. serriola*, and of *L. virosa*, but only as a single-copy sequence. We also found that the mtDNAs of *L. sativa* and of *L. serriola* differ in ways that cannot be explained by simple homologous recombination events involving repeated regions. Indeed, as represented in Figure 5A, block C (dark green) in *L. serriola* has an insertion coming from block B, a genomic rearrangement that we could not find associated with any repeated sequence or microhomology. Moreover, as discussed above, *L. serriola* has a much larger repeat R03 (10,137 bp as compared to 3,553 bp in *L. sativa*), an extension that came from the 3′ end of the block D. However, these differences between the mtDNAs of the *L. serriola* accessions that we sequenced and the mtDNA of *L. sativa* are not shared with the recently published mtDNA of *L. serriola* US96UC23, which is apparently a simple isoform of the *L. sativa* mtDNA (Figure 5B).

In fact, the mtDNA that is the closest in structure to *L. sativa* is the one of *L. virosa* (Figure 5A). Indeed, the only structural divergence between these two mitogenomes is a sequence inversion in the middle of block F, which involves the intermediate-size repeated regions R06 (186 bp). The sequence delimited by this pair of inverted repeats is also larger in *L. virosa* (30,576 bp long in *L. virosa* as compared to 21,320 bp in *L. sativa*). This extension in *L. virosa* carries the *dpo* and *rpo* sequences of apparently viral origin. Consequently it is quite possible that these gene sequences were inserted along other exogenous sequences in the mtDNA by horizontal transfer, explaining the size difference. Thus, in terms of the mitochondrial genome structure, these comparisons show that *L. sativa* is closer to *L. virosa*.

### Chloroplastic Diversity Among the *Lactuca* Accessions

Because the mitogenomes of *L. sativa* and *L. virosa* are closely related, in contrast to their nuclear sequences, we considered the possibility that the organellar genomes, which are predominantly maternally inherited, had a different origin. In that case it would be expected that the mitochondrial and plastidial genomes show the same phylogenetic origin. We have therefore tested the chloroplastic diversity among the accessions.

**FIGURE 6:**
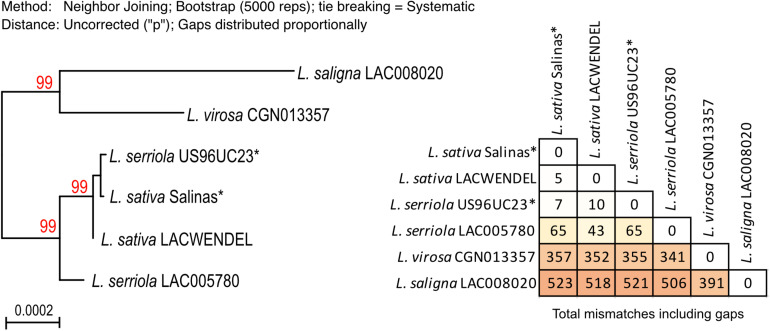
Phylogenetic relation between the plastid genomes of the selected *Lactuca* species. Maximum likelihood phylogram based on the alignment of the complete cpDNA genomes assembled form the same sequences datasets used to assemble the mitogenomes. Bootstrap values are shown in red and branches with BS <80 are collapsed. Sequences labeled with an asterisk (*) were assembled with raw reads from Kozik et al. (2019), SRX5097892 (*L. serriola* US96UC23) and SRR577192 (*L. sativa* cv. *Salinas*). The table with the number of SNPs and gaps is given.

To do so, we build the complete plastidial genome sequences for the four accessions that were sequenced in this study (*L. saligna* LAC008020, *L. sativa* LACWendel, *L. serriola* LAC005780, and *L. virosa* CGN013357), and for the *L. sativa* cv. *Salinas* and *L. serriola* US96UC23 accessions from which the mtDNAs have been recently published (Kozik et al., 2019). The alignment of these six *Lactuca* chloroplast genomes, excluding the second copy of the inverted repeat, was 127,738 nucleotides long, comprising 126,686 conserved positions. The polyphyletic tree using Maximum likelihood from this alignment is shown in Figure 6. In this tree, the cpDNA sequences branched as for the nuclear marker (Figure 3). It is possible to distinguish *L. saligna* and *L. virosa* from *L. sativa* and *L. serriola*, which fall in the same branch of the phylogenetic tree. The number of SNPs and gaps between the cpDNA sequences are also indicated in the figure, showing that there are about eightfold more SNPs/gaps between the cpDNAs of *L. sativa* and *L. virosa* than between the cpDNAs of *L. sativa* and *L. serriola*, supporting the conclusion that the cpDNA of *L. sativa* is indeed closer in evolution to the one of *L. serriola.* Regarding the cpDNA that was assembled from the *L. serriola* accession US96UC23 raw reads, it is surprisingly virtually identical to the cpDNA genomes of *L. sativa.* This analysis of the plastidial genome sequences surprisingly suggests divergent origins of the organellar genomes of *L. sativa*. The possible causes for such observation are discussed below.

## Discussion

### Mitochondrial Diversity Is Low Within *Lactuca* spp.

In this study we assembled the mtDNA genomes of nine *Lactuca* spp. accessions. Our assembly of the mtDNA of *L. sativa* Butterhead lettuce (var. *capitata* L. *nidus tenerrima*) is robust, based on Illumina short reads and PacBio long reads, which allowed us to infer one of the major isoform structures of the mtDNA. During this work the mtDNA of a Crisphead lettuce accession was published by Kozik et al. (2019), corresponding to the same genome in a different isomeric form. The concordance of the two independent results validated our assembly. We further built eight additional mtDNA genomes of other accessions and species, from Illumina sequence data obtained from purified mitochondria. The assemblies were based on the possible order of scaffolds inferred from overlapping sequences. We validated our assembly pipeline on *L. sativa*, because from just Illumina sequence data we obtained the same genome structure as the one assembled with the help of PacBio long reads. Our assembly of the mtDNA of *L. saligna* LAC008020 further supported the validity of our assembly pipeline, because the structure we inferred is a perfect isoform to the published mtDNA of *L. saligna* CGN5271 (Kozik et al., 2019). We are therefore confident that the structures we present are valid, and correspond to one of the major isoforms of the mtDNA, interconvertible to other possible ones by recombination involving the large repeated sequences.

Unlike what we expected, we found very little mitochondrial diversity at both the interspecies and intraspecies levels in this panel. This is surprising, when considering the very distant geographical origins of the accessions (Supplementary Table 1). But the geographical origins might just reflect the recent human-mediated dispersal of the species, distorting the picture of their natural geographical range and of the origin of the domesticated species. Indeed, at the intraspecies level we found no differences between the mitogenomes of all *L. sativa* accessions analyzed, although they belong to different typologies, nor among the three *L. virosa* accessions, or between the two *L. serriola* accessions we sequenced. The mtDNA of the *L. saligna* we sequenced and assembled is also identical to the one of the *L. saligna* accession published by Kozik et al. (2019). This is in contrast with what was found in other species, such as Arabidopsis, *Brassica napus* or maize, in which the mitogenomes from different subspecies or even different accessions can vary significantly in organization. We did not expect major differences between species with respect to the mtDNA coding content, because the content of plant mitogenomes varies barely interspecies and differences are further reduced when comparing evolutionary close species. The gene sequences were also expected to be highly conserved, because apart from some notorious genus such as *Silene* and *Plantago* (Mower et al., 2007; Sloan et al., 2012), the sequences of higher plant mitochondrial genes evolve very slowly (Palmer and Herbon, 1988). Still, apart from the re-organization of genomic regions, we found very few SNPs or indels between the mitogenomes of the *Lactuca* species we studied. The little mitochondrial diversity at the interspecies and intraspecies levels indicates recent divergence of these species and accessions. However, a demographic history inference based on nuclear SNPs suggested that cultivated lettuce likely originated from the Fertile Crescent more than 12,000 years ago (Zhang et al., 2017). And while *L. saligna* and *L. serriola* have geographical origins overlapping with the presumed origin of domesticated *L. sativa*, which is South-West Asia, *L. virosa* has a distinct geographical origin (Kesseli et al., 1991).

## Divergence of mtDna Sequences and Structures Among *L. serriola* Accessions

The major intraspecies differences we found concerned the mtDNA of the *L. serriola* accessions we assembled (CGN004799 and LAC005780) and the one of *L. serriola* US96UC23 that was published by Kozik et al. (2019). Its structure is very different from the ones of CGN004799 and LAC005780 (Figure 5B). It is also different in sequence, as the mitogenomes of CGN004799 and LAC005780 carry one additional copy of the *trnD*(GUC) gene of plastidial origin, which is absent in US96UC23 (Supplementary Table 6). Most significant, the mtDNA of *L. serriola* US96UC23 carries the same plastidial *rpoC1* insertion that is found in the mtDNAs of *L. sativa* and of *L. virosa*, while at the same position the mitogenomes of the two other *L. serriola* accessions contain instead the *psaA-psaB* insertion that is also found in the mtDNA of *L. saligna*. Moreover, the mitogenome of *L. serriola* US96UC23 does not contain any remaining sequences of the *dpo-rpo* insertion, like in *L. sativa*. Thus, the structure and sequence of the mitogenome of *L. serriola* US96UC23 is very different from the mtDNAs of CGN004799 and LAC005780 and rather looks like the mtDNA of *L. sativa* (Figure 5B). These observations and their implications are further discussed below.

### Analysis of the Lettuce Mitogenomes Suggest an Important Role of *L. virosa* in Lettuce Diversification

The involvement of the different *Lactuca* species in the domestication and/or diversification of *L. sativa* is not clearly understood. From a nuclear genome point of view *L. serriola* is considered as one of the direct ancestors and the closest relative to *L. sativa* (de Vries, 1990; de Vries and van Raamsdonk, 1994). A recent study based on nuclear RNA-seq analysis further supported the hypothesis that all typologies of cultivated lettuce originated from a single domestication event and a common ancestor, which was a wild *L. serriola* or an already domesticated one (Zhang et al., 2017). Further diversification of *L. sativa* would have led first to the Butterhead typology and later to Crisphead lettuces (Zhang et al., 2017). Our results on the nuclear and chloroplastic sequence diversity among our accessions are in line with this assumption. Indeed, according to these results, *L. saligna* and *L. virosa* are distant from the pair *L. serriola-L. sativa*. Consequently, the role of the others *Lactuca* spp. seemed limited to the development of new lettuce cultivars (Zhang et al., 2017). Indeed, intraspecies crosses have been used by breeders for the development of new cultivars, and a relevant example is found in the genealogy of North American lettuces, which showed that current Crisphead cultivars can be split into two groups, one derived from cultivars where *L. virosa* was introgressed in *L. sativa* to breed for robust root system and decreased leaf drop, and a second group that is not *L. virosa* derived (Mikel, 2007). But besides the development of current cultivars, no involvement of *L. virosa* on Lettuce domestication has been described, and was also considered unlikely because it has a different geographical center of origin than *L. serriola* and *L. sativa* (Lebeda et al., 2004).

In this work, we have for the first time assembled mitogenomes from *L. virosa.* We have also built the mtDNA of three additional *L. sativa* accessions. Among these, two (LAC004500 and UC12100) are established and documented as Crisphead lettuce, respectively, while the third (LACWendel) is a Butterhead lettuce. Unexpectedly, both in sequence and structure we found higher similarity between the mtDNAs of *L. virosa* and *L. sativa*, including Crisphead and Butterhead lettuces, than with *L. serriola* and *L. saligna.* Most significantly, the *L. virosa* and *L. sativa* mitogenomes share the same chloroplastic insertion, while a different one is found at the same locus in *L. serriola* and *L. saligna*. The possibility that a same event of horizontal transfer happened twice in evolution is unrealistic. Moreover, the alignment of the concatenated mtDNA protein coding sequences supported that the *L. sativa* mtDNA is closer to the one of *L. virosa* than to the ones of *L. serriola* (CGN004799 and LAC005780) or *L. saligna* (Figure 4A).

This closeness between the mtDNAs of *L. virosa* and *L. sativa*, of both Crisphead and Butterhead typologies, suggests that cultivated lettuce might be derived from an ancestral cross where *L. virosa* was the female partner that transmitted its mitogenome, and that consequently *L. virosa* played a more important role in *L. sativa* domestication than previously suspected. Backcrosses would have cleaned the actual *L. sativa* nuclear genome from most *L. virosa* sequences, as observed at present (Zhang et al., 2017). However, this scenario does not explain the heterogeneity of mitogenomes observed among *L. serriola* accessions, with the one of *L. serriola* US96UC23 almost identical to the one of *L. sativa*. The cpDNA genome assembled from the same Illumina sequences data set is also virtually identical to the cpDNA of *L. sativa.* This observation calls for the hypothesis that this sequence data set originates from a *L. sativa* accession mislabeled as *L. serriola.* An alternative possibility is that *L. virosa* had previously been hybridized with *L. serriola*, leading to two different *L. serriola* groups; a wild one harboring a mtDNA distinct from the one of *L. virosa* and a second group derived from the hybrid, which was later the precursor of cultivated *L. sativa*.

### The Divergence Between cpDNA and mtDNA Suggests Paternal Leakage

The hypothesis according to which *L. virosa* was introgressed as a cytoplasm donor during *L. sativa* diversification can explain why the mitogenomes of the two species are so similar. However, it does not fit with the phylogeny based on the chloroplast genomes alignment. Indeed, while the mtDNA of *L. sativa* is closer to the one of *L. virosa*, its cpDNA is closer to the cpDNA of *L. serriola*. In most flowering plant species, lettuces included, the mtDNA and cpDNA are both maternally transmitted and should follow the same inheritance. The apparent divergent origins of the organellar genomes could be because one was transmitted paternally by *paternal leakage*, meaning that during a cross organelles and their genomes were transmitted *via* the pollen.

Plastids and the cpDNA are maternally inherited due to the degeneration/exclusion of plastids from the pollen generative cell, or because elimination of paternal plastids during fertilization (Hagemann and Schröder, 1989; Mogensen, 1996). But a low-frequency leakage of plastids is commonly admitted in plants. In *A. thaliana*, plastids are absent in pollen sperm cells and consequently only maternally inherited (Martínez et al., 1997; Nagata et al., 1999). The frequency of paternal plastid transmission was evaluated as low as 1.9-3.9 × 10^–5^ (Azhagiri and Maliga, 2007; Schneider et al., 2015). Other studies described similar low frequencies in other species, like in *Setaria italica, Brassica napus*, and *Antirrhinum majus* (Diers, 1967; Wang et al., 2004; Schneider et al., 2015). However, because plastid diversity follows nuclear diversity within the *Lactuca* population, with the couple *L. sativa/L. serriola* distinct from the couple *L. saligna/L. virosa*, it appears that if paternal leakage did indeed occur, it involved the mitochondrial genome.

**FIGURE 7:**
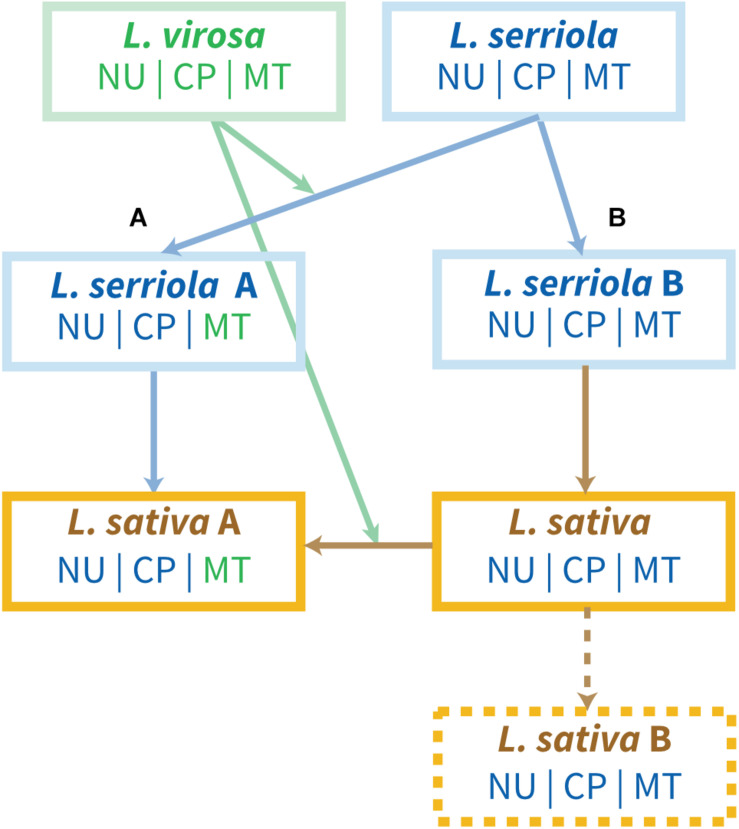
Hypothesis for the introgression of *L. virosa* mtDNA in *L. sativa*. Hypothesis **(A)** postulates a single introgression of *L. virosa* before the domestication of *L. sativa*. Hypothesis **(B)** involves introgression of *L. virosa* during the diversification of *L. sativa*. *L. serriola* US96UC23 could correspond to *L. serriola* “A”; *L. serriola* CGN004799 and LAC05780B to *L. serriola* “B”; *L sativa* “A” corresponds to all *L. sativa* mtDNA sequences available to date; *L. sativa* “B” remains hypothetical. NU, nuclear genome; CP, cpDNA; MT, mtDNA. Their origins are color-coded.

Mitochondrial paternal leakage events have been highlighted in natural populations and linked with heteroplasmy in *Silene acaulis* (Delph et al., 2007), *Silene vulgaris* (Wade and McCauley, 2005) or in *Daucus carota* ssp. *carota*, a weedy relative to domesticated carrot (Mandel et al., 2012). Preferential paternal transmission of the mitochondrial genome has been described in melon (*Cucumis melo*) and cucumber (*Cucumis sativus*), with rare maternal and biparental transmission (Havey et al., 1998; Shen et al., 2019). But there is no evidence of paternal mtDNA transmission in *Lactuca* spp. If we agree on an ancestral event of mitochondrial paternal leakage within the *Lactuca* population, and considering the heterogeneity of the mitogenomes among *L. serriola* accessions, we can consider an alternative hypothesis to explain the role of *L. virosa* in the evolution of the *L. sativa* mitogenome (Figure 7). This hypothesis relies on introgression of *L. virosa* either before the domestication of *L. sativa*, during a cross with an ancestral *L. serriola*, or later during the diversification of *L. sativa*, which would have led to mitochondrial paternal leakage. Paternal leakage would result in heteroplasmic mtDNA in the cell, and it has been shown that the coexistence of two mitogenomes results in recombination and sorting of a new predominant mtDNA containing pieces of the original genomes (Sanchez-Puerta et al., 2015). That could explain why the *L. sativa* mtDNA has certain regions that seem to be closer to *L. serriola*, like the loss of *dpo* and *rpo* and the size of repeated sequence R04, and other regions that are clearly related to the mtDNA of *L. virosa*, such as the cpDNA insertion. Later, the progeny of this hybridization, carrying nuclear and cpDNA genomes still related to *L. serriola* and an mtDNA mostly derived from *L. virosa* would have been used in *L. sativa* domestication. A future analysis of the mtDNA variability among *Asteraceae* should support or disprove this hypothesis. Our present study was restricted to a few accessions of a just a few species of the genus *Lactuca*, the ones most often used in crosses for the diversification of *L. sativa*, and a more comprehensive collection of *Lactuca* species is needed to achieve a clearer picture of the evolution of their mitogenomes. The study of a broader collection of *L. sativa* accessions covering all know typologies would also help to shed light on when its mtDNA diverged from the one of its *L. serriola* ancestor. Extraction of the mtDNA sequences from the recently described data set of 445 accessions (Wei et al., 2021) seems to be an obvious next step.

## Data Availability Statement

The datasets presented in this study can be found in online repositories. The names of the repository/repositories and accession number(s) can be found below: https://www.ncbi.nlm.nih.gov/genbank/, MZ159953; https://www.ncbi.nlm.nih.gov/genbank/, MZ159954; https://www.ncbi.nlm.nih.gov/genbank/, MZ159955; https://www.ncbi.nlm.nih.gov/genbank/, MZ159956; https://www.ncbi.nlm.nih.gov/genbank/, MZ159957; https://www.ncbi.nlm.nih.gov/genbank/, MZ159958; https://www.ncbi. nlm.nih.gov/genbank/, MZ159959; https://www.ncbi.nlm.nih. gov/genbank/, MZ159960; https://www.ncbi.nlm.nih.gov/genbank/, MZ159961; https://www.ncbi.nlm.nih.gov/genbank/, ERS5267070; https://www.ncbi.nlm.nih.gov/genbank/, ERS5267071; https://www.ncbi.nlm.nih.gov/genbank/, ERS5267072; https://www.ncbi.nlm.nih.gov/genbank/, ERS5267073; https://www.ncbi.nlm.nih.gov/genbank/, ERS5267074; https://www.ncbi.nlm.nih.gov/genbank/, ERS5267075; https://www.ncbi. nlm.nih.gov/genbank/, ERS5267076; https://www.ncbi.nlm.nih. gov/genbank/, ERS5267077; and https://www.ncbi.nlm.nih.gov/genbank/, ERS5267078.

## Author Contributions

AF, EG-F, G-JB, and JG designed the experiments. AF, SK, and EG-F performed the experimental work.

## Conflict of Interest

G-JB and EG-F were employed by the company Enza Zaden. The remaining authors declare that the research was conducted in the absence of any commercial or financial relationships that could be construed as a potential conflict of interest.

## Publisher’s Note

All claims expressed in this article are solely those of the authors and do not necessarily represent those of their affiliated organizations, or those of the publisher, the editors and the reviewers. Any product that may be evaluated in this article, or claim that may be made by its manufacturer, is not guaranteed or endorsed by the publisher.

## Footnotes

1 www.ebi.ac.uk/ena

2 http://github.com/ExpressionAnalysis/ea-utils
