# Supplementary material for: Sequence of the Mitochondrial Genome of Lactuca virosa Suggests an Unexpected Role in Lactuca sativa’s Evolution
Source: Front Plant Sci. 2021 Jul 26;12:697136. doi: 10.3389/fpls.2021.697136 (PMC8350775; doi:10.3389/fpls.2021.697136)
Supplement: Supplementary file 1 [file Data_Sheet_1.PDF]

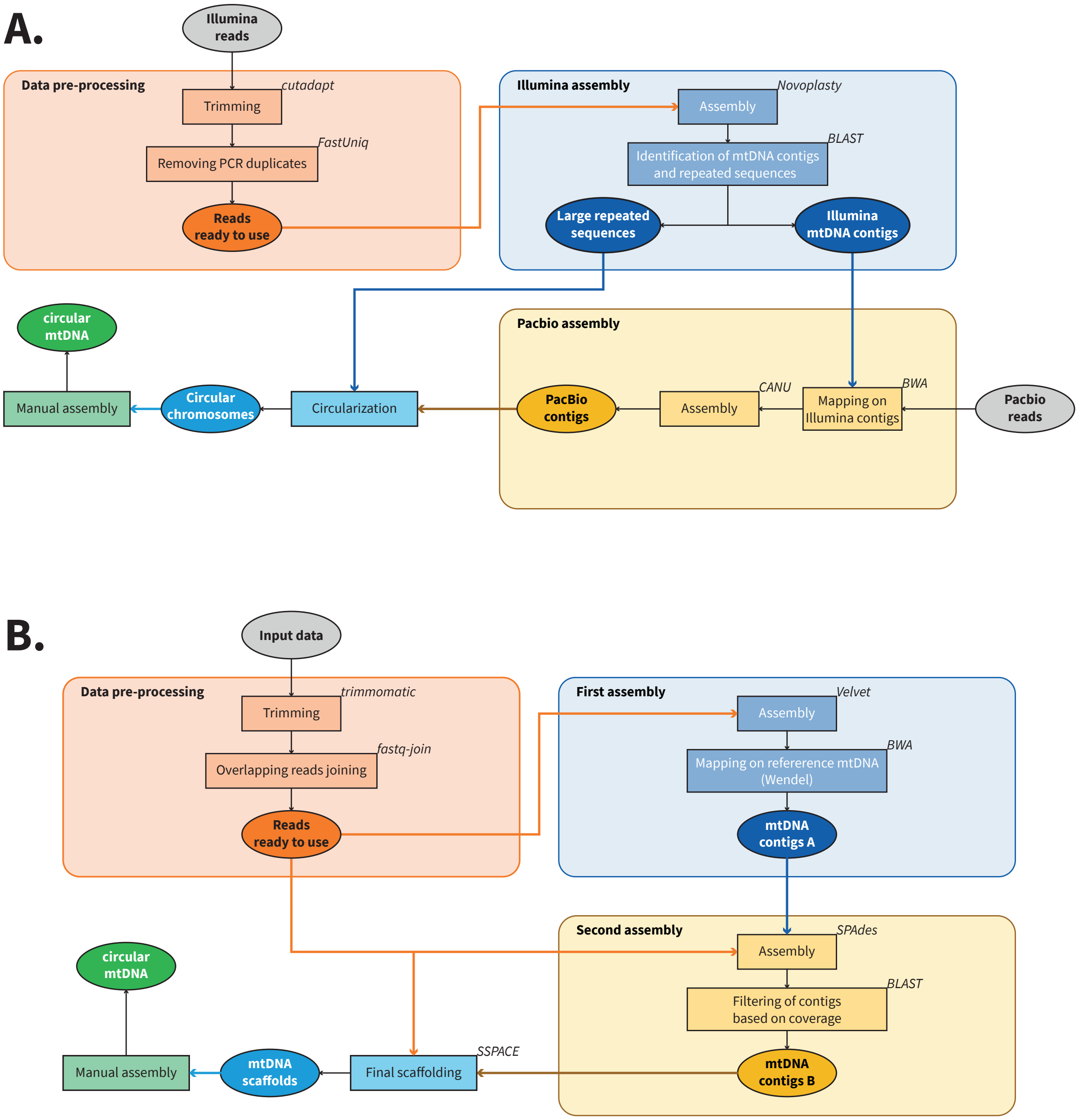

Supplemental Figure S1. Pipelines used for mtDNA assemblies. (**A**) Pipeline used for the assembly of the mtDNA of *L. sativa* var. *capitata* L. *nidus tenerrima* (LACWendel), combining Illumina and Pacbio reads. (**B**) Pipeline used for the assembly of the mtDNAs of the additional *L. saligna*, *L. sativa*, *L. serriola* and *L. virosa* accessions, from illumina reads.

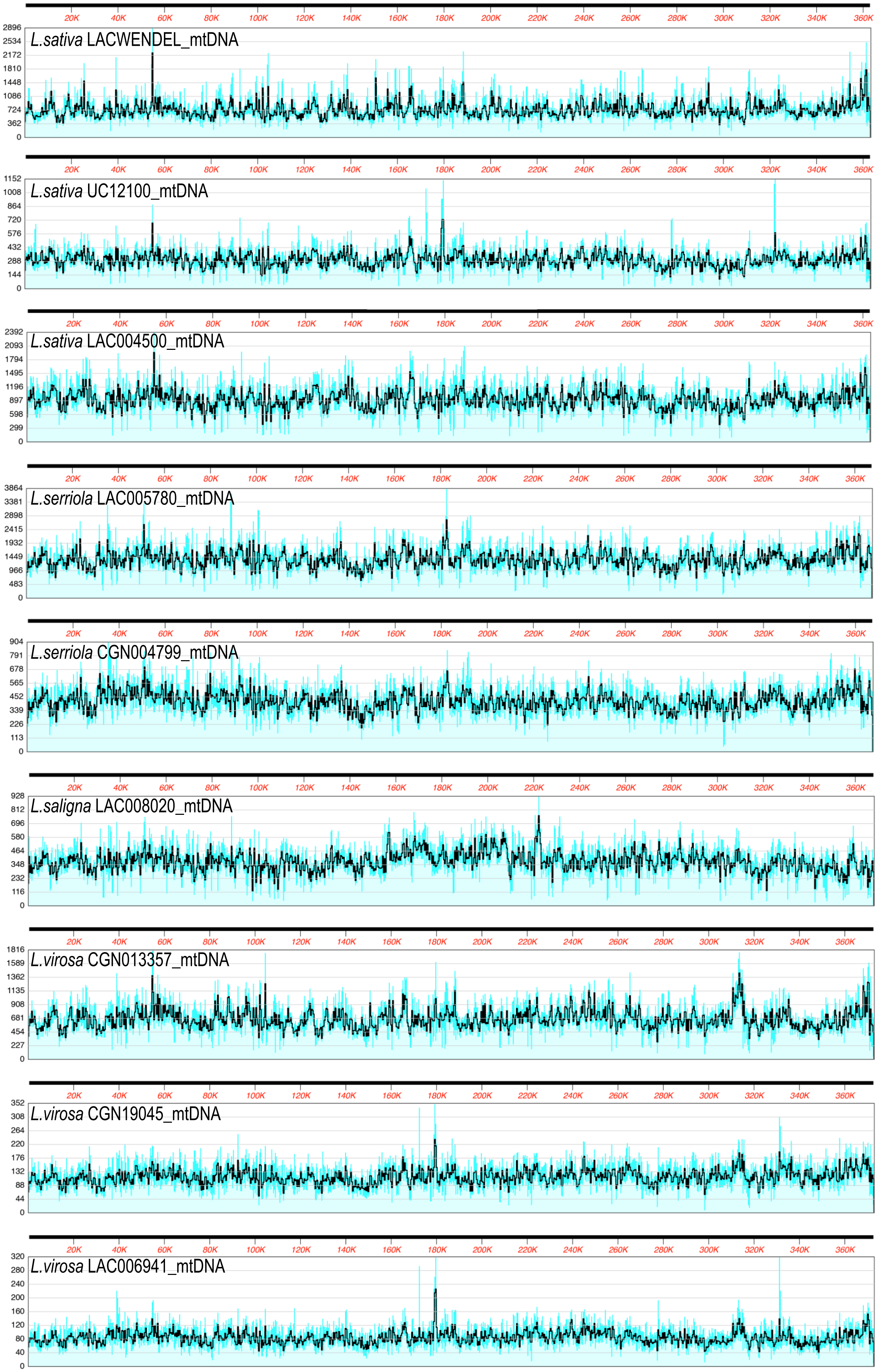

Supplemental Figure S2. Coverage of reads mapped back on the assembled mtDNA sequences
