# Supplementary material for: Sequence of the Mitochondrial Genome of Lactuca virosa Suggests an Unexpected Role in Lactuca sativa’s Evolution
Source: Front Plant Sci. 2021 Jul 26;12:697136. doi: 10.3389/fpls.2021.697136 (PMC8350775; doi:10.3389/fpls.2021.697136)
Supplement: Supplementary file 3 [file Data_Sheet_3.docx]

**ClustalW multiple sequence alignment of cpDNA sequences**

6 Sequences Aligned Processing time: 66 mins 3971 secs

Gaps Inserted = 17 Conserved Identities = 126686

Score = 175488372

Pairwise Alignment Mode: Fast

Pairwise Alignment Parameters:

ktup = 2 Gap Penalty = 5 Top Diagonals = 4 Window Size = 4

Multiple Alignment Parameters:

Open Gap Penalty = 15.0 Extend Gap Penalty = 6.7

Delay Divergent = 30% Transitions: Weighted

Lsat_Salinas 1 TAAATTCAATTTGGGCGAACGACGGGAATTGAACCCGCGCATGGTGGATT 50

Lsat_WENDEL 1 TAAATTCAATTTGGGCGAACGACGGGAATTGAACCCGCGCATGGTGGATT 50

Lser_US96UC23 1 TAAATTCAATTTGGGCGAACGACGGGAATTGAACCCGCGCATGGTGGATT 50

Lser_LAC005780 1 TAAATTCAATTTGGGCGAACGACGGGGATTGAACCCGCGCGTGGTGGATT 50

Lvir_CGN013357 1 TAAATTAAATTTGGGCGAACGACGGGAATTGAACCCGCGCGTGGTGGATT 50

Lsal_LAC008020 1 TAAATTAAATTTGGGCGAACGACGGGGATTGAACCCGCGCGTGGTGGATT 50

Lsat_Salinas 51 CACAATCCACTGCCTTGATCCACTTGGCTACATCCGCCCCTCTACTATTA 100

Lsat_WENDEL 51 CACAATCCACTGCCTTGATCCACTTGGCTACATCCGCCCCTCTACTATTA 100

Lser_US96UC23 51 CACAATCCACTGCCTTGATCCACTTGGCTACATCCGCCCCTCTACTATTA 100

Lser_LAC005780 51 CACAATCCACTGCCTTGATCCACTTGGCTACATCCGCCCCTCTACTATTA 100

Lvir_CGN013357 51 CACAATCCACTGCCTTGATCCACTTGGCTACATCCGCCCCTCTACTATTA 100

Lsal_LAC008020 51 CACAATCCACTGCCTTGATCCACTTGGCTACATCCGCCCCTCTACTATTA 100

Lsat_Salinas 101 TCTAGTATTTCTAGATTTTTCCATTAACAGAAAAAAAAAGAGCATATTAT 150

Lsat_WENDEL 101 TCTAGTATTTCTAGATTTTTCCATTAACAGAAAAAAAAAGAGCATATTAT 150

Lser_US96UC23 101 TCTAGTATTTCTAGATTTTTCCATTAACAGAAAAAAAAAGAGCATATTAT 150

Lser_LAC005780 101 TCTAGTATTTCTAGATTTTTCCATTAACAGAAAAAAAAAGAGCATATTAT 150

Lvir_CGN013357 101 TCTAGTATTTCTAGATTTTTCCATTAACAGAAAAAAAAAGAGCATATTAT 150

Lsal_LAC008020 101 TCTAGGATTTCTAGATTTTTCCATTAACAGAAAAAAAAAGAGCATATTAT 150

Lsat_Salinas 151 TTCTTTCTTATTTTCTTTCTTATTTCTGAAATCAAAGAAATAAATAATAA 200

Lsat_WENDEL 151 TTCTTTCTTATTTTCTTTCTTATTTCTGAAATCAAAGAAATAAATAATAA 200

Lser_US96UC23 151 TTCTTTCTTATTTTCTTTCTTATTTCTGAAATCAAAGAAATAAATAATAA 200

Lser_LAC005780 151 TTCTTTCTTATTTTCTTTCTTATTTCTGAAATCAAAGAAATAAATAATAA 200

Lvir_CGN013357 151 TTCTTTCTTATTTTCTTTCTTATTTCTGAAATCAAAGAAATAAATAATAA 200

Lsal_LAC008020 151 TTCTTTCTTATTTTCTTTCTTATTTCTGAAATCAAAGAAATAAATAATAA 200

Lsat_Salinas 201 AAATTTTCATTTTTATCTATTTTAGATTGAAATTCAATTGTAAATCAAAC 250

Lsat_WENDEL 201 AAATTTTCATTTTTATCTATTTTAGATTGAAATTCAATTGTAAATCAAAC 250

Lser_US96UC23 201 AAATTTTCATTTTTATCTATTTTAGATTGAAATTCAATTGTAAATCAAAC 250

Lser_LAC005780 201 AAATTTTCATTTTTATCTATTTTAGATTGAAATTCAATTGTAAATCAAAC 250

Lvir_CGN013357 201 AAATTTTCATTTTTATCTATTTTAGATTGAAATTCAATTGTAAATCAAAC 250

Lsal_LAC008020 201 AAATTTTCATTTTTATCTATTTTAGATTGAAATTCAATTGTAAATCAAAC 250

Lsat_Salinas 251 TTCATAAAACATTGGGAAAAGAATATATAAACCTCTAAATTAATACAAAG 300

Lsat_WENDEL 251 TTCATAAAACATTGGGAAAAGAATATATAAACCTCTAAATTAATACAAAG 300

Lser_US96UC23 251 TTCATAAAACATTGGGAAAAGAATATATAAACCTCTAAATTAATACAAAG 300

Lser_LAC005780 251 TTCATAAAACATTGGGAAAAGAATATATAAACCTCTAAATTAATACAAAG 300

Lvir_CGN013357 251 TTCATAAAACATTGGGAAAAGAATATATAAACCTCTAAATTAATACAAAG 300

Lsal_LAC008020 251 TTCATAAAACATTGGGAAAAGAATATATAAACCTCTAAATTAATACAAAG 300

Lsat_Salinas 301 AGAAAACATGCTAATCGAACCAAACAAAAGACCTTGTTATTTCTAAAGAA 350

Lsat_WENDEL 301 AGAAAACATGCTAATCGAACCAAACAAAAGACCTTGTTATTTCTAAAGAA 350

Lser_US96UC23 301 AGAAAACATGCTAATCGAACCAAACAAAAGACCTTGTTATTTCTAAAGAA 350

Lser_LAC005780 301 AGAAAACATGCTAATCGAACCAAACAAAAGACCTTGTTATTTCTAAAGAA 350

Lvir_CGN013357 301 AGAAAACATGCTAATCGAACCAAACAAAAGACCTTGTTATTTCTAAAGAA 350

Lsal_LAC008020 301 AGAAAACATGCTAATCGAACCAAACAAAAGACCTTGTTATTTCTAAAGAA 350

Lsat_Salinas 351 ACTATGTAAGGCAAATACTACTAAATAAAAAAAAAGGAGCAATAGCTTCC 400

Lsat_WENDEL 351 ACTATGTAAGGCAAATACTACTAAATAAAAAAAAAGGAGCAATAGCTTCC 400

Lser_US96UC23 351 ACTATGTAAGGCAAATACTACTAAATAAAAAAAAAGGAGCAATAGCTTCC 400

Lser_LAC005780 351 ACTATGTAAGGCAAATACTACTAAATAAAAAAAAAGGAGCAATAGCTTCC 400

Lvir_CGN013357 351 ACTATGTAAGGCAAATACTACTAAATAAAAAAAAAGGAGCAATAGCTTCC 400

Lsal_LAC008020 351 ACTATGTAAGGCAAATACTACTAAATAAAAAAAAAGGAGCAATAGCTTCC 400

Lsat_Salinas 401 CTCTTGTTTTATCAAGAGGGCGTTATTGCTCCTTTTTTAGTTCAAAAACT 450

Lsat_WENDEL 401 CTCTTGTTTTATCAAGAGGGCGTTATTGCTCCTTTTTTAGTTCAAAAACT 450

Lser_US96UC23 401 CTCTTGTTTTATCAAGAGGGCGTTATTGCTCCTTTTTTAGTTCAAAAACT 450

Lser_LAC005780 401 CTCTTGTTTTATCAAGAGGGCGTTATTGCTCCTTTTTTAGTTCAAAAACT 450

Lvir_CGN013357 401 CTCTTGTTTTATCAAGAGGGCGTTATTGCTCCTTTTTTAGTTCAAAAACT 450

Lsal_LAC008020 401 CTCTTGTTTTATCAAGAGGGCGTTATTGCTCCTTTTTTAGTTCAAAAACT 450

Lsat_Salinas 451 CCTATACAATTACAATAAGACCAAAGTCTTATCCATTTGTAGATGGAGCT 500

Lsat_WENDEL 451 CCTATACAATTACAATAAGACCAAAGTCTTATCCATTTGTAGATGGAGCT 500

Lser_US96UC23 451 CCTATACAATTACAATAAGACCAAAGTCTTATCCATTTGTAGATGGAGCT 500

Lser_LAC005780 451 CCTATACAATTACAATAAGACCAAAGTCTTATCCATTTGTAGATGGAGCT 500

Lvir_CGN013357 451 CCTATACAATTACAATAAGACCAAAGTCTTATCCATTTGTAGATGGAGCT 500

Lsal_LAC008020 451 CCTATACAATTACAATAAGACCAAAGTCTTATCCATTTGTAGATGGAGCT 500

Lsat_Salinas 501 TCGATAGCAGCTAAGTCTAGAGGGAAATTATGAGCATTACGTTCATGCAT 550

Lsat_WENDEL 501 TCGATAGCAGCTAAGTCTAGAGGGAAATTATGAGCATTACGTTCATGCAT 550

Lser_US96UC23 501 TCGATAGCAGCTAAGTCTAGAGGGAAATTATGAGCATTACGTTCATGCAT 550

Lser_LAC005780 501 TCGATAGCAGCTAAGTCTAGAGGGAAATTATGAGCATTACGTTCATGCAT 550

Lvir_CGN013357 501 TCGATAGCAGCTAAGTCTAGAGGGAAATTATGAGCATTACGTTCATGCAT 550

Lsal_LAC008020 501 TCGATAGCAGCTAAGTCTAGAGGGAAATTATGAGCATTACGTTCATGCAT 550

Lsat_Salinas 551 AACTTCCATACCAAGGTTAGCACGGTTAATGATATCAGCCCAAGTATTAA 600

Lsat_WENDEL 551 AACTTCCATACCAAGGTTAGCACGGTTAATGATATCAGCCCAAGTATTAA 600

Lser_US96UC23 551 AACTTCCATACCAAGGTTAGCACGGTTAATGATATCAGCCCAAGTATTAA 600

Lser_LAC005780 551 AACTTCCATACCAAGGTTAGCACGGTTAATGATATCAGCCCAAGTATTAA 600

Lvir_CGN013357 551 AACTTCCATACCAAGGTTAGCACGGTTAATGATATCAGCCCAAGTATTAA 600

Lsal_LAC008020 551 AACTTCCATACCAAGGTTAGCACGGTTAATGATATCAGCCCAAGTATTAA 600

Lsat_Salinas 601 TTACACGGCCTTGACTATCAACTACCGATTGGTTGAAATTGAAACCATTT 650

Lsat_WENDEL 601 TTACACGGCCTTGACTATCAACTACCGATTGGTTGAAATTGAAACCATTT 650

Lser_US96UC23 601 TTACACGGCCTTGACTATCAACTACCGATTGGTTGAAATTGAAACCATTT 650

Lser_LAC005780 601 TTACACGGCCTTGACTATCAACTACCGATTGGTTGAAATTGAAACCATTT 650

Lvir_CGN013357 601 TTACACGGCCTTGACTATCAACTACCGATTGGTTGAAATTGAAACCATTT 650

Lsal_LAC008020 601 TTACACGGCCTTGACTATCAACTACCGATTGGTTGAAATTGAAACCATTT 650

Lsat_Salinas 651 AGGTTGAAAGCCATAGTGCTGATACCTAAAGCAGTGAACCAGATACCTAC 700

Lsat_WENDEL 651 AGGTTGAAAGCCATAGTGCTGATACCTAAAGCAGTGAACCAGATACCTAC 700

Lser_US96UC23 651 AGGTTGAAAGCCATAGTGCTGATACCTAAAGCAGTGAACCAGATACCTAC 700

Lser_LAC005780 651 AGGTTGAAAGCCATAGTGCTGATACCTAAAGCAGTGAACCAGATACCTAC 700

Lvir_CGN013357 651 AGGTTGAAAGCCATAGTGCTGATACCTAAAGCAGTGAACCAGATACCTAC 700

Lsal_LAC008020 651 AGGTTGAAAGCCATAGTGCTGATACCTAAAGCAGTGAACCAGATACCTAC 700

Lsat_Salinas 701 TACAGGCCAAGCAGCTAGGAAGAAATGTAAAGAACGAGAGTTGTTGAAAC 750

Lsat_WENDEL 701 TACAGGCCAAGCAGCTAGGAAGAAATGTAAAGAACGAGAGTTGTTGAAAC 750

Lser_US96UC23 701 TACAGGCCAAGCAGCTAGGAAGAAATGTAAAGAACGAGAGTTGTTGAAAC 750

Lser_LAC005780 701 TACAGGCCAAGCAGCTAGGAAGAAATGTAAAGAACGAGAGTTGTTGAAAC 750

Lvir_CGN013357 701 TACAGGCCAAGCAGCTAGGAAGAAATGTAAAGAACGAGAGTTATTGAAAC 750

Lsal_LAC008020 701 TACAGGCCAAGCAGCTAGGAAGAAATGTAAAGAACGAGAGTTGTTGAAAC 750

Lsat_Salinas 751 TAGCATATTGGAAGATCAATCGGCCAAAATAACCATGAGCGGCTACGATA 800

Lsat_WENDEL 751 TAGCATATTGGAAGATCAATCGGCCAAAATAACCATGAGCGGCTACGATA 800

Lser_US96UC23 751 TAGCATATTGGAAGATCAATCGGCCAAAATAACCATGAGCGGCTACGATA 800

Lser_LAC005780 751 TAGCATATTGGAAGATCAATCGGCCAAAATAACCATGAGCGGCTACGATA 800

Lvir_CGN013357 751 TAGCATATTGGAAGATCAATCGGCCAAAATAACCATGAGCGGCTACGATA 800

Lsal_LAC008020 751 TAGCATATTGGAAGATCAATCGGCCAAAATAACCATGAGCGGCTACGATA 800

Lsat_Salinas 801 TTATAAGTTTCTTCTTCTTGACCGAATCTGTAACCTTCATTAGCAGATTC 850

Lsat_WENDEL 801 TTATAAGTTTCTTCTTCTTGACCGAATCTGTAACCTTCATTAGCAGATTC 850

Lser_US96UC23 801 TTATAAGTTTCTTCTTCTTGACCGAATCTGTAACCTTCATTAGCAGATTC 850

Lser_LAC005780 801 TTATAAGTTTCTTCTTCTTGACCGAATCTGTAACCTTCATTAGCAGATTC 850

Lvir_CGN013357 801 TTATAAGTTTCTTCTTCTTGACCGAATCTGTAACCTTCATTAGCAGATTC 850

Lsal_LAC008020 801 TTATAAGTTTCTTCTTCTTGACCGAATCTGTAACCTTCATTAGCAGATTC 850

Lsat_Salinas 851 ATTTTCTGTGGTTTCCCTGATCAAACTAGAGGTTACCAAAGAACCATGCA 900

Lsat_WENDEL 851 ATTTTCTGTGGTTTCCCTGATCAAACTAGAGGTTACCAAAGAACCATGCA 900

Lser_US96UC23 851 ATTTTCTGTGGTTTCCCTGATCAAACTAGAGGTTACCAAAGAACCATGCA 900

Lser_LAC005780 851 ATTTTCTGTGGTTTCCCTGATCAAACTAGAGGTTACCAAAGAACCATGCA 900

Lvir_CGN013357 851 ATTTTCTGTGGTTTCCCTGATCAAACTAGAGGTTACCAAAGAACCATGCA 900

Lsal_LAC008020 851 ATTTTCTGTGGTTTCCCTGATCAAACTAGAGGTTACCAAAGAACCATGCA 900

Lsat_Salinas 901 TAGCACTAAATAGGGAGCCGCCGAATACACCAGCTACGCCTAGCATGTGA 950

Lsat_WENDEL 901 TAGCACTAAATAGGGAGCCGCCGAATACACCAGCTACGCCTAGCATGTGA 950

Lser_US96UC23 901 TAGCACTAAATAGGGAGCCGCCGAATACACCAGCTACGCCTAGCATGTGA 950

Lser_LAC005780 901 TAGCACTAAATAGGGAGCCGCCGAATACACCAGCTACGCCTAGCATGTGA 950

Lvir_CGN013357 901 TAGCACTAAATAGGGAGCCGCCGAATACACCAGCTACGCCTAGCATGTGA 950

Lsal_LAC008020 901 TAGCACTAAATAGGGAGCCGCCGAATACACCAGCTACGCCTAGCATGTGA 950

Lsat_Salinas 951 AATGGGTGCATAAGGATGTTGTGCTCAGCCTGGAATACAATCATGAAGTT 1000

Lsat_WENDEL 951 AATGGGTGCATAAGGATGTTGTGCTCAGCCTGGAATACAATCATGAAGTT 1000

Lser_US96UC23 951 AATGGGTGCATAAGGATGTTGTGCTCAGCCTGGAATACAATCATGAAGTT 1000

Lser_LAC005780 951 AATGGGTGCATAAGGATGTTGTGCTCAGCCTGGAATACAATCATGAAGTT 1000

Lvir_CGN013357 951 AATGGGTGCATAAGGATGTTGTGCTCAGCCTGGAATACAATCATGAAGTT 1000

Lsal_LAC008020 951 AATGGGTGCATAAGGATGTTGTGCTCAGCCTGGAATACAATCATGAAGTT 1000

Lsat_Salinas 1001 GAAAGTACCAGAAATTCCTAGAGGCATACCATCAGAAAAGCTTCCTTGAC 1050

Lsat_WENDEL 1001 GAAAGTACCAGAAATTCCTAGAGGCATACCATCAGAAAAGCTTCCTTGAC 1050

Lser_US96UC23 1001 GAAAGTACCAGAAATTCCTAGAGGCATACCATCAGAAAAGCTTCCTTGAC 1050

Lser_LAC005780 1001 GAAAGTACCAGAAATTCCTAGAGGCATACCATCAGAAAAGCTTCCTTGAC 1050

Lvir_CGN013357 1001 GAAAGTACCAGAAATTCCTAGAGGCATACCATCAGAAAAGCTTCCTTGAC 1050

Lsal_LAC008020 1001 GAAAGTACCAGAAATTCCTAGAGGCATACCATCAGAAAAGCTTCCTTGAC 1050

Lsat_Salinas 1051 CAATTGGGTAGATCAAGAAAACAGCAGTCGCAGCTGCAACAGGAGCTGAA 1100

Lsat_WENDEL 1051 CAATTGGGTAGATCAAGAAAACAGCAGTCGCAGCTGCAACAGGAGCTGAA 1100

Lser_US96UC23 1051 CAATTGGGTAGATCAAGAAAACAGCAGTCGCAGCTGCAACAGGAGCTGAA 1100

Lser_LAC005780 1051 CAATTGGGTAGATCAAGAAAACAGCAGTCGCAGCTGCAACAGGAGCTGAA 1100

Lvir_CGN013357 1051 CAATTGGGTAGATCAAGAAAACAGCAGTCGCAGCTGCAACAGGAGCTGAA 1100

Lsal_LAC008020 1051 CAATTGGGTAGATCAAGAAAACAGCAGTCGCAGCTGCAACAGGAGCTGAA 1100

Lsat_Salinas 1101 TATGCAACAGCAATCCAAGGTCGCATACCCAGACGGAAACTAAGCTCCCA 1150

Lsat_WENDEL 1101 TATGCAACAGCAATCCAAGGTCGCATACCCAGACGGAAACTAAGCTCCCA 1150

Lser_US96UC23 1101 TATGCAACAGCAATCCAAGGTCGCATACCCAGACGGAAACTAAGCTCCCA 1150

Lser_LAC005780 1101 TATGCAACAGCAATCCAAGGTCGCATACCCAGACGGAAACTAAGCTCCCA 1150

Lvir_CGN013357 1101 TATGCAACAGCAATCCAAGGTCGCATACCCAGACGGAAACTAAGCTCCCA 1150

Lsal_LAC008020 1101 TATGCAACAGCAATCCAAGGTCGCATACCCAGACGGAAACTAAGCTCCCA 1150

Lsat_Salinas 1151 CTCACGACCCATGTAACAAGCTACACCAAGTAAGAAGTGTAGAACAATTA 1200

Lsat_WENDEL 1151 CTCACGACCCATGTAACAAGCTACACCAAGTAAGAAGTGTAGAACAATTA 1200

Lser_US96UC23 1151 CTCACGACCCATGTAACAAGCTACACCAAGTAAGAAGTGTAGAACAATTA 1200

Lser_LAC005780 1151 CTCACGACCCATGTAACAAGCTACACCAAGTAAGAAGTGTAGAACAATTA 1200

Lvir_CGN013357 1151 CTCACGACCCATGTAACAAGCTACACCAAGTAAGAAGTGTAGAACAATTA 1200

Lsal_LAC008020 1151 CTCACGACCCATGTAACAAGCTACACCAAGTAAGAAGTGTAGAACAATTA 1200

Lsat_Salinas 1201 GTTCATAAGGACCACCATTGTATAACCATTCATCAACGGATGCTGCTTCC 1250

Lsat_WENDEL 1201 GTTCATAAGGACCACCATTGTATAACCATTCATCAACGGATGCTGCTTCC 1250

Lser_US96UC23 1201 GTTCATAAGGACCACCATTGTATAACCATTCATCAACGGATGCTGCTTCC 1250

Lser_LAC005780 1201 GTTCATAAGGACCACCATTGTATAACCATTCATCAACGGATGCTGCTTCC 1250

Lvir_CGN013357 1201 GTTCATAAGGACCACCATTGTATAACCATTCATCAACGGATGCTGCTTCC 1250

Lsal_LAC008020 1201 GTTCATAAGGACCACCATTGTATAACCATTCATCAACAGATGCTGCTTCC 1250

Lsat_Salinas 1251 CATATTGGGTAAAAATGCAAACCTATAGCTGCAGAAGTAGGAATAATGGC 1300

Lsat_WENDEL 1251 CATATTGGGTAAAAATGCAAACCTATAGCTGCAGAAGTAGGAATAATGGC 1300

Lser_US96UC23 1251 CATATTGGGTAAAAATGCAAACCTATAGCTGCAGAAGTAGGAATAATGGC 1300

Lser_LAC005780 1251 CAGATTGGGTAAAAATGCAAACCTATAGCTGCAGAAGTAGGAATAATGGC 1300

Lvir_CGN013357 1251 CATATTGGGTAAAAATGCAAACCTATAGCTGCAGAAGTAGGAATAATGGC 1300

Lsal_LAC008020 1251 CATATTGGGTAAAAATGCAAACCTATAGCTGCAGAAGTAGGAATAATGGC 1300

Lsat_Salinas 1301 ACCTGAAATAATATTGTTTCCATAAAGTAGAGATCCAGAAACAGGTTCAC 1350

Lsat_WENDEL 1301 ACCTGAAATAATATTGTTTCCATAAAGTAGAGATCCAGAAACAGGTTCAC 1350

Lser_US96UC23 1301 ACCTGAAATAATATTGTTTCCATAAAGTAGAGATCCAGAAACAGGTTCAC 1350

Lser_LAC005780 1301 ACCTGAAATAATATTGTTTCCATAAAGTAGAGATCCAGAAACAGGTTCAC 1350

Lvir_CGN013357 1301 ACCTGAAATAATATTGTTTCCATAAAGTAGAGATCCAGAAACAGGTTCAC 1350

Lsal_LAC008020 1301 ACCTGAAATAATATTGTTTCCATAAAGTAGAGATCCAGAAACAGGTTCAC 1350

Lsat_Salinas 1351 GAATACCATCAATATCCACTGGAGGAGCAGCAATGAAGGCGATAATAAAT 1400

Lsat_WENDEL 1351 GAATACCATCAATATCCACTGGAGGAGCAGCAATGAAGGCGATAATAAAT 1400

Lser_US96UC23 1351 GAATACCATCAATATCCACTGGAGGAGCAGCAATGAAGGCGATAATAAAT 1400

Lser_LAC005780 1351 GAATACCATCAATATCCACTGGAGGAGCAGCAATGAAGGCGATAATAAAT 1400

Lvir_CGN013357 1351 GAATACCATCAATATCCACTGGAGGAGCAGCAATGAAGGCGATAATAAAT 1400

Lsal_LAC008020 1351 GAATACCATCAATATCCACTGGAGGAGCAGCAATGAAGGCGATAATAAAT 1400

Lsat_Salinas 1401 ACAGAAGTTGCGGTCAATAAGGTAGGGATCATCAAAACACCAAACCATCC 1450

Lsat_WENDEL 1401 ACAGAAGTTGCGGTCAATAAGGTAGGGATCATCAAAACACCAAACCATCC 1450

Lser_US96UC23 1401 ACAGAAGTTGCGGTCAATAAGGTAGGGATCATCAAAACACCAAACCATCC 1450

Lser_LAC005780 1401 ACAGAAGTTGCGGTCAATAAGGTAGGGATCATCAAAACACCAAACCATCC 1450

Lvir_CGN013357 1401 ACAGAAGTTGCGGTCAATAAGGTAGGGATCATCAAAACACCAAACCATCC 1450

Lsal_LAC008020 1401 ACAGAAGTTGCGGTCAATAAGGTAGGGATCATCAAAACACCAAACCATCC 1450

Lsat_Salinas 1451 AATGTAAAGACGGTTTTCGGTGCTGGTTATCCAGTTACAGAAGCGACCCC 1500

Lsat_WENDEL 1451 AATGTAAAGACGGTTTTCGGTGCTGGTTATCCAGTTACAGAAGCGACCCC 1500

Lser_US96UC23 1451 AATGTAAAGACGGTTTTCGGTGCTGGTTATCCAGTTACAGAAGCGACCCC 1500

Lser_LAC005780 1451 AATGTAAAGACGGTTTTCGGTGCTGGTTATCCAGTTACAGAAGCGACCCC 1500

Lvir_CGN013357 1451 AATGTAAAGACGGTTTTCGGTGCTGGTTATCCAGTTACAGAAGCGACCCC 1500

Lsal_LAC008020 1451 AATGTAAAGACGGTTTTCGGTGCTGGTTATCCAGTTACAGAAGCGACCCC 1500

Lsat_Salinas 1501 ATAGGCTTTCGCTTTCGCGTCTCTCTAAAATTGCAGTCATGGTAAAATCT 1550

Lsat_WENDEL 1501 ATAGGCTTTCGCTTTCGCGTCTCTCTAAAATTGCAGTCATGGTAAAATCT 1550

Lser_US96UC23 1501 ATAGGCTTTCGCTTTCGCGTCTCTCTAAAATTGCAGTCATGGTAAAATCT 1550

Lser_LAC005780 1501 ATAGGCTTTCGCTTTCGCGTCTCTCTAAAATTGCAGTCATGGTAAAATCT 1550

Lvir_CGN013357 1501 ATAGGCTTTCGCTTTCGCGTCTCTCTAAAATTGCAGTCATGGTAAAATCT 1550

Lsal_LAC008020 1501 ATAGGCTTTCGCTTTCGCGTCTCTCTAAAATTGCAGTCATGGTAAAATCT 1550

Lsat_Salinas 1551 TGGTTTATTTAATTATCAGGGACTCCCAAGCGCACGAATTTTCAAATGGA 1600

Lsat_WENDEL 1551 TGGTTTATTTAATTATCAGGGACTCCCAAGCGCACGAATTTTCAAATGGA 1600

Lser_US96UC23 1551 TGGTTTATTTAATTATCAGGGACTCCCAAGCGCACGAATTTTCAAATGGA 1600

Lser_LAC005780 1551 TGGTTTATTTAATTATCAGGGACTCCCAAGCGCACGAATTTTCAAATGGA 1600

Lvir_CGN013357 1551 TGGTTTATTTAATTATCAGGGACTCCCAAGCGCACGAATTTTCAAATGGA 1600

Lsal_LAC008020 1551 TGGTTTATTTAATTATCAGGGACTCCCAAGCGCACGAATTTTCAAATGGA 1600

Lsat_Salinas 1601 AAACTAAAGGCTTGTTATTTAACAGTATAACATGACTTATATGCCCGTGT 1650

Lsat_WENDEL 1601 AAACTAAAGGCTTGTTATTTAACAGTATAACATGACTTATATGCCCGTGT 1650

Lser_US96UC23 1601 AAACTAAAGGCTTGTTATTTAACAGTATAACATGACTTATATGCCCGTGT 1650

Lser_LAC005780 1601 AAACTAAAGGCTTGTTATTTAACAGTATAACATGACTTATATGCCCGTGT 1650

Lvir_CGN013357 1601 AAACTAAAGGCTTGTTATTTAACAGTATAACATGACTTATATGCCCGTGT 1650

Lsal_LAC008020 1601 AAACTAAAGGCTTGTTATTTAACAGTATAACATGACTTATATGCCCGTGT 1650

Lsat_Salinas 1651 CAACCAATATCTATCTGGATCTAGTTCAATTTTTTGTAAATCAAAAGCGG 1700

Lsat_WENDEL 1651 CAACCAATATCTATCTGGATCTAGTTCAATTTTTTGTAAATCAAAAGCGG 1700

Lser_US96UC23 1651 CAACCAATATCTATCTGGATCTAGTTCAATTTTTTGTAAATCAAAAGCGG 1700

Lser_LAC005780 1651 CAACCAATATCTATCTGGATCTAGTTCAATTTTTTGTAAATCAAAAGCGG 1700

Lvir_CGN013357 1651 CAACCAATATCTATCTGGATCTAGTTCAATTTTTTGTAAATCAAAAGCGG 1700

Lsal_LAC008020 1651 CAACCAATATCTATCTGGATCTAGTTCAATTTTTTGTAAATCAAAAGCGG 1700

Lsat_Salinas 1701 TTTGCAAAAATAAAAAAAAAAGGATTTCTATACACTATGCATATAATTTA 1750

Lsat_WENDEL 1701 TTTGCAAAAATAAAAAAAAAAGGATTTCTATACACTATGCATATAATTTA 1750

Lser_US96UC23 1701 TTTGCAAAAGTAAAAAAAAAAGGATTTCTATACACTATGCATATAATTTA 1750

Lser_LAC005780 1701 TTTGCAAAAATAAAAAAAAAAGGATTTCTATACACTATGCATATAATTTA 1750

Lvir_CGN013357 1701 TTTGCAAAAATAAAAAAAAAAGGATTTCTATACACTATGCATATAATATA 1750

Lsal_LAC008020 1701 TTTGCAAAAATAAAAAAAAAAGGATTTCTATACACTATGCATATAATATA 1750

Lsat_Salinas 1751 ATTTCACTATGACAATGGGTTGCCCGGGATTCGAACCCGGAACTAGTCGG 1800

Lsat_WENDEL 1751 ATTTCACTATGACAATGGGTTGCCCGGGATTCGAACCCGGAACTAGTCGG 1800

Lser_US96UC23 1751 ATTTCACTATGACAATGGGTTGCCCGGGATTCGAACCCGGAACTAGTCGG 1800

Lser_LAC005780 1751 ATTTCACTATGACAATGGGTTGCCCGGGATTCGAACCCGGAACTAGTCGG 1800

Lvir_CGN013357 1751 ATTTCACTATGACAATGGGTTGCCCGGGATTCGAACCCGGAACTAGTCGG 1800

Lsal_LAC008020 1751 ATTTCACTATGACAATGGGTTGCCCGGGATTCGAACCCGGAACTAGTCGG 1800

Lsat_Salinas 1801 ATGGAGTAGATAATTTCCTTGTTAAAGTTAAATAAGTAAAAATCCCTCCC 1850

Lsat_WENDEL 1801 ATGGAGTAGATAATTTCCTTGTTAAAGTTAAATAAGTAAAAATCCCTCCC 1850

Lser_US96UC23 1801 ATGGAGTAGATAATTTCCTTGTTAAAGTTAAATAAGTAAAAATCCCTCCC 1850

Lser_LAC005780 1801 ATGGAGTAGATAATTTCCTTGTTAAAGTTAAATAAGTAAAAATCCCTCCC 1850

Lvir_CGN013357 1801 ATGGAGTAGATAATTTCCTTGTTAAAGTTAAATAAGTAAAAATCCCTCCC 1850

Lsal_LAC008020 1801 ATGGAGTAGATAATTTCCTTGTTAAAGTTAAATAAGTAAAAATCCCTCCC 1850

Lsat_Salinas 1851 CAAGCCGTGCTTGCATTTTTCATTGCACACGGCTTTCCCTCTGTATACAT 1900

Lsat_WENDEL 1851 CAAGCCGTGCTTGCATTTTTCATTGCACACGGCTTTCCCTCTGTATACAT 1900

Lser_US96UC23 1851 CAAGCCGTGCTTGCATTTTTCATTGCACACGGCTTTCCCTCTGTATACAT 1900

Lser_LAC005780 1851 CAAGCCGTGCTTGCATTTTTCATTGCACACGGCTTTCCCTCTGTATACAT 1900

Lvir_CGN013357 1851 CAAGCCGTGCTTGCATTTTTCATTGCACACGGCTTTCCCTCTGTATACAT 1900

Lsal_LAC008020 1851 CAAGCCGTGCTTGCATTTTTCATTGCACACGGCTTTCCCTCTGTATACAT 1900

Lsat_Salinas 1901 CTAAAACTCAGTTCCTTCATTAGACAAGAAAAGATTGAATACTCAGTTGA 1950

Lsat_WENDEL 1901 CTAAAACTCAGTTCCTTCATTAGACAAGAAAAGATTGAATACTCAGTTGA 1950

Lser_US96UC23 1901 CTAAAACTCAGTTCCTTCATTAGACAAGAAAAGATTGAATACTCAGTTGA 1950

Lser_LAC005780 1901 CTAAAACTCAGTTCCTTCATTAGACAAGAAAAGATTGAATACTCAGTTGA 1950

Lvir_CGN013357 1901 CTAAAACTCAGTTCCTTCATTAGACAAGAAAAGATTGAATACTCAGTTGA 1950

Lsal_LAC008020 1901 CTAAAACTCAGTTCCTTCATTAGACAAGAAAAGATTGAATACTCAGTTGA 1950

Lsat_Salinas 1951 TTTCATCCTTACTACATACTACATCAACATTTCAGAATAGAAAGAAATAA 2000

Lsat_WENDEL 1951 TTTCATCCTTACTACATACTACATCAACATTTCAGAATAGAAAGAAATAA 2000

Lser_US96UC23 1951 TTTCATCCTTACTACATACTACATCAACATTTCAGAATAGAAAGAAATAA 2000

Lser_LAC005780 1951 TTTCATCCTTACTACATACTACATCAACATTTCAGAATAGAAAGAAATAA 2000

Lvir_CGN013357 1951 TTTCATCCTTACTACATACTACATCAACATTTCAGAATAGAAAGAAATAA 2000

Lsal_LAC008020 1951 TTTCATCCTTACTACATACTACATCAACATTTCAGAATAGAAAGAAATAA 2000

Lsat_Salinas 2001 TTTTTTGTTAGCTCTTCATTATTTACATTATTTATTAGATTATTTAGTGA 2050

Lsat_WENDEL 2001 TTTTTTGTTAGCTCTTCATTATTTACATTATTTATTAGATTATTTAGTGA 2050

Lser_US96UC23 2001 TTTTTTGTTAGCTCTTCATTATTTACATTATTTATTAGATTATTTAGTGA 2050

Lser_LAC005780 2001 TTTTTTGTTAGCTCTTCATTATTTACATTATTTATTAGATTATTTAGTGA 2050

Lvir_CGN013357 2001 TTTTTTGTTAGCTCTTCATTATTTACATTATTTATTAGATTATTTAGTGA 2050

Lsal_LAC008020 2001 GTTTTTGTTAGCTCTTCATTATTTACATTATTTATTAGATTATTTAGTGA 2050

Lsat_Salinas 2051 TGAAATTTATATTTACAAGGTTTCATAACGAATCATTCATGATTGGCCAA 2100

Lsat_WENDEL 2051 TGAAATTTATATTTACAAGGTTTCATAACGAATCATTCATGATTGGCCAA 2100

Lser_US96UC23 2051 TGAAATTTATATTTACAAGGTTTCATAACGAATCATTCATGATTGGCCAA 2100

Lser_LAC005780 2051 TGAAATTTATATTTACAAGGTTTCATAACGAATCATTCATGATTGGCCAA 2100

Lvir_CGN013357 2051 TGCAATTTATATTTACAAGGTTTCATAACGAATCATTCATGATTGGCCAA 2100

Lsal_LAC008020 2051 TGAAATTTATATTTACAAGGTTTCATAACGAATCATTCATGATTGGCCAA 2100

Lsat_Salinas 2101 ATCATTGATACAAATAATATCCAAATACCAAATCCGCCTTCTAGATAACC 2150

Lsat_WENDEL 2101 ATCATTGATACAAATAATATCCAAATACCAAATCCGCCTTCTAGATAACC 2150

Lser_US96UC23 2101 ATCATTGATACAAATAATATCCAAATACCAAATCCGCCTTCTAGATAACC 2150

Lser_LAC005780 2101 ATCATTGATACAAATAATATCCAAATACCAAATCCGCCTTCTAGATAACC 2150

Lvir_CGN013357 2101 ATCATTGATACAAATAATATCCAAATACCAAATCCGCCTTCTAGATAACC 2150

Lsal_LAC008020 2101 ATCATTGATACAAATAATATCCAAATACCAAATCCGCCTTCTAGATAACC 2150

Lsat_Salinas 2151 TTCGCGAAATGGAAGAAACCCTTGGAAAGGTCAGGGAAAAAACTTGTTCT 2200

Lsat_WENDEL 2151 TTCGCGAAATGGAAGAAACCCTTGGAAAGGTCAGGGAAAAAACTTGTTCT 2200

Lser_US96UC23 2151 TTCGCGAAATGGAAGAAACCCTTGGAAAGGTCAGGGAAAAAACTTGTTCT 2200

Lser_LAC005780 2151 TTCGCGAAATGGAAGAAACCCTTGGAAAGGTCAGGGAAAAAACTTGTTCT 2200

Lvir_CGN013357 2151 TTCGCGAAATGGAAGAAACCCTTGGAAAGGTCAGGGAAAAAACTTGTTCT 2200

Lsal_LAC008020 2151 TTCGCGAAATGGAAGAAACCCTTGGAAAGGTCAGGGAAAAAACTTGTTCT 2200

Lsat_Salinas 2201 TCTTCCGTAAAGAATTCTTCTAATAATTCCGAGCCGAATCTTTTCAAAAA 2250

Lsat_WENDEL 2201 TCTTCCGTAAAGAATTCTTCTAATAATTCCGAGCCGAATCTTTTCAAAAA 2250

Lser_US96UC23 2201 TCTTCCGTAAAGAATTCTTCTAATAATTCCGAGCCGAATCTTTTCAAAAA 2250

Lser_LAC005780 2201 TCTTCCGTAAAGAATTCTTCTAATAATTCCGAGCCGAATCTTTTCAAAAA 2250

Lvir_CGN013357 2201 TCTTCCGTAAAGAATTCTTCTAATAATTCCGAGCCGAATCTTTTCAAAAA 2250

Lsal_LAC008020 2201 TCTTCCGTAAAGAATTCTTCTAATAATTCCGAGCCGAATCTTTTCAAAAA 2250

Lsat_Salinas 2251 AGCACGTACAGTGCTTTTATGCTTACGAGCTAAAGTTCTAGCACAAGAAA 2300

Lsat_WENDEL 2251 AGCACGTACAGTGCTTTTATGCTTACGAGCTAAAGTTCTAGCACAAGAAA 2300

Lser_US96UC23 2251 AGCACGTACAGTGCTTTTATGCTTACGAGCTAAAGTTCTAGCACAAGAAA 2300

Lser_LAC005780 2251 AGCACGTACAGTGCTTTTATGCTTACGAGCTAAAGTTCTAGCACAAGAAA 2300

Lvir_CGN013357 2251 AGCACGTACAGTGCTTTTATGCTTACGAGCTAAAGTTCTAGCACAAGAAA 2300

Lsal_LAC008020 2251 AGCACGTACAGTGCTTTTATGCTTACGAGCTAAAGTTCTAGCACAAGAAA 2300

Lsat_Salinas 2301 GTCGAAGTATATACTTTACTCGATACAAACTCTTTTTTTTTGAAGATCCA 2350

Lsat_WENDEL 2301 GTCGAAGTATATACTTTACTCGATACAAACTCTTTTTTTTTGAAGATCCA 2350

Lser_US96UC23 2301 GTCGAAGTATATACTTTACTCGATACAAACTCTTTTTTTTTGAAGATCCA 2350

Lser_LAC005780 2301 GTCGAAGTATATACTTTACTCGATACAAACTCTTTTTTTTTGAAGATCCA 2350

Lvir_CGN013357 2301 GTCGAAGTATATACTTTACTCGATACAAACTCTTTTTTTTTGAAGATCCA 2350

Lsal_LAC008020 2301 GTCGAAGTATATACTTTACTCGATACAAACTCTTTTTTTTTGAAGATCCA 2350

Lsat_Salinas 2351 CTATGATAATGAGAAAGATTTCTGTATATACGCCCAAAGCGGTCAATAAT 2400

Lsat_WENDEL 2351 CTATGATAATGAGAAAGATTTCTGTATATACGCCCAAAGCGGTCAATAAT 2400

Lser_US96UC23 2351 CTATGATAATGAGAAAGATTTCTGTATATACGCCCAAAGCGGTCAATAAT 2400

Lser_LAC005780 2351 CTATGATAATGAGAAAGATTTCTGTATATACGCCCAAAGCGGTCAATAAT 2400

Lvir_CGN013357 2351 CTATGATAATGAGAAAGATTTCTGTATATACGCCCAAAGCGGTCAATAAT 2400

Lsal_LAC008020 2351 CTATGATAATGAGAAAGATTTCTGTATATACGCCCAAAGCGGTCAATAAT 2400

Lsat_Salinas 2401 ATCAGAATCTGAGAAATCGGCCCAAATCGCCTTACCAATAGGATGCCCCA 2450

Lsat_WENDEL 2401 ATCAGAATCTGAGAAATCGGCCCAAATCGCCTTACCAATAGGATGCCCCA 2450

Lser_US96UC23 2401 ATCAGAATCTGAGAAATCGGCCCAAATCGCCTTACCAATAGGATGCCCCA 2450

Lser_LAC005780 2401 ATCAGAATCTGAGAAATCGGCCCAAATCGCCTTACCAATAGGATGCCCCA 2450

Lvir_CGN013357 2401 ATCAGAATCTGAGAAATCGGCCCAAATCGCCTTACCAATAGGATGCCCCA 2450

Lsal_LAC008020 2401 ATCAGAATCTGAGAAATCGGCCCAAATCGCCTTACCAATAGGATGCCCCA 2450

Lsat_Salinas 2451 ATGCATTACAAAATTTAGATTTAGCCAATGATCCAATCAAAGGCATAATT 2500

Lsat_WENDEL 2451 ATGCATTACAAAATTTAGATTTAGCCAATGATCCAATCAAAGGCATAATT 2500

Lser_US96UC23 2451 ATGCATTACAAAATTTAGATTTAGCCAATGATCCAATCAAAGGCATAATT 2500

Lser_LAC005780 2451 ATGCATTACAAAATTTAGATTTAGCCAATGATCCAATCAAAGGCATAATT 2500

Lvir_CGN013357 2451 ATGCATTACAAAATTTAGATTTAGCCAATGATCCAATCAAAGGCATAATT 2500

Lsal_LAC008020 2451 ATGCATTACAAAATTTAGATTTAGCCAATGATCCAATCAAAGGCATAATT 2500

Lsat_Salinas 2501 GGAACAATAGTCTCAAACTTCTTAATAGCATTTTCAATTATAAATGCATT 2550

Lsat_WENDEL 2501 GGAACAATAGTCTCAAACTTCTTAATAGCATTTTCAATTATAAATGCATT 2550

Lser_US96UC23 2501 GGAACAATAGTCTCAAACTTCTTAATAGCATTTTCAATTATAAATGCATT 2550

Lser_LAC005780 2501 GGAACAATAGTCTCAAACTTCTTAATAGCATTTTCAATTATAAATGCATT 2550

Lvir_CGN013357 2501 GGAACAATAGTCTCAAACTTCTTAATAGCATTTTCAATTATAAATGCATT 2550

Lsal_LAC008020 2501 GGAACAATAGTCTCAAACTTCTTAATAGCATTTTCAATTATAAATGAATT 2550

Lsat_Salinas 2551 TTCTAGCATTTGACTGCGTACCATTGAAGGCTTTAGCCGCACACTTGAAC 2600

Lsat_WENDEL 2551 TTCTAGCATTTGACTGCGTACCATTGAAGGCTTTAGCCGCACACTTGAAC 2600

Lser_US96UC23 2551 TTCTAGCATTTGACTGCGTACCATTGAAGGCTTTAGCCGCACACTTGAAC 2600

Lser_LAC005780 2551 TTCTAGCATTTGACTGCGTACCATTGAAGGCTTTAGCCGCACACTTGAAC 2600

Lvir_CGN013357 2551 TTCTAGCATTTGACTGCGTACCATTGAAGGCTTTAGCCGCACACTTGAAC 2600

Lsal_LAC008020 2551 TTCTAGCATTTGACTGCGTACCATTGAAGGCTTTAGCCGCACACTTGAAC 2600

Lsat_Salinas 2601 GATAACCCAGAAAGTCAAGGGAATGATTGGATAATTGGTTTATATAAATC 2650

Lsat_WENDEL 2601 GATAACCCAGAAAGTCAAGGGAATGATTGGATAATTGGTTTATATAAATC 2650

Lser_US96UC23 2601 GATAACCCAGAAAGTCAAGGGAATGATTGGATAATTGGTTTATATAAATC 2650

Lser_LAC005780 2601 GATAACCCAGAAAGTCAAGGGAATGATTGGATAATTGGTTTATATAAATC 2650

Lvir_CGN013357 2601 GATAACCCAGAAAGTCAAGGGAATGATTGGATAATTGGTTTATATAAATC 2650

Lsal_LAC008020 2601 GATAACCCAGAAAGTCAAGGGAATGATTGGATAATTGGTTTATATAAATC 2650

Lsat_Salinas 2651 CTTCCTGGTTGAGACCACAGGTAAAAATAAGATTTCCAGAAATTGACAAA 2700

Lsat_WENDEL 2651 CTTCCTGGTTGAGACCACAGGTAAAAATAAGATTTCCAGAAATTGACAAA 2700

Lser_US96UC23 2651 CTTCCTGGTTGAGACCACAGGTAAAAATAAGATTTCCAGAAATTGACAAA 2700

Lser_LAC005780 2651 CTTCCTGGTTGAGACCACAGGTAAAAATAAGATTTCCAGAAATTGACAAA 2700

Lvir_CGN013357 2651 CTTCCTGGTTGAGACCACAGGTAAAAATAAGATTTCCAGAAATTGACAAA 2700

Lsal_LAC008020 2651 CTTCCTGGTTGAGACCACAGGTAAAAATAAGATTTCCAGAAATTGATAAA 2700

Lsat_Salinas 2701 GTAATATTTCCATTTATTCATCAAAAGAAACGTCCCTTTTGAACCAAGAA 2750

Lsat_WENDEL 2701 GTAATATTTCCATTTATTCATCAAAAGAAACGTCCCTTTTGAACCAAGAA 2750

Lser_US96UC23 2701 GTAATATTTCCATTTATTCATCAAAAGAAACGTCCCTTTTGAACCAAGAA 2750

Lser_LAC005780 2701 GTAATATTTCCATTTATTCATCAAAAGAAACGTCCCTTTTGAACCAAGAA 2750

Lvir_CGN013357 2701 GTAATATTTCCATTTATTCATCAAAAGAAACGTCCCTTTTGAACCAAGAA 2750

Lsal_LAC008020 2701 GTAATATTTCCATTTATTCATCAAAAGAAACGTCCCTTTTGAACCAAGAA 2750

Lsat_Salinas 2751 TGGATTTTCCTTGATACCTAACATAATGCATGAAAGGATCTTTGAACAAC 2800

Lsat_WENDEL 2751 TGGATTTTCCTTGATACCTAACATAATGCATGAAAGGATCTTTGAACAAC 2800

Lser_US96UC23 2751 TGGATTTTCCTTGATACCTAACATAATGCATGAAAGGATCTTTGAACAAC 2800

Lser_LAC005780 2751 TGGATTTTCCTTGATACCTAACATAATGCATGAAAGGATCTTTGAACAAC 2800

Lvir_CGN013357 2751 TGGATTTTCCTTGATACCTAACATAATGCATGAAAGGATCTTTGAACAAC 2800

Lsal_LAC008020 2751 TGGATTTTCCTTGATACCTAACATAATGCATGAAAGGATCTTTGAACAAC 2800

Lsat_Salinas 2801 CATAAATTCGCTTGAAAAGCCCTGGCAAAGACTTCTGCAAGATGATCTAT 2850

Lsat_WENDEL 2801 CATAAATTCGCTTGAAAAGCCCTGGCAAAGACTTCTGCAAGATGATCTAT 2850

Lser_US96UC23 2801 CATAAATTCGCTTGAAAAGCCCTGGCAAAGACTTCTGCAAGATGATCTAT 2850

Lser_LAC005780 2801 CATAAATTCGCTTGAAAAGCCCTGGCAAAGACTTCTGCAAGATGATCTAT 2850

Lvir_CGN013357 2801 CATAAATTCGCTTGAAAAGCCCTGGCAAAGACTTCTGCAAGATGATCTAT 2850

Lsal_LAC008020 2801 CATAAATTCGCTTGAAAAGCCCTGGCAAAGACTTCTGCAAGATGATCTAT 2850

Lsat_Salinas 2851 TTTTCCATAGAAATATATTCGTTCAATAAGGGCTCCAGAAGATGTTGATC 2900

Lsat_WENDEL 2851 TTTTCCATAGAAATATATTCGTTCAATAAGGGCTCCAGAAGATGTTGATC 2900

Lser_US96UC23 2851 TTTTCCATAGAAATATATTCGTTCAATAAGGGCTCCAGAAGATGTTGATC 2900

Lser_LAC005780 2851 TTTTCCATAGAAATATATTCGTTCAATAAGGGCTCCAGAAGATGTTGATC 2900

Lvir_CGN013357 2851 TTTTCCATAGAAATATATTCGTTCAATAAGGGCTCCAGAAGATGTTGATC 2900

Lsal_LAC008020 2851 TTTTCCATAGAAATATATTCGTTCAATAAGGGCTCCAGAAGATGTTGATC 2900

Lsat_Salinas 2901 GTAAGTGAGAAGATTGGTTACGGAGAAAGAGGAAGCCGGATTCATATTCA 2950

Lsat_WENDEL 2901 GTAAGTGAGAAGATTGGTTACGGAGAAAGAGGAAGCCGGATTCATATTCA 2950

Lser_US96UC23 2901 GTAAGTGAGAAGATTGGTTACGGAGAAAGAGGAAGCCGGATTCATATTCA 2950

Lser_LAC005780 2901 GTAAGTGAGAAGATTGGTTACGGAGAAAGAGGAAGCCGGATTCATATTCA 2950

Lvir_CGN013357 2901 GTAAGTGAGAAGATTGGTTACGGAGAAAGAGGAAGCCGGATTCATATTCA 2950

Lsal_LAC008020 2901 GTAAGTGAGAAGATTGGTTACGGAGAAAGAGGAAGCCGGATTCATATTCA 2950

Lsat_Salinas 2951 CATAAATGAGAAGTATATAGGAAGAAGAATAGTCTGTGATTTCTTTTTGA 3000

Lsat_WENDEL 2951 CATAAATGAGAAGTATATAGGAAGAAGAATAGTCTGTGATTTCTTTTTGA 3000

Lser_US96UC23 2951 CATAAATGAGAAGTATATAGGAAGAAGAATAGTCTGTGATTTCTTTTTGA 3000

Lser_LAC005780 2951 CATAAATGAGAAGTATATAGGAAGAAGAATAGTCTGTGATTTCTTTTTGA 3000

Lvir_CGN013357 2951 CATAAATGAGAAGTATATAGGAAGAAGAATAGTCTGTGATTTCTTTTTGA 3000

Lsal_LAC008020 2951 CATAAATGAGAAGTATATAGGAAGAAGAATAGTCTGTGATTTCTTTTTGA 3000

Lsat_Salinas 3001 AAAAGAAGAACTGGCTTTCTTTGAATTTGAAGTAATCAGACTATCCCAAT 3050

Lsat_WENDEL 3001 AAAAGAAGAACTGGCTTTCTTTGAATTTGAAGTAATCAGACTATCCCAAT 3050

Lser_US96UC23 3001 AAAAGAAGAACTGGCTTTCTTTGAATTTGAAGTAATCAGACTATCCCAAT 3050

Lser_LAC005780 3001 AAAAGAAGAACTGGCTTTCTTTGAATTTGAAGTAATCAGACTATCCCAAT 3050

Lvir_CGN013357 3001 AAAAGAAGAACTGGCTTTCTTTGAATTTGAAGTAATCAGACTATCCCAAT 3050

Lsal_LAC008020 3001 AAAAGAAGAACTGGCTTTCTTTGAATTTGAAGTAATCAGACTATCCCAAT 3050

Lsat_Salinas 3051 TATGACACTCATGGAGAAAGAATCTTAATAAATGCAAAGAGGAAGCATCT 3100

Lsat_WENDEL 3051 TATGACACTCATGGAGAAAGAATCTTAATAAATGCAAAGAGGAAGCATCT 3100

Lser_US96UC23 3051 TATGACACTCATGGAGAAAGAATCTTAATAAATGCAAAGAGGAAGCATCT 3100

Lser_LAC005780 3051 TATGACACTCATGGAGAAAGAATCTTAATAAATGCAAAGAGGAAGCATCT 3100

Lvir_CGN013357 3051 TATGACACTCATGGAGAAAGAATCTTAATAAATGCAAAGAGGAAGCATCT 3100

Lsal_LAC008020 3051 TATGACACTCATGGAGAAAGAATCTTAATAAATGCAAAGAGGAAGCATCT 3100

Lsat_Salinas 3101 TTTATCCAATAGCGAAGAGCCTGAACCAAGATTTCCAGATGGGCTGGGTA 3150

Lsat_WENDEL 3101 TTTATCCAATAGCGAAGAGCCTGAACCAAGATTTCCAGATGGGCTGGGTA 3150

Lser_US96UC23 3101 TTTATCCAATAGCGAAGAGCCTGAACCAAGATTTCCAGATGGGCTGGGTA 3150

Lser_LAC005780 3101 TTTATCCAATAGCGAAGAGCCTGAACCAAGATTTCCAGATGGGCTGGGTA 3150

Lvir_CGN013357 3101 TTTATCCAATAGCGAAGAGCCTGAACCAAGATTTCCAGATGGGCTGGGTA 3150

Lsal_LAC008020 3101 TTTATCCAATAGCGAAGAGCCTGAACCAAGATTTCCAGATGGGCTGGGTA 3150

Lsat_Salinas 3151 AGGTATTAGTATATCTAATACATAATTTAAATGTGAAAAGTTGTCCTCTA 3200

Lsat_WENDEL 3151 AGGTATTAGTATATCTAATACATAATTTAAATGTGAAAAGTTGTCCTCTA 3200

Lser_US96UC23 3151 AGGTATTAGTATATCTAATACATAATTTAAATGTGAAAAGTTGTCCTCTA 3200

Lser_LAC005780 3151 AGGTATTAGTATATCTAATACATAATTTAAATGTGAAAAGTTGTCCTCTA 3200

Lvir_CGN013357 3151 AGGTATTAGTATATCTAATACATAATTTAAATGTGAAAAGTTGTCCTCTA 3200

Lsal_LAC008020 3151 AGGTATTAGTATATCTAATACATAATTTAAATGTGAAAAGTTGTCCTCTA 3200

Lsat_Salinas 3201 AAAAAGAAAATATTGAATGAATTGATCGTAAATTATCGGATTTGACTACC 3250

Lsat_WENDEL 3201 AAAAAGAAAATATTGAATGAATTGATCGTAAATTATCGGATTTGACTACC 3250

Lser_US96UC23 3201 AAAAAGAAAATATTGAATGAATTGATCGTAAATTATCGGATTTGACTACC 3250

Lser_LAC005780 3201 AAAAAGAAAATATTGAATGAATTGATCGTAAATTATCGGATTTGACTACC 3250

Lvir_CGN013357 3201 AAAAAGAAAATATTGAATGAATTGATCGTAAATTATCGGATTTGACTACC 3250

Lsal_LAC008020 3201 AAAAAGAAAATATTGAATGAATTGATCGTAAATTATCGGATTTGACTACC 3250

Lsat_Salinas 3251 CCTTTCCTTTCTAGGTAAGATATTAATCGCAGAGACAATGGAATTTCCAT 3300

Lsat_WENDEL 3251 CCTTTCCTTTCTAGGTAAGATATTAATCGCAGAGACAATGGAATTTCCAT 3300

Lser_US96UC23 3251 CCTTTCCTTTCTAGGTAAGATATTAATCGCAGAGACAATGGAATTTCCAT 3300

Lser_LAC005780 3251 CCTTTCCTTTCTAGGTAAGATATTAATCGCAGAGACAATGGAATTTCCAT 3300

Lvir_CGN013357 3251 CCTTTCCTTTCTAGGTAAGATATTAATCGCAGAGACAATGGAATTTCCAT 3300

Lsal_LAC008020 3251 CCTTTCCTTTCTAGGTAAGATATTAATCGCAGAGACAATGGAATTTCCAT 3300

Lsat_Salinas 3301 AATGATTGAAGAGACCTCGGACATTACTTGCGAATAAAAATTCTTGTTGT 3350

Lsat_WENDEL 3301 AATGATTGAAGAGACCTCGGACATTACTTGCGAATAAAAATTCTTGTTGT 3350

Lser_US96UC23 3301 AATGATTGAAGAGACCTCGGACATTACTTGCGAATAAAAATTCTTGTTGT 3350

Lser_LAC005780 3301 AATGATTGAAGAGACCTCGGACATTACTTGCGAATAAAAATTCTTGTTGT 3350

Lvir_CGN013357 3301 AATGATTGAAGAGACCTCGGACATTACTTGCGAATAAAAATTTTTGTTGT 3350

Lsal_LAC008020 3301 AATGATTGAAGAGACCTCGGACATTACTTGCGAATAAAAATTCTTGTTGT 3350

Lsat_Salinas 3351 GCCCCAAAAATGGAGTCTGTTTAGAATTATTAACCGAAAGAATCAAATGA 3400

Lsat_WENDEL 3351 GCCCCAAAAATGGAGTCTGTTTAGAATTATTAACCGAAAGAATCAAATGA 3400

Lser_US96UC23 3351 GCCCCAAAAATGGAGTCTGTTTAGAATTATTAACCGAAAGAATCAAATGA 3400

Lser_LAC005780 3351 GCCCCAAAAATGGAGTCTGTTTAGAATTATTAACCGAAAGAATCAAATGA 3400

Lvir_CGN013357 3351 GCCCCAAAAATGGAGTCTGTTTAGAATTATTAACCGAAAGAATCAAATGA 3400

Lsal_LAC008020 3351 GCCCCAAAAATGGAGTCTGTTTAGAATTATTAACCGAAAGAATCAAATGA 3400

Lsat_Salinas 3401 TTCTGTTGATACATTCGATTGATTAAACGTTTCACAATTAGTAAGCTGGA 3450

Lsat_WENDEL 3401 TTCTGTTGATACATTCGATTGATTAAACGTTTCACAATTAGTAAGCTGGA 3450

Lser_US96UC23 3401 TTCTGTTGATACATTCGATTGATTAAACGTTTCACAATTAGTAAGCTGGA 3450

Lser_LAC005780 3401 TTCTGTTGATACATTCGATTGATTAAACGTTTCACAATTAGTAAGCTGGA 3450

Lvir_CGN013357 3401 TTCTGTTGATACATTCGATTGATTAAACGTTTCACAATTAGTAAGCTGGA 3450

Lsal_LAC008020 3401 TTCTGTTGATACATTCGATTGATTAAACGTTTCACAATTAGTAAGCTGGA 3450

Lsat_Salinas 3451 TTTATTGTCATAACCTGCATTTTCCAACAAAATCGATCTAGTTAAACCAT 3500

Lsat_WENDEL 3451 TTTATTGTCATAACCTGCATTTTCCAACAAAATCGATCTAGTTAAACCAT 3500

Lser_US96UC23 3451 TTTATTGTCATAACCTGCATTTTCCAACAAAATCGATCTAGTTAAACCAT 3500

Lser_LAC005780 3451 TTTATTGTCATAACCTGCATTTTCCAACAAAATCGATCTAGTTAAACCAT 3500

Lvir_CGN013357 3451 TTTATTGTCATAACCTGCATTTTCCAACAAAATCGATCTAGTTAAACCAT 3500

Lsal_LAC008020 3451 TTTATTGTCATAACCTGCATTTTCCAACAAAATCGATCTAGTTAAACCAT 3500

Lsat_Salinas 3501 GATCATGAGCAAGTACATAAATATACTCCTGAAAGATAAGTGGATATAAG 3550

Lsat_WENDEL 3501 GATCATGAGCAAGTACATAAATATACTCCTGAAAGATAAGTGGATATAAG 3550

Lser_US96UC23 3501 GATCATGAGCAAGTACATAAATATACTCCTGAAAGATAAGTGGATATAAG 3550

Lser_LAC005780 3501 GATCATGAGCAAGTACATAAATATACTCCTGAAAGATAAGTGGATATAAG 3550

Lvir_CGN013357 3501 GATCATGAGCAAGTACATAAATATACTCCTGAAAGATAAGTGGATATAAG 3550

Lsal_LAC008020 3501 GATCATGAGCAAGTACATAAATATACTCCTGAAAGATAAGTGGATATAAG 3550

Lsat_Salinas 3551 AAGTGGTGTTGTTGAGATCTATCTAGCCCTAAATAGCTTTGGAATTTCTC 3600

Lsat_WENDEL 3551 AAGTGGTGTTGTTGAGATCTATCTAGCCCTAAATAGCTTTGGAATTTCTC 3600

Lser_US96UC23 3551 AAGTGGTGTTGTTGAGATCTATCTAGCCCTAAATAGCTTTGGAATTTCTC 3600

Lser_LAC005780 3551 AAGTGGTGTTGTTGAGATCTATCTAGCCCTAAATAGCTTTGGAATTTCTC 3600

Lvir_CGN013357 3551 AAGTGGTGTTGTTGAGATCTATCTAGCCCTAAATAGCTTTGGAATTTCTC 3600

Lsal_LAC008020 3551 AAGTGGTGTTGTTGAGATCTATCTAGCCCTAAATAGCTTTGGAATTTCTC 3600

Lsat_Salinas 3601 CATTTGAAATTCTATTTAAATGAAAGGTAGAGGATTTTGGGGGTTATCAA 3650

Lsat_WENDEL 3601 CATTTGAAATTCTATTTAAATGAAAGGTAGAGGATTTTGGGGGTTATCAA 3650

Lser_US96UC23 3601 CATTTGAAATTCTATTTAAATGAAAGGTAGAGGATTTTGGGGGTTATCAA 3650

Lser_LAC005780 3601 CATTTGAAATTCTATTTAAATGAAAGGTAGAGGATTTTGGGGGTTATCAA 3650

Lvir_CGN013357 3601 CATTTGAAATTCTATTTAAATGAAAGGTAGAGGATTTTGGGGGTTAGCAA 3650

Lsal_LAC008020 3601 CATTTGAAATTCTATTTAAATGAAAGGTAGAGGATTTTGGGGGTTATCAA 3650

Lsat_Salinas 3651 ATGATACATAGTGCGATACAGTCAAAACAAGGTATTATAGTACAAACCGA 3700

Lsat_WENDEL 3651 ATGATACATAGTGCGATACAGTCAAAACAAGGTATTATAGTACAAACCGA 3700

Lser_US96UC23 3651 ATGATACATAGTGCGATACAGTCAAAACAAGGTATTATAGTACAAACCGA 3700

Lser_LAC005780 3651 ATGATACATAGTGCGATACAGTCAAAACAAGGTATTATAGTACAAACCGA 3700

Lvir_CGN013357 3651 ATGATACATAGTGCGATACAGTCAAAACAAGGTATTATAGTACAAACCGA 3700

Lsal_LAC008020 3651 ATGATACATAGTGCGATACAGTCAAAACAAGGTATTATAGTACAAACCGA 3700

Lsat_Salinas 3701 ATAGATACCTCGGATACAGATAAACTTATCAACAGATTCTCTAGCCTCTC 3750

Lsat_WENDEL 3701 ATAGATACCTCGGATACAGATAAACTTATCAACAGATTCTCTAGCCTCTC 3750

Lser_US96UC23 3701 ATAGATACCTCGGATACAGATAAACTTATCAACAGATTCTCTAGCCTCTC 3750

Lser_LAC005780 3701 ATAGATACCTCGGATACAGATAAACTTATCAACAGATTCTCTAGCCTCTC 3750

Lvir_CGN013357 3701 ATAGATACCTCGGATACAGATAAACTTATCAACAGATTCTCTATCCTCTC 3750

Lsal_LAC008020 3701 ATAGATACCTCGGATACAGATAAACTTATCAACAGATTCTCTATCCTCTC 3750

Lsat_Salinas 3751 TTTTTCCATTTCAATTTCAATATTTATGTTCGTTTTCATTATAGGATAAC 3800

Lsat_WENDEL 3751 TTTTTCCATTTCAATTTCAATATTTATGTTCGTTTTCATTATAGGATAAC 3800

Lser_US96UC23 3751 TTTTTCCATTTCAATTTCAATATTTATGTTCGTTTTCATTATAGGATAAC 3800

Lser_LAC005780 3751 TTTTTCCATTTCAATTTCAATATTTATGTTCGTTTTCATTATAGGATAAC 3800

Lvir_CGN013357 3751 TTTTTCCATTTCAATTTCAATATTTATGTTCGTTTTCATTATAGGATAAC 3800

Lsal_LAC008020 3751 TTTTTCCATTTCAATTTCAATATTGATGTTCGTTTTCATTATAGGATAAC 3800

Lsat_Salinas 3801 AAGATGATTAGAAATCCTTTACTTTTTTTTCAACCCAATCGCTCTTTTGA 3850

Lsat_WENDEL 3801 AAGATGATTAGAAATCCTTTACTTTTTTTTCAACCCAATCGCTCTTTTGA 3850

Lser_US96UC23 3801 AAGATGATTAGAAATCCTTTACTTTTTTTTCAACCCAATCGCTCTTTTGA 3850

Lser_LAC005780 3801 AAGATGATTAGAAATCCTTTACTTTTTTTTCAACCCAATCGCTCTTTTGA 3850

Lvir_CGN013357 3801 AAGATGATTAGAAATCCTTTACTTTTTTTTCAACCCAATCGCTCTTTTGA 3850

Lsal_LAC008020 3801 AAGATGATTAGAAATCCTTTACTTTTTTTTCAACCCAATCGCTCTTTTGA 3850

Lsat_Salinas 3851 TTTTGGAAATTTTTTATCAATATACTGTTTCTTATACACACACATCTCCA 3900

Lsat_WENDEL 3851 TTTTGGAAATTTTTTATCAATATACTGTTTCTTATACACACACATCTCCA 3900

Lser_US96UC23 3851 TTTTGGAAATTTTTTATCAATATACTGTTTCTTATACACACACATCTCCA 3900

Lser_LAC005780 3851 TTTTGGAAATTTTTTATCAATATACTGTTTCTTATACACACACATCTCCA 3900

Lvir_CGN013357 3851 TTTTGGAAATTTTTTATCAATATACTGTTTCTTATACACACACATCTCCA 3900

Lsal_LAC008020 3851 TTTTGGAAATTTTTTATCAATATACTGTTTCTTATACACACACATCTCCA 3900

Lsat_Salinas 3901 TTATGGAATGGAGAATGGTAATATTTAGGATTCATTAAAAAATCAAGAAT 3950

Lsat_WENDEL 3901 TTATGGAATGGAGAATGGTAATATTTAGGATTCATTAAAAAATCAAGAAT 3950

Lser_US96UC23 3901 TTATGGAATGGAGAATGGTAATATTTAGGATTCATTAAAAAATCAAGAAT 3950

Lser_LAC005780 3901 TTATGGAATGGAGAATGGTAATATTTAGGATTCATTAAAAAATCAAGAAT 3950

Lvir_CGN013357 3901 TTATGGAATGGAGAATGGTAATATTTAGGATTCATTAAAAAATCAAGAAT 3950

Lsal_LAC008020 3901 TTATGGAATGGAGAATGGTAATATTTAGGATTCATTAAAAAATCAAGAAT 3950

Lsat_Salinas 3951 ACATTCCTTCCTGGGAGAAAGCCTTCCCGTATTGGGCACTAATATTTTTT 4000

Lsat_WENDEL 3951 ACATTCCTTCCTGGGAGAAAGCCTTCCCGTATTGGGCACTAATATTTTTT 4000

Lser_US96UC23 3951 ACATTCCTTCCTGGGAGAAAGCCTTCCCGTATTGGGCACTAATATTTTTT 4000

Lser_LAC005780 3951 ACATTCCTTCCTGGGAGAAAGCCTTCCCGTATTGGGCACTAATATTTTTT 4000

Lvir_CGN013357 3951 ACATTCCTTCCTGGGAGAAAGCCTTCCCGTATTGGGCACTAATATTTTTT 4000

Lsal_LAC008020 3951 ACATTCCTTCCTGGGAGAAAGCCTTCCCGTATTGGGCACTAATATTTTTT 4000

Lsat_Salinas 4001 TAACGTCTAATTAGATCGGGTAATCATTCAAATTAAGAACGGAAGCTCGT 4050

Lsat_WENDEL 4001 TAACGTCTAATTAGATCGGGTAATCATTCAAATTAAGAACGGAAGCTCGT 4050

Lser_US96UC23 4001 TAACGTCTAATTAGATCGGGTAATCATTCAAATTAAGAACGGAAGCTCGT 4050

Lser_LAC005780 4001 TAACGTCTAATTAGATCGGGTAATCATTCAAATTAAGAACGGAAGCTCGT 4050

Lvir_CGN013357 4001 TAACGTCTAATTAGATCGGGTAATCATTCAAATTAAGAACGGAAGCTCGT 4050

Lsal_LAC008020 4001 TAACGTCTAATTAGATCGGGTAATCATTCAAATTAAGAACGGAAGCTCGT 4050

Lsat_Salinas 4051 TGCTTTTTCTTTCCCTATAATCAAAATGAAATTAATTGAAGCCGCGGGGC 4100

Lsat_WENDEL 4051 TGCTTTTTCTTTCCCTATAATCAAAATGAAATTAATTGAAGCCGCGGGGC 4100

Lser_US96UC23 4051 TGCTTTTTCTTTCCCTATAATCAAAATGAAATTAATTGAAGCCGCGGGGC 4100

Lser_LAC005780 4051 TGCTTTTTCTTTCCCTATAATCAAAATGAAATTAATTGAAGCCGCGGGGC 4100

Lvir_CGN013357 4051 TGCTTTTTCTTTCCCTATAATCAAAATGAAATTAATTGAAGCCGCGGGGC 4100

Lsal_LAC008020 4051 TGCTTTTTCTTTCCCTATAATCAAAATGAAATTAATTGAAGCCGCAGGGC 4100

Lsat_Salinas 4101 CCTATCCATTTATTAATTCGACCCAACTTGAAATTGACTTCGGTTTGCTC 4150

Lsat_WENDEL 4101 CCTATCCATTTATTAATTCGACCCAACTTGAAATTGACTTCGGTTTGCTC 4150

Lser_US96UC23 4101 CCTATCCATTTATTAATTCGACCCAACTTGAAATTGACTTCGGTTTGCTC 4150

Lser_LAC005780 4101 CCTATCCATTTATTAATTCGACCCAACTTGAAATTGACTTCGGTTTGCTC 4150

Lvir_CGN013357 4101 CCTATCCATTTATTAATTCGACCCAACTTGAAATTGACTTCGGTTTGCTC 4150

Lsal_LAC008020 4101 CCTATCCATTTATTAATTCGACCCAACTTGAAATTGACTTCGGTTTGCTC 4150

Lsat_Salinas 4151 CCTTTCACTAATTTAAAGAAGGTTTTGTACCGAGCCGGAATAAATTATTC 4200

Lsat_WENDEL 4151 CCTTTCACTAATTTAAAGAAGGTTTTGTACCGAGCCGGAATAAATTATTC 4200

Lser_US96UC23 4151 CCTTTCACTAATTTAAAGAAGGTTTTGTACCGAGCCGGAATAAATTATTC 4200

Lser_LAC005780 4151 CCTTTCACTAATTTAAAGAAGGTTTTGTACCGAGCCGGAATAAATTATTC 4200

Lvir_CGN013357 4151 CCTTTCACTAATTTAAAGAAGGTTTTGTACCGAGCCGGAATAAATTATTC 4200

Lsal_LAC008020 4151 CCTTTCACTAATTTAAAGAAGGTTTTGTACCGAGCCGGAATAAATTATTC 4200

Lsat_Salinas 4201 TCAGAACTCTTAATTGATACGACATGCTATTTTTTCCATTCATTCCCTTT 4250

Lsat_WENDEL 4201 TCAGAACTCTTAATTGATACGACATGCTATTTTTTCCATTCATTCCCTTT 4250

Lser_US96UC23 4201 TCAGAACTCTTAATTGATACGACATGCTATTTTTTCCATTCATTCCCTTT 4250

Lser_LAC005780 4201 TCAGAACTCTTAATTGATACGACATGCTATTTTTTCCATTCATTCCCTTT 4250

Lvir_CGN013357 4201 TCAGAACTCTTAATTGATACGACATGCTATTTTTTCCATTCATTCCCTTT 4250

Lsal_LAC008020 4201 TCAGAACTCTTAATTGATACGACATGCTATTTTTTCCATTCATTCCCTTT 4250

Lsat_Salinas 4251 CAGGATCAGTCGTGGTCTTCCAAACTTTACCGATGGTATGGATGAATCCC 4300

Lsat_WENDEL 4251 CAGGATCAGTCGTGGTCTTCCAAACTTTACCGATGGTATGGATGAATCCC 4300

Lser_US96UC23 4251 CAGGATCAGTCGTGGTCTTCCAAACTTTACCGATGGTATGGATGAATCCC 4300

Lser_LAC005780 4251 CAGGATCAGTCGTGGTCTTCCAAACTTTACCGATGGTATGGATGAATCCC 4300

Lvir_CGN013357 4251 CAGGATCAGTCGTGGTCTTCCAAACTTTACCGATGGTATGGATGAATCCC 4300

Lsal_LAC008020 4251 CAGGATCAGTCGTGGTCTTCCAAACTTTACCGATGGTATGGATGAATCCC 4300

Lsat_Salinas 4301 TCGCTTCATCTAATGTGTAAAAGATCCTAGCCGCACTTAAAAGCCGAGTA 4350

Lsat_WENDEL 4301 TCGCTTCATCTAATGTGTAAAAGATCCTAGCCGCACTTAAAAGCCGAGTA 4350

Lser_US96UC23 4301 TCGCTTCATCTAATGTGTAAAAGATCCTAGCCGCACTTAAAAGCCGAGTA 4350

Lser_LAC005780 4301 TCGCTTCATCTAATGTGTAAAAGATCCTAGCCGCACTTAAAAGCCGAGTA 4350

Lvir_CGN013357 4301 TCGCTTCATCTAATGTGTAAAAGATCCTAGCCGCACTTAAAAGCCGAGTA 4350

Lsal_LAC008020 4301 TCGCTTCATCTAATGTGTAAAAGATCCTAGCCGCACTTAAAAGCCGAGTA 4350

Lsat_Salinas 4351 CTCTACCGTTGAGTTAGCAACCCAAATAGAAAACGGGTATGTAGATACAA 4400

Lsat_WENDEL 4351 CTCTACCGTTGAGTTAGCAACCCAAATAGAAAACGGGTATGTAGATACAA 4400

Lser_US96UC23 4351 CTCTACCGTTGAGTTAGCAACCCAAATAGAAAACGGGTATGTAGATACAA 4400

Lser_LAC005780 4351 CTCTACCGTTGAGTTAGCAACCCAAATAGAAAACGGGTATGTAGATACAA 4400

Lvir_CGN013357 4351 CTCTACCGTTGAGTTAGCAACCCAAATAGAAAACGGGTATGTAGATACAA 4400

Lsal_LAC008020 4351 CTCTACCGTTGAGTTAGCAACCCAAATAGAAAACGGGTATGTAGATACAA 4400

Lsat_Salinas 4401 TCAGAATAAAACAAAATGATGAAATAAAAGCATTAAAGTACGAAATCAAA 4450

Lsat_WENDEL 4401 TCAGAATAAAACAAAATGATGAAATAAAAGCATTAAAGTACGAAATCAAA 4450

Lser_US96UC23 4401 TCAGAATAAAACAAAATGATGAAATAAAAGCATTAAAGTACGAAATCAAA 4450

Lser_LAC005780 4401 TCAGAATAAAACAAAATGATGAAATAAAAGCATTCAAGTACGAAATCAAA 4450

Lvir_CGN013357 4401 TCAGAATAAAACAAAATGATGAAATAAAAGCATTAAAGTACGAAATCAAA 4450

Lsal_LAC008020 4401 TCAGAATAAAACAAAATGATGAAATAAAAGCATTAAAGTACGAAATCAAA 4450

Lsat_Salinas 4451 AAATATATAAAAAAATTAGAATCTATTTGGAACATAAAAATGACTAAATC 4500

Lsat_WENDEL 4451 AAATATATAAAAAAATTAGAATCTATTTGGAACATAAAAATGACTAAATC 4500

Lser_US96UC23 4451 AAATATATAAAAAAATTAGAATCTATTTGGAACATAAAAATGACTAAATC 4500

Lser_LAC005780 4451 AAATATATAAAAAAATTAGAATCTATTTGGAACATAAAAATGACTAAATC 4500

Lvir_CGN013357 4451 AAATATATATAAAAATTAGAATCTAKTTGGAACATAAAAATGACTAAATC 4500

Lsal_LAC008020 4451 AAATATATAAAAAAATTAGAATCTATTTGGAACATAAAAATGACTAAATC 4500

Lsat_Salinas 4501 AGATGAAAAAATAAAAAATTTTCCGATAAAAAAAGAAATAAAGAAATCGA 4550

Lsat_WENDEL 4501 AGATGAAAAAATAAAAAATTTTCCGATAAAAAAAGAAATAAAGAAATCGA 4550

Lser_US96UC23 4501 AGATGAAAAAATAAAAAATTTTCCGATAAAAAAAGAAATAAAGAAATCGA 4550

Lser_LAC005780 4501 AGATGAAAAAATAAAAAATTTTCCGATAAAAAAAGAAATAAAGAAATGGA 4550

Lvir_CGN013357 4501 AGATGAAAAAATAAAAAATTTTCCGATAAAAAAAGAAATAAAGAAATGGA 4550

Lsal_LAC008020 4501 AGATGAAAAAATAAAAAATTTTCCGATAAAAAAAGAAATAAAGAAATGGA 4550

Lsat_Salinas 4551 AAAAATGCTAGACCATCCCCTATTTCTATGTTATCCACCTTTTTCAATCA 4600

Lsat_WENDEL 4551 AAAAATGCTAGACCATCCCCTATTTCTATGTTATCCACCTTTTTCAATCA 4600

Lser_US96UC23 4551 AAAAATGCTAGACCATCCCCTATTTCTATGTTATCCACCTTTTTCAATCA 4600

Lser_LAC005780 4551 AAAAATGCTAGACCATCCCCTATTTCTATGTTATCCACCTTTTTCAATCA 4600

Lvir_CGN013357 4551 AAAAATGCTAGACCATCCCCTATTTCTATGTTATCCACCTTTTTCAATCA 4600

Lsal_LAC008020 4551 AAAAATGCTAAACCATCCCCTATTTCTATGTTATCCACCTTTTTCAATCA 4600

Lsat_Salinas 4601 AAAATGCGGGTATCAAAGCTAATCCAACGAACCCATAATTTGAATCAACT 4650

Lsat_WENDEL 4601 AAAATGCGGGTATCAAAGCTAATCCAACGAACCCATAATTTGAATCAACT 4650

Lser_US96UC23 4601 AAAATGCGGGTATCAAAGCTAATCCAACGAACCCATAATTTGAATCAACT 4650

Lser_LAC005780 4601 AAAATGCGGGTATCAAAGCTAATCCAACGAACCCATAATTTGAATCAACT 4650

Lvir_CGN013357 4601 AAAATGCGGGTATCAAAGCTAATCCAACGAACCCATAATTTGAATCAACT 4650

Lsal_LAC008020 4601 AAAATGCGGGTATCAAAGCTAATCCAACGAACCCATAATTTGAATCAACT 4650

Lsat_Salinas 4651 AAGGTAAAAAATCAAATGGGACGGAAATAAATAGAGCTGCTTATCACGAT 4700

Lsat_WENDEL 4651 AAGGTAAAAAATCAAATGGGACGGAAATAAATAGAGCTGCTTATCACGAT 4700

Lser_US96UC23 4651 AAGGTAAAAAATCAAATGGGACGGAAATAAATAGAGCTGCTTATCACGAT 4700

Lser_LAC005780 4651 AAGGTAAAAAATCAAATGGGACGGAAATAAATAGAGCTGCTTATCACGAT 4700

Lvir_CGN013357 4651 AAGGTAAAAAATCAAATGGGACGGAAATAAATAGAGCTGCTTATCACGAT 4700

Lsal_LAC008020 4651 AAGGTAAAAAATCAAATGGGACGGAAATAAATAGAGCTGCTTATCACGAT 4700

Lsat_Salinas 4701 GAATTATATTTGTTCGATACAATGTTGTCAATATAAAGGGTAAGAAAAGA 4750

Lsat_WENDEL 4701 GAATTATATTTGTTCGATACAATGTTGTCAATATAAAGGGTAAGAAAAGA 4750

Lser_US96UC23 4701 GAATTATATTTGTTCGATACAATGTTGTCAATATAAAGGGTAAGAAAAGA 4750

Lser_LAC005780 4701 GAATTATATTTGTTCGATACAATGTTGTCAATATAAAGGGTAAGAAAAGA 4750

Lvir_CGN013357 4701 GAATTATATTTGTTCGATACAATGTTGTCAATATAAAGGGTAAGAAAAGA 4750

Lsal_LAC008020 4701 GAATTATATTTGTTCGATACAATGTTGTCAATATAAAGGGTAAGAAAAGA 4750

Lsat_Salinas 4751 ATACGATGGAAAAAATACAAGAACTTAAATTGAAATAATAGTAAAAAAAA 4800

Lsat_WENDEL 4751 ATACGATGGAAAAAATACAAGAACTTAAATTGAAATAATAGTAAAAAAAA 4800

Lser_US96UC23 4751 ATACGATGGAAAAAATACAAGAACTTAAATTGAAATAATAGTAAAAAAAA 4800

Lser_LAC005780 4751 ATACGATGGAAAAAATACAAGAACTTAAATTGAAATAATAGTAAAAAAAA 4800

Lvir_CGN013357 4751 ATACGATGGAAAAAATACAAGAACTTAAATTGAAATAATAGTAAAAAAAA 4800

Lsal_LAC008020 4751 ATACGATGGAAAAAATACAAGAACTTAAATTGAAATAATAGTAAAAAAAC 4800

Lsat_Salinas 4801 GGACTTGTGTTGGATTGGCACTGCATATATACTAAAATTTTTAGTTTCTA 4850

Lsat_WENDEL 4801 GGACTTGTGTTGGATTGGCACTGCATATATACTAAAATTTTTAGTTTCTA 4850

Lser_US96UC23 4801 GGACTTGTGTTGGATTGGCACTGCATATATACTAAAATTTTTAGTTTCTA 4850

Lser_LAC005780 4801 GGACTTGTGTTGGATTGGCACTGCATATATACTAAAATTTTTAGTTTCTA 4850

Lvir_CGN013357 4801 GGACTTGTGTTGGATTGGCACTGCATATATACTAAAATTTTTAGTTTCTA 4850

Lsal_LAC008020 4801 GGACTTGTGTTGGATTGGCACTGCATATATACTAAAATTTTTAGTTTCTA 4850

Lsat_Salinas 4851 AATAAAGAAGAAGTAGAAAAAGGATTTATGCAATCAATAGTATATTGAAT 4900

Lsat_WENDEL 4851 AATAAAGAAGAAGTAGAAAAAGGATTTATGCAATCAATAGTATATTGAAT 4900

Lser_US96UC23 4851 AATAAAGAAGAAGTAGAAAAAGGATTTATGCAATCAATAGTATATTGAAT 4900

Lser_LAC005780 4851 AATAAAGAAGAAGTAGAAAAAGGATTTATGCAATCAATAGTATATTGAAT 4900

Lvir_CGN013357 4851 AATAAAGAAGAAGTAGAAAAAGGATTTATGCAATCAATAGTGTATTGAAT 4900

Lsal_LAC008020 4851 AATAAAGAAGAAGTAGAAAAAGGATTTATGCAATCAATAGTGTATTGAAT 4900

Lsat_Salinas 4901 TAATATAAGTCGAATAAAGAACTTCGATTCCTTGTGTCTTACTTGGTATT 4950

Lsat_WENDEL 4901 TAATATAAGTCGAATAAAGAACTTCGATTCCTTGTGTCTTACTTGGTATT 4950

Lser_US96UC23 4901 TAATATAAGTCGAATAAAGAACTTCGATTCCTTGTGTCTTACTTGGTATT 4950

Lser_LAC005780 4901 TAATATAAGTCGAATAAAGAACTTCGATTCCTTGTGTCTTACTTGGTATT 4950

Lvir_CGN013357 4901 TAATATAAGTCGAATAAAGAACTTCGATTCCTTGTGTCTTACTTGGTATT 4950

Lsal_LAC008020 4901 TAATATAAGTCGAATAAAGAACTTCGATTCCTTGTGTCTTACTTGGTATT 4950

Lsat_Salinas 4951 AGAATTTAGAATTCGAATGAAAACCACCCGATTGTAGGCAAGAATTTTTG 5000

Lsat_WENDEL 4951 AGAATTTAGAATTCGAATGAAAACCACCCGATTGTAGGCAAGAATTTTTG 5000

Lser_US96UC23 4951 AGAATTTAGAATTCGAATGAAAACCACCCGATTGTAGGCAAGAATTTTTG 5000

Lser_LAC005780 4951 AGAATTTAGAATTCGAATGAAAACCACCCGATTGTAGGCAAGAATTTTTG 5000

Lvir_CGN013357 4951 AGAATTTAGAATTCGAATGAAAACCACCCGATTGTAGGCAAGAATTTTTG 5000

Lsal_LAC008020 4951 AGAATTTAGAATTCGAATGAAAACCACCCGATTGTAGGCAAGAATTTTTG 5000

Lsat_Salinas 5001 ATTTTGTGAGGATCAATCAAATAAGGTTTTCGTTAGAAAAAGATTCTATA 5050

Lsat_WENDEL 5001 ATTTTGTGAGGATCAATCAAATAAGGTTTTCGTTAGAAAAAGATTCTATA 5050

Lser_US96UC23 5001 ATTTTGTGAGGATCAATCAAATAAGGTTTTCGTTAGAAAAAGATTCTATA 5050

Lser_LAC005780 5001 ATTTTGTGAGGATCAATCAAATAAGGTTTTCGTTAGAAAAAGATTCTATA 5050

Lvir_CGN013357 5001 ATTTTGTGAGGATCAATCAAATAAGGTTTTCGTTAGAAAAAGATTCTATA 5050

Lsal_LAC008020 5001 ATTTTGTGAGGATCAATCAAATAAGGTTTTCGTTAGAAAAAGATTCTATA 5050

Lsat_Salinas 5051 AAAGAAATGATAAATAGATACTAATAGAGCAAGAAAAGAAGGAAATAAAA 5100

Lsat_WENDEL 5051 AAAGAAATGATAAATAGATACTAATAGAGCAAGAAAAGAAGGAAATAAAA 5100

Lser_US96UC23 5051 AAAGAAATGATAAATAGATACTAATAGAGCAAGAAAAGAAGGAAATAAAA 5100

Lser_LAC005780 5051 AAAGAAATGATAAATAGATACTAATAGAGCAAGAAAAGAAGGAAATAAAA 5100

Lvir_CGN013357 5051 AAAGAAATGATAAATAGATACTAATAGAGCAAGAAAAGAAGGAAATAAAA 5100

Lsal_LAC008020 5051 AAAGAAATGATAAATAGATACTAATAGAGCAAGAAAAGAAGGAAATAAAA 5100

Lsat_Salinas 5101 AAAACATAGTATAGAAAAGATATCCAAAATTATATAGAAATCACTATCCT 5150

Lsat_WENDEL 5101 AAAACATAGTATAGAAAAGATATCCAAAATTATATAGAAATCACTATCCT 5150

Lser_US96UC23 5101 AAAACATAGTATAGAAAAGATATCCAAAATTATATAGAAATCACTATCCT 5150

Lser_LAC005780 5101 AAAACATAGTATAGAAAAGATATCCAAAATTATATAGAAATCACTATCCT 5150

Lvir_CGN013357 5101 AAAACATAGTATAGAAAAGATATCCAAAATTATATAGAAATCACTATCCT 5150

Lsal_LAC008020 5101 AAAACATAGTATAGAAAAGATATCCAAAATTATATAGAAATCACTATCCT 5150

Lsat_Salinas 5151 ACCCTTAGTTTTTATTTCCTTAATTTAATTCCTTAATTTAATTGAATTTC 5200

Lsat_WENDEL 5151 ACCCTTAGTTTTTATTTCCTTAATTTAATTCCTTAATTTAATTGAATTTC 5200

Lser_US96UC23 5151 ACCCTTAGTTTTTATTTCCTTAATTTAATTCCTTAATTTAATTGAATTTC 5200

Lser_LAC005780 5151 ACCCTTAGTTTTTATTTCCTTAATTTAATTCCTTAATTTAATTGAATTTC 5200

Lvir_CGN013357 5151 ACCCTTAGTTTTTATTTCCTTAATTTAATTCCTTAATTTAATTGAATTTC 5200

Lsal_LAC008020 5151 ACCCTTAGTTTTTATTTCCTTAATTTAATTCCTTAATTTAATTGAATTTC 5200

Lsat_Salinas 5201 GTTTGATTAGGGCAAAGTTCCTTAAAAACCTCTGCCTTTTTTAAAATATC 5250

Lsat_WENDEL 5201 GTTTGATTAGGGCAAAGTTCCTTAAAAACCTCTGCCTTTTTTAAAATATC 5250

Lser_US96UC23 5201 GTTTGATTAGGGCAAAGTTCCTTAAAAACCTCTGCCTTTTTTAAAATATC 5250

Lser_LAC005780 5201 GTTTGATTAGGGCAAAGTTCCTTAAAAACCTCTGCCTTTTTTAAAATATC 5250

Lvir_CGN013357 5201 GTTTGATTAGGGCAAAGTTCCTTAAAAACCTCTGCCTTTTTTAAAATATC 5250

Lsal_LAC008020 5201 GTTTGATTAGGGCAAAGTTCCTTAAAAACCTCTGCCTTTTTTAAAATATC 5250

Lsat_Salinas 5251 CTGAACAGTTCCTGTAGGCTGAGCGCCTTTTTCAAGGAAATAGAGAATAG 5300

Lsat_WENDEL 5251 CTGAACAGTTCCTGTAGGCTGAGCGCCTTTTTCAAGGAAATAGAGAATAG 5300

Lser_US96UC23 5251 CTGAACAGTTCCTGTAGGCTGAGCGCCTTTTTCAAGGAAATAGAGAATAG 5300

Lser_LAC005780 5251 CTGAACAGTTCCTGTAGGCTGAGCGCCTTTTTCAAGGAAATAGAGAATAG 5300

Lvir_CGN013357 5251 CTGAACAGTTCCTGTAGGCTGAGCGCCTTTTTCAAGGAAATAGAGAATAG 5300

Lsal_LAC008020 5251 CTGAACAGTTCCTGTAGGCTGAGCGCCTTTTTCAAGGAAATAGAGAATAG 5300

Lsat_Salinas 5301 CGGGAACGTTTAAATAAGTTTGATTCTTGATCGGATCATAAAAACCCACT 5350

Lsat_WENDEL 5301 CGGGAACGTTTAAATAAGTTTGATTCTTGATCGGATCATAAAAACCCACT 5350

Lser_US96UC23 5301 CGGGAACGTTTAAATAAGTTTGATTCTTGATCGGATCATAAAAACCCACT 5350

Lser_LAC005780 5301 CGGGAACGTTTAAATAAGTTTGATTCTTGATCGGATCATAAAAACCCACT 5350

Lvir_CGN013357 5301 CGGGAACGTTTAAATAAGTTTGATTCTTGATCGGATCATAAAAACCCACT 5350

Lsal_LAC008020 5301 CGGGAACGTTTAAATAAGTTTGATTCTTGATCGGATCATAAAAACCCACT 5350

Lsat_Salinas 5351 TTCCGAAGATCTCTTCCTTCTCTTCGGGATCGAACATCAATTGCAACGAT 5400

Lsat_WENDEL 5351 TTCCGAAGATCTCTTCCTTCTCTTCGGGATCGAACATCAATTGCAACGAT 5400

Lser_US96UC23 5351 TTCCGAAGATCTCTTCCTTCTCTTCGGGATCGAACATCAATTGCAACGAT 5400

Lser_LAC005780 5351 TTCCGAAGATCTCTTCCTTCTCTTCGGGATCGAACATCAATTGCAACGAT 5400

Lvir_CGN013357 5351 TTCCGAAGATCTCTTCCTTCTCTTCGGGATCGAACATCAATTGCAACGAT 5400

Lsal_LAC008020 5351 TTCCGAAGATCTCTTCCTTCTCTTCGGGATCGAACATCAATTGCAACGAT 5400

Lsat_Salinas 5401 TCGATAGACGGCTCATCGGGATAGATGTAGATGAAAAAGAACCCCCCCCT 5450

Lsat_WENDEL 5401 TCGATAGACGGCTCATCGGGATAGATGTAGATGAAAAAGAACCCCCCCCT 5450

Lser_US96UC23 5401 TCGATAGACGGCTCATCGGGATAGATGTAGATGAAAAAGAACCCCCCCCT 5450

Lser_LAC005780 5401 TCGATAGACGGCTCATCGGGATAGATGTAGATGAAAAAGAACCCCCCCCT 5450

Lvir_CGN013357 5401 TCGATAGACGGCTCATCGGGATAGATGTAGATGAAAAAGAACCCCCCCCT 5450

Lsal_LAC008020 5401 TCGATAGACGGCTCATCGGGATAGATGTAGATGAAAAAGAACCCCCCCCT 5450

Lsat_Salinas 5451 AGAACCGTATAGGAAGTTTTCTCCTCGTACGGCTCGAGAAAAAATGATTT 5500

Lsat_WENDEL 5451 AGAACCGTATAGGAAGTTTTCTCCTCGTACGGCTCGAGAAAAAATGATTT 5500

Lser_US96UC23 5451 AGAACCGTATAGGAAGTTTTCTCCTCGTACGGCTCGAGAAAAAATGATTT 5500

Lser_LAC005780 5451 AGAACCGTATAGGAAGTTTTCTCCTCGTACGGCTCGAGAAAAAATGATTT 5500

Lvir_CGN013357 5451 AGAACCGTATAGGAAGTTTTCTCCTCGTACGGCTCGAGAAAAAATGATTT 5500

Lsal_LAC008020 5451 AGAACCGTATAGGAAGTTTTCTCCTCGTACGGCTCGAGAAAAAATGATTT 5500

Lsat_Salinas 5501 GAAGTTTTGTCTATGGATAAAATTATAATAAATAGTAAAGGAATCCGTAA 5550

Lsat_WENDEL 5501 GAAGTTTTGTCTATGGATAAAATTATAATAAATAGTAAAGGAATCCGTAA 5550

Lser_US96UC23 5501 GAAGTTTTGTCTATGGATAAAATTATAATAAATAGTAAAGGAATCCGTAA 5550

Lser_LAC005780 5501 GAAGTTTTGTCTATGGATAAAATTATAATAAATAGTAAAGGAATCCGTAA 5550

Lvir_CGN013357 5501 GAAGTTTTGTCTATGGATAAAATTATAATAAATAGTAAAGGAATCCGTAA 5550

Lsal_LAC008020 5501 GAAGTTTTGTCTATGGATAAAATTATAATAAATAGTAAAGGAATCCGTAA 5550

Lsat_Salinas 5551 AATAAATTAGCCTATAATTTAACTTATAGTCATTTTTATTTAACTTACTT 5600

Lsat_WENDEL 5551 AATAAATTAGCCTATAATTTAACTTATAGTCATTTTTATTTAACTTACTT 5600

Lser_US96UC23 5551 AATAAATTAGCCTATAATTTAACTTATAGTCATTTTTATTTAACTTACTT 5600

Lser_LAC005780 5551 AATAAATTAGCCTATAATTTAACTTATAGTCATTTTTATTTAACTTACTT 5600

Lvir_CGN013357 5551 AAGAAATTAGCCTATAATTTAACTCATAGTCATTTTTATTTAACTTCCTT 5600

Lsal_LAC008020 5551 AAGAAATTAGCCTATAATTTAACTCATAGTCATTTTTATTTAACTTCCTT 5600

Lsat_Salinas 5601 ACTGAAAACTAAAAAATCATTTGTACTCATAACTCAAGTTCAATAATTAT 5650

Lsat_WENDEL 5601 ACTGAAAACTAAAAAATCATTTGTACTCATAACTCAAGTTCAATAATTAT 5650

Lser_US96UC23 5601 ACTGAAAACTAAAAAATCATTTGTACTCATAACTCAAGTTCAATAATTAT 5650

Lser_LAC005780 5601 ACTGAAAACTAAAAAATCATTTGTACTCATAACTCAAGTTCAATAATTAT 5650

Lvir_CGN013357 5601 ACTGAAAACTAAAAAATCATTTGTACTCATAACTCAAGTTCAATAATTAT 5650

Lsal_LAC008020 5601 ACTGAAAACTAAAAAATCATTTGTACTCATAACTCAAGTTCAATAATTAT 5650

Lsat_Salinas 5651 CAAATATATTAAATAAAATTAAGATATTTTTTTATTGAGTGGTCTTTAAC 5700

Lsat_WENDEL 5651 CAAATATATTAAATAAAATTAAGATATTTTTTTATTGAGTGGTCTTTAAC 5700

Lser_US96UC23 5651 CAAATATATTAAATAAAATTAAGATATTTTTTTATTGAGTGGTCTTTAAC 5700

Lser_LAC005780 5651 CAAATATATTAAATAAAATTAAGATATTTTTTTATTGAGTGGTCTTTAAC 5700

Lvir_CGN013357 5651 CAAATATATTAAATAAAATTAAGATATTTTTTTATTGAGTGGTCTTTAAC 5700

Lsal_LAC008020 5651 CAAATATATTAAATAAAATTAAGATATTTTTTTATTGAGTGGTCTTTAAC 5700

Lsat_Salinas 5701 CCCCCCTTTTGTCTCGTTGAAAATCTATTTGGATTCTTTATTCGGATCTG 5750

Lsat_WENDEL 5701 CCCCCCTTTTGTCTCGTTGAAAATCTATTTGGATTCTTTATTCGGATCTG 5750

Lser_US96UC23 5701 CCCCCCTTTTGTCTCGTTGAAAATCTATTTGGATTCTTTATTCGGATCTG 5750

Lser_LAC005780 5701 CCCCCCTTTTGTCTCGTTGAAAATCTATTTGGATTCTTTATTCGGATCTG 5750

Lvir_CGN013357 5701 CCCCCCTTTTGTCTCGTTGAAAATCTATTTGGATTCTTTATTCGGATCTG 5750

Lsal_LAC008020 5701 CCCCCCTTTTGTCTCGTTGAAAATCTATTTGGATTCTTTATTCGGATCTG 5750

Lsat_Salinas 5751 TGAGACAATTGAAGTGGTGTTTCCTTGTTCTGGGATCCTTTATCTTTGTT 5800

Lsat_WENDEL 5751 TGAGACAATTGAAGTGGTGTTTCCTTGTTCTGGGATCCTTTATCTTTGTT 5800

Lser_US96UC23 5751 TGAGACAATTGAAGTGGTGTTTCCTTGTTCTGGGATCCTTTATCTTTGTT 5800

Lser_LAC005780 5751 TGAGACAATTGAAGTGGTGTTTCCTTGTTCTGGGATCCTTTATCTTTGTT 5800

Lvir_CGN013357 5751 TGAGACAATTGAAGCGGTGTTTCCTTGTTCTGGGATCCTTTATCTYTGTT 5800

Lsal_LAC008020 5751 TGAGACAATTGAAGCGGTGTTTCCTTGTTCTGGGATCCTTTATCTTTGTT 5800

Lsat_Salinas 5801 TTAAATCATTGGGTTTAGACATTACTTCGGTGCTTCTGAATCCTTTCAAA 5850

Lsat_WENDEL 5801 TTAAATCATTGGGTTTAGACATTACTTCGGTGCTTCTGAATCCTTTCAAA 5850

Lser_US96UC23 5801 TTAAATCATTGGGTTTAGACATTACTTCGGTGCTTCTGAATCCTTTCAAA 5850

Lser_LAC005780 5801 TTAAATCATTGGGTTTAGACATTACTTCGGTGCTTCTGAATCCTTTCAAA 5850

Lvir_CGN013357 5801 TTAAATCATTGGGTTTAGACATTACTTCGGTGCTTCTGAATCCTTTCAAA 5850

Lsal_LAC008020 5801 TTAAATCATTGGGTTTAGACATTACTTCGGTGCTTCTGAATCCTTTCAAA 5850

Lsat_Salinas 5851 ATGGTAGCAACATACCCCTTTTGTGATTTCTTTCTAGAATCATACCGACG 5900

Lsat_WENDEL 5851 ATGGTAGCAACATACCCCTTTTGTGATTTCTTTCTAGAATCATACCGACG 5900

Lser_US96UC23 5851 ATGGTAGCAACATACCCCTTTTGTGATTTCTTTCTAGAATCATACCGACG 5900

Lser_LAC005780 5851 ATGGTAGCAACATACCCCTTTTGTGATTTCTTTCTAGAATCATACCGACG 5900

Lvir_CGN013357 5851 ATGGTAGCAACATACCCCTTTTGTGATTTCTTTCTAGAATCATACCGACG 5900

Lsal_LAC008020 5851 ATGGTAGCAACATACCCCTTTTGTGATTTCTTTCTAGAATCATACCGACG 5900

Lsat_Salinas 5901 GTTGATTCGTGCGCGATACACTGTGGATCGAAAACGTTTTGCGGTTCCAA 5950

Lsat_WENDEL 5901 GTTGATTCGTGCGCGATACACTGTGGATCGAAAACGTTTTGCGGTTCCAA 5950

Lser_US96UC23 5901 GTTGATTCGTGCGCGATACACTGTGGATCGAAAACGTTTTGCGGTTCCAA 5950

Lser_LAC005780 5901 GTTGATTCGTGCGCGATACACTGTGGATCGAAAACGTTTTGCGGTTCCAA 5950

Lvir_CGN013357 5901 GTTGATTCGTGCGCGATACACTGTGGATCGAAAACGTTTTGCGGTTCCAA 5950

Lsal_LAC008020 5901 GTTGATTCGTGCGCGATACACTGTGGATCGAAAACGTTTTGCGGTTCCAA 5950

Lsat_Salinas 5951 CAATTTTTTTGAATTGAAAATTTGCTCGAATCGGATCCTTTCAATTTCTA 6000

Lsat_WENDEL 5951 CAATTTTTTTGAATTGAAAATTTGCTCGAATCGGATCCTTTCAATTTCTA 6000

Lser_US96UC23 5951 CAATTTTTTTGAATTGAAAATTTGCTCGAATCGGATCCTTTCAATTTCTA 6000

Lser_LAC005780 5951 CAATTTTTTTGAATTGAAAATTTGCTCGAATCGGATCCTTTCAATTTCTA 6000

Lvir_CGN013357 5951 CAATTTTTTTGAATTGAAAATTTGCTCGAATCGGATCCTTTCAATTTCTA 6000

Lsal_LAC008020 5951 CAATTTTTTTGAATTGAAAATTTGCTCGAATCGGATCCTTTCAATTTCTA 6000

Lsat_Salinas 6001 TATCGATATCGAAGATATACTTACGAAGTTGTTCCAATTTATTGATTGGC 6050

Lsat_WENDEL 6001 TATCGATATCGAAGATATACTTACGAAGTTGTTCCAATTTATTGATTGGC 6050

Lser_US96UC23 6001 TATCGATATCGAAGATATACTTACGAAGTTGTTCCAATTTATTGATTGGC 6050

Lser_LAC005780 6001 TATCGATATCGAAGATATACTTACGAAGTTGTTCCAATTTATTGATTGGC 6050

Lvir_CGN013357 6001 TATCGATATCGAAGATATACTTACGAAGTTGTTCCAATTTATTGATTGGC 6050

Lsal_LAC008020 6001 TATCGATATCGAAGATATACTTACGAAGTTGTTCCAATTTATTGATTGGC 6050

Lsat_Salinas 6051 ATTAACCCTAGATCGTTGCCCCTGAGAAATTAATCCATACTTTCTACTCG 6100

Lsat_WENDEL 6051 ATTAACCCTAGATCGTTGCCCCTGAGAAATTAATCCATACTTTCTACTCG 6100

Lser_US96UC23 6051 ATTAACCCTAGATCGTTGCCCCTGAGAAATTAATCCATACTTTCTACTCG 6100

Lser_LAC005780 6051 ATTAACCCTAGATCGTTGCCCCTGAGAAATTAATCCATACTTTCTACTCG 6100

Lvir_CGN013357 6051 ATTAACCCTAGATCGTTGCCCCTGAGAAATTAATCCATACTTTCTACTCG 6100

Lsal_LAC008020 6051 ATTAACCCTAGATCGTTGCCCCTGAGAAATTAATCCATACTTTCTACTCG 6100

Lsat_Salinas 6101 AGCTCCATCATGAACTATTTACATTACAACCCAATAAAAAAGAAGGGTTC 6150

Lsat_WENDEL 6101 AGCTCCATCATGAACTATTTACATTACAACCCAATAAAAAAGAAGGGTTC 6150

Lser_US96UC23 6101 AGCTCCATCATGAACTATTTACATTACAACCCAATAAAAAAGAAGGGTTC 6150

Lser_LAC005780 6101 AGCTCCATCATGAACTATTTACATTACAACCCAATAAAAAAGAAGGGTTC 6150

Lvir_CGN013357 6101 AGCTCCATCATGAACTATTTACATTACAACCCAATAAAAAAGAAGGGTTC 6150

Lsal_LAC008020 6101 AGCTCCATCATGAACTATTTACATTACAACCCAATAAAAAAGAAGGGTTC 6150

Lsat_Salinas 6151 TAGTAGAATAGAACAAACGACGTCGAGCCAAGAGCACCTTCATTCCTATA 6200

Lsat_WENDEL 6151 TAGTAGAATAGAACAAACGACGTCGAGCCAAGAGCACCTTCATTCCTATA 6200

Lser_US96UC23 6151 TAGTAGAATAGAACAAACGACGTCGAGCCAAGAGCACCTTCATTCCTATA 6200

Lser_LAC005780 6151 TAGTAGAATAGAACAAACGACGTCGAGCCAAGAGCACCTTCATTCCTATA 6200

Lvir_CGN013357 6151 TAGTAGAATAGAACAAACGACGTCGAGCCAAGAGCACCTTCATTCCTATA 6200

Lsal_LAC008020 6151 TAGTAGAATAGAACAAACGACGTCGAGCCAAGAGCACCTTCATTCCTATA 6200

Lsat_Salinas 6201 TAAAAGAAAATGGTGGATGTAAGAATAAAAATCCACACCGGATCGTGTCC 6250

Lsat_WENDEL 6201 TAAAAGAAAATGGTGGATGTAAGAATAAAAATCCACACCGGATCGTGTCC 6250

Lser_US96UC23 6201 TAAAAGAAAATGGTGGATGTAAGAATAAAAATCCACACCGGATCGTGTCC 6250

Lser_LAC005780 6201 TAAAAGAAAATGGTGGATGTAAGAATAAAAATCCACACCGGATCGTGTCC 6250

Lvir_CGN013357 6201 TAAAAGAAAATGGTGGATGTAAGAATAAAAATCCACACCGGATCGTGTCC 6250

Lsal_LAC008020 6201 TAAAAGAAAATGGTGGATGTAAGAATAAAAATCCACACCGGATCGTGTCC 6250

Lsat_Salinas 6251 TTCAAGTCGCACGTTGCTTTCTACCACATCGTTTTAAACGAAGTTTTACC 6300

Lsat_WENDEL 6251 TTCAAGTCGCACGTTGCTTTCTACCACATCGTTTTAAACGAAGTTTTACC 6300

Lser_US96UC23 6251 TTCAAGTCGCACGTTGCTTTCTACCACATCGTTTTAAACGAAGTTTTACC 6300

Lser_LAC005780 6251 TTCAAGTCGCACGTTGCTTTCTACCACATCGTTTTAAACGAAGTTTTACC 6300

Lvir_CGN013357 6251 TTCAAGTCGCACGTTGCTTTCTACCACATCGTTTTAAACGAAGTTTTACC 6300

Lsal_LAC008020 6251 TTCAAGTCGCACGTTGCTTTCTACCACATCGTTTTAAACGAAGTTTTACC 6300

Lsat_Salinas 6301 ATAACATTCCTCTAATTTGGAACCAGTATGGAATTGATTCAATTATGGAA 6350

Lsat_WENDEL 6301 ATAACATTCCTCTAATTTGGAACCAGTATGGAATTGATTCAATTATGGAA 6350

Lser_US96UC23 6301 ATAACATTCCTCTAATTTGGAACCAGTATGGAATTGATTCAATTATGGAA 6350

Lser_LAC005780 6301 ATAACATTCCTCTAATTTGGAACCAGTATGGAATTGATTCAATTATGGAA 6350

Lvir_CGN013357 6301 ATAACATTCCTCTAATTTGGAACCAGTATGGAATTGATTCAATTATGGAA 6350

Lsal_LAC008020 6301 ATAACATTCCTCTAATTTGGAACCAGTATGGAATTGATTCAATTATGGAA 6350

Lsat_Salinas 6351 TCATGAATAGTCATTGGTTCAGTCGGTACAGAGACATAGTCATTTATATT 6400

Lsat_WENDEL 6351 TCATGAATAGTCATTGGTTCAGTCGGTACAGAGACATAGTCATTTATATT 6400

Lser_US96UC23 6351 TCATGAATAGTCATTGGTTCAGTCGGTACAGAGACATAGTCATTTATATT 6400

Lser_LAC005780 6351 TCATGAATAGTCATTGGTTCAGTCGGTACAGAGACATAGTCATTTATATT 6400

Lvir_CGN013357 6351 TCATGAATAGTCATTGGTTCAGTCGGTACAGAGACATAGTCATTTATATT 6400

Lsal_LAC008020 6351 TCATGAATAGTCATTGGTTCAGTCGGTACAGAGACATAGTCATTTATATT 6400

Lsat_Salinas 6401 TTTTTTCCTAGCTACATAGATAATAAATATTTTTTATTAAGAAATCTTAG 6450

Lsat_WENDEL 6401 TTTTTTCCTAGCTACATAGATAATAAATATTTTTTATTAAGAAATCTTAG 6450

Lser_US96UC23 6401 TTTTTTCCTAGCTACATAGATAATAAATATTTTTTATTAAGAAATCTTAG 6450

Lser_LAC005780 6401 TTTTTTCCTAGCTACATAGATAATAAATATTTTTTATTAAGAAATCTTAG 6450

Lvir_CGN013357 6401 TTTTTTCCTAGCTACATAGATAATAAATATTTTTTATTAAGAAATCTTAG 6450

Lsal_LAC008020 6401 TTTTTTCCTAGCTACATAAATAATAAATATTTTTTATTAAGAAATCTTAG 6450

Lsat_Salinas 6451 CAAGACCCGGGCTTTTTTGTATGAACGGAAATTTTTTCTATAAATCTATA 6500

Lsat_WENDEL 6451 CAAGACCCGGGCTTTTTTGTATGAACGGAAATTTTTTCTATAAATCTATA 6500

Lser_US96UC23 6451 CAAGACCCGGGCTTTTTTGTATGAACGGAAATTTTTTCTATAAATCTATA 6500

Lser_LAC005780 6451 CAAGACCCGGGCTTTTTTGTATGAACGGAAATTTTTTCTATAAATCTATA 6500

Lvir_CGN013357 6451 CAAGACCCGGGCTTTTTTGTATGAACGGAAATTTTTTCTATAAATCTATA 6500

Lsal_LAC008020 6451 CAAGACCCGGGCTTTTTTGTATGAACGGAAATTTTTTCTATAAATCTAYA 6500

Lsat_Salinas 6501 TCTCAAAAAAATATCTAACAGAAATATCCAGAAATCTAAATATAGAAATA 6550

Lsat_WENDEL 6501 TCTCAAAAAAATATCTAACAGAAATATCCAGAAATCTAAATATAGAAATA 6550

Lser_US96UC23 6501 TCTCAAAAAAATATCTAACAGAAATATCCAGAAATCTAAATATAGAAATA 6550

Lser_LAC005780 6501 TCTCAAAAAAATATCTAACAGAAATATCCAGAAATCTAAATATAGAAATA 6550

Lvir_CGN013357 6501 TCTCAAAAAAATATCTAACAGAAATATCCAGAAATCTAAATATAGAAATA 6550

Lsal_LAC008020 6501 TCTCAAAAAAATATCTAACAGAAATATCCAGAAATCTAAATATAGAAATA 6550

Lsat_Salinas 6551 ACATAACTAACAGAAATAACACGAATAAATAAATTCTATTTGACTCTGCC 6600

Lsat_WENDEL 6551 ACATAACTAACAGAAATAACACGAATAAATAAATTCTATTTGACTCTGCC 6600

Lser_US96UC23 6551 ACATAACTAACAGAAATAACACGAATAAATAAATTCTATTTGACTCTGCC 6600

Lser_LAC005780 6551 ACATAACTAACAGAAATAACACGAATAAATAAATTCTATTTGACTCTGCC 6600

Lvir_CGN013357 6551 ACATAACTAACAGAAATAACACGAATAAATAAATTCTATTTGACTCTGCC 6600

Lsal_LAC008020 6551 ACATAACTAACAGAAATAACACGAATAAATAAATTCTATTTGACTCTGCC 6600

Lsat_Salinas 6601 TGTTCAAGTGACTCAATCAATAAGATAGACTGACCCATTTCCAACTTCTC 6650

Lsat_WENDEL 6601 TGTTCAAGTGACTCAATCAATAAGATAGACTGACCCATTTCCAACTTCTC 6650

Lser_US96UC23 6601 TGTTCAAGTGACTCAATCAATAAGATAGACTGACCCATTTCCAACTTCTC 6650

Lser_LAC005780 6601 TGTTCAAGTGACTCAATCAATAAGATAGACTGACCCATTTCCAACTTCTC 6650

Lvir_CGN013357 6601 TGTTCAAGTGACTCAATCAATAAGATAGACTGACCCATTTCCAACTTCTC 6650

Lsal_LAC008020 6601 TGTTCAAGTGACTCAATCAATAAGATAGACTGACCCATTTCCAACTTCTC 6650

Lsat_Salinas 6651 AAAGATTCAACCCATAAGGAATCATTACAAATCTTGATAATTGAAAAAGT 6700

Lsat_WENDEL 6651 AAAGATTCAACCCATAAGGAATCATTACAAATCTTGATAATTGAAAAAGT 6700

Lser_US96UC23 6651 AAAGATTCAACCCATAAGGAATCATTACAAATCTTGATAATTGAAAAAGT 6700

Lser_LAC005780 6651 AAAGATTCAACCCATAAGGAATCATTACAAATCTTGATAATTGAAAAAGT 6700

Lvir_CGN013357 6651 AAAGATTCAACCCATAAGGAATCATTACAAATCTTGATAATTGAAAAAGT 6700

Lsal_LAC008020 6651 AAAGATTCAACCCATAAGGAATCATTACAAATCTTGATAATTGAAAAAGT 6700

Lsat_Salinas 6701 TTCCTACTTCGACATCATTATTTGAGTCAAGTTTTACTTTTACAGTAAAG 6750

Lsat_WENDEL 6701 TTCCTACTTCGACATCATTATTTGAGTCAAGTTTTACTTTTACAGTAAAG 6750

Lser_US96UC23 6701 TTCCTACTTCGACATCATTATTTGAGTCAAGTTTTACTTTTACAGTAAAG 6750

Lser_LAC005780 6701 TTCCTACTTCGACATCATTATTTGAGTCAAGTTTTACTTTTACAGTAAAG 6750

Lvir_CGN013357 6701 TTCCTACTTCGACATCATTATTTGAGTCAAGTTTTACTTTTACAGTAAAG 6750

Lsal_LAC008020 6701 TTCCTACTTCGACATCATTATTTGAGTCAAGTTTTACTTTTACAGTAAAG 6750

Lsat_Salinas 6751 CCTACATTTGATTATCATAAGAAATAAGAATTTCTGTTTCTTTTCGAATA 6800

Lsat_WENDEL 6751 CCTACATTTGATTATCATAAGAAATAAGAATTTCTGTTTCTTTTCGAATA 6800

Lser_US96UC23 6751 CCTACATTTGATTATCATAAGAAATAAGAATTTCTGTTTCTTTTCGAATA 6800

Lser_LAC005780 6751 CCTACATTTGATTATCATAAGAAATAAGAATTTCTGTTTCTTTTCGAATA 6800

Lvir_CGN013357 6751 CCTACATTTGATTATCATAAGAAATAAGAATTTCTGTTTCTTTTCGAATA 6800

Lsal_LAC008020 6751 CCTACATTTGATTATCATAAGAAATAAGAATTTCTGTTTCTTTTCGAATA 6800

Lsat_Salinas 6801 GAATTGTCAATGATTTAAAGCAAATAAGCTAAAAAGATCGAACAATAAAA 6850

Lsat_WENDEL 6801 GAATTGTCAATGATTTAAAGCAAATAAGCTAAAAAGATCGAACAATAAAA 6850

Lser_US96UC23 6801 GAATTGTCAATGATTTAAAGCAAATAAGCTAAAAAGATCGAACAATAAAA 6850

Lser_LAC005780 6801 GAATTGTCAATGATTTAAAGCAAATAAGCTAAAAAGATCGAACAATAAAA 6850

Lvir_CGN013357 6801 GAATTGTCAATGATTTAAAGCAAATAAGCTAAATAGATCGAACAATAAAA 6850

Lsal_LAC008020 6801 GAATTGTCAATGATTTAAAGCAAATAAGCTAAATAGATCGAACAATAAAA 6850

Lsat_Salinas 6851 AGAAATATCATTGTTAAATATTTAAATTTGAATATTTTAGGGATGCAAAT 6900

Lsat_WENDEL 6851 AGAAATATCATTGTTAAATATTTAAATTTGAATATTTTAGGGATGCAAAT 6900

Lser_US96UC23 6851 AGAAATATCATTGTTAAATATTTAAATTTGAATATTTTAGGGATGCAAAT 6900

Lser_LAC005780 6851 AGAAATATCATTGTTAAATATTTAAATTTGAATATTTTAGGGATGCAAAT 6900

Lvir_CGN013357 6851 AGAAATATCATTGTTAAATATTTAAATTTGAATATTTTAGGGATGCAAAT 6900

Lsal_LAC008020 6851 AGAAATATCATTGTTAAATATTTAAATTTGAATATTTTAGGGATGCAAAT 6900

Lsat_Salinas 6901 TATTTTTCACAAAAATAGAAAGACTATTGTCACTGAAATAGGATAACAAT 6950

Lsat_WENDEL 6901 TATTTTTCACAAAAATAGAAAGACTATTGTCACTGAAATAGGATAACAAT 6950

Lser_US96UC23 6901 TATTTTTCACAAAAATAGAAAGACTATTGTCACTGAAATAGGATAACAAT 6950

Lser_LAC005780 6901 TATTTTTCACAAAAATAGAAAGACTATTGTCACTGAAATAGGATAACAAT 6950

Lvir_CGN013357 6901 TCTTTTTCACAAAAATAGAAAGACTATTGTCACTGAAATAGGATAACAAT 6950

Lsal_LAC008020 6901 TCTTTTTCACAAAAATAGAAAGACTATTGTCACTGAAATAGGATAACAAT 6950

Lsat_Salinas 6951 ACATACCCATAGGCTAAGCACTTCCTCGTTTTATATGTATTTTCATATTT 7000

Lsat_WENDEL 6951 ACATACCCATAGGCTAAGCACTTCCTCGTTTTATATGTATTTTCATATTT 7000

Lser_US96UC23 6951 ACATACCCATAGGCTAAGCACTTCCTCGTTTTATATGTATTTTCATATTT 7000

Lser_LAC005780 6951 ACATACCCATAGGCTAAGCACTTCCTCGTTTTATATGTATTTTCATATTT 7000

Lvir_CGN013357 6951 ACATACCCATAGGCTAAGCACTTCCTCGTTTTATATGTATTTTCATATTT 7000

Lsal_LAC008020 6951 ACATACCCATAGGCTAAGCACTTCCTCATTTTATATGTATTTTCATATTT 7000

Lsat_Salinas 7001 TGTTTAACCTGAGGTTAAGTAATCTTTCTGCAACTGTGATCTATGCCCCA 7050

Lsat_WENDEL 7001 TGTTTAACCTGAGGTTAAGTAATCTTTCTGCAACTGTGATCTATGCCCCA 7050

Lser_US96UC23 7001 TGTTTAACCTGAGGTTAAGTAATCTTTCTGCAACTGTGATCTATGCCCCA 7050

Lser_LAC005780 7001 TGTTTAACCTGAGGTTAAGTAATCTTTCTGCAACTGTGATCTATGCCCCA 7050

Lvir_CGN013357 7001 TGTTTAACCTGAGGTTAAGTAATCTTTCTGCAACTGTGATCTATGCCCCA 7050

Lsal_LAC008020 7001 TGTTTAACCTGAGGTTAAGTAATCTTTCTGCAACTGTGATCTATGCCCCA 7050

Lsat_Salinas 7051 TCTTATCTTTATCTGGATCACAAAAGGAGTCAGTTACATTTGCATGTCAT 7100

Lsat_WENDEL 7051 TCTTATCTTTATCTGGATCACAAAAGGAGTCAGTTACATTTGCATGTCAT 7100

Lser_US96UC23 7051 TCTTATCTTTATCTGGATCACAAAAGGAGTCAGTTACATTTGCATGTCAT 7100

Lser_LAC005780 7051 TCTTATCTTTATCTGGATCACAAAAGGAGTCAGTTACATTTGCATGTCAT 7100

Lvir_CGN013357 7051 TCTTATCTTTATCTGGATCACAAAAGGAGTCAGTTACATTTGCATGTCAT 7100

Lsal_LAC008020 7051 TCTTATCTTTATCTGGATCACAAAAGGAGTCAGTTACATTTGCATGTCAT 7100

Lsat_Salinas 7101 TTGAACTCGATTTAGTTCATTTTGTGAAAAACTGAGATATGATTCCGAAA 7150

Lsat_WENDEL 7101 TTGAACTCGATTTAGTTCATTTTGTGAAAAACTGAGATATGATTCCGAAA 7150

Lser_US96UC23 7101 TTGAACTCGATTTAGTTCATTTTGTGAAAAACTGAGATATGATTCCGAAA 7150

Lser_LAC005780 7101 TTGAACTCGATTTAGTTCATTTTGTGAAAAACTGAGATATGATTCCGAAA 7150

Lvir_CGN013357 7101 TTGAACTCGATTTAGTTCATTTTGTGAAAAACTGAGATATGATTCCGAAA 7150

Lsal_LAC008020 7101 TTGAACTCGATTTAGTTCATTTTGTGAAAAACTGAGATATGATTCCGAAA 7150

Lsat_Salinas 7151 AGAATCATTAAAAATCAAAAAAGAAAGAAGAATAAGATTCTATCCAATAT 7200

Lsat_WENDEL 7151 AGAATCATTAAAAATCAAAAAAGAAAGAAGAATAAGATTCTATCCAATAT 7200

Lser_US96UC23 7151 AGAATCATTAAAAATCAAAAAAGAAAGAAGAATAAGATTCTATCCAATAT 7200

Lser_LAC005780 7151 AGAATCATTAAAAATCAAAAAAGAAAGAAGAATAAGATTCTATCCAATAT 7200

Lvir_CGN013357 7151 AGAATCATTAAAAATCAAAAAAGAAAGAAGAATAAGATTCTATCCAATAT 7200

Lsal_LAC008020 7151 AGAATCATTAAAAATCAAAAAAGAAAGAAGAATAAGATTCTATCCAATAT 7200

Lsat_Salinas 7201 TAGACCTTGAAACAGAACCTTGTTGAACAAAAAAGATGTATTCGGATACA 7250

Lsat_WENDEL 7201 TAGACCTTGAAACAGAACCTTGTTGAACAAAAAAGATGTATTCGGATACA 7250

Lser_US96UC23 7201 TAGACCTTGAAACAGAACCTTGTTGAACAAAAAAGATGTATTCGGATACA 7250

Lser_LAC005780 7201 TAGACCTTGAAACAGAACCTTGTTGAACAAAAAAGATGTATTCGGATACA 7250

Lvir_CGN013357 7201 TAGACCTTGAAACAGAACCTTGTTGAACAAAAAAGATGTATTCGGATACA 7250

Lsal_LAC008020 7201 TAGACCTTGAAACAGAACCTTGTTGAACAAAAAAGATGTATTCGGATACA 7250

Lsat_Salinas 7251 ATGGATATGCTCTGGGACGGAAGGATTCGAACCTCCGAATAGCGGGACCA 7300

Lsat_WENDEL 7251 ATGGATATGCTCTGGGACGGAAGGATTCGAACCTCCGAATAGCGGGACCA 7300

Lser_US96UC23 7251 ATGGATATGCTCTGGGACGGAAGGATTCGAACCTCCGAATAGCGGGACCA 7300

Lser_LAC005780 7251 ATGGATATGCTCTGGGACGGAAGGATTCGAACCTCCGAATAGCGGGACCA 7300

Lvir_CGN013357 7251 ATGGATATGCTCTGGGACGGAAGGATTCGAACCTCCGAATAGCGGGACCA 7300

Lsal_LAC008020 7251 ATGGATATGCTCTGGGACGGAAGGATTCGAACCTCCGAATAGCGGGACCA 7300

Lsat_Salinas 7301 AAACCCGTTGCCTTACCACTTGGCTACGCCCCATTTATATTGTTATTGGA 7350

Lsat_WENDEL 7301 AAACCCGTTGCCTTACCACTTGGCTACGCCCCATTTATATTGTTATTGGA 7350

Lser_US96UC23 7301 AAACCCGTTGCCTTACCACTTGGCTACGCCCCATTTATATTGTTATTGGA 7350

Lser_LAC005780 7301 AAACCCGTTGCCTTACCACTTGGCTACGCCCCATTTATATTGTTATTGGA 7350

Lvir_CGN013357 7301 AAACCCGTTGCCTTACCACTTGGCTACGCCCCATTTATATTGTTATTGGA 7350

Lsal_LAC008020 7301 AAACCCGTTGCCTTACCACTTGGCTACGCCCCATTTATATTGTTATTGGA 7350

Lsat_Salinas 7351 CACTAATACTAATAAAGAATAATATTGGTATTAAGTGTTCGTCAATTCCA 7400

Lsat_WENDEL 7351 CACTAATACTAATAAAGAATAATATTGGTATTAAGTGTTCGTCAATTCCA 7400

Lser_US96UC23 7351 CACTAATACTAATAAAGAATAATATTGGTATTAAGTGTTCGTCAATTCCA 7400

Lser_LAC005780 7351 CACTAATACTAATAAAGAATAATATTGGTATTAAGTGTTCGTCAATTCCA 7400

Lvir_CGN013357 7351 CACTAATACTAATAAAGAATAATATTGGTATTAAGTGTTCGTCAATTCCA 7400

Lsal_LAC008020 7351 CACTAATACTAATAAAGAATAATATTGGTATTAAGTGTTCGTCAATTCCA 7400

Lsat_Salinas 7401 GCCCAACTATCTATAGAAAATCTTTCTTCTTTATTCTTCTTTATAGAATA 7450

Lsat_WENDEL 7401 GCCCAACTATCTATAGAAAATCTTTCTTCTTTATTCTTCTTTATAGAATA 7450

Lser_US96UC23 7401 GCCCAACTATCTATAGAAAATCTTTCTTCTTTATTCTTCTTTATAGAATA 7450

Lser_LAC005780 7401 GCCCAACTATCTATAGAAAATCTTTCTTCTTTATTCTTCTTTATAGAATA 7450

Lvir_CGN013357 7401 GCCCAACTATCTATAGAAAATCTTTCTTCTTTATTCTTCTTTATAGAATA 7450

Lsal_LAC008020 7401 GCCCAACTATCTATAGAAAATCTTTCTTCTTTATTCTTCTTTATAGAATA 7450

Lsat_Salinas 7451 TATTATAGATTCGTGCTAGGATTTTGACATGTGTATATCTAGAATTCAAC 7500

Lsat_WENDEL 7451 TATTATAGATTCGTGCTAGGATTTTGACATGTGTATATCTAGAATTCAAC 7500

Lser_US96UC23 7451 TATTATAGATTCGTGCTAGGATTTTGACATGTGTATATCTAGAATTCAAC 7500

Lser_LAC005780 7451 TATTATAGATTCGTGCTAGGATTTTGACATGTGTATATCTAGAATTCAAC 7500

Lvir_CGN013357 7451 TATTATAGATTCGTGCTAGGATTTTGACATGTGTATATCTAGAATTCAAC 7500

Lsal_LAC008020 7451 TATTATAGATTCGTGCTAGGATTTTGACATGTGTATATCTAGAATTCAAC 7500

Lsat_Salinas 7501 TGAATTTATTGATCATTACATACAATTCAATTAAGATATTGTATGAAAGT 7550

Lsat_WENDEL 7501 TGAATTTATTGATCATTACATACAATTCAATTAAGATATTGTATGAAAGT 7550

Lser_US96UC23 7501 TGAATTTATTGATCATTACATACAATTCAATTAAGATATTGTATGAAAGT 7550

Lser_LAC005780 7501 TGAATTTATTGATCATTACATACAATTCAATTAAGATATTGTATGAAAGT 7550

Lvir_CGN013357 7501 TGAATTTATTGATCATTACATACAATTCAATTAAGATATTGTATGAAAGT 7550

Lsal_LAC008020 7501 TGAATTTATTGATCATTACATACAATTCAATTAAGATATTGTATGAAAGT 7550

Lsat_Salinas 7551 ATGATTTCTTCTATTCTCCTTTGAGAATTGGAGGATTTTTGATTGAGCGG 7600

Lsat_WENDEL 7551 ATGATTTCTTCTATTCTCCTTTGAGAATTGGAGGATTTTTGATTGAGCGG 7600

Lser_US96UC23 7551 ATGATTTCTTCTATTCTCCTTTGAGAATTGGAGGATTTTTGATTGAGCGG 7600

Lser_LAC005780 7551 ATGATTTCTTCTATTCTCCTTTGAGAATTGGAGGATTTTTGATTGAGCGG 7600

Lvir_CGN013357 7551 ATGATTTCTTCTATTCTCCTTTGAGAATTGGAGGATTTTTGATTGAGCGG 7600

Lsal_LAC008020 7551 ATGATTTCTTCTATTCTCCTTTGAGAATTGGAGGATTTTTGATTGAGCGG 7600

Lsat_Salinas 7601 AAAAAGAAGGAGTTTTTTGTCTACCTTACTTTCTTCATTTTTCCTTATAT 7650

Lsat_WENDEL 7601 AAAAAGAAGGAGTTTTTTGTCTACCTTACTTTCTTCATTTTTCCTTATAT 7650

Lser_US96UC23 7601 AAAAAGAAGGAGTTTTTTGTCTACCTTACTTTCTTCATTTTTCCTTATAT 7650

Lser_LAC005780 7601 AAAAAGAAGGAGTTTTTTGTCTACCTTACTTTCTTCATTTTTCCTTATAT 7650

Lvir_CGN013357 7601 AAAAAGAAGGAGTTTTTTGTCTACCTTACTTTCTTCATTTTTCCTTATAT 7650

Lsal_LAC008020 7601 AAAAAGAAGGAGTTTTTTGTCTACCTTACTTTCTTCATTTTTCCTTATAT 7650

Lsat_Salinas 7651 AAATAACTCAATCAAAATGCAATTATCTCGACGAACAAAATGTCTGTTAT 7700

Lsat_WENDEL 7651 AAATAACTCAATCAAAATGCAATTATCTCGACGAACAAAATGTCTGTTAT 7700

Lser_US96UC23 7651 AAATAACTCAATCAAAATGCAATTATCTCGACGAACAAAATGTCTGTTAT 7700

Lser_LAC005780 7651 AAATAACTCAATCAAAATGCAATTATCTCGACGAACAAAATGTCTGTTAT 7700

Lvir_CGN013357 7651 AAATAACTCAATCAAAATGCAATTATCTCGACGAACAAAATGTCTGTTAT 7700

Lsal_LAC008020 7651 AAATAACTCAATCAAAATGCAATTATCTCGACGAACAAAATGTCTGTTAT 7700

Lsat_Salinas 7701 GCTTAATATCTTTAGTTTGATCTGTCTTAATTCTGCCCTTTATCCGAGTA 7750

Lsat_WENDEL 7701 GCTTAATATCTTTAGTTTGATCTGTCTTAATTCTGCCCTTTATCCGAGTA 7750

Lser_US96UC23 7701 GCTTAATATCTTTAGTTTGATCTGTCTTAATTCTGCCCTTTATCCGAGTA 7750

Lser_LAC005780 7701 GCTTAATATCTTTAGTTTGATCTGTCTTAATTCTGCCCTTTATCCGAGTA 7750

Lvir_CGN013357 7701 GCTTAATATCTTTAGTTTGATCTGTCTTAATTCTGCCCTTTATCCGAGTA 7750

Lsal_LAC008020 7701 GCTTAATATCTTTAGTTTGATCTGTCTTAATTCTGCCCTTTATCCGAGTA 7750

Lsat_Salinas 7751 GTCTTTTCTTCGCCAAATTGCCCGAAGCCTATGCTTTTTTGAATCCAATC 7800

Lsat_WENDEL 7751 GTCTTTTCTTCGCCAAATTGCCCGAAGCCTATGCTTTTTTGAATCCAATC 7800

Lser_US96UC23 7751 GTCTTTTCTTCGCCAAATTGCCCGAAGCCTATGCTTTTTTGAATCCAATC 7800

Lser_LAC005780 7751 GTCTTTTCTTCGCCAAATTGCCCGAAGCCTATGCTTTTTTGAATCCAATC 7800

Lvir_CGN013357 7751 GTCTTTTCTTCGCCAAATTGCCCGAAGCCTATGCTTTTTTGAATCCAATC 7800

Lsal_LAC008020 7751 GTCTTTTCTTCGCCAAATTGCCCGAAGCCTATGCTTTTTTGAATCCAATC 7800

Lsat_Salinas 7801 GTAGATGTTATGCCAGTCATACCCCTGTTCTTTTTTCTTTTAGCCTTTGT 7850

Lsat_WENDEL 7801 GTAGATGTTATGCCAGTCATACCCCTGTTCTTTTTTCTTTTAGCCTTTGT 7850

Lser_US96UC23 7801 GTAGATGTTATGCCAGTCATACCCCTGTTCTTTTTTCTTTTAGCCTTTGT 7850

Lser_LAC005780 7801 GTAGATGTTATGCCAGTCATACCCCTGTTCTTTTTTCTTTTAGCCTTTGT 7850

Lvir_CGN013357 7801 GTAGATGTTATGCCAGTCATACCCCTGTTCTTTTTTCTTTTAGCCTTTGT 7850

Lsal_LAC008020 7801 GTAGATGTTATGCCAGTCATACCCCTGTTCTTTTTTCTTTTAGCCTTTGT 7850

Lsat_Salinas 7851 TTGGCAAGCTGCTGTAAGTTTTCGATAAGATCTTGAATACTATCCTAGAA 7900

Lsat_WENDEL 7851 TTGGCAAGCTGCTGTAAGTTTTCGATAAGATCTTGAATACTATCCTAGAA 7900

Lser_US96UC23 7851 TTGGCAAGCTGCTGTAAGTTTTCGATAAGATCTTGAATACTATCCTAGAA 7900

Lser_LAC005780 7851 TTGGCAAGCTGCTGTAAGTTTTCGATAAGATCTTGAATACTATCCTAGAA 7900

Lvir_CGN013357 7851 TTGGCAAGCTGCTGTAAGTTTTCGATAAGATCTTGAATACTATCCTAGAA 7900

Lsal_LAC008020 7851 TTGGCAAGCTGCTGTAAGTTTTCGATAAGATCTTGAATACTATCCTAGAA 7900

Lsat_Salinas 7901 AATTCATGATTTATTCGAGAAAAATTATAACAATTGATAAGATCAAATAA 7950

Lsat_WENDEL 7901 AATTCATGATTTATTCGAGAAAAATTATAACAATTGATAAGATCAAATAA 7950

Lser_US96UC23 7901 AATTCATGATTTATTCGAGAAAAATTATAACAATTGATAAGATCAAATAA 7950

Lser_LAC005780 7901 AATTCATGATTTATTCGAGAAAAATTATAACAATTGATAAGATCAAATAA 7950

Lvir_CGN013357 7901 AATTCATGATTTATTCGAGAAAAATTATAACAATTGATAAGATCAAATAA 7950

Lsal_LAC008020 7901 AATTCATGATTTATTCGATAAAAATTATAACAATTGATAAGATCAAATAA 7950

Lsat_Salinas 7951 GTCTGGGAGTATGAACCTTCAATTCAAACATTGAAATTCTTGGCTAGCCG 8000

Lsat_WENDEL 7951 GTCTGGGAGTATGAACCTTCAATTCAAACATTGAAATTCTTGGCTAGCCG 8000

Lser_US96UC23 7951 GTCTGGGAGTATGAACCTTCAATTCAAACATTGAAATTCTTGGCTAGCCG 8000

Lser_LAC005780 7951 GTCTGGGAGTATGAACCTTCAATTCAAACATTGAAATTCTTGGCTAGCCG 8000

Lvir_CGN013357 7951 GTCTGGGAGTATGAACCTTCAATTCAAACATTGAAATTCTTGGCTAGCCG 8000

Lsal_LAC008020 7951 GTCTGGGAGTATGAACCTTCAATTCAAACATTGAAATTCTTGGCTAGCCG 8000

Lsat_Salinas 8001 CAATAAATTTGGCTCGCCCCATTTCCCGATTCTAATCCTTTTTCCGGCGA 8050

Lsat_WENDEL 8001 CAATAAATTTGGCTCGCCCCATTTCCCGATTCTAATCCTTTTTCCGGCGA 8050

Lser_US96UC23 8001 CAATAAATTTGGCTCGCCCCATTTCCCGATTCTAATCCTTTTTCCGGCGA 8050

Lser_LAC005780 8001 CAATAAATTTGGCTCGCCCCATTTCCCGATTCTAATCCTTTTTCCGGCGA 8050

Lvir_CGN013357 8001 CAATAAATTTGGCTCGCCCCATTTCCCGATTCTAATCCTTTTTCCGGCGA 8050

Lsal_LAC008020 8001 CAATAAATTTGGCTCGCCCCATTTCCCGATTCTAATCCTTTTTCCGGCGA 8050

Lsat_Salinas 8051 AAGACCTTATTAGGTTAGGGTCCCTTAGAATATCTAATTGTGGGTATGAA 8100

Lsat_WENDEL 8051 AAGACCTTATTAGGTTAGGGTCCCTTAGAATATCTAATTGTGGGTATGAA 8100

Lser_US96UC23 8051 AAGACCTTATTAGGTTAGGGTCCCTTAGAATATCTAATTGTGGGTATGAA 8100

Lser_LAC005780 8051 AAGACCTTATTAGGTTAGGGTCCCTTAGAATATCTAATTGTGGGTATGAA 8100

Lvir_CGN013357 8051 AAGACCTTATTAGGTTAGGGTCCCTTAGAATATCTAATTGTGGGTATGAA 8100

Lsal_LAC008020 8051 AAGACCTTATTAGGTTAGGGTCCCTTAGAATATCTAATTGTGGGTATGAA 8100

Lsat_Salinas 8101 AGTGATTTGTGATAATCAAAGACTCTTATTACAAATTTAAACTTCAGTTG 8150

Lsat_WENDEL 8101 AGTGATTTGTGATAATCAAAGACTCTTATTACAAATTTAAACTTCAGTTG 8150

Lser_US96UC23 8101 AGTGATTTGTGATAATCAAAGACTCTTATTACAAATTTAAACTTCAGTTG 8150

Lser_LAC005780 8101 AGTGATTTGTGATAATCAAAGACTCTTATTACAAATTTAAACTTCAGTTG 8150

Lvir_CGN013357 8101 AATGATTTGTGATAATCAAAGACTCTTATTACAAATTTAAACTTCAGTTG 8150

Lsal_LAC008020 8101 AGTGATTTGTGATAATCAAAGACTCTTATTACAAATTTAAACTTCAGTTG 8150

Lsat_Salinas 8151 AATTTCTGAACATTCTTTTCTATTTCTAGAATTCATTTCTTGGTGTCAAA 8200

Lsat_WENDEL 8151 AATTTCTGAACATTCTTTTCTATTTCTAGAATTCATTTCTTGGTGTCAAA 8200

Lser_US96UC23 8151 AATTTCTGAACATTCTTTTCTATTTCTAGAATTCATTTCTTGGTGTCAAA 8200

Lser_LAC005780 8151 AATTTCTGAACATTCTTTTCTATTTCTAGAATTCATTTCTTGGTGTCAAA 8200

Lvir_CGN013357 8151 AATTTCTGAACATTCTTTTCTATTTCTAGAATTCATTTCTTGGTGTCAAA 8200

Lsal_LAC008020 8151 AATTTCTGAACATTCTTTTCTATTTCTAGAATTCATTTCTTGGTGTCAAA 8200

Lsat_Salinas 8201 ATAGGATATGTGATATAAAAATGGAGGATCTATTATCTTTTCTCGTTTTT 8250

Lsat_WENDEL 8201 ATAGGATATGTGATATAAAAATGGAGGATCTATTATCTTTTCTCGTTTTT 8250

Lser_US96UC23 8201 ATAGGATATGTGATATAAAAATGGAGGATCTATTATCTTTTCTCGTTTTT 8250

Lser_LAC005780 8201 ATAGGATATGTGATATAAAAATGGAGGATCTATTATCTTTTCTCGTTTTT 8250

Lvir_CGN013357 8201 ATAGGATATGTGATATAAAAATGGAGGATCTATTATCTTTTCTCGTTTTT 8250

Lsal_LAC008020 8201 ATAGGATATGTGATATAAAAATGAAGGATCTATTATCTTTTCTCGTTTTT 8250

Lsat_Salinas 8251 CTTTTTCAAAAAATGATCTTGGAGGTTGTGTAATGCTTACTCTCAAACTT 8300

Lsat_WENDEL 8251 CTTTTTCAAAAAATGATCTTGGAGGTTGTGTAATGCTTACTCTCAAACTT 8300

Lser_US96UC23 8251 CTTTTTCAAAAAATGATCTTGGAGGTTGTGTAATGCTTACTCTCAAACTT 8300

Lser_LAC005780 8251 CTTTTTCAAAAAATGATCTTGGAGGTTGTGTAATGCTTACTCTCAAACTT 8300

Lvir_CGN013357 8251 CTTTTTCAAAAAATGATCTTGGAGGTTGTGTAATGCTTACTCTCAAACTT 8300

Lsal_LAC008020 8251 CTTTTTCAAAAAATGATCTTGGAGGTTGTGTAATGCTTACTCTCAAACTT 8300

Lsat_Salinas 8301 TTCGTTTACACAGTAGTAATATTCTTTGTTTCTCTCTTCATCTTTGGATT 8350

Lsat_WENDEL 8301 TTCGTTTACACAGTAGTAATATTCTTTGTTTCTCTCTTCATCTTTGGATT 8350

Lser_US96UC23 8301 TTCGTTTACACAGTAGTAATATTCTTTGTTTCTCTCTTCATCTTTGGATT 8350

Lser_LAC005780 8301 TTCGTTTACACAGTAGTAATATTCTTTGTTTCTCTCTTCATCTTTGGATT 8350

Lvir_CGN013357 8301 TTTGTTTACACAGTAGTAATATTCTTTGTTTCTCTCTTCATCTTTGGATT 8350

Lsal_LAC008020 8301 TTCGTTTACACAGTAGTAATATTCTTTGTTTCTCTCTTCATCTTTGGATT 8350

Lsat_Salinas 8351 CCTATCCAATGATCCAGGACGTAATCCTGGACGTGAAGAATAAAATAAAA 8400

Lsat_WENDEL 8351 CCTATCCAATGATCCAGGACGTAATCCTGGACGTGAAGAATAAAATAAAA 8400

Lser_US96UC23 8351 CCTATCCAATGATCCAGGACGTAATCCTGGACGTGAAGAATAAAATAAAA 8400

Lser_LAC005780 8351 CCTATCCAATGATCCAGGACGTAATCCTGGACGTGAAGAATAAAATAAAA 8400

Lvir_CGN013357 8351 CCTATCCAATGATCCAGGACGTAATCCTGGACGTGAAGAATAAAATAAAA 8400

Lsal_LAC008020 8351 CCTATCCAATGATCCAGGACGTAATCCTGGACGTGAAGAATAAAATAAAA 8400

Lsat_Salinas 8401 ATTTCGTTTTTCTTGCTTGATTTACAATTTTCTTAAGATTTTATTTTATA 8450

Lsat_WENDEL 8401 ATTTCGTTTTTCTTGCTTGATTTACAATTTTCTTAAGATTTTATTTTATA 8450

Lser_US96UC23 8401 ATTTCGTTTTTCTTGCTTGATTTACAATTTTCTTAAGATTTTATTTTATA 8450

Lser_LAC005780 8401 ATTTCGTTTTTCTTGCTTGATTTACAATTTTCTTAAGATTTTATTTTATA 8450

Lvir_CGN013357 8401 ATTTCGTTTTTCTTGCTTGATTTACAATTTTCTTAAGATTTTATTTTATA 8450

Lsal_LAC008020 8401 ATTTCGTTTTTCTTGCTTGATTTACAATTTTCTTAAGATTTTATTTTATA 8450

Lsat_Salinas 8451 TATTCATATTCCATACGTTTAACTAGTAAAAAAATCATTTAATTTAAAAG 8500

Lsat_WENDEL 8451 TATTCATATTCCATACGTTTAACTAGTAAAAAAATCATTTAATTTAAAAG 8500

Lser_US96UC23 8451 TATTCATATTCCATACGTTTAACTAGTAAAAAAATCATTTAATTTAAAAG 8500

Lser_LAC005780 8451 TATTCATATTCCATACGTTTAACTAGTAAAAAAATCATTTAATTTAAAAG 8500

Lvir_CGN013357 8451 TATTCATATTCCATACGTTTAACTAGTAAAAAAATCATTTAATTTAAAAG 8500

Lsal_LAC008020 8451 TATTCATATTCCATACGTTTAACTAGTAAAAAAATCATTTAATTTAAAAG 8500

Lsat_Salinas 8501 GGGTTTGCGAAATTTGAAAGAAAAAAAATAGAAAGTCATCAACGGAAACG 8550

Lsat_WENDEL 8501 GGGTTTGCGAAATTTGAAAGAAAAAAAATAGAAAGTCATCAACGGAAACG 8550

Lser_US96UC23 8501 GGGTTTGCGAAATTTGAAAGAAAAAAAATAGAAAGTCATCAACGGAAACG 8550

Lser_LAC005780 8501 GGGTTTGCGAAATTTGAAAGAAAAAAAATAGAAAGTCATCAACGGAAACG 8550

Lvir_CGN013357 8501 GGGTTTGCGAAATTTGAAAGAAAAAAAATAGAAAGTCATCAACGGAAACG 8550

Lsal_LAC008020 8501 GGGTTTGCGAAATTTGAAAGAAAAAAA-TAGAAAGTCATCAACGGAAACG 8549

Lsat_Salinas 8551 GAAAGAGAGGGATTCGAACCCTCGGTACGAATAACTCGTACAACGGATTA 8600

Lsat_WENDEL 8551 GAAAGAGAGGGATTCGAACCCTCGGTACGAATAACTCGTACAACGGATTA 8600

Lser_US96UC23 8551 GAAAGAGAGGGATTCGAACCCTCGGTACGAATAACTCGTACAACGGATTA 8600

Lser_LAC005780 8551 GAAAGAGAGGGATTCGAACCCTCGGTACGAATAACTCGTACAACGGATTA 8600

Lvir_CGN013357 8551 GAAAGAGAGGGATTCGAACCCTCGGTACGAATAACTCGTACAACGGATTA 8600

Lsal_LAC008020 8550 GAAAGAGAGGGATTCGAACCCTCGGTACGAATAACTCGTACAACGGATTA 8599

Lsat_Salinas 8601 GCAATCCGTCGCTTTCGTCCACTCAGCCATCTCTCCCAATTGAAAAAGAT 8650

Lsat_WENDEL 8601 GCAATCCGTCGCTTTCGTCCACTCAGCCATCTCTCCCAATTGAAAAAGAT 8650

Lser_US96UC23 8601 GCAATCCGTCGCTTTCGTCCACTCAGCCATCTCTCCCAATTGAAAAAGAT 8650

Lser_LAC005780 8601 GCAATCCGTCGCTTTCGTCCACTCAGCCATCTCTCCCAATTGAAAAAGAT 8650

Lvir_CGN013357 8601 GCAATCCGCCGCTTTCGTCCACTCAGCCATCTCTCCCAATTGAAAAAGAT 8650

Lsal_LAC008020 8600 GCAATCCGCCGCTTTCGTCCACTCAGCCATCTCTCCCAATTGAAAAAGAT 8649

Lsat_Salinas 8651 AATTACTATGTTACATTACACATCAAGTAAGGATTAACGAAAGTATTTCT 8700

Lsat_WENDEL 8651 AATTACTATGTTACATTACACATCAAGTAAGGATTAACGAAAGTATTTCT 8700

Lser_US96UC23 8651 AATTACTATGTTACATTACACATCAAGTAAGGATTAACGAAAGTATTTCT 8700

Lser_LAC005780 8651 AATTACTATGTTACATTACACATCAAGTAAGGATTAACGAAAGTATTTCT 8700

Lvir_CGN013357 8651 AATTACTATGTTACATTACACATCAAGTAAGGATTAACGAAAGTATTTCT 8700

Lsal_LAC008020 8650 AATTACTATGTTACATTACACATCAAGTAAGGATTAACGAAAGTATTTCT 8699

Lsat_Salinas 8701 TTCACATTCTTTATCGTTATTATATAATTAGCAATTCCATTTAGATGCTC 8750

Lsat_WENDEL 8701 TTCACATTCTTTATCGTTATTATATAATTAGCAATTCCATTTAGATGCTC 8750

Lser_US96UC23 8701 TTCACATTCTTTATCGTTATTATATAATTAGCAATTCCATTTAGATGCTC 8750

Lser_LAC005780 8701 TTCACATTCTTTATCGTTATTATATAATTAGCAATTCCATTTAGATGCTC 8750

Lvir_CGN013357 8701 TTCACATTCTTTATCGTTATTATATAATTAGCAATTCCATTTAGATGCTC 8750

Lsal_LAC008020 8700 TTCACATTCTTTATCGTTATTATATAATTAGCAATTCCATTTAGATGCTC 8749

Lsat_Salinas 8751 GAAAGATCCAAATAGAAAGATAAATAAAGAAGACCTTCTTGCTTTTATTT 8800

Lsat_WENDEL 8751 GAAAGATCCAAATAGAAAGATAAATAAAGAAGACCTTCTTGCTTTTATTT 8800

Lser_US96UC23 8751 GAAAGATCCAAATAGAAAGATAAATAAAGAAGACCTTCTTGCTTTTATTT 8800

Lser_LAC005780 8751 GAAAGATCCAAATAGAAAGATAAATAAAGAAGACCTTCTTGCTTTTATTT 8800

Lvir_CGN013357 8751 GAAAGATCCAAATAGAAAGATAAATAAAGAAGACCTTCTTGCTTTTATTT 8800

Lsal_LAC008020 8750 GAAAGATCCAAATAGAAAGATAAATAAAGAAGACCTTCTTGCTTTTATTT 8799

Lsat_Salinas 8801 TGTTCGAAGTGCCTTTTGGGCCTGGCCCGGTCAATACCCAGCCGGGCCTT 8850

Lsat_WENDEL 8801 TGTTCGAAGTGCCTTTTGGGCCTGGCCCGGTCAATACCCAGCCGGGCCTT 8850

Lser_US96UC23 8801 TGTTCGAAGTGCCTTTTGGGCCTGGCCCGGTCAATACCCAGCCGGGCCTT 8850

Lser_LAC005780 8801 TGTTCGAAGTGCCTTTTGGGCCTGGCCCGGTCAATACCCAGCCGGGCCTT 8850

Lvir_CGN013357 8801 TGTTCGAAGTGCCTTTTGGGCCTGGCCCGGTCAATACCCAGCCGGGCCTT 8850

Lsal_LAC008020 8800 TGTTCGAAGTGCCTTTTGGGCCTGGCCCGGTCAATACCCAGCCGGGCCTT 8849

Lsat_Salinas 8851 TTTTTGTTCCAACGAATTCCATATATTTATAGGTATAGGAAACATACTCT 8900

Lsat_WENDEL 8851 TTTTTGTTCCAACGAATTCCATATATTTATAGGTATAGGAAACATACTCT 8900

Lser_US96UC23 8851 TTTTTGTTCCAACGAATTCCATATATTTATAGGTATAGGAAACATACTCT 8900

Lser_LAC005780 8851 TTTTTGTTCCAACGAATTCCATATATTTATAGGTATAGGAAACATACTCT 8900

Lvir_CGN013357 8851 TTTTTGTTCCAACGAATTCCATATATTTATAGGTATAGGAAACATACTCT 8900

Lsal_LAC008020 8850 TTTTTGTTCCAACGAATTCCATATATTTATAGGTATAGGAAACATACTCT 8899

Lsat_Salinas 8901 AAAAAAGATACCCAATTTGGTATCTGTGTAAGAATTTCCATTGTGGGGTT 8950

Lsat_WENDEL 8901 AAAAAAGATACCCAATTTGGTATCTGTGTAAGAATTTCCATTGTGGGGTT 8950

Lser_US96UC23 8901 AAAAAAGATACCCAATTTGGTATCTGTGTAAGAATTTCCATTGTGGGGTT 8950

Lser_LAC005780 8901 AAAAAAGATACCCAATTTGGTATCTGTGTAAGAATTTCCATTGTGGGGTT 8950

Lvir_CGN013357 8901 AAAAAAGATACCCAATTTGGTATCTGTGTAAGAATTTCCATTGTGGGGTT 8950

Lsal_LAC008020 8900 AAAAAAGATACCCAATTTGGTATCTGTGTAAGAATTTCCATTGTGGGGTT 8949

Lsat_Salinas 8951 TACATATACTTATCGTTTTTGTTATAATGGAAATTGAAAGGATTAAGAAT 9000

Lsat_WENDEL 8951 TACATATACTTATCGTTTTTGTTATAATGGAAATTGAAAGGATTAAGAAT 9000

Lser_US96UC23 8951 TACATATACTTATCGTTTTTGTTATAATGGAAATTGAAAGGATTAAGAAT 9000

Lser_LAC005780 8951 TACATATACTTATCGTTTTTGTTATAATGGAAATTGAAAGGATTAAGAAT 9000

Lvir_CGN013357 8951 TACATATACTTATCGTTTTTGTTATAATGGAAATTGAAAGGATTAAGAAT 9000

Lsal_LAC008020 8950 TACATATACTTATCGTTTTTGTTATAATGGAAATTGAAAGGATTAAGAAT 8999

Lsat_Salinas 9001 CAATTAAGTATGTTTTGTAGTTTCTTATGTCATTAGGCAACCCCAATTTT 9050

Lsat_WENDEL 9001 CAATTAAGTATGTTTTGTAGTTTCTTATGTCATTAGGCAACCCCAATTTT 9050

Lser_US96UC23 9001 CAATTAAGTATGTTTTGTAGTTTCTTATGTCATTAGGCAACCCCAATTTT 9050

Lser_LAC005780 9001 CAATTAAGTATGTTTTGTAGTTTCTTATGTCATTAGGCAACCCCAATTTT 9050

Lvir_CGN013357 9001 CAATTAAGTATGTTTTGTAGTTTCTTATGTCATTAGGCAACCCCAATTTT 9050

Lsal_LAC008020 9000 CAATTAAGTATGTTTTGTAGTTTCTTATGTCATTAGGCAACCCCAATTTT 9049

Lsat_Salinas 9051 AGATTCAAATCCAAGAATCATTCAGGAATTCTCAGTCAACGAATAGTTAA 9100

Lsat_WENDEL 9051 AGATTCAAATCCAAGAATCATTCAGGAATTCTCAGTCAACGAATAGTTAA 9100

Lser_US96UC23 9051 AGATTCAAATCCAAGAATCATTCAGGAATTCTCAGTCAACGAATAGTTAA 9100

Lser_LAC005780 9051 AGATTCAAATCCAAGAATCATTCAGGAATTCTCAGTCAACGAATAGTTAA 9100

Lvir_CGN013357 9051 AGATTCAAATCCAAGAATCATTCAGGAATTCTCAGTCAACGAATAGTTAA 9100

Lsal_LAC008020 9050 AGATTCAAATCCAAGAATCATTCAGGAATTCTCAGTCAACGAATAGTTAA 9099

Lsat_Salinas 9101 TGGTTCCCATTTGTCATAAATTTTGTGACATAAATTTTGGCTTTTTTGAA 9150

Lsat_WENDEL 9101 TGGTTCCCATTTGTCATAAATTTTGTGACATAAATTTTGGCTTTTTTGAA 9150

Lser_US96UC23 9101 TGGTTCCCATTTGTCATAAATTTTGTGACATAAATTTTGGCTTTTTTGAA 9150

Lser_LAC005780 9101 TGGTTCCCATTTGTCATAAATTTTGTGACATAAATTTTGGCTTTTTTGAA 9150

Lvir_CGN013357 9101 TGGTTCCCATTTGTCATAAATTTTGTGTCATAAATTTTGGCTTTTTTGAA 9150

Lsal_LAC008020 9100 TGGTTCCCATTTGTCATAAATTTTGTGTCATAAATTTTGGCTTTTTTGAA 9149

Lsat_Salinas 9151 TGAAAATATACATTTGATTTTTCAATAGAAAAGTGAGGGGAAGTTTTTCG 9200

Lsat_WENDEL 9151 TGAAAATATACATTTGATTTTTCAATAGAAAAGTGAGGGGAAGTTTTTCG 9200

Lser_US96UC23 9151 TGAAAATATACATTTGATTTTTCAATAGAAAAGTGAGGGGAAGTTTTTCG 9200

Lser_LAC005780 9151 TGAAAATATACATTTGATTTTTCAATAGAAAAGTGAGGGGAAGTTTTTCG 9200

Lvir_CGN013357 9151 TGAAAATATACATTTGATTTTTCAATAGAAAAGTGAGGGGAAGTTTTTCG 9200

Lsal_LAC008020 9150 TGAAAATATACATTTGATTTTTCAATAGAAAAGTGAGGGGAAGTTTTTCG 9199

Lsat_Salinas 9201 ACATTTTATGTTTTGAGATACTATACAATCAATCGAAGGGGTGGTCAAAC 9250

Lsat_WENDEL 9201 ACATTTTATGTTTTGAGATACTATACAATCAATCGAAGGGGTGGTCAAAC 9250

Lser_US96UC23 9201 ACATTTTATGTTTTGAGATACTATACAATCAATCGAAGGGGTGGTCAAAC 9250

Lser_LAC005780 9201 ACATTTTATGTTTTGAGATACTATACAATCAATCGAAGGGGTGGTCAAAC 9250

Lvir_CGN013357 9201 ACATTTTATGTTTTGAGATACTATACAATCAATCGAAGGGGTGGTCAAAC 9250

Lsal_LAC008020 9200 ACATTTTATGTTTTGAGATACTATACAATCAATCGAAGGGGTGGTCAAAC 9249

Lsat_Salinas 9251 AAAAGGGGAAAAGGGTTTCTTTTAGAATTTTTATAAATTTAGAAAAAAAA 9300

Lsat_WENDEL 9251 AAAAGGGGAAAAGGGTTTCTTTTAGAATTTTTATAAATTTAGAAAAAAAA 9300

Lser_US96UC23 9251 AAAAGGGGAAAAGGGTTTCTTTTAGAATTTTTATAAATTTAGAAAAAAAA 9300

Lser_LAC005780 9251 AAAAGGGGAAAAGGGTTTCTTTTAGAATTTTTATAAATTTAGAAAAAAAA 9300

Lvir_CGN013357 9251 AAAAGGGGAAAAGGGTTTCTTTTAGAATTTTTATAAATTTAGAAAAAAAA 9300

Lsal_LAC008020 9250 AAAAGGGGAAAAGGGTTTCTTTTAGAATTTTTATAAATTTAGAAAAAAA- 9298

Lsat_Salinas 9301 GGATGAAATTAAAAAAGGGATGCAAGTACAATAAACTAAAGTTTAGTAAT 9350

Lsat_WENDEL 9301 GGATGAAATTAAAAAAGGGATGCAAGTACAATAAACTAAAGTTTAGTAAT 9350

Lser_US96UC23 9301 GGATGAAATTAAAAAAGGGATGCAAGTACAATAAACTAAAGTTTAGTAAT 9350

Lser_LAC005780 9301 GGATGAAATTAAAAAAGGGATGCAAGTACAATAAACTAAAGTTTAGTAAT 9350

Lvir_CGN013357 9301 GGATGAAATTAAAAAAGGGATGCAAGTACAATAAACTAAAGTTTAGTAAT 9350

Lsal_LAC008020 9299 GGATGAAATTAAAAAAGGGATGCAAGTACAATAAACTAAAGTTTAGTAAT 9348

Lsat_Salinas 9351 CCAACCCATAAAATTTTATCTTATTATGTATCGTTTTGGCGGCATGGCCG 9400

Lsat_WENDEL 9351 CCAACCCATAAAATTTTATCTTATTATGTATCGTTTTGGCGGCATGGCCG 9400

Lser_US96UC23 9351 CCAACCCATAAAATTTTATCTTATTATGTATCGTTTTGGCGGCATGGCCG 9400

Lser_LAC005780 9351 CCAACCCATAAAATTTTATCTTATTATGTATCGTTTTGGCGGCATGGCCG 9400

Lvir_CGN013357 9351 CCAACCCATAAAATTTTATCTTATTGTGTATCGTTTTGGCGGCATGGCCG 9400

Lsal_LAC008020 9349 CCAACCCATAAAATTTTATCTTATTGTGTATCGTTTTGGCGGCATGGCCG 9398

Lsat_Salinas 9401 AGTGGTAAGGCGGGGGACTGCAAATCCTTTTTCCCCAGTTCAAATCCGGG 9450

Lsat_WENDEL 9401 AGTGGTAAGGCGGGGGACTGCAAATCCTTTTTCCCCAGTTCAAATCCGGG 9450

Lser_US96UC23 9401 AGTGGTAAGGCGGGGGACTGCAAATCCTTTTTCCCCAGTTCAAATCCGGG 9450

Lser_LAC005780 9401 AGTGGTAAGGCGGGGGACTGCAAATCCTTTTTCCCCAGTTCAAATCCGGG 9450

Lvir_CGN013357 9401 AGTGGTAAGGCGGGGGACTGCAAATCCTTTTTCCCCAGTTCAAATCCGGG 9450

Lsal_LAC008020 9399 AGTGGTAAGGCGGGGGACTGCAAATCCTTTTTCCCCAGTTCAAATCCGGG 9448

Lsat_Salinas 9451 TGCCGCCTCATCAACAAACGAATCAAAATTTATTATCTGCTGATATAAAT 9500

Lsat_WENDEL 9451 TGCCGCCTCATCAACAAACGAATCAAAATTTATTATCTGCTGATATAAAT 9500

Lser_US96UC23 9451 TGCCGCCTCATCAACAAACGAATCAAAATTTATTATCTGCTGATATAAAT 9500

Lser_LAC005780 9451 TGCCGCCTCATCAACAAACGAATCAAAATTTATTATCTGCTGATATAAAT 9500

Lvir_CGN013357 9451 TGCCGCCTCATCAACAAACGAATCAAAATTTATTATCTGCTGATATAAAT 9500

Lsal_LAC008020 9449 TGCCGCCTCATCAACAAACGAATCAAAATTTATTATCTGCTGATATAAAT 9498

Lsat_Salinas 9501 CACCCAAATTTTACCCAACAGATAAGGCGATTGTTGATACTTGGTTGATT 9550

Lsat_WENDEL 9501 CACCCAAATTTTACCCAACAGATAAGGCGATTGTTGATACTTGGTTGATT 9550

Lser_US96UC23 9501 CACCCAAATTTTACCCAACAGATAAGGCGATTGTTGATACTTGGTTGATT 9550

Lser_LAC005780 9501 CACCCAAATTTTACCCAACAGATAAGGCGATTGTTGATACTTGGTTGATT 9550

Lvir_CGN013357 9501 CACCCAAATTTTACCCAACAGATAAGGCGATTGTTGATACTTGGTTGATT 9550

Lsal_LAC008020 9499 CACCCAAATTTTACCCAACAGATAAGGCGATTGTTGATACTTGGTTGATT 9548

Lsat_Salinas 9551 CTAAACATCTGTTCTGGGGATTTTGTAAAAGATTGGAAATCTTTCAATCT 9600

Lsat_WENDEL 9551 CTAAACATCTGTTCTGGGGATTTTGTAAAAGATTGGAAATCTTTCAATCT 9600

Lser_US96UC23 9551 CTAAACATCTGTTCTGGGGATTTTGTAAAAGATTGGAAATCTTTCAATCT 9600

Lser_LAC005780 9551 CTAAACATCTGTTCTGGGGATTTTGTAAAAGATTGGAAATCTTTCAATCT 9600

Lvir_CGN013357 9551 CTAAACATCTGTTCTGGGRATTTTGTAAAAGATTGGAAATCTTTCAATCT 9600

Lsal_LAC008020 9549 CTAAACATCTGTTCTGGGGATTTTGTAAAAGATTGGAAATCTTTCAATCT 9598

Lsat_Salinas 9601 AAAATTCAAATAAAGATTATATTATAATGGTCGAAGCAAGACTTCCCGAT 9650

Lsat_WENDEL 9601 AAAATTCAAATAAAGATTATATTATAATGGTCGAAGCAAGACTTCCCGAT 9650

Lser_US96UC23 9601 AAAATTCAAATAAAGATTATATTATAATGGTCGAAGCAAGACTTCCCGAT 9650

Lser_LAC005780 9601 AAAATTCAAATAAAGATTATATTATAATGGTCGAAGCAAGACTTCCCGAT 9650

Lvir_CGN013357 9601 AAAATTCAAATAAAGATTATATTATAATGGTCGAAGCAAGACTTCCCGAT 9650

Lsal_LAC008020 9599 AAAATTCAAATAAAGATTATATTATAATGGTCGAAGCAAGACTTCCCGAT 9648

Lsat_Salinas 9651 TCCCTTCTACTGCTAATGTCTACTGATTGGGTCTTGAATTTCATTGGCAT 9700

Lsat_WENDEL 9651 TCCCTTCTACTGCTAATGTCTACTGATTGGGTCTTGAATTTCATTGGCAT 9700

Lser_US96UC23 9651 TCCCTTCTACTGCTAATGTCTACTGATTGGGTCTTGAATTTCATTGGCAT 9700

Lser_LAC005780 9651 TCCCTTCTACTGCTAATGTCTACTGATTGGGTCTTGAATTTCATTGGCAT 9700

Lvir_CGN013357 9651 TCCCTTCTACTGCTAATGTCTACTAATTGGGTCTTGAATTTCATTGGCAT 9700

Lsal_LAC008020 9649 TCCCTTCTACTGCTAATGTCTACTGATTGGGTCTTGAATTTCATTGGCAT 9698

Lsat_Salinas 9701 AGCCGCCGGCGGCTAACTAGTTGTGGAAAGTCCTTTATGTAATCTACATA 9750

Lsat_WENDEL 9701 AGCCGCCGGCGGCTAACTAGTTGTGGAAAGTCCTTTATGTAATCTACATA 9750

Lser_US96UC23 9701 AGCCGCCGGCGGCTAACTAGTTGTGGAAAGTCCTTTATGTAATCTACATA 9750

Lser_LAC005780 9701 AGCCGCCGGCGGCTAACTAGTTGTGGAAAGTCCTTTATGTAATCTACATA 9750

Lvir_CGN013357 9701 AGCCGCCGGCGGCTAACTAGTTGTGGGAAGTCCTTTATGTAATCTACATA 9750

Lsal_LAC008020 9699 AGCCGCCGGCGGCTAACTAGTTGTGGAAAGTCCTTTATGTAATCTACATA 9748

Lsat_Salinas 9751 TATAGACTCGCGAGAATCTCTGAGTGTTCAGGCATTCAATCACTATTAGA 9800

Lsat_WENDEL 9751 TATAGACTCGCGAGAATCTCTGAGTGTTCAGGCATTCAATCACTATTAGA 9800

Lser_US96UC23 9751 TATAGACTCGCGAGAATCTCTGAGTGTTCAGGCATTCAATCACTATTAGA 9800

Lser_LAC005780 9751 TATAGACTCGCGAGAATCTCTGAGTGTTCAGGCATTCAATCACTATTAGA 9800

Lvir_CGN013357 9751 TATAGACTCGCGAGAATCTCTGAGTGTTCAGGCATTCAATCACTATTAGA 9800

Lsal_LAC008020 9749 TATAGACTCGCGAGAATCTCTGAGTGTTCAGGCATTCAATCACTATTAGA 9798

Lsat_Salinas 9801 TTGAGATTGGATGGATAATTTACTTTTTTAAAGTGAAAAAAAAATTATTA 9850

Lsat_WENDEL 9801 TTGAGATTGGATGGATAATTTACTTTTTTAAAGTGAAAAAAAAATTATTA 9850

Lser_US96UC23 9801 TTGAGATTGGATGGATAATTTACTTTTTTAAAGTGAAAAAAAAATTATTA 9850

Lser_LAC005780 9801 TTGAGATTGGATGGATAATTTACTTTTTTAAAGTGAAAAAAAAATTATTA 9850

Lvir_CGN013357 9801 TTGAGATTGGATGGATAATTTACTTTTTTAAAGTGAAAAAAAAATTATTA 9850

Lsal_LAC008020 9799 TTGAGATTGGATGGATAATTTACTTTTTTAAAGTGAAAAAAAAATTATTA 9848

Lsat_Salinas 9851 TTATTTTTTTTTTTAACCCCTCGTCAAGAGTATAATATACTATCTCCCAA 9900

Lsat_WENDEL 9851 TTATTTTTTTTTTTAACCCCTCGTCAAGAGTATAATATACTATCTCCCAA 9900

Lser_US96UC23 9851 TTATTTTTTTTTTTAACCCCTCGTCAAGAGTATAATATACTATCTCCCAA 9900

Lser_LAC005780 9851 TTATTTTTTTTTTTAACCCCTCGTCAAGAGTATAATATACTATCTCCCAA 9900

Lvir_CGN013357 9851 TTATTTTTTTTTTAAACCCCTCGCCAAGAGTATAATATACTATCTCCCAA 9900

Lsal_LAC008020 9849 TTATTTTTTTTTT-AACCCCTCGTCAAGAGTATAATATACTATCTCCCAA 9897

Lsat_Salinas 9901 CTGCTTCAGAGAGACAATCGGGAAAGGAAAGGGTACGGATTAGATCAAAT 9950

Lsat_WENDEL 9901 CTGCTTCAGAGAGACAATCGGGAAAGGAAAGGGTACGGATTAGATCAAAT 9950

Lser_US96UC23 9901 CTGCTTCAGAGAGACAATCGGGAAAGGAAAGGGTACGGATTAGATCAAAT 9950

Lser_LAC005780 9901 CTGCTTCAGAGAGACAATCGGGAAAGGAAAGGGTACGGATTAGATCAAAT 9950

Lvir_CGN013357 9901 CTGCTTCAGAGAGACAATCGGGAAAGGAAAGGGTACGGATTAGATCAAAT 9950

Lsal_LAC008020 9898 CTGCTTCAGAGAGACAATCGGGAAAGGAAAGGGTACGGATTAGATCAAAT 9947

Lsat_Salinas 9951 AGGAAATAAAAGTATGTAATAGATAGCAATTGATCTATTTGTACTTTGAA 10000

Lsat_WENDEL 9951 AGGAAATAAAAGTATGTAATAGATAGCAATTGATCTATTTGTACTTTGAA 10000

Lser_US96UC23 9951 AGGAAATAAAAGTATGTAATAGATAGCAATTGATCTATTTGTACTTTGAA 10000

Lser_LAC005780 9951 AGGAAATAAAAGTATGTAATAGATAGCAATTGATCTATTTGTACTTTGAA 10000

Lvir_CGN013357 9951 AGGAAATAAAAGTCTGTAATAGATAGCAATTGATCTATTTGTACTKTGAA 10000

Lsal_LAC008020 9948 AGGAAATAAAAGTATGTAATAGATAGCAATTGATCTATTTGTACTTTGAA 9997

Lsat_Salinas 10001 GGGCAACAAAGAATGGGCCCTGTTTTTTTATTACTATATGTTCCATTAGT 10050

Lsat_WENDEL 10001 GGGCAACAAAGAATGGGCCCTGTTTTTTTATTACTATATGTTCCATTAGT 10050

Lser_US96UC23 10001 GGGCAACAAAGAATGGGCCCTGTTTTTTTATTACTATATGTTCCATTAGT 10050

Lser_LAC005780 10001 GGGCAACAAAGAATGGGCCCTGTTTTTTTATTACTATATGTTCCATTAGT 10050

Lvir_CGN013357 10001 GGGCAACAAAGAATGGGCCCTGTTTTTTTATTACTATATGTTCCATTAGT 10050

Lsal_LAC008020 9998 GGGCAACAAAGAATGGGCCCTGTTTTTTTATTACTATATGTTCCATTAGT 10047

Lsat_Salinas 10051 AACATTCCTTTGTAGCGTCATTGTGTATTTTAGTTGTGTTTAGTCTTTCC 10100

Lsat_WENDEL 10051 AACATTCCTTTGTAGCGTCATTGTGTATTTTAGTTGTGTTTAGTCTTTCC 10100

Lser_US96UC23 10051 AACATTCCTTTGTAGCGTCATTGTGTATTTTAGTTGTGTTTAGTCTTTCC 10100

Lser_LAC005780 10051 AACATTCCTTTGTAGCGTCATTGTGTATTTTAGTTGTGTTTAGTCTTTCC 10100

Lvir_CGN013357 10051 AACATTCCTTTGTAGCGTCATTGTGTATTTTAGTTGTGTTTAGTCTTTCC 10100

Lsal_LAC008020 10048 AACATTCCTTTGTAGCGTCATTGTGTATTTTAGTTGTGTTTAGTCTTTCC 10097

Lsat_Salinas 10101 CGGTTAGAAATCATATAGGAATTTTTTAGAAATGGATTTATTTGATTGGT 10150

Lsat_WENDEL 10101 CGGTTAGAAATCATATAGGAATTTTTTAGAAATGGATTTATTTGATTGGT 10150

Lser_US96UC23 10101 CGGTTAGAAATCATATAGGAATTTTTTAGAAATGGATTTATTTGATTGGT 10150

Lser_LAC005780 10101 CGGTTAGAAATCATATAGGAATTTTTTAGAAATGGATTTATTTGATTGGT 10150

Lvir_CGN013357 10101 CGGTTAGAAATCATATAGGAATTTTTTAGAAATGGATTTATTTGATTGGT 10150

Lsal_LAC008020 10098 CGGTTAGAAATCATATAGGAATTTTTTAGAAATGGATTTATTTGATTGGT 10147

Lsat_Salinas 10151 TCATCAATTTTATTTGATTGGTTCATCAATAGTGTTCGGCCAGAATCCCT 10200

Lsat_WENDEL 10151 TCATCAATTTTATTTGATTGGTTCATCAATAGTGTTCGGCCAGAATCCCT 10200

Lser_US96UC23 10151 TCATCAATTTTATTTGATTGGTTCATCAATAGTGTTCGGCCAGAATCCCT 10200

Lser_LAC005780 10151 TCATCAATTTTATTTGATTGGTTCATCAATAGTGTTCGGCCAGAATCCCT 10200

Lvir_CGN013357 10151 TCATCAATTTTATTTGATTGGTTCATCAATAGTGTTCGGCCAGAATCCCT 10200

Lsal_LAC008020 10148 TCATCAATTTTATTTGATTGGTTCATCAATAGTGTTCGGCCAGAATCCCT 10197

Lsat_Salinas 10201 TTTTGACTCTGGACCATGGATTCTACTATTATTAGTGAGCAATACAATAA 10250

Lsat_WENDEL 10201 TTTTGACTCTGGACCATGGATTCTACTATTATTAGTGAGCAATACAATAA 10250

Lser_US96UC23 10201 TTTTGACTCTGGACCATGGATTCTACTATTATTAGTGAGCAATACAATAA 10250

Lser_LAC005780 10201 TTTTGACTCTGGACCATGGATTCTACTATTATTAGTGAGCAATACAATAA 10250

Lvir_CGN013357 10201 TTTTGACTCTGGACCATGGATTCTACTATTATTAGTGAGCAATACAATAA 10250

Lsal_LAC008020 10198 TTTTGACTCTGGACCATGGATTCTACTATTATTAGTGAGCAATACAATAA 10247

Lsat_Salinas 10251 TGGAATATTTCTAGCATAAAGATAAGGGACATAATTGACATGGATATAGT 10300

Lsat_WENDEL 10251 TGGAATATTTCTAGCATAAAGATAAGGGACATAATTGACATGGATATAGT 10300

Lser_US96UC23 10251 TGGAATATTTCTAGCATAAAGATAAGGGACATAATTGACATGGATATAGT 10300

Lser_LAC005780 10251 TGGAATATTTCTAGCATAAAGATAAGGGACATAATTGACATGGATATAGT 10300

Lvir_CGN013357 10251 TGGAATATTTCTAGCATAAAGATAAGGGACATAATTGACATGGATATAGT 10300

Lsal_LAC008020 10248 TGGAATATTTCTAGCATAAAGATAAGGGACATAATTGACATGGATATAGT 10297

Lsat_Salinas 10301 AAGTCTCGCTTGGGCTGCTTTAATGGTAGTCTTTACATTTTCCCTTTCAC 10350

Lsat_WENDEL 10301 AAGTCTCGCTTGGGCTGCTTTAATGGTAGTCTTTACATTTTCCCTTTCAC 10350

Lser_US96UC23 10301 AAGTCTCGCTTGGGCTGCTTTAATGGTAGTCTTTACATTTTCCCTTTCAC 10350

Lser_LAC005780 10301 AAGTCTCGCTTGGGCTGCTTTAATGGTAGTCTTTACATTTTCCCTTTCAC 10350

Lvir_CGN013357 10301 AAGTCTCGCTTGGGCTGCTTTAATGGTAGTCTTTACATTTTCCCTTTCAC 10350

Lsal_LAC008020 10298 AAGTCTCGCTTGGGCTGCTTTAATGGTAGTCTTTACATTTTCCCTTTCAC 10347

Lsat_Salinas 10351 TCGTAGTATGGGGAAGAAGTGGACTTTAGAAGGACTACTAATTTAGTTTA 10400

Lsat_WENDEL 10351 TCGTAGTATGGGGAAGAAGTGGACTTTAGAAGGACTACTAATTTAGTTTA 10400

Lser_US96UC23 10351 TCGTAGTATGGGGAAGAAGTGGACTTTAGAAGGACTACTAATTTAGTTTA 10400

Lser_LAC005780 10351 TCGTAGTATGGGGAAGAAGTGGACTTTAGAAGGACTACTAATTTAGTTTA 10400

Lvir_CGN013357 10351 TCGTAGTATGGGGAAGAAGTGGACTTTAGAAGGACTACTAATTTAGTTTA 10400

Lsal_LAC008020 10348 TCGTAGTATGGGGAAGAAGTGGACTTTAGAAGGACTACTAATTTAGTTTA 10397

Lsat_Salinas 10401 GGAATGAAATGGTATCAATTGTTTTATAGATCGTTCTGCAACGCATTTTT 10450

Lsat_WENDEL 10401 GGAATGAAATGGTATCAATTGTTTTATAGATCGTTCTGCAACGCATTTTT 10450

Lser_US96UC23 10401 GGAATGAAATGGTATCAATTGTTTTATAGATCGTTCTGCAACGCATTTTT 10450

Lser_LAC005780 10401 GGAATGAAATGGTATCAATTGTTTTATAGATCGTTCTGCAACGCATTTTT 10450

Lvir_CGN013357 10401 GGAATGAAATGGTATCAATTGTTTTATAGATCGTTCTGCAACGCATTTTT 10450

Lsal_LAC008020 10398 GGAATGAAATGGTATCAATTGTTTTATAGATCGTTCTGCAACGCATTTTT 10447

Lsat_Salinas 10451 TATTTTTTATTTGAATTGAAATAATTTTCAAATCAAAATTTATTCATTTC 10500

Lsat_WENDEL 10451 TATTTTTTATTTGAATTGAAATAATTTTCAAATCAAAATTTATTCATTTC 10500

Lser_US96UC23 10451 TATTTTTTATTTGAATTGAAATAATTTTCAAATCAAAATTTATTCATTTC 10500

Lser_LAC005780 10451 TATTTTTTATTTGAATTGAAATAATTTTCAAATCAAAATTTATTCATTTC 10500

Lvir_CGN013357 10451 TMTTTTTTATTTGAATTGAAATAATTTTCAAATCAAAATTTATTCATTTC 10500

Lsal_LAC008020 10448 TATTTTTTATTTGAATTGAAATAATTTTCAAATCAAAATTTATTCATTTC 10497

Lsat_Salinas 10501 GAAATTCCATTGGATTCTAGTGGAGAAATGTATTATATAACAGTAAAACA 10550

Lsat_WENDEL 10501 GAAATTCCATTGGATTCTAGTGGAGAAATGTATTATATAACAGTAAAACA 10550

Lser_US96UC23 10501 GAAATTCCATTGGATTCTAGTGGAGAAATGTATTATATAACAGTAAAACA 10550

Lser_LAC005780 10501 GAAATTCCATTGGATTCTAGTGGAGAAATGTATTATATAACAGTAAAACA 10550

Lvir_CGN013357 10501 GAAATTCCATTGGATTCTAGTGGAGAAATGTATTATATAACAGTAAAACA 10550

Lsal_LAC008020 10498 GAAATTCCATTGGATTCTAGTGGAGAAATGTATTATATAACAGCAAAACA 10547

Lsat_Salinas 10551 ATTATTCAGTAAAACAATTATTTCAGAAAGAAAGAGAATTGGGTCCTACG 10600

Lsat_WENDEL 10551 ATTATTCAGTAAAACAATTATTTCAGAAAGAAAGAGAATTGGGTCCTACG 10600

Lser_US96UC23 10551 ATTATTCAGTAAAACAATTATTTCAGAAAGAAAGAGAATTGGGTCCTACG 10600

Lser_LAC005780 10551 ATTATTCAGTAAAACAATTATTTCAGAAAGAAAGAGAATTGGGTCCTACG 10600

Lvir_CGN013357 10551 ATWATTCAGTAAAACAATTATTTCAGAAAGAAAGAGAATTGGGTCCTACG 10600

Lsal_LAC008020 10548 ATTATTCAGCAAAACAATTATTTCAGAAAGAAAGAGAATTGGGTCCTACG 10597

Lsat_Salinas 10601 TTAATTCCATATATGGATTAATCACTACATATATTGAAGATAGAAGCTAA 10650

Lsat_WENDEL 10601 TTAATTCCATATATGGATTAATCACTACATATATTGAAGATAGAAGCTAA 10650

Lser_US96UC23 10601 TTAATTCCATATATGGATTAATCACTACATATATTGAAGATAGAAGCTAA 10650

Lser_LAC005780 10601 TTAATTCCATATATGGATTAATCACTACATATATTGAAGATAGAAGCTAA 10650

Lvir_CGN013357 10601 TTAATTCCATATATGGATTAATCACTACATATATTGAAGATAGAAGCTAA 10650

Lsal_LAC008020 10598 TTAATTCCATATATGGATTAATCACTACATATATTGAAGATAGAAGCTAA 10647

Lsat_Salinas 10651 TATAGTATACCAACTTTATTAGAAAGTAAAGTACAACTTTATACACTACT 10700

Lsat_WENDEL 10651 TATAGTATACCAACTTTATTAGAAAGTAAAGTACAACTTTATACACTACT 10700

Lser_US96UC23 10651 TATAGTATACCAACTTTATTAGAAAGTAAAGTACAACTTTATACACTACT 10700

Lser_LAC005780 10651 TATAGTATACCAACTTTATTAGAAAGTAAAGTACAACTTTATACACTACT 10700

Lvir_CGN013357 10651 TATAGTATACCAACT--ATTAGAAAGTAAAGTACAACTTTATACACTACT 10698

Lsal_LAC008020 10648 TATAGTATACCAACTTTATTAGAAAGTAAAGTACAACTTTATACACTACT 10697

Lsat_Salinas 10701 ATAACACTAATAGAGAGTATGGTAAGAAATTTCTTACCATACTCTCGGAT 10750

Lsat_WENDEL 10701 ATAACACTAATAGAGAGTATGGTAAGAAATTTCTTACCATACTCTCGGAT 10750

Lser_US96UC23 10701 ATAACACTAATAGAGAGTATGGTAAGAAATTTCTTACCATACTCTCGGAT 10750

Lser_LAC005780 10701 ATAACACTAATAGAGAGTATGGTAAGAAATTTCTTACCATACTCTCGGAT 10750

Lvir_CGN013357 10699 ATAACACTAATAGAGAGTATGGTAAGAAATTTCTTACCATACTCTCGGAT 10748

Lsal_LAC008020 10698 ATAACACTAATAGAGAGTATGGTAAGAAATTTCTTACCATACTCTCGGAT 10747

Lsat_Salinas 10751 CTCATAGAATACCGCTCATTATAGCCCGTTGATTTCATTTAAAACGCAAA 10800

Lsat_WENDEL 10751 CTCATAGAATACCGCTCATTATAGCCCGTTGATTTCATTTAAAACGCAAA 10800

Lser_US96UC23 10751 CTCATAGAATACCGCTCATTATAGCCCGTTGATTTCATTTAAAACGCAAA 10800

Lser_LAC005780 10751 CTCATAGAATACCGCTCATTATAGCCCGTTGATTTCATTTAAAACGCAAA 10800

Lvir_CGN013357 10749 CTCATAGAATACCGCTCATTATAGCCCGTTGATTTCATTTAAAACGCAAA 10798

Lsal_LAC008020 10748 CTCATAGAATACCGCTCATTATAGCCCGTTGATTTCATTTAAAACGCAAA 10797

Lsat_Salinas 10801 AAAATAATTCTTTTGATTTGATTTCTTCAACTATTGATAAGAACTCAGAA 10850

Lsat_WENDEL 10801 AAAATAATTCTTTTGATTTGATTTCTTCAACTATTGATAAGAACTCAGAA 10850

Lser_US96UC23 10801 AAAATAATTCTTTTGATTTGATTTCTTCAACTATTGATAAGAACTCAGAA 10850

Lser_LAC005780 10801 AAAATAATTCTTTTGATTTGATTTCTTCAACTATTGATAAGAACTCAAAA 10850

Lvir_CGN013357 10799 AAAATAATTCTTTTGATTTGATTTCTTCAACTATTGATAAGAACTCAGAA 10848

Lsal_LAC008020 10798 AAAATAATTCTTTTGATTTGATTTCTTCAACTATTGATAAGAACTCAGAA 10847

Lsat_Salinas 10851 GTCAAGTTTCATTTCAAGTAATTAATTATTTTGACTGACTGTTTTTACGT 10900

Lsat_WENDEL 10851 GTCAAGTTTCATTTCAAGTAATTAATTATTTTGACTGACTGTTTTTACGT 10900

Lser_US96UC23 10851 GTCAAGTTTCATTTCAAGTAATTAATTATTTTGACTGACTGTTTTTACGT 10900

Lser_LAC005780 10851 GTCAAGTTTCATTTCAAGTAATTAATTATTTTGACTGACTGTTTTTACGT 10900

Lvir_CGN013357 10849 GTCAAGTTTCATTTCAAGTAATTAATTATTTTGACTGACTGTTTTTACGT 10898

Lsal_LAC008020 10848 GTCAAGTTTCATTTCAAGTAATTAATTATTTTGACTGACTGTTTTTACGT 10897

Lsat_Salinas 10901 AAATGATAAGTAGAAAAGCAGTAGGAACTAAAATGAACAGTGCAGTAGCA 10950

Lsat_WENDEL 10901 AAATGATAAGTAGAAAAGCAGTAGGAACTAAAATGAACAGTGCAGTAGCA 10950

Lser_US96UC23 10901 AAATGATAAGTAGAAAAGCAGTAGGAACTAAAATGAACAGTGCAGTAGCA 10950

Lser_LAC005780 10901 AAATGATAAGTAGAAAAGCAGTAGGAACTAAAATGAACAGTGCAGTAGCA 10950

Lvir_CGN013357 10899 AAATGATAAGTAGAAAAGCAGTAGGAACTAAAATGAACAGTGCAGTAGCA 10948

Lsal_LAC008020 10898 AAATGATAAGTAGAAAAGCAGTAGGAACTAAAATGAACAGTGCAGTAGCA 10947

Lsat_Salinas 10951 ATAAATGCAAGAATATTTACTTCCATAATCTCAATCTCATCGTTTTTTTT 11000

Lsat_WENDEL 10951 ATAAATGCAAGAATATTTACTTCCATAATCTCAATCTCATCGTTTTTTTT 11000

Lser_US96UC23 10951 ATAAATGCAAGAATATTTACTTCCATAATCTCAATCTCATCGTTTTTTTT 11000

Lser_LAC005780 10951 ATAAATGCAAGAATATTTACTTCCATAATCTCAATCTCATCGTTTTTTTT 11000

Lvir_CGN013357 10949 ATAAATGCAAGAATATTTACTTCCATAATCTCAATCTCATCGTTTTTTTT 10998

Lsal_LAC008020 10948 ATAAATGCAAGAATATTTACTTCCATAATCTCAATCTCATCGTTTTTTTT 10997

Lsat_Salinas 11001 TATTTCACAATAACTCGGGATTTAATCCCATAGAGATGATAAATCTTTCA 11050

Lsat_WENDEL 11001 TATTTCACAATAACTCGGGATTTAATCCCATAGAGATGATAAATCTTTCA 11050

Lser_US96UC23 11001 TATTTCACAATAACTCGGGATTTAATCCCATAGAGATGATAAATCTTTCA 11050

Lser_LAC005780 11001 TATTTCACAATAACTCGGGATTTAATCCCATAGAGATGATAAATCTTTCA 11050

Lvir_CGN013357 10999 TATTTCACAATAACTCGGGATTTAATCCCATAGAGATGATAAATCTTTCA 11048

Lsal_LAC008020 10998 TATTTCACAATAACTCGGGATTTAATCCCATAGAGATGATAAATCTTTCA 11047

Lsat_Salinas 11051 CCTGTCAATTCCATGAATGCATTACCTATCGATGATCTTGAATCGGATCA 11100

Lsat_WENDEL 11051 CCTGTCAATTCCATGAATGCATTACCTATCGATGATCTTGAATCGGATCA 11100

Lser_US96UC23 11051 CCTGTCAATTCCATGAATGCATTACCTATCGATGATCTTGAATCGGATCA 11100

Lser_LAC005780 11051 CCTGTCAATTCCATGAATGCATTACCTATCGATGATCTTGAATCGGATCA 11100

Lvir_CGN013357 11049 CCTGTCAATTCAATGAATGCATTACCTATCGATGATCTTGAATCGGATCA 11098

Lsal_LAC008020 11048 CCTGTCAATTCAATGAATGCATTACCTATCGATGATCTTGAATCGGATCA 11097

Lsat_Salinas 11101 ATATCATGAATAACAATATCTGAGCTATTAAATTAATTCGTCGTCGAGAA 11150

Lsat_WENDEL 11101 ATATCATGAATAACAATATCTGAGCTATTAAATTAATTCGTCGTCGAGAA 11150

Lser_US96UC23 11101 ATATCATGAATAACAATATCTGAGCTATTAAATTAATTCGTCGTCGAGAA 11150

Lser_LAC005780 11101 ATATCATGAATAACAATATCTGAGCTATTAAATTAATTCGTCGTCGAGAA 11150

Lvir_CGN013357 11099 ATATCATGAATAACAATATCTGAGCTATTAAATTAATTCGTCGTCGAGAA 11148

Lsal_LAC008020 11098 ATATCATGAATAACAATATCTGAGCTATTAAATTAATTCGTCGTCGAGAA 11147

Lsat_Salinas 11151 TTGAATAGTATAACATACGAAGATCTTTTATCCATACCGAATCCAAGATT 11200

Lsat_WENDEL 11151 TTGAATAGTATAACATACGAAGATCTTTTATCCATACCGAATCCAAGATT 11200

Lser_US96UC23 11151 TTGAATAGTATAACATACGAAGATCTTTTATCCATACCGAATCCAAGATT 11200

Lser_LAC005780 11151 TTGAATAGTATAACATACGAAGATCTTTTATCCATACCGAATCCAAGATT 11200

Lvir_CGN013357 11149 TTGAATAGTATAACATACGAAGATCTTTTATCCATACCGAATCCAAGATT 11198

Lsal_LAC008020 11148 TTGAATAGTATAACATACGAAGATCTTTTATCCATACCGAATCCAAGATT 11197

Lsat_Salinas 11201 GGATTCCCGGACCAATCAAAAATTCCTTTATTTATCCTTCTTTTCTGTTC 11250

Lsat_WENDEL 11201 GGATTCCCGGACCAATCAAAAATTCCTTTATTTATCCTTCTTTTCTGTTC 11250

Lser_US96UC23 11201 GGATTCCCGGACCAATCAAAAATTCCTTTATTTATCCTTCTTTTCTGTTC 11250

Lser_LAC005780 11201 GGATTCCCGGACCAATCAAAAATTCCTTTATTTATCCTTCTTTTCTGTTC 11250

Lvir_CGN013357 11199 GGATTCCCGGACCAATCAAAAATTCCTTTATTTATCCTTCTTTTCTGTTC 11248

Lsal_LAC008020 11198 GGATTCCCGGACCAATCAAAAATTCCTTTATTTATCCTTCTTTTCTTTTC 11247

Lsat_Salinas 11251 TTTCTTTTCTATAACCTACCTTAGGTCTTCCGTATAGAACCATCAAATGA 11300

Lsat_WENDEL 11251 TTTCTTTTCTATAACCTACCTTAGGTCTTCCGTATAGAACCATCAAATGA 11300

Lser_US96UC23 11251 TTTCTTTTCTATAACCTACCTTAGGTCTTCCGTATAGAACCATCAAATGA 11300

Lser_LAC005780 11251 TTTCTTTTCTATAACCTACCTTAGGTCTTCCGTATAGAACCATCAAATGA 11300

Lvir_CGN013357 11249 TTTCTTTTCTATAACCTACCTTAGGTCTTCCGTATAGAACCATCAAATGA 11298

Lsal_LAC008020 11248 TTTCTTTTCTATAACCTACCCTAGGTCTTCCGTATAGAACCATCAAATGA 11297

Lsat_Salinas 11301 AGTATCCTCGTCCGTTTCCATTTCCATTAACTACAAAACGCCAACAAAAA 11350

Lsat_WENDEL 11301 AGTATCCTCGTCCGTTTCCATTTCCATTAACTACAAAACGCCAACAAAAA 11350

Lser_US96UC23 11301 AGTATCCTCGTCCGTTTCCATTTCCATTAACTACAAAACGCCAACAAAAA 11350

Lser_LAC005780 11301 AGTATCCTCGTCCGTTTCCATTTCCATTAACTACAAAACGCCAACAAAAA 11350

Lvir_CGN013357 11299 AGTATCCTCGTCCGTTTCCATTTCCATTAACTACAAAACGCCAACAAAAA 11348

Lsal_LAC008020 11298 AGTATCCTCGTCCGTTTCCRTTTCCATTAACTACAAAACGCCAACAAAAA 11347

Lsat_Salinas 11351 ATACAAGCGAAGTGGAAAAAGAGAGGAATTAGGTTGTAAATTTGAATGAT 11400

Lsat_WENDEL 11351 ATACAAGCGAAGTGGAAAAAGAGAGGAATTAGGTTGTAAATTTGAATGAT 11400

Lser_US96UC23 11351 ATACAAGCGAAGTGGAAAAAGAGAGGAATTAGGTTGTAAATTTGAATGAT 11400

Lser_LAC005780 11351 ATACAAGCGAAGTGGAAAAAGAGAGGAATTAGGTTGTAAATTTGAATGAT 11400

Lvir_CGN013357 11349 ATACAAGCGAAGTGGAAAAAGAGAGGAATTAGGTTGTAAATTTGAATGAT 11398

Lsal_LAC008020 11348 ATACAAGCGAAGTGGAAAAAGAGAGGAATTAGGTTGTAAATTTGAATGAT 11397

Lsat_Salinas 11401 CCAATTTTATTTGGAAGACAAAGAAGTATGAGAAAGAGGAGTCGCGGGAG 11450

Lsat_WENDEL 11401 CCAATTTTATTTGGAAGACAAAGAAGTATGAGAAAGAGGAGTCGCGGGAG 11450

Lser_US96UC23 11401 CCAATTTTATTTGGAAGACAAAGAAGTATGAGAAAGAGGAGTCGCGGGAG 11450

Lser_LAC005780 11401 CCAATTTTATTTGGAAGACAAAGAAGTATGAGAAAGAGGAGTCGCGGGAG 11450

Lvir_CGN013357 11399 CCAATTTTATTTGGAAGACAAAGAAGTATGAGAAAGAGGAGTCGCGGGAG 11448

Lsal_LAC008020 11398 CCAATTTTATTTGGAAGACAAAGAAGTATGAGAAAGAGGAGTCGCGGGAG 11447

Lsat_Salinas 11451 AAAAGATGGAATATTCTATCAACTTCACTATTTTAGTTATTTTCATTTTA 11500

Lsat_WENDEL 11451 AAAAGATGGAATATTCTATCAACTTCACTATTTTAGTTATTTTCATTTTA 11500

Lser_US96UC23 11451 AAAAGATGGAATATTCTATCAACTTCACTATTTTAGTTATTTTCATTTTA 11500

Lser_LAC005780 11451 AAAAGATGGAATATTCTATCAACTTCACTATTTTAGTTATTTTCATTTTA 11500

Lvir_CGN013357 11449 AAAAGATGGAATATTCTATCAACTTCACTATTTTAGTTATTTTCATTTTA 11498

Lsal_LAC008020 11448 AAAAGATGGAATATTCTATCAACTTCACTGTTTTAGTTATTTTCATTTTA 11497

Lsat_Salinas 11501 TTTTAGTTTAGTGTTTGTTCTTTCTTCGACAGAATCCTGAAAAAAAGAGG 11550

Lsat_WENDEL 11501 TTTTAGTTTAGTGTTTGTTCTTTCTTCGACAGAATCCTGAAAAAAAGAGG 11550

Lser_US96UC23 11501 TTTTAGTTTAGTGTTTGTTCTTTCTTCGACAGAATCCTGAAAAAAAGAGG 11550

Lser_LAC005780 11501 TTTTAGTTTAGTGTTTGTTCTTTCTTCGACAGAATCCTGAAAAAAAGAGG 11550

Lvir_CGN013357 11499 TTTTAGTTTAGTGTTTGTTCTTTCTTCGACAGAATCCTGAAAAAAAGAGG 11548

Lsal_LAC008020 11498 TTTTAGTTTAGTGTTTGTTCTTTCTTCGACAGAATCCTGAAAAAAAGAGG 11547

Lsat_Salinas 11551 GGAAACCCCTTCGGAATTAAATTATGATCTCTGTCCCTTCTTTCATTTCA 11600

Lsat_WENDEL 11551 GGAAACCCCTTCGGAATTAAATTATGATCTCTGTCCCTTCTTTCATTTCA 11600

Lser_US96UC23 11551 GGAAACCCCTTCGGAATTAAATTATGATCTCTGTCCCTTCTTTCATTTCA 11600

Lser_LAC005780 11551 GGAAACCCCTTCGGAATTAAATTATGATCTCTGTCCCTTCTTTCATTTCA 11600

Lvir_CGN013357 11549 GGAAACCCCTTCGGAATTAAATTATGATCTCTGTCCCTTCTTTCATTTCA 11598

Lsal_LAC008020 11548 GGAAACCCCTTCGGAATTAAATTATGATCTCTGTCCCTTCTTTCATTTCA 11597

Lsat_Salinas 11601 CATAAAATGGAGAGGTGAATTGATGTACTTATTGGATCCGTCGGGACTGA 11650

Lsat_WENDEL 11601 CAKAAAATGGAGAGRKGAATTGATGTMYTTWTTGRATCCGTCGGGACTGA 11650

Lser_US96UC23 11601 CATAAAATGGAGAGGTGAATTGATGTACTTATTGGATCCGTCGGGACTGA 11650

Lser_LAC005780 11601 CAGAAAATGGAGAGAGGAATTGATGTCTTTTTTGAATCCGTCGGGACTGA 11650

Lvir_CGN013357 11599 CAKAAAATGGAGAGRKGAATTGATGTMYTTWTTGRATCCGTCGGGACTGA 11648

Lsal_LAC008020 11598 CATAAAATGGAGAGRKGAATTGATGTMYTTWTTGRATCCGTCGGGACTGA 11647

Lsat_Salinas 11651 CGGGGCTCGAACCCGCAACGTCCGCCTTGACAGGGCGGTGCTCTTGCCTA 11700

Lsat_WENDEL 11651 CGGGGCTCGAACCCGCAACTTCCGCCTTGACAGGGCGGTGCTCTGRCCKA 11700

Lser_US96UC23 11651 CGGGGCTCGAACCCGCAACGTCCGCCTTGACAGGGCGGTGCTCTTGCCTA 11700

Lser_LAC005780 11651 CGGGGCTCGAACCCGCAACTTCCGCCTTGACAGGGCGGTGCTCTGACCGA 11700

Lvir_CGN013357 11649 CGGGGCTCGAACCCGCAACKTCCGCCTTGACAGGGCGGTGCTCTKRCCKA 11698

Lsal_LAC008020 11648 CGGGGCTCGAACCCGCAACKTCCGCCTTGACAGGGCGGTGCTCTKRCCKA 11697

Lsat_Salinas 11701 TTGAACTACAATCCCAGGGAAATAAGAAGAGATCTAACAGAAAATTTGGC 11750

Lsat_WENDEL 11701 TTGAACTACAATCCCAGGRAAATAAGAAGAGATCTAACAGAAAATTTGGC 11750

Lser_US96UC23 11701 TTGAACTACAATCCCAGGGAAATAAGAAGAGATCTAACAGAAAATTTGGC 11750

Lser_LAC005780 11701 TTGAACTACAATCCCAGGGAAATAAGAAGAGATCTAACAGAAAATTTGGC 11750

Lvir_CGN013357 11699 TTGAACTACAATCCCAGGGAAATAAGAAGAGATCTAACAGAAAATTTGGC 11748

Lsal_LAC008020 11698 TTGAACTACAATCCCAGGRAAATAAGAAGAGATCTAACAGAAAATTTGGC 11747

Lsat_Salinas 11751 TTCTTTTTTATTCTCTCTTATCAGGTATTTCTTAAGAACAAGAGGGTTCT 11800

Lsat_WENDEL 11751 TTCTTTTTTATTCTCTCTTATCAGGTATTTCTTAAGAACAAGAGGGTTCT 11800

Lser_US96UC23 11751 TTCTTTTTTATTCTCTCTTATCAGGTATTTCTTAAGAACAAGAGGGTTCT 11800

Lser_LAC005780 11751 TTCTTTTTTATTCTCTCTTATCAGGTATTTCTTAAGAACAAGAGGGTTCT 11800

Lvir_CGN013357 11749 TTCTTTTTTATTCTCTCTTATCAGGTATTTCTTAAGAACAAGAGGGTTCT 11798

Lsal_LAC008020 11748 TTCTTTTTTATTCTCTCTTATCAGGTATTTCTTAAGAACAAGAGGGTTCT 11797

Lsat_Salinas 11801 ACCATTTGATAGTATATTGGCGAATTTTTGGGCCGAGCTGGATTTGAACC 11850

Lsat_WENDEL 11801 ACCATTTGATAGTATATTGGCGAATTTTTGGGCCGAGCTGGATTTGAACC 11850

Lser_US96UC23 11801 ACCATTTGATAGTATATTGGCGAATTTTTGGGCCGAGCTGGATTTGAACC 11850

Lser_LAC005780 11801 ACCATTTGATAGTATATTGGCGAATTTTTGGGCCGAGCTGGATTTGAACC 11850

Lvir_CGN013357 11799 ACCATTTGATAGTATATTGGCGAATTTTTGGGCCGAGCTGGATTTGAACC 11848

Lsal_LAC008020 11798 ACCATTTGATAGTATATTGGCGAATTTTTGGGCCGAGCTGGATTTGAACC 11847

Lsat_Salinas 11851 AGCGTAGACATATTGTCAACGAATTTACAGTCCGTCCCCATTAACCACTC 11900

Lsat_WENDEL 11851 AGCGTAGACATATTGTCAACGAATTTACAGTCCGTCCCCATTAACCACTC 11900

Lser_US96UC23 11851 AGCGTAGACATATTGTCAACGAATTTACAGTCCGTCCCCATTAACCACTC 11900

Lser_LAC005780 11851 AGCGTAGACATATTGTCAACGAATTTACAGTCCGTCCCCATTAACCACTC 11900

Lvir_CGN013357 11849 AGCGTAGACATATTGTCAACGAATTTACAGTCCGTCCCCATTAACCACTC 11898

Lsal_LAC008020 11848 AGCGTAGACATATTGTCAACGAATTTACAGTCCGTCCCCATTAACCACTC 11897

Lsat_Salinas 11901 GGGCATCGACCCAACGCCTAATTAGACGTTCCATAATCAACTTCCTTTCG 11950

Lsat_WENDEL 11901 GGGCATCGACCCAACGCCTAATTAGACGTTCCATAATCAACTTCCTTTCG 11950

Lser_US96UC23 11901 GGGCATCGACCCAACGCCTAATTAGACGTTCCATAATCAACTTCCTTTCG 11950

Lser_LAC005780 11901 GGGCATCGACCCAACGCCTAATTAGACGTTCCATAATCAACTTCCTTTCG 11950

Lvir_CGN013357 11899 GGGCATCGACCCAACGCCTAATTAGACGTTCCATAATCAACTTCCTTTCG 11948

Lsal_LAC008020 11898 GGGCATCGACCCAACGCCTAATTAGACGTTCCATAATCAACTTCCTTTCG 11947

Lsat_Salinas 11951 TCTACCAGGCGGACTTGACAAATACATTTCACTTTTGATAAAATCCTACT 12000

Lsat_WENDEL 11951 TCTACCAGGCGGACTTGACAAATACATTTCACTTTTGATAAAATCCTACT 12000

Lser_US96UC23 11951 TCTACCAGGCGGACTTGACAAATACATTTCACTTTTGATAAAATCCTACT 12000

Lser_LAC005780 11951 TCTACCAGGCGGACTTGACAAATACATTTCACTTTTGATAAAATCCTACT 12000

Lvir_CGN013357 11949 TCTACCAGGCGGACTTGACAAATACATTTCACTTTTGATAAAATCCTACT 11998

Lsal_LAC008020 11948 TCTACCAGGCGGACTTGACAAATACATTTCACTTTTGATAAAATCCTACT 11997

Lsat_Salinas 12001 CCTATGGTCTTGGTATACCCCTAGAGGGACTTGAACCCTCGTTTTCTCCG 12050

Lsat_WENDEL 12001 CCTATGGTCTTGGTATACCCCTAGAGGGACTTGAACCCTCGTTTTCTCCG 12050

Lser_US96UC23 12001 CCTATGGTCTTGGTATACCCCTAGAGGGACTTGAACCCTCGTTTTCTCCG 12050

Lser_LAC005780 12001 CCTATGGTCTTGGTATACCCCTAGAGGGACTTGAACCCTCGTTTTCTCCG 12050

Lvir_CGN013357 11999 CCTATGGTCTTGGTATACCCCTAGAGGGACTTGAACCCTCGTTTTCTCCG 12048

Lsal_LAC008020 11998 CCTATGGTCTTGGTATACCCCTAGAGGGACTTGAACCCTCGTTTTCTCCG 12047

Lsat_Salinas 12051 TGAAAGAGAGAGGTCGTAACCACTGGACCATAGGGGCACCAATGGACCAT 12100

Lsat_WENDEL 12051 TGAAAGAGAGAGGTCGTAACCACTGGACCATAGGGGCACCAATGGACCAT 12100

Lser_US96UC23 12051 TGAAAGAGAGAGGTCGTAACCACTGGACCATAGGGGCACCAATGGACCAT 12100

Lser_LAC005780 12051 TGAAAGAGAGAGGTCGTAACCACTGGACCATAGGGGCACCAATGGACCAT 12100

Lvir_CGN013357 12049 TGAAAGAGAGAGGTCGTAACCACTGGACCATAGGGGCACCAATGGACCAT 12098

Lsal_LAC008020 12048 TGAAAGAGAGAGGTCGTAACCACTGGACCATAGGGGCACCAATGGACCAT 12097

Lsat_Salinas 12101 AGGGGCCTATGGCCACCTTTAGGAAATCAAAAAGCACTACACAATACAAA 12150

Lsat_WENDEL 12101 AGGGGCCTATGGCCACCTTTAGGAAATCAAAAAGCACTACACAATACAAA 12150

Lser_US96UC23 12101 AGGGGCCTATGGCCACCTTTAGGAAATCAAAAAGCACTACACAATACAAA 12150

Lser_LAC005780 12101 AGGGGCCTATGGCCACCTTTAGGAAATCAAAAAGCACTACACAATACAAA 12150

Lvir_CGN013357 12099 AGGGGCCTATGGCCACCTTTAGGAAATCAAAAAGCACTACACAATACAAA 12148

Lsal_LAC008020 12098 AGGGGCCTATGGCCACCTTTAGGAAATCAAAAAGCACTACACAATACAAA 12147

Lsat_Salinas 12151 TACAATAGGGAATAAGGCCTAAGGCCACCTTAACTTAAATATAAATCAGC 12200

Lsat_WENDEL 12151 TACAATAGGGAATAAGGCCTAAGGCCACCTTAACTTAAATATAAATCAGC 12200

Lser_US96UC23 12151 TACAATAGGGAATAAGGCCTAAGGCCACCTTAACTTAAATATAAATCAGC 12200

Lser_LAC005780 12151 TACAATAGGGAATAAGGCCTAAGGCCACCTTAACTTAAATATAAATCAGC 12200

Lvir_CGN013357 12149 TACAATAGGGAATAAGGCCTAAGGCCACCTTAACTTAAATATAAATCAGC 12198

Lsal_LAC008020 12148 TACAATAGGGAATAAGGCCTAAGGCCACCTTAACTTAAATATAAATCAGC 12197

Lsat_Salinas 12201 CGAGGGACACCTTTAGAAGATGAATAGGATATGAATCAAACCGAAAAACT 12250

Lsat_WENDEL 12201 CGAGGGACACCTTTAGAAGATGAATAGGATATGAATCAAACCGAAAAACT 12250

Lser_US96UC23 12201 CGAGGGACACCTTTAGAAGATGAATAGGATATGAATCAAACCGAAAAACT 12250

Lser_LAC005780 12201 CGAGGGACACCTTTAGAAGATGAATAGGATATGAATCAAACCGAAAAACT 12250

Lvir_CGN013357 12199 CGAGGGACACCTTTAGAAGATGAATAGGATATGAATCAAACCGAAAAACT 12248

Lsal_LAC008020 12198 CGAGGGACACCTTTAGAAGATGAATAGGATATGAATCAAACCGAAAAACT 12247

Lsat_Salinas 12251 CGTTAACTTTTCTGGGGGTTAACGGTAATCAAACTTTACCATTAAACTAT 12300

Lsat_WENDEL 12251 CGTTAACTTTTCTGGGGGTTAACGGTAATCAAACTTTACCATTAAACTAT 12300

Lser_US96UC23 12251 CGTTAACTTTTCTGGGGGTTAACGGTAATCAAACTTTACCATTAAACTAT 12300

Lser_LAC005780 12251 CGTTAACTTTTCTGGGGGTTAATGGTAATCAAACTTTACCATTAAACTAT 12300

Lvir_CGN013357 12249 CATTAACTTTTCTGGGGGTTAATGGTAATCAAACTTTACCATTAAACTAT 12298

Lsal_LAC008020 12248 CGTTAACTTTTCTGGGGGTTAATGGTAATCAAACTTTACCATTAAACTAT 12297

Lsat_Salinas 12301 ACAATCTTTCAACTTTATTTCATTGGTTTTTCTCGTCTTTATCTCTCTCA 12350

Lsat_WENDEL 12301 ACAATCTTTCAACTTTATTTCATTGGTTTTTCTCGTCTTTATCTCTCTCA 12350

Lser_US96UC23 12301 ACAATCTTTCAACTTTATTTCATTGGTTTTTCTCGTCTTTATCTCTCTCA 12350

Lser_LAC005780 12301 ACAATCTTTCAACTTTATTTCATTGGTTTTTCTCGTCTTTATCTCTCTCA 12350

Lvir_CGN013357 12299 ACAATCTTTCAACTTTATTTCATTGGTTTTTCTCGTCTTTATCTCTCTCA 12348

Lsal_LAC008020 12298 ACAATCTTTCAACTTTATTTCATTGGTTTTTCTCGTCTTTATCTCTCTCA 12347

Lsat_Salinas 12351 TTCAGAAAGAGTTTTGCATTGGATCCAAGATACGGTACCTCTGAATCAGC 12400

Lsat_WENDEL 12351 TTCAGAAAGAGTTTTGCATTGGATCCAAGATACGGTACCTCTGAATCAGC 12400

Lser_US96UC23 12351 TTCAGAAAGAGTTTTGCATTGGATCCAAGATACGGTACCTCTGAATCAGC 12400

Lser_LAC005780 12351 TTCAGAAAGAGTTTTGCATTGGATCCAAGATACGGTACCTCTGAATCAGC 12400

Lvir_CGN013357 12349 TTCAGAAAGAGTTTTGCATTGGATCCAAGATACGGTACCTCTGAATCAGC 12398

Lsal_LAC008020 12348 TTCAGAAAGAGTTTTGCATTGGATCCAAGATACGGTACCTCTGAATCAGC 12397

Lsat_Salinas 12401 AGAGCAGGAGATGCAAAAAAAAATGATTTACCAAAGTCTGATTTGGGAAT 12450

Lsat_WENDEL 12401 AGAGCAGGAGATGCAAAAAAAAATGATTTACCAAAGTCTGATTTGGGAAT 12450

Lser_US96UC23 12401 AGAGCAGGAGATGCAAAAAAAAATGATTTACCAAAGTCTGATTTGGGAAT 12450

Lser_LAC005780 12401 AGAGCAGGAGATGCAAAAAAAAATGATTTACCAAAGTCTGATTTGGGAAT 12450

Lvir_CGN013357 12399 AGAGCAGGAGATGCAAAAAAAAATCATTTACCAAAGTCTGATTTGGGAAT 12448

Lsal_LAC008020 12398 AGAGCAGGAGATGCAAAAAAAAATGATTTACCAAAGTCTGATTTGGGAAT 12447

Lsat_Salinas 12451 TTTATTGAGCCCTAAATCATATCAAAGCCATATCAAATTATATATATATA 12500

Lsat_WENDEL 12451 TTTATTGAGCCCTAAATCATATCAAAGCCATATCAAATTATATATATATA 12500

Lser_US96UC23 12451 TTTATTGAGCCCTAAATCATATCAAAGCCATATCAAATTATATATATATA 12500

Lser_LAC005780 12451 TTTATTGAGCCCTAAATCATATCAAAGCCATATCAAATTATATATATATA 12500

Lvir_CGN013357 12449 TTTATTGAGCCCTAAATCATATCAAAGTCATATCAAATTATATATATATA 12498

Lsal_LAC008020 12448 TTTATTGAGCCCTAAATCATATCAAAGTCATATCAAATTATATATATATA 12497

Lsat_Salinas 12501 TAAATAATACTGTCAACTGTCAATTGACGACAGGAGGGCAATAAAAAATA 12550

Lsat_WENDEL 12501 TAAATAATACTGTCAACTGTCAATTGACGACAGGAGGGCAATAAAAAATA 12550

Lser_US96UC23 12501 TAAATAATACTGTCAACTGTCAATTGACGACAGGAGGGCAATAAAAAATA 12550

Lser_LAC005780 12501 TAAATAATACTGTCAACTGTCAATTGACGACAGGAGGGCAATAAAAAATA 12550

Lvir_CGN013357 12499 TAAATAATACTGTCAACTGTCAATTGACGACAGGAGGGCAATAAAAAATA 12548

Lsal_LAC008020 12498 TAAATAATACTGTCAACTGTCAATTGACGACAGGAGGGCAATAAAAAATA 12547

Lsat_Salinas 12551 AAAGAAGAGAAAAGACATTAGGTATATCCGCCGGGTTCATCAATACAACA 12600

Lsat_WENDEL 12551 AAAGAAGAGAAAAGACATTAGGTATATCCGCCGGGTTCATCAATACAACA 12600

Lser_US96UC23 12551 AAAGAAGAGAAAAGACATTAGGTATATCCGCCGGGTTCATCAATACAACA 12600

Lser_LAC005780 12551 AAAGAAGAGAAAAGACATTAGGTATATCCGCCGGGTTCATCAATACAACA 12600

Lvir_CGN013357 12549 AAAGAAGAGAAAAGACATTAGGTATATCCGCCGGGTTCATCAATACAACA 12598

Lsal_LAC008020 12548 AAAGAAGAGAAAAGACATTAGGTATATCCGCTGGGTTCATCAATACAACA 12597

Lsat_Salinas 12601 TTCCTTTAGTTTTATGGTATTAAATATTCAAGTAGATTAGAATATCAATA 12650

Lsat_WENDEL 12601 TTCCTTTAGTTTTATGGTATTAAATATTCAAGTAGATTAGAATATCAATA 12650

Lser_US96UC23 12601 TTCCTTTAGTTTTATGGTATTAAATATTCAAGTAGATTAGAATATCAATA 12650

Lser_LAC005780 12601 TTCCTTTAGTTTTATGGTATTAAATATTCAAGTAGATTAGAATATCAATA 12650

Lvir_CGN013357 12599 TTCCTTTAGTTTTATGGTATTAAATATTCAAGTAGATTAGAATATCAATA 12648

Lsal_LAC008020 12598 TTCCTTTAGTTTTATGGTATTAAATATTCAAGTAGATTAGAATATCAATA 12647

Lsat_Salinas 12651 CCGTTTTACATTTTACATTATAATATAAGAAACTGACTCAGTTTTTATAT 12700

Lsat_WENDEL 12651 CCGTTTTACATTTTACATTATAATATAAGAAACTGACTCAGTTTTTATAT 12700

Lser_US96UC23 12651 CCGTTTTACATTTTACATTATAATATAAGAAACTGACTCAGTTTTTATAT 12700

Lser_LAC005780 12651 CCGTTTTACATTTTACATTATAATATAAGAAACTGACTCAGTTTTTATAT 12700

Lvir_CGN013357 12649 CCGTTTTACATTTTACATTATAATATAAGAAACTGACTCAGTTTTTATAT 12698

Lsal_LAC008020 12648 CCGTTTTACATTTTACATTATAATATAAGAAACTGACGCAGTTTTTATAT 12697

Lsat_Salinas 12701 TATAAAAAAAATCCCAAAGCATAGTAAGACTAAGCAGGAGAATTCTGTTT 12750

Lsat_WENDEL 12701 TATAAAAAAAATCCCAAAGCATAGTAAGACTAAGCAGGAGAATTCTGTTT 12750

Lser_US96UC23 12701 TATAAAAAAAATCCCAAAGCATAGTAAGACTAAGCAGGAGAATTCTGTTT 12750

Lser_LAC005780 12701 TATAAAAAAAATCCCAAAGCATAGTAAGACTAAGCAGGAGAATTCTGTTT 12750

Lvir_CGN013357 12699 TATAAAAAAAATCCCAAAGCATAGTAAGACTAAGCAGGAGAATTCTGTTT 12748

Lsal_LAC008020 12698 TATAAAAAAAATCCCAAAGCATAGTAAGACTAAGCAGGAGAATTCTGTTT 12747

Lsat_Salinas 12751 CTAGGACTGTTTTCTCAATTCCGTTCCATTTGCATCTCTTAAAAATGACA 12800

Lsat_WENDEL 12751 CTAGGACTGTTTTCTCAATTCCGTTCCATTTGCATCTCTTAAAAATGACA 12800

Lser_US96UC23 12751 CTAGGACTGTTTTCTCAATTCCGTTCCATTTGCATCTCTTAAAAATGACA 12800

Lser_LAC005780 12751 CTAGGACTGTTTTCTCAATTCCGTTCCATTTGCATCTCTTAAAAATGACA 12800

Lvir_CGN013357 12749 CTAGGACTGTTTTCTCAATTCCGTTCCATTTGCATCTCTTAAAAATGACA 12798

Lsal_LAC008020 12748 CTAGGACTGTTTTCTCAATTCCGTTCCATTTGCATCTCTTAAAAATGACA 12797

Lsat_Salinas 12801 ATTTAAATGAAATTGAAATTAAGTATAAATTTGAATTCCACCTATCTCAT 12850

Lsat_WENDEL 12801 ATTTAAATGAAATTGAAATTAAGTATAAATTTGAATTCCACCTATCTCAT 12850

Lser_US96UC23 12801 ATTTAAATGAAATTGAAATTAAGTATAAATTTGAATTCCACCTATCTCAT 12850

Lser_LAC005780 12801 ATTTAAATGAAATTGAAATTAAGTATAAATTTGAATTCCACCTATCTCAT 12850

Lvir_CGN013357 12799 ATTTAAATTAAATTGAAATTAAGTATAAATTTGAATTCCACCTATCTCAT 12848

Lsal_LAC008020 12798 ATTTAAATTAAATTGAAATTAAGTATAAATTTGAATTCCACCTATCTCAT 12847

Lsat_Salinas 12851 AGATTCCAGTCAAAGATTCATGGAATCGAAATTCCATCATTGCTATAGGA 12900

Lsat_WENDEL 12851 AGATTCCAGTCAAAGATTCATGGAATCGAAATTCCATCATTGCTATAGGA 12900

Lser_US96UC23 12851 AGATTCCAGTCAAAGATTCATGGAATCGAAATTCCATCATTGCTATAGGA 12900

Lser_LAC005780 12851 AGATTCCAGTCAAAGATTCATGGAATCGAAATTCCATCATTGCTATAGGA 12900

Lvir_CGN013357 12849 AGATTCCAGTCAAAGATTCATGGAATCGAAATTCCATCATTGCTATAGGA 12898

Lsal_LAC008020 12848 AGATTCCAGTCAAAGATTCATGGAATCGAAATTCCATCATTGCTATAGGA 12897

Lsat_Salinas 12901 TTTGATGGATAATGAGCCAGCCAAAATGGTATTTCTTTTTTTTTTTTTTG 12950

Lsat_WENDEL 12901 TTTGATGGATAATGAGCCAGCCAAAATGGTATTTCTTTTTTTTTTTTTTG 12950

Lser_US96UC23 12901 TTTGATGGATAATGAGCCAGCCAAAATGGTATTTCTTTTTTTTTTTTTTG 12950

Lser_LAC005780 12901 TTTGATGGATAATGAGCCAGCCAAAATGGTATTTCTTTTTTTTTTTTTTG 12950

Lvir_CGN013357 12899 TTTGATGGATAATGAGCCAGCCAAAATGGTATTTCTTTTTTTTTTTTTTG 12948

Lsal_LAC008020 12898 TTTGATGGATAATGAGCCAGCCAAAATGGTATTTCTTTTTTTTTTTTTTG 12947

Lsat_Salinas 12951 TTTTTGGAGAATTCGAGATGGATGAATATAAATAACTGACAATTTCAAAA 13000

Lsat_WENDEL 12951 TTTTTGGAGAATTCGAGATGGATGAATATAAATAACTGACAATTTCAAAA 13000

Lser_US96UC23 12951 TTTTTGGAGAATTCGAGATGGATGAATATAAATAACTGACAATTTCAAAA 13000

Lser_LAC005780 12951 TTTTTGGAGAATTCGAGATGGATGAATATAAATAACTGACAATTTCAAAA 13000

Lvir_CGN013357 12949 TTTTTGGAGAATTCGAGATGGATGAATATAAATAACTGACAATTTCAAAA 12998

Lsal_LAC008020 12948 TTTTTGGAGAATTCGAGATGGATGAATATAAATAACTGACAATTTCAAAA 12997

Lsat_Salinas 13001 TAATCCGGGATAGACGCAATGTCCACAATACCTGGATTTAATCAGATACA 13050

Lsat_WENDEL 13001 TAATCCGGGATAGACGCAATGTCCACAATACCTGGATTTAATCAGATACA 13050

Lser_US96UC23 13001 TAATCCGGGATAGACGCAATGTCCACAATACCTGGATTTAATCAGATACA 13050

Lser_LAC005780 13001 TAATCCGGGATAGACGCAATGTCCACAATACCTGGATTTAATCAGATACA 13050

Lvir_CGN013357 12999 TAATCCGGGATAGACGCAATGTCCACAATACCTGGATTTAATCAGATACA 13048

Lsal_LAC008020 12998 TAATCCGGGATAGACGCAATGTCCACAATACCTGGATTTAATCAGATACA 13047

Lsat_Salinas 13051 ATTTGAAGGATTTTGTAGGTTCATTGATCAGGGTTTGACAGAAGAACTTT 13100

Lsat_WENDEL 13051 ATTTGAAGGATTTTGTAGGTTCATTGATCAGGGTTTGACAGAAGAACTTT 13100

Lser_US96UC23 13051 ATTTGAAGGATTTTGTAGGTTCATTGATCAGGGTTTGACAGAAGAACTTT 13100

Lser_LAC005780 13051 ATTTGAAGGATTTTGTAGGTTCATTGATCAGGGTTTGACAGAAGAACTTT 13100

Lvir_CGN013357 13049 ATTTGAAGGATTTTGTAGGTTCATTGATCAGGGTTTGACAGAAGAACTTT 13098

Lsal_LAC008020 13048 ATTTGAAGGATTTTGTAGGTTCATTGATCAGGGTTTGACAGAAGAACTTT 13097

Lsat_Salinas 13101 CTAAGTTTCCAAAAATTGAAGATACAAATCAAGAAATTGACTTTGAATTA 13150

Lsat_WENDEL 13101 CTAAGTTTCCAAAAATTGAAGATACAAATCAAGAAATTGACTTTGAATTA 13150

Lser_US96UC23 13101 CTAAGTTTCCAAAAATTGAAGATACAAATCAAGAAATTGACTTTGAATTA 13150

Lser_LAC005780 13101 CTAAGTTTCCAAAAATTGAAGATACAAATCAAGAAATTGACTTTGAATTA 13150

Lvir_CGN013357 13099 CTAAGTTTCCAAAAATTGAAGATACAAATCAAGAAATTGACTTTGAATTA 13148

Lsal_LAC008020 13098 CTAAGTTTCCAAAAATTGAAGATACAAATCAAGAAATTGACTTTGAATTA 13147

Lsat_Salinas 13151 TTTTTGGAAAGATATCAATTGGTAGAACCCTCGATAAAGGAAAGAGATGC 13200

Lsat_WENDEL 13151 TTTTTGGAAAGATATCAATTGGTAGAACCCTCGATAAAGGAAAGAGATGC 13200

Lser_US96UC23 13151 TTTTTGGAAAGATATCAATTGGTAGAACCCTCGATAAAGGAAAGAGATGC 13200

Lser_LAC005780 13151 TTTTTGGAAAGATATCAATTGGTAGAACCCTCGATAAAGGAAAGAGATGC 13200

Lvir_CGN013357 13149 TTTTTGGAAAGATATCAATTGGTAGAACCCTCGATAAAGGAAAGAAATGC 13198

Lsal_LAC008020 13148 TTTTTGGAAAGATATCAATTGGTAGAACCCTCGATAAAGGAAAGAGATGC 13197

Lsat_Salinas 13201 TGTGTATGAATCACTCACATATTCTTCTGAATTATATGTATCTGCGAGAC 13250

Lsat_WENDEL 13201 TGTGTATGAATCACTCACATATTCTTCTGAATTATATGTATCTGCGAGAC 13250

Lser_US96UC23 13201 TGTGTATGAATCACTCACATATTCTTCTGAATTATATGTATCTGCGAGAC 13250

Lser_LAC005780 13201 TGTGTATGAATCACTCACATATTCTTCTGAATTATATGTATCTGCGAGAC 13250

Lvir_CGN013357 13199 TGTGTATGAATCACTCACATATTCTTCTGAATTATATGTATCTGCGAGAC 13248

Lsal_LAC008020 13198 TGTGTATGAATCACTCACATATTCTTCTGAATTATATGTATCTGCGAGAC 13247

Lsat_Salinas 13251 TAATTTGGAAAAACGATAGGCGTAGGTATATCCAAGAACAAACAATTTTG 13300

Lsat_WENDEL 13251 TAATTTGGAAAAACGATAGGCGTAGGTATATCCAAGAACAAACAATTTTG 13300

Lser_US96UC23 13251 TAATTTGGAAAAACGATAGGCGTAGGTATATCCAAGAACAAACAATTTTG 13300

Lser_LAC005780 13251 TAATTTGGAAAAACGATAGGCGTAGGTATATCCAAGAACAAACAATTTTG 13300

Lvir_CGN013357 13249 TAATTTGGAAAAACGATAGGCGTAGGTATATCCAAGAACAAACAATTTTG 13298

Lsal_LAC008020 13248 TAATTTGGAAAAACGATAGGCGTAGGTATATCCAAGAACAAACAATTTTG 13297

Lsat_Salinas 13301 ATAGGAAAGATCCCTCTAATGACTTCTCTGGGAGCTTTTATAGTAAATGG 13350

Lsat_WENDEL 13301 ATAGGAAAGATCCCTCTAATGACTTCTCTGGGAGCTTTTATAGTAAATGG 13350

Lser_US96UC23 13301 ATAGGAAAGATCCCTCTAATGACTTCTCTGGGAGCTTTTATAGTAAATGG 13350

Lser_LAC005780 13301 ATAGGAAAGATCCCTCTAATGACTTCTCTGGGAGCTTTTATAGTAAATGG 13350

Lvir_CGN013357 13299 ATAGGAAAGATCCCTCTAATGACTTCTCTGGGAGCTTTTATAGTAAATGG 13348

Lsal_LAC008020 13298 ATAGGAAAGATCCCTCTAATGACTTCTCTGGGAGCTTTTATAGTAAATGG 13347

Lsat_Salinas 13351 AATATATAGAATTGTGATCAATCAAATATTGCAAAGTCCCGGTATTTATT 13400

Lsat_WENDEL 13351 AATATATAGAATTGTGATCAATCAAATATTGCAAAGTCCCGGTATTTATT 13400

Lser_US96UC23 13351 AATATATAGAATTGTGATCAATCAAATATTGCAAAGTCCCGGTATTTATT 13400

Lser_LAC005780 13351 AATATATAGAATTGTGATCAATCAAATATTGCAAAGTCCCGGTATTTATT 13400

Lvir_CGN013357 13349 AATATATAGAATTGTGATCAATCAAATATTGCAAAGTCCCGGTATTTATT 13398

Lsal_LAC008020 13348 AATATATAGAATTGTGATCAATCAAATATTGCAAAGTCCCGGTATTTATT 13397

Lsat_Salinas 13401 ACCAGTCAGAATTGAACGATAATGGAATTTCCGTCTATACCGGCACCATA 13450

Lsat_WENDEL 13401 ACCAGTCAGAATTGAACGATAATGGAATTTCCGTCTATACCGGCACCATA 13450

Lser_US96UC23 13401 ACCAGTCAGAATTGAACGATAATGGAATTTCCGTCTATACCGGCACCATA 13450

Lser_LAC005780 13401 ACCAGTCAGAATTGAACGATAATGGAATTTCCGTCTATACCGGCACCATA 13450

Lvir_CGN013357 13399 ACCAGTCAGAATTGAACGATAATGGAATTTCCGTCTATACCGGCACCATA 13448

Lsal_LAC008020 13398 ACCAGTCAGAATTGAACGATAATGGAATTTCCGTCTATACCGGCACCATA 13447

Lsat_Salinas 13451 ATATCAGATTGGGGAGGAAGATTAGAATTAGAGATTGATAGAAAAACAAG 13500

Lsat_WENDEL 13451 ATATCAGATTGGGGAGGAAGATTAGAATTAGAGATTGATAGAAAAACAAG 13500

Lser_US96UC23 13451 ATATCAGATTGGGGAGGAAGATTAGAATTAGAGATTGATAGAAAAACAAG 13500

Lser_LAC005780 13451 ATATCAGATTGGGGAGGAAGATTAGAATTAGAGATTGATAGAAAAACAAG 13500

Lvir_CGN013357 13449 ATATCAGATTGGGGAGGAAGATTAGAATTAGAGATTGATAGAAAAACAAG 13498

Lsal_LAC008020 13448 ATATCAGATTGGGGAGGAAGATTAGAATTAGAGATTGATAGAAAAACAAG 13497

Lsat_Salinas 13501 GATATGGGTTCGCGTGAGTAGGCAACAAAAACTATCTATTCTAGTTCTAT 13550

Lsat_WENDEL 13501 GATATGGGTTCGCGTGAGTAGGCAACAAAAACTATCTATTCTAGTTCTAT 13550

Lser_US96UC23 13501 GATATGGGTTCGCGTGAGTAGGCAACAAAAACTATCTATTCTAGTTCTAT 13550

Lser_LAC005780 13501 GATATGGGTTCGCGTGAGTAGGCAACAAAAACTATCTATTCTAGTTCTAT 13550

Lvir_CGN013357 13499 GATATGGGTTCGCGTGAGTAGGCAACAAAAACTATCTATTCTAGTTCTAT 13548

Lsal_LAC008020 13498 GATATGGGTTCGCGTGAGTAGGCAACAAAAACTATCTATTCTAGTTCTAT 13547

Lsat_Salinas 13551 TATCAGCTATGGGGTTGAATATAAGAGAAATTCTAGAGAATGTTTGCTAT 13600

Lsat_WENDEL 13551 TATCAGCTATGGGGTTGAATATAAGAGAAATTCTAGAGAATGTTTGCTAT 13600

Lser_US96UC23 13551 TATCAGCTATGGGGTTGAATATAAGAGAAATTCTAGAGAATGTTTGCTAT 13600

Lser_LAC005780 13551 TATCAGCTATGGGGTTGAATATAAGAGAAATTCTAGAGAATGTTTGCTAT 13600

Lvir_CGN013357 13549 TATCAGCTATGGGGTTGAATATAAGAGAAATTCTAGAGAATGTTTGCTAT 13598

Lsal_LAC008020 13548 TATCAGCTATGGGGTTGAATATAAGAGAAATTCTAGAGAATGTTTGCTAT 13597

Lsat_Salinas 13601 CCTGAACTATTTTTGTCTTTTCTGAATGATAAAAAACAAATAGGGTCAAA 13650

Lsat_WENDEL 13601 CCTGAACTATTTTTGTCTTTTCTGAATGATAAAAAACAAATAGGGTCAAA 13650

Lser_US96UC23 13601 CCTGAACTATTTTTGTCTTTTCTGAATGATAAAAAACAAATAGGGTCAAA 13650

Lser_LAC005780 13601 CCTGAACTATTTTTGTCTTTTCTGAATGATAAAAAACAAATAGGGTCAAA 13650

Lvir_CGN013357 13599 CCTGAACTATTTTTGTCTTTTCTGAATGATAAAAAACAAATAGGGTCAAA 13648

Lsal_LAC008020 13598 CCTGAACTATTTTTGTCTTTTCTGAATGATAAAAAACAAATAGGGTCAAA 13647

Lsat_Salinas 13651 AGAAAATGCTATTTTGGAGTTTTATCAACAATTTGCTTGTGTAGAGGGAG 13700

Lsat_WENDEL 13651 AGAAAATGCTATTTTGGAGTTTTATCAACAATTTGCTTGTGTAGAGGGAG 13700

Lser_US96UC23 13651 AGAAAATGCTATTTTGGAGTTTTATCAACAATTTGCTTGTGTAGAGGGAG 13700

Lser_LAC005780 13651 AGAAAATGCTATTTTGGAGTTTTATCAACAATTTGCTTGTGTAGAGGGAG 13700

Lvir_CGN013357 13649 AGAAAATGCTATTTTGGAGTTTTATCAACAATTTGCTTGTGTAGAGGGAG 13698

Lsal_LAC008020 13648 AGAAAATGCTATTTTGGAGTTTTATCAACAATTTGCTTGTGTAGAGGGAG 13697

Lsat_Salinas 13701 ATCCGGTATTTTCTGAATCCTTATCTAAGGATTTACAAAAAAAATTCTTT 13750

Lsat_WENDEL 13701 ATCCGGTATTTTCTGAATCCTTATCTAAGGATTTACAAAAAAAATTCTTT 13750

Lser_US96UC23 13701 ATCCGGTATTTTCTGAATCCTTATCTAAGGATTTACAAAAAAAATTCTTT 13750

Lser_LAC005780 13701 ATCCGGTATTTTCTGAATCCTTATCTAAGGATTTACAAAAAAAATTCTTT 13750

Lvir_CGN013357 13699 ATCCGGTATTTTCTGAATCCTTATCTAAGGATTTACAAAAAAAATTCTTT 13748

Lsal_LAC008020 13698 ATCCGGTATTTTCTGAATCCTTATCTAAGGATTTACAAAAAAAATTCTTT 13747

Lsat_Salinas 13751 CAACAAAGATGTGAATTGGGAGGGATTGGTCGACGAAATATGAATCGGAG 13800

Lsat_WENDEL 13751 CAACAAAGATGTGAATTGGGAGGGATTGGTCGACGAAATATGAATCGGAG 13800

Lser_US96UC23 13751 CAACAAAGATGTGAATTGGGAGGGATTGGTCGACGAAATATGAATCGGAG 13800

Lser_LAC005780 13751 CAACAAAGATGTGAATTGGGAGGGATTGGTCGACGAAATATGAATCGGAG 13800

Lvir_CGN013357 13749 CAACAAAGATGTGAATTGGGAGGGATTGGTCGACGAAATATGAATCGGAG 13798

Lsal_LAC008020 13748 CAACAAAGATGTGAATTGGGAGGGATTGGTCGACGAAATATGAATCGGAG 13797

Lsat_Salinas 13801 ACTGAACCTTGATATACCCCAGAACAATACATTTTTGTTACCGCGAGATA 13850

Lsat_WENDEL 13801 ACTGAACCTTGATATACCCCAGAACAATACATTTTTGTTACCGCGAGATA 13850

Lser_US96UC23 13801 ACTGAACCTTGATATACCCCAGAACAATACATTTTTGTTACCGCGAGATA 13850

Lser_LAC005780 13801 ACTGAACCTTGATATACCCCAGAACAATACATTTTTGTTACCGCGAGATA 13850

Lvir_CGN013357 13799 ACTGAACCTTGATATACCCCAGAACAATACATTTTTGTTACCGCGAGATA 13848

Lsal_LAC008020 13798 ACTGAACCTTGATATACCCCAGAACAATACATTTTTGTTACCGCGAGATA 13847

Lsat_Salinas 13851 TATTGGCAGCCGCGGATCGTTTGATTCGAATCAAATTTGGAATGGGTACA 13900

Lsat_WENDEL 13851 TATTGGCAGCCGCGGATCGTTTGATTCGAATCAAATTTGGAATGGGTACA 13900

Lser_US96UC23 13851 TATTGGCAGCCGCGGATCGTTTGATTCGAATCAAATTTGGAATGGGTACA 13900

Lser_LAC005780 13851 TATTGGCAGCCGCGGATCGTTTGATTCGAATCAAATTTGGAATGGGTACA 13900

Lvir_CGN013357 13849 TATTGGCAGCCGCGGATCGTTTGATTCGAATCAAATTTGGAATGGGTACA 13898

Lsal_LAC008020 13848 TATTGGCAGCCGCGGATCGTTTGATTCGAATCAAATTTGGAATGGGTACA 13897

Lsat_Salinas 13901 CTTGACGATATGAATCATTTGCAAAATAAACGTATTCGTTCTGTAGCAGA 13950

Lsat_WENDEL 13901 CTTGACGATATGAATCATTTGCAAAATAAACGTATTCGTTCTGTAGCAGA 13950

Lser_US96UC23 13901 CTTGACGATATGAATCATTTGCAAAATAAACGTATTCGTTCTGTAGCAGA 13950

Lser_LAC005780 13901 CTTGACGATATGAATCATTTGCAAAATAAACGTATTCGTTCTGTAGCAGA 13950

Lvir_CGN013357 13899 CTTGACGATATGAATCATTTGCAAAATAAACGTATTCGTTCTGTAGCAGA 13948

Lsal_LAC008020 13898 CTTGACGATATGAATCATTTGCAAAATAAACGTATTCGTTCTGTAGCAGA 13947

Lsat_Salinas 13951 TCTTTTACAAGAGCAATTTGGATTGGCCTTGGTTCGTTTAGAAAATATGG 14000

Lsat_WENDEL 13951 TCTTTTACAAGAGCAATTTGGATTGGCCTTGGTTCGTTTAGAAAATATGG 14000

Lser_US96UC23 13951 TCTTTTACAAGAGCAATTTGGATTGGCCTTGGTTCGTTTAGAAAATATGG 14000

Lser_LAC005780 13951 TCTTTTACAAGAGCAATTTGGATTGGCCTTGGTTCGTTTAGAAAATATGG 14000

Lvir_CGN013357 13949 TCTTTTACAAGAGCAATTTGGATTGGCCTTGGTTCGTTTAGAAAATATGG 13998

Lsal_LAC008020 13948 TCTTTTACAAGAGCAATTTGGATTGGCCTTGGTTCGTTTAGAAAATATGG 13997

Lsat_Salinas 14001 CTCGAGGAAACATATATGCAGCACTTAAGCATAACTGGACACCAACTCCT 14050

Lsat_WENDEL 14001 CTCGAGGAAACATATATGCAGCACTTAAGCATAACTGGACACCAACTCCT 14050

Lser_US96UC23 14001 CTCGAGGAAACATATATGCAGCACTTAAGCATAACTGGACACCAACTCCT 14050

Lser_LAC005780 14001 CTCGAGGAAACATATATGCAGCACTTAAGCATAACTGGACACCAACTCCT 14050

Lvir_CGN013357 13999 CTCGAGGAAACATATATGCAGCACTTAAGCATAACTGGACACCAACTCCT 14048

Lsal_LAC008020 13998 CTCGAGGAAACATATATGCAGCACTTAAGCATAACTGGACACCAACTCCT 14047

Lsat_Salinas 14051 CAGAACTTGGTAAATTCAACCCCATTAACAGATACTTATAAAGTTTTTTT 14100

Lsat_WENDEL 14051 CAGAACTTGGTAAATTCAACCCCATTAACAGATACTTATAAAGTTTTTTT 14100

Lser_US96UC23 14051 CAGAACTTGGTAAATTCAACCCCATTAACAGATACTTATAAAGTTTTTTT 14100

Lser_LAC005780 14051 CAGAACTTGGTAAATTCAACCCCATTAACAGATACTTATAAAGTTTTTTT 14100

Lvir_CGN013357 14049 CAGAACTTGGTAAATTCAACCCCATTAACAGATACTTATAAAGTTTTTTT 14098

Lsal_LAC008020 14048 CAGAACTTGGTAAATTCAACCCCATTAACAGATACTTATAAAGTTTTTTT 14097

Lsat_Salinas 14101 CCGTTTACACCCATTATCTCAAGTTTTGGATCGAACTAATCCATTGACAC 14150

Lsat_WENDEL 14101 CCGTTTACACCCATTATCTCAAGTTTTGGATCGAACTAATCCATTGACAC 14150

Lser_US96UC23 14101 CCGTTTACACCCATTATCTCAAGTTTTGGATCGAACTAATCCATTGACAC 14150

Lser_LAC005780 14101 CCGTTTACACCCATTATCTCAAGTTTTGGATCGAACTAATCCATTGACAC 14150

Lvir_CGN013357 14099 CCGTTTACACCCATTATCTCAAGTTTTGGATCGAACTAATCCATTGACAC 14148

Lsal_LAC008020 14098 CCGTTTACACCCATTATCTCAAGTTTTGGATCGAACTAATCCATTGACAC 14147

Lsat_Salinas 14151 AAATAGTTCATGGGAGAAAATTGAGTTATTTGGGCCCGGGGGGATTGACT 14200

Lsat_WENDEL 14151 AAATAGTTCATGGGAGAAAATTGAGTTATTTGGGCCCGGGGGGATTGACT 14200

Lser_US96UC23 14151 AAATAGTTCATGGGAGAAAATTGAGTTATTTGGGCCCGGGGGGATTGACT 14200

Lser_LAC005780 14151 AAATAGTTCATGGGAGAAAATTGAGTTATTTGGGCCCGGGGGGATTGACT 14200

Lvir_CGN013357 14149 AAATAGTTCATGGGAGAAAATTGAGTTATTTGGGCCCGGGGGGATTGACT 14198

Lsal_LAC008020 14148 AAATAGTTCATGGGAGAAAATTGAGTTATTTGGGCCCGGGGGGATTGACT 14197

Lsat_Salinas 14201 GCGCGAACTGCTACTTTTCCAATACGAGATATTCATCCTAGTCACTATGG 14250

Lsat_WENDEL 14201 GCGCGAACTGCTACTTTTCCAATACGAGATATTCATCCTAGTCACTATGG 14250

Lser_US96UC23 14201 GCGCGAACTGCTACTTTTCCAATACGAGATATTCATCCTAGTCACTATGG 14250

Lser_LAC005780 14201 GCGCGAACTGCTACTTTTCCAATACGAGATATTCATCCTAGTCACTATGG 14250

Lvir_CGN013357 14199 GCGCGAACTGCTACTTTTCCAATACGAGATATTCATCCTAGTCACTATGG 14248

Lsal_LAC008020 14198 GCGCGAACTGCTACTTTTCCAATACGAGATATTCATCCTAGTCACTATGG 14247

Lsat_Salinas 14251 GCGTATTTGCCCAATTGACACATCTGAAGGAATAAATGTTGGACTTATTG 14300

Lsat_WENDEL 14251 GCGTATTTGCCCAATTGACACATCTGAAGGAATAAATGTTGGACTTATTG 14300

Lser_US96UC23 14251 GCGTATTTGCCCAATTGACACATCTGAAGGAATAAATGTTGGACTTATTG 14300

Lser_LAC005780 14251 GCGTATTTGCCCAATTGACACATCTGAAGGAATAAATGTTGGACTTATTG 14300

Lvir_CGN013357 14249 GCGTATTTGCCCAATTGACACATCTGAAGGAATAAATGTTGGACTTATTG 14298

Lsal_LAC008020 14248 GCGTATTTGCCCAATTGACACATCTGAAGGAATAAATGTTGGACTTATTG 14297

Lsat_Salinas 14301 GATCCTTAGCAATTCATGCCAGGATTGGTCGTTGGGGGTCTCTAGAAAGT 14350

Lsat_WENDEL 14301 GATCCTTAGCAATTCATGCCAGGATTGGTCGTTGGGGGTCTCTAGAAAGT 14350

Lser_US96UC23 14301 GATCCTTAGCAATTCATGCCAGGATTGGTCGTTGGGGGTCTCTAGAAAGT 14350

Lser_LAC005780 14301 GATCCTTAGCAATTCATGCCAGGATTGGTCGTTGGGGGTCTCTAGAAAGT 14350

Lvir_CGN013357 14299 GATCCTTAGCAATTCATGCCAGGATTGGTCGTTGGGGGTCTCTAGAAAGT 14348

Lsal_LAC008020 14298 GATCCTTAGCAATTCATGCCAGGATTGGTCGTTGGGGGTCTCTAGAAAGT 14347

Lsat_Salinas 14351 CCGTTTTATAAAATTTCTGAGAGATCAAAAGGGGCGCGGATGCTTTATTT 14400

Lsat_WENDEL 14351 CCGTTTTATAAAATTTCTGAGAGATCAAAAGGGGCGCGGATGCTTTATTT 14400

Lser_US96UC23 14351 CCGTTTTATAAAATTTCTGAGAGATCAAAAGGGGCGCGGATGCTTTATTT 14400

Lser_LAC005780 14351 CCGTTTTATAAAATTTCTGAGAGATCAAAAGGGGCGCGGATGCTTTATTT 14400

Lvir_CGN013357 14349 CCGTTTTATAAAATTTCTGAGAGATCAAAAGGGGCGCGGATGCTTTATTT 14398

Lsal_LAC008020 14348 CCGTTTTATAAAATTTCTGAGAGATCAAAAGGGGCGCGGATGCTTTATTT 14397

Lsat_Salinas 14401 ATCACCGGGCAGAGATGAATACTATATGGTAGCGGCAGGAAATTCTTTGG 14450

Lsat_WENDEL 14401 ATCACCGGGCAGAGATGAATACTATATGGTAGCGGCAGGAAATTCTTTGG 14450

Lser_US96UC23 14401 ATCACCGGGCAGAGATGAATACTATATGGTAGCGGCAGGAAATTCTTTGG 14450

Lser_LAC005780 14401 ATCACCGGGCAGAGATGAATACTATATGGTAGCGGCAGGAAATTCTTTGG 14450

Lvir_CGN013357 14399 ATCACCGGGCAGAGATGAATACTATATGGTAGCGGCAGGAAATTCTTTGG 14448

Lsal_LAC008020 14398 ATCACCGGGCAGAGATGAATACTATATGGTAGCGGCAGGAAATTCTTTGG 14447

Lsat_Salinas 14451 CCTTGAATCAGGGTATTCAGGAAGAACAGGTTGTTCCAGCTCGATATCGT 14500

Lsat_WENDEL 14451 CCTTGAATCAGGGTATTCAGGAAGAACAGGTTGTTCCAGCTCGATATCGT 14500

Lser_US96UC23 14451 CCTTGAATCAGGGTATTCAGGAAGAACAGGTTGTTCCAGCTCGATATCGT 14500

Lser_LAC005780 14451 CCTTGAATCAGGGTATTCAGGAAGAACAGGTTGTTCCAGCTCGATATCGT 14500

Lvir_CGN013357 14449 CCTTGAATCAGGGTATTCAGGAAGAACAGGTTGTTCCAGCTCGATATCGT 14498

Lsal_LAC008020 14448 CCTTGAATCAGGGTATTCAGGAAGAACAGGTTGTTCCAGCTCGATATCGT 14497

Lsat_Salinas 14501 CAAGAATTCCTGACTATTGCATGGGAACAGGTTCATCTTCGAAGTATTTT 14550

Lsat_WENDEL 14501 CAAGAATTCCTGACTATTGCATGGGAACAGGTTCATCTTCGAAGTATTTT 14550

Lser_US96UC23 14501 CAAGAATTCCTGACTATTGCATGGGAACAGGTTCATCTTCGAAGTATTTT 14550

Lser_LAC005780 14501 CAAGAATTCCTGACTATTGCATGGGAACAGGTTCATCTTCGAAGTATTTT 14550

Lvir_CGN013357 14499 CAAGAATTCCTGACTATTGCATGGGAACAGGTTCATCTTCGAAGTATTTT 14548

Lsal_LAC008020 14498 CAAGAATTCCTGACTATTGCATGGGAACAGGTTCATCTTCGAAGTATTTT 14547

Lsat_Salinas 14551 TTCCTTCCAATATTTTTCTATTGGAGCTTCCCTCATTCCTTTTATCGAGC 14600

Lsat_WENDEL 14551 TTCCTTCCAATATTTTTCTATTGGAGCTTCCCTCATTCCTTTTATCGAGC 14600

Lser_US96UC23 14551 TTCCTTCCAATATTTTTCTATTGGAGCTTCCCTCATTCCTTTTATCGAGC 14600

Lser_LAC005780 14551 TTCCTTCCAATATTTTTCTATTKGAGCTTCCCTYWTTCCTTTTATCGAGC 14600

Lvir_CGN013357 14549 TTCCTTCCAATATTTTTCTATTGGAGCTTCCCTTATTCCTTTTATCGAGC 14598

Lsal_LAC008020 14548 TTCCTTCCAATATTTTTCTATTKGAGCTTCCCTYWTTCCTTTTATCGAGC 14597

Lsat_Salinas 14601 ATAATGATGCGAATCGGGCTTTAATGAGTTCTAACATGCAACGCCAAGCA 14650

Lsat_WENDEL 14601 ATAATGATKCGAATCGGGCTTTAATGAGTTCTAACATGCAACGCCAAGCA 14650

Lser_US96UC23 14601 ATAATGATGCGAATCGGGCTTTAATGAGTTCTAACATGCAACGCCAAGCA 14650

Lser_LAC005780 14601 ATAATGATKCGAATCGGGCTTTAATGAGTTCTAACATGCAACGCCAAGCA 14650

Lvir_CGN013357 14599 ATAATGATGCGAATCGGGCTTTAATGAGTTCTAACATGCAACGCCAAGCA 14648

Lsal_LAC008020 14598 ATAATGATKCGAATCGGGCTTTAATGAGTTCTAACATGCAACGCCAAGCA 14647

Lsat_Salinas 14651 GTCCCTCTTTCTCAGTCCGAGAAGTGCATTGTTGGAACTGGATTGGAAGG 14700

Lsat_WENDEL 14651 GTCCCTCTTTCTCAGTCCGAGAAGTGCATTGTTGGAACTGGATTGGAAGG 14700

Lser_US96UC23 14651 GTCCCTCTTTCTCAGTCCGAGAAGTGCATTGTTGGAACTGGATTGGAAGG 14700

Lser_LAC005780 14651 GTCCCTCTTTCTCAGTCCGAGAAGTGCATTGTTGGAACTGGATTGGAAGG 14700

Lvir_CGN013357 14649 GTCCCTCTTTCTCAGTCCGAGAAGTGCATTGTTGGAACTGGATTGGAAGG 14698

Lsal_LAC008020 14648 GTCCCTCTTTCTCAGTCCGAGAAGTGCATTGTTGGAACTGGATTGGAAGG 14697

Lsat_Salinas 14701 CCAAGCGGCTCTAGATTCAGGGGCTCTTGCTATAGCCGAACACGAGGGAG 14750

Lsat_WENDEL 14701 CCAAGCGGCTCTAGATTCAGGGGCTCTTGCTATAGCCGAACACGAGGGAG 14750

Lser_US96UC23 14701 CCAAGCGGCTCTAGATTCAGGGGCTCTTGCTATAGCCGAACACGAGGGAG 14750

Lser_LAC005780 14701 CCAAGCGGCTCTAGATTCAGGGGCTCTTGCTATAGCCGAACACGAGGGAG 14750

Lvir_CGN013357 14699 CCAAGCGGCTCTAGATTCAGGGGCTCTTGCTATAGCCGAACACGAGGGAG 14748

Lsal_LAC008020 14698 CCAAGCGGCTCTAGATTCAGGGGCTCTTGCTATAGCCGAACACGAGGGAG 14747

Lsat_Salinas 14751 AGATTATTTATACCGATACTGACAAGATCCTTTTATCAGGTAATGGGGAT 14800

Lsat_WENDEL 14751 AGATTATTTATACCGATACTGACAAGATCCTTTTATCAGGTAATGGGGAT 14800

Lser_US96UC23 14751 AGATTATTTATACCGATACTGACAAGATCCTTTTATCAGGTAATGGGGAT 14800

Lser_LAC005780 14751 AGATTATTTATACCGATACTGACAAGATCCTTTTATCAGGTAATGGGGAT 14800

Lvir_CGN013357 14749 AGATTATTTATACCGATACTGACAAGATCCTTTTATCAGGTAATGGGGAT 14798

Lsal_LAC008020 14748 AGATTATTTATACCGATACTGACAAGATCCTTTTATCAGGTAATGGGGAT 14797

Lsat_Salinas 14801 ACTCTAAGGATTCCATTAGTTATGTATCAACGTTCCAACAAAAATACTTG 14850

Lsat_WENDEL 14801 ACTCTAAGGATTCCATTAGTTATGTATCAACGTTCCAACAAAAATACTTG 14850

Lser_US96UC23 14801 ACTCTAAGGATTCCATTAGTTATGTATCAACGTTCCAACAAAAATACTTG 14850

Lser_LAC005780 14801 ACTCTAAGGATTCCATTAGTTATGTATCAACGTTCCAACAAAAATACTTG 14850

Lvir_CGN013357 14799 ACTCTAAGGATTCCATTAGTTATGTATCAACGTTCCAACAAAAATACTTG 14848

Lsal_LAC008020 14798 ACTCTAAGGATTCCATTAGTTATGTATCAACGTTCCAACAAAAATACTTG 14847

Lsat_Salinas 14851 TATGCATCAAAAACCCCAGGTTCAGCGGGGTAAATGCATTAAAAAGGGAC 14900

Lsat_WENDEL 14851 TATGCATCAAAAACCCCAGGTTCAGCGGGGTAAATGCATTAAAAAGGGAC 14900

Lser_US96UC23 14851 TATGCATCAAAAACCCCAGGTTCAGCGGGGTAAATGCATTAAAAAGGGAC 14900

Lser_LAC005780 14851 TATGCATCAAAAACCCCAGGTTCAGCGGGGTAAATGCATTAAAAAGGGAC 14900

Lvir_CGN013357 14849 TATGCATCAAAAACCCCAGGTTCAGCGGGGTAAATGCATTAAAAAGGGAC 14898

Lsal_LAC008020 14848 TATGCATCAAAAACCCCAGGTTCAGCGGGGTAAATGCATTAAAAAGGGAC 14897

Lsat_Salinas 14901 AAATTTTAGCGTATGGTGCTGCTACAGTTGGTGGCGAACTCGCTTTGGGG 14950

Lsat_WENDEL 14901 AAATTTTAGCGTATGGTGCTGCTACAGTTGGTGGCGAACTCGCTTTGGGG 14950

Lser_US96UC23 14901 AAATTTTAGCGTATGGTGCTGCTACAGTTGGTGGCGAACTCGCTTTGGGG 14950

Lser_LAC005780 14901 AAATTTTAGCGTATGGTGCTGCTACAGTTGGTGGCGAACTCGCTTTGGGG 14950

Lvir_CGN013357 14899 AAATTTTAGCGTATGGTGCTGCTACAGTTGGTGGCGAACTCGCTTTGGGG 14948

Lsal_LAC008020 14898 AAATTTTAGCGTATGGTGCTGCTACAGTTGGTGGCGAACTCGCTTTGGGG 14947

Lsat_Salinas 14951 AAAAACGTATTAGTAGCTTATATGCCATGGGAAGGTTACAATTTTGAAGA 15000

Lsat_WENDEL 14951 AAAAACGTATTAGTAGCTTATATGCCATGGGAAGGTTACAATTTTGAAGA 15000

Lser_US96UC23 14951 AAAAACGTATTAGTAGCTTATATGCCATGGGAAGGTTACAATTTTGAAGA 15000

Lser_LAC005780 14951 AAAAACGTATTAGTAGCTTATATGCCATGGGAAGGTTACAATTTTGAAGA 15000

Lvir_CGN013357 14949 AAAAACGTATTAGTAGCTTATATGCCATGGGAAGGTTACAATTTTGAAGA 14998

Lsal_LAC008020 14948 AAAAACGTATTAGTAGCTTATATGCCATGGGAAGGTTACAATTTTGAAGA 14997

Lsat_Salinas 15001 TGCAGTACTCATTAGTGAGCGCTTAGTATATGAAGATATTTATACTTCTT 15050

Lsat_WENDEL 15001 TGCAGTACTCATTAGTGAGCGCTTAGTATATGAAGATATTTATACTTCTT 15050

Lser_US96UC23 15001 TGCAGTACTCATTAGTGAGCGCTTAGTATATGAAGATATTTATACTTCTT 15050

Lser_LAC005780 15001 TGCAGTACTCATTAGTGAGCGCTTAGTATATGAAGATATTTATACTTCTT 15050

Lvir_CGN013357 14999 TGCAGTACTCATTAGTGAGCGCTTAGTATATGAAGATATTTATACTTCTT 15048

Lsal_LAC008020 14998 TGCAGTACTCATTAGTGAGCGCTTAGTATATGAAGATATTTATACTTCTT 15047

Lsat_Salinas 15051 TTCACATACGTAAATATGAAATTCAGATTAACCAAGGCTCCGAAAGGGTC 15100

Lsat_WENDEL 15051 TTCACATACGTAAATATGAAATTCAGATTAACCAAGGCTCCGAAAGGGTC 15100

Lser_US96UC23 15051 TTCACATACGTAAATATGAAATTCAGATTAACCAAGGCTCCGAAAGGGTC 15100

Lser_LAC005780 15051 TTCACATACGTAAATATGAAATTCAGATTAACCAAGGCTCCGAAAGGGTC 15100

Lvir_CGN013357 15049 TTCACATACGTAAATATGAAATTCAGATTAACCAAGGCTCCGAAAGGGTC 15098

Lsal_LAC008020 15048 TTCACATACGTAAATATGAAATTCAGATTAACCAAGGCTCCGAAAGAGTC 15097

Lsat_Salinas 15101 ACTAATGAAATACCACATTTAGAAGTCCATTTACTCCGAAATTTAGACAA 15150

Lsat_WENDEL 15101 ACTAATGAAATACCACATTTAGAAGTCCATTTACTCCGAAATTTAGACAA 15150

Lser_US96UC23 15101 ACTAATGAAATACCACATTTAGAAGTCCATTTACTCCGAAATTTAGACAA 15150

Lser_LAC005780 15101 ACTAATGAAATACCACATTTAGAAGTCCATTTACTCCGAAATTTAGACAA 15150

Lvir_CGN013357 15099 ACTAATGAAATACCGCATTTAGAAGTCCATTTACTCCGAAATTTAGACAA 15148

Lsal_LAC008020 15098 ACTAATGAAATACCGCATTTAGAAGTCCATTTACTCCGAAATTTAGACAA 15147

Lsat_Salinas 15151 AAATGGAATTGTAATGCTGGGATCTTGGGTGGAAACAGGTGATATTTTAG 15200

Lsat_WENDEL 15151 AAATGGAATTGTAATGCTGGGATCTTGGGTGGAAACAGGTGATATTTTAG 15200

Lser_US96UC23 15151 AAATGGAATTGTAATGCTGGGATCTTGGGTGGAAACAGGTGATATTTTAG 15200

Lser_LAC005780 15151 AAATGGAATTGTAATGCTGGGATCTTGGGTGGAAACAGGTGATATTTTAG 15200

Lvir_CGN013357 15149 AAATGGAATTGTAATGCTGGGATCTTGGGTGGAAACAGGTGATATTTTAG 15198

Lsal_LAC008020 15148 AAATGGAATTGTAATGCTGGGATCTTGGGTGGAAACAGGTGATATTTTAG 15197

Lsat_Salinas 15201 TAGGTAAATTAACGCCCCAAATGGTGAAAGAATCATCGTATGCCCCCGAA 15250

Lsat_WENDEL 15201 TAGGTAAATTAACGCCCCAAATGGTGAAAGAATCATCGTATGCCCCCGAA 15250

Lser_US96UC23 15201 TAGGTAAATTAACGCCCCAAATGGTGAAAGAATCATCGTATGCCCCCGAA 15250

Lser_LAC005780 15201 TAGGTAAATTAACGCCCCAAATGGTGAAAGAATCATCGTATGCCCCCGAA 15250

Lvir_CGN013357 15199 TAGGTAAATTAACGCCCCAAATGGTGAAAGAATCCTCGTATGCCCCCGAA 15248

Lsal_LAC008020 15198 TAGGTAAATTAACGCCCCAAATGGTGAAAGAATCATCGTATGCCCCCGAA 15247

Lsat_Salinas 15251 GATAGATTGTTACGAACCATACTTGGCATGCGGGTATATACTTCAAAAGA 15300

Lsat_WENDEL 15251 GATAGATTGTTACGAACCATACTTGGCATGCGGGTATATACTTCAAAAGA 15300

Lser_US96UC23 15251 GATAGATTGTTACGAACCATACTTGGCATGCGGGTATATACTTCAAAAGA 15300

Lser_LAC005780 15251 GATAGATTGTTACGAACCATACTTGGCATGCGGGTATATACTTCAAAAGA 15300

Lvir_CGN013357 15249 GATAGATTGTTACGAACCATACTTGGCATGCGGGTATATACTTCAAAAGA 15298

Lsal_LAC008020 15248 GATAGATTGTTACGAACCATACTTGGCATGCGGGTATATACTTCAAAAGA 15297

Lsat_Salinas 15301 AACTTGTCTAAAATTACCTATAGGAGGTAGGGGTCGGGTGATTGATGTGA 15350

Lsat_WENDEL 15301 AACTTGTCTAAAATTACCTATAGGAGGTAGGGGTCGGGTGATTGATGTGA 15350

Lser_US96UC23 15301 AACTTGTCTAAAATTACCTATAGGAGGTAGGGGTCGGGTGATTGATGTGA 15350

Lser_LAC005780 15301 AACTTGTCTAAAATTACCTATAGGAGGTAGGGGTCGGGTGATTGATGTGA 15350

Lvir_CGN013357 15299 AACTTGTCTAAAATTACCTATAGGAGGTAGGGGTCGGGTGATTGATGTGA 15348

Lsal_LAC008020 15298 AACTTGTCTAAAATTACCTATAGGAGGTAGGGGTCGGGTGATTGATGTGA 15347

Lsat_Salinas 15351 GATGGGTCCAGAGTTCTAAGACAGATGAGACAGAGAAAACAGAAAGTATT 15400

Lsat_WENDEL 15351 GATGGGTCCAGAGTTCTAAGACAGATGAGACAGAGAAAACAGAAAGTATT 15400

Lser_US96UC23 15351 GATGGGTCCAGAGTTCTAAGACAGATGAGACAGAGAAAACAGAAAGTATT 15400

Lser_LAC005780 15351 GATGGGTCCAGAGTTCTAAGACAGATGAGACAGAGAAAACAGAAAGTATT 15400

Lvir_CGN013357 15349 GATGGGTCCAGAGTTCTAAGACAGATGAGACAGAGAAAACAGAAAGTATT 15398

Lsal_LAC008020 15348 GATGGGTCCAGAGTTCTAAGACAGATGAGACAGAGAAAACAGAAAGTATT 15397

Lsat_Salinas 15401 CGTGTATATATTTTACAGAAACGTGAAATAAAAGTAGGCGATAAAGTAGC 15450

Lsat_WENDEL 15401 CGTGTATATATTTTACAGAAACGTGAAATAAAAGTAGGCGATAAAGTAGC 15450

Lser_US96UC23 15401 CGTGTATATATTTTACAGAAACGTGAAATAAAAGTAGGCGATAAAGTAGC 15450

Lser_LAC005780 15401 CGTGTATATATTTTACAGAAACGTGAAATAAAAGTAGGCGATAAAGTAGC 15450

Lvir_CGN013357 15399 CGTGTATATATTTTACAGAAACGTGAAATAAAAGTAGGCGATAAAGTAGC 15448

Lsal_LAC008020 15398 CGTGTATATATTTTACAGAAACGTGAAATAAAAGTAGGCGATAAAGTAGC 15447

Lsat_Salinas 15451 TGGAAGACATGGAAATAAGGGTATCATTTCAAAAATTTTGCCTAGACAAG 15500

Lsat_WENDEL 15451 TGGAAGACATGGAAATAAGGGTATCATTTCAAAAATTTTGCCTAGACAAG 15500

Lser_US96UC23 15451 TGGAAGACATGGAAATAAGGGTATCATTTCAAAAATTTTGCCTAGACAAG 15500

Lser_LAC005780 15451 TGGAAGACATGGAAATAAGGGTATCATTTCAAAAATTTTGCCTAGACAAG 15500

Lvir_CGN013357 15449 TGGAAGACATGGAAATAAGGGTATCATTTCAAAAATTTTGCCTAGACAAG 15498

Lsal_LAC008020 15448 TGGAAGACATGGAAATAAGGGTATCATTTCAAAAATTTTGCCTAGACAAG 15497

Lsat_Salinas 15501 ATATGCCTTATTTGCAAGATGGAAGACCTGTTGATATGGTCTTCAACCCA 15550

Lsat_WENDEL 15501 ATATGCCTTATTTGCAAGATGGAAGACCTGTTGATATGGTCTTCAACCCA 15550

Lser_US96UC23 15501 ATATGCCTTATTTGCAAGATGGAAGACCTGTTGATATGGTCTTCAACCCA 15550

Lser_LAC005780 15501 ATATGCCTTATTTGCAAGATGGAAGACCTGTTGATATGGTCTTCAACCCA 15550

Lvir_CGN013357 15499 ATATGCCTTATTTGCAAGATGGAAGACCTGTTGATATGGTCTTCAACCCA 15548

Lsal_LAC008020 15498 ATATGCCTTATTTGCAAGATGGAAGACCTGTTGATATGGTCTTCAACCCA 15547

Lsat_Salinas 15551 TTAGGAGTACCTTCCCGAATGAATGTAGGACAAATATTTGAATCTTCACT 15600

Lsat_WENDEL 15551 TTAGGAGTACCTTCCCGAATGAATGTAGGACAAATATTTGAATCTTCACT 15600

Lser_US96UC23 15551 TTAGGAGTACCTTCCCGAATGAATGTAGGACAAATATTTGAATCTTCACT 15600

Lser_LAC005780 15551 TTAGGAGTACCTTCCCGAATGAATGTAGGACAAATATTTGAATCTTCACT 15600

Lvir_CGN013357 15549 TTAGGAGTACCTTCCCGAATGAATGTAGGACAAATATTTGAATCTTCACT 15598

Lsal_LAC008020 15548 TTAGGAGTACCTTCCCGAATGAATGTAGGACAAATATTTGAATCTTCACT 15597

Lsat_Salinas 15601 CGGGTTAGCTGGGGGTTTGCTAGACAGACATTATCGAATAGCGCCTTTTG 15650

Lsat_WENDEL 15601 CGGGTTAGCTGGGGGTTTGCTAGACAGACATTATCGAATAGCGCCTTTTG 15650

Lser_US96UC23 15601 CGGGTTAGCTGGGGGTTTGCTAGACAGACATTATCGAATAGCGCCTTTTG 15650

Lser_LAC005780 15601 CGGGTTAGCTGGGGGTTTGCTAGACAGACATTATCGAATAGCGCCTTTTG 15650

Lvir_CGN013357 15599 CGGGTTAGCTGGGGGTTTGCTAGACAGACATTATCGAATAGCGCCTTTTG 15648

Lsal_LAC008020 15598 CGGGTTAGCTGGGGGTTTGCTAGACAGACATTATCGAATAGCACCTTTTG 15647

Lsat_Salinas 15651 ATGAGAGATATGAACAAGAAGCTTCGAGAAAACTGGTGTTTTCTGAATTA 15700

Lsat_WENDEL 15651 ATGAGAGATATGAACAAGAAGCTTCGAGAAAACTGGTGTTTTCTGAATTA 15700

Lser_US96UC23 15651 ATGAGAGATATGAACAAGAAGCTTCGAGAAAACTGGTGTTTTCTGAATTA 15700

Lser_LAC005780 15651 ATGAGAGATATGAACAAGAAGCTTCGAGAAAACTGGTGTTTTCTGAATTA 15700

Lvir_CGN013357 15649 ATGAGAGATATGAACAAGAAGCTTCGAGAAAACTGGTGTTTTCTGAATTA 15698

Lsal_LAC008020 15648 ATGAGAGATATGAACAAGAAGCTTCGAGAAAACTGGTGTTTTCTGAATTA 15697

Lsat_Salinas 15701 TATGAAGCCAGTAAGCAAACAGTGAATCCATGGATATTTGAACCCGAGTC 15750

Lsat_WENDEL 15701 TATGAAGCCAGTAAGCAAACAGTGAATCCATGGATATTTGAACCCGAGTC 15750

Lser_US96UC23 15701 TATGAAGCCAGTAAGCAAACAGTGAATCCATGGATATTTGAACCCGAGTC 15750

Lser_LAC005780 15701 TATGAAGCCAGTAAGCAAACAGTGAATCCATGGATATTTGAACCCGAGTC 15750

Lvir_CGN013357 15699 TATGAAGCCAGTAAGCAAACAGTGAATCCATGGATATTTGAACCCGAGTC 15748

Lsal_LAC008020 15698 TATGAAGCCAGTAAGCAAACAGTGAATCCATGGATATTTGAACCCGAGTC 15747

Lsat_Salinas 15751 TCCAGGAAAAAGCAGAATATTTGATGGAAGAACAGGGGATCCTTTTGAAC 15800

Lsat_WENDEL 15751 TCCAGGAAAAAGCAGAATATTTGATGGAAGAACAGGGGATCCTTTTGAAC 15800

Lser_US96UC23 15751 TCCAGGAAAAAGCAGAATATTTGATGGAAGAACAGGGGATCCTTTTGAAC 15800

Lser_LAC005780 15751 TCCAGGAAAAAGCAGAATATTTGATGGAAGAACAGGGGATCCTTTTGAAC 15800

Lvir_CGN013357 15749 TCCAGGAAAAAGCAGAATATTTGATGGAAGAACAGGGGATCCTTTTGAAC 15798

Lsal_LAC008020 15748 TCCAGGAAAAAGCAGAATATTTGATGGAAGAACAGGGGATCCTTTTGAAC 15797

Lsat_Salinas 15801 AACCTGTTATAATAGGAAAGCCTTATATCTTGAAATTAATTCATCAAGTT 15850

Lsat_WENDEL 15801 AACCTGTTATAATAGGAAAGCCTTATATCTTGAAATTAATTCATCAAGTT 15850

Lser_US96UC23 15801 AACCTGTTATAATAGGAAAGCCTTATATCTTGAAATTAATTCATCAAGTT 15850

Lser_LAC005780 15801 AACCTGTTATAATAGGAAAGCCTTATATCTTGAAATTAATTCATCAAGTT 15850

Lvir_CGN013357 15799 AACCTGTTATAATAGGAAAGCCTTATATCTTGAAATTAATTCATCAAGTT 15848

Lsal_LAC008020 15798 AACCTGTTATAATAGGAAAGCCTTATATCTTGAAATTAATTCATCAAGTT 15847

Lsat_Salinas 15851 GATGATAAAATCCATGGGCGTTCCAGTGGGCGTTATTCACGTCTTACACA 15900

Lsat_WENDEL 15851 GATGATAAAATCCATGGGCGTTCCAGTGGGCGTTATTCACGTCTTACACA 15900

Lser_US96UC23 15851 GATGATAAAATCCATGGGCGTTCCAGTGGGCGTTATTCACGTCTTACACA 15900

Lser_LAC005780 15851 GATGATAAAATCCATGGGCGTTCCAGTGGGCGTTATTCACGTCTTACACA 15900

Lvir_CGN013357 15849 GATGATAAAATCCATGGGCGTTCCAGTGGGCGTTATTCACGTCTTACACA 15898

Lsal_LAC008020 15848 GATGATAAAATCCATGGGCGTTCCAGTGGGCGTTATTCACGTCTTACACA 15897

Lsat_Salinas 15901 ACAACCCCTTAAAGGAAGGGCCAAGAAAGGGGGACAACGGGTAGGAGAAA 15950

Lsat_WENDEL 15901 ACAACCCCTTAAAGGAAGGGCCAAGAAAGGGGGACAACGGGTAGGAGAAA 15950

Lser_US96UC23 15901 ACAACCCCTTAAAGGAAGGGCCAAGAAAGGGGGACAACGGGTAGGAGAAA 15950

Lser_LAC005780 15901 ACAACCCCTTAAAGGAAGGGCCAAGAAAGGGGGACAACGGGTAGGAGAAA 15950

Lvir_CGN013357 15899 ACAACCCCTTAAAGGAAGGGCCAAGAAAGGGGGACAACGGGTAGGAGAAA 15948

Lsal_LAC008020 15898 ACAACCCCTTAAAGGAAGGGCCAAGAAAGGGGGACAACGGGTAGGAGAAA 15947

Lsat_Salinas 15951 TGGAGGTTTGGGCTTTAGAGGGGTTTGGCGTTGCTTATATTTTACAAGAG 16000

Lsat_WENDEL 15951 TGGAGGTTTGGGCTTTAGAGGGGTTTGGCGTTGCTTATATTTTACAAGAG 16000

Lser_US96UC23 15951 TGGAGGTTTGGGCTTTAGAGGGGTTTGGCGTTGCTTATATTTTACAAGAG 16000

Lser_LAC005780 15951 TGGAGGTTTGGGCTTTAGAGGGGTTTGGCGTTGCTTATATTTTACAAGAG 16000

Lvir_CGN013357 15949 TGGAGGTTTGGGCTTTAGAGGGGTTTGGCGTTGCTTATATTTTACAAGAG 15998

Lsal_LAC008020 15948 TGGAGGTTTGGGCTTTAGAGGGGTTTGGCGTTGCTTATATTTTACAAGAG 15997

Lsat_Salinas 16001 ATGCTTACTTATAAATCTGATCATATTAGAGCGCGCCAGGAAGTACTTGG 16050

Lsat_WENDEL 16001 ATGCTTACTTATAAATCTGATCATATTAGAGCGCGCCAGGAAGTACTTGG 16050

Lser_US96UC23 16001 ATGCTTACTTATAAATCTGATCATATTAGAGCGCGCCAGGAAGTACTTGG 16050

Lser_LAC005780 16001 ATGCTTACTTATAAATCTGATCATATTAGAGCGCGCCAGGAAGTACTTGG 16050

Lvir_CGN013357 15999 ATGCTTACTTATAAATCTGATCATATTAGAGCGCGCCAGGAAGTACTTGG 16048

Lsal_LAC008020 15998 ATGCTTACTTATAAATCTGATCATATTAGAGCGCGCCAGGAAGTACTTGG 16047

Lsat_Salinas 16051 TACTATAATCTTTGGAGGAAGAATACCGACTCCTGAAGATGCTCCAGAAT 16100

Lsat_WENDEL 16051 TACTATAATCTTTGGAGGAAGAATACCGACTCCTGAAGATGCTCCAGAAT 16100

Lser_US96UC23 16051 TACTATAATCTTTGGAGGAAGAATACCGACTCCTGAAGATGCTCCAGAAT 16100

Lser_LAC005780 16051 TACTATAATCTTTGGAGGAAGAATACCGACTCCTGAAGATGCTCCAGAAT 16100

Lvir_CGN013357 16049 TACTATAATCTTTGGAGGAAGAATACCGACTCCTGAAGATGCTCCAGAAT 16098

Lsal_LAC008020 16048 TACTATAATCTTTGGAGGAAGAATACCGACTCCTGAAGATGCTCCAGAAT 16097

Lsat_Salinas 16101 CTTTTCGGTTGTTCGTTCGAGAACTACGATCTTTAGCTCTGGAACTGAAT 16150

Lsat_WENDEL 16101 CTTTTCGGTTGTTCGTTCGAGAACTACGATCTTTAGCTCTGGAACTGAAT 16150

Lser_US96UC23 16101 CTTTTCGGTTGTTCGTTCGAGAACTACGATCTTTAGCTCTGGAACTGAAT 16150

Lser_LAC005780 16101 CTTTTCGGTTGTTCGTTCGAGAACTACGATCTTTAGCTCTGGAACTGAAT 16150

Lvir_CGN013357 16099 CTTTTCGGTTGTTCGTTCGAGAACTACGATCTTTAGCTCTGGAACTGAAT 16148

Lsal_LAC008020 16098 CTTTTCGGTTGTTCGTTCGAGAACTACGATCTTTAGCTCTGGAACTGAAT 16147

Lsat_Salinas 16151 CATTTTCTTGTATCTGAGAAGACTTTCCAGCTTAATAGGAAGGAAGCTTA 16200

Lsat_WENDEL 16151 CATTTTCTTGTATCTGAGAAGACTTTCCAGCTTAATAGGAAGGAAGCTTA 16200

Lser_US96UC23 16151 CATTTTCTTGTATCTGAGAAGACTTTCCAGCTTAATAGGAAGGAAGCTTA 16200

Lser_LAC005780 16151 CATTTTCTTGTATCTGAGAAGACTTTCCAGCTTAATAGGAAGGAAGCTTA 16200

Lvir_CGN013357 16149 CATTTTCTTGTATCTGAGAAGACTTTCCAGCTTAATAGGAAGGAAGCTTA 16198

Lsal_LAC008020 16148 CATTTTCTTGTATCTGAGAAGACTTTCCAGCTTAATAGGAAGGAAGCTTA 16197

Lsat_Salinas 16201 ATCGAAATCAAGCAGAAGTTTTCTTCTATGATCGATCGATATACACATCA 16250

Lsat_WENDEL 16201 ATCGAAATCAAGCAGAAGTTTTCTTCTATGATCGATCGATATACACATCA 16250

Lser_US96UC23 16201 ATCGAAATCAAGCAGAAGTTTTCTTCTATGATCGATCGATATACACATCA 16250

Lser_LAC005780 16201 ATCGAAATCAAGCAGAAGTTTTCTTCTATGATCGATCGATATACACATCA 16250

Lvir_CGN013357 16199 ATCGAAATCAAGCAGAAGTTTTCTTCTATGATCGATCGATATACACATCA 16248

Lsal_LAC008020 16198 ATCGAAATCAAGCAGAAGTTTTCTTCTATGATCGATCGATATACACATCA 16247

Lsat_Salinas 16251 ACAACTCCGAATTGGATTAGTTTCTCCTCAACAAATAAGTACTTGGTCCA 16300

Lsat_WENDEL 16251 ACAACTCCGAATTGGATTAGTTTCTCCTCAACAAATAAGTACTTGGTCCA 16300

Lser_US96UC23 16251 ACAACTCCGAATTGGATTAGTTTCTCCTCAACAAATAAGTACTTGGTCCA 16300

Lser_LAC005780 16251 ACAACTCCGAATTGGATTAGTTTCTCCTCAACAAATAAGTACTTGGTCCA 16300

Lvir_CGN013357 16249 ACAACTCCGAATTGGATTAGTTTCTCCTCAACAAATAAGTACTTGGTCCA 16298

Lsal_LAC008020 16248 ACAACTCCGAATTGGATTAGTTTCTCCTCAACAAATAAGTACTTGGTCCA 16297

Lsat_Salinas 16301 AAAAAATCCTGCCTAATGGCGAGATAGTTGGAGAGGTGACAAAACCTTAT 16350

Lsat_WENDEL 16301 AAAAAATCCTGCCTAATGGCGAGATAGTTGGAGAGGTGACAAAACCTTAT 16350

Lser_US96UC23 16301 AAAAAATCCTGCCTAATGGCGAGATAGTTGGAGAGGTGACAAAACCTTAT 16350

Lser_LAC005780 16301 AAAAAATCCTGCCTAATGGCGAGATAGTTGGAGAGGTGACAAAACCTTAT 16350

Lvir_CGN013357 16299 AAAAAATCCTGCCTAATGGCGAGATAGTTGGAGAGGTGACAAAACCTTAT 16348

Lsal_LAC008020 16298 AAAAAATCCTGCCTAATGGCGAGATAGTTGGAGAGGTGACAAAACCTTAT 16347

Lsat_Salinas 16351 ACCTTTCATTACAAAACCAATAAACCAGAAAAAGATGGATTATTTTGTGA 16400

Lsat_WENDEL 16351 ACCTTTCATTACAAAACCAATAAACCAGAAAAAGATGGATTATTTTGTGA 16400

Lser_US96UC23 16351 ACCTTTCATTACAAAACCAATAAACCAGAAAAAGATGGATTATTTTGTGA 16400

Lser_LAC005780 16351 ACCTTTCATTACAAAACCAATAAACCAGAAAAAGATGGATTATTTTGTGA 16400

Lvir_CGN013357 16349 ACCTTTCATTACAAAACCAATAAACCAGAAAAAGATGGATTATTTTGTGA 16398

Lsal_LAC008020 16348 ACCTTTCATTACAAAACCAATAAACCAGAAAAAGATGGATTATTTTGTGA 16397

Lsat_Salinas 16401 AAGAATTTTTGGGCCTATCAAAAGTGGAATTTGTGCTTGTGGAAATTATC 16450

Lsat_WENDEL 16401 AAGAATTTTTGGGCCTATCAAAAGTGGAATTTGTGCTTGTGGAAATTATC 16450

Lser_US96UC23 16401 AAGAATTTTTGGGCCTATCAAAAGTGGAATTTGTGCTTGTGGAAATTATC 16450

Lser_LAC005780 16401 AAGAATTTTTGGGCCTATCAAAAGTGGAATTTGTGCTTGTGGAAATTATC 16450

Lvir_CGN013357 16399 AAGAATTTTTGGGCCTATCAAAAGTGGAATTTGTGCTTGTGGAAATTATC 16448

Lsal_LAC008020 16398 AAGAATTTTTGGGCCTATCAAAAGTGGAATTTGTGCTTGTGGAAATTATC 16447

Lsat_Salinas 16451 GAGTAATCGGAGATGAAAAGGAAGACCCGCAATTTTGTGAACAATGCGGA 16500

Lsat_WENDEL 16451 GAGTAATCGGAGATGAAAAGGAAGACCCGCAATTTTGTGAACAATGCGGA 16500

Lser_US96UC23 16451 GAGTAATCGGAGATGAAAAGGAAGACCCGCAATTTTGTGAACAATGCGGA 16500

Lser_LAC005780 16451 GAGTAATCGGAGATGAAAAGGAAGACCCGCAATTTTGTGAACAATGCGGA 16500

Lvir_CGN013357 16449 GAGTAATCGGAGATGAAAAGGAAGACCCGCAATTTTGTGAACAATGCGGA 16498

Lsal_LAC008020 16448 GAGTAATCGGAGATGAAAAGGAAGACCCGCAATTTTGTGAACAATGCGGA 16497

Lsat_Salinas 16501 GTCGAGTTTGTTGATTCTCGGATACGAAGATATCAAATGGGCTACATCAA 16550

Lsat_WENDEL 16501 GTCGAGTTTGTTGATTCTCGGATACGAAGATATCAAATGGGCTACATCAA 16550

Lser_US96UC23 16501 GTCGAGTTTGTTGATTCTCGGATACGAAGATATCAAATGGGCTACATCAA 16550

Lser_LAC005780 16501 GTCGAGTTTGTTGATTCTCGGATACGAAGATATCAAATGGGCTACATCAA 16550

Lvir_CGN013357 16499 GTCGAGTTTGTTGATTCTCGGATACGAAGATATCAAATGGGCTACATCAA 16548

Lsal_LAC008020 16498 GTCGAGTTTGTTGATTCTCGGATACGAAGATATCAAATGGGCTACATCAA 16547

Lsat_Salinas 16551 ACTCGCATACCCGGTAATGCATGTGTGGTATTTGAAACGTCTTCCTAGTT 16600

Lsat_WENDEL 16551 ACTCGCATACCCGGTAATGCATGTGTGGTATTTGAAACGTCTTCCTAGTT 16600

Lser_US96UC23 16551 ACTCGCATACCCGGTAATGCATGTGTGGTATTTGAAACGTCTTCCTAGTT 16600

Lser_LAC005780 16551 ACTCGCATACCCGGTAATGCATGTGTGGTATTTGAAACGTCTTCCTAGTT 16600

Lvir_CGN013357 16549 ACTCGCATACCCGGTAATGCATGTGTGGTATTTGAAACGTCTTCCTAGTT 16598

Lsal_LAC008020 16548 ACTCGCATACCCGGTAATGCATGTGTGGTATTTGAAACGTCTTCCTAGTT 16597

Lsat_Salinas 16601 ATATTGTGACTCTTTTAGATAAACCTCTTAACGAATTAGAAGACCTAGTA 16650

Lsat_WENDEL 16601 ATATTGTGACTCTTTTAGATAAACCTCTTAACGAATTAGAAGACCTAGTA 16650

Lser_US96UC23 16601 ATATTGTGACTCTTTTAGATAAACCTCTTAACGAATTAGAAGACCTAGTA 16650

Lser_LAC005780 16601 ATATTGTGACTCTTTTAGATAAACCTCTTAACGAATTAGAAGACCTAGTA 16650

Lvir_CGN013357 16599 ATATTGTGACTCTTTTAGATAAACCTCTTAACGAATTAGAAGACCTAGTA 16648

Lsal_LAC008020 16598 ATATTGTGACTCTTTTAGATAAACCTCTTAACGAATTAGAAGACCTAGTA 16647

Lsat_Salinas 16651 TACTGCGGTGTGTGATTTGATCGAAATTCTGATTTTACAGATTCGAAATG 16700

Lsat_WENDEL 16651 TACTGCGGTGTGTGATTTGATCGAAATTCTGATTTTACAGATTCGAAATG 16700

Lser_US96UC23 16651 TACTGCGGTGTGTGATTTGATCGAAATTCTGATTTTACAGATTCGAAATG 16700

Lser_LAC005780 16651 TACTGCGGTGTGTGATTTGATCGAAATTCTGATTTTACAGATTCGAAATG 16700

Lvir_CGN013357 16649 TACTGCGGTGTGTGATTTGATCGAAATTCTGATTTTACAGATTCGAAATG 16698

Lsal_LAC008020 16648 TACTGCGGTGTGTGATTTGATCGAAATTCTGATTTTACAGATTCGAAATG 16697

Lsat_Salinas 16701 AGAAACTGTCATCCCATTTAATCCAATCGGGATGCCCTGTACCTGACATG 16750

Lsat_WENDEL 16701 AGAAACTGTCATCCCATTTAATCCAATCGGGATGCCCTGTACCTGACATG 16750

Lser_US96UC23 16701 AGAAACTGTCATCCCATTTAATCCAATCGGGATGCCCTGTACCTGACATG 16750

Lser_LAC005780 16701 AGAAACTGTCATCCCATTTAATCCAATCGGGATGCCCTGTACCTGACATG 16750

Lvir_CGN013357 16699 AGAAACTGTCATCCCATTTAATCCAATCGGGATGCCCTGTACCTGACATG 16748

Lsal_LAC008020 16698 AGAAACTGTCATCCCATTTAATCCAATCGGGATGCCCTGTACCTGACATG 16747

Lsat_Salinas 16751 TTTCTTGGTAGGAGTAACATGAAGCTCAGAATTAGGGATGTATTCAAGAC 16800

Lsat_WENDEL 16751 TTTCTTGGTAGGAGTAACATGAAGCTCAGAATTAGGGATGTATTCAAGAC 16800

Lser_US96UC23 16751 TTTCTTGGTAGGAGTAACATGAAGCTCAGAATTAGGGATGTATTCAAGAC 16800

Lser_LAC005780 16751 TTTCTTGGTAGGAGTAACATGAAGCTCAGAATTAGGGATGTATTCAAGAC 16800

Lvir_CGN013357 16749 TTTCTTGGTAGGAGTAACATGAAGCTCAGAATTAGGGATGTATTCAAGAC 16798

Lsal_LAC008020 16748 TTTCTTGGTAGGAGTAACATGAAGCTCAGAATTAGGGATGTATTCAAGAC 16797

Lsat_Salinas 16801 GCTCCCCAAAAAGGGAATTGATCTATGGTCGATTTCATAAGAATTTATAG 16850

Lsat_WENDEL 16801 GCTCCCCAAAAAGGGAATTGATCTATGGTCGATTTCATAAGAATTTATAG 16850

Lser_US96UC23 16801 GCTCCCCAAAAAGGGAATTGATCTATGGTCGATTTCATAAGAATTTATAG 16850

Lser_LAC005780 16801 GCTCCCCAAAAAGGGAATTGATCTATGGTCGATTTCATAAGAATTTATAG 16850

Lvir_CGN013357 16799 GCTCCCCAAAAAGGGAATTGATCTATGGTCGATTTCATAAGAATTTATAG 16848

Lsal_LAC008020 16798 GCTCCCCAAAAAGGGAATTGATCTATGGTCGATTTCATAAGAATTTATAG 16847

Lsat_Salinas 16851 TTAGACCTCGTAAAAAAAAGACTTTTTCTTTTGTGGAATTAAGCAGTTCC 16900

Lsat_WENDEL 16851 TTAGACCTCGTAAAAAAAAGACTTTTTCTTTTGTGGAATTAAGCAGTTCC 16900

Lser_US96UC23 16851 TTAGACCTCGTAAAAAAAAGACTTTTTCTTTTGTGGAATTAAGCAGTTCC 16900

Lser_LAC005780 16851 TTAGACCTCGTAAAAAAAAGACTTTTTCTTTTGTGGAATTAAGCAGTTCC 16900

Lvir_CGN013357 16849 TTAGACCTCGTAAAAAAAAGACTTTTTCTTTTGTGGAATTAAGCAGTTCC 16898

Lsal_LAC008020 16848 TTAGACCTCGTAAAAAAAAGACTTTTTCTTTTGTGGAATTAAGCAGTTCC 16897

Lsat_Salinas 16901 TTTTTTAGAAAGAAATTATGTTTAAGTAAGCAAATAGAAAAGATGTCATG 16950

Lsat_WENDEL 16901 TTTTTTAGAAAGAAATTATGTTTAAGTAAGCAAATAGAAAAGATGTCATG 16950

Lser_US96UC23 16901 TTTTTTAGAAAGAAATTATGTTTAAGTAAGCAAATAGAAAAGATGTCATG 16950

Lser_LAC005780 16901 TTTTTTAGAAAGAAATTATGTTTAAGTAAGCAAATAGAAAAGATGTCATG 16950

Lvir_CGN013357 16899 TTTTTTAGAAAGAAATTATGTTTAAGTAAGCAAATAGAAAAGATGTCATG 16948

Lsal_LAC008020 16898 TTTTTTAGAAACAAATTATGTTTAAGTAAGCAAATAGAAAAGATGTCATG 16947

Lsat_Salinas 16951 GTTACAAGAGTCTATCTATCGCATATAGACTTTAAGGGCATCGTGGCCTA 17000

Lsat_WENDEL 16951 GTTACAAGAGTCTATCTATCGCATATAGACTTTAAGGGCATCGTGGCCTA 17000

Lser_US96UC23 16951 GTTACAAGAGTCTATCTATCGCATATAGACTTTAAGGGCATCGTGGCCTA 17000

Lser_LAC005780 16951 GTTACAAGAGTCTATCTATCGCATATAGACTTTAAGGGCATCGTGGCCTA 17000

Lvir_CGN013357 16949 GTTACAAGAGTCTATCTATCGCATATAGACTTTAAGGGCATCGTGGCCTA 16998

Lsal_LAC008020 16948 GTTACAAGAGTCTATCTATCGCATATAGACTTTAAGGGCATCGTGGCCTA 16997

Lsat_Salinas 17001 ACCGTCGAGGTGAAGTCGGGACCTAAAAGATCAAATGGAACAGTACAATA 17050

Lsat_WENDEL 17001 ACCGTCGAGGTGAAGTCGGGACCTAAAAGATCAAATGGAACAGTACAATA 17050

Lser_US96UC23 17001 ACCGTCGAGGTGAAGTCGGGACCTAAAAGATCAAATGGAACAGTACAATA 17050

Lser_LAC005780 17001 ACCGTCGAGGTGAAGTCGGGACCTAAAAGATCAAATGGAACAGTACAATA 17050

Lvir_CGN013357 16999 ACCGTCGAGGTGAAGTCGGGACCTAAAAGATCAAATGGAACAGTACAATA 17048

Lsal_LAC008020 16998 ACCGTCGAGGTGAAGTCGGGACCTAAAAGATCAAATGGAACAGTACAATA 17047

Lsat_Salinas 17051 GACAAGTAAATTCCTTCTGAATTCTAAGGTACTCTCTTTCATTAAGAATT 17100

Lsat_WENDEL 17051 GACAAGTAAATTCCTTCTGAATTCTAAGGTACTCTCTTTCATTAAGAATT 17100

Lser_US96UC23 17051 GACAAGTAAATTCCTTCTGAATTCTAAGGTACTCTCTTTCATTAAGAATT 17100

Lser_LAC005780 17051 GACAAGTAAATTCCTTCTGAATTCTAAGGTACTCTCTTTCATTAAGAATT 17100

Lvir_CGN013357 17049 GACAAGTAAATTCCTTCTGAATTCTAAGGTACTCTCTTTCATTAAGAATT 17098

Lsal_LAC008020 17048 GACAAGTAAATTCCTTCTGAATTCTAAGGTACTCTCTTTCATTAAGAATT 17097

Lsat_Salinas 17101 ACGGGATTCATCATTCGAGGGGAAGTAGACTACTCAAGAATTTCACATTT 17150

Lsat_WENDEL 17101 ACGGGATTCATCATTCGAGGGGAAGTAGACTACTCAAGAATTTCACATTT 17150

Lser_US96UC23 17101 ACGGGATTCATCATTCGAGGGGAAGTAGACTACTCAAGAATTTCACATTT 17150

Lser_LAC005780 17101 ACGGGATTCATCATTCGAGGGGAAGTAGACTACTCAAGAATTTCACATTT 17150

Lvir_CGN013357 17099 ACGGGATTCATCATTCGAGGGGAAGTAGACTACTCAAGAATTTCACATTT 17148

Lsal_LAC008020 17098 ACGGGATTCATCATTCGAGGGGAAGTAGACTACTCAAGAATTTCACATTT 17147

Lsat_Salinas 17151 CATTTATGTCATAATTGAATAAAGAATTCATAAAATCTAAATAAAAATAA 17200

Lsat_WENDEL 17151 CATTTATGTCATAATTGAATAAAGAATTCATAAAATCTAAATAAAAATAA 17200

Lser_US96UC23 17151 CATTTATGTCATAATTGAATAAAGAATTCATAAAATCTAAATAAAAATAA 17200

Lser_LAC005780 17151 CATTTATGTCATAATTGAATAAAGAATTCATAAAATCTAAATAAAAATAA 17200

Lvir_CGN013357 17149 CATTTATGTCATAATTGAATAAAGAATTCATAAAATCTAAATAAAAATAA 17198

Lsal_LAC008020 17148 CATTTATGTCATAATTGAATAAAGAATTCATAAAATCTAAATAAAAATAA 17197

Lsat_Salinas 17201 TTAAGGAAGACGGAATCCATGAAGTTTTGCTTGGTCTTCACTGGAAACTT 17250

Lsat_WENDEL 17201 TTAAGGAAGACGGAATCCATGAAGTTTTGCTTGGTCTTCACTGGAAACTT 17250

Lser_US96UC23 17201 TTAAGGAAGACGGAATCCATGAAGTTTTGCTTGGTCTTCACTGGAAACTT 17250

Lser_LAC005780 17201 TTAAGGAAGACGGAATCCATGAAGTTTTGCTTGGTCTTCACTGGAAACTT 17250

Lvir_CGN013357 17199 TTAAGGAAGACGGAATCCATGAAGTTTTGCTTGGTCTTCACTGGAAACTT 17248

Lsal_LAC008020 17198 TTAAGGAAGACGGAATCCATGAAGTTTTGCTTGGTCTTCACTGGAAACTT 17247

Lsat_Salinas 17251 GAGTAAGGAGTAGATCTTTTTGGAGTTTTCTAGAATTTGAAAGCGAGAAC 17300

Lsat_WENDEL 17251 GAGTAAGGAGTAGATCTTTTTGGAGTTTTCTAGAATTTGAAAGCGAGAAC 17300

Lser_US96UC23 17251 GAGTAAGGAGTAGATCTTTTTGGAGTTTTCTAGAATTTGAAAGCGAGAAC 17300

Lser_LAC005780 17251 GAGTAAGGAGTAGATCTTTTTGGAGTTTTCTAGAATTTGAAAGCGAGAAC 17300

Lvir_CGN013357 17249 GAGTAAGGAGTAGATCTTTTTGGAGTTTTCTAGAATTTGAAAGCGAGAAC 17298

Lsal_LAC008020 17248 GAGTAAGGAGTAGATCTTTTTGGAGTTTTCTAGAATTTGAAAGCAAGAAC 17297

Lsat_Salinas 17301 TCCTTTCTTTTTCTTTTTTTGGTATACCTACTTGAGCCGGATGAAAGGAA 17350

Lsat_WENDEL 17301 TCCTTTCTTTTTCTTTTTTTGGTATACCTACTTGAGCCGGATGAAAGGAA 17350

Lser_US96UC23 17301 TCCTTTCTTTTTCTTTTTTTGGTATACCTACTTGAGCCGGATGAAAGGAA 17350

Lser_LAC005780 17301 TCCTTTCTTTTTCTTTTTTTGGTATACCTACTTGAGCCGGATGAAAGGAA 17350

Lvir_CGN013357 17299 TCCTTTCTTTTTCTTTTTTTGGTATACCTACTTGAGCCGGATGAAAGGAA 17348

Lsal_LAC008020 17298 TCCTTTCTTTTTCTTTTTTTGGTATACCTACTTGAGCCGGATGAAAGGAA 17347

Lsat_Salinas 17351 ACTTTCACGTCCGATTTTGAGGGGGGGAGATCCTATCCCAATTTTTATTT 17400

Lsat_WENDEL 17351 ACTTTCACGTCCGATTTTGMGGGGGGGAGATCCTATCCCAATTTTTATTT 17400

Lser_US96UC23 17351 ACTTTCACGTCCGATTTTGAGGGGGGGAGATCCTATCCCAATTTTTATTT 17400

Lser_LAC005780 17351 ACTTTCACGTCCGATTTTGAGGGGGGGAGATCCTATCCCAATTTTTATTT 17400

Lvir_CGN013357 17349 ACTTTCACGTCCGATTTTGCGGGGGGGAGATCCTATCCCAATTTTTATTT 17398

Lsal_LAC008020 17348 ACTTTCACGTCCGATTTTGAGGGGGGGAGATCCTATCCCAATTTTTATTT 17397

Lsat_Salinas 17401 TGCTAGGCCCATAGATAAAAAACCTACTTTTTTACGATTACGGGGTTTAT 17450

Lsat_WENDEL 17401 TGCTAGGCCCATAGATAAAAAACCTACTTTTTTACGATTACGGGGTTTAT 17450

Lser_US96UC23 17401 TGCTAGGCCCATAGATAAAAAACCTACTTTTTTACGATTACGGGGTTTAT 17450

Lser_LAC005780 17401 TGCTAGGCCCATAGATAAAAAACCTACTTTTTTACGATTACGGGGTTTAT 17450

Lvir_CGN013357 17399 TGCTAGGCCCATAGATAAAAAACCTACTTTTTTACGATTACGGGGTTTAT 17448

Lsal_LAC008020 17398 TGCTAGGCCCATAGATAAAAAACCTACTTTTTTACGATTACGGGGTTTAT 17447

Lsat_Salinas 17451 TAGAATATGAAATTCAACCCTGGAAATACAGGATCCCCATTTTTTTTACT 17500

Lsat_WENDEL 17451 TAGAATATGAAATTCAACCCTGGAAATACAGGATCCCCATTTTTTTTACT 17500

Lser_US96UC23 17451 TAGAATATGAAATTCAACCCTGGAAATACAGGATCCCCATTTTTTTTACT 17500

Lser_LAC005780 17451 TAGAATATGAAATTCAACCCTGGAAATACAGGATCCCCATTTTTTTTACT 17500

Lvir_CGN013357 17449 TAGAATATGAAATTCAACCCTGGAAATACAGGATCCCCATTTTTTTTACT 17498

Lsal_LAC008020 17448 TAGAATATGAAATTCAACCCTGGAAATACAGGATCCCCATTTTTTTTACT 17497

Lsat_Salinas 17501 ACCCGGAGCTTCGATACATTTCGAAATCGAGAGATGTCTACCGGGGGAGG 17550

Lsat_WENDEL 17501 ACCCGGAGCTTCGATACATTTCGAAATCGAGAGATGTCTACCGGGGGAGG 17550

Lser_US96UC23 17501 ACCCGGAGCTTCGATACATTTCGAAATCGAGAGATGTCTACCGGGGGAGG 17550

Lser_LAC005780 17501 ACCCGGAGCTTCGATACATTTCGAAATCGAGAGATGTCTACCGGGGGAGG 17550

Lvir_CGN013357 17499 ACCCGGAGCTTCGATACATTTCGAAATCGAGAGATGTCTACCGGGGGAGG 17548

Lsal_LAC008020 17498 ACCCGGAGCTTCGATACATTTCGAAATCGAGAGATGTCTACTGGGGGAGG 17547

Lsat_Salinas 17551 TTCTATCAGACAACAATTAGCCAATCTAGATTTACGAATTATTATAGACT 17600

Lsat_WENDEL 17551 TTCTATCAGACAACAATTAGCCAATCTAGATTTACGAATTATTATAGACT 17600

Lser_US96UC23 17551 TTCTATCAGACAACAATTAGCCAATCTAGATTTACGAATTATTATAGACT 17600

Lser_LAC005780 17551 TTCTATCAGACAACAATTAGCCAATCTAGATTTACGAATTATTATAGACT 17600

Lvir_CGN013357 17549 TTCTATCAGACAACAATTAGCCAATCTAGATTTACGAATTATTATAGACT 17598

Lsal_LAC008020 17548 TTCTATTAGACAACAATTAGCCAATCTAGATTTACGAATTATTATAGACT 17597

Lsat_Salinas 17601 ATTCATTGGTAGAATGGAAAGAATTGGAGGAAGAGGAACCCACAGGGAAC 17650

Lsat_WENDEL 17601 ATTCATTGGTAGAATGGAAAGAATTGGAGGAAGAGGAACCCACAGGGAAC 17650

Lser_US96UC23 17601 ATTCATTGGTAGAATGGAAAGAATTGGAGGAAGAGGAACCCACAGGGAAC 17650

Lser_LAC005780 17601 ATTCATTGGTAGAATGGAAAGAATTGGAGGAAGAGGAACCCACAGGGAAC 17650

Lvir_CGN013357 17599 ATTCATTGGTAGAATGGAAAGAATTGGAGGAAGAGGAACCCACAGGGAAC 17648

Lsal_LAC008020 17598 ATTCATTGGTAGAATGGAAAGAATTGGAGGAAGAGGAACCCACAGGGAAC 17647

Lsat_Salinas 17651 GAATGGGAAGATCGAAAAGTTGGAAGAAGAAAAGATTTTTTGCTTAGACG 17700

Lsat_WENDEL 17651 GAATGGGAAGATCGAAAAGTTGGAAGAAGAAAAGATTTTTTGCTTAGACG 17700

Lser_US96UC23 17651 GAATGGGAAGATCGAAAAGTTGGAAGAAGAAAAGATTTTTTGCTTAGACG 17700

Lser_LAC005780 17651 GAATGGGAAGATCGAAAAGTTGGAAGAAGAAAAGATTTTTTGCTTAGACG 17700

Lvir_CGN013357 17649 GAATGGGAAGATCGAAAAGTTGGAAGAAGAAAAGATTTTTTGCTTAGACG 17698

Lsal_LAC008020 17648 GAATGGGAAGATCGAAAAGTTGGAAGAAGAAAAGATTTTTTGCTTAGACG 17697

Lsat_Salinas 17701 CATGGAATTGGCTAAGCATTTTATTCGAACAAATATAGAACCAAAATGGA 17750

Lsat_WENDEL 17701 CATGGAATTGGCTAAGCATTTTATTCGAACAAATATAGAACCAAAATGGA 17750

Lser_US96UC23 17701 CATGGAATTGGCTAAGCATTTTATTCGAACAAATATAGAACCAAAATGGA 17750

Lser_LAC005780 17701 CATGGAATTGGCTAAGCATTTTATTCGAACAAATATAGAACCAAAATGGA 17750

Lvir_CGN013357 17699 CATGGAATTGGCTAAGCATTTTATTCGAACAAATATAGAACCAAAATGGA 17748

Lsal_LAC008020 17698 CATGGAATTGGCTAAGCATTTTATTCGAACAAATATAGAACCAAAATGGA 17747

Lsat_Salinas 17751 TGGTTTTGCGTCTATTACCAGTTCTTCCTCCTGAGTTGAGACCAATCTAT 17800

Lsat_WENDEL 17751 TGGTTTTGCGTCTATTACCAGTTCTTCCTCCTGAGTTGAGACCAATCTAT 17800

Lser_US96UC23 17751 TGGTTTTGCGTCTATTACCAGTTCTTCCTCCTGAGTTGAGACCAATCTAT 17800

Lser_LAC005780 17751 TGGTTTTGCGTCTATTACCAGTTCTTCCTCCTGAGTTGAGACCAATCTAT 17800

Lvir_CGN013357 17749 TGGTTTTGCGTCTATTACCAGTTCTTCCTCCTGAGTTGAGACCAATCTAT 17798

Lsal_LAC008020 17748 TGGTTTTGCGTCTATTACCAGTTCTTCCTCCTGAGTTGAGACCAATCTAT 17797

Lsat_Salinas 17801 CATATAGATGAGGATAAACTAGTGACCTCGGATATTAATGAAATCTATAG 17850

Lsat_WENDEL 17801 CATATAGATGAGGATAAACTAGTGACCTCGGATATTAATGAAATCTATAG 17850

Lser_US96UC23 17801 CATATAGATGAGGATAAACTAGTGACCTCGGATATTAATGAAATCTATAG 17850

Lser_LAC005780 17801 CATATAGATGAGGATAAACTAGTGACCTCGGATATTAATGAAATCTATAG 17850

Lvir_CGN013357 17799 CATATAGATGAGGATAAACTAGTGACCTCGGATATTAATGAAATCTATAG 17848

Lsal_LAC008020 17798 CATATAGATGAGGATAAACTAGTGACCTCGGATATTAATGAAATCTATAG 17847

Lsat_Salinas 17851 AAGAATTATCTATCGGAATAATACTCTTACAGATCTATTAACAACAAGTA 17900

Lsat_WENDEL 17851 AAGAATTATCTATCGGAATAATACTCTTACAGATCTATTAACAACAAGTA 17900

Lser_US96UC23 17851 AAGAATTATCTATCGGAATAATACTCTTACAGATCTATTAACAACAAGTA 17900

Lser_LAC005780 17851 AAGAATTATCTATCGGAATAATACTCTTACAGATCTATTAACAACAAGTA 17900

Lvir_CGN013357 17849 AAGAATTATCTATCGGAATAATACTCTTACAGATCTATTAACAACAAGTA 17898

Lsal_LAC008020 17848 AAGAATTATCTATCGGAATAATACTCTTACAGATCTATTAACAACAAGTA 17897

Lsat_Salinas 17901 TAGCTACGCCAGAAGAATTAATAATATCTCAGGAAAAATTGCTACAAGAA 17950

Lsat_WENDEL 17901 TAGCTACGCCAGAAGAATTAATAATATCTCAGGAAAAATTGCTACAAGAA 17950

Lser_US96UC23 17901 TAGCTACGCCAGAAGAATTAATAATATCTCAGGAAAAATTGCTACAAGAA 17950

Lser_LAC005780 17901 TAGCTACGCCAGAAGAATTAATAATATCTCAGGAAAAATTGCTACAAGAA 17950

Lvir_CGN013357 17899 TAGCTACGCCAGAAGAATTAATAATATCTCAGGAAAAATTGCTACAAGAA 17948

Lsal_LAC008020 17898 TAGCTACGCCAGAAGAATTAATAATATCTCAGGAAAAATTGCTACAAGAA 17947

Lsat_Salinas 17951 GCCGTGGATGCACTTCTTGATAATGGAATCTGCGGACAACCAATGAGGGA 18000

Lsat_WENDEL 17951 GCCGTGGATGCACTTCTTGATAATGGAATCTGCGGACAACCAATGAGGGA 18000

Lser_US96UC23 17951 GCCGTGGATGCACTTCTTGATAATGGAATCTGCGGACAACCAATGAGGGA 18000

Lser_LAC005780 17951 GCCGTGGATGCACTTCTTGATAATGGAATCTGCGGACAACCAATGAGGGA 18000

Lvir_CGN013357 17949 GCCGTGGATGCACTTCTTGATAATGGAATCTGCGGACAACCAATGAGGGA 17998

Lsal_LAC008020 17948 GCCGTGGATGCACTTCTTGATAATGGAATCTGCGGACAACCAATGAGGGA 17997

Lsat_Salinas 18001 TGATCATAATAGAGTTTACAAGTCGCTTTCAGATGTAATTGAAGGCAAAG 18050

Lsat_WENDEL 18001 TGATCATAATAGAGTTTACAAGTCGCTTTCAGATGTAATTGAAGGCAAAG 18050

Lser_US96UC23 18001 TGATCATAATAGAGTTTACAAGTCGCTTTCAGATGTAATTGAAGGCAAAG 18050

Lser_LAC005780 18001 TGATCATAATAGAGTTTACAAGTCGCTTTCAGATGTAATTGAAGGCAAAG 18050

Lvir_CGN013357 17999 TGATCATAATAGAGTTTACAAGTCGCTTTCAGATGTAATTGAAGGCAAAG 18048

Lsal_LAC008020 17998 TGATCATAATAGAGTTTACAAGTCGCTTTCAGATGTAATTGAAGGCAAAG 18047

Lsat_Salinas 18051 AAGGAAGAGTTCGCGAGACTCTGCTTGGTAAACGAGTCGATTATTCAGGG 18100

Lsat_WENDEL 18051 AAGGAAGAGTTCGCGAGACTCTGCTTGGTAAACGGGTCGATTATTCAGGG 18100

Lser_US96UC23 18051 AAGGAAGAGTTCGCGAGACTCTGCTTGGTAAACGAGTCGATTATTCAGGG 18100

Lser_LAC005780 18051 AAGGAAGAGTTCGCGAGACTCTGCTTGGTAAACGAGTCGATTATTCAGGG 18100

Lvir_CGN013357 18049 AAGGAAGAGTTCGCGAGACTCTGCTTGGTAAACGGGTCGATTATTCAGGG 18098

Lsal_LAC008020 18048 AAGGAAGAGTTCGCGAGACTCTGCTTGGTAAACGGGTCGATTATTCAGGG 18097

Lsat_Salinas 18101 CGGTCCGTGATTGTCGTGGGCCCTTCACTTTCATTACATCGATGTGGATT 18150

Lsat_WENDEL 18101 CGGTCCGTGATTGTCGTGGGCCCTTCACTTTCATTACATCGATGTGGATT 18150

Lser_US96UC23 18101 CGGTCCGTGATTGTCGTGGGCCCTTCACTTTCATTACATCGATGTGGATT 18150

Lser_LAC005780 18101 CGGTCCGTGATTGTCGTGGGCCCTTCACTTTCATTACATCGATGTGGATT 18150

Lvir_CGN013357 18099 CGGTCCGTGATTGTCGTGGGCCCTTCACTTTCATTACATCGATGTGGATT 18148

Lsal_LAC008020 18098 CGGTCCGTGATTGTCGTGGGCCCTTCACTTTCATTACATCGATGTGGATT 18147

Lsat_Salinas 18151 GCCTCGCGAAATAGCAATAGAACTTTTCCAGGCATTTGTAATTCGTGACC 18200

Lsat_WENDEL 18151 GCCTCGCGAAATAGCAATAGAACTTTTCCAGGCATTTGTAATTCGTGACC 18200

Lser_US96UC23 18151 GCCTCGCGAAATAGCAATAGAACTTTTCCAGGCATTTGTAATTCGTGACC 18200

Lser_LAC005780 18151 GCCTCGCGAAATAGCAATAGAACTTTTCCAGGCATTTGTAATTCGTGACC 18200

Lvir_CGN013357 18149 GCCTCGCGAAATAGCAATAGAACTTTTCCAGGCATTTGTAATTCGTGACC 18198

Lsal_LAC008020 18148 GCCTCGCGAAATAGCAATAGAACTTTTCCAGGCATTTGTAATTCGTGACC 18197

Lsat_Salinas 18201 TAATTAGAAAACATCTTGCTTCGAACATAGGAGTTGCTAAGAGTCAAATT 18250

Lsat_WENDEL 18201 TAATTAGAAAACATCTTGCTTCGAACATAGGAGTTGCTAAGAGTCAAATT 18250

Lser_US96UC23 18201 TAATTAGAAAACATCTTGCTTCGAACATAGGAGTTGCTAAGAGTCAAATT 18250

Lser_LAC005780 18201 TAATTAGAAAACATCTTGCTTCGAACATAGGAGTTGCTAAGAGTCAAATT 18250

Lvir_CGN013357 18199 TAATTAGAAAACATCTTGCTTCGAACATAGGAGTTGCTAAGAGTCAAATT 18248

Lsal_LAC008020 18198 TAATTAGAAAACATCTTGCTTCGAACATAGGAGTTGCTAAGAGTCAAATT 18247

Lsat_Salinas 18251 CGGAAAAAAAAACCGATTGTATGGGAAATACTTCAGGAAATTCTGGATGA 18300

Lsat_WENDEL 18251 CGGAAAAAAAAACCGATTGTATGGGAAATACTTCAGGAAATTCTGGATGA 18300

Lser_US96UC23 18251 CGGAAAAAAAAACCGATTGTATGGGAAATACTTCAGGAAATTCTGGATGA 18300

Lser_LAC005780 18251 CGGAAAAAAAAACCGATTGTATGGGAAATACTTCAGGAAATTCTGGATGA 18300

Lvir_CGN013357 18249 CGGAAAAAAAAACCGATTGTATGGGAAATACTTCAGGAAATTCTGGATGA 18298

Lsal_LAC008020 18248 CGGAAAAAAAAACCGATTGTATGGGAAATACTTCAGGAAATTCTGGATGA 18297

Lsat_Salinas 18301 CCATCCTGTATTGCTGAATAGAGCGCCTACTCTGCATAGATTAGGCATAC 18350

Lsat_WENDEL 18301 CCATCCTGTATTGCTGAATAGAGCGCCTACTCTGCATAGATTAGGCATAC 18350

Lser_US96UC23 18301 CCATCCTGTATTGCTGAATAGAGCGCCTACTCTGCATAGATTAGGCATAC 18350

Lser_LAC005780 18301 CCATCCTGTATTGCTGAATAGAGCGCCTACTCTGCATAGATTAGGCATAC 18350

Lvir_CGN013357 18299 CCATCCTGTATTGCTGAATAGAGCGCCTACTCTGCATAGATTAGGCATAC 18348

Lsal_LAC008020 18298 CCATCCTGTATTGCTGAATAGAGCGCCTACTCTGCATAGATTAGGCATAC 18347

Lsat_Salinas 18351 AGGCATTCCTCCCCGTTTTAGTGGAAGGGCGCGCTATTTGTTTACATCCA 18400

Lsat_WENDEL 18351 AGGCATTCCTCCCCGTTTTAGTGGAAGGGCGCGCTATTTGTTTACATCCA 18400

Lser_US96UC23 18351 AGGCATTCCTCCCCGTTTTAGTGGAAGGGCGCGCTATTTGTTTACATCCA 18400

Lser_LAC005780 18351 AGGCATTCCTCCCCGTTTTAGTGGAAGGGCGCGCTATTTGTTTACATCCA 18400

Lvir_CGN013357 18349 AGGCATTCCTCCCCGTTTTAGTGGAAGGGCGCGCTATTTGTTTACATCCA 18398

Lsal_LAC008020 18348 AGGCATTCCTCCCCGTTTTAGTGGAAGGGCGCGCTATTTGTTTACATCCA 18397

Lsat_Salinas 18401 TTAGTTTGTAAGGGCTTCAATGCAGACTTTGACGGGGATCAAATGGCTGT 18450

Lsat_WENDEL 18401 TTAGTTTGTAAGGGCTTCAATGCAGACTTTGACGGGGATCAAATGGCTGT 18450

Lser_US96UC23 18401 TTAGTTTGTAAGGGCTTCAATGCAGACTTTGACGGGGATCAAATGGCTGT 18450

Lser_LAC005780 18401 TTAGTTTGTAAGGGCTTCAATGCAGACTTTGACGGGGATCAAATGGCTGT 18450

Lvir_CGN013357 18399 TTAGTTTGTAAGGGCTTCAATGCAGACTTTGACGGGGATCAAATGGCTGT 18448

Lsal_LAC008020 18398 TTAGTTTGTAAGGGCTTCAATGCAGACTTTGACGGGGATCAAATGGCTGT 18447

Lsat_Salinas 18451 TCATGTGCCTTTATCTTTGGAGGCTCAAGCAGAGGCGCGTTTACTTATGT 18500

Lsat_WENDEL 18451 TCATGTGCCTTTATCTTTGGAGGCTCAAGCAGAGGCGCGTTTACTTATGT 18500

Lser_US96UC23 18451 TCATGTGCCTTTATCTTTGGAGGCTCAAGCAGAGGCGCGTTTACTTATGT 18500

Lser_LAC005780 18451 TCATGTGCCTTTATCTTTGGAGGCTCAAGCAGAGGCGCGTTTACTTATGT 18500

Lvir_CGN013357 18449 TCATGTGCCTTTATCTTTGGAGGCTCAAGCAGAGGCGCGTTTACTTATGT 18498

Lsal_LAC008020 18448 TCATGTGCCTTTATCTTTGGAGGCTCAAGCAGAGGCGCGTTTACTTATGT 18497

Lsat_Salinas 18501 TTTCTCATATGAATCTTTTGTCTCCAACTATTGGGGATCCGATTTCGGCA 18550

Lsat_WENDEL 18501 TTTCTCATATGAATCTTTTGTCTCCAACTATTGGGGATCCGATTTCGGCA 18550

Lser_US96UC23 18501 TTTCTCATATGAATCTTTTGTCTCCAACTATTGGGGATCCGATTTCGGCA 18550

Lser_LAC005780 18501 TTTCTCATATGAATCTTTTGTCTCCAACTATTGGGGATCCGATTTCGGCA 18550

Lvir_CGN013357 18499 TTTCTCATATGAATCTTTTGTCTCCAACTATTGGGGATCCGATTTCGGCA 18548

Lsal_LAC008020 18498 TTTCTCATATGAATCTTTTGTCTCCAACTATTGGGGATCCGATTTCGGCA 18547

Lsat_Salinas 18551 CCAACTCAAGATATGCTTAGTGGACTCTATGTCTTAACGAGTGGAAATCG 18600

Lsat_WENDEL 18551 CCAACTCAAGATATGCTTAGTGGACTCTATGTCTTAACGAGTGGAAATCG 18600

Lser_US96UC23 18551 CCAACTCAAGATATGCTTAGTGGACTCTATGTCTTAACGAGTGGAAATCG 18600

Lser_LAC005780 18551 CCAACTCAAGATATGCTTAGTGGACTCTATGTCTTAACGAGTGGAAATCG 18600

Lvir_CGN013357 18549 CCAACTCAAGATATGCTTAGTGGACTCTATGTCTTAACGAGTGGAAATCG 18598

Lsal_LAC008020 18548 CCAACTCAAGATATGCTTAGTGGACTCTATGTCTTAACGAGTGGAAATCG 18597

Lsat_Salinas 18601 TCGGGGTATTTGTGTAAATAGGTATAATCCATGTAATCGTAGAAACTATC 18650

Lsat_WENDEL 18601 TCGGGGTATTTGTGTAAATAGGTATAATCCATGTAATCGTAGAAACTATC 18650

Lser_US96UC23 18601 TCGGGGTATTTGTGTAAATAGGTATAATCCATGTAATCGTAGAAACTATC 18650

Lser_LAC005780 18601 TCGGGGTATTTGTGTAAATAGGTATAATCCATGTAATCGTAGAAACTATC 18650

Lvir_CGN013357 18599 TCGGGGTATTTGTGTAAATAGGTATAATCCATGTAATCGTAGAAACTATC 18648

Lsal_LAC008020 18598 TCGGGGTATTTGTGTAAATAGGTATAATCCATGTAATCGTAGAAACTATC 18647

Lsat_Salinas 18651 AAAATGAAGATAATAACTATAAGTATACAAAAAAAAAAGAACCCTTTTTT 18700

Lsat_WENDEL 18651 AAAATGAAGATAATAACTATAAGTATACAAAAAAAAAAGAACCCTTTTTT 18700

Lser_US96UC23 18651 AAAATGAAGATAATAACTATAAGTATACAAAAAAAAAAGAACCCTTTTTT 18700

Lser_LAC005780 18651 AAAATGAAGATAATAACTATAAGTATACAAAAAAAAAAGAACCCTTTTTT 18700

Lvir_CGN013357 18649 AAAATGAAGATAATAACTATAAGTATACAAAAAAAAAAGAACCCTTTTTT 18698

Lsal_LAC008020 18648 AAAATGAAGATAATAACTATAAGTATACAAAAAAAAAAGAACCCTTTTTT 18697

Lsat_Salinas 18701 TGTAATCCCTATGATGCAATTGGAGCTTATCGGCAAAAACGAATCAATTT 18750

Lsat_WENDEL 18701 TGTAATCCCTATGATGCAATTGGAGCTTATCGGCAAAAACGAATCAATTT 18750

Lser_US96UC23 18701 TGTAATCCCTATGATGCAATTGGAGCTTATCGGCAAAAACGAATCAATTT 18750

Lser_LAC005780 18701 TGTAATCCCTATGATGCAATTGGAGCTTATCGGCAAAAACGAATCAATTT 18750

Lvir_CGN013357 18699 TGTAATCCCTATGATGCAATTGGAGCTTATCGGCAAAAACGAATCAATTT 18748

Lsal_LAC008020 18698 TGTAATCCCTATGATGCAATTGGAGCTTATCGGCAAAAACGAATCAATTT 18747

Lsat_Salinas 18751 AGGTAGTCCTTTGTGGCTGCGGTGGCGACTAGATCAACGCGTTATTGCTG 18800

Lsat_WENDEL 18751 AGGTAGTCCTTTGTGGCTGCGGTGGCGACTAGATCAACGCGTTATTGCTG 18800

Lser_US96UC23 18751 AGGTAGTCCTTTGTGGCTGCGGTGGCGACTAGATCAACGCGTTATTGCTG 18800

Lser_LAC005780 18751 AGGTAGTCCTTTGTGGCTGCGGTGGCGACTAGATCAACGCGTTATTGCTG 18800

Lvir_CGN013357 18749 AGGTAGTCCTTTGTGGCTGCGGTGGCGACTAGATCAACGCGTTATTGCTG 18798

Lsal_LAC008020 18748 AGGTAGTCCTTTGTGGCTGCGGTGGCGACTAGATCAACGCGTTATTACTG 18797

Lsat_Salinas 18801 CAAGAGAAGTTCCCATCGAAATTCACTATGAATCTGTGGGGACCTATTAT 18850

Lsat_WENDEL 18801 CAAGAGAAGTTCCCATCGAAATTCACTATGAATCTGTGGGGACCTATTAT 18850

Lser_US96UC23 18801 CAAGAGAAGTTCCCATCGAAATTCACTATGAATCTGTGGGGACCTATTAT 18850

Lser_LAC005780 18801 CAAGAGAAGTTCCCATCGAAATTCACTATGAATCTGTGGGGACCTATTAT 18850

Lvir_CGN013357 18799 CAAGAGAAGTTCCCATCGAAATTCACTATGAATCTGTGGGGACCTATTAT 18848

Lsal_LAC008020 18798 CAAGAGAAGTTCCCATCGAAATTCACTATGAATCTGTGGGGACCTATTAT 18847

Lsat_Salinas 18851 GAGATTTATGGACACTATCTAATAGTACGAAGTATAAAAAAAGAAATTCT 18900

Lsat_WENDEL 18851 GAGATTTATGGACACTATCTAATAGTACGAAGTATAAAAAAAGAAATTCT 18900

Lser_US96UC23 18851 GAGATTTATGGACACTATCTAATAGTACGAAGTATAAAAAAAGAAATTCT 18900

Lser_LAC005780 18851 GAGATTTATGGACACTATCTAATAGTACGAAGTATAAAAAAAGAAATTCT 18900

Lvir_CGN013357 18849 GAGATTTATGGACACTATCTAATAGTACGAAGTATAAAAAAAGAAATTCT 18898

Lsal_LAC008020 18848 GAGATTTATGGACACTATCTAATAGTACGAAGTATAAAAAAAGAAATTCT 18897

Lsat_Salinas 18901 TTATATATATATTCGAACCACTCTTGGTCATATTTCCCTTTATCGAGAAA 18950

Lsat_WENDEL 18901 TTATATATATATTCGAACCACTCTTGGTCATATTTCCCTTTATCGAGAAA 18950

Lser_US96UC23 18901 TTATATATATATTCGAACCACTCTTGGTCATATTTCCCTTTATCGAGAAA 18950

Lser_LAC005780 18901 TTATATATATATTCGAACCACTCTTGGTCATATTTCCCTTTATCGAGAAA 18950

Lvir_CGN013357 18899 TTATATATATATTCGAACCACTCTTGGTCATATTTCCCTTTATCGAGAAA 18948

Lsal_LAC008020 18898 TTATATATATATTCGAACCACTCTTGGTCATATTTCCCTTTATCGAGAAA 18947

Lsat_Salinas 18951 TAGAAGAAGCCATACAGGGGTTTTGGCAGGGCTGTTGTAATTCTATGCTA 19000

Lsat_WENDEL 18951 TAGAAGAAGCCATACAGGGGTTTTGGCAGGGCTGTTGTAATTCTATGCTA 19000

Lser_US96UC23 18951 TAGAAGAAGCCATACAGGGGTTTTGGCAGGGCTGTTGTAATTCTATGCTA 19000

Lser_LAC005780 18951 TAGAAGAAGCCATACAGGGGTTTTGGCAGGGCTGTTGTAATTCTATGCTA 19000

Lvir_CGN013357 18949 TAGAAGAAGCCATACAGGGGTTTTGGCAGGGCTGTTGTAATTCTATGCTA 18998

Lsal_LAC008020 18948 TAGAAGAAGCCATACAGGGGTTTTGGCAGGGCTGTTGTAATTCTATGCTA 18997

Lsat_Salinas 19001 CCAACGGGAATTCGAGTCTCTCCGGGTTAACTAGGACCATAAGTGCAGAA 19050

Lsat_WENDEL 19001 CCAACGGGAATTCGAGTCTCTCCGGGTTAACTAGGACCATAAGTGCAGAA 19050

Lser_US96UC23 19001 CCAACGGGAATTCGAGTCTCTCCGGGTTAACTAGGACCATAAGTGCAGAA 19050

Lser_LAC005780 19001 CCAACGGGAATTCGAGTCTCTCCGGGTTAACTAGGACCATAAGTGCAGAA 19050

Lvir_CGN013357 18999 CCAACGGGAATTCGAGTCTCTCCGGGTTAACTAGGACCATAAGTGCAGAA 19048

Lsal_LAC008020 18998 CCAACGGGAATTCGAGTCTCTCCGGGTTAACTAGGACCATAAGTGCAGAA 19047

Lsat_Salinas 19051 TTTCTACGTGAATCAAGATCTAGAAAAGGAAGTTTTCCAATCACTGACTC 19100

Lsat_WENDEL 19051 TTTCTACGTGAATCAAGATCTAGAAAAGGAAGTTTTCCAATCACTGACTC 19100

Lser_US96UC23 19051 TTTCTACGTGAATCAAGATCTAGAAAAGGAAGTTTTCCAATCACTGACTC 19100

Lser_LAC005780 19051 TTTCTACGTGAATCAAGATCTAGAAAAGGAAGTTTTCCAATCACTGACTC 19100

Lvir_CGN013357 19049 TTTCTACGTGAATCAAGATCTAGAAAAGGAAGTTTTCCAATCACTGACTC 19098

Lsal_LAC008020 19048 TTTATACGTGAATCAAGATCTAGAAAAGGAAGTTTTCCAATCACTGACTC 19097

Lsat_Salinas 19101 AAGCCCATTGTAAAATTCTACTCAGCGCAATATGGAGGTACTGATGGCAG 19150

Lsat_WENDEL 19101 AAGCCCATTGTAAAATTCTACTCAGCGCAATATGGAGGTACTGATGGCAG 19150

Lser_US96UC23 19101 AAGCCCATTGTAAAATTCTACTCAGCGCAATATGGAGGTACTGATGGCAG 19150

Lser_LAC005780 19101 AAGCCCATTGTAAAATTCTACTCAGCGCAATATGGAGGTACTGATGGCAG 19150

Lvir_CGN013357 19099 AAGCCCATTGTAAAATTCTACTCAGCGCAATATGGAGGTACTGATGGCAG 19148

Lsal_LAC008020 19098 AAGCCCATTGTAAAATTCTACTCAGCGCAATATGGAGGTACTGATGGCAG 19147

Lsat_Salinas 19151 AACGGCCCACTCAGGTCTTTCACAATAAAGTGATAGACGGAACTGCCATG 19200

Lsat_WENDEL 19151 AACGGCCCACTCAGGTCTTTCACAATAAAGTGATAGACGGAACTGCCATG 19200

Lser_US96UC23 19151 AACGGCCCACTCAGGTCTTTCACAATAAAGTGATAGACGGAACTGCCATG 19200

Lser_LAC005780 19151 AACGGCCCACTCAGGTCTTTCACAATAAAGTGATAGACGGAACTGCCATG 19200

Lvir_CGN013357 19149 AACGGCCCACTCAGGTCTTTCACAATAAAGTGATAGACGGAACTGCCATG 19198

Lsal_LAC008020 19148 AACGGCCCACTCAGGTCTTTCACAATAAAGTGATAGACGGAACTGCCATG 19197

Lsat_Salinas 19201 AAACGGCTTATTAGTCGATTTATTGATCACTATGGAATAGGATATACATC 19250

Lsat_WENDEL 19201 AAACGGCTTATTAGTCGATTTATTGATCACTATGGAATAGGATATACATC 19250

Lser_US96UC23 19201 AAACGGCTTATTAGTCGATTTATTGATCACTATGGAATAGGATATACATC 19250

Lser_LAC005780 19201 AAACGGCTTATTAGTCGATTTATTGATCACTATGGAATAGGATATACATC 19250

Lvir_CGN013357 19199 AAACGGCTTATTAGTCGATTTATTGATCACTATGGAATAGGATATACATC 19248

Lsal_LAC008020 19198 AAACGACTTATTAGTCGATTTATTGATCACTATGGAATAGGATATACATC 19247

Lsat_Salinas 19251 ACATATCCTGGATCAAGTAAAGACTCTGGGTTTCCGACAAGCTACCGCCG 19300

Lsat_WENDEL 19251 ACATATCCTGGATCAAGTAAAGACTCTGGGTTTCCGACAAGCTACCGCCG 19300

Lser_US96UC23 19251 ACATATCCTGGATCAAGTAAAGACTCTGGGTTTCCGACAAGCTACCGCCG 19300

Lser_LAC005780 19251 ACATATCCTGGATCAAGTAAAGACTCTGGGTTTCCGACAAGCTACCGCCG 19300

Lvir_CGN013357 19249 ACATATCCTGGATCAAGTAAAGACTCTGGGTTTCCGACAAGCTACCGCCG 19298

Lsal_LAC008020 19248 ACATATCTTAGATCAAGTAAAGACTCTGGGTTTCCGACAAGCTACCGCCG 19297

Lsat_Salinas 19301 CATCCATTTCATTAGGAATTGATGATCTTTTAACAATACCTTCTAAAAGA 19350

Lsat_WENDEL 19301 CATCCATTTCATTAGGAATTGATGATCTTTTAACAATACCTTCTAAAAGA 19350

Lser_US96UC23 19301 CATCCATTTCATTAGGAATTGATGATCTTTTAACAATACCTTCTAAAAGA 19350

Lser_LAC005780 19301 CATCCATTTCATTAGGAATTGATGATCTTTTAACAATACCTTCTAAAAGA 19350

Lvir_CGN013357 19299 CATCCATTTCATTAGGAATTGATGATCTTTTAACAATACCTTCTAAAAGA 19348

Lsal_LAC008020 19298 CATCCATTTCATTAGGAATTGATGATCTTTTAACAATACCTTCTAAAAGA 19347

Lsat_Salinas 19351 TGGCTAGTTCAAGACGCTGAACAACAAAGTTTTATTTTGGAAAAACACCA 19400

Lsat_WENDEL 19351 TGGCTAGTTCAAGACGCTGAACAACAAAGTTTTATTTTGGAAAAACACCA 19400

Lser_US96UC23 19351 TGGCTAGTTCAAGACGCTGAACAACAAAGTTTTATTTTGGAAAAACACCA 19400

Lser_LAC005780 19351 TGGCTAGTTCAAGACGCTGAACAACAAAGTTTTATTTTGGAAAAACACCA 19400

Lvir_CGN013357 19349 TGGCTAGTTCAAGACGCTGAACAACAAAGTTTTATTTTGGAAAAACACCA 19398

Lsal_LAC008020 19348 TGGCTAGTTCAAGACGCTGAACAACAAAGTTTTATTTTGGAAAAACACCA 19397

Lsat_Salinas 19401 TCATTATGGGAATGTACACGCGGTAGAAAAATTACGTCAATCGATCGAAA 19450

Lsat_WENDEL 19401 TCATTATGGGAATGTACACGCGGTAGAAAAATTACGTCAATCGATCGAAA 19450

Lser_US96UC23 19401 TCATTATGGGAATGTACACGCGGTAGAAAAATTACGTCAATCGATCGAAA 19450

Lser_LAC005780 19401 TCATTATGGGAATGTACACGCGGTAGAAAAATTACGTCAATCGATCGAAA 19450

Lvir_CGN013357 19399 TCATTATGGGAATGTACACGCGGTAGAAAAATTACGTCAATCGATCGAAA 19448

Lsal_LAC008020 19398 TCATTATGGGAATGTACACGCGGTAGAAAAATTACGTCAATCGATCGAAA 19447

Lsat_Salinas 19451 TATGGTATGCCACAAGTGAATATTTGCGACAAGAAATGAATCCTAATTTT 19500

Lsat_WENDEL 19451 TATGGTATGCCACAAGTGAATATTTGCGACAAGAAATGAATCCTAATTTT 19500

Lser_US96UC23 19451 TATGGTATGCCACAAGTGAATATTTGCGACAAGAAATGAATCCTAATTTT 19500

Lser_LAC005780 19451 TATGGTATGCCACAAGTGAATATTTGCGACAAGAAATGAATCCTAATTTT 19500

Lvir_CGN013357 19449 TATGGTATGCCACAAGTGAATATTTGCGACAAGAAATGAATCCTAATTTT 19498

Lsal_LAC008020 19448 TATGGTATGCCACAAGTGAATATTTGCGACAAGAAATGAATCCTAATTTT 19497

Lsat_Salinas 19501 CGGATGACCGATCCTTTTAATCCAGTCCATATAATGTCTTTTTCGGGAGC 19550

Lsat_WENDEL 19501 CGGATGACCGATCCTTTTAATCCAGTCCATATAATGTCTTTTTCGGGAGC 19550

Lser_US96UC23 19501 CGGATGACCGATCCTTTTAATCCAGTCCATATAATGTCTTTTTCGGGAGC 19550

Lser_LAC005780 19501 CGGATGACCGATCCTTTTAATCCAGTCCATATAATGTCTTTTTCGGGAGC 19550

Lvir_CGN013357 19499 CGGATGACCGATCCTTTTAATCCAGTCCATATAATGTCTTTTTCGGGAGC 19548

Lsal_LAC008020 19498 CGGATGACCGATCCTTTTAATCCAGTCCATATAATGTCTTTTTCGGGAGC 19547

Lsat_Salinas 19551 CAGAGGAAATGCATCTCAGGTACATCAATTAGTAGGTATGAGAGGATTAA 19600

Lsat_WENDEL 19551 CAGAGGAAATGCATCTCAGGTACATCAATTAGTAGGTATGAGAGGATTAA 19600

Lser_US96UC23 19551 CAGAGGAAATGCATCTCAGGTACATCAATTAGTAGGTATGAGAGGATTAA 19600

Lser_LAC005780 19551 CAGAGGAAATGCATCTCAGGTACATCAATTAGTAGGTATGAGAGGATTAA 19600

Lvir_CGN013357 19549 CAGAGGAAATGCATCTCAGGTACATCAATTAGTAGGTATGAGAGGATTAA 19598

Lsal_LAC008020 19548 CAGAGGAAATGCATCTCAGGTACATCAATTAGTAGGTATGAGAGGATTAA 19597

Lsat_Salinas 19601 TGTCGGATCCTCAAGGGCAAATGATTGATTTACCCATTCAAAGCAATTTA 19650

Lsat_WENDEL 19601 TGTCGGATCCTCAAGGGCAAATGATTGATTTACCCATTCAAAGCAATTTA 19650

Lser_US96UC23 19601 TGTCGGATCCTCAAGGGCAAATGATTGATTTACCCATTCAAAGCAATTTA 19650

Lser_LAC005780 19601 TGTCGGATCCTCAAGGGCAAATGATTGATTTACCCATTCAAAGCAATTTA 19650

Lvir_CGN013357 19599 TGTCGGATCCTCAAGGGCAAATGATTGATTTACCCATTCAAAGCAATTTA 19648

Lsal_LAC008020 19598 TGTCGGATCCTCAAGGGCAAATGATTGATTTACCCATTCAAAGCAATTTA 19647

Lsat_Salinas 19651 CGCGAAGGACTGTCTTTAACAGAATACATTATTTCTTGTTACGGAGCCCG 19700

Lsat_WENDEL 19651 CGCGAAGGACTGTCTTTAACAGAATACATTATTTCTTGTTACGGAGCCCG 19700

Lser_US96UC23 19651 CGCGAAGGACTGTCTTTAACAGAATACATTATTTCTTGTTACGGAGCCCG 19700

Lser_LAC005780 19651 CGCGAAGGACTGTCTTTAACAGAATACATTATTTCTTGTTACGGAGCCCG 19700

Lvir_CGN013357 19649 CGCGAAGGACTGTCTTTAACAGAATACATTATTTCTTGTTACGGAGCCCG 19698

Lsal_LAC008020 19648 CGCGAAGGACTGTCGTTAACAGAATACATTATTTCTTGTTACGGAGCCCG 19697

Lsat_Salinas 19701 TAAAGGGGTTGTGGATACCGCTATCCGAACATCAGATGCAGGATATCTCA 19750

Lsat_WENDEL 19701 TAAAGGGGTTGTGGATACCGCTATCCGAACATCAGATGCAGGATATCTCA 19750

Lser_US96UC23 19701 TAAAGGGGTTGTGGATACCGCTATCCGAACATCAGATGCAGGATATCTCA 19750

Lser_LAC005780 19701 TAAAGGGGTTGTGGATACCGCTATCCGAACATCAGATGCAGGATATCTCA 19750

Lvir_CGN013357 19699 TAAAGGGGTTGTGGATACCGCTATCCGAACATCAGATGCAGGATATCTCA 19748

Lsal_LAC008020 19698 TAAAGGGGTTGTGGATACCGCTATCCGAACATCAGATGCAGGATATCTCA 19747

Lsat_Salinas 19751 CGCGCAGACTTGTTGAAGTAGTTCAACACATTGTTGTACGTCGAACAGAT 19800

Lsat_WENDEL 19751 CGCGCAGACTTGTTGAAGTAGTTCAACACATTGTTGTACGTCGAACAGAT 19800

Lser_US96UC23 19751 CGCGCAGACTTGTTGAAGTAGTTCAACACATTGTTGTACGTCGAACAGAT 19800

Lser_LAC005780 19751 CGCGCAGACTTGTTGAAGTAGTTCAACACATTGTTGTACGTCGAACAGAT 19800

Lvir_CGN013357 19749 CGCGCAGACTTGTTGAAGTAGTTCAACACATTGTTGTACGTCGAACAGAT 19798

Lsal_LAC008020 19748 CGCGCAGACTTGTTGAAGTAGTTCAACACATTGTTGTACGTCGAACAGAT 19797

Lsat_Salinas 19801 TGTGGCACCGTTCGAGGTATTTCTGTAAGTCCTCGAAATGGAATGATGAC 19850

Lsat_WENDEL 19801 TGTGGCACCGTTCGAGGTATTTCTGTAAGTCCTCGAAATGGAATGATGAC 19850

Lser_US96UC23 19801 TGTGGCACCGTTCGAGGTATTTCTGTAAGTCCTCGAAATGGAATGATGAC 19850

Lser_LAC005780 19801 TGTGGCACCGTTCGAGGTATTTCTGTAAGTCCTCGAAATGGAATGATGAC 19850

Lvir_CGN013357 19799 TGTGGCACCGTTCGAGGTATTTCTGTAAGTCCTCGAAATGGAATGATGAC 19848

Lsal_LAC008020 19798 TGTGGCACCGTTCGAGGTATTTCTGTAAGTCCTCGAAATGGAATGATGAC 19847

Lsat_Salinas 19851 GGACAGGATTTTTATCCAAACATTAATTGGCCGTGTATTAGCAGATGATA 19900

Lsat_WENDEL 19851 GGACAGGATTTTTATCCAAACATTAATTGGCCGTGTATTAGCAGATGATA 19900

Lser_US96UC23 19851 GGACAGGATTTTTATCCAAACATTAATTGGCCGTGTATTAGCAGATGATA 19900

Lser_LAC005780 19851 GGACAGGATTTTTATCCAAACATTAATTGGCCGTGTATTAGCAGATGATA 19900

Lvir_CGN013357 19849 GGACAGGATTTTTATCCAAACATTAATTGGCCGTGTATTAGCAGATGATA 19898

Lsal_LAC008020 19848 GGACAGGATTTTTATCCAAACATTAATTGGCCGTGTATTAGCAGATGATA 19897

Lsat_Salinas 19901 TATATATAGGTTCGCGATGTATTGCTACTAGAAATCAAGATATTGGGGTT 19950

Lsat_WENDEL 19901 TATATATAGGTTCGCGATGTATTGCTACTAGAAATCAAGATATTGGGGTT 19950

Lser_US96UC23 19901 TATATATAGGTTCGCGATGTATTGCTACTAGAAATCAAGATATTGGGGTT 19950

Lser_LAC005780 19901 TATATATAGGTTCGCGATGTATTGCTACTAGAAATCAAGATATTGGGGTT 19950

Lvir_CGN013357 19899 TATATATAGGTTCGCGATGTATTGCTACTAGAAATCAAGATATTGGGGTT 19948

Lsal_LAC008020 19898 TATATATAGGTTCGCGATGTATTGCTACTAGAAATCAAGATATTGGGGTT 19947

Lsat_Salinas 19951 GGACTTGTCAGTAGATTCATAACTTTTAGAGCACAACCAATCTCTATTCG 20000

Lsat_WENDEL 19951 GGACTTGTCAGTAGATTCATAACTTTTAGAGCACAACCAATCTCTATTCG 20000

Lser_US96UC23 19951 GGACTTGTCAGTAGATTCATAACTTTTAGAGCACAACCAATCTCTATTCG 20000

Lser_LAC005780 19951 GGACTTGTCAGTAGATTCATAACTTTTAGAGCACAACCAATCTCTATTCG 20000

Lvir_CGN013357 19949 GGACTTGTCAGTAGATTCATAACTTTTAGAGCACAACCAATCTCTATTCG 19998

Lsal_LAC008020 19948 GGACTTGTCAGTAGATTCATAACTTTTAGAGCACACCCAATCTCTATTCG 19997

Lsat_Salinas 20001 AACCCCCTTTACTTGTAGGAGTACATCTTGGATTTGTCAATTATGTTATG 20050

Lsat_WENDEL 20001 AACCCCCTTTACTTGTAGGAGTACATCTTGGATTTGTCAATTATGTTATG 20050

Lser_US96UC23 20001 AACCCCCTTTACTTGTAGGAGTACATCTTGGATTTGTCAATTATGTTATG 20050

Lser_LAC005780 20001 AACCCCCTTTACTTGTAGGAGTACATCTTGGATTTGTCAATTATGTTATG 20050

Lvir_CGN013357 19999 AACCCCCTTTACTTGTAGGAGTACATCTTGGATTTGTCAATTATGTTATG 20048

Lsal_LAC008020 19998 AACCCCCTTTACTTGTAGGAGTACATCTTGGATTTGTCAATTATGTTATG 20047

Lsat_Salinas 20051 GCCGGAGTCCAGCGCATGACGACCTGGTCGAATTGGGAGAAGCCGTAGGT 20100

Lsat_WENDEL 20051 GCCGGAGTCCAGCGCATGACGACCTGGTCGAATTGGGAGAAGCCGTAGGT 20100

Lser_US96UC23 20051 GCCGGAGTCCAGCGCATGACGACCTGGTCGAATTGGGAGAAGCCGTAGGT 20100

Lser_LAC005780 20051 GCCGGAGTCCAGCGCATGACGACCTGGTCGAATTGGGAGAAGCCGTAGGT 20100

Lvir_CGN013357 20049 GCCGGAGTCCAGCTCATGACGACCTGGTCGAATTGGGAGAAGCCGTAGGT 20098

Lsal_LAC008020 20048 GCCGGAGTCCAGCTCATGACGACCTGGTCGAATTGGGAGAAGCCGTAGGT 20097

Lsat_Salinas 20101 ATTATTGCAGGTCAATCTATTGGAGAACCGGGCACTCAATTAACATTAAG 20150

Lsat_WENDEL 20101 ATTATTGCAGGTCAATCTATTGGAGAACCGGGCACTCAATTAACATTAAG 20150

Lser_US96UC23 20101 ATTATTGCAGGTCAATCTATTGGAGAACCGGGCACTCAATTAACATTAAG 20150

Lser_LAC005780 20101 ATTATTGCAGGTCAATCTATTGGAGAACCGGGCACTCAATTAACATTAAG 20150

Lvir_CGN013357 20099 ATTATTGCAGGTCAATCTATTGGAGAACCGGGCACTCAATTAACATTAAG 20148

Lsal_LAC008020 20098 ATTATTGCAGGTCAATCTATTGGAGAACCGGGCACTCAATTAACATTAAG 20147

Lsat_Salinas 20151 AACTTTTCATACCGGCGGAGTATTTACAGGGGGTACTGCAGAACATGTGC 20200

Lsat_WENDEL 20151 AACTTTTCATACCGGCGGAGTATTTACAGGGGGTACTGCAGAACATGTGC 20200

Lser_US96UC23 20151 AACTTTTCATACCGGCGGAGTATTTACAGGGGGTACTGCAGAACATGTGC 20200

Lser_LAC005780 20151 AACTTTTCATACCGGCGGAGTATTTACAGGGGGTACTGCAGAACATGTGC 20200

Lvir_CGN013357 20149 AACTTTTCATACCGGCGGAGTATTTACAGGGGGTACTGCAGAACATGTGC 20198

Lsal_LAC008020 20148 AACTTTTCATACCGGCGGAGTATTTACAGGGGGTACTGCAGAACATGTGC 20197

Lsat_Salinas 20201 GAGCCCCTTCTAATGGAAAAATAAAATTCAACGAGGATTTGGTTCATCCG 20250

Lsat_WENDEL 20201 GAGCCCCTTCTAATGGAAAAATAAAATTCAACGAGGATTTGGTTCATCCG 20250

Lser_US96UC23 20201 GAGCCCCTTCTAATGGAAAAATAAAATTCAACGAGGATTTGGTTCATCCG 20250

Lser_LAC005780 20201 GAGCCCCTTCTAATGGAAAAATAAAATTCAACGAGGATTTGGTTCATCCG 20250

Lvir_CGN013357 20199 GAGCCCCTTCTAATGGAAAAATAAAATTCAACGAGGATTTGGTTCATCCG 20248

Lsal_LAC008020 20198 GAGCCCCTTCTAATGGAAAAATAAAATTCAACGAGGATTTGGTTCATCCG 20247

Lsat_Salinas 20251 ACACGTACACGTCATGGACATCCTGCTTTTCTATGTTCTAGAGACTTGTA 20300

Lsat_WENDEL 20251 ACACGTACACGTCATGGACATCCTGCTTTTCTATGTTCTAGAGACTTGTA 20300

Lser_US96UC23 20251 ACACGTACACGTCATGGACATCCTGCTTTTCTATGTTCTAGAGACTTGTA 20300

Lser_LAC005780 20251 ACACGTACACGTCATGGACATCCTGCTTTTCTATGTTCTAGAGACTTGTA 20300

Lvir_CGN013357 20249 ACACGTACACGTCATGGACATCCTGCTTTTCTATGTTCTAGAGACTTGTA 20298

Lsal_LAC008020 20248 ACACGTACACGTCATGGACATCCTGCTTTTCTATGTTCTAGAGACTTGTA 20297

Lsat_Salinas 20301 TGTAACTATTGAGAGTGAAGATATTATACACAATGTGTGTATTCCGCCCA 20350

Lsat_WENDEL 20301 TGTAACTATTGAGAGTGAAGATATTATACACAATGTGTGTATTCCGCCCA 20350

Lser_US96UC23 20301 TGTAACTATTGAGAGTGAAGATATTATACACAATGTGTGTATTCCGCCCA 20350

Lser_LAC005780 20301 TGTAACTATTGAGAGTGAAGATATTATACACAATGTGTGTATTCCGCCCA 20350

Lvir_CGN013357 20299 TGTAACTATTGAGAGTGAAGATATTATACACAATGTGTGTATTCCGCCCA 20348

Lsal_LAC008020 20298 TGTAACTATTGAGAGTGAAGATATTATACACAATGTGTGTATTCCGCCCA 20347

Lsat_Salinas 20351 AAAGTTTTCTTTTAGTTCAAAACGATCAATATGTAGAATCAGAACAAGTG 20400

Lsat_WENDEL 20351 AAAGTTTTCTTTTAGTTCAAAACGATCAATATGTAGAATCAGAACAAGTG 20400

Lser_US96UC23 20351 AAAGTTTTCTTTTAGTTCAAAACGATCAATATGTAGAATCAGAACAAGTG 20400

Lser_LAC005780 20351 AAAGTTTTCTTTTAGTTCAAAACGATCAATATGTAGAATCAGAACAAGTG 20400

Lvir_CGN013357 20349 AAAGTTTTCTTTTAGTTCAAAACGATCAATATGTAGAATCAGAACAAGTG 20398

Lsal_LAC008020 20348 AAAGTTTTCTTTTAGTTCAAAACGATCAATATGTAGAATCAGAACAAGTG 20397

Lsat_Salinas 20401 ATTGCTGAGATTCGCGCGAGAACCTCCACTTTGAATTTGAAAGAGAAGGT 20450

Lsat_WENDEL 20401 ATTGCTGAGATTCGCGCGAGAACCTCCACTTTGAATTTGAAAGAGAAGGT 20450

Lser_US96UC23 20401 ATTGCTGAGATTCGCGCGAGAACCTCCACTTTGAATTTGAAAGAGAAGGT 20450

Lser_LAC005780 20401 ATTGCTGAGATTCGCGCGAGAACCTCCACTTTGAATTTGAAAGAGAAGGT 20450

Lvir_CGN013357 20399 ATTGCTGAGATTCGCGCGAGAACCTCCACTTTGAATTTGAAAGAGAAGGT 20448

Lsal_LAC008020 20398 ATTGCTGAGATTCGCGCGAGAACCTCCACTTTGAATTTGAAAGAGAAGGT 20447

Lsat_Salinas 20451 TCGAAAACATATTTATTCTGACTCAGAAGGCGAAATGCACTGGAATACTG 20500

Lsat_WENDEL 20451 TCGAAAACATATTTATTCTGACTCAGAAGGCGAAATGCACTGGAATACTG 20500

Lser_US96UC23 20451 TCGAAAACATATTTATTCTGACTCAGAAGGCGAAATGCACTGGAATACTG 20500

Lser_LAC005780 20451 TCGAAAACATATTTATTCTGACTCAGAAGGCGAAATGCACTGGAATACTG 20500

Lvir_CGN013357 20449 TCGAAAACATATTTATTCTGACTCAGAAGGCGAAATGCACTGGAATACTG 20498

Lsal_LAC008020 20448 TCGAAAACATATTTATTCTGACTCAGAAGGCGAAATGCACTGGAATACTG 20497

Lsat_Salinas 20501 ATGTGTACCATGCACCTGAATTTACATATGGTAATATTCATCTCTTACCA 20550

Lsat_WENDEL 20501 ATGTGTACCATGCACCTGAATTTACATATGGTAATATTCATCTCTTACCA 20550

Lser_US96UC23 20501 ATGTGTACCATGCACCTGAATTTACATATGGTAATATTCATCTCTTACCA 20550

Lser_LAC005780 20501 ATGTGTACCATGCACCTGAATTTACATATGGTAATATTCATCTCTTACCA 20550

Lvir_CGN013357 20499 ATGTGTACCATGCACCTGAATTTACATATGGTAATATTCATCTCTTACCA 20548

Lsal_LAC008020 20498 ATGTGTACCATGCACCTGAATTTACATATGGTAATATTCATCTCTTACCA 20547

Lsat_Salinas 20551 AAAACAAGTCATTTATGGATATTATTAGGGGAGCCCTGGAGATACAGTCT 20600

Lsat_WENDEL 20551 AAAACAAGTCATTTATGGATATTATTAGGGGAGCCCTGGAGATACAGTCT 20600

Lser_US96UC23 20551 AAAACAAGTCATTTATGGATATTATTAGGGGAGCCCTGGAGATACAGTCT 20600

Lser_LAC005780 20551 AAAACAAGTCATTTATGGATATTATTAGGGGAGCCCTGGAGATACAGTCT 20600

Lvir_CGN013357 20549 AAAACAAGTCATTTATGGATATTATTAGGGGAGCCCTGGAGATACAGTCT 20598

Lsal_LAC008020 20548 AAAACAAGTCATTTATGGATATTATTAGGGGAGCCCTGGAGATACAGTCT 20597

Lsat_Salinas 20601 AGGCCCTTGTTCGATCCACAAGGATCAAGATCAAATGAACGCTTATTCTC 20650

Lsat_WENDEL 20601 AGGCCCTTGTTCGATCCACAAGGATCAAGATCAAATGAACGCTTATTCTC 20650

Lser_US96UC23 20601 AGGCCCTTGTTCGATCCACAAGGATCAAGATCAAATGAACGCTTATTCTC 20650

Lser_LAC005780 20601 AGGCCCTTGTTCGATCCACAAGGATCAAGATCAAATGAACGCTTATTCTC 20650

Lvir_CGN013357 20599 AGGCCCTTGTTCGATCCACAAGGATCAAGATCAAATGAACGCTTATTCTC 20648

Lsal_LAC008020 20598 AGGCCCTTGTTCGATCCACAAGGATCAAGATCAAATGAACGCTTATTCTC 20647

Lsat_Salinas 20651 TTTCTGTCAAGCCAAGATATATTGCTAACCCCTCAGTAACTAATAATCAA 20700

Lsat_WENDEL 20651 TTTCTGTCAAGCCAAGATATATTGCTAACCCCTCAGTAACTAATAATCAA 20700

Lser_US96UC23 20651 TTTCTGTCAAGCCAAGATATATTGCTAACCCCTCAGTAACTAATAATCAA 20700

Lser_LAC005780 20651 TTTCTGTCAAGCCAAGATATATTGCTAACCCCTCAGTAACTAATAATCAA 20700

Lvir_CGN013357 20649 TTTCTGTCAAGCCAAGATATATTGCTAACCCCTCAGTAACTAATAATCAA 20698

Lsal_LAC008020 20648 TTTCTGTCAAGCCAAGATATATTGCTAACCCCTCAGTAACTAATAATCAA 20697

Lsat_Salinas 20701 GTGAGACACAAATTTTTTAGTTCGTATTTTTCTGGTAAAAATCAAAAAGG 20750

Lsat_WENDEL 20701 GTGAGACACAAATTTTTTAGTTCGTATTTTTCTGGTAAAAATCAAAAAGG 20750

Lser_US96UC23 20701 GTGAGACACAAATTTTTTAGTTCGTATTTTTCTGGTAAAAATCAAAAAGG 20750

Lser_LAC005780 20701 GTGAGACACAAATTTTTTAGTTCGTATTTTTCTGGTAAAAATCAAAAAGG 20750

Lvir_CGN013357 20699 GTGAGACACAAATTTTTTAGTTCGTATTTTTCTGGTAAAAATCAAAAAGG 20748

Lsal_LAC008020 20698 GTGAGACACAAATTTTTTAGTTCGTATTTTTCTGGTAAAAATCAAAAAGG 20747

Lsat_Salinas 20751 AGATAGGATTCCTGATTGTTCAGAACTGAATCGAATGACATGTACGGATC 20800

Lsat_WENDEL 20751 AGATAGGATTCCTGATTGTTCAGAACTGAATCGAATGACATGTACGGATC 20800

Lser_US96UC23 20751 AGATAGGATTCCTGATTGTTCAGAACTGAATCGAATGACATGTACGGATC 20800

Lser_LAC005780 20751 AGATAGGATTCCTGATTATTCAGAACTGAATCGAATGACATGTACGGATC 20800

Lvir_CGN013357 20749 AGATAGGATTCCTGATTATTCAGAACTGAATCGAATGACATGTACGGATC 20798

Lsal_LAC008020 20748 AGATAGGATTCCTGATTATTCAGAACTGAATCGAATGACATGTACGGATC 20797

Lsat_Salinas 20801 ATTCTAATCTCAGATATCCGGCCATTCTCGACGGTAATTCTGATTTATTG 20850

Lsat_WENDEL 20801 ATTCTAATCTCAGATATCCGGCCATTCTCGACGGTAATTCTGATTTATTG 20850

Lser_US96UC23 20801 ATTCTAATCTCAGATATCCGGCCATTCTCGACGGTAATTCTGATTTATTG 20850

Lser_LAC005780 20801 ATTCTAATCTCAGATATCCGGCCATTCTCGACGGTAATTCTGATTTATTG 20850

Lvir_CGN013357 20799 ATTGTAATCTCAGATATCCGGCCATTCTCGACGGTAATTCTGATTTATTG 20848

Lsal_LAC008020 20798 ATTGTAATCTCAGATATCCGGCCATTCTCGACGGTAATTCTGATTTATTG 20847

Lsat_Salinas 20851 GCAAAGAGGCGAAGAAATAGATTCATCATCCCACTCGAATCGATTCAAGA 20900

Lsat_WENDEL 20851 GCAAAGAGGCGAAGAAATAGATTCATCATCCCACTCGAATCGATTCAAGA 20900

Lser_US96UC23 20851 GCAAAGAGGCGAAGAAATAGATTCATCATCCCACTCGAATCGATTCAAGA 20900

Lser_LAC005780 20851 GCAAAGAGGCGAAGAAATAGATTCATCATCCCACTCGAATCGATTCAAGA 20900

Lvir_CGN013357 20849 GCAAAGAGGCGAAGAAATAGATTCATCATCCCACTCGAATCGATTCAAGA 20898

Lsal_LAC008020 20848 GCAAAGAGGCGAAGAAATAGATTCATCATCCCACTCGAATCGATTCAAGA 20897

Lsat_Salinas 20901 AGGCGAGAACCAACTAATACCTTCTTCAGGTATCTCAATGGAAATACCCA 20950

Lsat_WENDEL 20901 AGGCGAGAACCAACTAATACCTTCTTCAGGTATCTCAATGGAAATACCCA 20950

Lser_US96UC23 20901 AGGCGAGAACCAACTAATACCTTCTTCAGGTATCTCAATGGAAATACCCA 20950

Lser_LAC005780 20901 AGGCGAGAACCAACTAATACCTTCTTCAGGTATCTCAATGGAAATACCCA 20950

Lvir_CGN013357 20899 AGGCGAGAACCAACTAATACCTTCTTCAGGTATCTCAATGGAAATACCCA 20948

Lsal_LAC008020 20898 AGGCGAGAACCAACTAATACCTTCTTCAGGTATCTCAATGGAAATACCCA 20947

Lsat_Salinas 20951 GAAATGGTATTCTCCGTAGAAATAGTATTCTTGCTTATTTCGATGATCCT 21000

Lsat_WENDEL 20951 GAAATGGTATTCTCCGTAGAAATAGTATTCTTGCTTATTTCGATGATCCT 21000

Lser_US96UC23 20951 GAAATGGTATTCTCCGTAGAAATAGTATTCTTGCTTATTTCGATGATCCT 21000

Lser_LAC005780 20951 GAAATGGTATTCTCCGTAGAAATAGTATTCTTGCTTATTTCGATGATCCT 21000

Lvir_CGN013357 20949 GAAATGGTATTCTCCGTAGAAATAGTATTCTTGCTTATTTCGATGATCCT 20998

Lsal_LAC008020 20948 GAAATGGTATTCTCCGTAGAAATAGTATTCTTGCTTATTTCGATGATCCT 20997

Lsat_Salinas 21001 CGATATATAAGAAAGAGCTCGGGACTTACTAAATATGAGACTCGAGAACT 21050

Lsat_WENDEL 21001 CGATATATAAGAAAGAGCTCGGGACTTACTAAATATGAGACTCGAGAACT 21050

Lser_US96UC23 21001 CGATATATAAGAAAGAGCTCGGGACTTACTAAATATGAGACTCGAGAACT 21050

Lser_LAC005780 21001 CGATATATAAGAAAGAGCTCGGGACTTACTAAATATGAGACTCGAGAACT 21050

Lvir_CGN013357 20999 CGATATATAAGAAAGAGCTCGGGACTTACTAAATATGAGACTCGAGAACT 21048

Lsal_LAC008020 20998 CGATATATAAGAAAGAGCTCGGGACTTACTAAATATGAGACTCGAGAACT 21047

Lsat_Salinas 21051 AAATTCAATCGTCAACGAAGAGAATTTGATTGAGTATCGAGGAGTCAAGG 21100

Lsat_WENDEL 21051 AAATTCAATCGTCAACGAAGAGAATTTGATTGAGTATCGAGGAGTCAAGG 21100

Lser_US96UC23 21051 AAATTCAATCGTCAACGAAGAGAATTTGATTGAGTATCGAGGAGTCAAGG 21100

Lser_LAC005780 21051 AAATTCAATCGTCAACGAAGAGAATTTGATTGAGTATCGAGGAGTCAAGG 21100

Lvir_CGN013357 21049 AAATTCAATCGTCAACGAAGAGAATTTGATTGAGTATCGAGGAGTCAAGG 21098

Lsal_LAC008020 21048 AAATTCAATCGTCAACGAAGAGAATTTGATTGAGTATCGAGGAGTCAAGG 21097

Lsat_Salinas 21101 TATTTTGGCCAAAATACCAAAAGGAAGTAAATCCATTTTTTTTTATTCCC 21150

Lsat_WENDEL 21101 TATTTTGGCCAAAATACCAAAAGGAAGTAAATCCATTTTTTTTTATTCCC 21150

Lser_US96UC23 21101 TATTTTGGCCAAAATACCAAAAGGAAGTAAATCCATTTTTTTTTATTCCC 21150

Lser_LAC005780 21101 TATTTTGGCCAAAATACCAAAAGGAAGTAAATCCATTTTTTTTTATTCCC 21150

Lvir_CGN013357 21099 TATTTTGGCCAAAATACCAAAAGGAAGTAAATCCATTTTTTTTTATTCCC 21148

Lsal_LAC008020 21098 TATTTTGGCCAAAATACCAAAAGGAAGTAAATCCATTTTTTTTTATTCCC 21147

Lsat_Salinas 21151 GTGGAAGTCCACATTTTGTCCGAATCTTCTTCCATAATGGTACGGCACAA 21200

Lsat_WENDEL 21151 GTGGAAGTCCACATTTTGTCCGAATCTTCTTCCATAATGGTACGGCACAA 21200

Lser_US96UC23 21151 GTGGAAGTCCACATTTTGTCCGAATCTTCTTCCATAATGGTACGGCACAA 21200

Lser_LAC005780 21151 GTAGAAGTCCACATTTTGTCCGAATCTTCTTCCATAATGGTACGGCACAA 21200

Lvir_CGN013357 21149 GTGGAAGTCCACATTTTGTCCGAATCTTCTTCCATAATGGTACGGCACAA 21198

Lsal_LAC008020 21148 GTGGAAGTCCACATTTTGTCCGAATCTTCTTCCATAATGGTACGGCACAA 21197

Lsat_Salinas 21201 TAGTATTATTGGGGCAGATACACAAATCACTTTCAATAGAAGAAGTCGGG 21250

Lsat_WENDEL 21201 TAGTATTATTGGGGCAGATACACAAATCACTTTCAATAGAAGAAGTCGGG 21250

Lser_US96UC23 21201 TAGTATTATTGGGGCAGATACACAAATCACTTTCAATAGAAGAAGTCGGG 21250

Lser_LAC005780 21201 TAGTATTATTGGGGCAGATACACAAATCACTTTCAATAGAAGAAGTCGGG 21250

Lvir_CGN013357 21199 TAGTATTATTGGGGCAGATACACAAATCACTTTCAATAGAAGAAGTCGGG 21248

Lsal_LAC008020 21198 TAGTATTATTGGGGCAGATACACAAATCACTTTCAATAGAAGAAGTCGGG 21247

Lsat_Salinas 21251 TAGGCGGATTGGTCCGAGTGAAGAAAAAAGCAGAAAAAATGAAACTGATA 21300

Lsat_WENDEL 21251 TAGGCGGATTGGTCCGAGTGAAGAAAAAAGCAGAAAAAATGAAACTGATA 21300

Lser_US96UC23 21251 TAGGCGGATTGGTCCGAGTGAAGAAAAAAGCAGAAAAAATGAAACTGATA 21300

Lser_LAC005780 21251 TAGGCGGATTGGTCCGAGTGAAGAAAAAAGCAGAAAAAATGAAACTGATA 21300

Lvir_CGN013357 21249 TAGGCGGATTGGTCCGAGTGAAGAAAAAAGCAGAAAAAATGAAACTGATA 21298

Lsal_LAC008020 21248 TAGGCGGATTGGTCCGAGTGAAGAAAAAAGCAGAAAAAATGAAACTGATA 21297

Lsat_Salinas 21301 ATCTTTTCTGGAGATATCCATTTTCCTGGAAAGACAAATAAGGCATTCCG 21350

Lsat_WENDEL 21301 ATCTTTTCTGGAGATATCCATTTTCCTGGAAAGACAAATAAGGCATTCCG 21350

Lser_US96UC23 21301 ATCTTTTCTGGAGATATCCATTTTCCTGGAAAGACAAATAAGGCATTCCG 21350

Lser_LAC005780 21301 ATCTTTTCTGGAGATATCCATTTTCCTGGAAAGACAAATAAGGCATTCCG 21350

Lvir_CGN013357 21299 ATCTTTTCTGGAGATATCCATTTTCCTGGAAAGACAAATAAGGCATTCCG 21348

Lsal_LAC008020 21298 ATCTTTTCTGGAGATATCCATTTTCCTGGAAAGACAAATAAGGCATTCCG 21347

Lsat_Salinas 21351 ATTGATACCGCCAGGAGGGGGAAAACCAAATTCCAAAGAATACAAAAAAT 21400

Lsat_WENDEL 21351 ATTGATACCGCCAGGAGGGGGAAAACCAAATTCCAAAGAATACAAAAAAT 21400

Lser_US96UC23 21351 ATTGATACCGCCAGGAGGGGGAAAACCAAATTCCAAAGAATACAAAAAAT 21400

Lser_LAC005780 21351 ATTGATACCGCCAGGAGGGGGAAAACCAAATTCCAAAGAATACAAAAAAT 21400

Lvir_CGN013357 21349 ATTGATACCGCCAGGAGGGGGAAAACCAAATTCCAAAGAATACAAAAAAT 21398

Lsal_LAC008020 21348 ATTGATACCGCCAGGAGGGGGAAAACCAAATTCCAAAGAATACAAAAAAT 21397

Lsat_Salinas 21401 TGAAAAATTGGCTCTATATCCAACGAATGAAACTTTCCAGGTATGAAAAA 21450

Lsat_WENDEL 21401 TGAAAAATTGGCTCTATATCCAACGAATGAAACTTTCCAGGTATGAAAAA 21450

Lser_US96UC23 21401 TGAAAAATTGGCTCTATATCCAACGAATGAAACTTTCCAGGTATGAAAAA 21450

Lser_LAC005780 21401 TGAAAAATTGGCTCTATATCCAACGAATGAAACTTTCCAGGTATGAAAAA 21450

Lvir_CGN013357 21399 TGAAAAATTGGCTCTATATCCAACGAATGAAACTTTCCAGGTATGAAAAA 21448

Lsal_LAC008020 21398 TGAAAAATTGGCTCTATATCCAACGAATGAAACTTTCCAGGTATGAAAAA 21447

Lsat_Salinas 21451 AAGTATTTTGTTTTGGTTCAACCTGTAGTCCCATATAAAAAAACGGATGG 21500

Lsat_WENDEL 21451 AAGTATTTTGTTTTGGTTCAACCTGTAGTCCCATATAAAAAAACGGATGG 21500

Lser_US96UC23 21451 AAGTATTTTGTTTTGGTTCAACCTGTAGTCCCATATAAAAAAACGGATGG 21500

Lser_LAC005780 21451 AAGTATTTTGTTTTGGTTCAACCTGTAGTCCCATATAAAAAAACGGATGG 21500

Lvir_CGN013357 21449 AAGTATTTTGTTTTGGTTCAACCTGTAGTCCCATATAAAAAAACGGATGG 21498

Lsal_LAC008020 21448 AAGTATTTTGTTTTGGTTCAACCTGTAGTCCCATATAAAAAAACGGATGG 21497

Lsat_Salinas 21501 TATAAATTTAGGAAGACTTTTCCCGCCGGATCTCTTGCAGGAAAGTGATA 21550

Lsat_WENDEL 21501 TATAAATTTAGGAAGACTTTTCCCGCCGGATCTCTTGCAGGAAAGTGATA 21550

Lser_US96UC23 21501 TATAAATTTAGGAAGACTTTTCCCGCCGGATCTCTTGCAGGAAAGTGATA 21550

Lser_LAC005780 21501 TATAAATTTAGGAAGACTTTTCCCGCCGGATCTCTTGCAGGAAAGTGATA 21550

Lvir_CGN013357 21499 TATAAATTTAGGAAGACTTTTCCCGCCGGATCTCTTGCAGGAAAGTGATA 21548

Lsal_LAC008020 21498 TATAAATTTAGGAAGACTTTTCCCGCCGGATCTCTTGCAGGAAAGTGATA 21547

Lsat_Salinas 21551 ATCTACAACTTCGAGTTGTCAATTATATCCTTTATTACGACCCAATTCTT 21600

Lsat_WENDEL 21551 ATCTACAACTTCGAGTTGTCAATTATATCCTTTATTACGACCCAATTCTT 21600

Lser_US96UC23 21551 ATCTACAACTTCGAGTTGTCAATTATATCCTTTATTACGACCCAATTCTT 21600

Lser_LAC005780 21551 ATCTACAACTTCGAGTTGTCAATTATATCCTTTATTACGACCCAATTCTT 21600

Lvir_CGN013357 21549 ATCTACAACTTCGAGTTGTCAATTATATCCTTTATTACGACCCAATTCTT 21598

Lsal_LAC008020 21548 ATCTACAACTTCGAGTTGTCAATTATATCCTTTATTACGACCCAATTCTT 21597

Lsat_Salinas 21601 GAAATTTGGGACACAAGTATTCAATTAGTTCGGACTTCTTTAGTGTTGAA 21650

Lsat_WENDEL 21601 GAAATTTGGGACACAAGTATTCAATTAGTTCGGACTTCTTTAGTGTTGAA 21650

Lser_US96UC23 21601 GAAATTTGGGACACAAGTATTCAATTAGTTCGGACTTCTTTAGTGTTGAA 21650

Lser_LAC005780 21601 GAAATTTGGGACACAAGTATTCAATTAGTTCGGACTTCTTTAGTGTTGAA 21650

Lvir_CGN013357 21599 GAAATTTGGGACACAAGTATTCAATTAGTTCGGACTTCTTTAGTGTTGAA 21648

Lsal_LAC008020 21598 GAAATTTGGGACACAAGTATTCAGTTAGTTCGGACTTCTTTAGTGTTGAA 21647

Lsat_Salinas 21651 TTGGGACCAAGACAAAAAAATCGAAAAGGCCTGTGCTTCCTTTGTTGAAA 21700

Lsat_WENDEL 21651 TTGGGACCAAGACAAAAAAATCGAAAAGGCCTGTGCTTCCTTTGTTGAAA 21700

Lser_US96UC23 21651 TTGGGACCAAGACAAAAAAATCGAAAAGGCCTGTGCTTCCTTTGTTGAAA 21700

Lser_LAC005780 21651 TTGGGACCAAGACAAAAAAATCGAAAAGGCCTGTGCTTCCTTTGTTGAAA 21700

Lvir_CGN013357 21649 TTGGGACCAAGACAAAAAAATCGAAAAGGCCTGTGCTTCCTTTGTTGAAA 21698

Lsal_LAC008020 21648 TTGGGACCAAGACAAAAAAATCGAAAAGGCCTGTGCTTCCTTTGTTGAAA 21697

Lsat_Salinas 21701 TAAGGACAAATGGTTTGCTTAGATATTTTCTAAGAATCGACTTAGCTAAG 21750

Lsat_WENDEL 21701 TAAGGACAAATGGTTTGCTTAGATATTTTCTAAGAATCGACTTAGCTAAG 21750

Lser_US96UC23 21701 TAAGGACAAATGGTTTGCTTAGATATTTTCTAAGAATCGACTTAGCTAAG 21750

Lser_LAC005780 21701 TAAGGACAAATGGTTTGCTTAGATATTTTCTAAGAATCGACTTAGCTAAG 21750

Lvir_CGN013357 21699 TAAGGACAAATGGTTTGCTTAGATATTTTCTAAGAATCGACTTAGCTAAG 21748

Lsal_LAC008020 21698 TAAGGACAAATGGTTTGCTTAGATATTTTCTAAGAATCGACTTAGCTAAG 21747

Lsat_Salinas 21751 TCACCTATTTCTTATACCGGAAAAAGGAACGATCTGTCGGGTTCAGGATT 21800

Lsat_WENDEL 21751 TCACCTATTTCTTATACCGGAAAAAGGAACGATCTGTCGGGTTCAGGATT 21800

Lser_US96UC23 21751 TCACCTATTTCTTATACCGGAAAAAGGAACGATCTGTCGGGTTCAGGATT 21800

Lser_LAC005780 21751 TCACCTATTTCTTATACCGGAAAAAGGAACGATCTGTCGGGTTCAGGATT 21800

Lvir_CGN013357 21749 TCACCTATTTCTTATACCGGAAAAAGGAACGATCTGTCGGGTTCAGGATT 21798

Lsal_LAC008020 21748 TCACCTATTTCTTATACCGGAAAAAGGAACGATCTGTCGGGTTCAGGATT 21797

Lsat_Salinas 21801 GATCTCTGAGAATGGATCAGATCGCGCTAATGTCAATCCATTTTCTTCCA 21850

Lsat_WENDEL 21801 GATCTCTGAGAATGGATCAGATCGCGCTAATGTCAATCCATTTTCTTCCA 21850

Lser_US96UC23 21801 GATCTCTGAGAATGGATCAGATCGCGCTAATGTCAATCCATTTTCTTCCA 21850

Lser_LAC005780 21801 GATCTCTGAGAATGGATCAGATCGCGCTAATGTCAATCCATTTTCTTCCA 21850

Lvir_CGN013357 21799 GATCTCTGAGAATGGATCAGATCGCGTTAATGTCAATCCATTTTCTTCCA 21848

Lsal_LAC008020 21798 GATCTCTGAGAATGGATCAGATCGCGCTAATGTCAATCCATTTTCTTCCA 21847

Lsat_Salinas 21851 TTTATTCCTATTCCAAGTCAAGGATTAAAGAATCCCTTAATCCAAATCAA 21900

Lsat_WENDEL 21851 TTTATTCCTATTCCAAGTCAAGGATTAAAGAATCCCTTAATCCAAATCAA 21900

Lser_US96UC23 21851 TTTATTCCTATTCCAAGTCAAGGATTAAAGAATCCCTTAATCCAAATCAA 21900

Lser_LAC005780 21851 TTTATTCCTATTCCAAGTCAAGGATTAAAGAATCCCTTAATCCAAATCAA 21900

Lvir_CGN013357 21849 TTTATTCCTATTCCAAGTCAAGGATTAAAGAATCCCTTAATCCAAATCAA 21898

Lsal_LAC008020 21848 TTTATTCCTATTCCAAGTCAAGGATTAAAGAATCCCTTAATCCAAATCAA 21897

Lsat_Salinas 21901 GGAACTATCCATACGTTGTTGAATCGAAATAAGGAATCTCAATCTTTGAT 21950

Lsat_WENDEL 21901 GGAACTATCCATACGTTGTTGAATCGAAATAAGGAATCTCAATCTTTGAT 21950

Lser_US96UC23 21901 GGAACTATCCATACGTTGTTGAATCGAAATAAGGAATCTCAATCTTTGAT 21950

Lser_LAC005780 21901 GGAACTATCCATACGTTGTTGAATCGAAATAAGGAATCTCAATCTTTGAT 21950

Lvir_CGN013357 21899 GGAACTATCCATACGTTGTTGAATCGAAATAAGGAATCTCAATCTTTGAT 21948

Lsal_LAC008020 21898 GGAACTATCCATACGTTGTTGAATCGAAATAAGGAATCTCAATCTTTGAT 21947

Lsat_Salinas 21951 AATTTTGTCATCATCCAATTGTTTTCGAATAGGCCCATTCAACGATGTAA 22000

Lsat_WENDEL 21951 AATTTTGTCATCATCCAATTGTTTTCGAATAGGCCCATTCAACGATGTAA 22000

Lser_US96UC23 21951 AATTTTGTCATCATCCAATTGTTTTCGAATAGGCCCATTCAACGATGTAA 22000

Lser_LAC005780 21951 AATTTTGTCATCATCCAATTGTTTTCGAATAGGCCCATTCAACGATGTAA 22000

Lvir_CGN013357 21949 AATTTTGTCATCATCCAATTGTTTTCGAATAGGCCCATTCAACGATGTAA 21998

Lsal_LAC008020 21948 AATTTTGTCATCATCCAATTGTTTTCGAATAGGCCCATTCAACGATGTAA 21997

Lsat_Salinas 22001 AATCTCCCAATGTGATAAAAGAATCAATCAAAAAGAACCCCCTAATTCCA 22050

Lsat_WENDEL 22001 AATCTCCCAATGTGATAAAAGAATCAATCAAAAAGAACCCCCTAATTCCA 22050

Lser_US96UC23 22001 AATCTCCCAATGTGATAAAAGAATCAATCAAAAAGAACCCCCTAATTCCA 22050

Lser_LAC005780 22001 AATCTCCCAATGTGATAAAAGAATCAATCAAAAAGAACCCCCTAATTCCA 22050

Lvir_CGN013357 21999 AATCTCCCAATGTGATAAAAGAATCAATCAAAAAGAACCCCCTAATTCCA 22048

Lsal_LAC008020 21998 AATCTCCCAATGCGATAAAAGAATCAATCAAAAAGAACCCCCTAATTCCA 22047

Lsat_Salinas 22051 ATTAGGAATTCGTTGGGCCCGTTAGGAACAGGTTTTCCAATTTATAATTT 22100

Lsat_WENDEL 22051 ATTAGGAATTCGTTGGGCCCGTTAGGAACAGGTTTTCCAATTTATAATTT 22100

Lser_US96UC23 22051 ATTAGGAATTCGTTGGGCCCGTTAGGAACAGGTTTTCCAATTTATAATTT 22100

Lser_LAC005780 22051 ATTAGGAATTCGTTGGGCCCGTTAGGAACAGGTTTTCCAATTTATAATTT 22100

Lvir_CGN013357 22049 ATTAGGAATTCGTTGGGCCCGTTAGGAACAGGTTTTCCAATTTATAATTT 22098

Lsal_LAC008020 22048 ATTAGGAATTCGTTGGGCCCGTTAGGAACAGGTTTTCCAATTTATAATTT 22097

Lsat_Salinas 22101 TGATTTATTTTCCCATTTAATAACCCATAATCAGATCTTGGTAACTAACT 22150

Lsat_WENDEL 22101 TGATTTATTTTCCCATTTAATAACCCATAATCAGATCTTGGTAACTAACT 22150

Lser_US96UC23 22101 TGATTTATTTTCCCATTTAATAACCCATAATCAGATCTTGGTAACTAACT 22150

Lser_LAC005780 22101 TGATTTATTTTCCCATTTAATAACCCATAATCAGATCTTGGTAACTAACT 22150

Lvir_CGN013357 22099 TGATTTATTTTCCCATTTAATAACCCATAATCAGATCTTGGTAACTAACT 22148

Lsal_LAC008020 22098 TGATTTCTTTTCCCATTTAATAACCCATAATCAGATCTTGGTAACTAACT 22147

Lsat_Salinas 22151 ATTTGCAACTTGACAATTTCAAACAGATTTTTCAAATACTTAAATATTAT 22200

Lsat_WENDEL 22151 ATTTGCAACTTGACAATTTCAAACAGATTTTTCAAATACTTAAATATTAT 22200

Lser_US96UC23 22151 ATTTGCAACTTGACAATTTCAAACAGATTTTTCAAATACTTAAATATTAT 22200

Lser_LAC005780 22151 ATTTGCAACTTGACAATTTCAAACAGATTTTTCAAATACTTAAATATTAT 22200

Lvir_CGN013357 22149 ATTTGCAACTTGACAATTTCAAACAGATTTTTCAAATACTTAAATATTAT 22198

Lsal_LAC008020 22148 ATTTGCAACTTGACAATTTCAAACAGATTTTTCAAATACTTAAATATTAT 22197

Lsat_Salinas 22201 TTACTGGATGAAAATGGTCAAATTTATAATCCCTATTCATGCAGTAACAT 22250

Lsat_WENDEL 22201 TTACTGGATGAAAATGGTCAAATTTATAATCCCTATTCATGCAGTAACAT 22250

Lser_US96UC23 22201 TTACTGGATGAAAATGGTCAAATTTATAATCCCTATTCATGCAGTAACAT 22250

Lser_LAC005780 22201 TTACTGGATGAAAATGGTCAAATTTATAATCCCTATTCATGCAGTAACAT 22250

Lvir_CGN013357 22199 TTACTGGATGAAAATGGTCAAATTTATAATCCCTATTCATGCAGTAACAT 22248

Lsal_LAC008020 22198 TTACTGGATGAAAATGGTCAAATTTATAATCCCTATTCATGCAGTAACAT 22247

Lsat_Salinas 22251 CATTTTGAATCCATTCCATTTGAATTGGTATTTTCTCCATTACAATTATT 22300

Lsat_WENDEL 22251 CATTTTGAATCCATTCCATTTGAATTGGTATTTTCTCCATTACAATTATT 22300

Lser_US96UC23 22251 CATTTTGAATCCATTCCATTTGAATTGGTATTTTCTCCATTACAATTATT 22300

Lser_LAC005780 22251 CATTTTGAATCCATTCCATTTGAATTGGTATTTTCTCCATTACAATTATT 22300

Lvir_CGN013357 22249 CATTTTGAATCCATTCCATTTGAATTGGTATTTTCTCCATTACAATTATT 22298

Lsal_LAC008020 22248 CATTTTGAATCCATTTCATTTGAATTGGTATTTTCTCCATTACAATTATT 22297

Lsat_Salinas 22301 GTGAAGAGACATCTCCAATCGTTAGTCTTGGGCAGTTTCTTTGTGAAAAT 22350

Lsat_WENDEL 22301 GTGAAGAGACATCTCCAATCGTTAGTCTTGGGCAGTTTCTTTGTGAAAAT 22350

Lser_US96UC23 22301 GTGAAGAGACATCTCCAATCGTTAGTCTTGGGCAGTTTCTTTGTGAAAAT 22350

Lser_LAC005780 22301 GTGAAGAGACATCTCCAATCGTTAGTCTTGGGCAGTTTCTTTGTGAAAAT 22350

Lvir_CGN013357 22299 GTGAAGAGACATCTCCAATCGTTAGTCTTGGGCAGTTTCTTTGTGAAAAT 22348

Lsal_LAC008020 22298 GTGAAGAGACATCTCCAATCGTTAGTCTTGGGCAGTTTCTTTGTGAAAAT 22347

Lsat_Salinas 22351 GTATGTATAGCAAAAAAAGGACCGCATTTAAAATCGGGTCAAGTTCTAAT 22400

Lsat_WENDEL 22351 GTATGTATAGCAAAAAAAGGACCGCATTTAAAATCGGGTCAAGTTCTAAT 22400

Lser_US96UC23 22351 GTATGTATAGCAAAAAAAGGACCGCATTTAAAATCGGGTCAAGTTCTAAT 22400

Lser_LAC005780 22351 GTATGTATAGCAAAAAAAGGACCGCATTTAAAATCGGGTCAAGTTCTAAT 22400

Lvir_CGN013357 22349 GTATGTATAGCAAAAAAAGGACCGCATTTAAAATCGGGTCAAGTTCTAAT 22398

Lsal_LAC008020 22348 GTATGTATAGCAAAAAAAGGACCGCATTTAAAATCGGGTCAAGTTCTAAT 22397

Lsat_Salinas 22401 TGTTCAAGTTGACTCTGTGGTAATACGATCAGCTAAGCCTTATTTGGCTA 22450

Lsat_WENDEL 22401 TGTTCAAGTTGACTCTGTGGTAATACGATCAGCTAAGCCTTATTTGGCTA 22450

Lser_US96UC23 22401 TGTTCAAGTTGACTCTGTGGTAATACGATCAGCTAAGCCTTATTTGGCTA 22450

Lser_LAC005780 22401 TGTTCAAGTTGACTCTGTGGTAATACGATCAGCTAAGCCTTATTTGGCTA 22450

Lvir_CGN013357 22399 TGTTCAAGTTGACTCTGTGGTAATACGATCAGCTAAGCCTTATTTGGCTA 22448

Lsal_LAC008020 22398 TGTTCAAGTTGACTCTGTGGTAATACGATCAGCTAAGCCTTATTTGGCTA 22447

Lsat_Salinas 22451 CTCCAGGAGCAACTGTTCATGGACATTATGGGGAAATCCTTTACGAAGGC 22500

Lsat_WENDEL 22451 CTCCAGGAGCAACTGTTCATGGACATTATGGGGAAATCCTTTACGAAGGC 22500

Lser_US96UC23 22451 CTCCAGGAGCAACTGTTCATGGACATTATGGGGAAATCCTTTACGAAGGC 22500

Lser_LAC005780 22451 CTCCAGGAGCAACTGTTCATGGACATTATGGGGAAATCCTTTACGAAGGC 22500

Lvir_CGN013357 22449 CTCCAGGAGCAACTGTTCATGGACATTATGGGGAAATCCTTTACGAAGGC 22498

Lsal_LAC008020 22448 CTCCAGGAGCAACTGTTCATGGACATTATGGGGAAATCCTTTACGAAGGC 22497

Lsat_Salinas 22501 GATACATTAGTTACATTTATATATGAAAAATCAAGATCTGGTGATATAAC 22550

Lsat_WENDEL 22501 GATACATTAGTTACATTTATATATGAAAAATCAAGATCTGGTGATATAAC 22550

Lser_US96UC23 22501 GATACATTAGTTACATTTATATATGAAAAATCAAGATCTGGTGATATAAC 22550

Lser_LAC005780 22501 GATACATTAGTTACATTTATATATGAAAAATCAAGATCTGGTGATATAAC 22550

Lvir_CGN013357 22499 GATACATTAGTTACATTTATATATGAAAAATCAAGATCTGGTGATATAAC 22548

Lsal_LAC008020 22498 GATACATTAGTTACATTTATATATGAAAAATCAAGATCTGGTGATATAAC 22547

Lsat_Salinas 22551 GCAAGGTCTTCCAAAAGTGGAACAGGTGTTAGAAGTGCGTTCGATTGATT 22600

Lsat_WENDEL 22551 GCAAGGTCTTCCAAAAGTGGAACAGGTGTTAGAAGTGCGTTCGATTGATT 22600

Lser_US96UC23 22551 GCAAGGTCTTCCAAAAGTGGAACAGGTGTTAGAAGTGCGTTCGATTGATT 22600

Lser_LAC005780 22551 GCAAGGTCTTCCAAAAGTGGAACAGGTGTTAGAAGTGCGTTCGATTGATT 22600

Lvir_CGN013357 22549 GCAAGGTCTTCCAAAAGTGGAACAGGTGTTAGAAGTGCGTTCGATTGATT 22598

Lsal_LAC008020 22548 GCAAGGTCTTCCAAAAGTGGAACAGGTGTTAGAAGTGCGTTCGATTGATT 22597

Lsat_Salinas 22601 CAATATCGATGAATCTCGAAAAGAGGATTGAGGGTTGGAACAAATCTATA 22650

Lsat_WENDEL 22601 CAATATCGATGAATCTCGAAAAGAGGATTGAGGGTTGGAACAAATCTATA 22650

Lser_US96UC23 22601 CAATATCGATGAATCTCGAAAAGAGGATTGAGGGTTGGAACAAATCTATA 22650

Lser_LAC005780 22601 CAATATCGATGAATCTCGAAAAGAGGATTGAGGGTTGGAACAAATCTATA 22650

Lvir_CGN013357 22599 CAATATCGATGAATCTCGAAAAGAGGATTGAGGGTTGGAACAAATCTATA 22648

Lsal_LAC008020 22598 CAATATCGATGAATCTCGAAAAGAGGATTGAGGGTTGGAACAAATCTATA 22647

Lsat_Salinas 22651 ACAAGAATTCTTGGAATTCCTTGGGCATTCTTGATTGGTGCTGAACTAAC 22700

Lsat_WENDEL 22651 ACAAGAATTCTTGGAATTCCTTGGGCATTCTTGATTGGTGCTGAACTAAC 22700

Lser_US96UC23 22651 ACAAGAATTCTTGGAATTCCTTGGGCATTCTTGATTGGTGCTGAACTAAC 22700

Lser_LAC005780 22651 ACAAGAATTCTTGGAATTCCTTGGGCATTCTTGATTGGTGCTGAACTAAC 22700

Lvir_CGN013357 22649 ACAAGAATTCTTGGAATTCCTTGGGCATTCTTGATTGGTGCTGAACTAAC 22698

Lsal_LAC008020 22648 ACAAGAATTCTTGGAATTCCTTGGGCATTCTTGATTGGTGCTGAACTAAC 22697

Lsat_Salinas 22701 TATAGTGCAAAGTCGTATCTCTTTGGTTAATAAGGTCCAAAAGGTTTATC 22750

Lsat_WENDEL 22701 TATAGTGCAAAGTCGTATCTCTTTGGTTAATAAGGTCCAAAAGGTTTATC 22750

Lser_US96UC23 22701 TATAGTGCAAAGTCGTATCTCTTTGGTTAATAAGGTCCAAAAGGTTTATC 22750

Lser_LAC005780 22701 TATAGTGCAAAGTCGTATCTCTTTGGTTAATAAGGTCCAAAAGGTTTATC 22750

Lvir_CGN013357 22699 TATAGTGCAAAGTCGTATCTCTTTGGTTAATAAGGTCCAAAAGGTTTATC 22748

Lsal_LAC008020 22698 TATAGTGCAAAGTCGTATCTCTTTGGTTAATAAGGTCCAAAAGGTTTATC 22747

Lsat_Salinas 22751 GCTCCCAGGGGGTGCAGATACATAATAGGCATATAGAAATTATTGTACGT 22800

Lsat_WENDEL 22751 GCTCCCAGGGGGTGCAGATACATAATAGGCATATAGAAATTATTGTACGT 22800

Lser_US96UC23 22751 GCTCCCAGGGGGTGCAGATACATAATAGGCATATAGAAATTATTGTACGT 22800

Lser_LAC005780 22751 GCTCCCAGGGGGTGCAGATACATAATAGGCATATAGAAATTATTGTACGT 22800

Lvir_CGN013357 22749 GCTCCCAGGGGGTGCAGATACATAATAGGCATATAGAAATTATTGTACGT 22798

Lsal_LAC008020 22748 GCTCCCAGGGGGTGCAGATACATAATAGGCATATAGAAATTATTGTACGT 22797

Lsat_Salinas 22801 CAAATAACATCAAAAGTTTTGGTTTCAGAAGATGAAATGTCTAATGTTTT 22850

Lsat_WENDEL 22801 CAAATAACATCAAAAGTTTTGGTTTCAGAAGATGAAATGTCTAATGTTTT 22850

Lser_US96UC23 22801 CAAATAACATCAAAAGTTTTGGTTTCAGAAGATGAAATGTCTAATGTTTT 22850

Lser_LAC005780 22801 CAAATAACATCAAAAGTTTTGGTTTCAGAAGATGAAATGTCTAATGTTTT 22850

Lvir_CGN013357 22799 CAAATAACATCAAAAGTTTTGGTTTCAGAAGATGAAATGTCTAATGTTTT 22848

Lsal_LAC008020 22798 CAAATAACATCAAAAGTTTTGGTTTCAGAAGATGAAATGTCTAATGTTTT 22847

Lsat_Salinas 22851 TTCGCCCGGAGAACTAATTGGATTGTTGCGAGCGGAACGAATGGGACGCG 22900

Lsat_WENDEL 22851 TTCGCCCGGAGAACTAATTGGATTGTTGCGAGCGGAACGAATGGGACGCG 22900

Lser_US96UC23 22851 TTCGCCCGGAGAACTAATTGGATTGTTGCGAGCGGAACGAATGGGACGCG 22900

Lser_LAC005780 22851 TTCGCCCGGAGAACTAATTGGATTGTTGCGAGCGGAACGAATGGGACGCG 22900

Lvir_CGN013357 22849 TTCGCCCGGAGAACTAATTGGATTGTTGCGAGCGGAACGAATGGGACGCG 22898

Lsal_LAC008020 22848 TTCGCCCGGAGAACTAATTGGATTGTTGCGAGCGGAACGAATGGGACGCG 22897

Lsat_Salinas 22901 CTTTGGAAGAAGCGATCTGTTACCAAGCCGTCTTATTGGGAATAACAAGA 22950

Lsat_WENDEL 22901 CTTTGGAAGAAGCGATCTGTTACCAAGCCGTCTTATTGGGAATAACAAGA 22950

Lser_US96UC23 22901 CTTTGGAAGAAGCGATCTGTTACCAAGCCGTCTTATTGGGAATAACAAGA 22950

Lser_LAC005780 22901 CTTTGGAAGAAGCGATCTGTTACCAAGCCGTCTTATTGGGAATAACAAGA 22950

Lvir_CGN013357 22899 CTTTGGAAGAAGCGATCTGTTACCAAGCCGTCTTATTGGGAATAACAAGA 22948

Lsal_LAC008020 22898 CTTTGGAAGAAGCGATCTGTTACCAAGCCGTCTTATTGGGAATAACAAGA 22947

Lsat_Salinas 22951 GCATCTATGAATACTCAAAGTTTCATATCCGAAGCTAGTTTTCAAGAAAC 23000

Lsat_WENDEL 22951 GCATCTATGAATACTCAAAGTTTCATATCCGAAGCTAGTTTTCAAGAAAC 23000

Lser_US96UC23 22951 GCATCTATGAATACTCAAAGTTTCATATCCGAAGCTAGTTTTCAAGAAAC 23000

Lser_LAC005780 22951 GCATCTATGAATACTCAAAGTTTCATATCCGAAGCTAGTTTTCAAGAAAC 23000

Lvir_CGN013357 22949 GCATCTATGAATACTCAAAGTTTCATATCCGAAGCTAGTTTTCAAGAAAC 22998

Lsal_LAC008020 22948 GCATCTATGAATACTCAAAGTTTCATATCCGAAGCTAGTTTTCAAGAAAC 22997

Lsat_Salinas 23001 TGCTAGAGTTTTAGCAAAAGCAGCTCTCCTGGGCCGTATCGATTGGTTGA 23050

Lsat_WENDEL 23001 TGCTAGAGTTTTAGCAAAAGCAGCTCTCCTGGGCCGTATCGATTGGTTGA 23050

Lser_US96UC23 23001 TGCTAGAGTTTTAGCAAAAGCAGCTCTCCTGGGCCGTATCGATTGGTTGA 23050

Lser_LAC005780 23001 TGCTAGAGTTTTAGCAAAAGCAGCTCTCCTGGGCCGTATCGATTGGTTGA 23050

Lvir_CGN013357 22999 TGCTAGAGTTTTAGCAAAAGCAGCTCTCCTGGGCCGTATCGATTGGTTGA 23048

Lsal_LAC008020 22998 TGCTAGAGTTTTAGCAAAAGCAGCTCTCCTGGGCCGTATCGATTGGTTGA 23047

Lsat_Salinas 23051 AAGGCCTGAAAGAAAACGTTGTTCTGGGGGGGATGATACCTGTTGGTAGC 23100

Lsat_WENDEL 23051 AAGGCCTGAAAGAAAACGTTGTTCTGGGGGGGATGATACCTGTTGGTAGC 23100

Lser_US96UC23 23051 AAGGCCTGAAAGAAAACGTTGTTCTGGGGGGGATGATACCTGTTGGTAGC 23100

Lser_LAC005780 23051 AAGGCCTGAAAGAAAACGTTGTTCTGGGGGGGATGATACCTGTTGGTAGC 23100

Lvir_CGN013357 23049 AAGGCCTGAAAGAAAACGTTGTTCTGGGGGGGATGATACCTGTTGGTAGC 23098

Lsal_LAC008020 23048 AAGGCCTGAAAGAAAACGTTGTTCTGGGGGGGATGATACCTGTTGGTAGC 23097

Lsat_Salinas 23101 GGCTTCAAAACACCTTCAAGCGAACCTAACAACATTCCTAACAACATTGC 23150

Lsat_WENDEL 23101 GGCTTCAAAACACCTTCAAGCGAACCTAACAACATTCCTAACAACATTGC 23150

Lser_US96UC23 23101 GGCTTCAAAACACCTTCAAGCGAACCTAACAACATTCCTAACAACATTGC 23150

Lser_LAC005780 23101 GGCTTCAAAACACCTTCAAGCGAACCTAACAACATTCCTAACAACATTGC 23150

Lvir_CGN013357 23099 GGCTTCAAAACACCTTCAAGCGAACCTAACAACATTCCTAACAACATTGC 23148

Lsal_LAC008020 23098 GGCTTCAAAACACCTTCAAGCGAACCTAACAACATTCCTAACAACATTGC 23147

Lsat_Salinas 23151 CTTTGAACTCCAAAAAAAGAATCTATTAGAGGGGGAAATGAAAGATATTT 23200

Lsat_WENDEL 23151 CTTTGAACTCCAAAAAAAGAATCTATTAGAGGGGGAAATGAAAGATATTT 23200

Lser_US96UC23 23151 CTTTGAACTCCAAAAAAAGAATCTATTAGAGGGGGAAATGAAAGATATTT 23200

Lser_LAC005780 23151 CTTTGAACTCCAAAAAAAGAATCTATTAGAGGGGGAAATGAAAGATATTT 23200

Lvir_CGN013357 23149 CTTTGAACTCCAAAAAAAGAATCTATTAGAGGGGGAAATGAAAGATATTT 23198

Lsal_LAC008020 23148 CTTTGAACTCCAAAAAAAGAATCTATTAGAGGGGGAAATGAAAGATATTT 23197

Lsat_Salinas 23201 TGTTCTACCACAGAAAATTATTTGATTCTTGCCTTTCAAATAATTTCCAT 23250

Lsat_WENDEL 23201 TGTTCTACCACAGAAAATTATTTGATTCTTGCCTTTCAAATAATTTCCAT 23250

Lser_US96UC23 23201 TGTTCTACCACAGAAAATTATTTGATTCTTGCCTTTCAAATAATTTCCAT 23250

Lser_LAC005780 23201 TGTTCTACCACAGAAAATTATTTGATTCTTGCCTTTCAAATAATTTCCAT 23250

Lvir_CGN013357 23199 TGTTCTACCACAGAAAATTATTTGATTCTTGCCTTTCAAATAATTTCCAT 23248

Lsal_LAC008020 23198 TGTTCTACCACAGAAAATTATTTGATTCTTGCCTTTCAAATAATTTACAT 23247

Lsat_Salinas 23251 GATACACAAGAACAATCATTTTTTTAGGATTTAATGATCTCTAAGAGCAG 23300

Lsat_WENDEL 23251 GATACACAAGAACAATCATTTTTTTAGGATTTAATGATCTCTAAGAGCAG 23300

Lser_US96UC23 23251 GATACACAAGAACAATCATTTTTTTAGGATTTAATGATCTCTAAGAGCAG 23300

Lser_LAC005780 23251 GATACACAAGAACAATCATTTTTTTAGGATTTAATGATCTCTAAGAGCAG 23300

Lvir_CGN013357 23249 AATACACAAGAACAATCATTTTTTTAGGATTTAATGATCTCTAAGAGCAG 23298

Lsal_LAC008020 23248 GATACACAAGAACAATCATTTTTTTAGGATTTAATGATCTCTAAGAGCGG 23297

Lsat_Salinas 23301 ATTCACCCCTTTCTTTTATCTTTTATAAAGGATCTGTTGGTCCAGTCATT 23350

Lsat_WENDEL 23301 ATTCACCCCTTTCTTTTATCTTTTATAAAGGATCTGTTGGTCCAGTCATT 23350

Lser_US96UC23 23301 ATTCACCCCTTTCTTTTATCTTTTATAAAGGATCTGTTGGTCCAGTCATT 23350

Lser_LAC005780 23301 ATTCACCCCTTTCTTTTATCTTTTATAAAGGATCTGTTGGTCCAGTCATT 23350

Lvir_CGN013357 23299 ATTCACCCCTTTCTTTTATCTTTTATAAAGGATCTGTTGGTCCAGTCATT 23348

Lsal_LAC008020 23298 ATTCACCCCTTTCTTTTATCTTTTATAAAGGATCTGTTGGTCCAGTCATT 23347

Lsat_Salinas 23351 TGATTTGGTAATAGTAATCAAAAAAAATAACAAATAGAAAAGAATAATGG 23400

Lsat_WENDEL 23351 TGATTTGGTAATAGTAATCAAAAAAAATAACAAATAGAAAAGAATAATGG 23400

Lser_US96UC23 23351 TGATTTGGTAATAGTAATCAAAAAAAATAACAAATAGAAAAGAATAATGG 23400

Lser_LAC005780 23351 TGATTTGGTAATAGTAATCAAAAAAAATAACAAATAGAAAAGAATAATGG 23400

Lvir_CGN013357 23349 TGATTTGGTAATAGTAATCAAAAAAAATAACAAATAGAAAAGAATAATGG 23398

Lsal_LAC008020 23348 TGATTTGGTAATAGTAATCAAAAAAAATAACAAATAGAAAAGAATAATGG 23397

Lsat_Salinas 23401 CTTGGGCTGTGTATCTACGCCAATCTATGGTATAAAAGAAGGTTCCATCG 23450

Lsat_WENDEL 23401 CTTGGGCTGTGTATCTACGCCAATCTATGGTATAAAAGAAGGTTCCATCG 23450

Lser_US96UC23 23401 CTTGGGCTGTGTATCTACGCCAATCTATGGTATAAAAGAAGGTTCCATCG 23450

Lser_LAC005780 23401 CTTGGGCTGTGTATCTACGCCAATCTATGGTATAAAAGAAGGTTCCATCG 23450

Lvir_CGN013357 23399 CTTGGGCTGTGTATCTACGCCAATCTATGGTATAAAAGAAGGTTCCATCG 23448

Lsal_LAC008020 23398 CTTGGGCTGTGTATCTACGCCAATCTATGGTATAAAAGAAGGTTCCATCG 23447

Lsat_Salinas 23451 GAACAATTATTTATTTCAGGGTACCTCTCTCTTTTTTTTTTCAAAAGGGG 23500

Lsat_WENDEL 23451 GAACAATTATTTATTTCAGGGTACCTCTCTCTTTTTTTTTTCAAAAGGGG 23500

Lser_US96UC23 23451 GAACAATTATTTATTTCAGGGTACCTCTCTCTTTTTTTTTTCAAAAGGGG 23500

Lser_LAC005780 23451 GAACAATTATTTATTTCAGGGTACCTCTCTCTTTTTTTTTTCAAAAGGGG 23500

Lvir_CGN013357 23449 GAACAATTATTTATTTCAGGGTACCTCTCTCTTTTTTTTTTCAAAAGGGG 23498

Lsal_LAC008020 23448 GAACAATTATTTATTTCAGGGTACCTCTCTCTTTTTTTTTTCAAAAGGGG 23497

Lsat_Salinas 23501 GAGGCAGTGTGGGGAGAAATGACAAGAAGATATTGGAACATTAATTTAGA 23550

Lsat_WENDEL 23501 GAGGCAGTGTGGGGAGAAATGACAAGAAGATATTGGAACATTAATTTAGA 23550

Lser_US96UC23 23501 GAGGCAGTGTGGGGAGAAATGACAAGAAGATATTGGAACATTAATTTAGA 23550

Lser_LAC005780 23501 GAGGCAGTGTGGGGAGAAATGACAAGAAGATATTGGAACATTAATTTAGA 23550

Lvir_CGN013357 23499 GAGGCAGTGTGGGGAGAAATGACAAGAAGATATTGGAACATTAATTTAGA 23548

Lsal_LAC008020 23498 GAGGCAGTGTGGGGAGAAATGACAAGAAGATATTGGAACATTAATTTAGA 23547

Lsat_Salinas 23551 AGAGATGATGGAAGCAGGAGTTCATTTTGGCCATGGTACTAGGAAATGGA 23600

Lsat_WENDEL 23551 AGAGATGATGGAAGCAGGAGTTCATTTTGGCCATGGTACTAGGAAATGGA 23600

Lser_US96UC23 23551 AGAGATGATGGAAGCAGGAGTTCATTTTGGCCATGGTACTAGGAAATGGA 23600

Lser_LAC005780 23551 AGAGATGATGGAAGCAGGAGTTCATTTTGGCCATGGTACTAGGAAATGGA 23600

Lvir_CGN013357 23549 AGAGATGATGGAAGCAGGAGTTCATTTTGGCCATGGTACTAGGAAATGGA 23598

Lsal_LAC008020 23548 AGAGATGATGGAAGCAGGAGTTCATTTTGGCCATGGTACTAGGAAATGGA 23597

Lsat_Salinas 23601 ATCCTAAAATGGCACCTTATATCTCTGCAAAACGTAAAGGTATTCATATT 23650

Lsat_WENDEL 23601 ATCCTAAAATGGCACCTTATATCTCTGCAAAACGTAAAGGTATTCATATT 23650

Lser_US96UC23 23601 ATCCTAAAATGGCACCTTATATCTCTGCAAAACGTAAAGGTATTCATATT 23650

Lser_LAC005780 23601 ATCCTAAAATGGCACCTTATATCTCTGCAAAACGTAAAGGTATTCATATT 23650

Lvir_CGN013357 23599 ATCCTAAAATGGCACCTTATATCTCTGCAAAACGTAAAGGTATTCATATT 23648

Lsal_LAC008020 23598 ATCCTAAAATGGCACCTTATATCTCTGCAAAACGTAAAGGTATTCATATT 23647

Lsat_Salinas 23651 ACAAATCTTACTAGAACTGCTCGTTTTTTATCAGAAGCTTGTGATTTGGT 23700

Lsat_WENDEL 23651 ACAAATCTTACTAGAACTGCTCGTTTTTTATCAGAAGCTTGTGATTTGGT 23700

Lser_US96UC23 23651 ACAAATCTTACTAGAACTGCTCGTTTTTTATCAGAAGCTTGTGATTTGGT 23700

Lser_LAC005780 23651 ACAAATCTTACTAGAACTGCTCGTTTTTTATCAGAAGCTTGTGATTTGGT 23700

Lvir_CGN013357 23649 ACAAATCTTACTAGAACTGCTCGTTTTTTATCAGAAGCTTGTGATTTGGT 23698

Lsal_LAC008020 23648 ACAAATCTTACTAGAACTGCTCGTTTTTTATCAGAAGCTTGTGATTTGGT 23697

Lsat_Salinas 23701 TTTTGATGCAGCAAGCAGAGGAAAACAATTCTTAATTGTTGGCACTAAAA 23750

Lsat_WENDEL 23701 TTTTGATGCAGCAAGCAGAGGAAAACAATTCTTAATTGTTGGCACTAAAA 23750

Lser_US96UC23 23701 TTTTGATGCAGCAAGCAGAGGAAAACAATTCTTAATTGTTGGCACTAAAA 23750

Lser_LAC005780 23701 TTTTGATGCAGCAAGCAGAGGAAAACAATTCTTAATTGTTGGCACTAAAA 23750

Lvir_CGN013357 23699 TTTTGATGCAGCAAGCAGAGGAAAACAATTCTTAATTGTTGGCACTAAAA 23748

Lsal_LAC008020 23698 TTTTGATGCAGCAAGCAGAGGAAAACAATTCTTAATTGTTGGCACTAAAA 23747

Lsat_Salinas 23751 ATAAAGAAGCTGATTCAGTAGCATGGGCTGCAATAAGGGCTCGGTGTCAT 23800

Lsat_WENDEL 23751 ATAAAGAAGCTGATTCAGTAGCATGGGCTGCAATAAGGGCTCGGTGTCAT 23800

Lser_US96UC23 23751 ATAAAGAAGCTGATTCAGTAGCATGGGCTGCAATAAGGGCTCGGTGTCAT 23800

Lser_LAC005780 23751 ATAAAGAAGCTGATTCAGTAGCATGGGCTGCAATAAGGGCTCGGTGTCAT 23800

Lvir_CGN013357 23749 ATAAAGAAGCTGATTCAGTAGCATGGGCTGCAATAAGGGCTCGGTGTCAT 23798

Lsal_LAC008020 23748 ATAAAGAAGCTGATTCAGTAGCATGGGCTGCAATAAGGGCTCGGTGTCAT 23797

Lsat_Salinas 23801 TATGTTAATAAAAAATGGCTCGGTGGTATGTTAACGAATTGGTCCACTAC 23850

Lsat_WENDEL 23801 TATGTTAATAAAAAATGGCTCGGTGGTATGTTAACGAATTGGTCCACTAC 23850

Lser_US96UC23 23801 TATGTTAATAAAAAATGGCTCGGTGGTATGTTAACGAATTGGTCCACTAC 23850

Lser_LAC005780 23801 TATGTTAATAAAAAATGGCTCGGTGGTATGTTAACGAATTGGTCCACTAC 23850

Lvir_CGN013357 23799 TATGTTAATAAAAAATGGCTCGGTGGTATGTTAACGAATTGGTCCACTAC 23848

Lsal_LAC008020 23798 TATGTTAATAAAAAATGGCTCGGTGGTATGTTAACGAATTGGTCCACTAC 23847

Lsat_Salinas 23851 AGAAACAAGACTTCATAAGTTTAGAGACTTGAGAACCGAACAAAAAACAG 23900

Lsat_WENDEL 23851 AGAAACAAGACTTCATAAGTTTAGAGACTTGAGAACCGAACAAAAAACAG 23900

Lser_US96UC23 23851 AGAAACAAGACTTCATAAGTTTAGAGACTTGAGAACCGAACAAAAAACAG 23900

Lser_LAC005780 23851 AGAAACAAGACTTCATAAGTTTAGAGACTTGAGAACCGAACAAAAAACAG 23900

Lvir_CGN013357 23849 AGAAACAAGACTTCATAAGTTTAGAGACTTGAGAACCGAACAAAAAACAG 23898

Lsal_LAC008020 23848 AGAAACAAGACTTCATAAGTTTAGAGACTTGAGAACCGAACAAAAAACAG 23897

Lsat_Salinas 23901 GGGGGCTCGACCGTCTTCCGAAAAGAGATGCGGCTATGTTGAAAAGACAA 23950

Lsat_WENDEL 23901 GGGGGCTCGACCGTCTTCCGAAAAGAGATGCGGCTATGTTGAAAAGACAA 23950

Lser_US96UC23 23901 GGGGGCTCGACCGTCTTCCGAAAAGAGATGCGGCTATGTTGAAAAGACAA 23950

Lser_LAC005780 23901 GGGGGCTCGACCGTCTTCCGAAAAGAGATGCGGCTATGTTGAAAAGACAA 23950

Lvir_CGN013357 23899 GGGGGCTCGACCGTCTTCCGAAAAGAGATGCGGCTATGTTGAAAAGACAA 23948

Lsal_LAC008020 23898 GGGGGCTCGACCGTCTTCCGAAAAGAGATGCGGCTATGTTGAAAAGACAA 23947

Lsat_Salinas 23951 TTATCTCATTTGCAAACATATCTGGGTGGGATTAAATATATGACAGGGTT 24000

Lsat_WENDEL 23951 TTATCTCATTTGCAAACATATCTGGGTGGGATTAAATATATGACAGGGTT 24000

Lser_US96UC23 23951 TTATCTCATTTGCAAACATATCTGGGTGGGATTAAATATATGACAGGGTT 24000

Lser_LAC005780 23951 TTATCTCATTTGCAAACATATCTGGGTGGGATTAAATATATGACAGGGTT 24000

Lvir_CGN013357 23949 TTATCTCATTTGCAAACATATCTGGGCGGGATTAAATATATGACAGGGTT 23998

Lsal_LAC008020 23948 TTATCTCATTTGCAAACATATCTGGGCGGGATTAAATATATGACAGGGTT 23997

Lsat_Salinas 24001 ACCCGATATTGTAATCATCGTTGATCAGCACGAAGAATATACGGCCCTTC 24050

Lsat_WENDEL 24001 ACCCGATATTGTAATCATCGTTGATCAGCACGAAGAATATACGGCCCTTC 24050

Lser_US96UC23 24001 ACCCGATATTGTAATCATCGTTGATCAGCACGAAGAATATACGGCCCTTC 24050

Lser_LAC005780 24001 ACCCGATATTGTAATCATCGTTGATCAGCACGAAGAATATACGGCCCTTC 24050

Lvir_CGN013357 23999 ACCCGATATTGTAATCATCGTTGATCAGCACGAAGAATATACGGCCCTTC 24048

Lsal_LAC008020 23998 ACCCGATATTGTAATCATCGTTGATCAGCACGAAGAATATACGGCCCTTC 24047

Lsat_Salinas 24051 AAGAATGTATCACGTTGGGAATTCCAACAATTTGTTTAATCGATACAAAT 24100

Lsat_WENDEL 24051 AAGAATGTATCACGTTGGGAATTCCAACAATTTGTTTAATCGATACAAAT 24100

Lser_US96UC23 24051 AAGAATGTATCACGTTGGGAATTCCAACAATTTGTTTAATCGATACAAAT 24100

Lser_LAC005780 24051 AAGAATGTATCACGTTGGGAATTCCAACAATTTGTTTAATCGATACAAAT 24100

Lvir_CGN013357 24049 AAGAATGTATCACGTTGGGAATTCCAACAATTTGTTTAATCGATACAAAT 24098

Lsal_LAC008020 24048 AAGAATGTATCACGTTGGGAATTCCAACAATTTGTTTAATCGATACAAAT 24097

Lsat_Salinas 24101 TGTGACCCCGATCTCGCAGATATTTCGATTCCAGCCAATGATGACGCTAT 24150

Lsat_WENDEL 24101 TGTGACCCCGATCTCGCAGATATTTCGATTCCAGCCAATGATGACGCTAT 24150

Lser_US96UC23 24101 TGTGACCCCGATCTCGCAGATATTTCGATTCCAGCCAATGATGACGCTAT 24150

Lser_LAC005780 24101 TGTGACCCCGATCTCGCAGATATTTCGATTCCAGCCAATGATGACGCTAT 24150

Lvir_CGN013357 24099 TGTGACCCCGATCTCGCAGATATTTCGATTCCAGCCAATGATGACGCTAT 24148

Lsal_LAC008020 24098 TGTGACCCCGATCTCGCAGATATTTCGATTCCAGCCAATGATGACGCTAT 24147

Lsat_Salinas 24151 ATCTTCAATCCGATTAATTCTTAACAAATTAGTATTTGCAATTTGTGAAG 24200

Lsat_WENDEL 24151 ATCTTCAATCCGATTAATTCTTAACAAATTAGTATTTGCAATTTGTGAAG 24200

Lser_US96UC23 24151 ATCTTCAATCCGATTAATTCTTAACAAATTAGTATTTGCAATTTGTGAAG 24200

Lser_LAC005780 24151 ATCTTCAATCCGATTAATTCTTAACAAATTAGTATTTGCAATTTGTGAAG 24200

Lvir_CGN013357 24149 ATCTTCAATCCGATTAATTCTTAACAAATTAGTATTTGCAATTTGTGAAG 24198

Lsal_LAC008020 24148 ATCTTCAATCCGATTAATTCTTAACAAATTAGTATTTGCAATTTGTGAAG 24197

Lsat_Salinas 24201 GCCGTTCTGGCTATATAAGAAATCCGTGATTAATAATAAGATAAATAACT 24250

Lsat_WENDEL 24201 GCCGTTCTGGCTATATAAGAAATCCGTGATTAATAATAAGATAAATAACT 24250

Lser_US96UC23 24201 GCCGTTCTGGCTATATAAGAAATCCGTGATTAATAATAAGATAAATAACT 24250

Lser_LAC005780 24201 GCCGTTCTGGCTATATAAGAAATCCGTGATTAATAATAAGATAAATAACT 24250

Lvir_CGN013357 24199 GCCGTTCTGGCTATATAAGAAATCCGTGATTAATAATAAGATAAATAACT 24248

Lsal_LAC008020 24198 GCCGTTCTGGCTATATAAGAAATCCGTGATTAATAATAAGATAAATAACT 24247

Lsat_Salinas 24251 TATTTCATTTCGGAACCTTCATAGATTTATCGAATCGGTTACTATTTCTG 24300

Lsat_WENDEL 24251 TATTTCATTTCGGAACCTTCATAGATTTATCGAATCGGTTACTATTTCTG 24300

Lser_US96UC23 24251 TATTTCATTTCGGAACCTTCATAGATTTATCGAATCGGTTACTATTTCTG 24300

Lser_LAC005780 24251 TATTTCATTTCGGAACCTTCATAGATTTATCGAATCGGTTACTATTTCTG 24300

Lvir_CGN013357 24249 TATTTCATTTCGGAACCTTCATAGATTTATCGAATCGGTTACTATTTCTG 24298

Lsal_LAC008020 24248 TATTTCATTTCGGAACCTTCATAGATTTATCGAATCGGTTACTATTTCTG 24297

Lsat_Salinas 24301 AATCTCAAAAATAGAGATAAAAATAACAAGGAATAGAATTTGATTAGTTG 24350

Lsat_WENDEL 24301 AATCTCAAAAATAGAGATAAAAATAACAAGGAATAGAATTTGATTAGTTG 24350

Lser_US96UC23 24301 AATCTCAAAAATAGAGATAAAAATAACAAGGAATAGAATTTGATTAGTTG 24350

Lser_LAC005780 24301 AATCTCAAAAATAGAGATAAAAATAACAAGGAATAGAATTTGATTAGTTG 24350

Lvir_CGN013357 24299 AATCTCAAAAATAGAGATAAAAATAACAAGGAATAGAATTTGATTAGTTG 24348

Lsal_LAC008020 24298 AATCTCAAAAATAGAGATAAAAATAACAAGGAATAGAATTTGATTAGTTG 24347

Lsat_Salinas 24351 GTATTCAAAATATATGATTCAAGTAGTCAAGTCGAGAAAGAGATGGTTGA 24400

Lsat_WENDEL 24351 GTATTCAAAATATATGATTCAAGTAGTCAAGTCGAGAAAGAGATGGTTGA 24400

Lser_US96UC23 24351 GTATTCAAAATATATGATTCAAGTAGTCAAGTCGAGAAAGAGATGGTTGA 24400

Lser_LAC005780 24351 GTATTCAAAATATATGATTCAAGTAGTCAAGTCGAGAAAGAGATGGTTGA 24400

Lvir_CGN013357 24349 GTATTCAAAATATATGATTCAAGTAGTCAAGTCGAGAAAGAGATGGTTGA 24398

Lsal_LAC008020 24348 GTATTCAAAATATATGATTCAAGTAGTCAAGTCGAGAAAGAGATGGTTGA 24397

Lsat_Salinas 24401 ATCAAAATAATTTTGTTTAAAATTCTATTTTTGTCAGAGGGCAATATGAA 24450

Lsat_WENDEL 24401 ATCAAAATAATTTTGTTTAAAATTCTATTTTTGTCAGAGGGCAATATGAA 24450

Lser_US96UC23 24401 ATCAAAATAATTTTGTTTAAAATTCTATTTTTGTCAGAGGGCAATATGAA 24450

Lser_LAC005780 24401 ATCAAAATAATTTTGTTTAAAATTCTATTTTTGTCAGAGGGCAATATGAA 24450

Lvir_CGN013357 24399 ATCAAAATAATTTTGTTTAAAATTCTATTTTTGTCAGAGGGCAATATGAA 24448

Lsal_LAC008020 24398 ATCAAAATAATTTTGTTTAAAATTCTATTTTTGTCAGAGGGCAATATGAA 24447

Lsat_Salinas 24451 TGTTCTATCATGTTCCATCAACACACTAAATGGGTTATACGATATATCCG 24500

Lsat_WENDEL 24451 TGTTCTATCATGTTCCATCAACACACTAAATGGGTTATACGATATATCCG 24500

Lser_US96UC23 24451 TGTTCTATCATGTTCCATCAACACACTAAATGGGTTATACGATATATCCG 24500

Lser_LAC005780 24451 TGTTCTATCATGTTCCATCAACACACTAAATGGGTTATACGATATATCCG 24500

Lvir_CGN013357 24449 TGTTCTATCATGTTCCATCAACACACTAAATGGGTTATACGATATATCCG 24498

Lsal_LAC008020 24448 TGTTCTATCATGTTCCATCAACACACTAAATGGGTTATACGATATATCCG 24497

Lsat_Salinas 24501 GTGTGGAAGTAGGCCAACATTTTTATTGGAAAATCGGGGGTTTCCAAGTC 24550

Lsat_WENDEL 24501 GTGTGGAAGTAGGCCAACATTTTTATTGGAAAATCGGGGGTTTCCAAGTC 24550

Lser_US96UC23 24501 GTGTGGAAGTAGGCCAACATTTTTATTGGAAAATCGGGGGTTTCCAAGTC 24550

Lser_LAC005780 24501 GTGTGGAAGTAGGCCAACATTTTTATTGGAAAATCGGGGGTTTCCAAGTC 24550

Lvir_CGN013357 24499 GTGTGGAAGTAGGCCAACATTTTTATTGGAAAATCGGGGGTTTCCAAGTC 24548

Lsal_LAC008020 24498 GTGTGGAAGTAGGCCAACATTTTTATTGGAAAATCGGGGGTTTCCAAGTC 24547

Lsat_Salinas 24551 CACGGCCAAGTACTTATTACTTCTTGGGTTGTAATTGCTATCTTATTAGC 24600

Lsat_WENDEL 24551 CACGGCCAAGTACTTATTACTTCTTGGGTTGTAATTGCTATCTTATTAGC 24600

Lser_US96UC23 24551 CACGGCCAAGTACTTATTACTTCTTGGGTTGTAATTGCTATCTTATTAGC 24600

Lser_LAC005780 24551 CACGGCCAAGTACTTATTACTTCTTGGGTTGTAATTGCTATCTTATTAGC 24600

Lvir_CGN013357 24549 CACGGCCAAGTACTTATTACTTCTTGGGTTGTAATTGCTATCTTATTAGC 24598

Lsal_LAC008020 24548 CACGGCCAAGTACTTATTACTTCTTGGGTTGTAATTGCTATCTTATTAGC 24597

Lsat_Salinas 24601 TTCAGCCACTCTAGCCGTTCGGAACCCACAAACCATTCCGACCAGCGGTC 24650

Lsat_WENDEL 24601 TTCAGCCACTCTAGCCGTTCGGAACCCACAAACCATTCCGACCAGCGGTC 24650

Lser_US96UC23 24601 TTCAGCCACTCTAGCCGTTCGGAACCCACAAACCATTCCGACCAGCGGTC 24650

Lser_LAC005780 24601 TTCAGCCACTCTAGCCGTTCGGAACCCACAAACCATTCCGACCAGCGGTC 24650

Lvir_CGN013357 24599 TTCAGCCACTCTAGCCGTTCGGAACCCACAAACCATTCCGACCAGCGGTC 24648

Lsal_LAC008020 24598 TTCAGCCACTCTAGCCGTTCGGAACCCACAAACCATTCCGACCAGCGGTC 24647

Lsat_Salinas 24651 AGAATTTCTTCGAATATGTCCTTGAATTTATTCGAGATGTGAGTAAAACT 24700

Lsat_WENDEL 24651 AGAATTTCTTCGAATATGTCCTTGAATTTATTCGAGATGTGAGTAAAACT 24700

Lser_US96UC23 24651 AGAATTTCTTCGAATATGTCCTTGAATTTATTCGAGATGTGAGTAAAACT 24700

Lser_LAC005780 24651 AGAATTTCTTCGAATATGTCCTTGAATTTATTCGAGATGTGAGTAAAACT 24700

Lvir_CGN013357 24649 AGAATTTCTTCGAATATGTCCTTGAATTTATTCGAGATGTGAGTAAAACT 24698

Lsal_LAC008020 24648 AGAATTTCTTTGAATATGTCCTTGAATTTATTCGAGATGTGAGTAAAACT 24697

Lsat_Salinas 24701 CAAATTGGAGAAGAATATGGTCCTTGGGTTCCTTTTATTGGAACTATGTT 24750

Lsat_WENDEL 24701 CAAATTGGAGAAGAATATGGTCCTTGGGTTCCTTTTATTGGAACTATGTT 24750

Lser_US96UC23 24701 CAAATTGGAGAAGAATATGGTCCTTGGGTTCCTTTTATTGGAACTATGTT 24750

Lser_LAC005780 24701 CAAATTGGAGAAGAATATGGTCCTTGGGTTCCTTTTATTGGAACTATGTT 24750

Lvir_CGN013357 24699 CAAATTGGAGAAGAATATGGTCCTTGGGTTCCTTTTATTGGAACTATGTT 24748

Lsal_LAC008020 24698 CAAATTGGAGAAGAATATGGTCCTTGGGTTCCTTTTATTGGAACTATGTT 24747

Lsat_Salinas 24751 TCTATTTATTTTTGTTTCTAATTGGTCAGGCGCCCTTTTACCTTGGAAAA 24800

Lsat_WENDEL 24751 TCTATTTATTTTTGTTTCTAATTGGTCAGGCGCCCTTTTACCTTGGAAAA 24800

Lser_US96UC23 24751 TCTATTTATTTTTGTTTCTAATTGGTCAGGCGCCCTTTTACCTTGGAAAA 24800

Lser_LAC005780 24751 TCTATTTATTTTTGTTTCTAATTGGTCAGGCGCCCTTTTACCTTGGAAAA 24800

Lvir_CGN013357 24749 TCTATTTATTTTTGTTTCTAATTGGTCAGGCGCCCTTTTACCTTGGAAAA 24798

Lsal_LAC008020 24748 TCTATTTATTTTTGTTTCTAATTGGTCAGGCGCCCTTTTACCTTGGAAAA 24797

Lsat_Salinas 24801 TCATACAATTACCTCATGGGGAGTTAGCCGCCCCCACGAATGATATAAAT 24850

Lsat_WENDEL 24801 TCATACAATTACCTCATGGGGAGTTAGCCGCCCCCACGAATGATATAAAT 24850

Lser_US96UC23 24801 TCATACAATTACCTCATGGGGAGTTAGCCGCCCCCACGAATGATATAAAT 24850

Lser_LAC005780 24801 TCATACAATTACCTCATGGGGAGTTAGCCGCCCCCACGAATGATATAAAT 24850

Lvir_CGN013357 24799 TCATACAATTACCTCATGGGGAGTTAGCCGCCCCCACGAATGATATAAAT 24848

Lsal_LAC008020 24798 TCATACAATTACCTCATGGGGAGTTAGCCGCCCCCACGAATGATATAAAT 24847

Lsat_Salinas 24851 ACTACTGTTGCTTTGGCTTTACTCACATCAGTGGCATATTTCTATGCGGG 24900

Lsat_WENDEL 24851 ACTACTGTTGCTTTGGCTTTACTCACATCAGTGGCATATTTCTATGCGGG 24900

Lser_US96UC23 24851 ACTACTGTTGCTTTGGCTTTACTCACATCAGTGGCATATTTCTATGCGGG 24900

Lser_LAC005780 24851 ACTACTGTTGCTTTGGCTTTACTCACATCAGTGGCATATTTCTATGCGGG 24900

Lvir_CGN013357 24849 ACTACTGTTGCTTTGGCTTTACTCACATCAGTGGCATATTTCTATGCGGG 24898

Lsal_LAC008020 24848 ACTACTGTTGCTTTGGCTTTACTCACATCAGTGGCATATTTCTATGCGGG 24897

Lsat_Salinas 24901 TCTTAGCAAAAAAGGATTAGGTTATTTCGGTAAATATATTCAACCAACTC 24950

Lsat_WENDEL 24901 TCTTAGCAAAAAAGGATTAGGTTATTTCGGTAAATATATTCAACCAACTC 24950

Lser_US96UC23 24901 TCTTAGCAAAAAAGGATTAGGTTATTTCGGTAAATATATTCAACCAACTC 24950

Lser_LAC005780 24901 TCTTAGCAAAAAAGGATTAGGTTATTTCGGTAAATATATTCAACCAACTC 24950

Lvir_CGN013357 24899 TCTTAGCAAAAAAGGATTAGGTTATTTCGGTAAATATATTCAACCAACTC 24948

Lsal_LAC008020 24898 TCTTAGCAAAAAAGGATTAGGTTATTTCGGTAAATATATTCAACCAACTC 24947

Lsat_Salinas 24951 CAATTCTTTTACCTATTAACATCTTAGAAGATTTCACAAAGCCCCTATCA 25000

Lsat_WENDEL 24951 CAATTCTTTTACCTATTAACATCTTAGAAGATTTCACAAAGCCCCTATCA 25000

Lser_US96UC23 24951 CAATTCTTTTACCTATTAACATCTTAGAAGATTTCACAAAGCCCCTATCA 25000

Lser_LAC005780 24951 CAATTCTTTTACCTATTAACATCTTAGAAGATTTCACAAAGCCCCTATCA 25000

Lvir_CGN013357 24949 CAATTCTTTTACCTATTAACATCTTAGAAGATTTCACAAAGCCCCTATCA 24998

Lsal_LAC008020 24948 CAATTCTTTTACCTATTAACATCTTAGAAGATTTCACAAAGCCCCTATCA 24997

Lsat_Salinas 25001 CTTAGTTTTCGACTTTTCGGAAATATATTAGCCGATGAATTAGTAGTTGT 25050

Lsat_WENDEL 25001 CTTAGTTTTCGACTTTTCGGAAATATATTAGCCGATGAATTAGTAGTTGT 25050

Lser_US96UC23 25001 CTTAGTTTTCGACTTTTCGGAAATATATTAGCCGATGAATTAGTAGTTGT 25050

Lser_LAC005780 25001 CTTAGTTTTCGACTTTTCGGAAATATATTAGCCGATGAATTAGTAGTTGT 25050

Lvir_CGN013357 24999 CTTAGTTTTCGACTTTTCGGAAATATATTAGCCGATGAATTAGTAGTTGT 25048

Lsal_LAC008020 24998 CTTAGTTTTCGACTTTTCGGAAATATATTAGCCGATGAATTAGTAGTTGT 25047

Lsat_Salinas 25051 TGTTCTTGTTTCTTTAGTACCTTCAGTGGTTCCTATCCCTGTCATGTTCC 25100

Lsat_WENDEL 25051 TGTTCTTGTTTCTTTAGTACCTTCAGTGGTTCCTATCCCTGTCATGTTCC 25100

Lser_US96UC23 25051 TGTTCTTGTTTCTTTAGTACCTTCAGTGGTTCCTATCCCTGTCATGTTCC 25100

Lser_LAC005780 25051 TGTTCTTGTTTCTTTAGTACCTTCAGTGGTTCCTATCCCTGTCATGTTCC 25100

Lvir_CGN013357 25049 TGTTCTTGTTTCTTTAGTACCTTCAGTGGTTCCTATCCCTGTCATGTTCC 25098

Lsal_LAC008020 25048 TGTTCTTGTTTCTTTAGTACCTTCAGTGGTTCCTATCCCTGTCATGTTCC 25097

Lsat_Salinas 25101 TTGGATTATTTACAAGTGGTATTCAAGCTCTTATTTTTGCAACTTTAGCT 25150

Lsat_WENDEL 25101 TTGGATTATTTACAAGTGGTATTCAAGCTCTTATTTTTGCAACTTTAGCT 25150

Lser_US96UC23 25101 TTGGATTATTTACAAGTGGTATTCAAGCTCTTATTTTTGCAACTTTAGCT 25150

Lser_LAC005780 25101 TTGGATTATTTACAAGTGGTATTCAAGCTCTTATTTTTGCAACTTTAGCT 25150

Lvir_CGN013357 25099 TTGGATTATTTACAAGTGGTATTCAAGCTCTTATTTTTGCAACTTTAGCT 25148

Lsal_LAC008020 25098 TTGGATTATTTACAAGTGGTATTCAAGCTCTTATTTTTGCAACTTTAGCT 25147

Lsat_Salinas 25151 GCGGCTTATATAGGTGAATCCATGGAGGGCCACCATTGACTAGTTTTCTA 25200

Lsat_WENDEL 25151 GCGGCTTATATAGGTGAATCCATGGAGGGCCACCATTGACTAGTTTTCTA 25200

Lser_US96UC23 25151 GCGGCTTATATAGGTGAATCCATGGAGGGCCACCATTGACTAGTTTTCTA 25200

Lser_LAC005780 25151 GCGGCTTATATAGGTGAATCCATGGAGGGCCACCATTGACTAGTTTTCTA 25200

Lvir_CGN013357 25149 GCGGCTTATATAGGTGAATCCATGGAGGGCCACCATTGACTAGTTTTCTA 25198

Lsal_LAC008020 25148 GCGGCTTATATAGGTGAATCCATGGAGGGCCACCATTGACTAGTTTTCTA 25197

Lsat_Salinas 25201 AATAGTCTTTTTTTAGTTTAACCTAAGGCAATGTTGTGTGGCTAAAAAAA 25250

Lsat_WENDEL 25201 AATAGTCTTTTTTTAGTTTAACCTAAGGCAATGTTGTGTGGCTAAAAAAA 25250

Lser_US96UC23 25201 AATAGTCTTTTTTTAGTTTAACCTAAGGCAATGTTGTGTGGCTAAAAAAA 25250

Lser_LAC005780 25201 AATAGTCTTTTTTTAGTTTAACCTAAGGCAATGTTGTGTGGCTAAAAAAA 25250

Lvir_CGN013357 25199 AATAGTCTTTTTTTAGTTTAACCTAAGGCAATGTGGTGTGGCTAAAAAAA 25248

Lsal_LAC008020 25198 AATAGTCTTTTTTTAGTTTAACCTAAGGCAATGTTGTGTGGCTAAAAAAA 25247

Lsat_Salinas 25251 ATTGACTTAGGGAATGAAAATACCCAACTACACTATATATGATCTAGAGT 25300

Lsat_WENDEL 25251 ATTGACTTAGGGAATGAAAATACCCAACTACACTATATATGATCTAGAGT 25300

Lser_US96UC23 25251 ATTGACTTAGGGAATGAAAATACCCAACTACACTATATATGATCTAGAGT 25300

Lser_LAC005780 25251 ATTGACTTAGGGAATGAAAATACCCAACTACACTATATATGATCTAGAGT 25300

Lvir_CGN013357 25249 ATTGACTTAGGGAATGAAAATACCCAACTACACTATATATGATCTAGAGT 25298

Lsal_LAC008020 25248 ATTGACTTAGGGAATGAAAATACCCAACTACACTATATATGATCTAGAGT 25297

Lsat_Salinas 25301 AATAGAAAAAAAGATTACAATAAAGATTACAATATTGATATTAGAGTAGA 25350

Lsat_WENDEL 25301 AATAGAAAAAAAGATTACAATAAAGATTACAATATTGATATTAGAGTAGA 25350

Lser_US96UC23 25301 AATAGAAAAAAAGATTACAATAAAGATTACAATATTGATATTAGAGTAGA 25350

Lser_LAC005780 25301 AATAGAAAAAAAGATTACAATAAAGATTACAATATTGATATTAGAGTAGA 25350

Lvir_CGN013357 25299 AATAGAAAAAAAGATTACAATAAAGATTACAATATTGATATTAGAGTAGA 25348

Lsal_LAC008020 25298 AATAGAAAAAAAGATTACAATAAAGATTACAATATTGATATTAGAGTAGA 25347

Lsat_Salinas 25351 GAATAAAAAAAAAAGGAAAGAGATCTCAAAGATCTTTTTCCTAAAATTCC 25400

Lsat_WENDEL 25351 GAATAAAAAAAAAAGGAAAGAGATCTCAAAGATCTTTTTCCTAAAATTCC 25400

Lser_US96UC23 25351 GAATAAAAAAAAAAGGAAAGAGATCTCAAAGATCTTTTTCCTAAAATTCC 25400

Lser_LAC005780 25351 GAATAAAAAAAAAAGGAAAGAGATCTCAAAGATCTTTTTCCTAAAATTCC 25400

Lvir_CGN013357 25349 GAATAAAAAAAAAAGGAAAGAGATCTCAAAGATCTTTTTCCTAAAATTCC 25398

Lsal_LAC008020 25348 GAATAAAAAAAAAAGGAAAGAGATCTCAAAGATCTTTTTCCTAAAATTCC 25397

Lsat_Salinas 25401 CGTTTGGTGCATTTATATCATAATCATACTTTCCCCTCAATGAATATTGA 25450

Lsat_WENDEL 25401 CGTTTGGTGCATTTATATCATAATCATACTTTCCCCTCAATGAATATTGA 25450

Lser_US96UC23 25401 CGTTTGGTGCATTTATATCATAATCATACTTTCCCCTCAATGAATATTGA 25450

Lser_LAC005780 25401 CGTTTGGTGCATTTATATCATAATCATACTTTCCCCTCAATGAATATTGA 25450

Lvir_CGN013357 25399 CGTTTGGTGCATTTATATCATAATCATACTTTCCCCTCAATGAATATTGA 25448

Lsal_LAC008020 25398 CGTTTGGTGCATTTATATCATAATCATACTTTCCCCTCAATGAATATTGA 25447

Lsat_Salinas 25451 GTTTAGATTGGTCATCGAATCCGAATATGAACTATTCGGAGTCGTAATTT 25500

Lsat_WENDEL 25451 GTTTAGATTGGTCATCGAATCCGAATATGAACTATTCGGAGTCGTAATTT 25500

Lser_US96UC23 25451 GTTTAGATTGGTCATCGAATCCGAATATGAACTATTCGGAGTCGTAATTT 25500

Lser_LAC005780 25451 GTTTAGATTGGTCATCGAATCCGAATATGAACTATTCGGAGTCGTAATTT 25500

Lvir_CGN013357 25449 GTTTAGATTGGTCATCGAATCCGAATATGAACTATTCGGAGTCGTAATTT 25498

Lsal_LAC008020 25448 GTTTAGATTGGTCATCGAATCCGAATATGAACTATTCGGAGTCGTAATTT 25497

Lsat_Salinas 25501 GACGAAGAAGTCTTGTACGATGTGTGATTAAATAAAAAAAGGATGTTCTA 25550

Lsat_WENDEL 25501 GACGAAGAAGTCTTGTACGATGTGTGATTAAATAAAAAAAGGATGTTCTA 25550

Lser_US96UC23 25501 GACGAAGAAGTCTTGTACGATGTGTGATTAAATAAAAAAAGGATGTTCTA 25550

Lser_LAC005780 25501 GACGAAGAAGTCTTGTACGATGTGTGATTAAATAAAAAAAGGATGTTCTA 25550

Lvir_CGN013357 25499 GACGAAGAAGTCTTGTACGATGTGTGATTAAATAAAAAAAGGATGTTCTA 25548

Lsal_LAC008020 25498 GACGAAGAAGTCTTGTACGATGTGTGATTAAATAAAAAAAGGATGTTCTA 25547

Lsat_Salinas 25551 GAGAACGATTCCCCCTTTTCAGTTGATTTTATTCAACGATTGACCAAACG 25600

Lsat_WENDEL 25551 GAGAACGATTCCCCCTTTTCAGTTGATTTTATTCAACGATTGACCAAACG 25600

Lser_US96UC23 25551 GAGAACGATTCCCCCTTTTCAGTTGATTTTATTCAACGATTGACCAAACG 25600

Lser_LAC005780 25551 GAGAACGATTCCCCCTTTTCAGTTGATTTTATTCAACGATTGACCAAACG 25600

Lvir_CGN013357 25549 GAGAACGATTCCCCCTTTTCAGTTGATTTTATTCAACGATTGACCAAACG 25598

Lsal_LAC008020 25548 GAGAACGATTCCCCCTTTTCAGTTGATTTTATTCAACGATTGACCAAACG 25597

Lsat_Salinas 25601 AAAATATTAATAAATAATAAATTGTACGAACTTAGATCAATCAAATCAAT 25650

Lsat_WENDEL 25601 AAAATATTAATAAATAATAAATTGTACGAACTTAGATCAATCAAATCAAT 25650

Lser_US96UC23 25601 AAAATATTAATAAATAATAAATTGTACGAACTTAGATCAATCAAATCAAT 25650

Lser_LAC005780 25601 AAAATATTAATAAATAATAAATTGTACGAACTTAGATCAATCAAATCAAT 25650

Lvir_CGN013357 25599 AAAATATTAATAAATAATAAATTGTACGAACTTAGATCAATCAAATCAAT 25648

Lsal_LAC008020 25598 AAAATATTAATAAATAATAAATTGTACGAACTTATATCAATCAAATCAAT 25647

Lsat_Salinas 25651 TTCTATGCAACGATTCGGCCCAATCAATTTATCCATTTATTATCTTATCA 25700

Lsat_WENDEL 25651 TTCTATGCAACGATTCGGCCCAATCAATTTATCCATTTATTATCTTATCA 25700

Lser_US96UC23 25651 TTCTATGCAACGATTCGGCCCAATCAATTTATCCATTTATTATCTTATCA 25700

Lser_LAC005780 25651 TTCTATGCAACGATTCGGCCCAATCAATTTATCCATTTATTATCTTATCA 25700

Lvir_CGN013357 25649 TTCTATGCAACGATTCGGCCCAATCAATTTATCCATTTATTATCTTATCA 25698

Lsal_LAC008020 25648 TTCTATGCAACGATTCGGCCCAATCAATTTATCCATTTATTATCTTATCA 25697

Lsat_Salinas 25701 ATTTAGGTTGCGTGATAATGATAAAGAAAGGGAAAGGATAATTTCTAAAA 25750

Lsat_WENDEL 25701 ATTTAGGTTGCGTGATAATGATAAAGAAAGGGAAAGGATAATTTCTAAAA 25750

Lser_US96UC23 25701 ATTTAGGTTGCGTGATAATGATAAAGAAAGGGAAAGGATAATTTCTAAAA 25750

Lser_LAC005780 25701 ATTTAGGTTGCGTGATAATGATAAAGAAAGGGAAAGGATAATTTCTAAAA 25750

Lvir_CGN013357 25699 ATTTAGGTTGCGTGATAATGATAAAGAAAGGGAAAGGATAATTTCTAAAA 25748

Lsal_LAC008020 25698 ATTTAGGTTGCGTGATAATGATAAAGAAAGGGAAAGGATAATTTCTAAAA 25747

Lsat_Salinas 25751 AAGGGGATTTATAAATGGTTCGTAGAGGCGAGGTCGAACTAGGTATATGG 25800

Lsat_WENDEL 25751 AAGGGGATTTATAAATGGTTCGTAGAGGCGAGGTCGAACTAGGTATATGG 25800

Lser_US96UC23 25751 AAGGGGATTTATAAATGGTTCGTAGAGGCGAGGTCGAACTAGGTATATGG 25800

Lser_LAC005780 25751 AAGGGGATTTATAAATGGTTCGTAGAGGCGAGGTCGAACTAGGTATATGG 25800

Lvir_CGN013357 25749 AAGGGGATTTATAAATGGTTCGTAGAGGCGAGGTCGAACTAGGTATATGG 25798

Lsal_LAC008020 25748 AAGGGGATTTATAAATGGTTCGTAGAGGCGAGGTCGAACTAGGTATATGG 25797

Lsat_Salinas 25801 AATTGAATGGCTATAAGTTAACTCTTGTCAAGGGTTAGACGCATCCTTAT 25850

Lsat_WENDEL 25801 AATTGAATGGCTATAAGTTAACTCTTGTCAAGGGTTAGACGCATCCTTAT 25850

Lser_US96UC23 25801 AATTGAATGGCTATAAGTTAACTCTTGTCAAGGGTTAGACGCATCCTTAT 25850

Lser_LAC005780 25801 AATTGAATGGCTATAAGTTAACTCTTGTCAAGGGTTAGACGCATCCTTAT 25850

Lvir_CGN013357 25799 AATTGAATGGCTATAAGTTAACTCTTGTCAAGGGTTAGACGCATCCTTAT 25848

Lsal_LAC008020 25798 AATTGAATGGCTATAAGTTAACTCTTGTCAAGGGTTAGACGCATCCTTAT 25847

Lsat_Salinas 25851 CAAATCAGCTTGAATTAAAGATTAGGGAAGAGTTGGTTTACATTGTGGAA 25900

Lsat_WENDEL 25851 CAAATCAGCTTGAATTAAAGATTAGGGAAGAGTTGGTTTACATTGTGGAA 25900

Lser_US96UC23 25851 CAAATCAGCTTGAATTAAAGATTAGGGAAGAGTTGGTTTACATTGTGGAA 25900

Lser_LAC005780 25851 CAAATCAGCTTGAATTAAAGATTAGGGAAGAGTTGGTTTACATTGTGGAA 25900

Lvir_CGN013357 25849 CAAATCAGCTTGAATTAAAGATTAGGGAAGAGTTGGTTTACATTGTGGAA 25898

Lsal_LAC008020 25848 CAAATCAGCTTGAATTAAAGATTAGGGAAGAGTTGGTTTACATTGTGGAA 25897

Lsat_Salinas 25901 GAAAGACATGTATATGTGATATTAGATATTGACTAGTTATATATGAGCTA 25950

Lsat_WENDEL 25901 GAAAGACATGTATATGTGATATTAGATATTGACTAGTTATATATGAGCTA 25950

Lser_US96UC23 25901 GAAAGACATGTATATGTGATATTAGATATTGACTAGTTATATATGAGCTA 25950

Lser_LAC005780 25901 GAAAGACATGTATATGTGATATTAGATATTGACTAGTTATATATGAGCTA 25950

Lvir_CGN013357 25899 GAAAGACATGTATATGTGATATTAGATATTGACTAGTTATATATGAGCTA 25948

Lsal_LAC008020 25898 GAAAGACATGTATATGTGATATTAGATATTGACTAGTTATATATGAGCTA 25947

Lsat_Salinas 25951 AAGATATATCTAATTTCCCCTACTAATCGAATGTGAATGTAGATGGGGAT 26000

Lsat_WENDEL 25951 AAGATATATCTAATTTCCCCTACTAATCGAATGTGAATGTAGATGGGGAT 26000

Lser_US96UC23 25951 AAGATATATCTAATTTCCCCTACTAATCGAATGTGAATGTAGATGGGGAT 26000

Lser_LAC005780 25951 AAGATATATCTAATTTCCCCTACTAATCGAATGTGAATGTAGATGGGGAT 26000

Lvir_CGN013357 25949 AAGATATATCTAATTTGCCCTACTAATTGAATGTGAATGTAGATGGGGAT 25998

Lsal_LAC008020 25948 AAGATATATGTAATTTGCCCTACTAATCGAATGTGAATGTAGATGGGGAT 25997

Lsat_Salinas 26001 TCTATATATATAAGTCCTTCTGTCTCATCTGTGACTGTGAGTTCAATGAA 26050

Lsat_WENDEL 26001 TCTATATATATAAGTCCTTCTGTCTCATCTGTGACTGTGAGTTCAATGAA 26050

Lser_US96UC23 26001 TCTATATATATAAGTCCTTCTGTCTCATCTGTGACTGTGAGTTCAATGAA 26050

Lser_LAC005780 26001 TCTATATATATAAGTCCTTCTGTCTCATCTGTGACTGTGAGTTCAATGAA 26050

Lvir_CGN013357 25999 TCTATATATATAAGTCCTTCTGTCTCATCTGTGACTGTGAGTTCAATGAA 26048

Lsal_LAC008020 25998 TCTATATATATAAGTCCTTCTGTCTCATCTGTGACTGTGAGTTCAATGAA 26047

Lsat_Salinas 26051 TAAACGGATGAAGTCAATAAAAAAATCGAAGAATTCAAAGAGCGTTTCGG 26100

Lsat_WENDEL 26051 TAAACGGATGAAGTCAATAAAAAAATCGAAGAATTCAAAGAGCGTTTCGG 26100

Lser_US96UC23 26051 TAAACGGATGAAGTCAATAAAAAAATCGAAGAATTCAAAGAGCGTTTCGG 26100

Lser_LAC005780 26051 TAAACGGATGAAGTCAATAAAAAAATCGAAGAATTCAAAGAGCGTTTCGG 26100

Lvir_CGN013357 26049 TAAACGGATGAAGTCAATAAAAAAATCGAAGAATTCAAAGAGCGTTTCGG 26098

Lsal_LAC008020 26048 TAAACGGATGAAGTCAATAAAAAAATCGAAGAATTCAAAGAGCGTTTCGG 26097

Lsat_Salinas 26101 GACAAAGAAACGGAAAAACTAAAGTTGTATGGATTCACAAAGACTTTCTC 26150

Lsat_WENDEL 26101 GACAAAGAAACGGAAAAACTAAAGTTGTATGGATTCACAAAGACTTTCTC 26150

Lser_US96UC23 26101 GACAAAGAAACGGAAAAACTAAAGTTGTATGGATTCACAAAGACTTTCTC 26150

Lser_LAC005780 26101 GACAAAGAAACGGAAAAACTAAAGTTGTATGGATTCACAAAGACTTTCTC 26150

Lvir_CGN013357 26099 GACAAAGAAACGGAAAAACTAAAGTTGTATGGATTCACAAAGACTTTCTC 26148

Lsal_LAC008020 26098 GACAAAGAAACGGAAAAACTAAAGTTGTATGGATTCACAAAGACTTTCTC 26147

Lsat_Salinas 26151 GAGAGAAACTAAAAAAGAGATATCAAAGTCGTTTTGATGATTCAAGAATG 26200

Lsat_WENDEL 26151 GAGAGAAACTAAAAAAGAGATATCAAAGTCGTTTTGATGATTCAAGAATG 26200

Lser_US96UC23 26151 GAGAGAAACTAAAAAAGAGATATCAAAGTCGTTTTGATGATTCAAGAATG 26200

Lser_LAC005780 26151 GAGAGAAACTAAAAAAGAGATATCAAAGTCGTTTTGATGATTCAAGAATG 26200

Lvir_CGN013357 26149 GAGAGAAACTAAAAAAGAGATATCAAAGTCGTTTTGATGATTCAAGAATG 26198

Lsal_LAC008020 26148 GAGAGAAACTAAAAAAGAGATATCAAAGTCGTTTTGATGATTCAAGAATG 26197

Lsat_Salinas 26201 TTATTACTTAAATTCAAAGTTCGTAGTTACTTCGACTGTATGAATCGTAG 26250

Lsat_WENDEL 26201 TTATTACTTAAATTCAAAGTTCGTAGTTACTTCGACTGTATGAATCGTAG 26250

Lser_US96UC23 26201 TTATTACTTAAATTCAAAGTTCGTAGTTACTTCGACTGTATGAATCGTAG 26250

Lser_LAC005780 26201 TTATTACTTAAATTCAAAGTTCGTAGTTACTTCGACTGTATGAATCGTAG 26250

Lvir_CGN013357 26199 TTATTACTTAAATTCAAAGTTCGTAGTTACTTCGACTGTATGAATCGTAG 26248

Lsal_LAC008020 26198 TTATTACTTAAATTCAAAGTTCGTAGTTACTTCGACTGTATGAATCGTAG 26247

Lsat_Salinas 26251 CAATGGAATCATTAAGTCATAGTTCATTGGTTGAATGTATCATTAACCAT 26300

Lsat_WENDEL 26251 CAATGGAATCATTAAGTCATAGTTCATTGGTTGAATGTATCATTAACCAT 26300

Lser_US96UC23 26251 CAATGGAATCATTAAGTCATAGTTCATTGGTTGAATGTATCATTAACCAT 26300

Lser_LAC005780 26251 CAATGGAATCATTAAGTCATAGTTCATTGGTTGAATGTATCATTAACCAT 26300

Lvir_CGN013357 26249 CAATGGAATCATTAAGTCATAGTTCATTGGTTGAATGTATCATTAACCAT 26298

Lsal_LAC008020 26248 CAATGGAATCATTAAGTCATAGTTCATTGGTTGAATGTATCATTAACCAT 26297

Lsat_Salinas 26301 TTTTTTTTGGTACGAGGAACTTATCATGAATCCACTGATTTCTGCCGCTT 26350

Lsat_WENDEL 26301 TTTTTTTTGGTACGAGGAACTTATCATGAATCCACTGATTTCTGCCGCTT 26350

Lser_US96UC23 26301 TTTTTTTTGGTACGAGGAACTTATCATGAATCCACTGATTTCTGCCGCTT 26350

Lser_LAC005780 26301 TTTTTTTTGGTACGAGGAACTTATCATGAATCCACTGATTTCTGCCGCTT 26350

Lvir_CGN013357 26299 TTTTTTTTGGTACGAGGAACTTATCATGAATCCACTGATTTCTGCCGCTT 26348

Lsal_LAC008020 26298 TTTTTTTTGGTACGAGGAACTTATCATGAATCCACTGATTTCTGCCGCTT 26347

Lsat_Salinas 26351 CCGTTATTGCTGCTGGATTGGCTGTAGGGCTTGCTTCTATTGGACCTGGA 26400

Lsat_WENDEL 26351 CCGTTATTGCTGCTGGATTGGCTGTAGGGCTTGCTTCTATTGGACCTGGA 26400

Lser_US96UC23 26351 CCGTTATTGCTGCTGGATTGGCTGTAGGGCTTGCTTCTATTGGACCTGGA 26400

Lser_LAC005780 26351 CCGTTATTGCTGCTGGATTGGCTGTAGGGCTTGCTTCTATTGGACCTGGA 26400

Lvir_CGN013357 26349 CCGTTATTGCTGCTGGATTGGCTGTAGGGCTTGCTTCTATTGGACCTGGA 26398

Lsal_LAC008020 26348 CCGTTATTGCTGCTGGATTGGCTGTAGGGCTTGCTTCTATTGGACCTGGA 26397

Lsat_Salinas 26401 GTTGGTCAAGGTACTGCTGCGGGTCAAGCTGTAGAAGGTATCGCGAGACA 26450

Lsat_WENDEL 26401 GTTGGTCAAGGTACTGCTGCGGGTCAAGCTGTAGAAGGTATCGCGAGACA 26450

Lser_US96UC23 26401 GTTGGTCAAGGTACTGCTGCGGGTCAAGCTGTAGAAGGTATCGCGAGACA 26450

Lser_LAC005780 26401 GTTGGTCAAGGTACTGCTGCGGGTCAAGCTGTAGAAGGTATCGCGAGACA 26450

Lvir_CGN013357 26399 GTTGGTCAAGGTACTGCTGCGGGTCAAGCTGTAGAAGGTATCGCGAGACA 26448

Lsal_LAC008020 26398 GTTGGTCAAGGTACTGCTGCGGGTCAAGCTGTAGAAGGTATCGCGAGACA 26447

Lsat_Salinas 26451 GCCCGAGGCGGAGGGAAAAATACGAGGTACTTTATTGCTTAGTCTAGCTT 26500

Lsat_WENDEL 26451 GCCCGAGGCGGAGGGAAAAATACGAGGTACTTTATTGCTTAGTCTAGCTT 26500

Lser_US96UC23 26451 GCCCGAGGCGGAGGGAAAAATACGAGGTACTTTATTGCTTAGTCTAGCTT 26500

Lser_LAC005780 26451 GCCCGAGGCGGAGGGAAAAATACGAGGTACTTTATTGCTTAGTCTAGCTT 26500

Lvir_CGN013357 26449 GCCCGAGGCGGAGGGAAAAATACGAGGTACTTTATTGCTTAGTCTAGCTT 26498

Lsal_LAC008020 26448 GCCCGAGGCGGAGGGAAAAATACGAGGTACTTTATTGCTTAGTCTAGCTT 26497

Lsat_Salinas 26501 TTATGGAAGCTTTAACCATTTATGGCCTGGTTGTAGCATTAGCACTTTTA 26550

Lsat_WENDEL 26501 TTATGGAAGCTTTAACCATTTATGGCCTGGTTGTAGCATTAGCACTTTTA 26550

Lser_US96UC23 26501 TTATGGAAGCTTTAACCATTTATGGCCTGGTTGTAGCATTAGCACTTTTA 26550

Lser_LAC005780 26501 TTATGGAAGCTTTAACCATTTATGGCCTGGTTGTAGCATTAGCACTTTTA 26550

Lvir_CGN013357 26499 TTATGGAAGCTTTAACCATTTATGGCCTGGTTGTAGCATTAGCACTTTTA 26548

Lsal_LAC008020 26498 TTATGGAAGCTTTAACCATTTATGGCCTGGTTGTAGCATTAGCACTTTTA 26547

Lsat_Salinas 26551 TTTGCGAATCCTTTTGTTTAATATTCGAAATATGAAAAATCAAAATTTTT 26600

Lsat_WENDEL 26551 TTTGCGAATCCTTTTGTTTAATATTCGAAATATGAAAAATCAAAATTTTT 26600

Lser_US96UC23 26551 TTTGCGAATCCTTTTGTTTAATATTCGAAATATGAAAAATCAAAATTTTT 26600

Lser_LAC005780 26551 TTTGCGAATCCTTTTGTTTAATATTCGAAATATGAAAAATCAAAATTTTT 26600

Lvir_CGN013357 26549 TTTGCGAATCCTTTTGTTTAATATTCGAAATATGAAAAATCAAAATTTTT 26598

Lsal_LAC008020 26548 TTTGCGAATCCTTTTGTTTAATATTCGAAATATGAAAAATCAAAATTTTT 26597

Lsat_Salinas 26601 CATATTTTATTGCCTTGGACTTGTGCTTTGCTTTTTCGAATTAAATAAAG 26650

Lsat_WENDEL 26601 CATATTTTATTGCCTTGGACTTGTGCTTTGCTTTTTCGAATTAAATAAAG 26650

Lser_US96UC23 26601 CATATTTTATTGCCTTGGACTTGTGCTTTGCTTTTTCGAATTAAATAAAG 26650

Lser_LAC005780 26601 CATATTTTATTGCCTTGGACTTGTGCTTTGCTTTTTCGAATTAAATAAAG 26650

Lvir_CGN013357 26599 CATATTTTATTGCCTTGGACTTGTGCTTTGCTTTTTCGAATTAAATAAAG 26648

Lsal_LAC008020 26598 CATATTTTATTGCCTTGGACTTGTGCTTTGCTTTTTCGAATTAAATAAAG 26647

Lsat_Salinas 26651 ATTTCATTCCTAGAATTACTTATTCGTTGAGAAAATAACCCACGGGAAGG 26700

Lsat_WENDEL 26651 ATTTCATTCCTAGAATTACTTATTCGTTGAGAAAATAACCCACGGGAAGG 26700

Lser_US96UC23 26651 ATTTCATTCCTAGAATTACTTATTCGTTGAGAAAATAACCCACGGGAAGG 26700

Lser_LAC005780 26651 ATTTCATTCCTAGAATTACTTATTCGTTGAGAAAATAACCCACGGGAAGG 26700

Lvir_CGN013357 26649 ATTTCATTCCTAGAATTACTTATTCGTTGAGAAAATAACCCACGGGAAGG 26698

Lsal_LAC008020 26648 ATTTCATTCCTAGAATTACTTATTCGTTGAGAAAATAACCCACGGGAAGG 26697

Lsat_Salinas 26701 GCTGATTTGCGGATGAGGAATTAGCATACCAACTTGCTTTCATCCTTCCC 26750

Lsat_WENDEL 26701 GCTGATTTGCGGATGAGGAATTAGCATACCAACTTGCTTTCATCCTTCCC 26750

Lser_US96UC23 26701 GCTGATTTGCGGATGAGGAATTAGCATACCAACTTGCTTTCATCCTTCCC 26750

Lser_LAC005780 26701 GCTGATTTGCGGATGAGGAATTAGCATACCAACTTGCTTTCATCCTTCCC 26750

Lvir_CGN013357 26699 GCTGATTTGCGGATGAGTAATTAGCATACCAACTCGCTTTCATCCTTCCC 26748

Lsal_LAC008020 26698 GCTGATTTGCGGATGAGGAATTAGCATACCAACTCGCTTTCATCCTTCCC 26747

Lsat_Salinas 26751 GTTCGTGTTCGTAGGCCTTTTTTTAGTTTTTAAGAGGGGTTGCAACCAAG 26800

Lsat_WENDEL 26751 GTTCGTGTTCGTAGGCCTTTTTTTAGTTTTTAAGAGGGGTTGCAACCAAG 26800

Lser_US96UC23 26751 GTTCGTGTTCGTAGGCCTTTTTTTAGTTTTTAAGAGGGGTTGCAACCAAG 26800

Lser_LAC005780 26751 GTTCGTGTTCGTAGGCCTTTTTTTAGTTTTTAAGAGGGGTTGCAACCAAG 26800

Lvir_CGN013357 26749 GTTCGTGTTCGTAGGCCTTTTTTTAGTTTTTAAGAGGGGTTGCAACCAAG 26798

Lsal_LAC008020 26748 GTTCGTGTTCGTAGGCCTTTTTTTAGTTTTTAAGAGGGGTTGCAACCAAG 26797

Lsat_Salinas 26801 GAGGTAATTCATTATTTCTAAATCGAATAAAAAATCTATTCTATTTAGAA 26850

Lsat_WENDEL 26801 GAGGTAATTCATTATTTCTAAATCGAATAAAAAATCTATTCTATTTAGAA 26850

Lser_US96UC23 26801 GAGGTAATTCATTATTTCTAAATCGAATAAAAAATCTATTCTATTTAGAA 26850

Lser_LAC005780 26801 GAGGTAATTCATTATTTCTAAATCGAATAAAAAATCTATTCTATTTAGAA 26850

Lvir_CGN013357 26799 GAGGTAATTCATTATTTCTAAATCGAATCAAAAATCTATTCTATTTAGAA 26848

Lsal_LAC008020 26798 GAGGTAATTCATTATTTCTAAATCGAATCAAAAATCTATTCTATTTAGAA 26847

Lsat_Salinas 26851 AGTAGGAAACTAGCAATATCAATATATATAAGAGGGCAAAGTAATACAAA 26900

Lsat_WENDEL 26851 AGTAGGAAACTAGCAATATCAATATATATAAGAGGGCAAAGTAATACAAA 26900

Lser_US96UC23 26851 AGTAGGAAACTAGCAATATCAATATATATAAGAGGGCAAAGTAATACAAA 26900

Lser_LAC005780 26851 AGTAGGAAACTAGCAATATCAATATATATAAGAGGGCAAAGTAATACAAA 26900

Lvir_CGN013357 26849 AGTAGGAAACTAGCAATATCAATATATAWAAGAGGGCAAAGTAATACAAA 26898

Lsal_LAC008020 26848 AGTAGGAAACTAGCAATATCAATATATAAAAGAGGGCAAAGTAATACAAA 26897

Lsat_Salinas 26901 AAGAACTCTGTTCGATTTTTTAGTCTATCTATACGAGGAGATCATATGAA 26950

Lsat_WENDEL 26901 AAGAACTCTGTTCGATTTTTTAGTCTATCTATACGAGGAGATCATATGAA 26950

Lser_US96UC23 26901 AAGAACTCTGTTCGATTTTTTAGTCTATCTATACGAGGAGATCATATGAA 26950

Lser_LAC005780 26901 AAGAACTCTGTTCGATTTTTTAGTCTATCTATACGAGGAGATCATATGAA 26950

Lvir_CGN013357 26899 AAGAACTCTGTTCGATTTTTTAGTCTATCTATACGAGGAGATCATATGAA 26948

Lsal_LAC008020 26898 AAGAACTCTGTTCAATTTTTTAGTCTATCTATACGAGGAGATCATATGAA 26947

Lsat_Salinas 26951 AAATGTAACCGATTCTTTCGTTTCTTTGGGCCACTGGCCATCCGCCGGGA 27000

Lsat_WENDEL 26951 AAATGTAACCGATTCTTTCGTTTCTTTGGGCCACTGGCCATCCGCCGGGA 27000

Lser_US96UC23 26951 AAATGTAACCGATTCTTTCGTTTCTTTGGGCCACTGGCCATCCGCCGGGA 27000

Lser_LAC005780 26951 AAATGTAACCGATTCTTTCGTTTCTTTGGGCCACTGGCCATCCGCCGGGA 27000

Lvir_CGN013357 26949 AAATGTAACCGATTCTTTCGTTTCTTTGGGCCACTGGCCATCCGCCGGGA 26998

Lsal_LAC008020 26948 AAATGTAACCGATTCTTTCGTTTCTTTGGGCCACTGGCCATCCGCCGGGA 26997

Lsat_Salinas 27001 GTTTCGGGTTTAATACCGATATTTTAGCAACAAATCTAATAAATCTAAGT 27050

Lsat_WENDEL 27001 GTTTCGGGTTTAATACCGATATTTTAGCAACAAATCTAATAAATCTAAGT 27050

Lser_US96UC23 27001 GTTTCGGGTTTAATACCGATATTTTAGCAACAAATCTAATAAATCTAAGT 27050

Lser_LAC005780 27001 GTTTCGGGTTTAATACCGATATTTTAGCAACAAATCTAATAAATCTAAGT 27050

Lvir_CGN013357 26999 GTTTCGGGTTTAATACCGATATTTTAGCAACAAATCTAATAAATCTAAGT 27048

Lsal_LAC008020 26998 GTTTCGGGTTTAATACCGATATTTTAGCAACAAATCTAATAAATCTAAGT 27047

Lsat_Salinas 27051 GTAGTGCTTGGGGTCTTGATCTTTTTTGGAAAGGGAGTGTGTGCGAGTTG 27100

Lsat_WENDEL 27051 GTAGTGCTTGGGGTCTTGATCTTTTTTGGAAAGGGAGTGTGTGCGAGTTG 27100

Lser_US96UC23 27051 GTAGTGCTTGGGGTCTTGATCTTTTTTGGAAAGGGAGTGTGTGCGAGTTG 27100

Lser_LAC005780 27051 GTAGTGCTTGGGGTCTTGATCTTTTTTGGAAAGGGAGTGTGTGCGAGTTG 27100

Lvir_CGN013357 27049 GTAGTGCTTGGGGTCTTGATCTTTTTTGGAAAGGGAGTGTGTGCGAGTTG 27098

Lsal_LAC008020 27048 GTAGTGCTTGGGGTCTTGATCTTTTTTGGAAAGGGAGTGTGTGCGAGTTG 27097

Lsat_Salinas 27101 TTTATTTCAAGAATAGGCTGGATCCAACCAGATGTACTTTTTCTGTTATA 27150

Lsat_WENDEL 27101 TTTATTTCAAGAATAGGCTGGATCCAACCAGATGTACTTTTTCTGTTATA 27150

Lser_US96UC23 27101 TTTATTTCAAGAATAGGCTGGATCCAACCAGATGTACTTTTTCTGTTATA 27150

Lser_LAC005780 27101 TTTATTTCAAGAATAGGCTGGATCCAACCAGATGTACTTTTTCTGTTATA 27150

Lvir_CGN013357 27099 TTTATTTCAAGAATAGGCTGGATCCAACCAGATGTACTTTTTCTGTTATA 27148

Lsal_LAC008020 27098 TTTATTTCAAGAATAGGCTGGATCGAACCAGATGTACTTTTTCTGTTATA 27147

Lsat_Salinas 27151 ACTAGGAAAGTTATACCTAATAAAGAAGGGTGCATGATCTCGCGAATTAC 27200

Lsat_WENDEL 27151 ACTAGGAAAGTTATACCTAATAAAGAAGGGTGCATGATCTCGCGAATTAC 27200

Lser_US96UC23 27151 ACTAGGAAAGTTATACCTAATAAAGAAGGGTGCATGATCTCGCGAATTAC 27200

Lser_LAC005780 27151 ACTAGGAAAGTTATACCTAATAAAGAAGGGTGCATGATCTCGCGAATTAC 27200

Lvir_CGN013357 27149 ACTAGGAAAGTTATACCTAATAAAGAAGGGTGCATGATCTCGCGAATTAC 27198

Lsal_LAC008020 27148 ACTAGGAAAGTTATACCTAATAAAGAAGGGTGCATGATCTCGCGAATTAC 27197

Lsat_Salinas 27201 TTCTGAATAAATTCAGAAATCATATGTAAGAACTATAGCATTTCGTAATT 27250

Lsat_WENDEL 27201 TTCTGAATAAATTCAGAAATCATATGTAAGAACTATAGCATTTCGTAATT 27250

Lser_US96UC23 27201 TTCTGAATAAATTCAGAAATCATATGTAAGAACTATAGCATTTCGTAATT 27250

Lser_LAC005780 27201 TTCTGAATAAATTCAGAAATCATATGTAAGAACTATAGCATTTCGTAATT 27250

Lvir_CGN013357 27199 TTCTGAATAAATTCAGAAATCATATGTAAGAACTATAGCATTTCGTAATT 27248

Lsal_LAC008020 27198 TTCTGAATAAATTCAGAAATCATATGTAAGAACTATAGCATTTCGTAATT 27247

Lsat_Salinas 27251 TATTGGAAAATCCACTTTGATTCTCTATCAACCAATAATGTGGGACCGTT 27300

Lsat_WENDEL 27251 TATTGGAAAATCCACTTTGATTCTCTATCAACCAATAATGTGGGACCGTT 27300

Lser_US96UC23 27251 TATTGGAAAATCCACTTTGATTCTCTATCAACCAATAATGTGGGACCGTT 27300

Lser_LAC005780 27251 TATTGGAAAATCCACTTTGATTCTCTATCAACCAATAATGTGGGACCGTT 27300

Lvir_CGN013357 27249 TATTGGAAAATCCACTTTGATTCTCTATCAACCAATAATGTGGGACCGTT 27298

Lsal_LAC008020 27248 TATTGGAAAATCCACTTTGATTCTCTATCAACCAATAATGTGGGACCGTT 27297

Lsat_Salinas 27301 CACATGGTTAAAGCTAAACTGTTTGAAGTCCAGACGCAGCATGGTACTCT 27350

Lsat_WENDEL 27301 CACATGGTTAAAGCTAAACTGTTTGAAGTCCAGACGCAGCATGGTACTCT 27350

Lser_US96UC23 27301 CACATGGTTAAAGCTAAACTGTTTGAAGTCCAGACGCAGCATGGTACTCT 27350

Lser_LAC005780 27301 CACATGGTTAAAGCTAAACTGTTTGAAGTCCAGACGCAGCATGGTACTCT 27350

Lvir_CGN013357 27299 CACATGGTTAAAGCTAAACTGTTTGAAGTCCAGACGCAGCATGGTACTCT 27348

Lsal_LAC008020 27298 CACATGGTTAAAGCTAAACTGTTTGAAGTCCAGACGCAGCATGGTACTCT 27347

Lsat_Salinas 27351 TTCGACCACTATGTTAATGTTAATATAGAGATGGTTTCAAAATAAAGATT 27400

Lsat_WENDEL 27351 TTCGACCACTATGTTAATGTTAATATAGAGATGGTTTCAAAATAAAGATT 27400

Lser_US96UC23 27351 TTCGACCACTATGTTAATGTTAATATAGAGATGGTTTCAAAATAAAGATT 27400

Lser_LAC005780 27351 TTCGACCACTATGTTAATGTTAATATAGAGATGGTTTCAAAATAAAGATT 27400

Lvir_CGN013357 27349 TTCGACCACTATGTTAATGTTAATATAGAGATGGTTTCAAAATAAAGATT 27398

Lsal_LAC008020 27348 TTCGACCACTATGTTAATGTTAATATAGAGATGGTTTCAAAATAAAGATT 27397

Lsat_Salinas 27401 TTATCAATATAGAACACTCATATCGATAAAATGATTTGAACTACTTAATT 27450

Lsat_WENDEL 27401 TTATCAATATAGAACACTCATATCGATAAAATGATTTGAACTACTTAATT 27450

Lser_US96UC23 27401 TTATCAATATAGAACACTCATATCGATAAAATGATTTGAACTACTTAATT 27450

Lser_LAC005780 27401 TTATCAATATAGAACACTCATATCGATAAAATGATTTGAACTACTTAATT 27450

Lvir_CGN013357 27399 TTATCAATATAGAACACTCATATCGATAAAATGATTTGAACTACTTAATT 27448

Lsal_LAC008020 27398 TTATCAATATAGAACACTCATATCGATAAAATGATTTGAACTACTTAATT 27447

Lsat_Salinas 27451 GGGGATTTTATCCCCTTTTTTAGCCAATGCTGAATCGATGACCTAGGTAT 27500

Lsat_WENDEL 27451 GGGGATTTTATCCCCTTTTTTAGCCAATGCTGAATCGATGACCTAGGTAT 27500

Lser_US96UC23 27451 GGGGATTTTATCCCCTTTTTTAGCCAATGCTGAATCGATGACCTAGGTAT 27500

Lser_LAC005780 27451 GGGGATTTTATCCCCTTTTTTAGCCAATGCTGAATCGATGACCTAGGTAT 27500

Lvir_CGN013357 27449 GGGGATTTTATCCCCTTTTTTAGCCAATGCTGAATCGATGACCTATGTAT 27498

Lsal_LAC008020 27448 GGGGATTTTATCCCCTTTTTTAGCCAATGCTGAATCGATGACCTATGTAT 27497

Lsat_Salinas 27501 TGTGTATAAAGTAAGAAAAACTTCTTGGATTAAAAAAAAAAGTAAACAAC 27550

Lsat_WENDEL 27501 TGTGTATAAAGTAAGAAAAACTTCTTGGATTAAAAAAAAAAGTAAACAAC 27550

Lser_US96UC23 27501 TGTGTATAAAGTAAGAAAAACTTCTTGGATTAAAAAAAAAAGTAAACAAC 27550

Lser_LAC005780 27501 TGTGTATAAAGTAAGAAAAACTTCTTGGATTAAAAAAAAAAGTAAACAAC 27550

Lvir_CGN013357 27499 TGTGTATAAAGTAAGAAAAACTTCTTGGATTAAAAAAAAAAGTAAACAAC 27548

Lsal_LAC008020 27498 TGTGTATAAAGTAAGAAAAACTTCTTGGATTAAAAAAAAAAGTAAACAAC 27547

Lsat_Salinas 27551 TTTGCTGACAATTACATATTTTTTGTTTGGTCAGAAGAGTCCTCCGAATA 27600

Lsat_WENDEL 27551 TTTGCTGACAATTACATATTTTTTGTTTGGTCAGAAGAGTCCTCCGAATA 27600

Lser_US96UC23 27551 TTTGCTGACAATTACATATTTTTTGTTTGGTCAGAAGAGTCCTCCGAATA 27600

Lser_LAC005780 27551 TTTGCTGACAATTACATATTTTTTGTTTGGTCAGAAGAGTCCTCCGAATA 27600

Lvir_CGN013357 27549 TTTGCTGACAATTACATATTTTTTGTTTGGTCAGAAGAGTCCTCCGAATA 27598

Lsal_LAC008020 27548 TTTGCTGACAATTACATATTTTTTGTTTGGTCAGAAGAGTCCTCCGAATA 27597

Lsat_Salinas 27601 TTTTGGTCTTGGATTAGTGATTCCTTTTGATATTTTGATTTCATTTTGGA 27650

Lsat_WENDEL 27601 TTTTGGTCTTGGATTAGTGATTCCTTTTGATATTTTGATTTCATTTTGGA 27650

Lser_US96UC23 27601 TTTTGGTCTTGGATTAGTGATTCCTTTTGATATTTTGATTTCATTTTGGA 27650

Lser_LAC005780 27601 TTTTGGTCTTGGATTAGTGATTCCTTTTGATATTTTGATTTCATTTTGGA 27650

Lvir_CGN013357 27599 TTTTGGTCTTGGATTAGTGATTCCTTTTGATATTTTGATTTCATTTTGGA 27648

Lsal_LAC008020 27598 TTTTGGTCTTGGATTAGTGATTCCTTTTGATATTTTGATTTCATTTTGGA 27647

Lsat_Salinas 27651 ATATGACAAGAGAATAGAGGATAGGCTCATTACATTAACAATAAAGATAT 27700

Lsat_WENDEL 27651 ATATGACAAGAGAATAGAGGATAGGCTCATTACATTAACAATAAAGATAT 27700

Lser_US96UC23 27651 ATATGACAAGAGAATAGAGGATAGGCTCATTACATTAACAATAAAGATAT 27700

Lser_LAC005780 27651 ATATGACAAGAGAATAGAGGATAGGCTCATTACATTAACAATAAAGATAT 27700

Lvir_CGN013357 27649 ATATGACAAGAGAATAGAGGATAGGCTCATTACATTAACAATAAAGATAT 27698

Lsal_LAC008020 27648 ATATGACAAGAGAATAGAGGATAGGCTCATTACATTAACAATAAAGATAT 27697

Lsat_Salinas 27701 GGGAATTTACATTGAGCGTGAGAGCCAAATGAATCGAAAGATTCATGTTT 27750

Lsat_WENDEL 27701 GGGAATTTACATTGAGCGTGAGAGCCAAATGAATCGAAAGATTCATGTTT 27750

Lser_US96UC23 27701 GGGAATTTACATTGAGCGTGAGAGCCAAATGAATCGAAAGATTCATGTTT 27750

Lser_LAC005780 27701 GGGAATTTACATTGAGCGTGAGAGCCAAATGAATCGAAAGATTCATGTTT 27750

Lvir_CGN013357 27699 GGGAATTTACATTGAGCGTGAGAGCCAAATGAATCGAAAGATTCATGTTT 27748

Lsal_LAC008020 27698 GGGAATTTACATTGAGCGTGAGAGCCAAATGAATCGAAAGATTCATGTTT 27747

Lsat_Salinas 27751 GGTTCGGGAAGGGATCATGGAATTTTTGAAATGAATGGAAAGATAATCTA 27800

Lsat_WENDEL 27751 GGTTCGGGAAGGGATCATGGAATTTTTGAAATGAATGGAAAGATAATCTA 27800

Lser_US96UC23 27751 GGTTCGGGAAGGGATCATGGAATTTTTGAAATGAATGGAAAGATAATCTA 27800

Lser_LAC005780 27751 GGTTCGGGAAGGGATCATGGAATTTTTGAAATGAATGGAAAGATAATCTA 27800

Lvir_CGN013357 27749 GGTTCGGGAAGGGATCATGGAATTTTTGAAATGAATGGAAAGATAATCTA 27798

Lsal_LAC008020 27748 GGTTCGGGAAGGGATCATGGAATTTTTGAAATGAATGGAAAGATAATCTA 27797

Lsat_Salinas 27801 CTTTCATTAAGTGATTTATTAGATAATCGAAAACAGAGAATCTTGAATAC 27850

Lsat_WENDEL 27801 CTTTCATTAAGTGATTTATTAGATAATCGAAAACAGAGAATCTTGAATAC 27850

Lser_US96UC23 27801 CTTTCATTAAGTGATTTATTAGATAATCGAAAACAGAGAATCTTGAATAC 27850

Lser_LAC005780 27801 CTTTCATTAAGTGATTTATTAGATAATCGAAAACAGAGAATCTTGAATAC 27850

Lvir_CGN013357 27799 CTTTCATTAAGTGATTTATTAGATAATCGAAAACAGAGAATCTTGAATAC 27848

Lsal_LAC008020 27798 CTTTCATTAAGTGATTTATTAGATAATCGAAAACAGAGAATCTTGAATAC 27847

Lsat_Salinas 27851 TATTAGAAATTCAGAAGAATTGCGCGAGGGGGCCATCGAACAGCTGGAAA 27900

Lsat_WENDEL 27851 TATTAGAAATTCAGAAGAATTGCGCGAGGGGGCCATCGAACAGCTGGAAA 27900

Lser_US96UC23 27851 TATTAGAAATTCAGAAGAATTGCGCGAGGGGGCCATCGAACAGCTGGAAA 27900

Lser_LAC005780 27851 TATTAGAAATTCAGAAGAATTGCGCGAGGGGGCCATCGAACAGCTGGAAA 27900

Lvir_CGN013357 27849 TATTAGAAATTCAGAAGAATTGCGCGAGGGGGCCATCGAACAGCTGGAAA 27898

Lsal_LAC008020 27848 TATTAGAAATTCAGAAGAATTGCGCGAGGGGGCCATCGAACAGCTGGAAA 27897

Lsat_Salinas 27901 AAGCCCGGGCTCGCTTACGGAAAGTAGAAATAGAAGCAGATCAGTTTCGC 27950

Lsat_WENDEL 27901 AAGCCCGGGCTCGCTTACGGAAAGTAGAAATAGAAGCAGATCAGTTTCGC 27950

Lser_US96UC23 27901 AAGCCCGGGCTCGCTTACGGAAAGTAGAAATAGAAGCAGATCAGTTTCGC 27950

Lser_LAC005780 27901 AAGCCCGGGCTCGCTTACGGAAAGTAGAAATAGAAGCAGATCAGTTTCGC 27950

Lvir_CGN013357 27899 AAGCCCGGGCTCGCTTACGGAAAGTAGAAATAGAAGCAGATCAGTTTCGC 27948

Lsal_LAC008020 27898 AAGCCCGGGCTCGCTTACGGAAAGTAGAAATAGAAGCAGATCAGTTTCGC 27947

Lsat_Salinas 27951 GTGAATGGATACTCTGAGATAGAGCGAGAAAAATTGAATTTGATTGATTC 28000

Lsat_WENDEL 27951 GTGAATGGATACTCTGAGATAGAGCGAGAAAAATTGAATTTGATTGATTC 28000

Lser_US96UC23 27951 GTGAATGGATACTCTGAGATAGAGCGAGAAAAATTGAATTTGATTGATTC 28000

Lser_LAC005780 27951 GTGAATGGATACTCTGAGATAGAGCGAGAAAAATTGAATTTGATTGATTC 28000

Lvir_CGN013357 27949 GTGAATGGATACTCTGAGATAGAGCGAGAAAAATTGAATTTGATTGATTC 27998

Lsal_LAC008020 27948 GTGAATGGATACTCTGAGATAGAGCGAGAAAAATTGAATTTGATTGATTC 27997

Lsat_Salinas 28001 AACTTATAAGACTTTGGAACAACTAGAAAATTACAAAAATGAAACTATAA 28050

Lsat_WENDEL 28001 AACTTATAAGACTTTGGAACAACTAGAAAATTACAAAAATGAAACTATAA 28050

Lser_US96UC23 28001 AACTTATAAGACTTTGGAACAACTAGAAAATTACAAAAATGAAACTATAA 28050

Lser_LAC005780 28001 AACTTATAAGACTTTGGAACAACTAGAAAATTACAAAAATGAAACTATAA 28050

Lvir_CGN013357 27999 AACTTATAAGACTTTGGAACAACTAGAAAATTACAAAAATGAAACTATAA 28048

Lsal_LAC008020 27998 AACTTATAAGACTTTGGAACAACTAGAAAATTACAAAAATGAAACTATAA 28047

Lsat_Salinas 28051 ATTTTGAACAACAAAAAGCGAGTAATCAAGTCCGACAACGGGTTTTCCAA 28100

Lsat_WENDEL 28051 ATTTTGAACAACAAAAAGCGAGTAATCAAGTCCGACAACGGGTTTTCCAA 28100

Lser_US96UC23 28051 ATTTTGAACAACAAAAAGCGAGTAATCAAGTCCGACAACGGGTTTTCCAA 28100

Lser_LAC005780 28051 ATTTTGAACAACAAAAAGCGAGTAATCAAGTCCGACAACGGGTTTTCCAA 28100

Lvir_CGN013357 28049 ATTTTGAACAACAAAAAGCGAGTAATCAAGTCCGACAACGGGTTTTCCAA 28098

Lsal_LAC008020 28048 ATTTTGAACAACAAAAAGCGAGTAATCAAGTCCGACAACGGGTTTTCCAA 28097

Lsat_Salinas 28101 CAAGCCTTACAAGGAGCTCTAGGAACTCTGAATAGTTGTTTAAACAGCGA 28150

Lsat_WENDEL 28101 CAAGCCTTACAAGGAGCTCTAGGAACTCTGAATAGTTGTTTAAACAGCGA 28150

Lser_US96UC23 28101 CAAGCCTTACAAGGAGCTCTAGGAACTCTGAATAGTTGTTTAAACAGCGA 28150

Lser_LAC005780 28101 CAAGCCTTACAAGGAGCTCTAGGAACTCTGAATAGTTGTTTAAACAGCGA 28150

Lvir_CGN013357 28099 CAAGCCTTACAAGGAGCTCTAGGAACTCTGAATAGTTGTTTAAACAGCGA 28148

Lsal_LAC008020 28098 CAAGCCTTACAAGGAGCTCTAGGAACTCTGAATAGTTGTTTAAACAGCGA 28147

Lsat_Salinas 28151 GTTACATTTACGTACTATCAGTGCCAATATTGGCATATTGGGGGCGATGA 28200

Lsat_WENDEL 28151 GTTACATTTACGTACTATCAGTGCCAATATTGGCATATTGGGGGCGATGA 28200

Lser_US96UC23 28151 GTTACATTTACGTACTATCAGTGCCAATATTGGCATATTGGGGGCGATGA 28200

Lser_LAC005780 28151 GTTACATTTACGTACTATCAGTGCCAATATTGGCATATTGGGGGCGATGA 28200

Lvir_CGN013357 28149 GTTACATTTACGTACTATCAGTGCCAATATTGGCATATTGGGGGCGATGA 28198

Lsal_LAC008020 28148 GTTACATTTACGTACTATCAGTGCCAATATTGGCATATTGGGGGCGATGA 28197

Lsat_Salinas 28201 AAGAAATAACGGATTAGTCCTTCTACTTTAGGCATTATTTTTGATTTATT 28250

Lsat_WENDEL 28201 AAGAAATAACGGATTAGTCCTTCTACTTTAGGCATTATTTTTGATTTATT 28250

Lser_US96UC23 28201 AAGAAATAACGGATTAGTCCTTCTACTTTAGGCATTATTTTTGATTTATT 28250

Lser_LAC005780 28201 AAGAAATAACGGATTAGTCCTTCTACTTTAGGCATTATTTTTGATTTATT 28250

Lvir_CGN013357 28199 AAGAAATAACGGATTAGTCCTTCTACTTTAGGCATTATTTTTGATTTATT 28248

Lsal_LAC008020 28198 AAGAAATAACGGATTAGTCCTTCTACTTTAGGCATTATTTTTGATTTATT 28247

Lsat_Salinas 28251 TTTTTTCCAAAAAAAGAATTAAGAAATACTCATGGTAACCATTCAAGCCG 28300

Lsat_WENDEL 28251 TTTTTTCCAAAAAAAGAATTAAGAAATACTCATGGTAACCATTCAAGCCG 28300

Lser_US96UC23 28251 TTTTTTCCAAAAAAAGAATTAAGAAATACTCATGGTAACCATTCAAGCCG 28300

Lser_LAC005780 28251 TTTTTTCCAAAAAAAGAATTAAGAAATACTCATGGTAACCATTCAAGCCG 28300

Lvir_CGN013357 28249 TTTTTTCCMAAAAAAGAATTAAGAAATACTCATGGTAACCATTCAAGCCG 28298

Lsal_LAC008020 28248 TTTTTTCCAAAAAAAGAATTAAGAAATACTCATGGTAACCATTCAAGCCG 28297

Lsat_Salinas 28301 ACGAAATTAGTAATATTATCCGTGAACGTATTGAGCAATATAATAGAGAA 28350

Lsat_WENDEL 28301 ACGAAATTAGTAATATTATCCGTGAACGTATTGAGCAATATAATAGAGAA 28350

Lser_US96UC23 28301 ACGAAATTAGTAATATTATCCGTGAACGTATTGAGCAATATAATAGAGAA 28350

Lser_LAC005780 28301 ACGAAATTAGTAATATTATCCGTGAACGTATTGAGCAATATAATAGAGAA 28350

Lvir_CGN013357 28299 ACGAAATTAGTAATATTATCCGTGAACGTATTGAGCAATATAATAGAGAA 28348

Lsal_LAC008020 28298 ACGAAATTAGTAATATTATCCGTGAACGTATTGAGCAATATAATAGAGAA 28347

Lsat_Salinas 28351 GTAAAGATTGTAAATACCGGTACCGTACTTCAAGTAGGTGATGGCATTGC 28400

Lsat_WENDEL 28351 GTAAAGATTGTAAATACCGGTACCGTACTTCAAGTAGGTGATGGCATTGC 28400

Lser_US96UC23 28351 GTAAAGATTGTAAATACCGGTACCGTACTTCAAGTAGGTGATGGCATTGC 28400

Lser_LAC005780 28351 GTAAAGATTGTAAATACCGGTACCGTACTTCAAGTAGGTGATGGCATTGC 28400

Lvir_CGN013357 28349 GTAAAGATTGTAAATACCGGTACCGTACTTCAAGTAGGTGATGGCATTGC 28398

Lsal_LAC008020 28348 GTAAAGATTGTAAATACCGGTACCGTACTTCAAGTAGGTGATGGCATTGC 28397

Lsat_Salinas 28401 TCGTATTCATGGTCTTGATGAAGTAATGGCGGGTGAATTAGTAGAATTTG 28450

Lsat_WENDEL 28401 TCGTATTCATGGTCTTGATGAAGTAATGGCGGGTGAATTAGTAGAATTTG 28450

Lser_US96UC23 28401 TCGTATTCATGGTCTTGATGAAGTAATGGCGGGTGAATTAGTAGAATTTG 28450

Lser_LAC005780 28401 TCGTATTCATGGTCTTGATGAAGTAATGGCGGGTGAATTAGTAGAATTTG 28450

Lvir_CGN013357 28399 TCGTATTCATGGTCTTGATGAAGTAATGGCGGGTGAATTAGTAGAATTTG 28448

Lsal_LAC008020 28398 TCGTATTCATGGTCTTGATGAAGTAATGGCGGGTGAATTAGTAGAATTTG 28447

Lsat_Salinas 28451 AAGAGGGTACAATAGGCATTGCTCTTAATTTGGAATCAACTAATGTTGGT 28500

Lsat_WENDEL 28451 AAGAGGGTACAATAGGCATTGCTCTTAATTTGGAATCAACTAATGTTGGT 28500

Lser_US96UC23 28451 AAGAGGGTACAATAGGCATTGCTCTTAATTTGGAATCAACTAATGTTGGT 28500

Lser_LAC005780 28451 AAGAGGGTACAATAGGCATTGCTCTTAATTTGGAATCAACTAATGTTGGT 28500

Lvir_CGN013357 28449 AAGAGGGTACAATAGGCATTGCTCTTAATTTGGAATCAACTAATGTTGGT 28498

Lsal_LAC008020 28448 AAGAGGGTACAATAGGCATTGCTCTTAATTTGGAATCAACTAATGTTGGT 28497

Lsat_Salinas 28501 GTTGTATTAATGGGTGATGGTTTGCTGATACAAGAAGGGAGTTCTGTAAA 28550

Lsat_WENDEL 28501 GTTGTATTAATGGGTGATGGTTTGCTGATACAAGAAGGGAGTTCTGTAAA 28550

Lser_US96UC23 28501 GTTGTATTAATGGGTGATGGTTTGCTGATACAAGAAGGGAGTTCTGTAAA 28550

Lser_LAC005780 28501 GTTGTATTAATGGGTGATGGTTTGCTGATACAAGAAGGGAGTTCTGTAAA 28550

Lvir_CGN013357 28499 GTTGTATTAATGGGTGATGGTTTGCTGATACAAGAAGGGAGTTCTGTAAA 28548

Lsal_LAC008020 28498 GTTGTATTAATGGGTGATGGTTTGCTGATACAAGAAGGGAGTTCTGTAAA 28547

Lsat_Salinas 28551 AGCAACAGGAAGAATTGCTCAGATACCAGTGAGTGAGGCCTATTTGGGTC 28600

Lsat_WENDEL 28551 AGCAACAGGAAGAATTGCTCAGATACCAGTGAGTGAGGCCTATTTGGGTC 28600

Lser_US96UC23 28551 AGCAACAGGAAGAATTGCTCAGATACCAGTGAGTGAGGCCTATTTGGGTC 28600

Lser_LAC005780 28551 AGCAACAGGAAGAATTGCTCAGATACCAGTGAGTGAGGCCTATTTGGGTC 28600

Lvir_CGN013357 28549 AGCAACAGGAAGAATTGCTCAGATACCAGTGAGTGAGGCCTATTTGGGTC 28598

Lsal_LAC008020 28548 AGCAACAGGAAGAATTGCTCAGATACCAGTGAGTGAGGCCTATTTGGGTC 28597

Lsat_Salinas 28601 GTGTTATAAACGCGCTGGCTAAACCTATTGATGGTAGAGGTGAAATTTCA 28650

Lsat_WENDEL 28601 GTGTTATAAACGCGCTGGCTAAACCTATTGATGGTAGAGGTGAAATTTCA 28650

Lser_US96UC23 28601 GTGTTATAAACGCGCTGGCTAAACCTATTGATGGTAGAGGTGAAATTTCA 28650

Lser_LAC005780 28601 GTGTTATAAACGCGCTGGCTAAACCTATTGATGGTAGAGGTGAAATTTCA 28650

Lvir_CGN013357 28599 GTGTTATAAACGCGCTGGCTAAACCTATTGATGGTAGAGGTGAAATTTCA 28648

Lsal_LAC008020 28598 GTGTTATAAACGCGCTGGCTAAACCTATTGATGGTAGAGGTGAAATTTCA 28647

Lsat_Salinas 28651 TCTTCTGAATATAGGTTAATTGAATCGCCCGCTCCAGGGATTATTTCTCG 28700

Lsat_WENDEL 28651 TCTTCTGAATATAGGTTAATTGAATCGCCCGCTCCAGGGATTATTTCTCG 28700

Lser_US96UC23 28651 TCTTCTGAATATAGGTTAATTGAATCGCCCGCTCCAGGGATTATTTCTCG 28700

Lser_LAC005780 28651 TCTTCTGAATATAGGTTAATTGAATCGCCCGCTCCAGGGATTATTTCTCG 28700

Lvir_CGN013357 28649 TCTTCTGAATATAGGTTAATTGAATCGCCCGCTCCAGGGATTATTTCTCG 28698

Lsal_LAC008020 28648 TCTTCTGAATATAGGTTAATTGAATCGCCCGCTCCAGGGATTATTTCTCG 28697

Lsat_Salinas 28701 ACGTTCTGTATATGAGCCTCTTCAAACAGGGCTTATTGCTATTGATTCAA 28750

Lsat_WENDEL 28701 ACGTTCTGTATATGAGCCTCTTCAAACAGGGCTTATTGCTATTGATTCAA 28750

Lser_US96UC23 28701 ACGTTCTGTATATGAGCCTCTTCAAACAGGGCTTATTGCTATTGATTCAA 28750

Lser_LAC005780 28701 ACGTTCTGTATATGAGCCTCTTCAAACAGGGCTTATTGCTATTGATTCAA 28750

Lvir_CGN013357 28699 ACGTTCTGTATATGAGCCTCTTCAAACAGGGCTTATTGCTATTGATTCAA 28748

Lsal_LAC008020 28698 ACGTTCTGTATATGAGCCTCTTCAAACAGGGCTTATTGCTATTGATTCAA 28747

Lsat_Salinas 28751 TGATTCCGATAGGACGTGGTCAGCGCGAATTAATTATTGGGGACAGGCAG 28800

Lsat_WENDEL 28751 TGATTCCGATAGGACGTGGTCAGCGCGAATTAATTATTGGGGACAGGCAG 28800

Lser_US96UC23 28751 TGATTCCGATAGGACGTGGTCAGCGCGAATTAATTATTGGGGACAGGCAG 28800

Lser_LAC005780 28751 TGATTCCGATAGGACGTGGTCAGCGCGAATTAATTATTGGGGACAGGCAG 28800

Lvir_CGN013357 28749 TGATTCCGATAGGACGTGGTCAGCGAGAATTAATTATTGGGGACAGGCAG 28798

Lsal_LAC008020 28748 TGATTCCGATAGGACGTGGTCAGCGAGAATTAATTATTGGGGACAGGCAG 28797

Lsat_Salinas 28801 ACCGGTAAAACAGCAGTAGCAACAGATACAATTCTAAATCAACAAGGCAA 28850

Lsat_WENDEL 28801 ACCGGTAAAACAGCAGTAGCAACAGATACAATTCTAAATCAACAAGGCAA 28850

Lser_US96UC23 28801 ACCGGTAAAACAGCAGTAGCAACAGATACAATTCTAAATCAACAAGGCAA 28850

Lser_LAC005780 28801 ACCGGTAAAACAGCAGTAGCAACAGATACAATTCTAAATCAACAAGGCAA 28850

Lvir_CGN013357 28799 ACCGGTAAAACAGCAGTAGCAACAGATACAATTCTAAATCAACAAGGCAA 28848

Lsal_LAC008020 28798 ACCGGTAAAACAGCAGTAGCAACAGATACAATTCTAAATCAACAAGGCAA 28847

Lsat_Salinas 28851 AAATGTAATATGCGTTTATGTAGCTATTGGTCAAAAAGCATCTTCTGTGG 28900

Lsat_WENDEL 28851 AAATGTAATATGCGTTTATGTAGCTATTGGTCAAAAAGCATCTTCTGTGG 28900

Lser_US96UC23 28851 AAATGTAATATGCGTTTATGTAGCTATTGGTCAAAAAGCATCTTCTGTGG 28900

Lser_LAC005780 28851 AAATGTAATATGCGTTTATGTAGCTATTGGTCAAAAAGCATCTTCTGTGG 28900

Lvir_CGN013357 28849 AAATGTAATATGCGTTTATGTAGCTATTGGTCAAAAAGCATCTTCTGTGG 28898

Lsal_LAC008020 28848 AAATGTAATATGCGTTTATGTAGCTATTGGTCAAAAAGCATCTTCTGTGG 28897

Lsat_Salinas 28901 CTCAGGTAGTGACTAATTTCCAGGAAAGGGGCGCGATGGAATATACCATT 28950

Lsat_WENDEL 28901 CTCAGGTAGTGACTAATTTCCAGGAAAGGGGCGCGATGGAATATACCATT 28950

Lser_US96UC23 28901 CTCAGGTAGTGACTAATTTCCAGGAAAGGGGCGCGATGGAATATACCATT 28950

Lser_LAC005780 28901 CTCAGGTAGTGACTAATTTCCAGGAAAGGGGCGCGATGGAATATACCATT 28950

Lvir_CGN013357 28899 CTCAGGTAGTGACTAATTTCCAGGAAAGGGGCGCGATGGAATATACCATT 28948

Lsal_LAC008020 28898 CTCAGGTAGTGACTAATTTCCAGGAAAGGGGCGCGATGGAATATACCATT 28947

Lsat_Salinas 28951 GTGGTAGCCGAAACGGCGGATTCCCCTGCTACATTACAATACCTCGCTCC 29000

Lsat_WENDEL 28951 GTGGTAGCCGAAACGGCGGATTCCCCTGCTACATTACAATACCTCGCTCC 29000

Lser_US96UC23 28951 GTGGTAGCCGAAACGGCGGATTCCCCTGCTACATTACAATACCTCGCTCC 29000

Lser_LAC005780 28951 GTGGTAGCCGAAACGGCGGATTCCCCTGCTACATTACAATACCTCGCTCC 29000

Lvir_CGN013357 28949 GTGGTAGCCGAAACGGCGGATTCCCCTGCTACATTACAATACCTCGCTCC 28998

Lsal_LAC008020 28948 GTGGTAGCCGAAACGGCGGATTCCCCTGCTACATTACAATACCTCGCTCC 28997

Lsat_Salinas 29001 TTATACAGGAGCAGCTCTGGCTGAATATTTTATGTACCGTGAACGACACA 29050

Lsat_WENDEL 29001 TTATACAGGAGCAGCTCTGGCTGAATATTTTATGTACCGTGAACGACACA 29050

Lser_US96UC23 29001 TTATACAGGAGCAGCTCTGGCTGAATATTTTATGTACCGTGAACGACACA 29050

Lser_LAC005780 29001 TTATACAGGAGCAGCTCTGGCTGAATATTTTATGTACCGTGAACGACACA 29050

Lvir_CGN013357 28999 TTATACAGGAGCAGCTCTGGCTGAATATTTTATGTACCGTGAACGACACA 29048

Lsal_LAC008020 28998 TTATACAGGAGCAGCTCTGGCTGAATATTTTATGTACCGTGAACGACACA 29047

Lsat_Salinas 29051 CTTCAATCATTTATGATGATCCCTCTAAACAAGCCCAAGCTTATCGCCAA 29100

Lsat_WENDEL 29051 CTTCAATCATTTATGATGATCCCTCTAAACAAGCCCAAGCTTATCGCCAA 29100

Lser_US96UC23 29051 CTTCAATCATTTATGATGATCCCTCTAAACAAGCCCAAGCTTATCGCCAA 29100

Lser_LAC005780 29051 CTTCAATCATTTATGATGATCCCTCTAAACAAGCCCAAGCTTATCGCCAA 29100

Lvir_CGN013357 29049 CTTCAATCATTTATGATGATCTCTCTAAACAAGCCCAAGCTTATCGCCAA 29098

Lsal_LAC008020 29048 CTTCAATCATTTATGATGATCCCTCTAAACAAGCCCAAGCTTATCGCCAA 29097

Lsat_Salinas 29101 ATGTCTCTTCTATTACGAAGACCGCCTGGGCGCGAAGCTTATCCAGGGGA 29150

Lsat_WENDEL 29101 ATGTCTCTTCTATTACGAAGACCGCCTGGGCGCGAAGCTTATCCAGGGGA 29150

Lser_US96UC23 29101 ATGTCTCTTCTATTACGAAGACCGCCTGGGCGCGAAGCTTATCCAGGGGA 29150

Lser_LAC005780 29101 ATGTCTCTTCTATTACGAAGACCGCCTGGGCGCGAAGCTTATCCAGGGGA 29150

Lvir_CGN013357 29099 ATGTCTCTTCTATTACGAAGACCGCCTGGGCGCGAAGCTTATCCAGGGGA 29148

Lsal_LAC008020 29098 ATGTCTCTTCTATTACGAAGACCGCCTGGGCGCGAAGCTTATCCAGGGGA 29147

Lsat_Salinas 29151 TGTTTTTTATTTACATTCACGCCTTTTGGAAAGAGCTGCTAAATTAAGTT 29200

Lsat_WENDEL 29151 TGTTTTTTATTTACATTCACGCCTTTTGGAAAGAGCTGCTAAATTAAGTT 29200

Lser_US96UC23 29151 TGTTTTTTATTTACATTCACGCCTTTTGGAAAGAGCTGCTAAATTAAGTT 29200

Lser_LAC005780 29151 TGTTTTTTATTTACATTCACGCCTTTTGGAAAGAGCTGCTAAATTAAGTT 29200

Lvir_CGN013357 29149 TGTTTTTTATTTACATTCACGCCTTTTGGAAAGAGCTGCTAAATTAAGTT 29198

Lsal_LAC008020 29148 TGTTTTTTATTTACATTCACGCCTTTTGGAAAGAGCTGCTAAATTAAGTT 29197

Lsat_Salinas 29201 CTCTTTTAGGTGAAGGAAGTATGACCGCTTTACCAATAGTGGAAACCCAA 29250

Lsat_WENDEL 29201 CTCTTTTAGGTGAAGGAAGTATGACCGCTTTACCAATAGTGGAAACCCAA 29250

Lser_US96UC23 29201 CTCTTTTAGGTGAAGGAAGTATGACCGCTTTACCAATAGTGGAAACCCAA 29250

Lser_LAC005780 29201 CTCTTTTAGGTGAAGGAAGTATGACCGCTTTACCAATAGTGGAAACCCAA 29250

Lvir_CGN013357 29199 CTCTTTTAGGTGAAGGAAGTATGACCGCTTTACCAATAGTGGAAACCCAA 29248

Lsal_LAC008020 29198 CTCTTTTAGGTGAAGGAAGTATGACCGCTTTACCAATAGTGGAAACCCAA 29247

Lsat_Salinas 29251 TCGGGAGATGTTTCGGCTTATATTCCTACTAATGTCATTTCGATTACTGA 29300

Lsat_WENDEL 29251 TCGGGAGATGTTTCGGCTTATATTCCTACTAATGTCATTTCGATTACTGA 29300

Lser_US96UC23 29251 TCGGGAGATGTTTCGGCTTATATTCCTACTAATGTCATTTCGATTACTGA 29300

Lser_LAC005780 29251 TCGGGAGATGTTTCGGCTTATATTCCTACTAATGTCATTTCGATTACTGA 29300

Lvir_CGN013357 29249 TCGGGAGATGTTTCGGCTTATATTCCTACTAATGTCATTTCGATTACTGA 29298

Lsal_LAC008020 29248 TCGGGAGATGTTTCGGCTTATATTCCTACTAATGTCATTTCGATTACTGA 29297

Lsat_Salinas 29301 TGGACAAATATTCTTATCTGCTGATCTATTCAATGCTGGAATCCGACCCG 29350

Lsat_WENDEL 29301 TGGACAAATATTCTTATCTGCTGATCTATTCAATGCTGGAATCCGACCCG 29350

Lser_US96UC23 29301 TGGACAAATATTCTTATCTGCTGATCTATTCAATGCTGGAATCCGACCCG 29350

Lser_LAC005780 29301 TGGACAAATATTCTTATCTGCTGATCTATTCAATGCTGGAATCCGACCCG 29350

Lvir_CGN013357 29299 TGGACAAATATTCTTATCTGCTGATCTATTCAATGCTGGAATCCGACCCG 29348

Lsal_LAC008020 29298 TGGACAAATATTCTTATCTGCTGATCTATTCAATGCTGGAATCCGACCCG 29347

Lsat_Salinas 29351 CTATTAATGTGGGGATCTCTGTTTCCAGAGTGGGGTCTGCAGCTCAAATT 29400

Lsat_WENDEL 29351 CTATTAATGTGGGGATCTCTGTTTCCAGAGTGGGGTCTGCAGCTCAAATT 29400

Lser_US96UC23 29351 CTATTAATGTGGGGATCTCTGTTTCCAGAGTGGGGTCTGCAGCTCAAATT 29400

Lser_LAC005780 29351 CTATTAATGTGGGGATCTCTGTTTCCAGAGTGGGGTCTGCAGCTCAAATT 29400

Lvir_CGN013357 29349 CTATTAATGTGGGGATCTCTGTTTCCAGAGTGGGGTCTGCAGCTCAAATT 29398

Lsal_LAC008020 29348 CTATTAATGTGGGGATCTCTGTTTCCAGAGTGGGGTCTGCAGCTCAAATT 29397

Lsat_Salinas 29401 AAAGCTATGAAACAAGTAGCCGGTAAATTAAAATTGGAACTGGCACAATT 29450

Lsat_WENDEL 29401 AAAGCTATGAAACAAGTAGCCGGTAAATTAAAATTGGAACTGGCACAATT 29450

Lser_US96UC23 29401 AAAGCTATGAAACAAGTAGCCGGTAAATTAAAATTGGAACTGGCACAATT 29450

Lser_LAC005780 29401 AAAGCTATGAAACAAGTAGCCGGTAAATTAAAATTGGAACTGGCACAATT 29450

Lvir_CGN013357 29399 AAAGCTATGAAACAAGTAGCCGGTAAATTAAAATTGGAACTGGCACAATT 29448

Lsal_LAC008020 29398 AAAGCTATGAAACAAGTAGCCGGTAAATTAAAATTGGAACTGGCACAATT 29447

Lsat_Salinas 29451 CGCAGAATTAGAAGCTTTTGCACAATTTGCTTCTGATCTCGATAAAGCTA 29500

Lsat_WENDEL 29451 CGCAGAATTAGAAGCTTTTGCACAATTTGCTTCTGATCTCGATAAAGCTA 29500

Lser_US96UC23 29451 CGCAGAATTAGAAGCTTTTGCACAATTTGCTTCTGATCTCGATAAAGCTA 29500

Lser_LAC005780 29451 CGCAGAATTAGAAGCTTTTGCACAATTTGCTTCTGATCTCGATAAAGCTA 29500

Lvir_CGN013357 29449 CGCAGAATTAGAAGCTTTTGCACAATTTGCTTCTGATCTCGATAAAGCTA 29498

Lsal_LAC008020 29448 CGCAGAATTAGAAGCTTTTGCACAATTTGCTTCTGATCTCGATAAAGCTA 29497

Lsat_Salinas 29501 CTCAGAATCAATTGGCAAGAGGTCAACGCTTACGTGAATTGCTTAAACAA 29550

Lsat_WENDEL 29501 CTCAGAATCAATTGGCAAGAGGTCAACGCTTACGTGAATTGCTTAAACAA 29550

Lser_US96UC23 29501 CTCAGAATCAATTGGCAAGAGGTCAACGCTTACGTGAATTGCTTAAACAA 29550

Lser_LAC005780 29501 CTCAGAATCAATTGGCAAGAGGTCAACGCTTACGTGAATTGCTTAAACAA 29550

Lvir_CGN013357 29499 CTCAGAATCAATTGGCAAGAGGTCAACGCTTACGTGAATTGCTTAAACAA 29548

Lsal_LAC008020 29498 CTCAGAATCAATTGGCAAGAGGTCAACGCTTACGTGAATTGCTTAAACAA 29547

Lsat_Salinas 29551 TCCCAATCCGCCCCTCTCGGGGTAGAAGAACAGGTACTGACTATTTATAC 29600

Lsat_WENDEL 29551 TCCCAATCCGCCCCTCTCGGGGTAGAAGAACAGGTACTGACTATTTATAC 29600

Lser_US96UC23 29551 TCCCAATCCGCCCCTCTCGGGGTAGAAGAACAGGTACTGACTATTTATAC 29600

Lser_LAC005780 29551 TCCCAATCCGCCCCTCTCGGGGTAGAAGAACAGGTACTGACTATTTATAC 29600

Lvir_CGN013357 29549 TCCCAATCCGCCCCTCTCGGGGTAGAAGAACAGGTACTGACTATTTATAC 29598

Lsal_LAC008020 29548 TCCCAATCCGCCCCTCTCGGGGTAGAAGAACAGGTACTGACTATTTATAC 29597

Lsat_Salinas 29601 CGGAACAAATGGTTATCTTGATTCATTAGAAATTGGACAGGTAAGGAAAT 29650

Lsat_WENDEL 29601 CGGAACAAATGGTTATCTTGATTCATTAGAAATTGGACAGGTAAGGAAAT 29650

Lser_US96UC23 29601 CGGAACAAATGGTTATCTTGATTCATTAGAAATTGGACAGGTAAGGAAAT 29650

Lser_LAC005780 29601 CGGAACAAATGGTTATCTTGATTCATTAGAAATTGGACAGGTAAGGAAAT 29650

Lvir_CGN013357 29599 CGGAACAAATGGTTATCTTGATTCATTAGAAATTGGACAGGTAAGGAAAT 29648

Lsal_LAC008020 29598 CGGAACAAATGGTTATCTTGATTCATTAGAAATTGGACAGGTAAGGAAAT 29647

Lsat_Salinas 29651 TTCTTGTTGAGTTACGTACTTACTTAAAAACCAATAAACCGCAGTTTCAA 29700

Lsat_WENDEL 29651 TTCTTGTTGAGTTACGTACTTACTTAAAAACCAATAAACCGCAGTTTCAA 29700

Lser_US96UC23 29651 TTCTTGTTGAGTTACGTACTTACTTAAAAACCAATAAACCGCAGTTTCAA 29700

Lser_LAC005780 29651 TTCTTGTTGAGTTACGTACTTACTTAAAAACCAATAAACCGCAGTTTCAA 29700

Lvir_CGN013357 29649 TTCTTGTTGAGTTACGTACTTACTTAAAAACCAATAAACCGCAGTTTCAA 29698

Lsal_LAC008020 29648 TTCTTGTTGAGTTACGTACTTACTTAAAAACCAATAAACCGCAGTTTCAA 29697

Lsat_Salinas 29701 GAAATAATATCTTCTACCAAGACATTCACCGAGGAAGCAGAAGCCATTTT 29750

Lsat_WENDEL 29701 GAAATAATATCTTCTACCAAGACATTCACCGAGGAAGCAGAAGCCATTTT 29750

Lser_US96UC23 29701 GAAATAATATCTTCTACCAAGACATTCACCGAGGAAGCAGAAGCCATTTT 29750

Lser_LAC005780 29701 GAAATAATATCTTCTACCAAGACATTCACCGAGGAAGCAGAAGCCATTTT 29750

Lvir_CGN013357 29699 GAAATAATATCTTCTACCAAGACATTCACCGAGGAAGCAGAAGCCATTTT 29748

Lsal_LAC008020 29698 GAAATAATATCTTCTACCAAGACATTCACCGAGGAAGCAGAAGCCATTTT 29747

Lsat_Salinas 29751 GAAAGAAGCTATTAAGGAACAGAGGGAACGTTTTATACTTCAGGAACAAG 29800

Lsat_WENDEL 29751 GAAAGAAGCTATTAAGGAACAGAGGGAACGTTTTATACTTCAGGAACAAG 29800

Lser_US96UC23 29751 GAAAGAAGCTATTAAGGAACAGAGGGAACGTTTTATACTTCAGGAACAAG 29800

Lser_LAC005780 29751 GAAAGAAGCTATTAAGGAACAGAGGGAACGTTTTATACTTCAGGAACAAG 29800

Lvir_CGN013357 29749 GAAAGAAGCTATTAAGGAACAGAGGGAACGTTTTATACTTCAGGAACAAG 29798

Lsal_LAC008020 29748 GAAAGAAGCTATTAAGGAACAGAGGGAACGTTTTATACTTCAGGAACAAG 29797

Lsat_Salinas 29801 CAGCCTAAACAAATTGATCACCCTTTTCAATAATTTTTTAAATAAAATAA 29850

Lsat_WENDEL 29801 CAGCCTAAACAAATTGATCACCCTTTTCAATAATTTTTTAAATAAAATAA 29850

Lser_US96UC23 29801 CAGCCTAAACAAATTGATCACCCTTTTCAATAATTTTTTAAATAAAATAA 29850

Lser_LAC005780 29801 CAGCCTAAACAAATTGATCACCCTTTTCAATAATTTTTTAAATAAAATAA 29850

Lvir_CGN013357 29799 CAGCCTAAACAAATTGATCACCCTTTTCAATAATTTTTTAAATAAAATAA 29848

Lsal_LAC008020 29798 CAGCCTAAACAAATGGATCACCCTTTTCAATAATTTTTTAAATAAAATAA 29847

Lsat_Salinas 29851 AAAGGGTACTCAAGCGTTTCGGAGTCAAATCATTCAAAATTTATTTTTTG 29900

Lsat_WENDEL 29851 AAAGGGTACTCAAGCGTTTCGGAGTCAAATCATTCAAAATTTATTTTTTG 29900

Lser_US96UC23 29851 AAAGGGTACTCAAGCGTTTCGGAGTCAAATCATTCAAAATTTATTTTTTG 29900

Lser_LAC005780 29851 AAAGGGTACTCAAGCGTTTCGGAGTCAAATCATTCAAAATTTATTTTTTG 29900

Lvir_CGN013357 29849 AAAGGGTACTCAAGCGTTTCGGAGTCAAATCATTCAAAATTTATTTTTTG 29898

Lsal_LAC008020 29848 AAAGGGTACTCAAGCGTTTCGGAGTCAAATCATTCAAAATTTATTTTTTG 29897

Lsat_Salinas 29901 ATATCTAAATCTAAAAAAATATCTGGAAAAAAATTGCGTCCAATAGGATT 29950

Lsat_WENDEL 29901 ATATCTAAATCTAAAAAAATATCTGGAAAAAAATTGCGTCCAATAGGATT 29950

Lser_US96UC23 29901 ATATCTAAATCTAAAAAAATATCTGGAAAAAAATTGCGTCCAATAGGATT 29950

Lser_LAC005780 29901 ATATCTAAATCTAAAAAAATATCTGGAAAAAAATTGCGTCCAATAGGATT 29950

Lvir_CGN013357 29899 ATATCTAAATCTAAAAAAATATCTGGAAAAAAATTGCGTCCAATAGGATT 29948

Lsal_LAC008020 29898 ATATCTAAATCTAAAAAAATATCTGGAAAAAAATTGCGTCCAATAGGATT 29947

Lsat_Salinas 29951 TGAACCTATACCAAAGGTTTAGAAGACCTCTGTCCTATCCATTAGACAAT 30000

Lsat_WENDEL 29951 TGAACCTATACCAAAGGTTTAGAAGACCTCTGTCCTATCCATTAGACAAT 30000

Lser_US96UC23 29951 TGAACCTATACCAAAGGTTTAGAAGACCTCTGTCCTATCCATTAGACAAT 30000

Lser_LAC005780 29951 TGAACCTATACCAAAGGTTTAGAAGACCTCTGTCCTATCCATTAGACAAT 30000

Lvir_CGN013357 29949 TGAACCTATACCAAAGGTTTAGAAGACCTCTGTCCTATCCATTAGACAAT 29998

Lsal_LAC008020 29948 TGAACCTATACCAAAGGTTTAGAAGACCTCTGTCCTATCCATTAGACAAT 29997

Lsat_Salinas 30001 GGACGCTTTTCATTGCGATTTTTTAGTAGTTTTACTTTCTGATCTTTTAT 30050

Lsat_WENDEL 30001 GGACGCTTTTCATTGCGATTTTTTAGTAGTTTTACTTTCTGATCTTTTAT 30050

Lser_US96UC23 30001 GGACGCTTTTCATTGCGATTTTTTAGTAGTTTTACTTTCTGATCTTTTAT 30050

Lser_LAC005780 30001 GGACGCTTTTCATTGCGATTTTTTAGTAGTTTTACTTTCTGATCTTTTAT 30050

Lvir_CGN013357 29999 GGACGCTTTTCATTGCGATTTTTTAGTAGTTTTACTTTCTGATCTTTTAT 30048

Lsal_LAC008020 29998 GGACGCTTTTCATTGCGATTTTTTAGTAGTTTTACTTTCTGATCTTTTAT 30047

Lsat_Salinas 30051 TTGAAAAAATACGAATCTAATATAGTAGGATTTCTGTAAGAAAATTATTT 30100

Lsat_WENDEL 30051 TTGAAAAAATACGAATCTAATATAGTAGGATTTCTGTAAGAAAATTATTT 30100

Lser_US96UC23 30051 TTGAAAAAATACGAATCTAATATAGTAGGATTTCTGTAAGAAAATTATTT 30100

Lser_LAC005780 30051 TTGAAAAAATACGAATCTAATATAGTAGGATTTCTGTAAGAAAATTATTT 30100

Lvir_CGN013357 30049 TTGAAAAAATACGAATCTAATATAGTAGGATTTCTGTAAGAAAATTCTTT 30098

Lsal_LAC008020 30048 TTGAAAAAATACGAATCTAATATAGTAGGATTTCTGTAAGAAAATTCTTT 30097

Lsat_Salinas 30101 GAATTGTATATTCAATGATGAATGATGTATTAATTAGAGTCTATTAGAGT 30150

Lsat_WENDEL 30101 GAATTGTATATTCAATGATGAATGATGTATTAATTAGAGTCTATTAGAGT 30150

Lser_US96UC23 30101 GAATTGTATATTCAATGATGAATGATGTATTAATTAGAGTCTATTAGAGT 30150

Lser_LAC005780 30101 GAATTGTATATTCAATGATGAATGATGTATTAATTAGAGTCTATTAGAGT 30150

Lvir_CGN013357 30099 GAATTGTATATTCAATGATGAATGATGTATTAATTAGAGTCTATTAGAGT 30148

Lsal_LAC008020 30098 GAATTGTATATTCAATGATGAATGATGTATTAATTAGAGTCTATTAGAGT 30147

Lsat_Salinas 30151 ATTTAATAATATATAAATATAGTAAGATAATAGAATAAATCTATAAGTAG 30200

Lsat_WENDEL 30151 ATTTAATAATATATAAATATAGTAAGATAATAGAATAAATCTATAAGTAG 30200

Lser_US96UC23 30151 ATTTAATAATATATAAATATAGTAAGATAATAGAATAAATCTATAAGTAG 30200

Lser_LAC005780 30151 ATTTAATAATATATAAATATAGTAAGATAATAGAATAAATCTATAAGTAG 30200

Lvir_CGN013357 30149 ATTTAATAATATATAAATATAGTAAGATAATAGAATAAATCTATAAGTAG 30198

Lsal_LAC008020 30148 ATTTAATAATATATAAATATAGTAAGATAATAGAATAAATCTATAAGTAG 30197

Lsat_Salinas 30201 AGGCTATAGAGCGGGTAGCGGGAATCGAACCCGCATCGTTAGCTTGGAAG 30250

Lsat_WENDEL 30201 AGGCTATAGAGCGGGTAGCGGGAATCGAACCCGCATCGTTAGCTTGGAAG 30250

Lser_US96UC23 30201 AGGCTATAGAGCGGGTAGCGGGAATCGAACCCGCATCGTTAGCTTGGAAG 30250

Lser_LAC005780 30201 AGGCTATAGAGCGGGTAGCGGGAATCGAACCCGCATCGTTAGCTTGGAAG 30250

Lvir_CGN013357 30199 AGGCTATAGAGCGGGTAGCGGGAATCGAACCCGCATCGTTAGCTTGGAAG 30248

Lsal_LAC008020 30198 AGGCTATAGAGCGGGTAGCGGGAATCGAACCCGCATCRTYAGCTTGGAAG 30247

Lsat_Salinas 30251 GCTAGGGGTTATAGTCGACGTCGATTCATCATTTTTAACGTCTCTAATTC 30300

Lsat_WENDEL 30251 GCTAGGGGTTATAGTCGACGTCGATTCATCATTTTTAACGTCTCTAATTC 30300

Lser_US96UC23 30251 GCTAGGGGTTATAGTCGACGTCGATTCATCATTTTTAACGTCTCTAATTC 30300

Lser_LAC005780 30251 GCTAGGGGTTATAGTCGACGTCGATTCATCATTTTTAACGTCTCTAATTC 30300

Lvir_CGN013357 30249 GCTAGGGGTTATAGTCGACGTCGATTCATCATTTTTAACGTCTCTAATTC 30298

Lsal_LAC008020 30248 GCTAGGGGTTATAGTCGACGTCGAGTCATCATTTTTAACGTCTCTAATTC 30297

Lsat_Salinas 30301 AAAACCGAACATGAAACTTTGGTTTCATTCGGCTCCTTTATGGAAAATGG 30350

Lsat_WENDEL 30301 AAAACCGAACATGAAACTTTGGTTTCATTCGGCTCCTTTATGGAAAATGG 30350

Lser_US96UC23 30301 AAAACCGAACATGAAACTTTGGTTTCATTCGGCTCCTTTATGGAAAATGG 30350

Lser_LAC005780 30301 AAAACCGAACATGAAACTTTGGTTTCATTCGGCTCCTTTATGGAAAATGG 30350

Lvir_CGN013357 30299 AAAACCGAACATGAAACTTTGGTTTCATTCGGCTCCTTTATGGAAAATGG 30348

Lsal_LAC008020 30298 AAAACCGAACATGAAACTTTGGTTTCATTCGGCTCCTTTATGGAAAATGG 30347

Lsat_Salinas 30351 ATAAATTGCTAGATTTCAGATGTGAACCAACTTACAAAAAAAAAATGATA 30400

Lsat_WENDEL 30351 ATAAATTGCTAGATTTCAGATGTGAACCAACTTACAAAAAAAAAATGATA 30400

Lser_US96UC23 30351 ATAAATTGCTAGATTTCAGATGTGAACCAACTTACAAAAAAAAAATGATA 30400

Lser_LAC005780 30351 ATAAATTGCTAGATTTCAGATGTGAACCAACTTACAAAAAAAAAATGATA 30400

Lvir_CGN013357 30349 ATAAATTGCTAGATTTCAGATGTGAACCAACTTACAAAAAAAAAATGATA 30398

Lsal_LAC008020 30348 ATAAATTGCTAGATTTCAGATGTGAACCAACTTAAAAAAAAAAAATGATA 30397

Lsat_Salinas 30401 CCCATAACATCTATGTCAGCTTTTTGTTTGAATATATTCAATTCAAAACG 30450

Lsat_WENDEL 30401 CCCATAACATCTATGTCAGCTTTTTGTTTGAATATATTCAATTCAAAACG 30450

Lser_US96UC23 30401 CCCATAACATCTATGTCAGCTTTTTGTTTGAATATATTCAATTCAAAACG 30450

Lser_LAC005780 30401 CCCATAACATCTATGTCAGCTTTTTGTTTGAATATATTCAATTCAAAACG 30450

Lvir_CGN013357 30399 CCCATAACATCTATGTCAGCTTTTTGTTTGAATATATTCAATTCAAAACG 30448

Lsal_LAC008020 30398 CCCATAACATCTATGTCAGCTTTTTGTTTGAATATATTTAATTCAAAACG 30447

Lsat_Salinas 30451 ACTCGCTTTATAGATGATCCCTCTAGAAGAAGGCAATTCTAACAATCTTT 30500

Lsat_WENDEL 30451 ACTCGCTTTATAGATGATCCCTCTAGAAGAAGGCAATTCTAACAATCTTT 30500

Lser_US96UC23 30451 ACTCGCTTTATAGATGATCCCTCTAGAAGAAGGCAATTCTAACAATCTTT 30500

Lser_LAC005780 30451 ACTCGCTTTATAGATGATCCCTCTAGAAGAAGGCAATTCTAACAATCTTT 30500

Lvir_CGN013357 30449 ACTCGCTTTATAGATGATCCCTCTAGAAGAAGGCAATTCTAACAATCTTT 30498

Lsal_LAC008020 30448 ACTCGCTTTATAGATGATCCCTCTAGAAGAAGGCAATTCTAACAATCTTT 30497

Lsat_Salinas 30501 CTAGTTACTTCGTTCTTTATTTCTATTTGAGAGGGTCCTAAGGAAAAGGA 30550

Lsat_WENDEL 30501 CTAGTTACTTCGTTCTTTATTTCTATTTGAGAGGGTCCTAAGGAAAAGGA 30550

Lser_US96UC23 30501 CTAGTTACTTCGTTCTTTATTTCTATTTGAGAGGGTCCTAAGGAAAAGGA 30550

Lser_LAC005780 30501 CTAGTTACTTCGTTCTTTATTTCTATTTGAGAGGGTCCTAAGGAAAAGGA 30550

Lvir_CGN013357 30499 CTAGTTACTTCGTTCTTTATTTCTATTTGAGAGGGTCCTAAGGAAAAGGA 30548

Lsal_LAC008020 30498 CTAGTTACTTCGTTCTTTATTTCTATTTGAGAGGGTCCTAAGGAAAAGGA 30547

Lsat_Salinas 30551 TTTTATTTCCACCGAGCTAAAATAATATGTCGATGTCTCTAGTAAACTAA 30600

Lsat_WENDEL 30551 TTTTATTTCCACCGAGCTAAAATAATATGTCGATGTCTCTAGTAAACTAA 30600

Lser_US96UC23 30551 TTTTATTTCCACCGAGCTAAAATAATATGTCGATGTCTCTAGTAAACTAA 30600

Lser_LAC005780 30551 TTTTATTTCCACCGAGCTAAAATAATATGTCGATGTCTCTAGTAAACTAA 30600

Lvir_CGN013357 30549 TTTTATTTCCACCGAGCTAAAATAATATGTCGATGTCTCTAGTAAACTAA 30598

Lsal_LAC008020 30548 TTTTATTTCCACCGAGCTAAAATAATATGTCGATGTCTCTAGTAAACTAA 30597

Lsat_Salinas 30601 AGATATTCTTTAATAGCTATTTTGCTTCAATTTATTCGCTACAAATGCAA 30650

Lsat_WENDEL 30601 AGATATTCTTTAATAGCTATTTTGCTTCAATTTATTCGCTACAAATGCAA 30650

Lser_US96UC23 30601 AGATATTCTTTAATAGCTATTTTGCTTCAATTTATTCGCTACAAATGCAA 30650

Lser_LAC005780 30601 AGATATTCTTTAATAGCTATTTTGCTTCAATTTATTCGCTACAAATGCAA 30650

Lvir_CGN013357 30599 AGATATTCTTTAATAGCTATTTTGCTTCAATTTATTCGCTACAAATGCAA 30648

Lsal_LAC008020 30598 AGATATTCTTTAATAGCTATTTTGCTTCAATTTATTCGCTACAAATGCAA 30647

Lsat_Salinas 30651 ACAAAAATTGAAGATTTAGTTACGATTAGAAATTAGAAATCTACTTTTCT 30700

Lsat_WENDEL 30651 ACAAAAATTGAAGATTTAGTTACGATTAGAAATTAGAAATCTACTTTTCT 30700

Lser_US96UC23 30651 ACAAAAATTGAAGATTTAGTTACGATTAGAAATTAGAAATCTACTTTTCT 30700

Lser_LAC005780 30651 ACAAAAATTGAAGATTTAGTTACGATTAGAAATTAGAAATCTACTTTTCT 30700

Lvir_CGN013357 30649 ACAAAAATTGAAGATTTAGTTACGATTAGAAATTAGAAATCTACTTTTCT 30698

Lsal_LAC008020 30648 ACAAAAATTGAAGATTTAGTTACGATTAGAAATTAGAAATCTACTTTTCT 30697

Lsat_Salinas 30701 ATCTTCATCCATGGATTCTTTATTCATACTCATTCAATTGGAAGTATTGA 30750

Lsat_WENDEL 30701 ATCTTCATCCATGGATTCTTTATTCATACTCATTCAATTGGAAGTATTGA 30750

Lser_US96UC23 30701 ATCTTCATCCATGGATTCTTTATTCATACTCATTCAATTGGAAGTATTGA 30750

Lser_LAC005780 30701 ATCTTCATCCATGGATTCTTTATTCATACTCATTCAATTGGAAGTATTGA 30750

Lvir_CGN013357 30699 ATCTTCATCCATGGATTCTTTATTCATACTCATTCAATTGGAAGTATTGA 30748

Lsal_LAC008020 30698 ATCTTCATCCATGGATTCTTTATTCATACTCATTCAATTGGAAGTATTGA 30747

Lsat_Salinas 30751 TCCAATTTTAAAATTTTGTTTCGCAATTTTATAATCCAAGTTTTCATTTT 30800

Lsat_WENDEL 30751 TCCAATTTTAAAATTTTGTTTCGCAATTTTATAATCCAAGTTTTCATTTT 30800

Lser_US96UC23 30751 TCCAATTTTAAAATTTTGTTTCGCAATTTTATAATCCAAGTTTTCATTTT 30800

Lser_LAC005780 30751 TCCAATTTTAAAATTTTGTTTCGCAATTTTATAATCCAAGTTTTCATTTT 30800

Lvir_CGN013357 30749 TCCAATTTTAAAATTTTGTTTCGCAATTTTATAATCCAAGTTTTCATTTT 30798

Lsal_LAC008020 30748 TCCAATTTTCAAATTTTGTTTCGCAATTTTATAATCCAAGTTTTCATTTT 30797

Lsat_Salinas 30801 TTAATTGACCTTTGGATAAAAATCCCGAAAATTTATATTTTTCCTTGAAT 30850

Lsat_WENDEL 30801 TTAATTGACCTTTGGATAAAAATCCCGAAAATTTATATTTTTCCTTGAAT 30850

Lser_US96UC23 30801 TTAATTGACCTTTGGATAAAAATCCCGAAAATTTATATTTTTCCTTGAAT 30850

Lser_LAC005780 30801 TTAATTGACCTTTGGATAAAAATCCCGAAAATTTATATTTTTCCTTGAAT 30850

Lvir_CGN013357 30799 TTAATTGACCTTTGGATAAAAATCCCGAAAATTTATATTTTTCCTTGAAT 30848

Lsal_LAC008020 30798 TTAATTGACCTTTGGATAAAAATCCCGAAAATTTATATTTTTCCTTGAAT 30847

Lsat_Salinas 30851 GTGTCATTGAAAGGTAAAGGATTGAATCCTTTTAATGAAATAAAGTTTTT 30900

Lsat_WENDEL 30851 GTGTCATTGAAAGGTAAAGGATTGAATCCTTTTAATGAAATAAAGTTTTT 30900

Lser_US96UC23 30851 GTGTCATTGAAAGGTAAAGGATTGAATCCTTTTAATGAAATAAAGTTTTT 30900

Lser_LAC005780 30851 GTGTCATTGAAAGGTAAAGGATTGAATCCTTTTAATGAAATAAAGTTTTT 30900

Lvir_CGN013357 30849 GTGTCATTGAAAGGTAAAGGATTGAATCCTTTTAATGAAATAAAGTTTTT 30898

Lsal_LAC008020 30848 GTGTCATTGAAAGGTAAAGGATTGAATCCTTTTAATGAAATAAAGTTTTT 30897

Lsat_Salinas 30901 GATCGGAATATGAATCAAACCGAAAGACCCTTTAACTATTAAGGGGGTTA 30950

Lsat_WENDEL 30901 GATCGGAATATGAATCAAACCGAAAGACCCTTTAACTATTAAGGGGGTTA 30950

Lser_US96UC23 30901 GATCGGAATATGAATCAAACCGAAAGACCCTTTAACTATTAAGGGGGTTA 30950

Lser_LAC005780 30901 GATCGGAATATGAATCAAACCGAAAGACCCTTTAACTATTAAGGGGGTTA 30950

Lvir_CGN013357 30899 GATCGGAATATGAATCAAACCGAAAGACCCTTTAACTATTAAGGGGGTTA 30948

Lsal_LAC008020 30898 GATCGGAATATGAATCAAACCGAAAGACCCTTTAACTATTAAGGGGGTTA 30947

Lsat_Salinas 30951 ATAGAACGAATCACACTTTTACCACTAAACTATACCCGCTACAGTGCAAA 31000

Lsat_WENDEL 30951 ATAGAACGAATCACACTTTTACCACTAAACTATACCCGCTACAGTGCAAA 31000

Lser_US96UC23 30951 ATAGAACGAATCACACTTTTACCACTAAACTATACCCGCTACAGTGCAAA 31000

Lser_LAC005780 30951 ATAGAACGAATCACACTTTTACCACTAAACTATACCCGCTACAGTGCAAA 31000

Lvir_CGN013357 30949 ATAGAACGAATCACACTTTTACCACTAAACTATACCCGCTACAGTGCAAA 30998

Lsal_LAC008020 30948 ATAGAACGAATCACACTTTTACCACTAAACTATACCCGCTACAGTGCAAA 30997

Lsat_Salinas 31001 TTATTATATATAAATGGACCTTTTGTCGAGGCGCTTCTGTCTATGTGTAG 31050

Lsat_WENDEL 31001 TTATTATATATAAATGGACCTTTTGTCGAGGCGCTTCTGTCTATGTGTAG 31050

Lser_US96UC23 31001 TTATTATATATAAATGGACCTTTTGTCGAGGCGCTTCTGTCTATGTGTAG 31050

Lser_LAC005780 31001 TTATTATATATAAATGGACCTTTTGTCGAGGCGCTTCTGTCTATGTGTAG 31050

Lvir_CGN013357 30999 TTATTATATATAAATGGACCTTTTGTCGAGGCGCTTCTGTCTATGTGTAG 31048

Lsal_LAC008020 30998 TTATTATATATAAATGGACCTTTTGTCGAGGCGCTTCTGTCTATGTGTAG 31047

Lsat_Salinas 31051 ATGGACTTCTTATACTTATATAAGAAGTAGGTATATATCTTGTTATATAG 31100

Lsat_WENDEL 31051 ATGGACTTCTTATACTTATATAAGAAGTAGGTATATATCTTGTTATATAG 31100

Lser_US96UC23 31051 ATGGACTTCTTATACTTATATAAGAAGTAGGTATATATCTTGTTATATAG 31100

Lser_LAC005780 31051 ATGGACTTCTTATACTTATATAAGAAGTAGGTATATATCTTGTTATATAG 31100

Lvir_CGN013357 31049 ATGGACTTCTTATACTTATATAAGAAGTAGGTATATATCTTGTTATATAG 31098

Lsal_LAC008020 31048 ATGGACTTCTTATACTTATATAAGAAGTAGGTATATATCYTGTTATATAG 31097

Lsat_Salinas 31101 ATATATTTTACCTAGATAGTAAAATGACCCGTTTAGGAATTCCATGAACC 31150

Lsat_WENDEL 31101 ATATATTTTACCTAGATAGTAAAATGACCCGTTTAGGAATTCCATGAACC 31150

Lser_US96UC23 31101 ATATATTTTACCTAGATAGTAAAATGACCCGTTTAGGAATTCCATGAACC 31150

Lser_LAC005780 31101 ATATATTTTACCTAGATAGTAAAATGACCCGTTTAGGAATTCCATGAACC 31150

Lvir_CGN013357 31099 ATATATTTTACCTAGATAGTAAAGTGACCCGTTTAGGAATTCCATGAACC 31148

Lsal_LAC008020 31098 ATATATTTTACCTAGATAGTAAAGTGACCCGTTTAGGAATTCCATGAACC 31147

Lsat_Salinas 31151 AAGCCCTTTTCTTTTAACTCAGTGGTAGAGTAATGACATGGTAAGGCGTA 31200

Lsat_WENDEL 31151 AAGCCCTTTTCTTTTAACTCAGTGGTAGAGTAATGACATGGTAAGGCGTA 31200

Lser_US96UC23 31151 AAGCCCTTTTCTTTTAACTCAGTGGTAGAGTAATGACATGGTAAGGCGTA 31200

Lser_LAC005780 31151 AAGCCCTTTTCTTTTAACTCAGTGGTAGAGTAATGACATGGTAAGGCGTA 31200

Lvir_CGN013357 31149 AAGCCCTTTTCTTTTAACTCAGTGGTAGAGTAATGACATGGTAAGGCGTA 31198

Lsal_LAC008020 31148 AAGCCCTTTTCTTTTAACTCAGTGGTAGAGTAATGACATGGTAAGGCGTA 31197

Lsat_Salinas 31201 AGCCCTCGGTTCAAATCCGATAAGGGGCTTTGTATTTTTTTCAGTCATAG 31250

Lsat_WENDEL 31201 AGCCCTCGGTTCAAATCCGATAAGGGGCTTTGTATTTTTTTCAGTCATAG 31250

Lser_US96UC23 31201 AGCCCTCGGTTCAAATCCGATAAGGGGCTTTGTATTTTTTTCAGTCATAG 31250

Lser_LAC005780 31201 AGCCCTCGGTTCAAATCCGATAAGGGGCTTTGTATTTTTTTCAGTCATAG 31250

Lvir_CGN013357 31199 AGCCCTCGGTTCAAATCCGATAAGGGGCTTTGTATTTTTTTCAGTCATAG 31248

Lsal_LAC008020 31198 AGCCCTCGGTTCAAATCCGATAAGGGGCTTTGTATTTTTTTCAGTCATAG 31247

Lsat_Salinas 31251 TATTCATATTTGAAAGAAGAATAGCGATATTATTAATAGTAATAAAAAAA 31300

Lsat_WENDEL 31251 TATTCATATTTGAAAGAAGAATAGCGATATTATTAATAGTAATAAAAAAA 31300

Lser_US96UC23 31251 TATTCATATTTGAAAGAAGAATAGCGATATTATTAATAGTAATAAAAAAA 31300

Lser_LAC005780 31251 TATTCATATTTGAAAGAAGAATAGCGATATTATTAATAGTAATAAAAAAA 31300

Lvir_CGN013357 31249 TATTCATATTTGAAAGAAGAATAGCGATATTATTAATAGTAATAAAAAAA 31298

Lsal_LAC008020 31248 TATTCATATTTGAAAGAAGAATAGCGATATTATTAATAGTAATAAAAAAA 31297

Lsat_Salinas 31301 AAATAGTAATAAAAAAGTAACCAACTGTATAATATTATCGTCTAATGAGT 31350

Lsat_WENDEL 31301 AAATAGTAATAAAAAAGTAACCAACTGTATAATATTATCGTCTAATGAGT 31350

Lser_US96UC23 31301 AAATAGTAATAAAAAAGTAACCAACTGTATAATATTATCGTCTAATGAGT 31350

Lser_LAC005780 31301 AAATAGTAATAAAAAAGTAACCAACTGTATAATATTATCGTCTAATGAGT 31350

Lvir_CGN013357 31299 AAATAGTAATAAAAAAGTAACCAACTGTATAATATTATCGTCTAATGAGT 31348

Lsal_LAC008020 31298 AA-TAGTAATAAAAAAGTAACCAACTGTATAATATTATCGTCTAATGAGT 31346

Lsat_Salinas 31351 TATAGTATATTAGTTGTTAGAGTATATTAGTTAGAGTTATCACGAATAAT 31400

Lsat_WENDEL 31351 TATAGTATATTAGTTGTTAGAGTATATTAGTTAGAGTTATCACGAATAAT 31400

Lser_US96UC23 31351 TATAGTATATTAGTTGTTAGAGTATATTAGTTAGAGTTATCACGAATAAT 31400

Lser_LAC005780 31351 TATAGTATATTAGTTGTTAGAGTATATTAGTTATAGTTATCACGAATAAT 31400

Lvir_CGN013357 31349 TATAGTATATTAGTTGTTAGAGTATATTAGTTATAGTTATCACGAATAAT 31398

Lsal_LAC008020 31347 TATAGTATATTAGTTGTTAGAGTATATTAGTTATAGTTATCACGAATAAT 31396

Lsat_Salinas 31401 GATAAGTCATCTGTTGAATCATCAAATCTTCCTATTTTCAATTAGGCCAA 31450

Lsat_WENDEL 31401 GATAAGTCATCTGTTGAATCATCAAATCTTCCTATTTTCAATTAGGCCAA 31450

Lser_US96UC23 31401 GATAAGTCATCTGTTGAATCATCAAATCTTCCTATTTTCAATTAGGCCAA 31450

Lser_LAC005780 31401 GATAAGTCATCTGTTGAATCATCAAATCTTCCTATTTTCAATTAGGCCAA 31450

Lvir_CGN013357 31399 GATAAGTCATCTGTTGAATCATCAAATCTTCCTATTTTCAATTAGGCCAA 31448

Lsal_LAC008020 31397 GATAAGTCATCTGTTGAATCATCAAATCTTCCTATTTTCAATTAGGCCAA 31446

Lsat_Salinas 31451 TGAAATCCATTGTAAAGATTATAGATCAATCAAAGAAAAATAAGTGGACC 31500

Lsat_WENDEL 31451 TGAAATCCATTGTAAAGATTATAGATCAATCAAAGAAAAATAAGTGGACC 31500

Lser_US96UC23 31451 TGAAATCCATTGTAAAGATTATAGATCAATCAAAGAAAAATAAGTGGACC 31500

Lser_LAC005780 31451 TGAAATCCATTGTAAAGATTATAGATCAATCAAAGAAAAATAAGTGGACC 31500

Lvir_CGN013357 31449 TGAAATCCATTGTAAAGATTATAGATCAATCAAAGAAAAATAAGTGGACC 31498

Lsal_LAC008020 31447 TGAAATCCATTGTAAAGATTATAGATCAATCAAAGAAAAATAAGTGGACC 31496

Lsat_Salinas 31501 TGACCCATTGAATCATTACTATATCCGCTATTCTGATATTCAAATTCAAT 31550

Lsat_WENDEL 31501 TGACCCATTGAATCATTACTATATCCGCTATTCTGATATTCAAATTCAAT 31550

Lser_US96UC23 31501 TGACCCATTGAATCATTACTATATCCGCTATTCTGATATTCAAATTCAAT 31550

Lser_LAC005780 31501 TGACCCATTGAATCATTACTATATCCGCTATTCTGATATTCAAATTCAAT 31550

Lvir_CGN013357 31499 TGACCCATTGAATCATTACTATATCCGCTATTCTGATATTCAAATTCAAT 31548

Lsal_LAC008020 31497 TGACCCATTGAATCATTACTATATCCGCTATTCTGATATTCAAATTCAAT 31546

Lsat_Salinas 31551 AGAGATGAAATTGGAACAAGTTGACCTTTTATTTATTTTCATTTCTTTGG 31600

Lsat_WENDEL 31551 AGAGATGAAATTGGAACAAGTTGACCTTTTATTTATTTTCATTTCTTTGG 31600

Lser_US96UC23 31551 AGAGATGAAATTGGAACAAGTTGACCTTTTATTTATTTTCATTTCTTTGG 31600

Lser_LAC005780 31551 AGAGATGAAATTGGAACAAGTTGACCTTTTATTTATTTTCATTTCTTTGG 31600

Lvir_CGN013357 31549 AGAGATGAAATTGGAACAAGTTGACCTTTTATTTATTTTCATTTCTTTGG 31598

Lsal_LAC008020 31547 AGAGATGAAATTGGAACAAGTTGACCTTTTATTTATTTTCATTTCTTTGG 31596

Lsat_Salinas 31601 ACTCCGCAAGAATTTGTCGATATTTCCAATTCAATCGTCTTGTTCCTAGA 31650

Lsat_WENDEL 31601 ACTCCGCAAGAATTTGTCGATATTTCCAATTCAATCGTCTTGTTCCTAGA 31650

Lser_US96UC23 31601 ACTCCGCAAGAATTTGTCGATATTTCCAATTCAATCGTCTTGTTCCTAGA 31650

Lser_LAC005780 31601 ACTCCGCAAGAATTTGTCGATATTTCCAATTCAATCGTCTTGTTCCTAGA 31650

Lvir_CGN013357 31599 ACTCCGCAAGAATTTGTCGATATTTCCAATTCAATCGTCTTGTTCCTAGA 31648

Lsal_LAC008020 31597 ACTCCGCAAGAATTTGTCGATATTTCCAATTCAATCGTCTTGTTCCTAGA 31646

Lsat_Salinas 31651 TGTTCTATAGGAATAAATTGTCATTTGGTTCCTTCATGGAGAACCTTTTA 31700

Lsat_WENDEL 31651 TGTTCTATAGGAATAAATTGTCATTTGGTTCCTTCATGGAGAACCTTTTA 31700

Lser_US96UC23 31651 TGTTCTATAGGAATAAATTGTCATTTGGTTCCTTCATGGAGAACCTTTTA 31700

Lser_LAC005780 31651 TGTTCTATAGGAATAAATTGTCATTTGGTTCCTTCATGGAGAACCTTTTA 31700

Lvir_CGN013357 31649 TGTTCTATAGGAATAAATTGTCATTTGGTTCCTTCATGGAGAACCTTTTA 31698

Lsal_LAC008020 31647 TGTTCTATAGGAATAAATTGTCATTTGGTTCCTTCATGGAGAACCTTTTA 31696

Lsat_Salinas 31701 TTCTAAGTCAAAAGATAAGAAAAATTTTCACTATCTTTCTTTGATTACAG 31750

Lsat_WENDEL 31701 TTCTAAGTCAAAAGATAAGAAAAATTTTCACTATCTTTCTTTGATTACAG 31750

Lser_US96UC23 31701 TTCTAAGTCAAAAGATAAGAAAAATTTTCACTATCTTTCTTTGATTACAG 31750

Lser_LAC005780 31701 TTCTAAGTCAAAAGATAAGAAAAATTTTCACTATCTTTCTTTGATTACAG 31750

Lvir_CGN013357 31699 TTCTAAGTCAAAAGATAAGAAAAATTTTCACTATCTTTCTTTGATTACAG 31748

Lsal_LAC008020 31697 TTCTAAGTCAAAAGATAAGAAAAATTTTCACTAGCTTTCTTTGATTACAG 31746

Lsat_Salinas 31751 GATCAAGATTAATTTATATCTATATAATATTTCTATATTTAGATTTATAG 31800

Lsat_WENDEL 31751 GATCAAGATTAATTTATATCTATATAATATTTCTATATTTAGATTTATAG 31800

Lser_US96UC23 31751 GATCAAGATTAATTTATATCTATATAATATTTCTATATTTAGATTTATAG 31800

Lser_LAC005780 31751 GATCAAGATTAATTTATATCTATATAATATTTCTATATTTAGATTTATAG 31800

Lvir_CGN013357 31749 GATCAAGATTAATTTATATCTATATAATATTTCTATATTTAGATTTATAG 31798

Lsal_LAC008020 31747 GATCAAGATTAATTTATATCTATATAATATTTCTATATTTAGATTTATAG 31796

Lsat_Salinas 31801 CTATGAGATCGCGGCTTGATAAAAATTTCCATTTCGTTGCATCCAAGATT 31850

Lsat_WENDEL 31801 CTATGAGATCGCGGCTTGATAAAAATTTCCATTTCGTTGCATCCAAGATT 31850

Lser_US96UC23 31801 CTATGAGATCGCGGCTTGATAAAAATTTCCATTTCGTTGCATCCAAGATT 31850

Lser_LAC005780 31801 CTATGAGATCGCGGCTTGATAAAAATTTCCATTTCGTTGCATCCAAGATT 31850

Lvir_CGN013357 31799 CTATGAGATCGCGGCTTGATAAAAATTTCCATTTCGTTGCATCCAAGATT 31848

Lsal_LAC008020 31797 CTATGAGATCGCGGCTTGATAAAAATTTCCATTTCGTTGCATCCAAGATT 31846

Lsat_Salinas 31851 TTTGTTCTGACAATCGTATGAAGAATGGATGCGATAAAAAGACTCTCATT 31900

Lsat_WENDEL 31851 TTTGTTCTGACAATCGTATGAAGAATGGATGCGATAAAAAGACTCTCATT 31900

Lser_US96UC23 31851 TTTGTTCTGACAATCGTATGAAGAATGGATGCGATAAAAAGACTCTCATT 31900

Lser_LAC005780 31851 TTTGTTCTGACAATCGTATGAAGAATGGATGCGAGAAAAAGACTCTCATT 31900

Lvir_CGN013357 31849 TTTGTTCTGACAATCGTATGAAGAATGGATGCGAGAAAAAGACTCTCATT 31898

Lsal_LAC008020 31847 TTTGTTCTGACAATCGTATGAAGAATGGATGCGAGAAAAAGACTCTCATT 31896

Lsat_Salinas 31901 CCCAGTTTCCTATTTATTTTATTGAATATTGTTATTGTATTGAGTATTGA 31950

Lsat_WENDEL 31901 CCCAGTTTCCTATTTATTTTATTGAATATTGTTATTGTATTGAGTATTGA 31950

Lser_US96UC23 31901 CCCAGTTTCCTATTTATTTTATTGAATATTGTTATTGTATTGAGTATTGA 31950

Lser_LAC005780 31901 CCCAGTTTCCTATTTATTTTATTGAATATTGTTATTGTATTGAGTATTGA 31950

Lvir_CGN013357 31899 CCCAGTTTCCTATTTATTTTATTGAATATTGTTATTGTATTGAGTATTGA 31948

Lsal_LAC008020 31897 CCCAGTTTCCTATTTATTTTATTGAATATTGTTATTGTATTGAGTATTGA 31946

Lsat_Salinas 31951 AGTGAAGTAAAAAATTGGAAACTCTCTCTTTTCTAACAGAGAAAAAGAAA 32000

Lsat_WENDEL 31951 AGTGAAGTAAAAAATTGGAAACTCTCTCTTTTCTAACAGAGAAAAAGAAA 32000

Lser_US96UC23 31951 AGTGAAGTAAAAAATTGGAAACTCTCTCTTTTCTAACAGAGAAAAAGAAA 32000

Lser_LAC005780 31951 AGTGAAGTAAAAAATTGGAAACTCTCTCTTTTCTAACAGAGAAAAAGAAA 32000

Lvir_CGN013357 31949 AGTGAAGTAAAAAATTGGAAACTCTCTCTTTTCTAACAGAGAAAAAGAAA 31998

Lsal_LAC008020 31947 AGTGAAGTAAAAAATTGGAAACTCTCTCTTTTCTAACAGAGAAAAAGAAA 31996

Lsat_Salinas 32001 TAGAAAAAGATTAGTAATTTACTATACATAACATAAAAATTAGAAGAATT 32050

Lsat_WENDEL 32001 TAGAAAAAGATTAGTAATTTACTATACATAACATAAAAATTAGAAGAATT 32050

Lser_US96UC23 32001 TAGAAAAAGATTAGTAATTTACTATACATAACATAAAAATTAGAAGAATT 32050

Lser_LAC005780 32001 TAGAAAAAGATTAGTAATTTACTATACATAACATAAAAATTAGAAGAATT 32050

Lvir_CGN013357 31999 TAGAAAAAGATTAGTAATTTACTATACATAACATAAAAATTAGAAGAATT 32048

Lsal_LAC008020 31997 TAGAAAAAGATTAGTAATTTACTATACATAACATAAAAATTAGAAGAATT 32046

Lsat_Salinas 32051 TAGAAAGAGAGTTCTTTTTCTTAATCTCATGAACAAGATCTAAGAATCCA 32100

Lsat_WENDEL 32051 TAGAAAGAGAGTTCTTTTTCTTAATCTCATGAACAAGATCTAAGAATCCA 32100

Lser_US96UC23 32051 TAGAAAGAGAGTTCTTTTTCTTAATCTCATGAACAAGATCTAAGAATCCA 32100

Lser_LAC005780 32051 TAGAAAGAGAGTTCTTTTTCTTAATCTCATGAACAAGATCTAAGAATCCA 32100

Lvir_CGN013357 32049 TAGAAAGAGAGTTCTTTTTCTTAATCTCATGAACAAGATCTAAGAATCCA 32098

Lsal_LAC008020 32047 TAGAAAGATAGTTCTTTTTCTTAATCTCATGAACAAGATCTAAGAATCCA 32096

Lsat_Salinas 32101 TTTAGTTGATGGAAGAAGAGGGGGGGGGGCAGGCCTGAGGATCAACCGGT 32150

Lsat_WENDEL 32101 TTTAGTTGATGGAAGAAGAGGGGGGGGGGCAGGCCTGAGGATCAACCGGT 32150

Lser_US96UC23 32101 TTTAGTTGATGGAAGAAGAGGGGGGGGGGCAGGCCTGAGGATCAACCGGT 32150

Lser_LAC005780 32101 TTTAGTTGATGGAAGAAGAGGGGGGGGGGCAGGCCTGAGGATCAACCGGT 32150

Lvir_CGN013357 32099 TTTAGTTGATGGAAGAAGAGGGGGGGGGGCAGGCCTGAGGATCAACCGGT 32148

Lsal_LAC008020 32097 TTTAGTTGGTGGAAGAAGAGGGGGGGGGGCAGGCCTGAGGATCAACCGGT 32146

Lsat_Salinas 32151 AGTGGGCGAGGGGGTTGCTTTTTCCTTGAACAGTTCTTTCAAAAAACGAA 32200

Lsat_WENDEL 32151 AGTGGGCGAGGGGGTTGCTTTTTCCTTGAACAGTTCTTTCAAAAAACGAA 32200

Lser_US96UC23 32151 AGTGGGCGAGGGGGTTGCTTTTTCCTTGAACAGTTCTTTCAAAAAACGAA 32200

Lser_LAC005780 32151 AGTGGGCGAGGGGGTTGCTTTTTCCTTGAACAGTTCTTTCAAAAAACGAA 32200

Lvir_CGN013357 32149 AGTGGGCGAGGGGGTTGCTTTTTCCTTGAACAGTTCTTTCAAAAAACGAA 32198

Lsal_LAC008020 32147 AGTGGGCGAGGGGGTTGCTTTTTCCTTGAACAGTTCTTTCAAAAAACGAA 32196

Lsat_Salinas 32201 TCTATCTGATTGATGAGTCATAAGAAGACAATTCATGATTCAGATGCTTA 32250

Lsat_WENDEL 32201 TCTATCTGATTGATGAGTCATAAGAAGACAATTCATGATTCAGATGCTTA 32250

Lser_US96UC23 32201 TCTATCTGATTGATGAGTCATAAGAAGACAATTCATGATTCAGATGCTTA 32250

Lser_LAC005780 32201 TCTATCTGATTGATGAGTCATAAGAAGACAATTCATGATTCAGATGCTTA 32250

Lvir_CGN013357 32199 TCTATCTGATTGATGAGTCATAAGAAGACAATTCATGATTCAGATGCTTA 32248

Lsal_LAC008020 32197 TCTATCTGATTGATGAGTCATAAGAAGACAATTCATGATTCAGATGCTTA 32246

Lsat_Salinas 32251 ATAATAAGAAGGAATAATCAAATTGAATTCATCGATTTACCTGGGTGGTC 32300

Lsat_WENDEL 32251 ATAATAAGAAGGAATAATCAAATTGAATTCATCGATTTACCTGGGTGGTC 32300

Lser_US96UC23 32251 ATAATAAGAAGGAATAATCAAATTGAATTCATCGATTTACCTGGGTGGTC 32300

Lser_LAC005780 32251 ATAATAAGAAGGAATAATCAAATTGAATTCATCGATTTACCTGGGTGGTC 32300

Lvir_CGN013357 32249 ATAATAAGAAGGAATAATCAAATTGAATTCATCGATTTACCTGGGTGGTC 32298

Lsal_LAC008020 32247 ATAATAAGAAGGAATAATCAAATTGAATTCATCGATTTACCTGGGTGGTC 32296

Lsat_Salinas 32301 AATTTATGGGCCAATAAAGGATTTTTATCTTCGAAACCCATTGGAAGGGG 32350

Lsat_WENDEL 32301 AATTTATGGGCCAATAAAGGATTTTTATCTTCGAAACCCATTGGAAGGGG 32350

Lser_US96UC23 32301 AATTTATGGGCCAATAAAGGATTTTTATCTTCGAAACCCATTGGAAGGGG 32350

Lser_LAC005780 32301 AATTTATGGGCCAATAAAGGATTTTTATCTTCGAAACCCATTGGAAGGGG 32350

Lvir_CGN013357 32299 AATTTATGGGCCAATAAAGGATTTTTATCTTCGAAACCCATTGGAAGGGG 32348

Lsal_LAC008020 32297 AATTTATGGGCCAATAAAGGATTTTTATCTTCGAAACCCATTGGAAGGGG 32346

Lsat_Salinas 32351 TAGTGCACGAGAAAAAAATCATGCAGAAATGATCGACTCTTTGGACGCCC 32400

Lsat_WENDEL 32351 TAGTGCACGAGAAAAAAATCATGCAGAAATGATCGACTCTTTGGACGCCC 32400

Lser_US96UC23 32351 TAGTGCACGAGAAAAAAATCATGCAGAAATGATCGACTCTTTGGACGCCC 32400

Lser_LAC005780 32351 TAGTGCACGAGAAAAAAATCATGCAGAAATGATCGACTCTTTGGACGCCC 32400

Lvir_CGN013357 32349 TAGTGCACGAGAAAAAAATCATGCAGAAATGATCGACTCTTTGGACGCCC 32398

Lsal_LAC008020 32347 TAGTGCACGAGAAAAAAATCATGCAGAAATGATCGACTCTTTGGACGCCC 32396

Lsat_Salinas 32401 CGAAAATACTATGAGGTGTTCGGAAATGGTCGAAGTAGTTGAATAGGAGG 32450

Lsat_WENDEL 32401 CGAAAATACTATGAGGTGTTCGGAAATGGTCGAAGTAGTTGAATAGGAGG 32450

Lser_US96UC23 32401 CGAAAATACTATGAGGTGTTCGGAAATGGTCGAAGTAGTTGAATAGGAGG 32450

Lser_LAC005780 32401 CGAAAATACTATGAGGTGTTCGGAAATGGTCGAAGTAGTTGAATAGGAGG 32450

Lvir_CGN013357 32399 CGAAAATACTATGAGGTGTTCGGAAATGGTCGAAGTAGTTGAATAGGAGG 32448

Lsal_LAC008020 32397 CGAAAATACTATGAGGTGTTCGGAAATGGTCGAAGTAGTTGAATAGGAGG 32446

Lsat_Salinas 32451 ATTACGATGACTATAGCCCTTGGTAAAGTTACCAAAGACGAAAATGATTT 32500

Lsat_WENDEL 32451 ATTACGATGACTATAGCCCTTGGTAAAGTTACCAAAGACGAAAATGATTT 32500

Lser_US96UC23 32451 ATTACGATGACTATAGCCCTTGGTAAAGTTACCAAAGACGAAAATGATTT 32500

Lser_LAC005780 32451 ATTACGATGACTATAGCCCTTGGTAAAGTTACCAAAGACGAAAATGATTT 32500

Lvir_CGN013357 32449 ATTACGATGACTATAGCCCTTGGTAAAGTTACCAAAGACGAAAATGATTT 32498

Lsal_LAC008020 32447 ATTACGATGACTATAGCCCTTGGTAAAGTTACCAAAGACGAAAATGATTT 32496

Lsat_Salinas 32501 GTTTGATATTATGGATGACTGGTTACGGAGGGACCGTTTCGTTTTTGTAG 32550

Lsat_WENDEL 32501 GTTTGATATTATGGATGACTGGTTACGGAGGGACCGTTTCGTTTTTGTAG 32550

Lser_US96UC23 32501 GTTTGATATTATGGATGACTGGTTACGGAGGGACCGTTTCGTTTTTGTAG 32550

Lser_LAC005780 32501 GTTTGATATTATGGATGACTGGTTACGGAGGGACCGTTTCGTTTTTGTAG 32550

Lvir_CGN013357 32499 GTTTGATATTATGGATGACTGGTTACGGAGGGACCGTTTCGTTTTTGTAG 32548

Lsal_LAC008020 32497 GTTTGATATTATGGATGACTGGTTACGGAGGGACCGTTTCGTTTTTGTAG 32546

Lsat_Salinas 32551 GCTGGTCCGGCCTATTGCTCTTTCCTTGTGCCTATTTCGCTGTAGGGGGT 32600

Lsat_WENDEL 32551 GCTGGTCCGGCCTATTGCTCTTTCCTTGTGCCTATTTCGCTGTAGGGGGT 32600

Lser_US96UC23 32551 GCTGGTCCGGCCTATTGCTCTTTCCTTGTGCCTATTTCGCTGTAGGGGGT 32600

Lser_LAC005780 32551 GCTGGTCCGGCCTATTGCTCTTTCCTTGTGCCTATTTCGCTGTAGGGGGT 32600

Lvir_CGN013357 32549 GCTGGTCCGGCCTATTGCTCTTTCCTTGTGCCTATTTCGCTGTAGGGGGT 32598

Lsal_LAC008020 32547 GCTGGTCCGGCCTATTGCTCTTTCCTTGTGCCTATTTCGCTGTAGGGGGT 32596

Lsat_Salinas 32601 TGGTTCACAGGTACAACCTTTGTAACTTCATGGTATACCCATGGATTGGC 32650

Lsat_WENDEL 32601 TGGTTCACAGGTACAACCTTTGTAACTTCATGGTATACCCATGGATTGGC 32650

Lser_US96UC23 32601 TGGTTCACAGGTACAACCTTTGTAACTTCATGGTATACCCATGGATTGGC 32650

Lser_LAC005780 32601 TGGTTCACAGGTACAACCTTTGTAACTTCATGGTATACCCATGGATTGGC 32650

Lvir_CGN013357 32599 TGGTTCACGGGTACAACCTTTGTAACTTCATGGTATACCCATGGATTGGC 32648

Lsal_LAC008020 32597 TGGTTCACGGGTACAACCTTTGTAACTTCATGGTATACCCATGGATTGGC 32646

Lsat_Salinas 32651 CAGTTCCTATTTGGAAGGCTGCAATTTCTTAACTGCCGCAGTTTCTACTC 32700

Lsat_WENDEL 32651 CAGTTCCTATTTGGAAGGCTGCAATTTCTTAACTGCCGCAGTTTCTACTC 32700

Lser_US96UC23 32651 CAGTTCCTATTTGGAAGGCTGCAATTTCTTAACTGCCGCAGTTTCTACTC 32700

Lser_LAC005780 32651 CAGTTCCTATTTGGAAGGCTGCAATTTCTTAACTGCCGCAGTTTCTACTC 32700

Lvir_CGN013357 32649 CAGTTCCTATTTGGAAGGCTGCAATTTCTTAACTGCCGCAGTTTCTACTC 32698

Lsal_LAC008020 32647 CAGTTCCTATTTGGAAGGCTGCAATTTCTTAACTGCCGCAGTTTCTACTC 32696

Lsat_Salinas 32701 CTGCGAATAGTTTAGCACATTCTTTGTTATTACTATGGGGTCCTGAAGCA 32750

Lsat_WENDEL 32701 CTGCGAATAGTTTAGCACATTCTTTGTTATTACTATGGGGTCCTGAAGCA 32750

Lser_US96UC23 32701 CTGCGAATAGTTTAGCACATTCTTTGTTATTACTATGGGGTCCTGAAGCA 32750

Lser_LAC005780 32701 CTGCGAATAGTTTAGCACATTCTTTGTTATTACTATGGGGTCCTGAAGCA 32750

Lvir_CGN013357 32699 CTGCGAATAGTTTAGCACATTCTTTGTTATTACTATGGGGTCCTGAAGCA 32748

Lsal_LAC008020 32697 CTGCGAATAGTTTAGCACATTCTTTGTTATTACTATGGGGTCCTGAAGCA 32746

Lsat_Salinas 32751 CAAGGAGATTTTACTCGTTGGTGTCAATTAGGCGGTCTGTGGACTTTTGT 32800

Lsat_WENDEL 32751 CAAGGAGATTTTACTCGTTGGTGTCAATTAGGCGGTCTGTGGACTTTTGT 32800

Lser_US96UC23 32751 CAAGGAGATTTTACTCGTTGGTGTCAATTAGGCGGTCTGTGGACTTTTGT 32800

Lser_LAC005780 32751 CAAGGAGATTTTACTCGTTGGTGTCAATTAGGCGGTCTGTGGACTTTTGT 32800

Lvir_CGN013357 32749 CAAGGAGATTTTACTCGTTGGTGTCAATTAGGCGGTCTGTGGACTTTTGT 32798

Lsal_LAC008020 32747 CAAGGAGATTTTACTCGTTGGTGTCAATTAGGCGGTCTGTGGACTTTTGT 32796

Lsat_Salinas 32801 TGCTCTCCACGGCGCTTTCGGACTAATAGGTTTCATGTTGCGTCAATTCG 32850

Lsat_WENDEL 32801 TGCTCTCCACGGCGCTTTCGGACTAATAGGTTTCATGTTGCGTCAATTCG 32850

Lser_US96UC23 32801 TGCTCTCCACGGCGCTTTCGGACTAATAGGTTTCATGTTGCGTCAATTCG 32850

Lser_LAC005780 32801 TGCTCTCCACGGCGCTTTCGGACTAATAGGTTTCATGTTGCGTCAATTCG 32850

Lvir_CGN013357 32799 TGCTCTCCACGGCGCTTTCGGACTAATAGGTTTCATGTTGCGTCAATTCG 32848

Lsal_LAC008020 32797 TGCTCTCCACGGCGCTTTCGGACTAATAGGTTTCATGTTGCGTCAATTCG 32846

Lsat_Salinas 32851 AACTTGCGCGATCTGTTCAATTGCGACCTTATAATGCAATCGCATTCTCT 32900

Lsat_WENDEL 32851 AACTTGCGCGATCTGTTCAATTGCGACCTTATAATGCAATCGCATTCTCT 32900

Lser_US96UC23 32851 AACTTGCGCGATCTGTTCAATTGCGACCTTATAATGCAATCGCATTCTCT 32900

Lser_LAC005780 32851 AACTTGCGCGATCTGTTCAATTGCGACCTTATAATGCAATCGCATTCTCT 32900

Lvir_CGN013357 32849 AACTTGCGCGATCTGTTCAATTGCGACCTTATAATGCAATCGCATTCTCT 32898

Lsal_LAC008020 32847 AACTTGCGCGATCTGTTCAATTGCGACCTTATAATGCAATCGCATTCTCT 32896

Lsat_Salinas 32901 GGTCCAATTGCCGTTTTTGTTTCTGTATTCCTGATTTATCCACTAGGTCA 32950

Lsat_WENDEL 32901 GGTCCAATTGCCGTTTTTGTTTCTGTATTCCTGATTTATCCACTAGGTCA 32950

Lser_US96UC23 32901 GGTCCAATTGCCGTTTTTGTTTCTGTATTCCTGATTTATCCACTAGGTCA 32950

Lser_LAC005780 32901 GGTCCAATTGCCGTTTTTGTTTCTGTATTCCTGATTTATCCACTAGGTCA 32950

Lvir_CGN013357 32899 GGTCCAATTGCCGTTTTTGTTTCTGTATTCCTGATTTATCCACTAGGTCA 32948

Lsal_LAC008020 32897 GGTCCAATTGCCGTTTTTGTTTCTGTATTCCTGATTTATCCACTAGGTCA 32946

Lsat_Salinas 32951 GTCTGGTTGGTTCTTTGCGCCTAGTTTTGGTGTAGCAGCTATATTTCGAT 33000

Lsat_WENDEL 32951 GTCTGGTTGGTTCTTTGCGCCTAGTTTTGGTGTAGCAGCTATATTTCGAT 33000

Lser_US96UC23 32951 GTCTGGTTGGTTCTTTGCGCCTAGTTTTGGTGTAGCAGCTATATTTCGAT 33000

Lser_LAC005780 32951 GTCTGGTTGGTTCTTTGCGCCTAGTTTTGGTGTAGCAGCTATATTTCGAT 33000

Lvir_CGN013357 32949 GTCTGGTTGGTTCTTTGCGCCTAGTTTTGGTGTAGCAGCTATATTTCGAT 32998

Lsal_LAC008020 32947 GTCTGGTTGGTTCTTTGCGCCTAGTTTTGGTGTAGCAGCTATATTTCGAT 32996

Lsat_Salinas 33001 TTATCCTCTTTTTTCAAGGATTTCATAACTGGACATTGAACCCATTTCAT 33050

Lsat_WENDEL 33001 TTATCCTCTTTTTTCAAGGATTTCATAACTGGACATTGAACCCATTTCAT 33050

Lser_US96UC23 33001 TTATCCTCTTTTTTCAAGGATTTCATAACTGGACATTGAACCCATTTCAT 33050

Lser_LAC005780 33001 TTATCCTCTTTTTTCAAGGATTTCATAACTGGACATTGAACCCATTTCAT 33050

Lvir_CGN013357 32999 TTATCCTCTTTTTTCAAGGATTTCATAACTGGACATTGAACCCATTTCAT 33048

Lsal_LAC008020 32997 TTATCCTCTTTTTTCAAGGATTTCATAACTGGACATTGAACCCATTTCAT 33046

Lsat_Salinas 33051 ATGATGGGAGTTGCAGGTGTATTGGGCGCTGCTTTGCTATGCGCTATTCA 33100

Lsat_WENDEL 33051 ATGATGGGAGTTGCAGGTGTATTGGGCGCTGCTTTGCTATGCGCTATTCA 33100

Lser_US96UC23 33051 ATGATGGGAGTTGCAGGTGTATTGGGCGCTGCTTTGCTATGCGCTATTCA 33100

Lser_LAC005780 33051 ATGATGGGAGTTGCAGGTGTATTGGGCGCTGCTTTGCTATGCGCTATTCA 33100

Lvir_CGN013357 33049 ATGATGGGAGTTGCAGGTGTATTGGGCGCTGCTTTGCTATGCGCTATTCA 33098

Lsal_LAC008020 33047 ATGATGGGAGTTGCAGGTGTATTGGGCGCTGCTTTGCTATGCGCTATTCA 33096

Lsat_Salinas 33101 TGGTGCTACCGTAGAAAATACTTTATTTGAAGATGGTGATGGGGCAAATA 33150

Lsat_WENDEL 33101 TGGTGCTACCGTAGAAAATACTTTATTTGAAGATGGTGATGGGGCAAATA 33150

Lser_US96UC23 33101 TGGTGCTACCGTAGAAAATACTTTATTTGAAGATGGTGATGGGGCAAATA 33150

Lser_LAC005780 33101 TGGTGCTACCGTAGAAAATACTTTATTTGAAGATGGTGATGGGGCAAATA 33150

Lvir_CGN013357 33099 TGGTGCTACCGTAGAAAATACTTTATTTGAAGATGGTGATGGGGCAAATA 33148

Lsal_LAC008020 33097 TGGTGCTACCGTAGAAAATACTTTATTTGAAGATGGTGATGGGGCAAATA 33146

Lsat_Salinas 33151 CATTCCGTGCTTTTAACCCAACTCAAGCTGAAGAAACTTATTCAATGGTC 33200

Lsat_WENDEL 33151 CATTCCGTGCTTTTAACCCAACTCAAGCTGAAGAAACTTATTCAATGGTC 33200

Lser_US96UC23 33151 CATTCCGTGCTTTTAACCCAACTCAAGCTGAAGAAACTTATTCAATGGTC 33200

Lser_LAC005780 33151 CATTCCGTGCTTTTAACCCAACTCAAGCTGAAGAAACTTATTCAATGGTC 33200

Lvir_CGN013357 33149 CATTCCGTGCTTTTAACCCAACTCAAGCCGAAGAAACTTATTCAATGGTC 33198

Lsal_LAC008020 33147 CATTCCGTGCTTTTAACCCAACTCAAGCTGAAGAAACTTATTCAATGGTC 33196

Lsat_Salinas 33201 ACTGCTAATCGTTTTTGGTCCCAAATCTTTGGGGTTGCTTTTTCCAATAA 33250

Lsat_WENDEL 33201 ACTGCTAATCGTTTTTGGTCCCAAATCTTTGGGGTTGCTTTTTCCAATAA 33250

Lser_US96UC23 33201 ACTGCTAATCGTTTTTGGTCCCAAATCTTTGGGGTTGCTTTTTCCAATAA 33250

Lser_LAC005780 33201 ACTGCTAATCGTTTTTGGTCCCAAATCTTTGGGGTTGCTTTTTCCAATAA 33250

Lvir_CGN013357 33199 ACTGCTAATCGCTTTTGGTCCCAAATCTTTGGGGTTGCTTTTTCCAATAA 33248

Lsal_LAC008020 33197 ACTGCTAATCGCTTTTGGTCCCAAATCTTTGGGGTTGCTTTTTCCAATAA 33246

Lsat_Salinas 33251 ACGTTGGTTACATTTCTTTATGTTATTTGTACCAGTAACTGGTTTATGGA 33300

Lsat_WENDEL 33251 ACGTTGGTTACATTTCTTTATGTTATTTGTACCAGTAACTGGTTTATGGA 33300

Lser_US96UC23 33251 ACGTTGGTTACATTTCTTTATGTTATTTGTACCAGTAACTGGTTTATGGA 33300

Lser_LAC005780 33251 ACGTTGGTTACATTTCTTTATGTTATTTGTACCAGTAACTGGTTTATGGA 33300

Lvir_CGN013357 33249 ACGTTGGTTACATTTCTTTATGTTATTTGTACCAGTAACTGGTTTATGGA 33298

Lsal_LAC008020 33247 ACGTTGGTTACATTTCTTTATGTTATTTGTACCAGTAACTGGTTTATGGA 33296

Lsat_Salinas 33301 TGAGTGCTCTTGGAGTAGTCGGTCTGGCCTTGAACCTACGTGCCTATGAC 33350

Lsat_WENDEL 33301 TGAGTGCTCTTGGAGTAGTCGGTCTGGCCTTGAACCTACGTGCCTATGAC 33350

Lser_US96UC23 33301 TGAGTGCTCTTGGAGTAGTCGGTCTGGCCTTGAACCTACGTGCCTATGAC 33350

Lser_LAC005780 33301 TGAGTGCTCTTGGAGTAGTCGGTCTGGCCTTGAACCTACGTGCCTATGAC 33350

Lvir_CGN013357 33299 TGAGTGCTCTTGGAGTAGTCGGTCTGGCCTTGAACCTACGTGCCTATGAC 33348

Lsal_LAC008020 33297 TGAGTGCTCTTGGAGTAGTCGGTCTGGCCTTGAACCTACGTGCCTATGAC 33346

Lsat_Salinas 33351 TTCGTTTCTCAGGAAATTCGCGCGGCGGAAGATCCTGAATTTGAAACTTT 33400

Lsat_WENDEL 33351 TTCGTTTCTCAGGAAATTCGCGCGGCGGAAGATCCTGAATTTGAAACTTT 33400

Lser_US96UC23 33351 TTCGTTTCTCAGGAAATTCGCGCGGCGGAAGATCCTGAATTTGAAACTTT 33400

Lser_LAC005780 33351 TTCGTTTCTCAGGAAATTCGCGCGGCGGAAGATCCTGAATTTGAAACTTT 33400

Lvir_CGN013357 33349 TTCGTTTCTCAGGAAATTCGCGCGGCGGAAGATCCTGAATTTGAAACTTT 33398

Lsal_LAC008020 33347 TTCGTTTCTCAGGAAATTCGCGCGGCGGAAGATCCTGAATTTGAAACTTT 33396

Lsat_Salinas 33401 CTACACCAAAAATATTCTCTTAAACGAAGGTATTCGTGCTTGGATGGCGG 33450

Lsat_WENDEL 33401 CTACACCAAAAATATTCTCTTAAACGAAGGTATTCGTGCTTGGATGGCGG 33450

Lser_US96UC23 33401 CTACACCAAAAATATTCTCTTAAACGAAGGTATTCGTGCTTGGATGGCGG 33450

Lser_LAC005780 33401 CTACACCAAAAATATTCTCTTAAACGAAGGTATTCGTGCTTGGATGGCGG 33450

Lvir_CGN013357 33399 CTACACCAAAAATATTCTCTTAAACGAAGGTATTCGTGCTTGGATGGCGG 33448

Lsal_LAC008020 33397 CTACACCAAAAATATTCTCTTAAACGAAGGTATTCGTGCTTGGATGGCGG 33446

Lsat_Salinas 33451 CTCAAGATCAGCCTCATGAAAACCTTATATTCCCTGAGGAGGTTCTACCC 33500

Lsat_WENDEL 33451 CTCAAGATCAGCCTCATGAAAACCTTATATTCCCTGAGGAGGTTCTACCC 33500

Lser_US96UC23 33451 CTCAAGATCAGCCTCATGAAAACCTTATATTCCCTGAGGAGGTTCTACCC 33500

Lser_LAC005780 33451 CTCAAGATCAGCCTCATGAAAACCTTATATTCCCTGAGGAGGTTCTACCC 33500

Lvir_CGN013357 33449 CTCAAGATCAGCCTCATGAAAACCTTATATTCCCTGAGGAGGTTCTACCC 33498

Lsal_LAC008020 33447 CTCAAGATCAGCCTCATGAAAACCTTATATTCCCTGAGGAGGTTCTACCC 33496

Lsat_Salinas 33501 CGTGGAAACGCTCTTTAATGGAACTTTAGCTTTAGCTGGTCGTGACCAAG 33550

Lsat_WENDEL 33501 CGTGGAAACGCTCTTTAATGGAACTTTAGCTTTAGCTGGTCGTGACCAAG 33550

Lser_US96UC23 33501 CGTGGAAACGCTCTTTAATGGAACTTTAGCTTTAGCTGGTCGTGACCAAG 33550

Lser_LAC005780 33501 CGTGGAAACGCTCTTTAATGGAACTTTAGCTTTAGCTGGTCGTGACCAAG 33550

Lvir_CGN013357 33499 CGTGGAAACGCTCTTTAATGGAACTTTAGCTTTAGCTGGTCGTGACCAAG 33548

Lsal_LAC008020 33497 CGTGGAAACGCTCTTTAATGGAACTTTAGCTTTAGCTGGTCGTGACCAAG 33546

Lsat_Salinas 33551 AAACCACTGGTTTCGCTTGGTGGGCCGGGAATGCCCGGCTTATCAATTTA 33600

Lsat_WENDEL 33551 AAACCACTGGTTTCGCTTGGTGGGCCGGGAATGCCCGGCTTATCAATTTA 33600

Lser_US96UC23 33551 AAACCACTGGTTTCGCTTGGTGGGCCGGGAATGCCCGGCTTATCAATTTA 33600

Lser_LAC005780 33551 AAACCACTGGTTTCGCTTGGTGGGCCGGGAATGCCCGGCTTATCAATTTA 33600

Lvir_CGN013357 33549 AAACCACCGGTTTCGCTTGGTGGGCCGGGAATGCCCGGCTTATCAATTTA 33598

Lsal_LAC008020 33547 AAACCACCGGTTTCGCTTGGTGGGCCGGGAATGCCCGGCTTATCAATTTA 33596

Lsat_Salinas 33601 TCTGGTAAACTACTAGGGGCTCATGTAGCCCATGCCGGATTAATCGTATT 33650

Lsat_WENDEL 33601 TCTGGTAAACTACTAGGGGCTCATGTAGCCCATGCCGGATTAATCGTATT 33650

Lser_US96UC23 33601 TCTGGTAAACTACTAGGGGCTCATGTAGCCCATGCCGGATTAATCGTATT 33650

Lser_LAC005780 33601 TCTGGTAAACTACTAGGGGCTCATGTAGCCCATGCCGGATTAATCGTATT 33650

Lvir_CGN013357 33599 TCTGGTAAACTACTAGGGGCTCATGTAGCCCATGCCGGATTAATCGTATT 33648

Lsal_LAC008020 33597 TCTGGTAAACTACTAGGGGCTCATGTAGCCCATGCCGGATTAATCGTATT 33646

Lsat_Salinas 33651 TTGGGCCGGAGCAATGAATCTATTTGAAGTGGCTCATTTCGTACCAGAGA 33700

Lsat_WENDEL 33651 TTGGGCCGGAGCAATGAATCTATTTGAAGTGGCTCATTTCGTACCAGAGA 33700

Lser_US96UC23 33651 TTGGGCCGGAGCAATGAATCTATTTGAAGTGGCTCATTTCGTACCAGAGA 33700

Lser_LAC005780 33651 TTGGGCCGGAGCAATGAATCTATTTGAAGTGGCTCATTTCGTACCAGAGA 33700

Lvir_CGN013357 33649 CTGGGCCGGAGCAATGAATCTATTTGAAGTGGCTCATTTCGTACCAGAGA 33698

Lsal_LAC008020 33647 CTGGGCCGGAGCAATGAATCTATTTGAAGTGGCTCATTTCGTACCAGAGA 33696

Lsat_Salinas 33701 AGCCTATGTATGAACAAGGATTAATTTTACTTCCCCACCTAGCTACTCTA 33750

Lsat_WENDEL 33701 AGCCTATGTATGAACAAGGATTAATTTTACTTCCCCACCTAGCTACTCTA 33750

Lser_US96UC23 33701 AGCCTATGTATGAACAAGGATTAATTTTACTTCCCCACCTAGCTACTCTA 33750

Lser_LAC005780 33701 AGCCTATGTATGAACAAGGATTAATTTTACTTCCCCACCTAGCTACTCTA 33750

Lvir_CGN013357 33699 AGCCTATGTATGAACAAGGATTAATTTTACTTCCCCACCTAGCTACTCTA 33748

Lsal_LAC008020 33697 AGCCTATGTATGAACAAGGATTAATTTTACTTCCCCACCTAGCTACTCTA 33746

Lsat_Salinas 33751 GGTTGGGGGGTGGGTCCTGGTGGGGAAGTTATAGATACTTTTCCATACTT 33800

Lsat_WENDEL 33751 GGTTGGGGGGTGGGTCCTGGTGGGGAAGTTATAGATACTTTTCCATACTT 33800

Lser_US96UC23 33751 GGTTGGGGGGTGGGTCCTGGTGGGGAAGTTATAGATACTTTTCCATACTT 33800

Lser_LAC005780 33751 GGTTGGGGGGTGGGTCCTGGTGGGGAAGTTATAGATACTTTTCCATACTT 33800

Lvir_CGN013357 33749 GGTTGGGGGGTGGGTCCTGGTGGGGAAGTTATAGATACTTTTCCATACTT 33798

Lsal_LAC008020 33747 GGTTGGGGGGTGGGTCCTGGTGGGGAAGTTATAGATACTTTTCCATACTT 33796

Lsat_Salinas 33801 TGTATCCGGAGTACTTCATTTAATTTCCTCTGCAGTATTGGGCTTTGGCG 33850

Lsat_WENDEL 33801 TGTATCCGGAGTACTTCATTTAATTTCCTCTGCAGTATTGGGCTTTGGCG 33850

Lser_US96UC23 33801 TGTATCCGGAGTACTTCATTTAATTTCCTCTGCAGTATTGGGCTTTGGCG 33850

Lser_LAC005780 33801 TGTATCCGGAGTACTTCATTTAATTTCCTCTGCAGTATTGGGCTTTGGCG 33850

Lvir_CGN013357 33799 TGTATCCGGAGTACTTCATTTAATTTCCTCTGCAGTATTGGGCTTTGGCG 33848

Lsal_LAC008020 33797 TGTATCCGGAGTACTTCATTTAATTTCCTCTGCAGTATTGGGCTTTGGCG 33846

Lsat_Salinas 33851 GTATTTATCATGCACTTTTAGGGCCTGAGACGCTTGAAGAATCTTTTCCA 33900

Lsat_WENDEL 33851 GTATTTATCATGCACTTTTAGGGCCTGAGACGCTTGAAGAATCTTTTCCA 33900

Lser_US96UC23 33851 GTATTTATCATGCACTTTTAGGGCCTGAGACGCTTGAAGAATCTTTTCCA 33900

Lser_LAC005780 33851 GTATTTATCATGCACTTTTAGGGCCTGAGACGCTTGAAGAATCTTTTCCA 33900

Lvir_CGN013357 33849 GTATTTATCATGCACTTTTAGGGCCTGAGACGCTTGAAGAATCTTTTCCA 33898

Lsal_LAC008020 33847 GTATTTATCATGCACTTTTAGGGCCTGAGACGCTTGAAGAATCTTTTCCA 33896

Lsat_Salinas 33901 TTCTTCGGTTATGTATGGAAAGATAGAAATAAAATGACCACAATTTTAGG 33950

Lsat_WENDEL 33901 TTCTTCGGTTATGTATGGAAAGATAGAAATAAAATGACCACAATTTTAGG 33950

Lser_US96UC23 33901 TTCTTCGGTTATGTATGGAAAGATAGAAATAAAATGACCACAATTTTAGG 33950

Lser_LAC005780 33901 TTCTTCGGTTATGTATGGAAAGATAGAAATAAAATGACCACAATTTTAGG 33950

Lvir_CGN013357 33899 TTCTTCGGTTATGTATGGAAAGATAGAAATAAAATGACCACAATTTTAGG 33948

Lsal_LAC008020 33897 TTCTTCGGTTATGTATGGAAAGATAGAAATAAAATGACCACAATTTTAGG 33946

Lsat_Salinas 33951 TATTCACTTAATCTTGTTAGGTATAGGTGCTTTTCTTCTAGTATTCAAGG 34000

Lsat_WENDEL 33951 TATTCACTTAATCTTGTTAGGTATAGGTGCTTTTCTTCTAGTATTCAAGG 34000

Lser_US96UC23 33951 TATTCACTTAATCTTGTTAGGTATAGGTGCTTTTCTTCTAGTATTCAAGG 34000

Lser_LAC005780 33951 TATTCACTTAATCTTGTTAGGTATAGGTGCTTTTCTTCTAGTATTCAAGG 34000

Lvir_CGN013357 33949 TATTCACTTAATCTTGTTAGGTATAGGTGCTTTTCTTCTAGTATTCAAGG 33998

Lsal_LAC008020 33947 TATTCACTTAATCTTGTTAGGTATAGGTGCTTTTCTTCTAGTATTCAAGG 33996

Lsat_Salinas 34001 CTCTTTATTTTGGGGGCGTATATGATACTTGGGCTCCGGGAGGAGGAGAT 34050

Lsat_WENDEL 34001 CTCTTTATTTTGGGGGCGTATATGATACTTGGGCTCCGGGAGGAGGAGAT 34050

Lser_US96UC23 34001 CTCTTTATTTTGGGGGCGTATATGATACTTGGGCTCCGGGAGGAGGAGAT 34050

Lser_LAC005780 34001 CTCTTTATTTTGGGGGCGTATATGATACTTGGGCTCCGGGAGGAGGAGAT 34050

Lvir_CGN013357 33999 CTCTTTATTTTGGGGGCGTATATGATACTTGGGCTCCGGGAGGAGGAGAT 34048

Lsal_LAC008020 33997 CTCTTTATTTTGGGGGCGTATATGATACTTGGGCTCCGGGAGGAGGAGAT 34046

Lsat_Salinas 34051 GTAAGAAAAATAACCAACTTGACGCTTAGCCCAAGTATCATATTTGGTTA 34100

Lsat_WENDEL 34051 GTAAGAAAAATAACCAACTTGACGCTTAGCCCAAGTATCATATTTGGTTA 34100

Lser_US96UC23 34051 GTAAGAAAAATAACCAACTTGACGCTTAGCCCAAGTATCATATTTGGTTA 34100

Lser_LAC005780 34051 GTAAGAAAAATAACCAACTTGACGCTTAGCCCAAGTATCATATTTGGTTA 34100

Lvir_CGN013357 34049 GTAAGAAAAATAACCAACTTGACGCTTAGCCCAAGTATCATATTTGGTTA 34098

Lsal_LAC008020 34047 GTAAGAAAAATAACCAACTTGACGCTTAGCCCAAGTATCATATTTGGTTA 34096

Lsat_Salinas 34101 TTTACTAAAATCGCCCTTTGGGGGAGAAGGATGGATTGTTAGTGTAGACG 34150

Lsat_WENDEL 34101 TTTACTAAAATCGCCCTTTGGGGGAGAAGGATGGATTGTTAGTGTAGACG 34150

Lser_US96UC23 34101 TTTACTAAAATCGCCCTTTGGGGGAGAAGGATGGATTGTTAGTGTAGACG 34150

Lser_LAC005780 34101 TTTACTAAAATCGCCCTTTGGGGGAGAAGGATGGATTGTTAGTGTAGACG 34150

Lvir_CGN013357 34099 TTTACTAAAATCGCCCTTTGGGGGAGAAGGATGGATTGTTAGTGTAGACG 34148

Lsal_LAC008020 34097 TTTACTAAAATCGCCCTTTGGGGGAGAAGGATGGATTGTTAGTGTAGACG 34146

Lsat_Salinas 34151 ATTTGGAAGATATAATCGGAGGCCATGTATGGTTAGGTTCCATTTGTATA 34200

Lsat_WENDEL 34151 ATTTGGAAGATATAATCGGAGGCCATGTATGGTTAGGTTCCATTTGTATA 34200

Lser_US96UC23 34151 ATTTGGAAGATATAATCGGAGGCCATGTATGGTTAGGTTCCATTTGTATA 34200

Lser_LAC005780 34151 ATTTGGAAGATATAATCGGAGGCCATGTATGGTTAGGTTCCATTTGTATA 34200

Lvir_CGN013357 34149 ATTTGGAAGATATAATCGGAGGCCATGTATGGTTAGGTTCCATTTGTATA 34198

Lsal_LAC008020 34147 ATTTGGAAGATATAATCGGAGGCCATGTATGGTTAGGTTCCATTTGTATA 34196

Lsat_Salinas 34201 CTTGGTGGAATTTGGCATATCTTAACCAAACCCTTCGCATGGGCTCGACG 34250

Lsat_WENDEL 34201 CTTGGTGGAATTTGGCATATCTTAACCAAACCCTTCGCATGGGCTCGACG 34250

Lser_US96UC23 34201 CTTGGTGGAATTTGGCATATCTTAACCAAACCCTTCGCATGGGCTCGACG 34250

Lser_LAC005780 34201 CTTGGTGGAATTTGGCATATCTTAACCAAACCCTTCGCATGGGCTCGACG 34250

Lvir_CGN013357 34199 CTTGGTGGAATTTGGCATATCTTAACCAAACCCTTCGCATGGGCTCGACG 34248

Lsal_LAC008020 34197 CTTGGTGGAATTTGGCATATCTTAACCAAACCCTTCGCATGGGCTCGACG 34246

Lsat_Salinas 34251 AGCACTTGTATGGTCTGGAGAGGCTTACTTATCTTATAGTTTAGCGGCTA 34300

Lsat_WENDEL 34251 AGCACTTGTATGGTCTGGAGAGGCTTACTTATCTTATAGTTTAGCGGCTA 34300

Lser_US96UC23 34251 AGCACTTGTATGGTCTGGAGAGGCTTACTTATCTTATAGTTTAGCGGCTA 34300

Lser_LAC005780 34251 AGCACTTGTATGGTCTGGAGAGGCTTACTTATCTTATAGTTTAGCGGCTA 34300

Lvir_CGN013357 34249 AGCACTTGTATGGTCTGGAGAGGCTTACTTATCTTATAGTTTAGCGGCTA 34298

Lsal_LAC008020 34247 AGCACTTGTATGGTCTGGAGAGGCTTACTTATCTTATAGTTTAGCGGCTA 34296

Lsat_Salinas 34301 TATCTGTCTTTGGTTTCATTGCTTGTTGTTTTGTCTGGTTCAATAATACC 34350

Lsat_WENDEL 34301 TATCTGTCTTTGGTTTCATTGCTTGTTGTTTTGTCTGGTTCAATAATACC 34350

Lser_US96UC23 34301 TATCTGTCTTTGGTTTCATTGCTTGTTGTTTTGTCTGGTTCAATAATACC 34350

Lser_LAC005780 34301 TATCTGTCTTTGGTTTCATTGCTTGTTGTTTTGTCTGGTTCAATAATACC 34350

Lvir_CGN013357 34299 TATCTGTCTTTGGTTTCATTGCTTGTTGTTTTGTCTGGTTCAATAATACC 34348

Lsal_LAC008020 34297 TATCTGTCTTTGGTTTCATTGCTTGTTGTTTTGTCTGGTTCAATAATACC 34346

Lsat_Salinas 34351 GCTTATCCTAGTGAGTTTTACGGACCCACTGGACCAGAAGCTTCTCAAGC 34400

Lsat_WENDEL 34351 GCTTATCCTAGTGAGTTTTACGGACCCACTGGACCAGAAGCTTCTCAAGC 34400

Lser_US96UC23 34351 GCTTATCCTAGTGAGTTTTACGGACCCACTGGACCAGAAGCTTCTCAAGC 34400

Lser_LAC005780 34351 GCTTATCCTAGTGAGTTTTACGGACCCACTGGACCAGAAGCTTCTCAAGC 34400

Lvir_CGN013357 34349 GCTTATCCTAGTGAGTTTTACGGACCCACTGGACCAGAAGCTTCTCAAGC 34398

Lsal_LAC008020 34347 GCTTATCCTAGTGAGTTTTACGGACCCACTGGACCAGAAGCTTCTCAAGC 34396

Lsat_Salinas 34401 TCAAGCATTTACTTTTCTAGTTAGAGACCAACGTCTTGGAGCTAACGTTG 34450

Lsat_WENDEL 34401 TCAAGCATTTACTTTTCTAGTTAGAGACCAACGTCTTGGAGCTAACGTTG 34450

Lser_US96UC23 34401 TCAAGCATTTACTTTTCTAGTTAGAGACCAACGTCTTGGAGCTAACGTTG 34450

Lser_LAC005780 34401 TCAAGCATTTACTTTTCTAGTTAGAGACCAACGTCTTGGAGCTAACGTTG 34450

Lvir_CGN013357 34399 TCAAGCATTTACTTTTCTAGTTAGAGACCAACGTCTTGGAGCTAACGTTG 34448

Lsal_LAC008020 34397 TCAAGCATTTACTTTTCTAGTTAGAGACCAACGTCTTGGAGCTAACGTTG 34446

Lsat_Salinas 34451 GATCTGCTCAAGGTCCTACCGGTTTAGGTAAATATTTAATGCGTTCCCCG 34500

Lsat_WENDEL 34451 GATCTGCTCAAGGTCCTACCGGTTTAGGTAAATATTTAATGCGTTCCCCG 34500

Lser_US96UC23 34451 GATCTGCTCAAGGTCCTACCGGTTTAGGTAAATATTTAATGCGTTCCCCG 34500

Lser_LAC005780 34451 GATCTGCTCAAGGTCCTACCGGTTTAGGTAAATATTTAATGCGTTCCCCG 34500

Lvir_CGN013357 34449 GATCTGCTCAAGGTCCTACCGGTTTAGGTAAATATTTAATGCGTTCCCCG 34498

Lsal_LAC008020 34447 GATCTGCTCAAGGTCCTACCGGTTTAGGTAAATATTTAATGCGTTCCCCG 34496

Lsat_Salinas 34501 ACTGGAGAAGTCATTTTTGGAGGAGAAACTATGCGTTTTTGGGATCTGCG 34550

Lsat_WENDEL 34501 ACTGGAGAAGTCATTTTTGGAGGAGAAACTATGCGTTTTTGGGATCTGCG 34550

Lser_US96UC23 34501 ACTGGAGAAGTCATTTTTGGAGGAGAAACTATGCGTTTTTGGGATCTGCG 34550

Lser_LAC005780 34501 ACTGGAGAAGTCATTTTTGGAGGAGAAACTATGCGTTTTTGGGATCTGCG 34550

Lvir_CGN013357 34499 ACCGGAGAAGTCATTTTTGGAGGAGAAACTATGCGTTTTTGGGATCTGCG 34548

Lsal_LAC008020 34497 ACTGGAGAAGTCATTTTTGGAGGAGAAACTATGCGTTTTTGGGATCTGCG 34546

Lsat_Salinas 34551 TGCTCCTTGGTTAGAACCCCTAAGGGGTCCAAATGGGTTGGACTTAAGTC 34600

Lsat_WENDEL 34551 TGCTCCTTGGTTAGAACCCCTAAGGGGTCCAAATGGGTTGGACTTAAGTC 34600

Lser_US96UC23 34551 TGCTCCTTGGTTAGAACCCCTAAGGGGTCCAAATGGGTTGGACTTAAGTC 34600

Lser_LAC005780 34551 TGCTCCTTGGTTAGAACCCCTAAGGGGTCCAAATGGGTTGGACTTAAGTC 34600

Lvir_CGN013357 34549 TGCTCCTTGGTTAGAACCCCTAAGGGGTCCAAATGGGTTGGACTTAAGTC 34598

Lsal_LAC008020 34547 TGCTCCTTGGTTAGAACCCCTAAGGGGTCCAAATGGGTTGGACTTAAGTC 34596

Lsat_Salinas 34601 GGCTGAAAAAAGACATACAACCTTGGCAAGAACGTCGTTCCGCAGAATAT 34650

Lsat_WENDEL 34601 GGCTGAAAAAAGACATACAACCTTGGCAAGAACGTCGTTCCGCAGAATAT 34650

Lser_US96UC23 34601 GGCTGAAAAAAGACATACAACCTTGGCAAGAACGTCGTTCCGCAGAATAT 34650

Lser_LAC005780 34601 GGCTGAAAAAAGACATACAACCTTGGCAAGAACGTCGTTCCGCAGAATAT 34650

Lvir_CGN013357 34599 GGCTGAAAAAAGACATACAACCTTGGCAAGAACGTCGTTCCGCAGAATAT 34648

Lsal_LAC008020 34597 GGCTGAAAAAAGACATACAACCTTGGCAAGAGCGTCGTTCCGCAGAATAT 34646

Lsat_Salinas 34651 ATGACCCATGCTCCTTTAGGTTCTTTAAATTCCGTGGGTGGCGTAGCTAC 34700

Lsat_WENDEL 34651 ATGACCCATGCTCCTTTAGGTTCTTTAAATTCCGTGGGTGGCGTAGCTAC 34700

Lser_US96UC23 34651 ATGACCCATGCTCCTTTAGGTTCTTTAAATTCCGTGGGTGGCGTAGCTAC 34700

Lser_LAC005780 34651 ATGACCCATGCTCCTTTAGGTTCTTTAAATTCCGTGGGTGGCGTAGCTAC 34700

Lvir_CGN013357 34649 ATGACCCATGCTCCTTTAGGTTCTTTAAATTCCGTGGGTGGCGTAGCTAC 34698

Lsal_LAC008020 34647 ATGACCCATGCTCCTTTAGGTTCTTTAAATTCCGTGGGTGGCGTAGCTAC 34696

Lsat_Salinas 34701 CGAGATCAATGCAGTCAATTATGTCTCTCCTAGAAGTTGGTTAGCTACTT 34750

Lsat_WENDEL 34701 CGAGATCAATGCAGTCAATTATGTCTCTCCTAGAAGTTGGTTAGCTACTT 34750

Lser_US96UC23 34701 CGAGATCAATGCAGTCAATTATGTCTCTCCTAGAAGTTGGTTAGCTACTT 34750

Lser_LAC005780 34701 CGAGATCAATGCAGTCAATTATGTCTCTCCTAGAAGTTGGTTAGCTACTT 34750

Lvir_CGN013357 34699 CGAGATCAATGCAGTCAATTATGTCTCTCCTAGAAGTTGGTTAGCTACTT 34748

Lsal_LAC008020 34697 CGAGATCAATGCAGTCAATTATGTCTCTCCTAGAAGTTGGTTAGCTACTT 34746

Lsat_Salinas 34751 CTCATTTTGTTCTAGGATTCTTCTTCTTCGTAGGGCATTTGTGGCACGCA 34800

Lsat_WENDEL 34751 CTCATTTTGTTCTAGGATTCTTCTTCTTCGTAGGGCATTTGTGGCACGCA 34800

Lser_US96UC23 34751 CTCATTTTGTTCTAGGATTCTTCTTCTTCGTAGGGCATTTGTGGCACGCA 34800

Lser_LAC005780 34751 CTCATTTTGTTCTAGGATTCTTCTTCTTCGTAGGGCATTTGTGGCACGCA 34800

Lvir_CGN013357 34749 CTCATTTTGTTCTAGGATTCTTCTTCTTCGTAGGGCATTTGTGGCACGCA 34798

Lsal_LAC008020 34747 CTCATTTTGTTCTAGGATTCTTCTTCTTCGTAGGGCATTTGTGGCACGCA 34796

Lsat_Salinas 34801 GGAAGAGCTCGTGCAGCTGCAGCAGGATTTGAAAAAGGAATTGATCGTGA 34850

Lsat_WENDEL 34801 GGAAGAGCTCGTGCAGCTGCAGCAGGATTTGAAAAAGGAATTGATCGTGA 34850

Lser_US96UC23 34801 GGAAGAGCTCGTGCAGCTGCAGCAGGATTTGAAAAAGGAATTGATCGTGA 34850

Lser_LAC005780 34801 GGAAGAGCTCGTGCAGCTGCAGCAGGATTTGAAAAAGGAATTGATCGTGA 34850

Lvir_CGN013357 34799 GGAAGAGCTCGTGCAGCTGCAGCAGGATTTGAAAAAGGAATTGATCGTGA 34848

Lsal_LAC008020 34797 GGAAGAGCTCGCGCAGCTGCAGCAGGATTTGAAAAAGGAATTGATCGTGA 34846

Lsat_Salinas 34851 TTTTGAACCTGTTCTTTCCATGACCCCTCTTAATTGAGACAGGAGATCAA 34900

Lsat_WENDEL 34851 TTTTGAACCTGTTCTTTCCATGACCCCTCTTAATTGAGACAGGAGATCAA 34900

Lser_US96UC23 34851 TTTTGAACCTGTTCTTTCCATGACCCCTCTTAATTGAGACAGGAGATCAA 34900

Lser_LAC005780 34851 TTTTGAACCTGTTCTTTCCATGACCCCTCTTAATTGAGACAGGAGATCAA 34900

Lvir_CGN013357 34849 TTTTGAACCTGTTCTTTCCATGACCCCTCTTAATTGAGACAGGAGATCAA 34898

Lsal_LAC008020 34847 TTTTGAACCTGTTCTTTCCATGACCCCTCTTAATTGAGACAGGAGATCAA 34896

Lsat_Salinas 34901 AGACTTGAACTAGGAGTCACTTCGGTTCCTTCATACATATAGGGATCATG 34950

Lsat_WENDEL 34901 AGACTTGAACTAGGAGTCACTTCGGTTCCTTCATACATATAGGGATCATG 34950

Lser_US96UC23 34901 AGACTTGAACTAGGAGTCACTTCGGTTCCTTCATACATATAGGGATCATG 34950

Lser_LAC005780 34901 AGACTTGAACTAGGAGTCACTTCGGTTCCTTCATACATATAGGGATCATG 34950

Lvir_CGN013357 34899 AGACTTGAACTAGGAGTCACTTCGGTTCCTTCATACATATAGGGATCATG 34948

Lsal_LAC008020 34897 AGACTTGAACTAGGAGTCACTTCGGTTCCTTCATACATATAGGGATCATG 34946

Lsat_Salinas 34951 TCATAAAAAAATATTCTTTTTTTTTTTTTCAACTCATTTATATTTAATTT 35000

Lsat_WENDEL 34951 TCATAAAAAAATATTCTTTTTTTTTTTTTCAACTCATTTATATTTAATTT 35000

Lser_US96UC23 34951 TCATAAAAAAATATTCTTTTTTTTTTTTTCAACTCATTTATATTTAATTT 35000

Lser_LAC005780 34951 TCATAAAAAAATATTCTTTTTTTTTTTTTCAACTCATTTATATTTAATTT 35000

Lvir_CGN013357 34949 TCATAAAAAAATATTCTTTTTTTTTTTTTCAACTCATTTATATTTAATTT 34998

Lsal_LAC008020 34947 TCATAAAAAAATATTCTTTTTTTTTTTTTCAACTCATTTATATTTAATTT 34996

Lsat_Salinas 35001 AATCCATTTTTCTGGCTTGGCTAGGCGGGATAGCCGAGCCACTCCCCTTT 35050

Lsat_WENDEL 35001 AATCCATTTTTCTGGCTTGGCTAGGCGGGATAGCCGAGCCACTCCCCTTT 35050

Lser_US96UC23 35001 AATCCATTTTTCTGGCTTGGCTAGGCGGGATAGCCGAGCCACTCCCCTTT 35050

Lser_LAC005780 35001 AATCCATTTTTCTGGCTTGGCTAGGCGGGATAGCCGAGCCACTCCCCTTT 35050

Lvir_CGN013357 34999 AATCCATTTTTCTGGCTTGGCTAGGCGGGATAGCCGAGCCACTCCCCTTT 35048

Lsal_LAC008020 34997 AATCCATTTTTCTGGCTTGGCTAGGCGGGATAGCCGAGCCACTCCCCTTT 35046

Lsat_Salinas 35051 CTTTATGATAATGAAACCATCCGGTCAAAACCAATCCAATAAAGAAACAA 35100

Lsat_WENDEL 35051 CTTTATGATAATGAAACCATCCGGTCAAAACCAATCCAATAAAGAAACAA 35100

Lser_US96UC23 35051 CTTTATGATAATGAAACCATCCGGTCAAAACCAATCCAATAAAGAAACAA 35100

Lser_LAC005780 35051 CTTTATGATAATGAAACCATCCGGTCAAAACCAATCCAATAAAGAAACAA 35100

Lvir_CGN013357 35049 CTTTATGATAATGAAACCATCCGGTCAAAACCAATCCAATAAAGAAACAA 35098

Lsal_LAC008020 35047 CTTTATGATAATGAAACCATCCGGTCAAAACCAATCCAATAAAGAAACAA 35096

Lsat_Salinas 35101 ATTTATTTAACAAGCAAAAAAGGAGAGAGAGGGATTCGAACCCTCGATAG 35150

Lsat_WENDEL 35101 ATTTATTTAACAAGCAAAAAAGGAGAGAGAGGGATTCGAACCCTCGATAG 35150

Lser_US96UC23 35101 ATTTATTTAACAAGCAAAAAAGGAGAGAGAGGGATTCGAACCCTCGATAG 35150

Lser_LAC005780 35101 ATTTATTTAACAAGCAAAAAAGGAGAGAGAGGGATTCGAACCCTCGATAG 35150

Lvir_CGN013357 35099 ATTTATTTAACAAGCAAAAAAGGAGAGAGAGGGATTCGAACCCTCGATAG 35148

Lsal_LAC008020 35097 ATTTATTTAACAAGCAAAAAAGGAGAGAGAGGGATTCGAACCCTCGATAG 35146

Lsat_Salinas 35151 TAAACTATACCGGTTTTCAAGACCGGGGCTTTCAACCACTCAGCCATCTC 35200

Lsat_WENDEL 35151 TAAACTATACCGGTTTTCAAGACCGGGGCTTTCAACCACTCAGCCATCTC 35200

Lser_US96UC23 35151 TAAACTATACCGGTTTTCAAGACCGGGGCTTTCAACCACTCAGCCATCTC 35200

Lser_LAC005780 35151 TAAACTATACCGGTTTTCAAGACCGGGGCTTTCAACCACTCAGCCATCTC 35200

Lvir_CGN013357 35149 TAAACTATACCGGTTTTCAAGACCGGGGCTTTCAACCACTCAGCCATCTC 35198

Lsal_LAC008020 35147 TAAACTATACCGGTTTTCAAGACCGGGGCTTTCAACCACTCAGCCATCTC 35196

Lsat_Salinas 35201 TCCGAAAGACAATTTTATTTTATTCCTCCTAATAGAACATGGCCATATGA 35250

Lsat_WENDEL 35201 TCCGAAAGACAATTTTATTTTATTCCTCCTAATAGAACATGGCCATATGA 35250

Lser_US96UC23 35201 TCCGAAAGACAATTTTATTTTATTCCTCCTAATAGAACATGGCCATATGA 35250

Lser_LAC005780 35201 TCCGAAAGACAATTTTATTTTATTCCTCCTAATAGAACATGGCCATATGA 35250

Lvir_CGN013357 35199 TCCGAAAGACAATTTTATTTTATTCCTCCGAATAGAACATGGCCATATGA 35248

Lsal_LAC008020 35197 TCCGAAAGACAATTTTATTTTATTCCTCCGAATAGAACATGGCCATATGA 35246

Lsat_Salinas 35251 GTGGATACCGCCACTATCAAAGATCTCGGGTGTGATGGTCAATCTATCTA 35300

Lsat_WENDEL 35251 GTGGATACCGCCACTATCAAAGATCTCGGGTGTGATGGTCAATCTATCTA 35300

Lser_US96UC23 35251 GTGGATACCGCCACTATCAAAGATCTCGGGTGTGATGGTCAATCTATCTA 35300

Lser_LAC005780 35251 GTGGATACCGCCACTATCAAAGATCTCGGGTGTGATGGTCAATCTATCTA 35300

Lvir_CGN013357 35249 GTGGATACCGCCACTATCAAAGATCTCGGGTGTGATGGTCAATATATCTA 35298

Lsal_LAC008020 35247 GTGGATACCGCCACTATCAAAGATCTCGGGTGTGATGGTCAATCTATCTA 35296

Lsat_Salinas 35301 TCCCGATATATGGATAGATATATGATCCAGCATGCCCATTTGTAAAATAA 35350

Lsat_WENDEL 35301 TCCCGATATATGGATAGATATATGATCCAGCATGCCCATTTGTAAAATAA 35350

Lser_US96UC23 35301 TCCCGATATATGGATAGATATATGATCCAGCATGCCCATTTGTAAAATAA 35350

Lser_LAC005780 35301 TCCCGATATATGGATAGATATATGATCCAGCATGCCCATTTGTAAAATAA 35350

Lvir_CGN013357 35299 TCCCGATATATGGATATATATATGATCCAGCATGCCCATTTGTAAAATAA 35348

Lsal_LAC008020 35297 TCCCGATATATGGATAGATATATGATCCAGCATGCCCATTTGTAAAATAA 35346

Lsat_Salinas 35351 AAAAACCAAATTCCATTCTCCCCCGACTCTATGTACGAATAAAGTGGTAA 35400

Lsat_WENDEL 35351 AAAAACCAAATTCCATTCTCCCCCGACTCTATGTACGAATAAAGTGGTAA 35400

Lser_US96UC23 35351 AAAAACCAAATTCCATTCTCCCCCGACTCTATGTACGAATAAAGTGGTAA 35400

Lser_LAC005780 35351 AAAAACCAAATTCCATTCTCCCCCGACTCTATGTACGAATAAAGTGGTAA 35400

Lvir_CGN013357 35349 AAAAACCAAATTCCATTCTCCCCCGACTCTATGTACGAATAAAGTGGTAA 35398

Lsal_LAC008020 35347 AAAAACCAAATTCCATTCTCCCCGGACTCTATGTACGAATAAAGTGGTAA 35396

Lsat_Salinas 35401 AGGGGTAGTAATATAATAAATCATATTGAATCAATGGATTCGTGGTAAAA 35450

Lsat_WENDEL 35401 AGGGGTAGTAATATAATAAATCATATTGAATCAATGGATTCGTGGTAAAA 35450

Lser_US96UC23 35401 AGGGGTAGTAATATAATAAATCATATTGAATCAATGGATTCGTGGTAAAA 35450

Lser_LAC005780 35401 AGGGGTAGTAATATAATAAATCATATTGAATCAATGGATTCGTGGTAAAA 35450

Lvir_CGN013357 35399 AGGGGTAGTAATATAATAAATCATATTGAATCAATGGATTCGTGGTAAAA 35448

Lsal_LAC008020 35397 AGGGGTAGTAATATAATAAATCATATTGAATCAATGGATTCGTGGTAAAA 35446

Lsat_Salinas 35451 TCCCTGTATGATGTATTTTAGTACAATTTTTGGCTGATATAGGGATCAAA 35500

Lsat_WENDEL 35451 TCCCTGTATGATGTATTTTAGTACAATTTTTGGCTGATATAGGGATCAAA 35500

Lser_US96UC23 35451 TCCCTGTATGATGTATTTTAGTACAATTTTTGGCTGATATAGGGATCAAA 35500

Lser_LAC005780 35451 TCCCTGTATGATGTATTTTAGTACAATTTTTGGCTGATATAGGGATCAAA 35500

Lvir_CGN013357 35449 TCCCTGTATGATGTATTTTAGTACAATTTTTGGCTGATATAGGGATCAAA 35498

Lsal_LAC008020 35447 TCCCTGTATGATGTATTTTAGTACAATTTTTGGCTGATATAGGGATCAAA 35496

Lsat_Salinas 35501 TGGTATAGTTCATTTGTTGGTAGCTTGGAGGATTAAAAGCATGACTCTTG 35550

Lsat_WENDEL 35501 TGGTATAGTTCATTTGTTGGTAGCTTGGAGGATTAAAAGCATGACTCTTG 35550

Lser_US96UC23 35501 TGGTATAGTTCATTTGTTGGTAGCTTGGAGGATTAAAAGCATGACTCTTG 35550

Lser_LAC005780 35501 TGGTATAGTTCATTTGTTGGTAGCTTGGAGGATTAAAAGCATGACTCTTG 35550

Lvir_CGN013357 35499 TGGTATAGTTCATTTGTTGGTAGCTTGGAGGATTAAAAGCATGACTCTTG 35548

Lsal_LAC008020 35497 TGGTATAGTTCATTTGTTGGTAGCTTGGAGGATTAAAAGCATGACTCTTG 35546

Lsat_Salinas 35551 CTTTCCAATTGGCTGTTTTTGCATTAATTGCTACTTCATCAATCTTATTG 35600

Lsat_WENDEL 35551 CTTTCCAATTGGCTGTTTTTGCATTAATTGCTACTTCATCAATCTTATTG 35600

Lser_US96UC23 35551 CTTTCCAATTGGCTGTTTTTGCATTAATTGCTACTTCATCAATCTTATTG 35600

Lser_LAC005780 35551 CTTTCCAATTGGCTGTTTTTGCATTAATTGCTACTTCATCAATCTTATTG 35600

Lvir_CGN013357 35549 CTTTCCAATTGGCTGTTTTTGCATTAATTGCTACTTCATCAATCTTATTG 35598

Lsal_LAC008020 35547 CTTTCCAATTGGCTGTTTTTGCATTAATTGCTACTTCATCAATCTTATTG 35596

Lsat_Salinas 35601 ATTAGCGTACCCGTTGTATTTGCTTCTCCTGATGGTTGGTCAAGTAACAA 35650

Lsat_WENDEL 35601 ATTAGCGTACCCGTTGTATTTGCTTCTCCTGATGGTTGGTCAAGTAACAA 35650

Lser_US96UC23 35601 ATTAGCGTACCCGTTGTATTTGCTTCTCCTGATGGTTGGTCAAGTAACAA 35650

Lser_LAC005780 35601 ATTAGCGTACCCGTTGTATTTGCTTCTCCTGATGGTTGGTCAAGTAACAA 35650

Lvir_CGN013357 35599 ATTAGCGTACCCGTTGTATTTGCTTCTCCTGATGGTTGGTCAAGTAACAA 35648

Lsal_LAC008020 35597 ATTAGCGTACCCGTTGTATTTGCTTCTCCTGATGGTTGGTCAAGTAACAA 35646

Lsat_Salinas 35651 AAATGTTGTATTTTCAGGTACATCATTATGGATTGGATTAGTCTTTCTGG 35700

Lsat_WENDEL 35651 AAATGTTGTATTTTCAGGTACATCATTATGGATTGGATTAGTCTTTCTGG 35700

Lser_US96UC23 35651 AAATGTTGTATTTTCAGGTACATCATTATGGATTGGATTAGTCTTTCTGG 35700

Lser_LAC005780 35651 AAATGTTGTATTTTCAGGTACATCATTATGGATTGGATTAGTCTTTCTGG 35700

Lvir_CGN013357 35649 AAATGTTGTATTTTCAGGTACATCATTATGGATTGGATTAGTCTTTCTGG 35698

Lsal_LAC008020 35647 AAATGTTGTATTTTCAGGTACATCATTATGGATTGGATTAGTCTTTCTGG 35696

Lsat_Salinas 35701 TGGGTATCCTTAATTCTCTCATCTCTTGAACCTATTGTTCTAGATCCAAA 35750

Lsat_WENDEL 35701 TGGGTATCCTTAATTCTCTCATCTCTTGAACCTATTGTTCTAGATCCAAA 35750

Lser_US96UC23 35701 TGGGTATCCTTAATTCTCTCATCTCTTGAACCTATTGTTCTAGATCCAAA 35750

Lser_LAC005780 35701 TGGGTATCCTTAATTCTCTCATCTCTTGAACCTATTGTTCTAGATCCAAA 35750

Lvir_CGN013357 35699 TGGGTATCCTTAATTCTCTCATCTCTTGAACCTATTGTTCTAGATCCAAA 35748

Lsal_LAC008020 35697 TGGGTATCCTTAATTCTCTCATCTCTTGAACCTATTGTTCTAGATCCAAA 35746

Lsat_Salinas 35751 ACCAAAATGACCCCCTGAATTATTCTTGGTTGTGAGACGCATTCAATATA 35800

Lsat_WENDEL 35751 ACCAAAATGACCCCCTGAATTATTCTTGGTTGTGAGACGCATTCAATATA 35800

Lser_US96UC23 35751 ACCAAAATGACCCCCTGAATTATTCTTGGTTGTGAGACGCATTCAATATA 35800

Lser_LAC005780 35751 ACCAAAATGACCCCCTGAATTATTCTTGGTTGTGAGACGCATTCAATATA 35800

Lvir_CGN013357 35749 ACCGAAATGACCCCCTGAATTATTCTTGGTTGTGAGATGCATTCAATATA 35798

Lsal_LAC008020 35747 ACCGAAATGACCCCCTGAATTATTCTTGGTTGTGAGACGCATTCAATATA 35796

Lsat_Salinas 35801 AGTCCCCAAAATCCAAATAAATATAAGAAAAAAATGAAAAATTAGAAGGG 35850

Lsat_WENDEL 35801 AGTCCCCAAAATCCAAATAAATATAAGAAAAAAATGAAAAATTAGAAGGG 35850

Lser_US96UC23 35801 AGTCCCCAAAATCCAAATAAATATAAGAAAAAAATGAAAAATTAGAAGGG 35850

Lser_LAC005780 35801 AGTCCCCAAAATCCAAATAAATATAAGAAAAAAATGAAAAATTAGAAGGG 35850

Lvir_CGN013357 35799 AGTCCCCAAAATCCAAATAAATATAAGAAAAAAATGAAAAATTAGAAGGG 35848

Lsal_LAC008020 35797 AGTCCCCAAAATCCAAATAAATATAAGAAAAAAATGAAAAATTAGAAGGG 35846

Lsat_Salinas 35851 GGTCAAACTTCTTGTTCTTGAATGAAAAAAAAGGAATATAAAATTCAAAT 35900

Lsat_WENDEL 35851 GGTCAAACTTCTTGTTCTTGAATGAAAAAAAAGGAATATAAAATTCAAAT 35900

Lser_US96UC23 35851 GGTCAAACTTCTTGTTCTTGAATGAAAAAAAAGGAATATAAAATTCAAAT 35900

Lser_LAC005780 35851 GGTCAAACTTCTTGTTCTTGAATGAAAAAAAAGGAATATAAAATTCAAAT 35900

Lvir_CGN013357 35849 GGTCAAACTTCTTGTTCTTGAATGAAAAAAAAGGAATATAAAATTCAAAT 35898

Lsal_LAC008020 35847 GGTCAAACTTCTTGTTCTTGAATGAAAAAAAAGGAATATAAAATTCAAAT 35896

Lsat_Salinas 35901 TAATAAAAAATTTGGGCTCAATCTGAAGAGAGTCTCCGGCCCAGCACTGC 35950

Lsat_WENDEL 35901 TAATAAAAAATTTGGGCTCAATCTGAAGAGAGTCTCCGGCCCAGCACTGC 35950

Lser_US96UC23 35901 TAATAAAAAATTTGGGCTCAATCTGAAGAGAGTCTCCGGCCCAGCACTGC 35950

Lser_LAC005780 35901 TAATAAAAAATTTGGGCTCAATCTGAAGAGAGTCTCCGGCCCAGCACTGC 35950

Lvir_CGN013357 35899 TAATAAAAAATTTGGGCTCAATCTGAAGAGAGTCTCCGGCCCAGCACTGC 35948

Lsal_LAC008020 35897 TAATAAAAAATTTGGGCTCAATCTGAAGAGAGTCTCCGGCCCAGCACTGC 35946

Lsat_Salinas 35951 ACAAATATGCTCCAGACATATATATCATATATGTGTGGACATATTGTGTA 36000

Lsat_WENDEL 35951 ACAAATATGCTCCAGACATATATATCATATATGTGTGGACATATTGTGTA 36000

Lser_US96UC23 35951 ACAAATATGCTCCAGACATATATATCATATATGTGTGGACATATTGTGTA 36000

Lser_LAC005780 35951 ACAAATATGCTCCAGACATATATATCATATATGTGTGGACATATTGTGTA 36000

Lvir_CGN013357 35949 ACAAATATGCTCCAGACATATATATCATATATGTGTGGACATATTGTGTA 35998

Lsal_LAC008020 35947 ACAAATATGCTCCAGACATATATATCATATATGTGTGGACATATTGTGTA 35996

Lsat_Salinas 36001 TTATGGACATATTGTGTATCAAGAACGAAACAAAAAGCGGATATAGTCGA 36050

Lsat_WENDEL 36001 TTATGGACATATTGTGTATCAAGAACGAAACAAAAAGCGGATATAGTCGA 36050

Lser_US96UC23 36001 TTATGGACATATTGTGTATCAAGAACGAAACAAAAAGCGGATATAGTCGA 36050

Lser_LAC005780 36001 TTATGGACATATTGTGTATCAAGAACGAAACAAAAAGCGGATATAGTCGA 36050

Lvir_CGN013357 35999 TTATGGACATATTGTGTATCAAGAACGAAACAAAAAGCGGATATAGTCGA 36048

Lsal_LAC008020 35997 TTATGGACATATTGTGTATCAAGAACGAAACAAAAAGCGGATATAGTCGA 36046

Lsat_Salinas 36051 ATGGTAAAATTTCTCTTTGCCAAGGAGAAGACGCGGGTTCGATTCCCGCT 36100

Lsat_WENDEL 36051 ATGGTAAAATTTCTCTTTGCCAAGGAGAAGACGCGGGTTCGATTCCCGCT 36100

Lser_US96UC23 36051 ATGGTAAAATTTCTCTTTGCCAAGGAGAAGACGCGGGTTCGATTCCCGCT 36100

Lser_LAC005780 36051 ATGGTAAAATTTCTCTTTGCCAAGGAGAAGACGCGGGTTCGATTCCCGCT 36100

Lvir_CGN013357 36049 ATGGTAAAATTTCTCTTTGCCAAGGAGAAGACGCGGGTTCGATTCCCGCT 36098

Lsal_LAC008020 36047 ATGGTAAAATTTCTCTTTGCCAAGGAGAAGACGCGGGTTCGATTCCCGCT 36096

Lsat_Salinas 36101 ATCCGCCCAAAATGAAGTCATTTATTTAATATCTAATAATATCTAATATC 36150

Lsat_WENDEL 36101 ATCCGCCCAAAATGAAGTCATTTATTTAATATCTAATAATATCTAATATC 36150

Lser_US96UC23 36101 ATCCGCCCAAAATGAAGTCATTTATTTAATATCTAATAATATCTAATATC 36150

Lser_LAC005780 36101 ATCCGCCCAAAATGAAGTCATTTATTTAATATCTAATAATATCTAATATC 36150

Lvir_CGN013357 36099 ATCCGCCCAAAATGAAGTCATTTATTTAATATCTAATAATATCTAATATC 36148

Lsal_LAC008020 36097 ATCCGCCCAAAATGAAGTCATTTATTTAATATCTAATAATATCTAATATC 36146

Lsat_Salinas 36151 ATAAAATGTAAAATGCGAAAGGTATAGTTAGCCTTCATAGTGTAGTGATA 36200

Lsat_WENDEL 36151 ATAAAATGTAAAATGCGAAAGGTATAGTTAGCCTTCATAGTGTAGTGATA 36200

Lser_US96UC23 36151 ATAAAATGTAAAATGCGAAAGGTATAGTTAGCCTTCATAGTGTAGTGATA 36200

Lser_LAC005780 36151 ATAAAATGTAAAATGCGAAAGGTATAGTTAGCCTTCATAGTGTAGTGATA 36200

Lvir_CGN013357 36149 ATAAAATGTAAAATGCGAAAGGTATAGTTAGCCTTCATAGTGTAGTGATA 36198

Lsal_LAC008020 36147 ATAAAATGTAAAATGCGAAAGGTATAGTTAGCCTTCATAGTGTAGTGATA 36196

Lsat_Salinas 36201 GTGTAGTGATTCTATCCTTCCCCTTTTTGCCTTCTACCCCAAAACAAAAA 36250

Lsat_WENDEL 36201 GTGTAGTGATTCTATCCTTCCCCTTTTTGCCTTCTACCCCAAAACAAAAA 36250

Lser_US96UC23 36201 GTGTAGTGATTCTATCCTTCCCCTTTTTGCCTTCTACCCCAAAACAAAAA 36250

Lser_LAC005780 36201 GTGTAGTGATTCTATCCTTCCCCTTTTTGCCTTCTACCCCAAAACAAAAA 36250

Lvir_CGN013357 36199 GTGTAGTGATTCTATCCTTCCCCTTTTTGCCTTCTACCCCAAAACAAAAA 36248

Lsal_LAC008020 36197 GTGTAGTGATTCTATCCTTCCCCTTTTTGCCTTCTACCCCAAAACAAAAA 36246

Lsat_Salinas 36251 GTGTTAACTAATTACTAGTTACCAGAGTAAAACATACAATTTTTTTGACA 36300

Lsat_WENDEL 36251 GTGTTAACTAATTACTAGTTACCAGAGTAAAACATACAATTTTTTTGACA 36300

Lser_US96UC23 36251 GTGTTAACTAATTACTAGTTACCAGAGTAAAACATACAATTTTTTTGACA 36300

Lser_LAC005780 36251 GTGTTAACTAATTACTAGTTACCAGAGTAAAACATACAATTTTTTTGACA 36300

Lvir_CGN013357 36249 GTGTTAACTAATTACTAGTTACCAGAGTAAAACATACAATTTTTTTGACA 36298

Lsal_LAC008020 36247 GTGTTAACTAATTACTAGTTACCAGAGTAAAACATACAATTTTTTTGACA 36296

Lsat_Salinas 36301 AAAAGTATTGCGGAGACAGGATTTGAACCCGTGACCTCAAGGTTATGAGC 36350

Lsat_WENDEL 36301 AAAAGTATTGCGGAGACAGGATTTGAACCCGTGACCTCAAGGTTATGAGC 36350

Lser_US96UC23 36301 AAAAGTATTGCGGAGACAGGATTTGAACCCGTGACCTCAAGGTTATGAGC 36350

Lser_LAC005780 36301 AAAAGTATTGCGGAGACAGGATTTGAACCCGTGACCTCAAGGTTATGAGC 36350

Lvir_CGN013357 36299 AAAAGTATTGCGGAGACAGGATTTGAACCCGTGACCTCAAGGTTATGAGC 36348

Lsal_LAC008020 36297 AAAAGTATTGCGGAGACAGGATTTGAACCCGTGACCTCAAGGTTATGAGC 36346

Lsat_Salinas 36351 CTTGCGAGCTACCAAACTGCTCTACCCCGCGCCGAAGAGAAGAACTGAAA 36400

Lsat_WENDEL 36351 CTTGCGAGCTACCAAACTGCTCTACCCCGCGCCGAAGAGAAGAACTGAAA 36400

Lser_US96UC23 36351 CTTGCGAGCTACCAAACTGCTCTACCCCGCGCCGAAGAGAAGAACTGAAA 36400

Lser_LAC005780 36351 CTTGCGAGCTACCAAACTGCTCTACCCCGCGCCGAAGAGAAGAACTGAAA 36400

Lvir_CGN013357 36349 CTTGCGAGCTACCAAACTGCTCTACCCCGCGCCGAAGAGAAGAACTGAAA 36398

Lsal_LAC008020 36347 CTTGCGAGCTACCAAACTGCTCTACCCCGCGCCGAAGAGAAGAACTGAAA 36396

Lsat_Salinas 36401 ACTAATAGACAAGCAAGGATTGAATGCGCCCCTCTACCATATCTGTACAA 36450

Lsat_WENDEL 36401 ACTAATAGACAAGCAAGGATTGAATGCGCCCCTCTACCATATCTGTACAA 36450

Lser_US96UC23 36401 ACTAATAGACAAGCAAGGATTGAATGCGCCCCTCTACCATATCTGTACAA 36450

Lser_LAC005780 36401 ACTAATAGACAAGCAAGGATTGAATGCGCCCCTCTACCATATCTGTACAA 36450

Lvir_CGN013357 36399 ACTAATAGACAAGCAAGGATTGAATGCGCCCCTCTACCATATCTGTACAA 36448

Lsal_LAC008020 36397 ACTAATAGACAAGCAAGGATTGAATGCGCCCCTCTACCATATCTGTACAA 36446

Lsat_Salinas 36451 ATAGAATAGCCTATTTATACAGAATGGTAAAGAGGCCGTCTATGATCATC 36500

Lsat_WENDEL 36451 ATAGAATAGCCTATTTATACAGAATGGTAAAGAGGCCGTCTATGATCATC 36500

Lser_US96UC23 36451 ATAGAATAGCCTATTTATACAGAATGGTAAAGAGGCCGTCTATGATCATC 36500

Lser_LAC005780 36451 ATAGAATAGCCTATTTATACAGAATGGTAAAGAGGCCGTCTATGATCATC 36500

Lvir_CGN013357 36449 ATAGAATAGCCTATTTATACAGAATGGTAAAGAGGCCGTCTATGATCATC 36498

Lsal_LAC008020 36447 ATAGAATAGCCTATTTATACAGAATGGTAAAGAGGCCGTCTATGATCATC 36496

Lsat_Salinas 36501 GATCATAGAAATGAAATAAAGAGATATTTTAATCCTTACCAACTCGATCT 36550

Lsat_WENDEL 36501 GATCATAGAAATGAAATAAAGAGATATTTTAATCCTTACCAACTCGATCT 36550

Lser_US96UC23 36501 GATCATAGAAATGAAATAAAGAGATATTTTAATCCTTACCAACTCGATCT 36550

Lser_LAC005780 36501 GATCATAGAAATGAAATAAAGAGATATTTTAATCCTTACCAACTCGATCT 36550

Lvir_CGN013357 36499 GATCATAGAAATGAAATAAAGAGATATTTTAATCCTTACCAACTCGATCT 36548

Lsal_LAC008020 36497 GATCATAGAAATGAAATAAAGAGATATTTTAATCCTTACCAACTCGATCT 36546

Lsat_Salinas 36551 TGTCGCCCCTGGCAACAAACATGCATGAACCATTTCACGAAGTATGTGTC 36600

Lsat_WENDEL 36551 TGTCGCCCCTGGCAACAAACATGCATGAACCATTTCACGAAGTATGTGTC 36600

Lser_US96UC23 36551 TGTCGCCCCTGGCAACAAACATGCATGAACCATTTCACGAAGTATGTGTC 36600

Lser_LAC005780 36551 TGTCGCCCCTGGCAACAAACATGCATGAACCATTTCACGAAGTATGTGTC 36600

Lvir_CGN013357 36549 TGTCGCCCCTGGCAACAAACATGCATGAACCATTTCACGAAGTATGTGTC 36598

Lsal_LAC008020 36547 TGTCGCCCCTGGCAACAAACATGCATGAACCATTTCACGAAGTATGTGTC 36596

Lsat_Salinas 36601 CGGATAGTCCAAAGTCTCGATAGTTAGCTCTTGGCCTTCCAGTTGAAAAA 36650

Lsat_WENDEL 36601 CGGATAGTCCAAAGTCTCGATAGTTAGCTCTTGGCCTTCCAGTTGAAAAA 36650

Lser_US96UC23 36601 CGGATAGTCCAAAGTCTCGATAGTTAGCTCTTGGCCTTCCAGTTGAAAAA 36650

Lser_LAC005780 36601 CGGATAGTCCAAAGTCTCGATAGTTAGCTCTTGGCCTTCCAGTTGAAAAA 36650

Lvir_CGN013357 36599 CGGATAGTCCAAAGTCTCGATAGTTAGCTCTTGGCCTTCCAGTTGAAAAA 36648

Lsal_LAC008020 36597 CGGATAGTCCAAAGTCTCGATAGTTAGCTCTTGGCCTTCCAGTTGAAAAA 36646

Lsat_Salinas 36651 CAACGTCGATGAAGGCGTGTAGGCGCGCTATTCCGCGGTGGGGATTGTAA 36700

Lsat_WENDEL 36651 CAACGTCGATGAAGGCGTGTAGGCGCGCTATTCCGCGGTGGGGATTGTAA 36700

Lser_US96UC23 36651 CAACGTCGATGAAGGCGTGTAGGCGCGCTATTCCGCGGTGGGGATTGTAA 36700

Lser_LAC005780 36651 CAACGTCGATGAAGGCGTGTAGGCGCGCTATTCCGCGGTGGGGATTGTAA 36700

Lvir_CGN013357 36649 CAACGTCGATGAAGGCGTGTAGGCGCACTATTCCGCGGTGGGGATTGTAA 36698

Lsal_LAC008020 36647 CAACGTCGATGAAGGCGTGTAGGCGCACTATTCCGCGGTGGGGATTGTAA 36696

Lsat_Salinas 36701 CTTTCCATAAATTTCCCATTTGTCACTCAACGATCGAACTTTGCTTATTT 36750

Lsat_WENDEL 36701 CTTTCCATAAATTTCCCATTTGTCACTCAACGATCGAACTTTGCTTATTT 36750

Lser_US96UC23 36701 CTTTCCATAAATTTCCCATTTGTCACTCAACGATCGAACTTTGCTTATTT 36750

Lser_LAC005780 36701 CTTTCCATAAATTTCCCATTTGTCACTCAACGATCGAACTTTGCTTATTT 36750

Lvir_CGN013357 36699 CTTTCCATAAATTTCCCATTTGTCACTCAACGATCGAACTTTGCTTATTT 36748

Lsal_LAC008020 36697 CTTTCCATAAATTTCCCATTTGTCACTCAACGATCGAACTTTGCTTATTT 36746

Lsat_Salinas 36751 CTTTTTTTGAGGATCGACGAATCAAATGATATTTCTGTTCCAATTTTTGC 36800

Lsat_WENDEL 36751 CTTTTTTTGAGGATCGACGAATCAAATGATATTTCTGTTCCAATTTTTGC 36800

Lser_US96UC23 36751 CTTTTTTTGAGGATCGACGAATCAAATGATATTTCTGTTCCAATTTTTGC 36800

Lser_LAC005780 36751 CTTTTTTTGAGGATCGACGAATCAAATGATATTTCTGTTCCAATTTTTGC 36800

Lvir_CGN013357 36749 CTTTTTTTGAGGATCGACGAATCAAATGATATTTCTGTTCCAATTTTTGC 36798

Lsal_LAC008020 36747 CTTTTTTTGAGGATCGACGAATCAAATGATATTTCTGTTCCAATTTTTGC 36796

Lsat_Salinas 36801 CTCTTCTTTTCCCTCTGAATCAAACTTTTTTTTGCCATACTCATTAAGTT 36850

Lsat_WENDEL 36801 CTCTTCTTTTCCCTCTGAATCAAACTTTTTTTTGCCATACTCATTAAGTT 36850

Lser_US96UC23 36801 CTCTTCTTTTCCCTCTGAATCAAACTTTTTTTTGCCATACTCATTAAGTT 36850

Lser_LAC005780 36801 CTCTTCTTTTCCCTCTGAATCAAACTTTTTTTTGCCATACTCATTAAGTT 36850

Lvir_CGN013357 36799 CTCTTCTTTTCCCTCTGAATCAAACTTTTTTTTGCCATACTCATTAAGTT 36848

Lsal_LAC008020 36797 CTCTTCTTTTCCCTCTGAATCAAACTTTTTTTTGCCATACTCATTAAGTT 36846

Lsat_Salinas 36851 CCTATTAGTATCCATGATACAAGTCGGATCCTAGATGTAGAAATATAAAA 36900

Lsat_WENDEL 36851 CCTATTAGTATCCATGATACAAGTCGGATCCTAGATGTAGAAATATAAAA 36900

Lser_US96UC23 36851 CCTATTAGTATCCATGATACAAGTCGGATCCTAGATGTAGAAATATAAAA 36900

Lser_LAC005780 36851 CCTATTAGTATCCATGATACAAGTCGGATCCTAGATGTAGAAATATAAAA 36900

Lvir_CGN013357 36849 CCTATTAGTATCCATGATACAAGTCGGATCCTAGATGTAGAAATATAAAA 36898

Lsal_LAC008020 36847 CCTATTAGTATCCATGATACAAGTCGGATCCTAGATGTAGAAATATAAAA 36896

Lsat_Salinas 36901 TAAGAAGGTGGATCTCTTCTTCATCGAAAGAAATGAGATTATCGCGGATA 36950

Lsat_WENDEL 36901 TAAGAAGGTGGATCTCTTCTTCATCGAAAGAAATGAGATTATCGCGGATA 36950

Lser_US96UC23 36901 TAAGAAGGTGGATCTCTTCTTCATCGAAAGAAATGAGATTATCGCGGATA 36950

Lser_LAC005780 36901 TAAGAAGGTGGATCTCTTCTTCATCGAAAGAAATGAGATTATCGCGGATA 36950

Lvir_CGN013357 36899 TAAGAAGGTGGATCTCTTCTTCATCGAAAGAAATGAGATTATCGCGGATA 36948

Lsal_LAC008020 36897 TAAGAAGGTGGATCTCTTCTTCATCGAAAGAAATGAGATTATCGCGGATA 36946

Lsat_Salinas 36951 CAAAACATTCAATTAACAAAATTAACCAAATTTCCCTGATGTAGAGGCAA 37000

Lsat_WENDEL 36951 CAAAACATTCAATTAACAAAATTAACCAAATTTCCCTGATGTAGAGGCAA 37000

Lser_US96UC23 36951 CAAAACATTCAATTAACAAAATTAACCAAATTTCCCTGATGTAGAGGCAA 37000

Lser_LAC005780 36951 CAAAACATTCAATTAACAAAATTAACCAAATTTCCCTGATGTAGAGGCAA 37000

Lvir_CGN013357 36949 CAAAACATTCAATTAACAAAATTAACCAAATTTCCCTGATGTAGAGGCAA 36998

Lsal_LAC008020 36947 CAAAACATTCAATTAACAAAATTAACCAAATTTCCCTGATGTAGAGGCAA 36996

Lsat_Salinas 37001 TCAAGAAAGCTGCATAAGTAAATATATAACCTACAGAAAAGTGGGCTAAT 37050

Lsat_WENDEL 37001 TCAAGAAAGCTGCATAAGTAAATATATAACCTACAGAAAAGTGGGCTAAT 37050

Lser_US96UC23 37001 TCAAGAAAGCTGCATAAGTAAATATATAACCTACAGAAAAGTGGGCTAAT 37050

Lser_LAC005780 37001 TCAAGAAAGCTGCATAAGTAAATATATAACCTACAGAAAAGTGGGCTAAT 37050

Lvir_CGN013357 36999 TCAAGAAAGCTGCATAAGTAAATATATAACCTACAGAAAAGTGGGCTAAT 37048

Lsal_LAC008020 36997 TCAAGAAAGCTGCATAAGTAAATATATAACCTACAGAAAAGTGGGCTAAT 37046

Lsat_Salinas 37051 CCAACCAATCTTGCTTGTACAATGGAAAGTGCCACCGGTTTATCTCTCCA 37100

Lsat_WENDEL 37051 CCAACCAATCTTGCTTGTACAATGGAAAGTGCCACCGGTTTATCTCTCCA 37100

Lser_US96UC23 37051 CCAACCAATCTTGCTTGTACAATGGAAAGTGCCACCGGTTTATCTCTCCA 37100

Lser_LAC005780 37051 CCAACCAATCTTGCTTGTACAATGGAAAGTGCCACCGGTTTATCTCTCCA 37100

Lvir_CGN013357 37049 CCAACCAATCTTGCTTGTACAATGGAAAGTGCCACCGGTTTATCTCTCCA 37098

Lsal_LAC008020 37047 CCAACCAATCTTGCTTGTACAATGGAAAGTGCCACCGGTTTATCTCTCCA 37096

Lsat_Salinas 37101 ACGAATCAAATTGGCCAAAGGTGTGCGTTCATGAGCCCATGCTAAAGTTT 37150

Lsat_WENDEL 37101 ACGAATCAAATTGGCCAAAGGTGTGCGTTCATGAGCCCATGCTAAAGTTT 37150

Lser_US96UC23 37101 ACGAATCAAATTGGCCAAAGGTGTGCGTTCATGAGCCCATGCTAAAGTTT 37150

Lser_LAC005780 37101 ACGAATCAAATTGGCCAAAGGTGTGCGTTCATGAGCCCATGCTAAAGTTT 37150

Lvir_CGN013357 37099 ACGAATCAAATTGGCCAAAGGTGTGCGTTCATGAGCCCATGCTAAAGTTT 37148

Lsal_LAC008020 37097 ACGAATCAAATTGGCCAAAGGTGTGCGTTCATGAGCCCATGCTAAAGTTT 37146

Lsat_Salinas 37151 CAATCAATTCCTGCCAATATCCACGCCAGGAAATTAAAAACATAAATCCA 37200

Lsat_WENDEL 37151 CAATCAATTCCTGCCAATATCCACGCCAGGAAATTAAAAACATAAATCCA 37200

Lser_US96UC23 37151 CAATCAATTCCTGCCAATATCCACGCCAGGAAATTAAAAACATAAATCCA 37200

Lser_LAC005780 37151 CAATCAATTCCTGCCAATATCCACGCCAGGAAATTAAAAACATAAATCCA 37200

Lvir_CGN013357 37149 CAATCAATTCCTGCCAATATCCACGCCAGGAAATTAAAAACATAAATCCA 37198

Lsal_LAC008020 37147 CAATCAATTCCTGCCAATATCCACGCCAGGAAATTAAAAACATAAATCCA 37196

Lsat_Salinas 37201 GTAGCCCAAACCAGATGTCCAAATAAAAACATCCATGCCCAGACTGATAA 37250

Lsat_WENDEL 37201 GTAGCCCAAACCAGATGTCCAAATAAAAACATCCATGCCCAGACTGATAA 37250

Lser_US96UC23 37201 GTAGCCCAAACCAGATGTCCAAATAAAAACATCCATGCCCAGACTGATAA 37250

Lser_LAC005780 37201 GTAGCCCAAACCAGATGTCCAAATAAAAACATCCATGCCCAGACTGATAA 37250

Lvir_CGN013357 37199 GTAGCCCAAACCAGATGTCCAAATAAAAACATCCATGCCCAGACTGATAA 37248

Lsal_LAC008020 37197 GTAGCCCAAACCAGATGTCCAAATAAAAACATCCATGCCCAGACTGATAA 37246

Lsat_Salinas 37251 ACTATTCATACCAAAAGGGTTATATCCATTAATAAGTTGTGAAGAGTTTA 37300

Lsat_WENDEL 37251 ACTATTCATACCAAAAGGGTTATATCCATTAATAAGTTGTGAAGAGTTTA 37300

Lser_US96UC23 37251 ACTATTCATACCAAAAGGGTTATATCCATTAATAAGTTGTGAAGAGTTTA 37300

Lser_LAC005780 37251 ACTATTCATACCAAAAGGGTTATATCCATTAATAAGTTGTGAAGAGTTTA 37300

Lvir_CGN013357 37249 ACTATTCATACCAAAAGGGTTATATCCATTAATAAGTTGTGAAGAGTTTA 37298

Lsal_LAC008020 37247 ACTATTCATACCAAAAGGGTTATATCCGTTAATAAGTTGTGAAGAGTTTA 37296

Lsat_Salinas 37301 ACCATAAATAATCTCTTAACCAGCCCATCAAATAAGTGGAAGATTCATTA 37350

Lsat_WENDEL 37301 ACCATAAATAATCTCTTAACCAGCCCATCAAATAAGTGGAAGATTCATTA 37350

Lser_US96UC23 37301 ACCATAAATAATCTCTTAACCAGCCCATCAAATAAGTGGAAGATTCATTA 37350

Lser_LAC005780 37301 ACCATAAATAATCTCTTAACCAGCCCATCAAATAAGTGGAAGATTCATTA 37350

Lvir_CGN013357 37299 ACCATAAATAATCTCTTAACCAGCCCATCAAATAAGTGGAAGATTCATTA 37348

Lsal_LAC008020 37297 ACCATAAATAATCTCTTAACCAGCCCATCAAATAAGTGGAAGATTCATTA 37346

Lsat_Salinas 37351 AACTGCGAAACATTACCCTGCCATAATGTAATGTGCTTCCAATGCCAATA 37400

Lsat_WENDEL 37351 AACTGCGAAACATTACCCTGCCATAATGTAATGTGCTTCCAATGCCAATA 37400

Lser_US96UC23 37351 AACTGCGAAACATTACCCTGCCATAATGTAATGTGCTTCCAATGCCAATA 37400

Lser_LAC005780 37351 AACTGCGAAACATTACCCTGCCATAATGTAATGTGCTTCCAATGCCAATA 37400

Lvir_CGN013357 37349 AACTGCGAAACATTACCCTGCCATAATGTAATGTGCTTCCAATGCCAATA 37398

Lsal_LAC008020 37347 AACTGCGAAACATTACCCTGCCATAATGTAATGTGCTTCCAATGCCAATA 37396

Lsat_Salinas 37401 AAAAGTAACCCATCCAATGGTATTTAACATCCAAAAAACTGCCAAATAAA 37450

Lsat_WENDEL 37401 AAAAGTAACCCATCCAATGGTATTTAACATCCAAAAAACTGCCAAATAAA 37450

Lser_US96UC23 37401 AAAAGTAACCCATCCAATGGTATTTAACATCCAAAAAACTGCCAAATAAA 37450

Lser_LAC005780 37401 AAAAGTAACCCATCCAATGGTATTTAACATCCAAAAAACTGCCAAATAAA 37450

Lvir_CGN013357 37399 AAAAGTAACCCATCCAATGGTATTTAACATCCAAAAAACTGCCAAATAAA 37448

Lsal_LAC008020 37397 AAAAGTAACCCATCCAATGGTATTTAACATCCAAAAAACTGCCAAATAAA 37446

Lsat_Salinas 37451 ATGCGTCCCAAGCCGAAATATCACAAGTACCGCCTCGTCCTGGGCCATCG 37500

Lsat_WENDEL 37451 ATGCGTCCCAAGCCGAAATATCACAAGTACCGCCTCGTCCTGGGCCATCG 37500

Lser_US96UC23 37451 ATGCGTCCCAAGCCGAAATATCACAAGTACCGCCTCGTCCTGGGCCATCG 37500

Lser_LAC005780 37451 ATGCGTCCCAAGCCGAAATATCACAAGTACCGCCTCGTCCTGGGCCATCG 37500

Lvir_CGN013357 37449 ATGCGTCCCAAGCCGAAATATCACAAGTACCGCCTCGTCCTGGGCCATCG 37498

Lsal_LAC008020 37447 ATGCGTCCCAAGCCGAAATATCACAAGTACCGCCTCGTCCTGGGCCATCG 37496

Lsat_Salinas 37501 CACGGAAAACTATAACCGAAATCCTTTTTATCTGGCATTAACTTGGAACC 37550

Lsat_WENDEL 37501 CACGGAAAACTATAACCGAAATCCTTTTTATCTGGCATTAACTTGGAACC 37550

Lser_US96UC23 37501 CACGGAAAACTATAACCGAAATCCTTTTTATCTGGCATTAACTTGGAACC 37550

Lser_LAC005780 37501 CACGGAAAACTATAACCGAAATCCTTTTTATCTGGCATTAACTTGGAACC 37550

Lvir_CGN013357 37499 CACGGAAAACTATAACCGAAATCCTTTTTATCTGGCATTAACTTGGAACC 37548

Lsal_LAC008020 37497 CACGGAAAACTATAACCGAAATCCTTTTTATCTGGCATTAACTTGGAACC 37546

Lsat_Salinas 37551 ACGCGCATCTAACGCACCTTTTACTAAGATCAACGTAGTTGTATGTAAAC 37600

Lsat_WENDEL 37551 ACGCGCATCTAACGCACCTTTTACTAAGATCAACGTAGTTGTATGTAAAC 37600

Lser_US96UC23 37551 ACGCGCATCTAACGCACCTTTTACTAAGATCAACGTAGTTGTATGTAAAC 37600

Lser_LAC005780 37551 ACGCGCATCTAACGCACCTTTTACTAAGATCAACGTAGTTGTATGTAAAC 37600

Lvir_CGN013357 37549 ACGTGCATCTAACGCACCTTTTACTAAGATCAACGTAGTTGTATGTAAAC 37598

Lsal_LAC008020 37547 ACGTGCATCTAACGCACCTTTTACTAAGATCAACGTAGTTGTATGTAAAC 37596

Lsat_Salinas 37601 CCAGAGCAATAGCATGATGAACCAAAAAGTCCCCAGGACCTATTGTTAAG 37650

Lsat_WENDEL 37601 CCAGAGCAATAGCATGATGAACCAAAAAGTCCCCAGGACCTATTGTTAAG 37650

Lser_US96UC23 37601 CCAGAGCAATAGCATGATGAACCAAAAAGTCCCCAGGACCTATTGTTAAG 37650

Lser_LAC005780 37601 CCAGAGCAATAGCATGATGAACCAAAAAGTCCCCAGGACCTATTGTTAAG 37650

Lvir_CGN013357 37599 CCAGAGCAATAGCATGATGAACCAAAAAGTCCCCAGGACCTATTGTTAAG 37648

Lsal_LAC008020 37597 CCAGAGCAATAGCATGATGAACCAAAAAGTCCCCAGGACCTATTGTTAAG 37646

Lsat_Salinas 37651 AATAGTGAATTACTATTCTCATTAATAGCATTTAACCAACCCGGCAACCA 37700

Lsat_WENDEL 37651 AATAGTGAATTACTATTCTCATTAATAGCATTTAACCAACCCGGCAACCA 37700

Lser_US96UC23 37651 AATAGTGAATTACTATTCTCATTAATAGCATTTAACCAACCCGGCAACCA 37700

Lser_LAC005780 37651 AATAGTGAATTACTATTCTCATTAATAGCATTTAACCAACCCGGCAACCA 37700

Lvir_CGN013357 37649 AATAGTGAATTACTATTCTCATTAATAGCATTTAACCAACCAGGCAACCA 37698

Lsal_LAC008020 37647 AATAGTGAATTACTATTCTCATTAATAGCATTTAACCAACCCGGCAACCA 37696

Lsat_Salinas 37701 TATGCTTCGACCCGCATTGAATGCCGGGCCATTCGTTGAAGATAAAAGTA 37750

Lsat_WENDEL 37701 TATGCTTCGACCCGCATTGAATGCCGGGCCATTCGTTGAAGATAAAAGTA 37750

Lser_US96UC23 37701 TATGCTTCGACCCGCATTGAATGCCGGGCCATTCGTTGAAGATAAAAGTA 37750

Lser_LAC005780 37701 TATGCTTCGACCCGCATTGAATGCCGGGCCATTCGTTGAAGATAAAAGTA 37750

Lvir_CGN013357 37699 TATGCTTCGACCCGCATTGAATGCCGGGCCATTCGTTGAAGATAAAAGTA 37748

Lsal_LAC008020 37697 TATGCTTCGACCCGCATTGAATGCCGGGCCATTCGTTGAAGATAAAAGTA 37746

Lsat_Salinas 37751 TATCGAACCCATATGAAGTTTTACCATGAGCAGATTGTATCCATTGAGCA 37800

Lsat_WENDEL 37751 TATCGAACCCATATGAAGTTTTACCATGAGCAGATTGTATCCATTGAGCA 37800

Lser_US96UC23 37751 TATCGAACCCATATGAAGTTTTACCATGAGCAGATTGTATCCATTGAGCA 37800

Lser_LAC005780 37751 TATCGAACCCATATGAAGTTTTACCATGAGCAGATTGTATCCATTGAGCA 37800

Lvir_CGN013357 37749 TATCGAACCCATATGAAGTTTTACCATGAGCAGATTGTATCCATTGAGCA 37798

Lsal_LAC008020 37747 TATCGAACCCATATGAAGTTTTACCATGAGCAGATTGTATCCATTGAGCA 37796

Lsat_Salinas 37801 AATATAGGTTCGATTAAGATTTGCTTCTCCGGAGTACCAAAGGCAAGCAT 37850

Lsat_WENDEL 37801 AATATAGGTTCGATTAAGATTTGCTTCTCCGGAGTACCAAAGGCAAGCAT 37850

Lser_US96UC23 37801 AATATAGGTTCGATTAAGATTTGCTTCTCCGGAGTACCAAAGGCAAGCAT 37850

Lser_LAC005780 37801 AATATAGGTTCGATTAAGATTTGCTTCTCCGGAGTACCAAAGGCAAGCAT 37850

Lvir_CGN013357 37799 AATATAGGTTCGATTAAGATTTGCTTCTCCGGAGTACCAAAGGCAAGCAT 37848

Lsal_LAC008020 37797 AATATAGGTTCGATTAAGATTTGCTTCTCCGGAGTACCAAAGGCAAGCAT 37846

Lsat_Salinas 37851 GACATCATTATGAACATAAAGTCCCAAGGTATGGAAACCCAAAAAGAGGC 37900

Lsat_WENDEL 37851 GACATCATTATGAACATAAAGTCCCAAGGTATGGAAACCCAAAAAGAGGC 37900

Lser_US96UC23 37851 GACATCATTATGAACATAAAGTCCCAAGGTATGGAAACCCAAAAAGAGGC 37900

Lser_LAC005780 37851 GACATCATTATGAACATAAAGTCCCAAGGTATGGAAACCCAAAAAGAGGC 37900

Lvir_CGN013357 37849 GACATCATTATGAACATAAAGTCCCAAGGTATGGAAACCCAAAAAGAGGC 37898

Lsal_LAC008020 37847 GACATCATTATGAACATAAAGTCCCAAGGTATGGAAACCCAAAAAGAGGC 37896

Lsat_Salinas 37901 TGGCCCAACTTAAATGAGATATGATAGCTTCTTTATGTTCTAACATTCGT 37950

Lsat_WENDEL 37901 TGGCCCAACTTAAATGAGATATGATAGCTTCTTTATGTTCTAACATTCGT 37950

Lser_US96UC23 37901 TGGCCCAACTTAAATGAGATATGATAGCTTCTTTATGTTCTAACATTCGT 37950

Lser_LAC005780 37901 TGGCCCAACTTAAATGAGATATGATAGCTTCTTTATGTTCTAACATTCGT 37950

Lvir_CGN013357 37899 TGGCCCAACTTAAATGAGATATGATAGCTTCTTTATGTTCTAACATTCGT 37948

Lsal_LAC008020 37897 TGGCCCAACTTAAATGAGATATGATAGCTTCTTTATGTTCTAACATTCGT 37946

Lsat_Salinas 37951 GCCAATACATTATCCTCATTCTGTTCCGGATTGTAATCCCTAATAAAAAA 38000

Lsat_WENDEL 37951 GCCAATACATTATCCTCATTCTGTTCCGGATTGTAATCCCTAATAAAAAA 38000

Lser_US96UC23 37951 GCCAATACATTATCCTCATTCTGTTCCGGATTGTAATCCCTAATAAAAAA 38000

Lser_LAC005780 37951 GCCAATACATTATCCTCATTCTGTTCCGGATTGTAATCCCTAATAAAAAA 38000

Lvir_CGN013357 37949 GCCAATACATTATCCTCATTCTGTTCCGGATTGTAATCCCTAATAAAAAA 37998

Lsal_LAC008020 37947 GCCAATACATTATCCTCATTCTGTTCCGGATTGTAATCCCTAATAAAAAA 37996

Lsat_Salinas 38001 TATAGCTCCATGAGCAAAAGCTCCTGTCATGATGAATCCTGCAATGTATT 38050

Lsat_WENDEL 38001 TATAGCTCCATGAGCAAAAGCTCCTGTCATGATGAATCCTGCAATGTATT 38050

Lser_US96UC23 38001 TATAGCTCCATGAGCAAAAGCTCCTGTCATGATGAATCCTGCAATGTATT 38050

Lser_LAC005780 38001 TATAGCTCCATGAGCAAAAGCTCCTGTCATGATGAATCCTGCAATGTATT 38050

Lvir_CGN013357 37999 TATAGCTCCATGAGCAAAAGCTCCTGTCATGATGAATCCTGCAATGTATT 38048

Lsal_LAC008020 37997 TATAGCTCCATGAGCAAAAGCTCCTGTCATGATGAATCCTGCAATGTATT 38046

Lsat_Salinas 38051 GGTGATGAGTATATAATGCAGCTTGAGTAGTAAAGTCTTGTGCTATAAAT 38100

Lsat_WENDEL 38051 GGTGATGAGTATATAATGCAGCTTGAGTAGTAAAGTCTTGTGCTATAAAT 38100

Lser_US96UC23 38051 GGTGATGAGTATATAATGCAGCTTGAGTAGTAAAGTCTTGTGCTATAAAT 38100

Lser_LAC005780 38051 GGTGATGAGTATATAATGCAGCTTGAGTAGTAAAGTCTTGTGCTATAAAT 38100

Lvir_CGN013357 38049 GGTGATGAGTATATAATGCAGCTTGAGTAGTAAAGTCTTGTGCTATAAAT 38098

Lsal_LAC008020 38047 GGTGATGAGTATATAATGCAGCTTGAGTAGTAAAGTCTTGTGCTATAAAT 38096

Lsat_Salinas 38101 GCATAAGCGGGTAAAGAGTACATGTGTTGAGCTACCAAAGAAGTAATAAC 38150

Lsat_WENDEL 38101 GCATAAGCGGGTAAAGAGTACATGTGTTGAGCTACCAAAGAAGTAATAAC 38150

Lser_US96UC23 38101 GCATAAGCGGGTAAAGAGTACATGTGTTGAGCTACCAAAGAAGTAATAAC 38150

Lser_LAC005780 38101 GCATAAGCGGGTAAAGAGTACATGTGTTGAGCTACCAAAGAAGTAATAAC 38150

Lvir_CGN013357 38099 GCATAAGCGGGTAAAGAGTACATGTGTTGAGCTACCAAAGAAGTAATAAC 38148

Lsal_LAC008020 38097 GCATAAGCGGGTAAAGAGTACATGTGTTGAGCTACCAAAGAAGTAATAAC 38146

Lsat_Salinas 38151 CCCTAAAGAAGCTAGAGCAAGGCCTAATTGAAAATGAAGCGAATTATTAA 38200

Lsat_WENDEL 38151 CCCTAAAGAAGCTAGAGCAAGGCCTAATTGAAAATGAAGCGAATTATTAA 38200

Lser_US96UC23 38151 CCCTAAAGAAGCTAGAGCAAGGCCTAATTGAAAATGAAGCGAATTATTAA 38200

Lser_LAC005780 38151 CCCTAAAGAAGCTAGAGCAAGGCCTAATTGAAAATGAAGCGAATTATTAA 38200

Lvir_CGN013357 38149 CCCTAAAGAAGCTAGAGCAAGGCCTAATTGAAAATGAAGCGAATTATTAA 38198

Lsal_LAC008020 38147 CCCTAAAGAAGCTAGAGCAAGGCCTAATTGAAAATGAAGCGAATTATTAA 38196

Lsat_Salinas 38201 TTGTGTCATAAAGGCCCTTATGCCCACGTCCCAATCGCCCTCCCGGAGGG 38250

Lsat_WENDEL 38201 TTGTGTCATAAAGGCCCTTATGCCCACGTCCCAATCGCCCTCCCGGAGGG 38250

Lser_US96UC23 38201 TTGTGTCATAAAGGCCCTTATGCCCACGTCCCAATCGCCCTCCCGGAGGG 38250

Lser_LAC005780 38201 TTGTGTCATAAAGGCCCTTATGCCCACGTCCCAATCGCCCTCCCGGAGGG 38250

Lvir_CGN013357 38199 TTGTGTCATAAAGGCCCTTATGCCCACGTCCCAATCGCCCTCCCGGAGGG 38248

Lsal_LAC008020 38197 TTGTGTCATAAAGGCCCTTATGCCCACGTCCCAATCGCCCTCCCGGAGGG 38246

Lsat_Salinas 38251 ATATGTGCATCTAAAAGATCTTTCATACTGTGCCCAATCCCAAAATTAGT 38300

Lsat_WENDEL 38251 ATATGTGCATCTAAAAGATCTTTCATACTGTGCCCAATCCCAAAATTAGT 38300

Lser_US96UC23 38251 ATATGTGCATCTAAAAGATCTTTCATACTGTGCCCAATCCCAAAATTAGT 38300

Lser_LAC005780 38251 ATATGTGCATCTAAAAGATCTTTCATACTGTGCCCAATCCCAAAATTAGT 38300

Lvir_CGN013357 38249 ATATGTGCATCTAAAAGATCTTTCATACTGTGCCCAATCCCAAAATTAGT 38298

Lsal_LAC008020 38247 ATATGTGCATCTAAAAGATCTTTCATACTGTGCCCAATCCCAAAATTAGT 38296

Lsat_Salinas 38301 TCTATACATATGCCCAGCAATAAGAAAAAGAAATGCAATAGCTAAATGAT 38350

Lsat_WENDEL 38301 TCTATACATATGCCCAGCAATAAGAAAAAGAAATGCAATAGCTAAATGAT 38350

Lser_US96UC23 38301 TCTATACATATGCCCAGCAATAAGAAAAAGAAATGCAATAGCTAAATGAT 38350

Lser_LAC005780 38301 TCTATACATATGCCCAGCAATAAGAAAAAGAAATGCAATAGCTAAATGAT 38350

Lvir_CGN013357 38299 TCTATACATATGCCCAGCAATAAGAAAAAGAAATGCAATAGCTAAATGAT 38348

Lsal_LAC008020 38297 TCTATACATATGCCCAGCAATAAGAAAAAGAAATGCAATAGCTAAATGAT 38346

Lsat_Salinas 38351 GATGAGCCATATCAGTCAGCCATAAACTTTGGGTTTGTGGATGGAATCCC 38400

Lsat_WENDEL 38351 GATGAGCCATATCAGTCAGCCATAAACTTTGGGTTTGTGGATGGAATCCC 38400

Lser_US96UC23 38351 GATGAGCCATATCAGTCAGCCATAAACTTTGGGTTTGTGGATGGAATCCC 38400

Lser_LAC005780 38351 GATGAGCCATATCAGTCAGCCATAAACTTTGGGTTTGTGGATGGAATCCC 38400

Lvir_CGN013357 38349 GATGAGCCATATCAGTCAGCCATAAACTTTGGGTTTGTGGATGGAATCCC 38398

Lsal_LAC008020 38347 GATGAGCCATATCAGTCAGCCATAAACTTTGGGTTTGTGGATGGAATCCC 38396

Lsat_Salinas 38401 CCGAGAAGGGTTAAAATGGCAGTTCCTGCTCCTTGGGAGGTACCAAATAA 38450

Lsat_WENDEL 38401 CCGAGAAGGGTTAAAATGGCAGTTCCTGCTCCTTGGGAGGTACCAAATAA 38450

Lser_US96UC23 38401 CCGAGAAGGGTTAAAATGGCAGTTCCTGCTCCTTGGGAGGTACCAAATAA 38450

Lser_LAC005780 38401 CCGAGAAGGGTTAAAATGGCAGTTCCTGCTCCTTGGGAGGTACCAAATAA 38450

Lvir_CGN013357 38399 CCGAGAAGGGTTAAAATGGCAGTTCCTGCTCCTTGGGAGGTACCAAATAA 38448

Lsal_LAC008020 38397 CCGAGAAGGGTTAAAATGGCAGTTCCTGCTCCTTGGGAGGTACCAAATAA 38446

Lsat_Salinas 38451 ATGACTACCTGAATCGGGATTTTGAGCATAAAGATTCCACTGACCTGTAA 38500

Lsat_WENDEL 38451 ATGACTACCTGAATCGGGATTTTGAGCATAAAGATTCCACTGACCTGTAA 38500

Lser_US96UC23 38451 ATGACTACCTGAATCGGGATTTTGAGCATAAAGATTCCACTGACCTGTAA 38500

Lser_LAC005780 38451 ATGACTACCTGAATCGGGATTTTGAGCATAAAGATTCCACTGACCTGTAA 38500

Lvir_CGN013357 38449 ATGACTACCTGAATCGGGATTTTGAGCATAAAGATTCCACTGACCTGTAA 38498

Lsal_LAC008020 38447 ATGACTACCTGAATCGGGATTTTGAGCATAAAGATTCCACTGACCTGTAA 38496

Lsat_Salinas 38501 AAAGTGGGCCTAATCCTTGGGGATGTGGTAATACATCTAAGAAATTATTC 38550

Lsat_WENDEL 38501 AAAGTGGGCCTAATCCTTGGGGATGTGGTAATACATCTAAGAAATTATTC 38550

Lser_US96UC23 38501 AAAGTGGGCCTAATCCTTGGGGATGTGGTAATACATCTAAGAAATTATTC 38550

Lser_LAC005780 38501 AAAGTGGGCCTAACCCTTGGGGATGTGGTAATACATCTAAGAAATTATTC 38550

Lvir_CGN013357 38499 AAAGTGGGCCTAACCCTTGGGGATGTGGTAATACATCTAAGAAATTATTC 38548

Lsal_LAC008020 38497 AAAGTGGGCCTAACCCTTGGGGATGTGGTAATACATCTAAGAAATTATTC 38546

Lsat_Salinas 38551 CATCGAACGTACTCCCCTCTGGATGCAGGAATAGCGACATGGACTAAATG 38600

Lsat_WENDEL 38551 CATCGAACGTACTCCCCTCTGGATGCAGGAATAGCGACATGGACTAAATG 38600

Lser_US96UC23 38551 CATCGAACGTACTCCCCTCTGGATGCAGGAATAGCGACATGGACTAAATG 38600

Lser_LAC005780 38551 CATCGAACGTACTCCCCTCTGGATGCAGGAATAGCGACATGGACTAAATG 38600

Lvir_CGN013357 38549 CATCGAACGTACTCCCCTCTGGATGCAGGAATAGCGACATGGACTAAATG 38598

Lsal_LAC008020 38547 CATCGAACGTACTCCCCTCTGGATGCAGGAATAGCGACATGGACTAAATG 38596

Lsat_Salinas 38601 CCCTGTCCAAGCCAAGGAACTTACGCCGAAGAGTCCTGATAAATGATGAT 38650

Lsat_WENDEL 38601 CCCTGTCCAAGCCAAGGAACTTACGCCGAAGAGTCCTGATAAATGATGAT 38650

Lser_US96UC23 38601 CCCTGTCCAAGCCAAGGAACTTACGCCGAAGAGTCCTGATAAATGATGAT 38650

Lser_LAC005780 38601 CCCTGTCCAAGCCAAGGAACTTACGCCGAAGAGTCCTGATAAATGATGAT 38650

Lvir_CGN013357 38599 CCCTGTCCAAGCCAAGGAACTTACGCCGAAGAGTCCTGATAAATGATGAT 38648

Lsal_LAC008020 38597 CCCTGTCCAAGCCAAGGAACTTACGCCGAAGAGTCCTGATAAATGATGAT 38646

Lsat_Salinas 38651 TGAGACGAGATTCTGCATTTTTGAACCACGAAACACTCGGTTTCCATTTC 38700

Lsat_WENDEL 38651 TGAGACGAGATTCTGCATTTTTGAACCACGAAACACTCGGTTTCCATTTC 38700

Lser_US96UC23 38651 TGAGACGAGATTCTGCATTTTTGAACCACGAAACACTCGGTTTCCATTTC 38700

Lser_LAC005780 38651 TGAGACGAGATTCTGCATTTTTGAACCACGAAACACTCGGTTTCCATTTC 38700

Lvir_CGN013357 38649 TGAGACGAGATTCTGCATTTTTGAACCACGAAACACTCGGTTTCCATTTC 38698

Lsal_LAC008020 38647 TGAGACGAGATTCTGCATTTTTGAACCACGAAACACTCGGTTTCCATTTC 38696

Lsat_Salinas 38701 GGTTGTAGGTGTAACCAACCCGCTATTAAAGATATGGCAGAAATTAATAA 38750

Lsat_WENDEL 38701 GGTTGTAGGTGTAACCAACCCGCTATTAAAGATATGGCAGAAATTAATAA 38750

Lser_US96UC23 38701 GGTTGTAGGTGTAACCAACCCGCTATTAAAGATATGGCAGAAATTAATAA 38750

Lser_LAC005780 38701 GGTTGTAGGTGTAACCAACCCGCTATTAAAGATATGGCAGAAATTAATAA 38750

Lvir_CGN013357 38699 GGTTGTAGGTGTAACCAACCCGCTATTAAAGATATGGCAGAAATTAATAA 38748

Lsal_LAC008020 38697 GGTTGTAGGTGTAACCAACCCGCTATTAAAGATATGGCAGAAATTAATAA 38746

Lsat_Salinas 38751 TAGAAAAAGAGCTCCAGTATAAAGATCTTCATTAGTTCGTAAACCGATTG 38800

Lsat_WENDEL 38751 TAGAAAAAGAGCTCCAGTATAAAGATCTTCATTAGTTCGTAAACCGATTG 38800

Lser_US96UC23 38751 TAGAAAAAGAGCTCCAGTATAAAGATCTTCATTAGTTCGTAAACCGATTG 38800

Lser_LAC005780 38751 TAGAAAAAGAGCTCCAGTATAAAGATCTTCATTAGTTCGTAAACCGATTG 38800

Lvir_CGN013357 38749 TAGAAAAAGAGCTCCAGTATAAAGATCTTCATTAGTTCGTAAACCGATTG 38798

Lsal_LAC008020 38747 TAGAAAAAGAGCTCCAGTATAAAGATCTTCATTAGTTCGTAAACCGATTG 38796

Lsat_Salinas 38801 TATACCACCACTGGTAAACACCAGAATAGGCGATATTCACTGGGCCAAGA 38850

Lsat_WENDEL 38801 TATACCACCACTGGTAAACACCAGAATAGGCGATATTCACTGGGCCAAGA 38850

Lser_US96UC23 38801 TATACCACCACTGGTAAACACCAGAATAGGCGATATTCACTGGGCCAAGA 38850

Lser_LAC005780 38801 TATACCACCACTGATAAACACCAGAATAGGCGATATTCACTGGGCCAAGA 38850

Lvir_CGN013357 38799 TATACCACCACTGATAAACACCAGAATAGGCGATATTCACTGGGCCAAGA 38848

Lsal_LAC008020 38797 TATACCACCACTGATAAACACCAGAATAGGCGATATTCACTGGGCCAAGA 38846

Lsat_Salinas 38851 GCACCCCCTCGAGTAAAAGCTTCTACAGCCGGTTGACCAAAATGAGGATC 38900

Lsat_WENDEL 38851 GCACCCCCTCGAGTAAAAGCTTCTACAGCCGGTTGACCAAAATGAGGATC 38900

Lser_US96UC23 38851 GCACCCCCTCGAGTAAAAGCTTCTACAGCCGGTTGACCAAAATGAGGATC 38900

Lser_LAC005780 38851 GCACCCCCTCGAGTAAAAGCTTCTACAGCCGGTTGACCAAAATGAGGATC 38900

Lvir_CGN013357 38849 GCACCCCCTCGAGTAAAAGCTTCTACAGCCGGTTGACCAAAATGAGGATC 38898

Lsal_LAC008020 38847 GCACCCCCTCGAGTAAAAGCTTCTACAGCCGGTTGACCAAAATGAGGATC 38896

Lsat_Salinas 38901 CCAAATTGCATGAGCAATAGGTCTTACATGTAAAGGGTCCTGTACCCATG 38950

Lsat_WENDEL 38901 CCAAATTGCATGAGCAATAGGTCTTACATGTAAAGGGTCCTGTACCCATG 38950

Lser_US96UC23 38901 CCAAATTGCATGAGCAATAGGTCTTACATGTAAAGGGTCCTGTACCCATG 38950

Lser_LAC005780 38901 CCAAATTGCATGAGCAATAGGTCTTACATGTAAAGGGTCCTGTACCCATG 38950

Lvir_CGN013357 38899 CCAAATTGCATGAGCAATAGGTCTTACATGTAAAGGGTCCTGTACCCATG 38948

Lsal_LAC008020 38897 CCAAATTGCATGAGCAATAGGTCTTACATGTAAAGGGTCCTGTACCCATG 38946

Lsat_Salinas 38951 ACTCAAAATTTCCTTGCCAAGCTACATGAAAGAGATTTCCGGAAGTCCAC 39000

Lsat_WENDEL 38951 ACTCAAAATTTCCTTGCCAAGCTACATGAAAGAGATTTCCGGAAGTCCAC 39000

Lser_US96UC23 38951 ACTCAAAATTTCCTTGCCAAGCTACATGAAAGAGATTTCCGGAAGTCCAC 39000

Lser_LAC005780 38951 ACTCAAAATTTCCTTGCCAAGCTACATGAAAGAGATTTCCGGAAGTCCAC 39000

Lvir_CGN013357 38949 ACTCAAAATTTCCTTGCCAAGCTACATGAAAGAGATTTCCGGAAGTCCAC 38998

Lsal_LAC008020 38947 ACTCAAAATTTCCTTGCCAAGCTACATGAAAGAGATTTCCGGAAGTCCAC 38996

Lsat_Salinas 39001 AGAAAAATTATTGCTAATTGACCGAAGTGAGAAGCAAAAATATTCTGATA 39050

Lsat_WENDEL 39001 AGAAAAATTATTGCTAATTGACCGAAGTGAGAAGCAAAAATATTCTGATA 39050

Lser_US96UC23 39001 AGAAAAATTATTGCTAATTGACCGAAGTGAGAAGCAAAAATATTCTGATA 39050

Lser_LAC005780 39001 AGAAAAATTATTGCTAATTGACCGAAGTGAGAAGCAAAAATATTCTGATA 39050

Lvir_CGN013357 38999 AGAAAAATTATTGCTAATTGACCGAAGTGAGAAGCAAAAATATTCTGATA 39048

Lsal_LAC008020 38997 AGAAAAATTATTGCTAATTGACCGAAGTGAGAAGCAAAAATATTCTGATA 39046

Lsat_Salinas 39051 AAGACGTTCCTCAGTAATATCATCATGACTCTCGAAGTCATGCGCGGTAG 39100

Lsat_WENDEL 39051 AAGACGTTCCTCAGTAATATCATCATGACTCTCGAAGTCATGCGCGGTAG 39100

Lser_US96UC23 39051 AAGACGTTCCTCAGTAATATCATCATGACTCTCGAAGTCATGCGCGGTAG 39100

Lser_LAC005780 39051 AAGACGTTCCTCAGTAATATCATCATGACTCTCGAAGTCATGCGCGGTAG 39100

Lvir_CGN013357 39049 AAGACGTTCCTCAGTAATATCATCATGACTCTCGAAGTCATGCGCGGTAG 39098

Lsal_LAC008020 39047 AAGACGTTCCTCAGTAATATCATCATGACTCTCGAAGTCATGCGCGGTAG 39096

Lsat_Salinas 39101 CAATACCAAACCAAATACGACGAGTAGTGGGGTCCTGAGCTAAGCCTTGG 39150

Lsat_WENDEL 39101 CAATACCAAACCAAATACGACGAGTAGTGGGGTCCTGAGCTAAGCCTTGG 39150

Lser_US96UC23 39101 CAATACCAAACCAAATACGACGAGTAGTGGGGTCCTGAGCTAAGCCTTGG 39150

Lser_LAC005780 39101 CAATACCAAACCAAATACGACGAGTAGTGGGGTCCTGAGCTAAGCCTTGG 39150

Lvir_CGN013357 39099 CAATACCAAACCAAATACGACGAGTAGTGGGGTCCTGAGCTAAGCCTTGG 39148

Lsal_LAC008020 39097 CAATACCAAACCAAATACGACGAGTAGTGGGGTCCTGAGCTAAGCCTTGG 39146

Lsat_Salinas 39151 CTAAACCTTGGAAATCTTAATGCCATAATGCCTTTCAAATCCTCCTAGCC 39200

Lsat_WENDEL 39151 CTAAACCTTGGAAATCTTAATGCCATAATGCCTTTCAAATCCTCCTAGCC 39200

Lser_US96UC23 39151 CTAAACCTTGGAAATCTTAATGCCATAATGCCTTTCAAATCCTCCTAGCC 39200

Lser_LAC005780 39151 CTAAACCTTGGAAATCTTAATGCCATAATGCCTTTCAAATCCTCCTAGCC 39200

Lvir_CGN013357 39149 CTAAACCTTGGAAATCTTAATGCCATAATGCCTTTCAAATCCTCCTAGCC 39198

Lsal_LAC008020 39147 CTAAACCTTGGAAATCTTAATGCCATAATGCCTTTCAAATCCTCCTAGCC 39196

Lsat_Salinas 39201 ATTATCCTACTGCAATAATTCTTGCTAAGAAGAACGCCCATGTTGTGGCA 39250

Lsat_WENDEL 39201 ATTATCCTACTGCAATAATTCTTGCTAAGAAGAACGCCCATGTTGTGGCA 39250

Lser_US96UC23 39201 ATTATCCTACTGCAATAATTCTTGCTAAGAAGAACGCCCATGTTGTGGCA 39250

Lser_LAC005780 39201 ATTATCCTACTGCAATAATTCTTGCTAAGAAGAACGCCCATGTTGTGGCA 39250

Lvir_CGN013357 39199 ATTATCCTACTGCAATAATTCTTGCTAAGAAGAACGCCCATGTTGTGGCA 39248

Lsal_LAC008020 39197 ATTATCCTACTGCAATAATTCTTGCTAAGAAGAACGCCCATGTTGTGGCA 39246

Lsat_Salinas 39251 ATTCCACCCAGAAGGTAATGGGTTACTCCTACAGCACGTCCTTGTACAAT 39300

Lsat_WENDEL 39251 ATTCCACCCAGAAGGTAATGGGTTACTCCTACAGCACGTCCTTGTACAAT 39300

Lser_US96UC23 39251 ATTCCACCCAGAAGGTAATGGGTTACTCCTACAGCACGTCCTTGTACAAT 39300

Lser_LAC005780 39251 ATTCCACCCAGAAGGTAATGGGTTACTCCTACAGCACGTCCTTGTACAAT 39300

Lvir_CGN013357 39249 ATTCCACCCAGAAGGTAATGGGTTACTCCTACAGCACGTCCTTGTACAAT 39298

Lsal_LAC008020 39247 ATTCCACCCAGAAGGTAATGGGTTACTCCTACAGCACGTCCTTGTACAAT 39296

Lsat_Salinas 39301 GCTTAAGGCTCTCGGCTGGGTAGCAGGAGCAACTTTTAATTTATTATGAG 39350

Lsat_WENDEL 39301 GCTTAAGGCTCTCGGCTGGGTAGCAGGAGCAACTTTTAATTTATTATGAG 39350

Lser_US96UC23 39301 GCTTAAGGCTCTCGGCTGGGTAGCAGGAGCAACTTTTAATTTATTATGAG 39350

Lser_LAC005780 39301 GCTTAAGGCTCTCGGCTGGGTAGCAGGAGCAACTTTTAATTTATTATGAG 39350

Lvir_CGN013357 39299 GCTTAAGGCTCTCGGCTGGGTAGCAGGAGCAACTTTTAATTTATTATGAG 39348

Lsal_LAC008020 39297 GCTTAAGGCTCTCGGCTGGGTAGCAGGAGCAACTTTKAATTTATTATGAG 39346

Lsat_Salinas 39351 CCCAAACGATGGATTCAATAAGTTCTTGCCAATAACCACGTCCACTGAAT 39400

Lsat_WENDEL 39351 CCCAAACGATGGATTCAATAAGTTCTTGCCAATAACCACGTCCACTGAAT 39400

Lser_US96UC23 39351 CCCAAACGATGGATTCAATAAGTTCTTGCCAATAACCACGTCCACTGAAT 39400

Lser_LAC005780 39351 CCCAAACGATGGATTCAATAAGTTCTTGCCAATAACCACGTCCACTGAAT 39400

Lvir_CGN013357 39349 CCCAAACGATGGATTCAATAAGTTCTTGCCAATAACCACGTCCACTGAAT 39398

Lsal_LAC008020 39347 CCCAAACGATGGATTCAATAAGTTCTTGCCAATAACCACGTCCACTGAAT 39396

Lsat_Salinas 39401 AAAAACATTAAACTAAAAGCCCATACAAAATGAGCACCTAGGAAAAAAAG 39450

Lsat_WENDEL 39401 AAAAACATTAAACTAAAAGCCCATACAAAATGAGCACCTAGGAAAAAAAG 39450

Lser_US96UC23 39401 AAAAACATTAAACTAAAAGCCCATACAAAATGAGCACCTAGGAAAAAAAG 39450

Lser_LAC005780 39401 AAAAACATTAAACTAAAAGCCCATACAAAATGAGCACCTAGGAAAAAAAG 39450

Lvir_CGN013357 39399 AAAAACATTAAACTAAAAGCCCATACAAAATGAGCACCTAGGAAAAAAAG 39448

Lsal_LAC008020 39397 AAAAACATTAAACTAAAAGCCCATACAAAATGAGCACCTAGGAAAAAAAG 39446

Lsat_Salinas 39451 GCCATATGCGGATAATGAAGAACCATAAGACTGAATTACCTGGGATGCCT 39500

Lsat_WENDEL 39451 GCCATATGCGGATAATGAAGAACCATAAGACTGAATTACCTGGGATGCCT 39500

Lser_US96UC23 39451 GCCATATGCGGATAATGAAGAACCATAAGACTGAATTACCTGGGATGCCT 39500

Lser_LAC005780 39451 GCCATATGCGGATAATGAAGAACCATAAGACTGAATTACCTGGGATGCCT 39500

Lvir_CGN013357 39449 GCCATATGCGGATAATGAAGAACCATAAGACTGAATTACCTGGGATGCCT 39498

Lsal_LAC008020 39447 GCCATATGCGGATAATGAAGAACCATAAGACTGAATTACCTGGGATGCCT 39496

Lsat_Salinas 39501 GTGCCCATAAGAAATCGCGGAGCCACCCATTAATAGTAATAGAACTCTGC 39550

Lsat_WENDEL 39501 GTGCCCATAAGAAATCGCGGAGCCACCCATTAATAGTAATAGAACTCTGC 39550

Lser_US96UC23 39501 GTGCCCATAAGAAATCGCGGAGCCACCCATTAATAGTAATAGAACTCTGC 39550

Lser_LAC005780 39501 GTGCCCATAAGAAATCGCGGAGCCACCCATTAATAGTAATAGAACTCTGC 39550

Lvir_CGN013357 39499 GTGCCCATAAGAAATCGCGGAGCCACCCATTAATAGTAATAGAACTCTGC 39548

Lsal_LAC008020 39497 GTGCCCATAAGAAATCGCGGAGCCACCCATTAATAGTAATAGAACTCTGC 39546

Lsat_Salinas 39551 GCAAAGTTTCCTCCCGTGATATGAGTTACTACTCCTTGATCGCTTATACT 39600

Lsat_WENDEL 39551 GCAAAGTTTCCTCCCGTGATATGAGTTACTACTCCTTGATCGCTTATACT 39600

Lser_US96UC23 39551 GCAAAGTTTCCTCCCGTGATATGAGTTACTACTCCTTGATCGCTTATACT 39600

Lser_LAC005780 39551 GCAAAGTTTCCTCCCGTGATATGAGTTACTACTCCTTGATCGCTTATACT 39600

Lvir_CGN013357 39549 GCAAAGTTTCCTCCCGTGATATGAGTTACTACTCCTTGATCGCTTATACT 39598

Lsal_LAC008020 39547 GCAAAGTTTCCTCCCGTGATATGAGTTACTACTCCTTGATCGCTTATACT 39596

Lsat_Salinas 39601 GCCCCAAACATCTGACTGCATTTTCCAACTGAAATGAAATATGACTACTG 39650

Lsat_WENDEL 39601 GCCCCAAACATCTGACTGCATTTTCCAACTGAAATGAAATATGACTACTG 39650

Lser_US96UC23 39601 GCCCCAAACATCTGACTGCATTTTCCAACTGAAATGAAATATGACTACTG 39650

Lser_LAC005780 39601 GCCCCAAACATCTGACTGCATTTTCCAACTGAAATGAAATATGACTACTG 39650

Lvir_CGN013357 39599 GCCCCAAACATCTGACTGCATTTTCCAACTGAAATGAAATATGACTACTG 39648

Lsal_LAC008020 39597 GCCCCAAACATCTGACTGCATTTTCCAACTGAAATGAAATATGACTACTG 39646

Lsat_Salinas 39651 AAATTGAATTGTACATCCAGAATAGTCCTAAGAAAACATGGTCCCAAGCT 39700

Lsat_WENDEL 39651 AAATTGAATTGTACATCCAGAATAGTCCTAAGAAAACATGGTCCCAAGCT 39700

Lser_US96UC23 39651 AAATTGAATTGTACATCCAGAATAGTCCTAAGAAAACATGGTCCCAAGCT 39700

Lser_LAC005780 39651 AAATTGAATTGTACATCCAGAATAGTCCTAAGAAAACATGGTCCCAAGCT 39700

Lvir_CGN013357 39649 AAATTGAATTGTACATCCAGAATAGTCCTAAGAAAACATGGTCCCAAGCT 39698

Lsal_LAC008020 39647 AAATTGAATTGTACATCCAGAATAGTCCTAAGAAAACATGGTCCCAAGCT 39696

Lsat_Salinas 39701 GATACTTGACATGTCCCCCCCCTTCCAGGCCCATCACAAGGAAAACGAAA 39750

Lsat_WENDEL 39701 GATACTTGACATGTCCCCCCCCTTCCAGGCCCATCACAAGGAAAACGAAA 39750

Lser_US96UC23 39701 GATACTTGACATGTCCCCCCCCTTCCAGGCCCATCACAAGGAAAACGAAA 39750

Lser_LAC005780 39701 GATACTTGACATGTCCCCCCCCTTCCAGGCCCATCACAAGGAAAACGAAA 39750

Lvir_CGN013357 39699 GATACTTGACATGTCCCCCCCCTTCCAGGCCCATCACAAGGAAAACGAAA 39748

Lsal_LAC008020 39697 GATACTTGACATGTCCCCCCCCTTCCAGGCCCATCACAAGGAAAACGAAA 39746

Lsat_Salinas 39751 ACCAAGATTTGCTTTATCCGGTATCAAACGGGAGCTACGAGCAAATAGAA 39800

Lsat_WENDEL 39751 ACCAAGATTTGCTTTATCCGGTATCAAACGGGAGCTACGAGCAAATAGAA 39800

Lser_US96UC23 39751 ACCAAGATTTGCTTTATCCGGTATCAAACGGGAGCTACGAGCAAATAGAA 39800

Lser_LAC005780 39751 ACCAAGATTTGCTTTATCCGGTATCAAACGGGAGCTACGAGCAAATAGAA 39800

Lvir_CGN013357 39749 ACCAAGATTTGCTTTATCCGGTATCAAACGGGAGCTACGAGCAAATAGAA 39798

Lsal_LAC008020 39747 ACCAAGATTTGCTTTATCCGGTATCAAACGGGAGCTACGAGCAAATAGAA 39796

Lsat_Salinas 39801 CACCTTTCAGAAGTATCAATACCGTCACATGAATCGTAAATGCATGAATG 39850

Lsat_WENDEL 39801 CACCTTTCAGAAGTATCAATACCGTCACATGAATCGTAAATGCATGAATG 39850

Lser_US96UC23 39801 CACCTTTCAGAAGTATCAATACCGTCACATGAATCGTAAATGCATGAATG 39850

Lser_LAC005780 39801 CACCTTTCAGAAGTATCAATACCGTCACATGAATCGTAAATGCATGAATG 39850

Lvir_CGN013357 39799 CACCTTTCAGAAGTATCAATACCGTCACATGAATCGTAAATGCATGAATG 39848

Lsal_LAC008020 39797 CACCTTTCAGAAGTATCAATACCGTCACATGAATCGTAAATGCATGAATG 39846

Lsat_Salinas 39851 TGATGTACCAAAAAATCCGCCGTTCCTAATGGAATAGGTAACAAAGCTAC 39900

Lsat_WENDEL 39851 TGATGTACCAAAAAATCCGCCGTTCCTAATGGAATAGGTAACAAAGCTAC 39900

Lser_US96UC23 39851 TGATGTACCAAAAAATCCGCCGTTCCTAATGGAATAGGTAACAAAGCTAC 39900

Lser_LAC005780 39851 TGATGTACCAAAAAATCCGCCGTTCCTAATGGAATAGGTAACAAAGCTAC 39900

Lvir_CGN013357 39849 TGATGTACCAAAAAATCCGCCGTTCCTAATGGAATAGGTAACAAAGCTAC 39898

Lsal_LAC008020 39847 TGATGTACCAAAAAATCCGCCGTTCCTAATGGAATAGGTAACAAAGCTAC 39896

Lsat_Salinas 39901 CTTGCCGCCCACTGCTACTAAATCACCGCCCCCCCAAGTTAAACTGGTAC 39950

Lsat_WENDEL 39901 CTTGCCGCCCACTGCTACTAAATCACCGCCCCCCCAAGTTAAACTGGTAC 39950

Lser_US96UC23 39901 CTTGCCGCCCACTGCTACTAAATCACCGCCCCCCCAAGTTAAACTGGTAC 39950

Lser_LAC005780 39901 CTTGCCGCCCACTGCTACTAAATCACCGCCCCCCCAAGTTAAACTGGTAC 39950

Lvir_CGN013357 39899 CTTGCCGCCCACTGCTACTAAATCACCGCCCCCCCAAGTTAAACTGGTAC 39948

Lsal_LAC008020 39897 CTTGCCGCCCACTGCTACTAAATCACCGCCCCCCCAAGTTAAACTGGTAC 39946

Lsat_Salinas 39951 TTGCTGTTGCACCAGGAGCCGTTGCACCAGGTGCTAAAGCGTGGGTGTTT 40000

Lsat_WENDEL 39951 TTGCTGTTGCACCAGGAGCCGTTGCACCAGGTGCTAAAGCGTGGGTGTTT 40000

Lser_US96UC23 39951 TTGCTGTTGCACCAGGAGCCGTTGCACCAGGTGCTAAAGCGTGGGTGTTT 40000

Lser_LAC005780 39951 TTGCTGTTGCACCAGGAGCCGTTGCACCAGGTGCTAAAGCGTGGGTGTTT 40000

Lvir_CGN013357 39949 TTGCTGTTGCACCAGGAGCCGTTGCACCAGGTGCTAAAGCGTGGGTGTTT 39998

Lsal_LAC008020 39947 TTGCTGTTGCACCAGGAGCCGTTGCACCAGGTGCTAAAGCGTGGGTGTTT 39996

Lsat_Salinas 40001 TGTATCCATTGAGCAAAGACGGGTTGTAATTGGATAGCGGTATCTGAAAA 40050

Lsat_WENDEL 40001 TGTATCCATTGAGCAAAGACGGGTTGTAATTGGATAGCGGTATCTGAAAA 40050

Lser_US96UC23 40001 TGTATCCATTGAGCAAAGACGGGTTGTAATTGGATAGCGGTATCTGAAAA 40050

Lser_LAC005780 40001 TGTATCCATTGAGCAAAGACGGGTTGTAATTGGATAGCGGTATCTGAAAA 40050

Lvir_CGN013357 39999 TGTATCCATTGAGCAAAGACGGGTTGTAATTGGATAGCGGTATCTGAAAA 40048

Lsal_LAC008020 39997 TGTATCCATTGAGCAAAGACGGGTTGTAATTGGATAGCGGTATCTGAAAA 40046

Lsat_Salinas 40051 CATATCTTGAGGACGCCCTAAAGCGCTCATGGTATCATTATGAATATACA 40100

Lsat_WENDEL 40051 CATATCTTGAGGACGCCCTAAAGCGCTCATGGTATCATTATGAATATACA 40100

Lser_US96UC23 40051 CATATCTTGAGGACGCCCTAAAGCGCTCATGGTATCATTATGAATATACA 40100

Lser_LAC005780 40051 CATATCTTGAGGACGCCCTAAAGCGCTCATGGTATCATTATGAATATACA 40100

Lvir_CGN013357 40049 CATATCTTGAGGACGCCCTAAAGCGCTCATGGTATCATTATGAATATACA 40098

Lsal_LAC008020 40047 CATATCTTGAGGACGCCCTAAAGCGCTCATGGTATCATTATGAATATACA 40096

Lsat_Salinas 40101 AACCAAAACTGTGAAAGCCTAGAAATATACATGCCCAGTTGAGATGTGAT 40150

Lsat_WENDEL 40101 AACCAAAACTGTGAAAGCCTAGAAATATACATGCCCAGTTGAGATGTGAT 40150

Lser_US96UC23 40101 AACCAAAACTGTGAAAGCCTAGAAATATACATGCCCAGTTGAGATGTGAT 40150

Lser_LAC005780 40101 AACCAAAACTGTGAAAGCCTAGAAATATACATGCCCAGTTGAGATGTGAT 40150

Lvir_CGN013357 40099 AACCAAAACTGTGAAAGCCTAGAAATATACATGCCCAGTTGAGATGTGAT 40148

Lsal_LAC008020 40097 AACCAAAACTGTGAAAGCCTAGAAATATACATGCCCAGTTGAGATGTGAT 40146

Lsat_Salinas 40151 ATGATTGCATCGCGATGCCTAAGAACACGATCTAATAGATCGTTGTATCG 40200

Lsat_WENDEL 40151 ATGATTGCATCGCGATGCCTAAGAACACGATCTAATAGATCGTTGTATCG 40200

Lser_US96UC23 40151 ATGATTGCATCGCGATGCCTAAGAACACGATCTAATAGATCGTTGTATCG 40200

Lser_LAC005780 40151 ATGATTGCATCGCGATGCCTAAGAACACGATCTAATAGATCGTTGTATCG 40200

Lvir_CGN013357 40149 ATGATTGCATCGCGATGCCTAAGAACACGATCTAATAGATCGTTGTATCG 40198

Lsal_LAC008020 40147 ATGATTGCATCGCGATGCCTAAGAACACGATCTAATAGATCGTTGTATCG 40196

Lsat_Salinas 40201 AGTAGTTGGATCATAGTCTCTTACCATAAAAATGGCTGCATGCGCAGCAG 40250

Lsat_WENDEL 40201 AGTAGTTGGATCATAGTCTCTTACCATAAAAATGGCTGCATGCGCAGCAG 40250

Lser_US96UC23 40201 AGTAGTTGGATCATAGTCTCTTACCATAAAAATGGCTGCATGCGCAGCAG 40250

Lser_LAC005780 40201 AGTAGTTGGATCATAGTCTCTTACCATAAAAATGGCTGCATGCGCAGCAG 40250

Lvir_CGN013357 40199 AGTAGTTGGATCATAGTCTCTTACCATAAAAATGGCTGCATGCGCAGCAG 40248

Lsal_LAC008020 40197 AGTAGTTGGATCATAGTCTCTTACCATAAAAATGGCTGCATGCGCAGCAG 40246

Lsat_Salinas 40251 CACCAACTATGAGAAATCCACCAATCCACATATGATGTGTGAACAATGAC 40300

Lsat_WENDEL 40251 CACCAACTATGAGAAATCCACCAATCCACATATGATGTGTGAACAATGAC 40300

Lser_US96UC23 40251 CACCAACTATGAGAAATCCACCAATCCACATATGATGTGTGAACAATGAC 40300

Lser_LAC005780 40251 CACCAACTATGAGAAATCCACCAATCCACATATGATGTGTGAACAATGAC 40300

Lvir_CGN013357 40249 CACCAACTATGAGAAATCCACCAATCCACATATGATGTGTGAACAATGAC 40298

Lsal_LAC008020 40247 CACCAACTATGAGAAATCCACCAATCCACATATGATGTGTGAACAATGAC 40296

Lsat_Salinas 40301 AGTTGTGTACCATAGTCAGTAGCTAGATATGGATAAGGGGGCATGGCATA 40350

Lsat_WENDEL 40301 AGTTGTGTACCATAGTCAGTAGCTAGATATGGATAAGGGGGCATGGCATA 40350

Lser_US96UC23 40301 AGTTGTGTACCATAGTCAGTAGCTAGATATGGATAAGGGGGCATGGCATA 40350

Lser_LAC005780 40301 AGTTGTGTACCATAGTCAGTAGCTAGATATGGATAAGGGGGCATGGCATA 40350

Lvir_CGN013357 40299 AGTTGTGTACCATAGTCAGTAGCTAGATATGGATAAGGGGGCATGGCATA 40348

Lsal_LAC008020 40297 AGTTGTGTACCATAGTCAGTAGCTAGATATGGATAAGGGGGCATGGCATA 40346

Lsat_Salinas 40351 CATATGGTGAGCTACAACAATGGTTAAAGAGCCTAACATAGCTAGGTTAA 40400

Lsat_WENDEL 40351 CATATGGTGAGCTACAACAATGGTTAAAGAGCCTAACATAGCTAGGTTAA 40400

Lser_US96UC23 40351 CATATGGTGAGCTACAACAATGGTTAAAGAGCCTAACATAGCTAGGTTAA 40400

Lser_LAC005780 40351 CATATGGTGAGCTACAACAATGGTTAAAGAGCCTAACATAGCTAGGTTAA 40400

Lvir_CGN013357 40349 CATATGGTGAGCTACAACAATGGTTAAAGAGCCTAACATAGCTAGGTTAA 40398

Lsal_LAC008020 40347 CATATGGTGAGCTACAACAATGGTTAAAGAGCCTAACATAGCTAGGTTAA 40396

Lsat_Salinas 40401 GAGATAATTGAGCATGCCATGACGTTGTTAGGATCTCATATAGGCCTTTA 40450

Lsat_WENDEL 40401 GAGATAATTGAGCATGCCATGACGTTGTTAGGATCTCATATAGGCCTTTA 40450

Lser_US96UC23 40401 GAGATAATTGAGCATGCCATGACGTTGTTAGGATCTCATATAGGCCTTTA 40450

Lser_LAC005780 40401 GAGATAATTGAGCATGCCATGACGTTGTTAGGATCTCATATAGGCCTTTA 40450

Lvir_CGN013357 40399 GAGATAATTGAGCATGCCATGACGTTGTTAGGATCTCATATAGGCCTTTA 40448

Lsal_LAC008020 40397 GAGATAATTGAGCATGCCATGACGTTGTTAGGATCTCATATAGGCCTTTA 40446

Lsat_Salinas 40451 TGGCCCTGGCCCGTAAATGGACCTTTATGAGCTTCTAAAATATCTTTTAG 40500

Lsat_WENDEL 40451 TGGCCCTGGCCCGTAAATGGACCTTTATGAGCTTCTAAAATATCTTTTAG 40500

Lser_US96UC23 40451 TGGCCCTGGCCCGTAAATGGACCTTTATGAGCTTCTAAAATATCTTTTAG 40500

Lser_LAC005780 40451 TGGCCCTGGCCCGTAAATGGACCTTTATGAGCTTCTAAAATATCTTTTAG 40500

Lvir_CGN013357 40449 TGGCCCTGGCCCGTAAATGGACCTTTATGAGCTTCTAAAATATCTTTTAG 40498

Lsal_LAC008020 40447 TGGCCCTGGCCCGTAAATGGACCTTTATGAGCTTCTAAAATATCTTTTAG 40496

Lsat_Salinas 40501 ACCATGACCAATGCCCCAGTTGGTCCTATACATGTGACCCGCTATCAGAA 40550

Lsat_WENDEL 40501 ACCATGACCAATGCCCCAGTTGGTCCTATACATGTGACCCGCTATCAGAA 40550

Lser_US96UC23 40501 ACCATGACCAATGCCCCAGTTGGTCCTATACATGTGACCCGCTATCAGAA 40550

Lser_LAC005780 40501 ACCATGACCAATGCCCCAGTTGGTCCTATACATGTGACCCGCTATCAGAA 40550

Lvir_CGN013357 40499 ACCATGACCAATGCCCCAGTTGGTCCTATACATGTGACCCGCTATCAGAA 40548

Lsal_LAC008020 40497 ACCATGACCAATGCCCCAGTTGGTCCTATACATGTGACCCGCTATCAGAA 40546

Lsat_Salinas 40551 AAAGAATTGCAATAGCTAAATGATGGTGTGCCGTATCAGTTAGCCATAGA 40600

Lsat_WENDEL 40551 AAAGAATTGCAATAGCTAAATGATGGTGTGCCGTATCAGTTAGCCATAGA 40600

Lser_US96UC23 40551 AAAGAATTGCAATAGCTAAATGATGGTGTGCCGTATCAGTTAGCCATAGA 40600

Lser_LAC005780 40551 AAAGAATTGCAATAGCTAAATGATGGTGTGCCGTATCAGTTAGCCATAGA 40600

Lvir_CGN013357 40549 AAAGAATTGCAATAGCTAAATGATGGTGTGCCGTATCAGTTAGCCATAGA 40598

Lsal_LAC008020 40547 AAAGAATTGCAATAGCTAAATGATGGTGTGCCGTATCAGTTAGCCATAGA 40596

Lsat_Salinas 40601 CCTCCAGTTACTGGGTCTAATCCTCCACGAAAAGTAAGAAAGTCCGCATA 40650

Lsat_WENDEL 40601 CCTCCAGTTACTGGGTCTAATCCTCCACGAAAAGTAAGAAAGTCCGCATA 40650

Lser_US96UC23 40601 CCTCCAGTTACTGGGTCTAATCCTCCACGAAAAGTAAGAAAGTCCGCATA 40650

Lser_LAC005780 40601 CCTCCAGTTACTGGGTCTAATCCTCCACGAAAAGTAAGAAAGTCCGCATA 40650

Lvir_CGN013357 40599 CCTCCAGTTACTGGGTCTAATCCTCCACGAAAAGTCAGAAAGTCCGCATA 40648

Lsal_LAC008020 40597 CCTCCAGTTACTGGGTCTAATCCTCCACGAAAAGTAAGAAAGTCCGCATA 40646

Lsat_Salinas 40651 TTTTGACCAATTCAAGGTGAAAAATGGGGTTGCTCCCTCGGCAAAACTGG 40700

Lsat_WENDEL 40651 TTTTGACCAATTCAAGGTGAAAAATGGGGTTGCTCCCTCGGCAAAACTGG 40700

Lser_US96UC23 40651 TTTTGACCAATTCAAGGTGAAAAATGGGGTTGCTCCCTCGGCAAAACTGG 40700

Lser_LAC005780 40651 TTTTGACCAATTCAAGGTGAAAAATGGGGTTGCTCCCTCGGCAAAACTGG 40700

Lvir_CGN013357 40649 TTTTGACCAATTCAAGGTGAAAAATGGGGTTGCTCCCTCGGCAAAACTGG 40698

Lsal_LAC008020 40647 TTTTGACCAATTCAAGGTGAAAAATGGGGTTGCTCCCTCGGCAAAACTGG 40696

Lsat_Salinas 40701 GATAAAGTTGAGCCAAAAGATCCCGATTCAAGATAAATTCATGAGGAAGT 40750

Lsat_WENDEL 40701 GATAAAGTTGAGCCAAAAGATCCCGATTCAAGATAAATTCATGAGGAAGT 40750

Lser_US96UC23 40701 GATAAAGTTGAGCCAAAAGATCCCGATTCAAGATAAATTCATGAGGAAGT 40750

Lser_LAC005780 40701 GATAAAGTTGAGCCAAAAGATCCCGATTCAAGATAAATTCATGAGGAAGT 40750

Lvir_CGN013357 40699 GATAAAGTTGAGCCAAAAGATCCCGATTCAAGATAAATTCATGAGGAAGT 40748

Lsal_LAC008020 40697 GATAAAGTTGAGCCAAAAGATCCCGATTCAAGATAAATTCATGAGGAAGT 40746

Lsat_Salinas 40751 GGTATTTCTTTCGGATCTACTCCAGCGTTTAGAAATTGGTTAATCGGTAA 40800

Lsat_WENDEL 40751 GGTATTTCTTTCGGATCTACTCCAGCGTTTAGAAATTGGTTAATCGGTAA 40800

Lser_US96UC23 40751 GGTATTTCTTTCGGATCTACTCCAGCGTTTAGAAATTGGTTAATCGGTAA 40800

Lser_LAC005780 40751 GGTATTTCTTTCGGATCTACTCCAGCGTTTAGAAATTGGTTAATCGGTAA 40800

Lvir_CGN013357 40749 GGTATTTCTTTCGGATCTACTCCAGCGTTTAGAAATTGGTTAATCGGTAA 40798

Lsal_LAC008020 40747 GGGATTTCTTTCGGATCTACTCCAGCGTTTAGAAATTGGTTAATCGGTAA 40796

Lsat_Salinas 40801 AGATACATGTACTTGATGTCCCGCCCAAGAGAGAGACCCAAGTCCTAGTA 40850

Lsat_WENDEL 40801 AGATACATGTACTTGATGTCCCGCCCAAGAGAGAGACCCAAGTCCTAGTA 40850

Lser_US96UC23 40801 AGATACATGTACTTGATGTCCCGCCCAAGAGAGAGACCCAAGTCCTAGTA 40850

Lser_LAC005780 40801 AGATACATGTACTTGATGCCCCGCCCAAGAGAGAGACCCAAGTCCTAGTA 40850

Lvir_CGN013357 40799 AGATACATGTACTTGATGCCCCGCCCAAGAGAGAGACCCAAGTCCTAGTA 40848

Lsal_LAC008020 40797 AGATACATGTACTTGATGCCCCGCCCAAGAGAGAGACCCAAGTCCTAGTA 40846

Lsat_Salinas 40851 GCCCCGCTAAATGGTGATTCAACATAGATTCTACATCTTGAAACCAAGCC 40900

Lsat_WENDEL 40851 GCCCCGCTAAATGGTGATTCAACATAGATTCTACATCTTGAAACCAAGCC 40900

Lser_US96UC23 40851 GCCCCGCTAAATGGTGATTCAACATAGATTCTACATCTTGAAACCAAGCC 40900

Lser_LAC005780 40851 GCCCCGCTAAATGGTGATTCAACATAGATTCTACATCTTGAAACCAAGCC 40900

Lvir_CGN013357 40849 GCCCCGCTAAATGGTGATTCAACATAGATTCTACATCTTGAAACCAAGCC 40898

Lsal_LAC008020 40847 GCCCCGCTAAATGGTGATTCAACATAGATTCTACATCTTGAAACCAAGCC 40896

Lsat_Salinas 40901 AGTTTTGGAGCAGCTTTATGATAATGAAACCAACCAGCAAAAAGCATTAA 40950

Lsat_WENDEL 40901 AGTTTTGGAGCAGCTTTATGATAATGAAACCAACCAGCAAAAAGCATTAA 40950

Lser_US96UC23 40901 AGTTTTGGAGCAGCTTTATGATAATGAAACCAACCAGCAAAAAGCATTAA 40950

Lser_LAC005780 40901 AGTTTTGGAGCAGCTTTATGATAATGAAACCAACCAGCAAAAAGCATTAA 40950

Lvir_CGN013357 40899 AGTTTTGGAGCAGCTTTATGATAATGAAACCAACCAGCAAAAAGCATTAA 40948

Lsal_LAC008020 40897 AGTTTTGGAGCAGCTTTATGATAATGAAACCAACCAGCAAAAAGCATTAA 40946

Lsat_Salinas 40951 CGCCGCAAAGACCAATCCACCAATTGCGGTACAGTAGAGTTGTAATTCGC 41000

Lsat_WENDEL 40951 CGCCGCAAAGACCAATCCACCAATTGCGGTACAGTAGAGTTGTAATTCGC 41000

Lser_US96UC23 40951 CGCCGCAAAGACCAATCCACCAATTGCGGTACAGTAGAGTTGTAATTCGC 41000

Lser_LAC005780 40951 CGCCGCAAAGACCAATCCACCAATTGCGGTACAGTAGAGTTGTAATTCGC 41000

Lvir_CGN013357 40949 CGCCGCAAAGACCAATCCACCAATTGCGGTACAGTAGAGTTGTAATTCGC 40998

Lsal_LAC008020 40947 CCCCGCAAAGACCAATCCACCAATTGCGGTACAGTAGAGTTGTAATTCGC 40996

Lsat_Salinas 41001 TAGTTATTCCAGATGCTCGCCAAATCTGAAAAAAACCAGAGGTTATTTGT 41050

Lsat_WENDEL 41001 TAGTTATTCCAGATGCTCGCCAAATCTGAAAAAAACCAGAGGTTATTTGT 41050

Lser_US96UC23 41001 TAGTTATTCCAGATGCTCGCCAAATCTGAAAAAAACCAGAGGTTATTTGT 41050

Lser_LAC005780 41001 TAGTTATTCCAGATGCTCGCCAAATCTGAAAAAAACCAGAGGTTATTTGT 41050

Lvir_CGN013357 40999 TAGTTATTCCAGATGCTCGCCAAATCTGAAAAAAACCAGAGGTTATTTGT 41048

Lsal_LAC008020 40997 TAGTTATTCCAGATGCTCGCCAAATCTGAAAAAAACCAGAGGTTATTTGT 41046

Lsat_Salinas 41051 ATTCCTCGGAAGCCCCCGCCCACATCACCATTCAATATTTCTTGGCCCAC 41100

Lsat_WENDEL 41051 ATTCCTCGGAAGCCCCCGCCCACATCACCATTCAATATTTCTTGGCCCAC 41100

Lser_US96UC23 41051 ATTCCTCGGAAGCCCCCGCCCACATCACCATTCAATATTTCTTGGCCCAC 41100

Lser_LAC005780 41051 ATTCCTCGGAAGCCCCCGCCCACATCACCATTCAATATTTCTTGGCCCAC 41100

Lvir_CGN013357 41049 ATTCCTCGGAAGCCCCCGCCCACATCACCATTCAATATTTCTTGGCCCAC 41098

Lsal_LAC008020 41047 ATTCCTCGGAAGCCCCCGCCCACATCACCATTCAATATTTCTTGGCCCAC 41096

Lsat_Salinas 41101 TATCGGCCAAACCACTTGGGCACTAGGCCTAATGTGAGTCGGATCGCTTA 41150

Lsat_WENDEL 41101 TATCGGCCAAACCACTTGGGCACTAGGCCTAATGTGAGTCGGATCGCTTA 41150

Lser_US96UC23 41101 TATCGGCCAAACCACTTGGGCACTAGGCCTAATGTGAGTCGGATCGCTTA 41150

Lser_LAC005780 41101 TATCGGCCAAACCACTTGGGCACTAGGCCTAATGTGAGTCGGATCGCTTA 41150

Lvir_CGN013357 41099 TATCGGCCAAACCACTTGGGCACTAGGCCTAATATGAGTCGGATCGCTTA 41148

Lsal_LAC008020 41097 TATCGGCCAAACCACTTGGGCACTAGGCCTAATGTGAGTCGGATCGCTTA 41146

Lsat_Salinas 41151 GCCATGCTTCATAATTGGAAAAACGAGCACCGTGGAAATACATGCCACTC 41200

Lsat_WENDEL 41151 GCCATGCTTCATAATTGGAAAAACGAGCACCGTGGAAATACATGCCACTC 41200

Lser_US96UC23 41151 GCCATGCTTCATAATTGGAAAAACGAGCACCGTGGAAATACATGCCACTC 41200

Lser_LAC005780 41151 GCCATGCTTCATAATTGGAAAAACGAGCACCGTGGAAATACATGCCACTC 41200

Lvir_CGN013357 41149 GCCATGCTTCATAATTGGAAAAACGAGCACCGTGGAAATACATGCCACTC 41198

Lsal_LAC008020 41147 GCCATGCTTCATAATTGGAAAAACGAGCACCGTGGAAATACATGCCACTC 41196

Lsat_Salinas 41201 AGCCAAAGGAAGATGATGGAGAGTTGACCGAAATGCGCACTAAATACTTT 41250

Lsat_WENDEL 41201 AGCCAAAGGAAGATGATGGAGAGTTGACCGAAATGCGCACTAAATACTTT 41250

Lser_US96UC23 41201 AGCCAAAGGAAGATGATGGAGAGTTGACCGAAATGCGCACTAAATACTTT 41250

Lser_LAC005780 41201 AGCCAAAGGAAGATGATGGAGAGTTGACCGAAATGCGCACTAAATACTTT 41250

Lvir_CGN013357 41199 AGCCAAAGGAAGATGATGGAGAGTTGACCGAAATGCGCACTAAATACTTT 41248

Lsal_LAC008020 41197 AGCCAAAGGAAGATGATGGAGAGTTGACCGAAATGCGCACTAAATACTTT 41246

Lsat_Salinas 41251 TCGAGAGATCTCCTCCAAATCACTGGTATGGCTATCGAAATCGTGAGCAT 41300

Lsat_WENDEL 41251 TCGAGAGATCTCCTCCAAATCACTGGTATGGCTATCGAAATCGTGAGCAT 41300

Lser_US96UC23 41251 TCGAGAGATCTCCTCCAAATCACTGGTATGGCTATCGAAATCGTGAGCAT 41300

Lser_LAC005780 41251 TCGAGAGATCTCCTCCAAATCACTGGTATGGCTATCGAAATCGTGAGCAT 41300

Lvir_CGN013357 41249 TCGAGAGATCTCCTCCAAATCACTGGTATGGCTATCGAAATCGTGAGCAT 41298

Lsal_LAC008020 41247 TCGAGAGATCTCCTCCAAATCACTGGTATGGCTATCGAAATCGTGAGCAT 41296

Lsat_Salinas 41301 CAGCATGTAGGTTCCAGATCCAAGTGGTAGTTTCAGGGCCTTTAGCTATT 41350

Lsat_WENDEL 41301 CAGCATGTAGGTTCCAGATCCAAGTGGTAGTTTCAGGGCCTTTAGCTATT 41350

Lser_US96UC23 41301 CAGCATGTAGGTTCCAGATCCAAGTGGTAGTTTCAGGGCCTTTAGCTATT 41350

Lser_LAC005780 41301 CAGCATGTAGGTTCCAGATCCAAGTGGTAGTTTCAGGGCCTTTAGCTATT 41350

Lvir_CGN013357 41299 CAGCATGTAGGTTCCAGATCCAAGTGGTAGTTTCAGGGCCTTTAGCTATT 41348

Lsal_LAC008020 41297 CAGCATGTAGGTTCCAGATCCAAGTGGTAGTTTCAGGGCCTTTAGCTATT 41346

Lsat_Salinas 41351 GTTCTTGAGAAATGACCCGGTCTAGCCCATTCCTCGAATGAAGTTTTTAT 41400

Lsat_WENDEL 41351 GTTCTTGAGAAATGACCCGGTCTAGCCCATTCCTCGAATGAAGTTTTTAT 41400

Lser_US96UC23 41351 GTTCTTGAGAAATGACCCGGTCTAGCCCATTCCTCGAATGAAGTTTTTAT 41400

Lser_LAC005780 41351 GTTCTTGAGAAATGACCCGGTCTAGCCCATTCCTCGAATGAAGTTTTTAT 41400

Lvir_CGN013357 41349 GTTCTTGAGAAATGACCCGGTCTAGCCCATTCCTCGAATGAAGTTTTTAT 41398

Lsal_LAC008020 41347 GTTCTTGAGAAATGACCCGGTCTAGCCCATTCCTCGAATGAAGTTTTTAT 41396

Lsat_Salinas 41401 GTGATCCCTATCTACCAAAATTTTTACTTCTGGTTCCGGCGAACGAATAA 41450

Lsat_WENDEL 41401 GTGATCCCTATCTACCAAAATTTTTACTTCTGGTTCCGGCGAACGAATAA 41450

Lser_US96UC23 41401 GTGATCCCTATCTACCAAAATTTTTACTTCTGGTTCCGGCGAACGAATAA 41450

Lser_LAC005780 41401 GTGATCCCTATCTACCAAAATTTTTACTTCTGGTTCCGGCGAACGAATAA 41450

Lvir_CGN013357 41399 GTGATCCCTATCTACCAAAATTTTTACTTCTGGTTCCGGCGAACGAATAA 41448

Lsal_LAC008020 41397 GTGATCCCTATCTACCAAAATTTTTACTTCTGGTTCCGGCGAACGAATAA 41446

Lsat_Salinas 41451 TCATTGAGTCCTCCTCTTTCCGGACAACACATACAAAGAGACCCGCCAAC 41500

Lsat_WENDEL 41451 TCATTGAGTCCTCCTCTTTCCGGACAACACATACAAAGAGACCCGCCAAC 41500

Lser_US96UC23 41451 TCATTGAGTCCTCCTCTTTCCGGACAACACATACAAAGAGACCCGCCAAC 41500

Lser_LAC005780 41451 TCATTGAGTCCTCCTCTTTCCGGACAACACATACAAAGAGACCCGCCAAC 41500

Lvir_CGN013357 41449 TCATTGAGTCCTCCTCTTTCCGGACAACACATACAAAGAGACCCGCCAAC 41498

Lsal_LAC008020 41447 TCATTGAGTCCTCCTCTTTCCGGACAACACATACAAAGAGACCCGCCAAC 41496

Lsat_Salinas 41501 AGTCAAATAATTAGTGAATCTCGGAGAGATATTTCTATTATATAATTAGT 41550

Lsat_WENDEL 41501 AGTCAAATAATTAGTGAATCTCGGAGAGATATTTCTATTATATAATTAGT 41550

Lser_US96UC23 41501 AGTCAAATAATTAGTGAATCTCGGAGAGATATTTCTATTATATAATTAGT 41550

Lser_LAC005780 41501 AGTCAAATAATTAGTGAATCTCGGAGAGATATTTCTATTATATAATTAGT 41550

Lvir_CGN013357 41499 AGTCAAATAATTAGTGAATCTCGGAGAGATATTTCTATTATATAATTAGT 41548

Lsal_LAC008020 41497 AGTCAAATAATTAGTGAATCTCGGAGAGATATTTCTATTATATAATTAGT 41546

Lsat_Salinas 41551 TTCTTTCTCTTCTAGTTTTCTATCTCCCATCTATCTATTTTCTTTAGTTA 41600

Lsat_WENDEL 41551 TTCTTTCTCTTCTAGTTTTCTATCTCCCATCTATCTATTTTCTTTAGTTA 41600

Lser_US96UC23 41551 TTCTTTCTCTTCTAGTTTTCTATCTCCCATCTATCTATTTTCTTTAGTTA 41600

Lser_LAC005780 41551 TTCTTTCTCTTCTAGTTTTCTATCTCCCATCTATCTATTTTCTTTAGTTA 41600

Lvir_CGN013357 41549 TTCTTTCTCTTCTAGTTTTCTATCTCCCATCTATCTATTTTCTTTAGTTA 41598

Lsal_LAC008020 41547 TTCTTTCTCTTCTAGTTTTCTATCTCCCATCTATCTATTTTCTTTAGTTA 41596

Lsat_Salinas 41601 TTCACTAGAGCAATTATGATCTGGAAGTCGATCCGGGGCAAGTGTTCGGA 41650

Lsat_WENDEL 41601 TTCACTAGAGCAATTATGATCTGGAAGTCGATCCGGGGCAAGTGTTCGGA 41650

Lser_US96UC23 41601 TTCACTAGAGCAATTATGATCTGGAAGTCGATCCGGGGCAAGTGTTCGGA 41650

Lser_LAC005780 41601 TTCACTAGAGCAATTATGATCTGGAAGTCGATCCGGGGCAAGTGTTCGGA 41650

Lvir_CGN013357 41599 TTCACTAGAGCAATTATGATCTGGAAGTCGATCCGGGGCAAGTGTTCGGA 41648

Lsal_LAC008020 41597 TTCACTAGAGCAATTATGATCTGGAAGTCGATCCGGGGCAAGTGTTCGGA 41646

Lsat_Salinas 41651 TCTATTATGACATAGCCATGAGGCGCTCAACGGACCTTTTGGATCTTCTA 41700

Lsat_WENDEL 41651 TCTATTATGACATAGCCATGAGGCGCTCAACGGACCTTTTGGATCTTCTA 41700

Lser_US96UC23 41651 TCTATTATGACATAGCCATGAGGCGCTCAACGGACCTTTTGGATCTTCTA 41700

Lser_LAC005780 41651 TCTATTATGACATAGCCATGAGGCGCTCAACGGACCTTTTGGATCTTCTA 41700

Lvir_CGN013357 41649 TCTATTATGACATAGCCATGAGGCGCTCAACGGACCTTTTGGATCTTATA 41698

Lsal_LAC008020 41647 TCTATTATGACATAGCCATGAGGCGCTCAACGGACCTTTTGGATCTTATA 41696

Lsat_Salinas 41701 AAACCCTTTCTGACTTTAGATTGATACAAAAACTACTTTTTGTGCAACCT 41750

Lsat_WENDEL 41701 AAACCCTTTCTGACTTTAGATTGATACAAAAACTACTTTTTGTGCAACCT 41750

Lser_US96UC23 41701 AAACCCTTTCTGACTTTAGATTGATACAAAAACTACTTTTTGTGCAACCT 41750

Lser_LAC005780 41701 AAACCCTTTCTGACTTTAGATTGATACAAAAACTACTTTTTGTGCAACCT 41750

Lvir_CGN013357 41699 AAACCCTTTCTGACTTTAGATTGATACAAAAACTACTTTTTGTGCAACCT 41748

Lsal_LAC008020 41697 AAACCCTTTCTGACTTTAGATTGATACAAAAACTACTTTTTGTGCAACCT 41746

Lsat_Salinas 41751 AGTATATTTCAGATCTCAATTAAAAGTTATTAGATGGAGCTGCTTCATGT 41800

Lsat_WENDEL 41751 AGTATATTTCAGATCTCAATTAAAAGTTATTAGATGGAGCTGCTTCATGT 41800

Lser_US96UC23 41751 AGTATATTTCAGATCTCAATTAAAAGTTATTAGATGGAGCTGCTTCATGT 41800

Lser_LAC005780 41751 AGTATATTTCAGATCTCAATTAAAAGTTATTAGATGGAGCTGCTTCATGT 41800

Lvir_CGN013357 41749 AGTATATTTCAGATCTCAATTAAAAGTTATTAGATGGAGCTGCTTCATGT 41798

Lsal_LAC008020 41747 AGTATATTTCAGATCTCAATTAAAAGTTATTAGATGGAGCTGCTTCATGT 41796

Lsat_Salinas 41801 TTTTTAGATAGTACTATTACTCTATTCCAAATCACGCGAGCAGCCATTAG 41850

Lsat_WENDEL 41801 TTTTTAGATAGTACTATTACTCTATTCCAAATCACGCGAGCAGCCATTAG 41850

Lser_US96UC23 41801 TTTTTAGATAGTACTATTACTCTATTCCAAATCACGCGAGCAGCCATTAG 41850

Lser_LAC005780 41801 TTTTTAGATAGTACTATTACTCTATTCCAAATCACGCGAGCAGCCATTAG 41850

Lvir_CGN013357 41799 TTTTTAGATAGTACTATTACTCTATTCCAAATCACGCGAGCAGCCATTAG 41848

Lsal_LAC008020 41797 TTTTTAGATAGTACTATTACTCTATTCCAAATCACGCGAGCAGCCATTAG 41846

Lsat_Salinas 41851 CCATTACTAAGAAATTTTCATATATTCATTGATTCGAAGTATTTTTTATT 41900

Lsat_WENDEL 41851 CCATTACTAAGAAATTTTCATATATTCATTGATTCGAAGTATTTTTTATT 41900

Lser_US96UC23 41851 CCATTACTAAGAAATTTTCATATATTCATTGATTCGAAGTATTTTTTATT 41900

Lser_LAC005780 41851 CCATTACTAAGAAATTTTCATATATTCATTGATTCGAAGTATTTTTTATT 41900

Lvir_CGN013357 41849 CCATTACTAAGAAATTTTCATATATTCATTGATTCGAAGTATTTTTTATT 41898

Lsal_LAC008020 41847 TCATTACTAAGAAATTTTCATATATTCATTCATTCGAAGTATTTTTTATT 41896

Lsat_Salinas 41901 TGATTAGTTTTAATCGTCTTTTTTTTTTAATAGAAAAAAAGAAAGACAAT 41950

Lsat_WENDEL 41901 TGATTAGTTTTAATCGTCTTTTTTTTTTAATAGAAAAAAAGAAAGACAAT 41950

Lser_US96UC23 41901 TGATTAGTTTTAATCGTCTTTTTTTTTTAATAGAAAAAAAGAAAGACAAT 41950

Lser_LAC005780 41901 TGATTAGTTTTAATCGTCTTTTTTTTTTAATAGAAAAAAAGAAAGACAAT 41950

Lvir_CGN013357 41899 TGATTAGTTTTAATCGTCTTTTTTTTTTAATAGAAAAAAAGAAAGACAAT 41948

Lsal_LAC008020 41897 TGATTAGTTTTAATCGTCTTTTTTTTTTAATAGAAAAAAAGAAAGACAAT 41946

Lsat_Salinas 41951 TAAAAAATAGATTTTGTAAGCTACCCATGTATCCTTTATTCGTACGAAAT 42000

Lsat_WENDEL 41951 TAAAAAATAGATTTTGTAAGCTACCCATGTATCCTTTATTCGTACGAAAT 42000

Lser_US96UC23 41951 TAAAAAATAGATTTTGTAAGCTACCCATGTATCCTTTATTCGTACGAAAT 42000

Lser_LAC005780 41951 TAAAAAATAGATTTTGTAAGCTACCCATGTATCCTTTATTCGTACGAAAT 42000

Lvir_CGN013357 41949 TAAAAAATAGATTTTGTAAGCTACCCATGTATCCTTTATTCGTACGAAAT 41998

Lsal_LAC008020 41947 TAAAAAATAGATTTTGTAAGCTACCCATGTATCCTTTATTCGTACGAAAT 41996

Lsat_Salinas 42001 ACCATACGAAATAGAACGCTTAGAATGGATATAAAGAAATTCTTTGATTG 42050

Lsat_WENDEL 42001 ACCATACGAAATAGAACGCTTAGAATGGATATAAAGAAATTCTTTGATTG 42050

Lser_US96UC23 42001 ACCATACGAAATAGAACGCTTAGAATGGATATAAAGAAATTCTTTGATTG 42050

Lser_LAC005780 42001 ACCATACGAAATAGAACGCTTAGAATGGATATAAAGAAATTCTTTGATTG 42050

Lvir_CGN013357 41999 ACCATACGAAATAGAACGCTTAGAATGGATATAAAGAAATTCTTTGATTG 42048

Lsal_LAC008020 41997 ACCATACGAAATAGAACGCTTAGAATGGATATAAAGAAATTCTTTGATTG 42046

Lsat_Salinas 42051 GTTCTTCCCAAAGGAATGATCTATTTTATTTGACTGATGGGGCCAACAAA 42100

Lsat_WENDEL 42051 GTTCTTCCCAAAGGAATGATCTATTTTATTTGACTGATGGGGCCAACAAA 42100

Lser_US96UC23 42051 GTTCTTCCCAAAGGAATGATCTATTTTATTTGACTGATGGGGCCAACAAA 42100

Lser_LAC005780 42051 GTTCTTCCCAAAGGAATGATCTATTTTATTTGACTGATGGGGCCAACAAA 42100

Lvir_CGN013357 42049 GTTCTTCCCAAAGGAATGATCTATTTTATTTGACTGATGGGGCCAACAAA 42098

Lsal_LAC008020 42047 GTTCTTCCCAAAGGAATGATCTATTTTATTTGACTGATGGGGCCAACAAA 42096

Lsat_Salinas 42101 CAATTAATTATAACAAAAAAATATCTAAAACAATCTAAATTAGAAATTCA 42150

Lsat_WENDEL 42101 CAATTAATTATAACAAAAAAATATCTAAAACAATCTAAATTAGAAATTCA 42150

Lser_US96UC23 42101 CAATTAATTATAACAAAAAAATATCTAAAACAATCTAAATTAGAAATTCA 42150

Lser_LAC005780 42101 CAATTAATTATAACAAAAAAATATCTAAAACAATCTAAATTAGAAATTCA 42150

Lvir_CGN013357 42099 CAATTAATTATAACAAAAAAATATCTAAAACAATCTAAATTAGAAATTCA 42148

Lsal_LAC008020 42097 CAATTAATTATAACAAAAAAATATCTAAAACAATCTAAATTAGAAATTCA 42146

Lsat_Salinas 42151 ATAATAACTAAAATAAAGAAAAGGGTATCGTCTTTTATTCGAAACGTCTA 42200

Lsat_WENDEL 42151 ATAATAACTAAAATAAAGAAAAGGGTATCGTCTTTTATTCGAAACGTCTA 42200

Lser_US96UC23 42151 ATAATAACTAAAATAAAGAAAAGGGTATCGTCTTTTATTCGAAACGTCTA 42200

Lser_LAC005780 42151 ATAATAACTAAAATAAAGAAAAGGGTATCGTCTTTTATTCGAAACGTCTA 42200

Lvir_CGN013357 42149 ATAATAACTAAAATAAAGAAAAGGGTATCGTCTTTTATTCGAAACGTCTA 42198

Lsal_LAC008020 42147 ATAATAACTAAAATAAAGAAAAGGGTATCGTCTTTTATTCGAAACGTCTA 42196

Lsat_Salinas 42201 GTGATCTTCAACCAATTATGCGCTTCAATATAATTACCAGGAGTAAGTGC 42250

Lsat_WENDEL 42201 GTGATCTTCAACCAATTATGCGCTTCAATATAATTACCAGGAGTAAGTGC 42250

Lser_US96UC23 42201 GTGATCTTCAACCAATTATGCGCTTCAATATAATTACCAGGAGTAAGTGC 42250

Lser_LAC005780 42201 GTGATCTTCAACCAATTATGCGCTTCAATATAATTACCAGGAGTAAGTGC 42250

Lvir_CGN013357 42199 GTGATCTTCAACCAATTATGCGCTTCAATATAATTACCAGGAGTAAGTGC 42248

Lsal_LAC008020 42197 GTGATCTTCAACCAATTATGCGCTTCAATATAATTACCAGGAGTAAGTGC 42246

Lsat_Salinas 42251 TATAGCCTGTTTCCAATACTCAGCGGCTTGGTCGAACCAAGCCTCCGCAA 42300

Lsat_WENDEL 42251 TATAGCCTGTTTCCAATACTCAGCGGCTTGGTCGAACCAAGCCTCCGCAA 42300

Lser_US96UC23 42251 TATAGCCTGTTTCCAATACTCAGCGGCTTGGTCGAACCAAGCCTCCGCAA 42300

Lser_LAC005780 42251 TATAGCCTGTTTCCAATACTCAGCGGCTTGGTCGAACCAAGCCTCCGCAA 42300

Lvir_CGN013357 42249 TATAGCCTGTTTCCAATACTCAGCGGCTTGGTCGAACCAAGCCTCCGCAA 42298

Lsal_LAC008020 42247 TATAGCCTGTTTCCAATACTCAGCGGCTTGGTCGAACCAAGCCTCCGCAA 42296

Lsat_Salinas 42301 TTTCAGAATCCCCCTGCCGAATGGCCTGTTCTCCCCGGTCGGAATAGGTG 42350

Lsat_WENDEL 42301 TTTCAGAATCCCCCTGCCGAATGGCCTGTTCTCCCCGGTCGGAATAGGTG 42350

Lser_US96UC23 42301 TTTCAGAATCCCCCTGCCGAATGGCCTGTTCTCCCCGGTCGGAATAGGTG 42350

Lser_LAC005780 42301 TTTCAGAATCCCCCTGCCGAATGGCCTGTTCTCCCCGGTCGGAATAGGTG 42350

Lvir_CGN013357 42299 TTTCAGAATCCCCCTGCCGAATGGCCTGTTCTCCCCGGTCGGAATAGGTG 42348

Lsal_LAC008020 42297 TTTCAGAATCCCCCTGCCGAATGGCCTGTTCTCCCCGGTCGGAATAGGTG 42346

Lsat_Salinas 42351 GGTTAATTCCTTCCCTTAGAACCGTACTTGAGAGTTTCCTACCTCATACG 42400

Lsat_WENDEL 42351 GGTTAATTCCTTCCCTTAGAACCGTACTTGAGAGTTTCCTACCTCATACG 42400

Lser_US96UC23 42351 GGTTAATTCCTTCCCTTAGAACCGTACTTGAGAGTTTCCTACCTCATACG 42400

Lser_LAC005780 42351 GGTTAATTCCTTCCCTTAGAACCGTACTTGAGAGTTTCCTACCTCATACG 42400

Lvir_CGN013357 42349 GGTTAATTCCTTCCCTTAGAACCGTACTTGAGAGTTTCCTACCTCATACG 42398

Lsal_LAC008020 42347 GGTTAATTCCTTCCCTTAGAACCGTACTTGAGAGTTTCCTACCTCATACG 42396

Lsat_Salinas 42401 GCTCGGCAGTCAACTCTTTTTGTACCCCATTTGAATCTACCATATCTAAC 42450

Lsat_WENDEL 42401 GCTCGGCAGTCAACTCTTTTTGTACCCCATTTGAATCTACCATATCTAAC 42450

Lser_US96UC23 42401 GCTCGGCAGTCAACTCTTTTTGTACCCCATTTGAATCTACCATATCTAAC 42450

Lser_LAC005780 42401 GCTCGGCAGTCAACTCTTTTTGTACCCCATTTGAATCTACCATATCTAAC 42450

Lvir_CGN013357 42399 GCTCGGCAGTCAACTCTTTTTGTACCCCATTTGAATCTACCATATCTAAC 42448

Lsal_LAC008020 42397 GCTCGGCAGTCAACTCTTTTTGTACCCCATTTGAATCTACCATATCTAAC 42446

Lsat_Salinas 42451 TGAATAAGATTTCTCGTAGATCTATTCCATTTTTCGGGTTAACGAAAAGA 42500

Lsat_WENDEL 42451 TGAATAAGATTTCTCGTAGATCTATTCCATTTTTCGGGTTAACGAAAAGA 42500

Lser_US96UC23 42451 TGAATAAGATTTCTCGTAGATCTATTCCATTTTTCGGGTTAACGAAAAGA 42500

Lser_LAC005780 42451 TGAATAAGATTTCTCGTAGATCTATTCCATTTTTCGGGTTAACGAAAAGA 42500

Lvir_CGN013357 42449 TGAATAAGATTTCTCGTAGATCTATTCCATTTTTCGGGTTAACGAAAAGA 42498

Lsal_LAC008020 42447 TGAATAAGATTTCTCGTAGATCTATTCCATTTTTCGGGTTAACGAAAAGA 42496

Lsat_Salinas 42501 AGTTAATAAAATGAGTTTCAAACTTAAATCTTAAGTTTGGATTAAAAATC 42550

Lsat_WENDEL 42501 AGTTAATAAAATGAGTTTCAAACTTAAATCTTAAGTTTGGATTAAAAATC 42550

Lser_US96UC23 42501 AGTTAATAAAATGAGTTTCAAACTTAAATCTTAAGTTTGGATTAAAAATC 42550

Lser_LAC005780 42501 AGTTAATAAAATGAGTTTCAAACTTAAATCTTAAGTTTGGATTAAAAATC 42550

Lvir_CGN013357 42499 AGTTAATAAAATGAGTTTCAAACTTAAATCTTAAGTTTGGATTCAAAATC 42548

Lsal_LAC008020 42497 AGTTAATAAAATGAGTTTCAAACTTAAATCTTAAGTTTGGATTAAAAATC 42546

Lsat_Salinas 42551 CGGTTTATTTTAGTTTTATCTTTTCTCCCACCTTCAGAAGAATAAAACAT 42600

Lsat_WENDEL 42551 CGGTTTATTTTAGTTTTATCTTTTCTCCCACCTTCAGAAGAATAAAACAT 42600

Lser_US96UC23 42551 CGGTTTATTTTAGTTTTATCTTTTCTCCCACCTTCAGAAGAATAAAACAT 42600

Lser_LAC005780 42551 CGGTTTATTTTAGTTTTATCTTTTCTCCCACCTTCAGAAGAATAAAACAT 42600

Lvir_CGN013357 42549 CGGTTTATTTTAGTTTTATCTTTTCTCCCACCTTCAGAAGAATAAAACAT 42598

Lsal_LAC008020 42547 CGGTTTATTTTAGTTTTATCTTTTCTCCCACCTTCAGAAGAATAAAACAT 42596

Lsat_Salinas 42601 AGACATTTCGCCTATCATTACAATTTTCTGAAAGGTAACTATCTCAGTTT 42650

Lsat_WENDEL 42601 AGACATTTCGCCTATCATTACAATTTTCTGAAAGGTAACTATCTCAGTTT 42650

Lser_US96UC23 42601 AGACATTTCGCCTATCATTACAATTTTCTGAAAGGTAACTATCTCAGTTT 42650

Lser_LAC005780 42601 AGACATTTCGCCTATCATTACAATTTTCTGAAAGGTAACTATCTCAGTTT 42650

Lvir_CGN013357 42599 AGACATTTCGCCTATCATTACAATTTTCTGAAAGGTAACTATCTCAGTTT 42648

Lsal_LAC008020 42597 AGACATTTCGCCTATCATTACAATTTTCTGAAAGGTAACTATCTCAGTTT 42646

Lsat_Salinas 42651 CATATCATATAGAAATTTATATAGAATTTTTGAAAAAGACTTTCGAAAGG 42700

Lsat_WENDEL 42651 CATATCATATAGAAATTTATATAGAATTTTTGAAAAAGACTTTCGAAAGG 42700

Lser_US96UC23 42651 CATATCATATAGAAATTTATATAGAATTTTTGAAAAAGACTTTCGAAAGG 42700

Lser_LAC005780 42651 CATATCATATAGAAATTTATATAGAATTTTTGAAAAAGACTTTCGAAAGG 42700

Lvir_CGN013357 42649 CATATCATATAGAAATTTATATAGAATTTTTGAAAAAGACTTTCGAAAGG 42698

Lsal_LAC008020 42647 CATATCATATAGAAATTTATATAGAATTTTTGAAAAAGACTTTCGAAAGG 42696

Lsat_Salinas 42701 AAAGACTTACTATCTTTGGGATCTGATCCTACACCGCTGCTCAATACCTT 42750

Lsat_WENDEL 42701 AAAGACTTACTATCTTTGGGATCTGATCCTACACCGCTGCTCAATACCTT 42750

Lser_US96UC23 42701 AAAGACTTACTATCTTTGGGATCTGATCCTACACCGCTGCTCAATACCTT 42750

Lser_LAC005780 42701 AAAGACTTACTATCTTTGGGATCTGATCCTACACCGCTGCTCAATACCTT 42750

Lvir_CGN013357 42699 AAAGACTTACTATCTTTGGGATCTGATCCTACACCGCTGCTCAATACCTT 42748

Lsal_LAC008020 42697 AAAGACTTACTATCTTTGGGATCTGATCCTACACCGCTGCTCAATACCTT 42746

Lsat_Salinas 42751 AGTGGATCGACTCTATTACATAAGTGGATTCCTAACGTTTGTCTCACATC 42800

Lsat_WENDEL 42751 AGTGGATCGACTCTATTACATAAGTGGATTCCTAACGTTTGTCTCACATC 42800

Lser_US96UC23 42751 AGTGGATCGACTCTATTACATAAGTGGATTCCTAACGTTTGTCTCACATC 42800

Lser_LAC005780 42751 AGTGGATCGACTCTATTACATAAGTGGATTCCTAACGTTTGTCTCACATC 42800

Lvir_CGN013357 42749 AGTGGATCGACTCTATTACATAAGTGGATTCCTAACGTTTGTCTCACATC 42798

Lsal_LAC008020 42747 AGTGGATCGACTCTATTACATAAGTGGATTCCTAACGTTTGTCTCACATC 42796

Lsat_Salinas 42801 ATGACATAAGTAAGCAGTTATTATTGTATCGGCCCAAAACCTCGCTAATT 42850

Lsat_WENDEL 42801 ATGACATAAGTAAGCAGTTATTATTGTATCGGCCCAAAACCTCGCTAATT 42850

Lser_US96UC23 42801 ATGACATAAGTAAGCAGTTATTATTGTATCGGCCCAAAACCTCGCTAATT 42850

Lser_LAC005780 42801 ATGACATAAGTAAGCAGTTATTATTGTATCGGCCCAAAACCTCGCTAATT 42850

Lvir_CGN013357 42799 ATGACATAAGTAAGCAGTTATTATTGTATCGGCCCAAAACCTCGCTAATT 42848

Lsal_LAC008020 42797 ATGACATAAGTAAGCAGTTATTATTGTATCGGCCCAAAACCTCGCTAATT 42846

Lsat_Salinas 42851 GATCTTTACGGTGCTTACTCTATCAATTAGATCCTTTACCCATAGAATAA 42900

Lsat_WENDEL 42851 GATCTTTACGGTGCTTACTCTATCAATTAGATCCTTTACCCATAGAATAA 42900

Lser_US96UC23 42851 GATCTTTACGGTGCTTACTCTATCAATTAGATCCTTTACCCATAGAATAA 42900

Lser_LAC005780 42851 GATCTTTACGGTGCTTACTCTATCAATTAGATCCTTTACCCATAGAATAA 42900

Lvir_CGN013357 42849 GATCTTTACGGTGCTTACTCTATCAATTAGATCCTTTACCCATAGAATAA 42898

Lsal_LAC008020 42847 GATCTTTACGGTGCTTACTCTATCAATTAGATCCTTTACCCATAGAATAA 42896

Lsat_Salinas 42901 AACAGCTAGGCATATCTATTTCTTCATATTTCAACTTCTATGAAGTTTCT 42950

Lsat_WENDEL 42901 AACAGCTAGGCATATCTATTTCTTCATATTTCAACTTCTATGAAGTTTCT 42950

Lser_US96UC23 42901 AACAGCTAGGCATATCTATTTCTTCATATTTCAACTTCTATGAAGTTTCT 42950

Lser_LAC005780 42901 AACAGCTAGGCATATCTATTTCTTCATATTTCAACTTCTATGAAGTTTCT 42950

Lvir_CGN013357 42899 AACAGCTAGGCATATCTATTTCTTCATATTTCAACTTCTATGAAGTTTCT 42948

Lsal_LAC008020 42897 AACAGCTAGGCATATCTATTTCTTCATATTTCAACTTCTATGAAGTTTAT 42946

Lsat_Salinas 42951 TTTCTACAGCTGATAAAAATCGTTGTTTTAGACGATGCATATGTAGAAAG 43000

Lsat_WENDEL 42951 TTTCTACAGCTGATAAAAATCGTTGTTTTAGACGATGCATATGTAGAAAG 43000
[truncated: 800,163 more chars]
