# Supplementary material for: Sequence of the Mitochondrial Genome of Lactuca virosa Suggests an Unexpected Role in Lactuca sativa’s Evolution
Source: Front Plant Sci. 2021 Jul 26;12:697136. doi: 10.3389/fpls.2021.697136 (PMC8350775; doi:10.3389/fpls.2021.697136)
Supplement: Supplementary file 4 [file Data_Sheet_4.docx]

**ClustalW multiple sequence alignment of concatenated mtDNA CDS**

7 Sequences Aligned Processing time: 11 mins 636 secs

Gaps Inserted = 19 Conserved Identities = 26887

Score = 25257369

Pairwise Alignment Mode: Fast

Pairwise Alignment Parameters:

ktup = 2 Gap Penalty = 5 Top Diagonals = 4 Window Size = 4

Multiple Alignment Parameters:

Open Gap Penalty = 15.0 Extend Gap Penalty = 6.7

Delay Divergent = 30% Transitions: Weighted

LsatLACWENDEL_C 1 ATGGAATTCTCTCCGAGAGCTGCTGAACTAACGACTCTATTAGAAAGTAG 50

LsatSalinas_CDS 1 ATGGAATTCTCTCCGAGAGCTGCTGAACTAACGACTCTATTAGAAAGTAG 50

LserUS96UC23_CD 1 ATGGAATTCTCTCCGAGAGCTGCTGAACTAACGACTCTATTAGAAAGTAG 50

LvirLAC006941_C 1 ATGGAATTCTCTCCGAGAGCTGCTGAACTAACGACTCTATTAGAAAGTAG 50

LserLAC005780_C 1 ATGGAATTCTCTCCGAGAGCTGCTGAACTAACGACTCTATTAGAAAGTAG 50

LsalLAC008020_C 1 ATGGAATTCTCTCCGAGAGCTGCTGAACTAACGACTCTATTAGAAAGTAG 50

LsalCGN5271_CDS 1 ATGGAATTCTCTCCGAGAGCTGCTGAACTAACGACTCTATTAGAAAGTAG 50

LsatLACWENDEL_C 51 AATTAGCAACTTTTACACGAATTTTCAAGTGGATGAGATTGGTCGAGTGG 100

LsatSalinas_CDS 51 AATTAGCAACTTTTACACGAATTTTCAAGTGGATGAGATTGGTCGAGTGG 100

LserUS96UC23_CD 51 AATTAGCAACTTTTACACGAATTTTCAAGTGGATGAGATTGGTCGAGTGG 100

LvirLAC006941_C 51 AATTAGCAACTTTTACACGAATTTTCAAGTGGATGAGATTGGTCGAGTGG 100

LserLAC005780_C 51 AATTAGCAACTTTTACACGAATTTTCAAGTGGATGAGATTGGTCGAGTGG 100

LsalLAC008020_C 51 AATTAGCAACTTTTACACGAATTTTCAAGTGGATGAGATTGGTCGAGTGG 100

LsalCGN5271_CDS 51 AATTAGCAACTTTTACACGAATTTTCAAGTGGATGAGATTGGTCGAGTGG 100

LsatLACWENDEL_C 101 TCTCAGTTGGAGATGGGATTGCACGTGTTTATGGGTTGAACGAGATTCAA 150

LsatSalinas_CDS 101 TCTCAGTTGGAGATGGGATTGCACGTGTTTATGGGTTGAACGAGATTCAA 150

LserUS96UC23_CD 101 TCTCAGTTGGAGATGGGATTGCACGTGTTTATGGGTTGAACGAGATTCAA 150

LvirLAC006941_C 101 TCTCAGTTGGAGATGGGATTGCACGTGTTTATGGGTTGAACGAGATTCAA 150

LserLAC005780_C 101 TCTCAGTTGGAGATGGGATTGCACGTGTTTATGGGTTGAACGAGATTCAA 150

LsalLAC008020_C 101 TCTCAGTTGGAGATGGGATTGCACGTGTTTATGGGTTGAACGAGATTCAA 150

LsalCGN5271_CDS 101 TCTCAGTTGGAGATGGGATTGCACGTGTTTATGGGTTGAACGAGATTCAA 150

LsatLACWENDEL_C 151 GCTGGGGAAATGGTTGAATTTGCCAGCGGTGTGAAAGGAATAGCCTTGAA 200

LsatSalinas_CDS 151 GCTGGGGAAATGGTTGAATTTGCCAGCGGTGTGAAAGGAATAGCCTTGAA 200

LserUS96UC23_CD 151 GCTGGGGAAATGGTTGAATTTGCCAGCGGTGTGAAAGGAATAGCCTTGAA 200

LvirLAC006941_C 151 GCTGGGGAAATGGTTGAATTTGCCAGCGGTGTGAAAGGAATAGCCTTGAA 200

LserLAC005780_C 151 GCTGGGGAAATGGTTGAATTTGCCAGCGGTGTGAAAGGAATAGCCTTGAA 200

LsalLAC008020_C 151 GCTGGGGAAATGGTTGAATTTGCCAGCGGTGTGAAAGGAATAGCCTTGAA 200

LsalCGN5271_CDS 151 GCTGGGGAAATGGTTGAATTTGCCAGCGGTGTGAAAGGAATAGCCTTGAA 200

LsatLACWENDEL_C 201 TCTTGAGAATGAGAATGTAGGGATTGTTGTCTTTGGTAGTGATACTGCTA 250

LsatSalinas_CDS 201 TCTTGAGAATGAGAATGTAGGGATTGTTGTCTTTGGTAGTGATACTGCTA 250

LserUS96UC23_CD 201 TCTTGAGAATGAGAATGTAGGGATTGTTGTCTTTGGTAGTGATACTGCTA 250

LvirLAC006941_C 201 TCTTGAGAATGAGAATGTAGGGATTGTTGTCTTTGGTAGTGATACTGCTA 250

LserLAC005780_C 201 TCTTGAGAATGAGAATGTAGGGATTGTTGTCTTTGGTAGTGATACTGCTA 250

LsalLAC008020_C 201 TCTTGAGAATGAGAATGTAGGGATTGTTGTCTTTGGTAGTGATACTGCTA 250

LsalCGN5271_CDS 201 TCTTGAGAATGAGAATGTAGGGATTGTTGTCTTTGGTAGTGATACTGCTA 250

LsatLACWENDEL_C 251 TTAAAGAAGGAGATCTTGTCAAGCGCACTGGCTCTATTGTGGATGTCCCT 300

LsatSalinas_CDS 251 TTAAAGAAGGAGATCTTGTCAAGCGCACTGGCTCTATTGTGGATGTCCCT 300

LserUS96UC23_CD 251 TTAAAGAAGGAGATCTTGTCAAGCGCACTGGCTCTATTGTGGATGTCCCT 300

LvirLAC006941_C 251 TTAAAGAAGGAGATCTTGTCAAGCGCACTGGCTCTATTGTGGATGTCCCT 300

LserLAC005780_C 251 TTAAAGAAGGAGATCTTGTCAAGCGCACTGGCTCTATTGTGGATGTCCCT 300

LsalLAC008020_C 251 TTAAAGAAGGAGATCTTGTCAAGCGCACTGGCTCTATTGTGGATGTCCCT 300

LsalCGN5271_CDS 251 TTAAAGAAGGAGATCTTGTCAAGCGCACTGGCTCTATTGTGGATGTCCCT 300

LsatLACWENDEL_C 301 GCGGGAAAGGCTATGCTAGGGCGTGTGGTCGACGCCTTGGGAGTACCTAT 350

LsatSalinas_CDS 301 GCGGGAAAGGCTATGCTAGGGCGTGTGGTCGACGCCTTGGGAGTACCTAT 350

LserUS96UC23_CD 301 GCGGGAAAGGCTATGCTAGGGCGTGTGGTCGACGCCTTGGGAGTACCTAT 350

LvirLAC006941_C 301 GCGGGAAAGGCTATGCTAGGGCGTGTGGTCGACGCCTTGGGAGTACCTAT 350

LserLAC005780_C 301 GCGGGAAAGGCTATGCTAGGGCGTGTGGTCGACGCCTTGGGAGTACCTAT 350

LsalLAC008020_C 301 GCGGGAAAGGCTATGCTAGGGCGTGTGGTCGACGCCTTGGGAGTACCTAT 350

LsalCGN5271_CDS 301 GCGGGAAAGGCTATGCTAGGGCGTGTGGTCGACGCCTTGGGAGTACCTAT 350

LsatLACWENDEL_C 351 TGATGGAAGAGGGGCTCTAAGCGATCACGAGCGAAGACGTGTCGAAGTGA 400

LsatSalinas_CDS 351 TGATGGAAGAGGGGCTCTAAGCGATCACGAGCGAAGACGTGTCGAAGTGA 400

LserUS96UC23_CD 351 TGATGGAAGAGGGGCTCTAAGCGATCACGAGCGAAGACGTGTCGAAGTGA 400

LvirLAC006941_C 351 TGATGGAAGAGGGGCTCTAAGCGATCACGAGCGAAGACGTGTCGAAGTGA 400

LserLAC005780_C 351 TGATGGAAGAGGGGCTCTAAGCGATCACGAGCGAAGACGTGTCGAAGTGA 400

LsalLAC008020_C 351 TGATGGAAGAGGGGCTCTAAGCGATCACGAGCGAAGACGTGTCGAAGTGA 400

LsalCGN5271_CDS 351 TGATGGAAGAGGGGCTCTAAGCGATCACGAGCGAAGACGTGTCGAAGTGA 400

LsatLACWENDEL_C 401 AAGCCCCTGGGATTATTGAACGTAAATCTGTGCACGAGCCTATGCAAACT 450

LsatSalinas_CDS 401 AAGCCCCTGGGATTATTGAACGTAAATCTGTGCACGAGCCTATGCAAACT 450

LserUS96UC23_CD 401 AAGCCCCTGGGATTATTGAACGTAAATCTGTGCACGAGCCTATGCAAACT 450

LvirLAC006941_C 401 AAGCCCCTGGGATTATTGAACGTAAATCTGTGCACGAGCCTATGCAAACT 450

LserLAC005780_C 401 AAGCCCCTGGGATTATTGAACGTAAATCTGTGCACGAGCCTATGCAAACT 450

LsalLAC008020_C 401 AAGCCCCTGGGATTATTGAACGTAAATCTGTGCACGAGCCTATGCAAACT 450

LsalCGN5271_CDS 401 AAGCCCCTGGGATTATTGAACGTAAATCTGTGCACGAGCCTATGCAAACT 450

LsatLACWENDEL_C 451 GGGTTAAAAGCGGTAGATAGCCTGGTTCCTATAGGCCGTGGTCAACGAGA 500

LsatSalinas_CDS 451 GGGTTAAAAGCGGTAGATAGCCTGGTTCCTATAGGCCGTGGTCAACGAGA 500

LserUS96UC23_CD 451 GGGTTAAAAGCGGTAGATAGCCTGGTTCCTATAGGCCGTGGTCAACGAGA 500

LvirLAC006941_C 451 GGGTTAAAAGCGGTAGATAGCCTGGTTCCTATAGGCCGTGGTCAACGAGA 500

LserLAC005780_C 451 GGGTTAAAAGCGGTAGATAGCCTGGTTCCTATAGGCCGTGGTCAACGAGA 500

LsalLAC008020_C 451 GGGTTAAAAGCGGTAGATAGCCTGGTTCCTATAGGCCGTGGTCAACGAGA 500

LsalCGN5271_CDS 451 GGGTTAAAAGCGGTAGATAGCCTGGTTCCTATAGGCCGTGGTCAACGAGA 500

LsatLACWENDEL_C 501 ACTTATAATCGGGGACCGACAAACTGGAAAAACAGCTATTGCTATCGATA 550

LsatSalinas_CDS 501 ACTTATAATCGGGGACCGACAAACTGGAAAAACAGCTATTGCTATCGATA 550

LserUS96UC23_CD 501 ACTTATAATCGGGGACCGACAAACTGGAAAAACAGCTATTGCTATCGATA 550

LvirLAC006941_C 501 ACTTATAATCGGGGACCGACAAACTGGAAAAACAGCTATTGCTATCGATA 550

LserLAC005780_C 501 ACTTATAATCGGGGACCGACAAACTGGAAAAACAGCTATTGCTATCGATA 550

LsalLAC008020_C 501 ACTTATAATCGGGGACCGACAAACTGGAAAAACAGCTATTGCTATCGATA 550

LsalCGN5271_CDS 501 ACTTATAATCGGGGACCGACAAACTGGAAAAACAGCTATTGCTATCGATA 550

LsatLACWENDEL_C 551 CCATATTAAACCAAAAGCAAATGAACTCAAGGAGCACCTCTGAGAGTGAG 600

LsatSalinas_CDS 551 CCATATTAAACCAAAAGCAAATGAACTCAAGGAGCACCTCTGAGAGTGAG 600

LserUS96UC23_CD 551 CCATATTAAACCAAAAGCAAATGAACTCAAGGAGCACCTCTGAGAGTGAG 600

LvirLAC006941_C 551 CCATATTAAACCAAAAGCAAATGAACTCAAGGAGCACCTCTGAGAGTGAG 600

LserLAC005780_C 551 CCATATTAAACCAAAAGCAAATGAACTCAAGGAGCACCTCTGAGAGTGAG 600

LsalLAC008020_C 551 CCATATTAAACCAAAAGCAAATGAACTCAAGGAGCACCTCTGAGAGTGAG 600

LsalCGN5271_CDS 551 CCATATTAAACCAAAAGCAAATGAACTCAAGGAGCACCTCTGAGAGTGAG 600

LsatLACWENDEL_C 601 ACATTGTATTGTGTCTATGTAGCGATTGGACAGAAACGCTCAACTGTGGC 650

LsatSalinas_CDS 601 ACATTGTATTGTGTCTATGTAGCGATTGGACAGAAACGCTCAACTGTGGC 650

LserUS96UC23_CD 601 ACATTGTATTGTGTCTATGTAGCGATTGGACAGAAACGCTCAACTGTGGC 650

LvirLAC006941_C 601 ACATTGTATTGTGTCTATGTAGCGATTGGACAGAAACGCTCAACTGTGGC 650

LserLAC005780_C 601 ACATTGTATTGTGTCTATGTAGCGATTGGACAGAAACGCTCAACTGTGGC 650

LsalLAC008020_C 601 ACATTGTATTGTGTCTATGTAGCGATTGGACAGAAACGCTCAACTGTGGC 650

LsalCGN5271_CDS 601 ACATTGTATTGTGTCTATGTAGCGATTGGACAGAAACGCTCAACTGTGGC 650

LsatLACWENDEL_C 651 ACAATTAGTTCAAATTCTTTCAGAAGCGAATGCTATGGAATATTCCATTC 700

LsatSalinas_CDS 651 ACAATTAGTTCAAATTCTTTCAGAAGCGAATGCTATGGAATATTCCATTC 700

LserUS96UC23_CD 651 ACAATTAGTTCAAATTCTTTCAGAAGCGAATGCTATGGAATATTCCATTC 700

LvirLAC006941_C 651 ACAATTAGTTCAAATTCTTTCAGAAGCGAATGCTATGGAATATTCCATTC 700

LserLAC005780_C 651 ACAATTAGTTCAAATTCTTTCAGAAGCGAATGCTATGGAATATTCCATTC 700

LsalLAC008020_C 651 ACAATTAGTTCAAATTCTTTCAGAAGCGAATGCTATGGAATATTCCATTC 700

LsalCGN5271_CDS 651 ACAATTAGTTCAAATTCTTTCAGAAGCGAATGCTATGGAATATTCCATTC 700

LsatLACWENDEL_C 701 TTGTAGCAGCCACCGCTTCGGATCCTGCTCCTCTGCAATTTCTGGCCCCA 750

LsatSalinas_CDS 701 TTGTAGCAGCCACCGCTTCGGATCCTGCTCCTCTGCAATTTCTGGCCCCA 750

LserUS96UC23_CD 701 TTGTAGCAGCCACCGCTTCGGATCCTGCTCCTCTGCAATTTCTGGCCCCA 750

LvirLAC006941_C 701 TTGTAGCAGCCACCGCTTCGGATCCTGCTCCTCTGCAATTTCTGGCCCCA 750

LserLAC005780_C 701 TTGTAGCAGCCACCGCTTCGGATCCTGCTCCTCTGCAATTTCTGGCCCCA 750

LsalLAC008020_C 701 TTGTAGCAGCCACCGCTTCGGATCCTGCTCCTCTGCAATTTCTGGCCCCA 750

LsalCGN5271_CDS 701 TTGTAGCAGCCACCGCTTCGGATCCTGCTCCTCTGCAATTTCTGGCCCCA 750

LsatLACWENDEL_C 751 TATTCTGGCTGTGCCATGGGGGAATATTTCCGCGATAATGGAATGCACGC 800

LsatSalinas_CDS 751 TATTCTGGCTGTGCCATGGGGGAATATTTCCGCGATAATGGAATGCACGC 800

LserUS96UC23_CD 751 TATTCTGGCTGTGCCATGGGGGAATATTTCCGCGATAATGGAATGCACGC 800

LvirLAC006941_C 751 TATTCTGGCTGTGCCATGGGGGAATATTTCCGCGATAATGGAATGCACGC 800

LserLAC005780_C 751 TATTCTGGCTGTGCCATGGGGGAATATTTCCGCGATAATGGAATGCACGC 800

LsalLAC008020_C 751 TATTCTGGCTGTGCCATGGGGGAATATTTCCGCGATAATGGAATGCACGC 800

LsalCGN5271_CDS 751 TATTCTGGCTGTGCCATGGGGGAATATTTCCGCGATAATGGAATGCACGC 800

LsatLACWENDEL_C 801 ATTAATAATCTATGATGATCTTAGTAAACAGGCAGTGGCATATCGACAAA 850

LsatSalinas_CDS 801 ATTAATAATCTATGATGATCTTAGTAAACAGGCAGTGGCATATCGACAAA 850

LserUS96UC23_CD 801 ATTAATAATCTATGATGATCTTAGTAAACAGGCAGTGGCATATCGACAAA 850

LvirLAC006941_C 801 ATTAATAATCTATGATGATCTTAGTAAACAGGCAGTGGCATATCGACAAA 850

LserLAC005780_C 801 ATTAATAATCTATGATGATCTTAGTAAACAGGCAGTGGCATATCGACAAA 850

LsalLAC008020_C 801 ATTAATAATCTATGATGATCTTAGTAAACAGGCAGTGGCATATCGACAAA 850

LsalCGN5271_CDS 801 ATTAATAATCTATGATGATCTTAGTAAACAGGCAGTGGCATATCGACAAA 850

LsatLACWENDEL_C 851 TGTCATTATTGTTACGCCGACCACCAGGCCGTGAGGCTTTCCCAGGGGAT 900

LsatSalinas_CDS 851 TGTCATTATTGTTACGCCGACCACCAGGCCGTGAGGCTTTCCCAGGGGAT 900

LserUS96UC23_CD 851 TGTCATTATTGTTACGCCGACCACCAGGCCGTGAGGCTTTCCCAGGGGAT 900

LvirLAC006941_C 851 TGTCATTATTGTTACGCCGACCACCAGGCCGTGAGGCTTTCCCAGGGGAT 900

LserLAC005780_C 851 TGTCATTATTGTTACGCCGACCACCAGGCCGTGAGGCTTTCCCAGGGGAT 900

LsalLAC008020_C 851 TGTCATTATTGTTACGCCGACCACCAGGCCGTGAGGCTTTCCCAGGGGAT 900

LsalCGN5271_CDS 851 TGTCATTATTGTTACGCCGACCACCAGGCCGTGAGGCTTTCCCAGGGGAT 900

LsatLACWENDEL_C 901 GTTTTCTATTTACATTCCCGTCTCTTAGAAAGAGCCGCTAAACGATCGGA 950

LsatSalinas_CDS 901 GTTTTCTATTTACATTCCCGTCTCTTAGAAAGAGCCGCTAAACGATCGGA 950

LserUS96UC23_CD 901 GTTTTCTATTTACATTCCCGTCTCTTAGAAAGAGCCGCTAAACGATCGGA 950

LvirLAC006941_C 901 GTTTTCTATTTACATTCCCGTCTCTTAGAAAGAGCCGCTAAACGATCGGA 950

LserLAC005780_C 901 GTTTTCTATTTACATTCCCGTCTCTTAGAAAGAGCCGCTAAACGATCGGA 950

LsalLAC008020_C 901 GTTTTCTATTTACATTCCCGTCTCTTAGAAAGAGCCGCTAAACGATCGGA 950

LsalCGN5271_CDS 901 GTTTTCTATTTACATTCCCGTCTCTTAGAAAGAGCCGCTAAACGATCGGA 950

LsatLACWENDEL_C 951 CCAGACAGGCGCAGGTAGCTTGACCGCCTTACCCGTCATTGAAACACAAG 1000

LsatSalinas_CDS 951 CCAGACAGGCGCAGGTAGCTTGACCGCCTTACCCGTCATTGAAACACAAG 1000

LserUS96UC23_CD 951 CCAGACAGGCGCAGGTAGCTTGACCGCCTTACCCGTCATTGAAACACAAG 1000

LvirLAC006941_C 951 CCAGACAGGCGCAGGTAGCTTGACCGCCTTACCCGTCATTGAAACACAAG 1000

LserLAC005780_C 951 CCAGACAGGCGCAGGTAGCTTGACCGCCTTACCCGTCATTGAAACACAAG 1000

LsalLAC008020_C 951 CCAGACAGGCGCAGGTAGCTTGACCGCCTTACCCGTCATTGAAACACAAG 1000

LsalCGN5271_CDS 951 CCAGACAGGCGCAGGTAGCTTGACCGCCTTACCCGTCATTGAAACACAAG 1000

LsatLACWENDEL_C 1001 CTGGAGACGTATCAGCCTATATTCCTACTAATGTGATCCCCATTACTGAT 1050

LsatSalinas_CDS 1001 CTGGAGACGTATCAGCCTATATTCCTACTAATGTGATCCCCATTACTGAT 1050

LserUS96UC23_CD 1001 CTGGAGACGTATCAGCCTATATTCCTACTAATGTGATCCCCATTACTGAT 1050

LvirLAC006941_C 1001 CTGGAGACGTATCAGCCTATATTCCTACTAATGTGATCCCCATTACTGAT 1050

LserLAC005780_C 1001 CTGGAGACGTATCAGCCTATATTCCTACTAATGTGATCCCCATTACTGAT 1050

LsalLAC008020_C 1001 CTGGAGACGTATCAGCCTATATTCCTACTAATGTGATCCCCATTACTGAT 1050

LsalCGN5271_CDS 1001 CTGGAGACGTATCAGCCTATATTCCTACTAATGTGATCCCCATTACTGAT 1050

LsatLACWENDEL_C 1051 GGACAAATCTGTTCGGAAACAGAGCTCTTTTATCGCGGAATTAGACCTGC 1100

LsatSalinas_CDS 1051 GGACAAATCTGTTCGGAAACAGAGCTCTTTTATCGCGGAATTAGACCTGC 1100

LserUS96UC23_CD 1051 GGACAAATCTGTTCGGAAACAGAGCTCTTTTATCGCGGAATTAGACCTGC 1100

LvirLAC006941_C 1051 GGACAAATCTGTTCGGAAACAGAGCTCTTTTATCGCGGAATTAGACCTGC 1100

LserLAC005780_C 1051 GGACAAATCTGTTCGGAAACAGAGCTCTTTTATCGCGGAATTAGACCTGC 1100

LsalLAC008020_C 1051 GGACAAATCTGTTCGGAAACAGAGCTCTTTTATCGCGGAATTAGACCTGC 1100

LsalCGN5271_CDS 1051 GGACAAATCTGTTCGGAAACAGAGCTCTTTTATCGCGGAATTAGACCTGC 1100

LsatLACWENDEL_C 1101 TATTAACGTCGGCTTATCTGTCAGTCGTGTTGGGTCTGCCGCTCAGTTGA 1150

LsatSalinas_CDS 1101 TATTAACGTCGGCTTATCTGTCAGTCGTGTTGGGTCTGCCGCTCAGTTGA 1150

LserUS96UC23_CD 1101 TATTAACGTCGGCTTATCTGTCAGTCGTGTTGGGTCTGCCGCTCAGTTGA 1150

LvirLAC006941_C 1101 TATTAACGTCGGCTTATCTGTCAGTCGTGTTGGGTCTGCCGCTCAGTTGA 1150

LserLAC005780_C 1101 TATTAACGTCGGCTTATCTGTCAGTCGTGTTGGGTCTGCCGCTCAGTTGA 1150

LsalLAC008020_C 1101 TATTAACGTCGGCTTATCTGTCAGTCGTGTTGGGTCTGCCGCTCAGTTGA 1150

LsalCGN5271_CDS 1101 TATTAACGTCGGCTTATCTGTCAGTCGTGTTGGGTCTGCCGCTCAGTTGA 1150

LsatLACWENDEL_C 1151 AAACTATGAAACAAGTCTGCGGTAGTTCAAAACTGGAATTGGCACAATAT 1200

LsatSalinas_CDS 1151 AAACTATGAAACAAGTCTGCGGTAGTTCAAAACTGGAATTGGCACAATAT 1200

LserUS96UC23_CD 1151 AAACTATGAAACAAGTCTGCGGTAGTTCAAAACTGGAATTGGCACAATAT 1200

LvirLAC006941_C 1151 AAACTATGAAACAAGTCTGCGGTAGTTCAAAACTGGAATTGGCACAATAT 1200

LserLAC005780_C 1151 AAACTATGAAACAAGTCTGCGGTAGTTCAAAACTGGAATTGGCACAATAT 1200

LsalLAC008020_C 1151 AAACTATGAAACAAGTCTGCGGTAGTTCAAAACTGGAATTGGCACAATAT 1200

LsalCGN5271_CDS 1151 AAACTATGAAACAAGTCTGCGGTAGTTCAAAACTGGAATTGGCACAATAT 1200

LsatLACWENDEL_C 1201 CGCGAAGTGGCCGCCCTTGCTCAATTTGGGTCAGACCTGGATGCTGCGAC 1250

LsatSalinas_CDS 1201 CGCGAAGTGGCCGCCCTTGCTCAATTTGGGTCAGACCTGGATGCTGCGAC 1250

LserUS96UC23_CD 1201 CGCGAAGTGGCCGCCCTTGCTCAATTTGGGTCAGACCTGGATGCTGCGAC 1250

LvirLAC006941_C 1201 CGCGAAGTGGCCGCCCTTGCTCAATTTGGGTCAGACCTGGATGCTGCGAC 1250

LserLAC005780_C 1201 CGCGAAGTGGCCGCCCTTGCTCAATTTGGGTCAGACCTGGATGCTGCGAC 1250

LsalLAC008020_C 1201 CGCGAAGTGGCCGCCCTTGCTCAATTTGGGTCAGACCTGGATGCTGCGAC 1250

LsalCGN5271_CDS 1201 CGCGAAGTGGCCGCCCTTGCTCAATTTGGGTCAGACCTGGATGCTGCGAC 1250

LsatLACWENDEL_C 1251 TCAGGCATTACTCAATAGAGGTGCAAGGCTTACAGAAGTACCGAAACAAC 1300

LsatSalinas_CDS 1251 TCAGGCATTACTCAATAGAGGTGCAAGGCTTACAGAAGTACCGAAACAAC 1300

LserUS96UC23_CD 1251 TCAGGCATTACTCAATAGAGGTGCAAGGCTTACAGAAGTACCGAAACAAC 1300

LvirLAC006941_C 1251 TCAGGCATTACTCAATAGAGGTGCAAGGCTTACAGAAGTACCGAAACAAC 1300

LserLAC005780_C 1251 TCAGGCATTACTCAATAGAGGTGCAAGGCTTACAGAAGTACCGAAACAAC 1300

LsalLAC008020_C 1251 TCAGGCATTACTCAATAGAGGTGCAAGGCTTACAGAAGTACCGAAACAAC 1300

LsalCGN5271_CDS 1251 TCAGGCATTACTCAATAGAGGTGCAAGGCTTACAGAAGTACCGAAACAAC 1300

LsatLACWENDEL_C 1301 CACAATATGCACCACTTCCTATTGAAAAAAAATTTTTAGCCCCTAGGGAA 1350

LsatSalinas_CDS 1301 CACAATATGCACCACTTCCTATTGAAAAAAAATTTTTAGCCCCTAGGGAA 1350

LserUS96UC23_CD 1301 CACAATATGCACCACTTCCTATTGAAAAAAAATTTTTAGCCCCTAGGGAA 1350

LvirLAC006941_C 1301 CACAATATGCACCACTTCCTATTGAAAAAAAATTTTTAGCCCCTAGGGAA 1350

LserLAC005780_C 1301 CACAATATGCACCACTTCCAATTGAAAAACAAATTTTAGTC---ATGGAA 1347

LsalLAC008020_C 1301 CACAATATGCACCACTTCCAATTGAAAAACAAATTTTAGTC---ATGGAA 1347

LsalCGN5271_CDS 1301 CACAATATGCACCACTTCCAATTGAAAAACAAATTTTAGTC---ATGGAA 1347

LsatLACWENDEL_C 1351 ATGGAACCGGATACATCCCCCCGCGGGCGCCGCTTTTT------------ 1388

LsatSalinas_CDS 1351 ATGGAACCGGATACATCCCCCCGCGGGCGCCGCTTTTT------------ 1388

LserUS96UC23_CD 1351 ATGGAACCGGATACATCCCCCCGCGGGCGCCGCTTTTT------------ 1388

LvirLAC006941_C 1351 ATGGAACCGGATACATCCCCCCGCGGGCGCCGCTTTTTATCTGTGGTCTT 1400

LserLAC005780_C 1348 CCAGATACATCCCCCCGCCCCCGTAAACGCCGCTTTCTCTCTTGGGTCTT 1397

LsalLAC008020_C 1348 CCAGATACATCCCCCCGCCCCCGTAAACGCCGCTTTCTCTCTTGGGTCTT 1397

LsalCGN5271_CDS 1348 CCAGATACATCCCCCCGCCCCCGTAAACGCCGCTTTCTCTCTTGGGTCTT 1397

LsatLACWENDEL_C 1389 ---CTCTTTTTTTATACTGGCGTTCGCCTTCATAGGCATAGGCTTAGGCA 1435

LsatSalinas_CDS 1389 ---CTCTTTTTTTATACTGGCGTTCGCCTTCATAGGCATAGGCTTAGGCA 1435

LserUS96UC23_CD 1389 ---CTCTTTTTTTATACTGGCGTTCGCCTTCATAGGCATAGGCTTAGGCA 1435

LvirLAC006941_C 1401 ACCCTCTTTTTTTATACTGGCGTTCGCCTTCATAGGCATAGGCTTAGGCA 1450

LserLAC005780_C 1398 ATCCTCTTTTGGTCTACTGGCGTTCGCCTTCATAGGCATAGGCTTAGGCA 1447

LsalLAC008020_C 1398 ATCCTCTTTTGGTCTACTGGCGTTCGCCTTCATAGGCATAGGCTTAGGCA 1447

LsalCGN5271_CDS 1398 ATCCTCTTTTGGTCTACTGGCGTTCGCCTTCATAGGCATAGGCTTAGGCA 1447

LsatLACWENDEL_C 1436 TAGACATAGAAAAAATGGGGCTAGAAATAGCTTGGTGTGACCACAGGAAC 1485

LsatSalinas_CDS 1436 TAGACATAGAAAAAATGGGGCTAGAAATAGCTTGGTGTGACCACAGGAAC 1485

LserUS96UC23_CD 1436 TAGACATAGAAAAAATGGGGCTAGAAATAGCTTGGTGTGACCACAGGAAC 1485

LvirLAC006941_C 1451 TAGACATAGAAAAAATGGGGCTAGAAATAGCTTGGTGTGACCACAGGAAC 1500

LserLAC005780_C 1448 TAGACATAGAAAATCTGGGGCTAGAGGTGGCATTATGTGACCACAGGAAC 1497

LsalLAC008020_C 1448 TAGACATAGAAAATCTGGGGCTAGAGGTGGCATTATGTGACCACAGGAAC 1497

LsalCGN5271_CDS 1448 TAGACATAGAAAATCTGGGGCTAGAGGTGGCATTATGTGACCACAGGAAC 1497

LsatLACWENDEL_C 1486 GCCTTCAGCGTTCCGCAGAGCCCAGCGGCG------TCTCAGGAATTACC 1529

LsatSalinas_CDS 1486 GCCTTCAGCGTTCCGCAGAGCCCAGCGGCG------TCTCAGGAATTACC 1529

LserUS96UC23_CD 1486 GCCTTCAGCGTTCCGCAGAGCCCAGCGGCG------TCTCAGGAATTACC 1529

LvirLAC006941_C 1501 GCCTTCAGCGTTCCGCAGAGCCCAGCGGCG------TCTCAGGAATTACC 1544

LserLAC005780_C 1498 GCCTTCAGCGTTCCGCAGAGCCCAACGACGCCGCCGTCTCAGGAATTACC 1547

LsalLAC008020_C 1498 GCCTTCAGCGTTCCGCAGAGCCCAACGGCGCCGCCGTCTCAGGAATTACC 1547

LsalCGN5271_CDS 1498 GCCTTCAGCGTTCCGCAGAGCCCAACGGCGCCGCCGTCTCAGGAATTACC 1547

LsatLACWENDEL_C 1530 GGAGTCCCCCCCTCCACCACCGGTGCCCCCCGCGGTACCAACCACCGAAC 1579

LsatSalinas_CDS 1530 GGAGTCCCCCCCTCCACCACCGGTGCCCCCCGCGGTACCAACCACCGAAC 1579

LserUS96UC23_CD 1530 GGAGTCCCCCCCTCCACCACCGGTGCCCCCCGCGGTACCAACCACCGAAC 1579

LvirLAC006941_C 1545 GGAGTCCCCCCCTCCACCACCGGTGCCCCCCGCGGTACCAACCACCGAAC 1594

LserLAC005780_C 1548 TGAGTCCCCCCCTCCACCACCGATGCCCCCCGCGGTACCAATCCCGGAAC 1597

LsalLAC008020_C 1548 TGAGTCCCCCCCTCCACCACCGATGCCCCCCGCGGTACCAATCCCGGAAC 1597

LsalCGN5271_CDS 1548 TGAGTCCCCCCCTCCACCACCGATGCCCCCCGCGGTACCAATCCCGGAAC 1597

LsatLACWENDEL_C 1580 CCTTGCTGTCCGATCAAGTACGAAACAGCATCCTCTATCAAAGGTATCTT 1629

LsatSalinas_CDS 1580 CCTTGCTGTCCGATCAAGTACGAAACAGCATCCTCTATCAAAGGTATCTT 1629

LserUS96UC23_CD 1580 CCTTGCTGTCCGATCAAGTACGAAACAGCATCCTCTATCAAAGGTATCTT 1629

LvirLAC006941_C 1595 CCTTGCTGTCCGATCAAGTACGAAACAGCATCCTCTATCAAAGGTATCTT 1644

LserLAC005780_C 1598 CATTGCTTCCCGATCAAGTACGAAGCAACAGCCTATATCAAAGGTATCTT 1647

LsalLAC008020_C 1598 CATTGCTTCCCGATCAAGTACGAAGCAACAGCCTATATCAAAGGTATCTT 1647

LsalCGN5271_CDS 1598 CATTGCTTCCCGATCAAGTACGAAGCAACAGCCTATATCAAAGGTATCTT 1647

LsatLACWENDEL_C 1630 ACCCTCGATTTTGGGGTAGGGGACCCCGGCGATTTAAGCCGCATGGTTTC 1679

LsatSalinas_CDS 1630 ACCCTCGATTTTGGGGTAGGGGACCCCGGCGATTTAAGCCGCATGGTTTC 1679

LserUS96UC23_CD 1630 ACCCTCGATTTTGGGGTAGGGGACCCCGGCGATTTAAGCCGCATGGTTTC 1679

LvirLAC006941_C 1645 ACCCTCGATTTTGGGGTAGGGGACCCCGGCGATTTAAGCCGCATGGTTTC 1694

LserLAC005780_C 1648 ATCCTCGATTTTGGGGTAGGGGACCCCGGAAATTTAGAACGCATGGTTTC 1697

LsalLAC008020_C 1648 ATCCTCGATTTTGGGGTAGGGGACCCCGGAAATTTAGAAAGCATGGTTTC 1697

LsalCGN5271_CDS 1648 ATCCTCGATTTTGGGGTAGGGGACCCCGGAAATTTAGAAAGCATGGTTTC 1697

LsatLACWENDEL_C 1680 GATCATAACTAACCAATTTGTTATAGAGAGGAGTGTCGAGCAAGCCCTGC 1729

LsatSalinas_CDS 1680 GATCATAACTAACCAATTTGTTATAGAGAGGAGTGTCGAGCAAGCCCTGC 1729

LserUS96UC23_CD 1680 GATCATAACTAACCAATTTGTTATAGAGAGGAGTGTCGAGCAAGCCCTGC 1729

LvirLAC006941_C 1695 GATCATAACTAACCAATTTGTTATAGAGAGGAGTGTCGAGCAAGCCCTGC 1744

LserLAC005780_C 1698 AATCATCACGAACCAGGTCTTTATAGAGAGGAGTGTCGAGCAAGCCCTGC 1747

LsalLAC008020_C 1698 AATCATCACGAACCAGGTCTTTATAGAGAGGAGTGTCGAGCAAGCCCTGC 1747

LsalCGN5271_CDS 1698 AATCATCACGAACCAGGTCTTTATAGAGAGGAGTGTCGAGCAAGCCCTGC 1747

LsatLACWENDEL_C 1730 TCCAAGACGGCTGGAGCGCTGGTTCGATTCTAGCGCAATACACGAGTATT 1779

LsatSalinas_CDS 1730 TCCAAGACGGCTGGAGCGCTGGTTCGATTCTAGCGCAATACACGAGTATT 1779

LserUS96UC23_CD 1730 TCCAAGACGGCTGGAGCGCTGGTTCGATTCTAGCGCAATACACGAGTATT 1779

LvirLAC006941_C 1745 TCCAAGACGGCTGGAGCGCTGGTTCGATTCTAGCGCAATACACGAGTATT 1794

LserLAC005780_C 1748 TCCAAGACGGCTGGGGCGCTGGTTCGATTCTAGCGCAATACACGAGTATT 1797

LsalLAC008020_C 1748 TCCAAGACGGCTGGGGCGCTGGTTCGATTCTAGCGCAATACACGAGTATT 1797

LsalCGN5271_CDS 1748 TCCAAGACGGCTGGGGCGCTGGTTCGATTCTAGCGCAATACACGAGTATT 1797

LsatLACWENDEL_C 1780 CGTGGCGTTGTTCACACTCCACAAGGGAGACTCCTAAGTCCGAGGACTTA 1829

LsatSalinas_CDS 1780 CGTGGCGTTGTTCACACTCCACAAGGGAGACTCCTAAGTCCGAGGACTTA 1829

LserUS96UC23_CD 1780 CGTGGCGTTGTTCACACTCCACAAGGGAGACTCCTAAGTCCGAGGACTTA 1829

LvirLAC006941_C 1795 CGTGGCGTTGTTCACACTCCACAAGGGAGACTCCTAAGTCCGAGGACTTA 1844

LserLAC005780_C 1798 CGTGGCATTGTTCACACTCCACAAGGGAGACTCCTAAGTCCGAGGACTTA 1847

LsalLAC008020_C 1798 CGTGGCATTGTTCACACTCCACAAGGGAGACTCCTAAGTCCGAGGACTTA 1847

LsalCGN5271_CDS 1798 CGTGGCATTGTTCACACTCCACAAGGGAGACTCCTAAGTCCGAGGACTTA 1847

LsatLACWENDEL_C 1830 TGAGTCTTACGTGTCGCAAATAAACGAACAGGGAACCCGGCAAAGTGTGC 1879

LsatSalinas_CDS 1830 TGAGTCTTACGTGTCGCAAATAAACGAACAGGGAACCCGGCAAAGTGTGC 1879

LserUS96UC23_CD 1830 TGAGTCTTACGTGTCGCAAATAAACGAACAGGGAACCCGGCAAAGTGTGC 1879

LvirLAC006941_C 1845 TGAGTCTTACGTGTCGCAAATAAACGAACAGGGAACCCGGCAAAGTGTGC 1894

LserLAC005780_C 1848 TGAGTCTTACGTGTCGCAAATAAACGAACAGGGAACCCGGCAAAGTGTGC 1897

LsalLAC008020_C 1848 TGAGTCTTACGTGTCGCAAATAAACGAACAGGGAACCCGGCAAAGTGTGC 1897

LsalCGN5271_CDS 1848 TGAGTCTTACGTGTCGCAAATAAACGAACAGGGAACCCGGCAAAGTGTGC 1897

LsatLACWENDEL_C 1880 CCTATCGGCGGATTACACGGGCTCTGAACAATTTCGATCTGGTGTTGGAG 1929

LsatSalinas_CDS 1880 CCTATCGGCGGATTACACGGGCTCTGAACAATTTCGATCTGGTGTTGGAG 1929

LserUS96UC23_CD 1880 CCTATCGGCGGATTACACGGGCTCTGAACAATTTCGATCTGGTGTTGGAG 1929

LvirLAC006941_C 1895 CCTATCGGCGGATTACACGGGCTCTGAACAATTTCGATCTGGTGTTGGAG 1944

LserLAC005780_C 1898 CCTATCGGCGGATTTACAGAGCTCTTAACAATTTTGATCTAGTATTGGAG 1947

LsalLAC008020_C 1898 CCTATCGGCGGATTTACAGAGCTCTTAACAATTTTGATCTAGTATTGGAG 1947

LsalCGN5271_CDS 1898 CCTATCGGCGGATTTACAGAGCTCTTAACAATTTTGATCTAGTATTGGAG 1947

LsatLACWENDEL_C 1930 AGAGCAGGGGGGGGA---------------TAAATGAGATTGAGTTCCAC 1964

LsatSalinas_CDS 1930 AGAGCAGGGGGGGGA---------------TAAATGAGATTGAGTTCCAC 1964

LserUS96UC23_CD 1930 AGAGCAGGGGGGGGA---------------TAAATGAGATTGAGTTCCAC 1964

LvirLAC006941_C 1945 AGAGCAGGGGGGGGA---------------TAAATGAGATTGAGTTCCAC 1979

LserLAC005780_C 1948 AGAGCAGGGGGGGGAGAGACGAGGGGGGCTTAGATGAGATTGAGTTCCAC 1997

LsalLAC008020_C 1948 AGAGCAGGGGGGGGAGAGACGAGGGGGGCTTAGATGAGATTGAGTTCCAC 1997

LsalCGN5271_CDS 1948 AGAGCAGGGGGGGGAGAGACGAGGGGGGCTTAGATGAGATTGAGTTCCAC 1997

LsatLACWENDEL_C 1965 GAATATGCAGGCTAGAAAGATGCTATTTGCTGCTATTCTATCTATTTGTG 2014

LsatSalinas_CDS 1965 GAATATGCAGGCTAGAAAGATGCTATTTGCTGCTATTCTATCTATTTGTG 2014

LserUS96UC23_CD 1965 GAATATGCAGGCTAGAAAGATGCTATTTGCTGCTATTCTATCTATTTGTG 2014

LvirLAC006941_C 1980 GAATATGCAGGCTAGAAAGATGCTATTTGCTGCTATTCTATCTATTTGTG 2029

LserLAC005780_C 1998 GAATATGCAGGCTAGAAAGATGCTATTTGCTGCTATTCTATCTATTTGTG 2047

LsalLAC008020_C 1998 GAATATGCAGGCTAGAAAGATGCTATTTGCTGCTATTCTATCTATTTGTG 2047

LsalCGN5271_CDS 1998 GAATATGCAGGCTAGAAAGATGCTATTTGCTGCTATTCTATCTATTTGTG 2047

LsatLACWENDEL_C 2015 CATCAAGTTCGAAGAAGATCTCAATCTATAATGAAGAAATGATAGTAGCT 2064

LsatSalinas_CDS 2015 CATCAAGTTCGAAGAAGATCTCAATCTATAATGAAGAAATGATAGTAGCT 2064

LserUS96UC23_CD 2015 CATCAAGTTCGAAGAAGATCTCAATCTATAATGAAGAAATGATAGTAGCT 2064

LvirLAC006941_C 2030 CATCAAGTTCGAAGAAGATCTCAATCTATAATGAAGAAATGATAGTAGCT 2079

LserLAC005780_C 2048 CATCAAGTTCGAAGAAGATCTCAATCTATAATGAAGAAATGATAGTAGCT 2097

LsalLAC008020_C 2048 CATCAAGTTCGAAGAAGATCTCAATCTATAATGAAGAAATGATAGTAGCT 2097

LsalCGN5271_CDS 2048 CATCAAGTTCGAAGAAGATCTCAATCTATAATGAAGAAATGATAGTAGCT 2097

LsatLACWENDEL_C 2065 CGTTGTTTTATAGGCTTTATCATATTCAGTCGGAAGAGTTTAGGTAAGAC 2114

LsatSalinas_CDS 2065 CGTTGTTTTATAGGCTTTATCATATTCAGTCGGAAGAGTTTAGGTAAGAC 2114

LserUS96UC23_CD 2065 CGTTGTTTTATAGGCTTTATCATATTCAGTCGGAAGAGTTTAGGTAAGAC 2114

LvirLAC006941_C 2080 CGTTGTTTTATAGGCTTTATCATATTCAGTCGGAAGAGTTTAGGTAAGAC 2129

LserLAC005780_C 2098 CGTTGTTTTATAGGCTTTATCATATTCAGTCGGAAGAGTTTAGGTAAGAC 2147

LsalLAC008020_C 2098 CGTTGTTTTATAGGCTTTATCATATTCAGTCGGAAGAGTTTAGGTAAGAC 2147

LsalCGN5271_CDS 2098 CGTTGTTTTATAGGCTTTATCATATTCAGTCGGAAGAGTTTAGGTAAGAC 2147

LsatLACWENDEL_C 2115 TTTCAAAGAGACTCTCGACGGGAGAATCCAGGCTATTCAGGAAGAATCAC 2164

LsatSalinas_CDS 2115 TTTCAAAGAGACTCTCGACGGGAGAATCCAGGCTATTCAGGAAGAATCAC 2164

LserUS96UC23_CD 2115 TTTCAAAGAGACTCTCGACGGGAGAATCCAGGCTATTCAGGAAGAATCAC 2164

LvirLAC006941_C 2130 TTTCAAAGAGACTCTCGACGGGAGAATCCAGGCTATTCAGGAAGAATCAC 2179

LserLAC005780_C 2148 TTTCAAAGAGACTCTCGACGGGAGAATCCAGGCTATTCAGGAAGAATCAC 2197

LsalLAC008020_C 2148 TTTCAAAGAGACTCTCGACGGGAGAATCCAGGCTATTCAGGAAGAATCAC 2197

LsalCGN5271_CDS 2148 TTTCAAAGAGACTCTCGACGGGAGAATCCAGGCTATTCAGGAAGAATCAC 2197

LsatLACWENDEL_C 2165 AGCAATTCCTCAATCCTAACGAAGTAGTTCCTCCGGAATCCAATGAACAA 2214

LsatSalinas_CDS 2165 AGCAATTCCTCAATCCTAACGAAGTAGTTCCTCCGGAATCCAATGAACAA 2214

LserUS96UC23_CD 2165 AGCAATTCCTCAATCCTAACGAAGTAGTTCCTCCGGAATCCAATGAACAA 2214

LvirLAC006941_C 2180 AGCAATTCCTCAATCCTAACGAAGTAGTTCCTCCGGAATCCAATGAACAA 2229

LserLAC005780_C 2198 AGCAATTCCTCAATCCTAACGAAGTAGTTCCTCCGGAATCCAATGAACAA 2247

LsalLAC008020_C 2198 AGCAATTCCTCAATCCTAACGAAGTAGTTCCTCCGGAATCCAATGAACAA 2247

LsalCGN5271_CDS 2198 AGCAATTCCTCAATCCTAACGAAGTAGTTCCTCCGGAATCCAATGAACAA 2247

LsatLACWENDEL_C 2215 CAACGATTACTTAGGATCAGCTTGCGAATTTGTGGCACCGTAGTAGAATC 2264

LsatSalinas_CDS 2215 CAACGATTACTTAGGATCAGCTTGCGAATTTGTGGCACCGTAGTAGAATC 2264

LserUS96UC23_CD 2215 CAACGATTACTTAGGATCAGCTTGCGAATTTGTGGCACCGTAGTAGAATC 2264

LvirLAC006941_C 2230 CAACGATTACTTAGGATCAGCTTGCGAATTTGTGGCACCGTAGTAGAATC 2279

LserLAC005780_C 2248 CAACGATTACTTAGGATCAGCTTGCGAATTTGTGGCACCGTAGTAGAATC 2297

LsalLAC008020_C 2248 CAACGATTACTTAGGATCAGCTTGCGAATTTGTGGCACCGTAGTAGAATC 2297

LsalCGN5271_CDS 2248 CAACGATTACTTAGGATCAGCTTGCGAATTTGTGGCACCGTAGTAGAATC 2297

LsatLACWENDEL_C 2265 ATTACCAATGGCACGCAGTGCGCCTAAGTGCGAAAAGACAGTGCAAGCTT 2314

LsatSalinas_CDS 2265 ATTACCAATGGCACGCAGTGCGCCTAAGTGCGAAAAGACAGTGCAAGCTT 2314

LserUS96UC23_CD 2265 ATTACCAATGGCACGCAGTGCGCCTAAGTGCGAAAAGACAGTGCAAGCTT 2314

LvirLAC006941_C 2280 ATTACCAATGGCACGCAGTGCGCCTAAGTGCGAAAAGACAGTGCAAGCTT 2329

LserLAC005780_C 2298 ATTACCAATGGCACGCAGTGCGCCTAAGTGCGAAAAGACAGTGCAAGCTT 2347

LsalLAC008020_C 2298 ATTACCAATGGCACGCAGTGCGCCTAAGTGCGAAAAGACAGTGCAAGCTT 2347

LsalCGN5271_CDS 2298 ATTACCAATGGCACGCAGTGCGCCTAAGTGCGAAAAGACAGTGCAAGCTT 2347

LsatLACWENDEL_C 2315 TGTTATGCCGAAACCTAAATGTTAAGTCAGCAACACTTCCAAATGCCACT 2364

LsatSalinas_CDS 2315 TGTTATGCCGAAACCTAAATGTTAAGTCAGCAACACTTCCAAATGCCACT 2364

LserUS96UC23_CD 2315 TGTTATGCCGAAACCTAAATGTTAAGTCAGCAACACTTCCAAATGCCACT 2364

LvirLAC006941_C 2330 TGTTATGCCGAAACCTAAATGTTAAGTCAGCAACACTTCCAAATGCCACT 2379

LserLAC005780_C 2348 TGTTATGCCGAAACCTAAATGTTAAGTCAGCAACACTTCCAAATGCCACT 2397

LsalLAC008020_C 2348 TGTTATGCCGAAACCTAAATGTTAAGTCAGCAACACTTCCAAATGCCACT 2397

LsalCGN5271_CDS 2348 TGTTATGCCGAAACCTAAATGTTAAGTCAGCAACACTTCCAAATGCCACT 2397

LsatLACWENDEL_C 2365 TCTTCCCGTCGCATCCGTCTTCAGGACGATCTAGTCACAGGTTTTCACTT 2414

LsatSalinas_CDS 2365 TCTTCCCGTCGCATCCGTCTTCAGGACGATCTAGTCACAGGTTTTCACTT 2414

LserUS96UC23_CD 2365 TCTTCCCGTCGCATCCGTCTTCAGGACGATCTAGTCACAGGTTTTCACTT 2414

LvirLAC006941_C 2380 TCTTCCCGTCGCATCCGTCTTCAGGACGATCTAGTCACAGGTTTTCACTT 2429

LserLAC005780_C 2398 TCTTCCCGTCGCATCCGTCTTCAGGACGATCTAGTCACAGGTTTTCACTT 2447

LsalLAC008020_C 2398 TCTTCCCGTCGCATCCGTCTTCAGGACGATCTAGTCACAGGTTTTCACTT 2447

LsalCGN5271_CDS 2398 TCTTCCCGTCGCATCCGTCTTCAGGACGATCTAGTCACAGGTTTTCACTT 2447

LsatLACWENDEL_C 2415 CTCAGTGAGTGAAAGATTTGTCCCCGGGTCTACGTTGAAAGCTTCTATAG 2464

LsatSalinas_CDS 2415 CTCAGTGAGTGAAAGATTTGTCCCCGGGTCTACGTTGAAAGCTTCTATAG 2464

LserUS96UC23_CD 2415 CTCAGTGAGTGAAAGATTTGTCCCCGGGTCTACGTTGAAAGCTTCTATAG 2464

LvirLAC006941_C 2430 CTCAGTGAGTGAAAGATTTGTCCCCGGGTCTACGTTGAAAGCTTCTATAG 2479

LserLAC005780_C 2448 CTCAGTGAGTGAAAGATTTGTCCCCGGGTCTACGTTGAAAGCTTCTATAG 2497

LsalLAC008020_C 2448 CTCAGTGAGTGAAAGATTTGTCCCCGGGTCTACGTTGAAAGCTTCTATAG 2497

LsalCGN5271_CDS 2448 CTCAGTGAGTGAAAGATTTGTCCCCGGGTCTACGTTGAAAGCTTCTATAG 2497

LsatLACWENDEL_C 2465 TAGAACTCATTCGGGAGGGCTTGGTGGTCTTAAGAATGGTTCGGGTGGGG 2514

LsatSalinas_CDS 2465 TAGAACTCATTCGGGAGGGCTTGGTGGTCTTAAGAATGGTTCGGGTGGGG 2514

LserUS96UC23_CD 2465 TAGAACTCATTCGGGAGGGCTTGGTGGTCTTAAGAATGGTTCGGGTGGGG 2514

LvirLAC006941_C 2480 TAGAACTCATTCGGGAGGGCTTGGTGGTCTTAAGAATGGTTCGGGTGGGG 2529

LserLAC005780_C 2498 TAGAACTCATTCGGGAGGGCTTGGTGGTCTTAAGAATGGTTCGGGTGGGG 2547

LsalLAC008020_C 2498 TAGAACTCATTCGGGAGGGCTTGGTGGTCTTAAGAATGGTTCGGGTGGGG 2547

LsalCGN5271_CDS 2498 TAGAACTCATTCGGGAGGGCTTGGTGGTCTTAAGAATGGTTCGGGTGGGG 2547

LsatLACWENDEL_C 2515 GCTGAATAAATGAAGATAGGAAACTTGTATTTCTCATTCACAAATTCATC 2564

LsatSalinas_CDS 2515 GCTGAATAAATGAAGATAGGAAACTTGTATTTCTCATTCACAAATTCATC 2564

LserUS96UC23_CD 2515 GCTGAATAAATGAAGATAGGAAACTTGTATTTCTCATTCACAAATTCATC 2564

LvirLAC006941_C 2530 GCTGAATAAATGAAGATAGGAAACTTGTATTTCTCATTCACAAATTCATC 2579

LserLAC005780_C 2548 GCTGAATAAATGAAGATAGGAAACTTGTATTTCTCATTCACAAATTCATC 2597

LsalLAC008020_C 2548 GCTGAATAAATGAAGATAGGAAACTTGTATTTCTCATTCACAAATTCATC 2597

LsalCGN5271_CDS 2548 GCTGAATAAATGAAGATAGGAAACTTGTATTTCTCATTCACAAATTCATC 2597

LsatLACWENDEL_C 2565 TTTGTTTATGCTGCTAACTCTCAGTTTGGTCCTACTTCTGATTCATTTTG 2614

LsatSalinas_CDS 2565 TTTGTTTATGCTGCTAACTCTCAGTTTGGTCCTACTTCTGATTCATTTTG 2614

LserUS96UC23_CD 2565 TTTGTTTATGCTGCTAACTCTCAGTTTGGTCCTACTTCTGATTCATTTTG 2614

LvirLAC006941_C 2580 TTTGTTTATGCTGCTAACTCTCAGTTTGGTCCTACTTCTGATTCATTTTG 2629

LserLAC005780_C 2598 TTTGTTTATGCTGCTAACTCTCAGTTTGGTCCTACTTCTGATTCATTTTG 2647

LsalLAC008020_C 2598 TTTGTTTATGCTGCTAACTCTCAGTTTGGTCCTACTTCTGATTCATTTTG 2647

LsalCGN5271_CDS 2598 TTTGTTTATGCTGCTAACTCTCAGTTTGGTCCTACTTCTGATTCATTTTG 2647

LsatLACWENDEL_C 2615 TTACAAAAAAAGGAGGAGGAAACTTAGTACCAAATGCTTGGCAATCCTTG 2664

LsatSalinas_CDS 2615 TTACAAAAAAAGGAGGAGGAAACTTAGTACCAAATGCTTGGCAATCCTTG 2664

LserUS96UC23_CD 2615 TTACAAAAAAAGGAGGAGGAAACTTAGTACCAAATGCTTGGCAATCCTTG 2664

LvirLAC006941_C 2630 TTACAAAAAAAGGAGGAGGAAACTTAGTACCAAATGCTTGGCAATCCTTG 2679

LserLAC005780_C 2648 TTACAAAAAAAGGAGGAGGAAACTTAGTACCAAATGCTTGGCAATCCTTG 2697

LsalLAC008020_C 2648 TTACAAAAAAAGGAGGAGGAAACTTAGTACCAAATGCTTGGCAATCCTTG 2697

LsalCGN5271_CDS 2648 TTACAAAAAAAGGAGGAGGAAACTTAGTACCAAATGCTTGGCAATCCTTG 2697

LsatLACWENDEL_C 2665 GTAGAGCTTATTTATGATTTCGTGCTTAACCTGGTAAACGAACAAATAGG 2714

LsatSalinas_CDS 2665 GTAGAGCTTATTTATGATTTCGTGCTTAACCTGGTAAACGAACAAATAGG 2714

LserUS96UC23_CD 2665 GTAGAGCTTATTTATGATTTCGTGCTTAACCTGGTAAACGAACAAATAGG 2714

LvirLAC006941_C 2680 GTAGAGCTTATTTATGATTTCGTGCTTAACCTGGTAAACGAACAAATAGG 2729

LserLAC005780_C 2698 GTAGAGCTTATTTATGATTTCGTGCTTAACCTGGTAAACGAACAAATAGG 2747

LsalLAC008020_C 2698 GTAGAGCTTATTTATGATTTCGTGCTTAACCTGGTAAACGAACAAATAGG 2747

LsalCGN5271_CDS 2698 GTAGAGCTTATTTATGATTTCGTGCTTAACCTGGTAAACGAACAAATAGG 2747

LsatLACWENDEL_C 2715 GGGTCTTTCCGGAAATGTTAAACAAAAGTTTTTCCCTTGCATCTTGGTCA 2764

LsatSalinas_CDS 2715 GGGTCTTTCCGGAAATGTTAAACAAAAGTTTTTCCCTTGCATCTTGGTCA 2764

LserUS96UC23_CD 2715 GGGTCTTTCCGGAAATGTTAAACAAAAGTTTTTCCCTTGCATCTTGGTCA 2764

LvirLAC006941_C 2730 GGGTCTTTCCGGAAATGTTAAACAAAAGTTTTTCCCTTGCATCTTGGTCA 2779

LserLAC005780_C 2748 GGGTCTTTCCGGAAATGTTAAACAAAAGTTTTTCCCTTGCATCTTGGTCA 2797

LsalLAC008020_C 2748 GGGTCTTTCCGGAAATGTTAAACAAAAGTTTTTCCCTTGCATCTTGGTCA 2797

LsalCGN5271_CDS 2748 GGGTCTTTCCGGAAATGTTAAACAAAAGTTTTTCCCTTGCATCTTGGTCA 2797

LsatLACWENDEL_C 2765 CTTTTACTTTTTTGTTATTTTGTAATCTTCAGGGTATGATACCTTATAGC 2814

LsatSalinas_CDS 2765 CTTTTACTTTTTTGTTATTTTGTAATCTTCAGGGTATGATACCTTATAGC 2814

LserUS96UC23_CD 2765 CTTTTACTTTTTTGTTATTTTGTAATCTTCAGGGTATGATACCTTATAGC 2814

LvirLAC006941_C 2780 CTTTTACTTTTTTGTTATTTTGTAATCTTCAGGGTATGATACCTTATAGC 2829

LserLAC005780_C 2798 CTTTTACTTTTTTGTTATTTTGTAATCTTCAGGGTATGATACCTTATAGC 2847

LsalLAC008020_C 2798 CTTTTACTTTTTTGTTATTTTGTAATCTTCAGGGTATGATACCTTATAGC 2847

LsalCGN5271_CDS 2798 CTTTTACTTTTTTGTTATTTTGTAATCTTCAGGGTATGATACCTTATAGC 2847

LsatLACWENDEL_C 2815 TTCACAGTTACAAGTCATTTTCTCATTACTTTAGGTCTCTCATTTTCCAT 2864

LsatSalinas_CDS 2815 TTCACAGTTACAAGTCATTTTCTCATTACTTTAGGTCTCTCATTTTCCAT 2864

LserUS96UC23_CD 2815 TTCACAGTTACAAGTCATTTTCTCATTACTTTAGGTCTCTCATTTTCCAT 2864

LvirLAC006941_C 2830 TTCACAGTTACAAGTCATTTTCTCATTACTTTAGGTCTCTCATTTTCCAT 2879

LserLAC005780_C 2848 TTCACAGTTACAAGTCATTTTCTCATTACTTTAGGTCTCTCATTTTCCAT 2897

LsalLAC008020_C 2848 TTCACAGTTACAAGTCATTTTCTCATTACTTTAGGTCTCTCATTTTCCAT 2897

LsalCGN5271_CDS 2848 TTCACAGTTACAAGTCATTTTCTCATTACTTTAGGTCTCTCATTTTCCAT 2897

LsatLACWENDEL_C 2865 TTTTATTGGCATTACTATAGTAGGATTTCAAAGAAATGGGCTTCATTTTT 2914

LsatSalinas_CDS 2865 TTTTATTGGCATTACTATAGTAGGATTTCAAAGAAATGGGCTTCATTTTT 2914

LserUS96UC23_CD 2865 TTTTATTGGCATTACTATAGTAGGATTTCAAAGAAATGGGCTTCATTTTT 2914

LvirLAC006941_C 2880 TTTTATTGGCATTACTATAGTAGGATTTCAAAGAAATGGGCTTCATTTTT 2929

LserLAC005780_C 2898 TTTTATTGGCATTACTATAGTAGGATTTCAAAGAAATGGGCTTCATTTTT 2947

LsalLAC008020_C 2898 TTTTATTGGCATTACTATAGTAGGATTTCAAAGAAATGGGCTTCATTTTT 2947

LsalCGN5271_CDS 2898 TTTTATTGGCATTACTATAGTAGGATTTCAAAGAAATGGGCTTCATTTTT 2947

LsatLACWENDEL_C 2915 TAAGCTTCTTATTACCCGCAGGAGTCCCACTGCCGTTAGCACCTTTTTTA 2964

LsatSalinas_CDS 2915 TAAGCTTCTTATTACCCGCAGGAGTCCCACTGCCGTTAGCACCTTTTTTA 2964

LserUS96UC23_CD 2915 TAAGCTTCTTATTACCCGCAGGAGTCCCACTGCCGTTAGCACCTTTTTTA 2964

LvirLAC006941_C 2930 TAAGCTTCTTATTACCCGCAGGAGTCCCACTGCCGTTAGCACCTTTTTTA 2979

LserLAC005780_C 2948 TAAGCTTCTTATTACCCGCAGGAGTCCCACTGCCGTTAGCACCTTTTTTA 2997

LsalLAC008020_C 2948 TAAGCTTCTTATTACCCGCAGGAGTCCCACTGCCGTTAGCACCTTTTTTA 2997

LsalCGN5271_CDS 2948 TAAGCTTCTTATTACCCGCAGGAGTCCCACTGCCGTTAGCACCTTTTTTA 2997

LsatLACWENDEL_C 2965 GTACTCCTTGAGCTAATTTCTTATTGTTTTCGCGCATTAAGCTTAGGAAT 3014

LsatSalinas_CDS 2965 GTACTCCTTGAGCTAATTTCTTATTGTTTTCGCGCATTAAGCTTAGGAAT 3014

LserUS96UC23_CD 2965 GTACTCCTTGAGCTAATTTCTTATTGTTTTCGCGCATTAAGCTTAGGAAT 3014

LvirLAC006941_C 2980 GTACTCCTTGAGCTAATTTCTTATTGTTTTCGCGCATTAAGCTTAGGAAT 3029

LserLAC005780_C 2998 GTACTCCTTGAGCTAATTTCTTATTGTTTTCGCGCATTAAGCTTAGGAAT 3047

LsalLAC008020_C 2998 GTACTCCTTGAGCTAATTTCTTATTGTTTTCGCGCATTAAGCTTAGGAAT 3047

LsalCGN5271_CDS 2998 GTACTCCTTGAGCTAATTTCTTATTGTTTTCGCGCATTAAGCTTAGGAAT 3047

LsatLACWENDEL_C 3015 ACGTTTATTTGCTAATATGATGGCCGGTCATAGTTTAGTAAAGATTTTAA 3064

LsatSalinas_CDS 3015 ACGTTTATTTGCTAATATGATGGCCGGTCATAGTTTAGTAAAGATTTTAA 3064

LserUS96UC23_CD 3015 ACGTTTATTTGCTAATATGATGGCCGGTCATAGTTTAGTAAAGATTTTAA 3064

LvirLAC006941_C 3030 ACGTTTATTTGCTAATATGATGGCCGGTCATAGTTTAGTAAAGATTTTAA 3079

LserLAC005780_C 3048 ACGTTTATTTGCTAATATGATGGCCGGTCATAGTTTAGTAAAGATTTTAA 3097

LsalLAC008020_C 3048 ACGTTTATTTGCTAATATGATGGCCGGTCATAGTTTAGTAAAGATTTTAA 3097

LsalCGN5271_CDS 3048 ACGTTTATTTGCTAATATGATGGCCGGTCATAGTTTAGTAAAGATTTTAA 3097

LsatLACWENDEL_C 3065 GTGGGTTCGCTTGGACTATGCTATGTATGAATGATATTTTGTTTTTTATA 3114

LsatSalinas_CDS 3065 GTGGGTTCGCTTGGACTATGCTATGTATGAATGATATTTTGTTTTTTATA 3114

LserUS96UC23_CD 3065 GTGGGTTCGCTTGGACTATGCTATGTATGAATGATATTTTGTTTTTTATA 3114

LvirLAC006941_C 3080 GTGGGTTCGCTTGGACTATGCTATGTATGAATGATATTTTGTTTTTTATA 3129

LserLAC005780_C 3098 GTGGGTTCGCTTGGACTATGCTATGTATGAATGATATTTTGTTTTTTATA 3147

LsalLAC008020_C 3098 GTGGGTTCGCTTGGACTATGCTATGTATGAATGATATTTTGTTTTTTATA 3147

LsalCGN5271_CDS 3098 GTGGGTTCGCTTGGACTATGCTATGTATGAATGATATTTTGTTTTTTATA 3147

LsatLACWENDEL_C 3115 GGGGATCTTGGTCCTTTATTTATAGTTCTTGCATTAACCGGTCTGGAATT 3164

LsatSalinas_CDS 3115 GGGGATCTTGGTCCTTTATTTATAGTTCTTGCATTAACCGGTCTGGAATT 3164

LserUS96UC23_CD 3115 GGGGATCTTGGTCCTTTATTTATAGTTCTTGCATTAACCGGTCTGGAATT 3164

LvirLAC006941_C 3130 GGGGATCTTGGTCCTTTATTTATAGTTCTTGCATTAACCGGTCTGGAATT 3179

LserLAC005780_C 3148 GGGGATCTTGGTCCTTTATTTATAGTTCTTGCATTAACCGGTCTGGAATT 3197

LsalLAC008020_C 3148 GGGGATCTTGGTCCTTTATTTATAGTTCTTGCATTAACCGGTCTGGAATT 3197

LsalCGN5271_CDS 3148 GGGGATCTTGGTCCTTTATTTATAGTTCTTGCATTAACCGGTCTGGAATT 3197

LsatLACWENDEL_C 3165 AGGTGTAGCTATATTACAAGCTTATGTTTTTACGATCTTAATCTGTATTT 3214

LsatSalinas_CDS 3165 AGGTGTAGCTATATTACAAGCTTATGTTTTTACGATCTTAATCTGTATTT 3214

LserUS96UC23_CD 3165 AGGTGTAGCTATATTACAAGCTTATGTTTTTACGATCTTAATCTGTATTT 3214

LvirLAC006941_C 3180 AGGTGTAGCTATATTACAAGCTTATGTTTTTACGATCTTAATCTGTATTT 3229

LserLAC005780_C 3198 AGGTGTAGCTATATTACAAGCTTATGTTTTTACGATCTTAATCTGTATTT 3247

LsalLAC008020_C 3198 AGGTGTAGCTATATTACAAGCTTATGTTTTTACGATCTTAATCTGTATTT 3247

LsalCGN5271_CDS 3198 AGGTGTAGCTATATTACAAGCTTATGTTTTTACGATCTTAATCTGTATTT 3247

LsatLACWENDEL_C 3215 ACTTGAATGATGCTATAAATCTCCATTAAATGCCTCAACTAGATCAATTC 3264

LsatSalinas_CDS 3215 ACTTGAATGATGCTATAAATCTCCATTAAATGCCTCAACTAGATCAATTC 3264

LserUS96UC23_CD 3215 ACTTGAATGATGCTATAAATCTCCATTAAATGCCTCAACTAGATCAATTC 3264

LvirLAC006941_C 3230 ACTTGAATGATGCTATAAATCTCCATTAAATGCCTCAACTAGATCAATTC 3279

LserLAC005780_C 3248 ACTTGAATGATGCTATAAATCTCCATTAAATGCCTCAACTAGATCAATTC 3297

LsalLAC008020_C 3248 ACTTGAATGATGCTATAAATCTCCATTAAATGCCTCAACTAGATCAATTC 3297

LsalCGN5271_CDS 3248 ACTTGAATGATGCTATAAATCTCCATTAAATGCCTCAACTAGATCAATTC 3297

LsatLACWENDEL_C 3265 ACTTATTTCACACAATTCTTCTGGTCATGCCTTTTCCTCTTTACTTTCTA 3314

LsatSalinas_CDS 3265 ACTTATTTCACACAATTCTTCTGGTCATGCCTTTTCCTCTTTACTTTCTA 3314

LserUS96UC23_CD 3265 ACTTATTTCACACAATTCTTCTGGTCATGCCTTTTCCTCTTTACTTTCTA 3314

LvirLAC006941_C 3280 ACTTATTTCACACAATTCTTCTGGTCATGCCTTTTCCTCTTTACTTTCTA 3329

LserLAC005780_C 3298 ACTTATTTCACACAATTCTTCTGGTCATGCCTTTTCCTCTTTACTTTCTA 3347

LsalLAC008020_C 3298 ACTTATTTCACACAATTCTTCTGGTCATGCCTTTTCCTCTTTACTTTCTA 3347

LsalCGN5271_CDS 3298 ACTTATTTCACACAATTCTTCTGGTCATGCCTTTTCCTCTTTACTTTCTA 3347

LsatLACWENDEL_C 3315 TATTGCCATATGCAATGATGGAGATGGACTACTTGGGATCAGCAGAATTC 3364

LsatSalinas_CDS 3315 TATTGCCATATGCAATGATGGAGATGGACTACTTGGGATCAGCAGAATTC 3364

LserUS96UC23_CD 3315 TATTGCCATATGCAATGATGGAGATGGACTACTTGGGATCAGCAGAATTC 3364

LvirLAC006941_C 3330 TATTGCCATATGCAATGATGGAGATGGACTACTTGGGATCAGCAGAATTC 3379

LserLAC005780_C 3348 TATTGCCATATGCAATGATGGAGATGGACTACTTGGGATCAGCAGAATTC 3397

LsalLAC008020_C 3348 TATTGCCATATGCAATGATGGAGATGGACTACTTGGGATCAGCAGAATTC 3397

LsalCGN5271_CDS 3348 TATTGCCATATGCAATGATGGAGATGGACTACTTGGGATCAGCAGAATTC 3397

LsatLACWENDEL_C 3365 TAAAACTACGTAACCAACTGCTTTCACACCGTACGAACAACATCCGGAGC 3414

LsatSalinas_CDS 3365 TAAAACTACGTAACCAACTGCTTTCACACCGTACGAACAACATCCGGAGC 3414

LserUS96UC23_CD 3365 TAAAACTACGTAACCAACTGCTTTCACACCGTACGAACAACATCCGGAGC 3414

LvirLAC006941_C 3380 TAAAACTACGTAACCAACTGCTTTCACACCGTACGAACAACATCCGGAGC 3429

LserLAC005780_C 3398 TAAAACTACGTAACCAACTGCTTTCACACCGTACGAACAACATCCGGAGC 3447

LsalLAC008020_C 3398 TAAAACTACGTAACCAACTGCTTTCACACCGTACGAACAACATCCGGAGC 3447

LsalCGN5271_CDS 3398 TAAAACTACGTAACCAACTGCTTTCACACCGTACGAACAACATCCGGAGC 3447

LsatLACWENDEL_C 3415 AAGGAGCCCAACAGTTTGGAAGATATCTTGAGAAAAGGTTTTAGCACCGG 3464

LsatSalinas_CDS 3415 AAGGAGCCCAACAGTTTGGAAGATATCTTGAGAAAAGGTTTTAGCACCGG 3464

LserUS96UC23_CD 3415 AAGGAGCCCAACAGTTTGGAAGATATCTTGAGAAAAGGTTTTAGCACCGG 3464

LvirLAC006941_C 3430 AAGGAGCCCAACAGTTTGGAAGATATCTTGAGAAAAGGTTTTAGCACCGG 3479

LserLAC005780_C 3448 AAGGAGCCCAACAGTTTGGAAGATATCTTGAGAAAAGGTTTTAGCACCGG 3497

LsalLAC008020_C 3448 AAGGAGCCCAACAGTTTGGAAGATATCTTGAGAAAAGGTTTTAGCACCGG 3497

LsalCGN5271_CDS 3448 AAGGAGCCCAACAGTTTGGAAGATATCTTGAGAAAAGGTTTTAGCACCGG 3497

LsatLACWENDEL_C 3465 TCTATCCTATATGTACTCCAGTTTATTCGAAGTCTCCCAATGGTGTAAGG 3514

LsatSalinas_CDS 3465 TCTATCCTATATGTACTCCAGTTTATTCGAAGTCTCCCAATGGTGTAAGG 3514

LserUS96UC23_CD 3465 TCTATCCTATATGTACTCCAGTTTATTCGAAGTCTCCCAATGGTGTAAGG 3514

LvirLAC006941_C 3480 TCTATCCTATATGTACTCCAGTTTATTCGAAGTCTCCCAATGGTGTAAGG 3529

LserLAC005780_C 3498 TCTATCCTATATGTACTCCAGTTTATTCGAAGTCTCCCAATGGTGTAAGG 3547

LsalLAC008020_C 3498 TCTATCCTATATGTACTCCAGTTTATTCGAAGTCTCCCAATGGTGTAAGG 3547

LsalCGN5271_CDS 3498 TCTATCCTATATGTACTCCAGTTTATTCGAAGTCTCCCAATGGTGTAAGG 3547

LsatLACWENDEL_C 3515 CCGTCGACTTATTGGGAAAAAGGAGGAAGATCACTTTGATCTCTTGTTTC 3564

LsatSalinas_CDS 3515 CCGTCGACTTATTGGGAAAAAGGAGGAAGATCACTTTGATCTCTTGTTTC 3564

LserUS96UC23_CD 3515 CCGTCGACTTATTGGGAAAAAGGAGGAAGATCACTTTGATCTCTTGTTTC 3564

LvirLAC006941_C 3530 CCGTCGACTTATTGGGAAAAAGGAGGAAGATCACTTTGATCTCTTGTTTC 3579

LserLAC005780_C 3548 CCGTCGACTTATTGGGAAAAAGGAGGAAGATCACTTTGATCTCTTGTTTC 3597

LsalLAC008020_C 3548 CCGTCGACTTATTGGGAAAAAGGAGGAAGATCACTTTGATCTCTTGTTTC 3597

LsalCGN5271_CDS 3548 CCGTCGACTTATTGGGAAAAAGGAGGAAGATCACTTTGATCTCTTGTTTC 3597

LsatLACWENDEL_C 3565 GGAGAAATCAGTGGCTCACGAGGAATGGAAAGGAACATCTTCTATTTGAT 3614

LsatSalinas_CDS 3565 GGAGAAATCAGTGGCTCACGAGGAATGGAAAGGAACATCTTCTATTTGAT 3614

LserUS96UC23_CD 3565 GGAGAAATCAGTGGCTCACGAGGAATGGAAAGGAACATCTTCTATTTGAT 3614

LvirLAC006941_C 3580 GGAGAAATCAGTGGCTCACGAGGAATGGAAAGGAACATCTTCTATTTGAT 3629

LserLAC005780_C 3598 GGAGAAATCAGTGGCTCACGAGGAATGGAAAGGAACATCTTCTATTTGAT 3647

LsalLAC008020_C 3598 GGAGAAATCAGTGGCTCACGAGGAATGGAAAGGAACATCTTCTATTTGAT 3647

LsalCGN5271_CDS 3598 GGAGAAATCAGTGGCTCACGAGGAATGGAAAGGAACATCTTCTATTTGAT 3647

LsatLACWENDEL_C 3615 CTCGAAGTCCTCATATAGCACTTCTTCCAATCCTGGATGGGGGATCACTT 3664

LsatSalinas_CDS 3615 CTCGAAGTCCTCATATAGCACTTCTTCCAATCCTGGATGGGGGATCACTT 3664

LserUS96UC23_CD 3615 CTCGAAGTCCTCATATAGCACTTCTTCCAATCCTGGATGGGGGATCACTT 3664

LvirLAC006941_C 3630 CTCGAAGTCCTCATATAGCACTTCTTCCAATCCTGGATGGGGGATCACTT 3679

LserLAC005780_C 3648 CTCGAAGTCCTCATATAGCACTTCTTCCAATCCTGGATGGGGGATCACTT 3697

LsalLAC008020_C 3648 CTCGAAGTCCTCATATAGCACTTCTTCCAATCCTGGATGGGGGATCACTT 3697

LsalCGN5271_CDS 3648 CTCGAAGTCCTCATATAGCACTTCTTCCAATCCTGGATGGGGGATCACTT 3697

LsatLACWENDEL_C 3665 GTAGGAATGACATAATGCTAATCCATGTTCCACACGGCCAAGGAAGCATC 3714

LsatSalinas_CDS 3665 GTAGGAATGACATAATGCTAATCCATGTTCCACACGGCCAAGGAAGCATC 3714

LserUS96UC23_CD 3665 GTAGGAATGACATAATGCTAATCCATGTTCCACACGGCCAAGGAAGCATC 3714

LvirLAC006941_C 3680 GTAGGAATGACATAATGCTAATCCATGTTCCACACGGCCAAGGAAGCATC 3729

LserLAC005780_C 3698 GTAGGAATGACATAATGCTAATCCATGTTCCACACGGCCAAGGAAGCATC 3747

LsalLAC008020_C 3698 GTAGGAATGACATAATGCTAATCCATGTTCCACACGGCCAAGGAAGCATC 3747

LsalCGN5271_CDS 3698 GTAGGAATGACATAATGCTAATCCATGTTCCACACGGCCAAGGAAGCATC 3747

LsatLACWENDEL_C 3715 GGTTTTTAAATGTTAGAAGGTGCAAAACTAATAGGGGCCGGAGCTGCTAC 3764

LsatSalinas_CDS 3715 GGTTTTTAAATGTTAGAAGGTGCAAAACTAATAGGGGCCGGAGCTGCTAC 3764

LserUS96UC23_CD 3715 GGTTTTTAAATGTTAGAAGGTGCAAAACTAATAGGGGCCGGAGCTGCTAC 3764

LvirLAC006941_C 3730 GGTTTTTAAATGTTAGAAGGTGCAAAACTAATAGGGGCCGGAGCTGCTAC 3779

LserLAC005780_C 3748 GGTTTTTAAATGTTAGAAGGTGCAAAACTAATAGGGGCCGGAGCTGCTAC 3797

LsalLAC008020_C 3748 GGTTTTTAAATGTTAGAAGGTGCAAAACTAATAGGGGCCGGAGCTGCTAC 3797

LsalCGN5271_CDS 3748 GGTTTTTAAATGTTAGAAGGTGCAAAACTAATAGGGGCCGGAGCTGCTAC 3797

LsatLACWENDEL_C 3765 AATTGCTTCAGCGGGAGCTGCTATCGGTATTGGAAACGTCCTTAGTTCCT 3814

LsatSalinas_CDS 3765 AATTGCTTCAGCGGGAGCTGCTATCGGTATTGGAAACGTCCTTAGTTCCT 3814

LserUS96UC23_CD 3765 AATTGCTTCAGCGGGAGCTGCTATCGGTATTGGAAACGTCCTTAGTTCCT 3814

LvirLAC006941_C 3780 AATTGCTTCAGCGGGAGCTGCTATCGGTATTGGAAACGTCCTTAGTTCCT 3829

LserLAC005780_C 3798 AATTGCTTCAGCGGGAGCTGCTATCGGTATTGGAAACGTCCTTAGTTCCT 3847

LsalLAC008020_C 3798 AATTGCTTCAGCGGGAGCTGCTATCGGTATTGGAAACGTCCTTAGTTCCT 3847

LsalCGN5271_CDS 3798 AATTGCTTCAGCGGGAGCTGCTATCGGTATTGGAAACGTCCTTAGTTCCT 3847

LsatLACWENDEL_C 3815 CGATTCATTCCGTGGCTCGGAATCCATCATTGGCTAAACAATCATTTGGT 3864

LsatSalinas_CDS 3815 CGATTCATTCCGTGGCTCGGAATCCATCATTGGCTAAACAATCATTTGGT 3864

LserUS96UC23_CD 3815 CGATTCATTCCGTGGCTCGGAATCCATCATTGGCTAAACAATCATTTGGT 3864

LvirLAC006941_C 3830 CGATTCATTCCGTGGCTCGGAATCCATCATTGGCTAAACAATCATTTGGT 3879

LserLAC005780_C 3848 CGATTCATTCCGTGGCTCGGAATCCATCATTGGCTAAACAATCATTTGGT 3897

LsalLAC008020_C 3848 CGATTCATTCCGTGGCTCGGAATCCATCATTGGCTAAACAATCATTTGGT 3897

LsalCGN5271_CDS 3848 CGATTCATTCCGTGGCTCGGAATCCATCATTGGCTAAACAATCATTTGGT 3897

LsatLACWENDEL_C 3865 TATGCCATTTTGGGCTTTGCTCTAACCGAAGCTATTGCATCGTTTGCCCC 3914

LsatSalinas_CDS 3865 TATGCCATTTTGGGCTTTGCTCTAACCGAAGCTATTGCATCGTTTGCCCC 3914

LserUS96UC23_CD 3865 TATGCCATTTTGGGCTTTGCTCTAACCGAAGCTATTGCATCGTTTGCCCC 3914

LvirLAC006941_C 3880 TATGCCATTTTGGGCTTTGCTCTAACCGAAGCTATTGCATCGTTTGCCCC 3929

LserLAC005780_C 3898 TATGCCATTTTGGGCTTTGCTCTAACCGAAGCTATTGCATCGTTTGCCCC 3947

LsalLAC008020_C 3898 TATGCCATTTTGGGCTTTGCTCTAACCGAAGCTATTGCATCGTTTGCCCC 3947

LsalCGN5271_CDS 3898 TATGCCATTTTGGGCTTTGCTCTAACCGAAGCTATTGCATCGTTTGCCCC 3947

LsatLACWENDEL_C 3915 AATGATGGCCTTTCTGATCTCATCCGTATTCCGATCAAAGAATCAAAGAA 3964

LsatSalinas_CDS 3915 AATGATGGCCTTTCTGATCTCATCCGTATTCCGATCAAAGAATCAAAGAA 3964

LserUS96UC23_CD 3915 AATGATGGCCTTTCTGATCTCATCCGTATTCCGATCAAAGAATCAAAGAA 3964

LvirLAC006941_C 3930 AATGATGGCCTTTCTGATCTCATCCGTATTCCGATCAAAGAATCAAAGAA 3979

LserLAC005780_C 3948 AATGATGGCCTTTCTGATCTCATCCGTATTCCGATCAAAGAATCAAAGAA 3997

LsalLAC008020_C 3948 AATGATGGCCTTTCTGATCTCATCCGTATTCCGATCAAAGAATCAAAGAA 3997

LsalCGN5271_CDS 3948 AATGATGGCCTTTCTGATCTCATCCGTATTCCGATCAAAGAATCAAAGAA 3997

LsatLACWENDEL_C 3965 AGAAGGTTTCAGTTTCATAAATGAGACGACTCTTTCTTGAACTATATTAT 4014

LsatSalinas_CDS 3965 AGAAGGTTTCAGTTTCATAAATGAGACGACTCTTTCTTGAACTATATTAT 4014

LserUS96UC23_CD 3965 AGAAGGTTTCAGTTTCATAAATGAGACGACTCTTTCTTGAACTATATTAT 4014

LvirLAC006941_C 3980 AGAAGGTTTCAGTTTCATAAATGAGACGACTCTTTCTTGAACTATATTAT 4029

LserLAC005780_C 3998 AGAAGGTTTCAGTTTCATAAATGAGACGACTCTTTCTTGAACTATATTAT 4047

LsalLAC008020_C 3998 AGAAGGTTTCAGTTTCATAAATGAGACGACTCTTTCTTGAACTATATTAT 4047

LsalCGN5271_CDS 3998 AGAAGGTTTCAGTTTCATAAATGAGACGACTCTTTCTTGAACTATATTAT 4047

LsatLACWENDEL_C 4015 AAACAGACCTTCCCCTCCACACCAATCACGAGTTTTTCTCCATTCCTCTC 4064

LsatSalinas_CDS 4015 AAACAGACCTTCCCCTCCACACCAATCACGAGTTTTTCTCCATTCCTCTC 4064

LserUS96UC23_CD 4015 AAACAGACCTTCCCCTCCACACCAATCACGAGTTTTTCTCCATTCCTCTC 4064

LvirLAC006941_C 4030 AAACAGACCTTCCCCTCCACACCAATCACGAGTTTTTCTCCATTCCTCTC 4079

LserLAC005780_C 4048 AAACAGACCTTCCCCTCCACACCAATCACGAGTTTTTCTCCATTCCTCTC 4097

LsalLAC008020_C 4048 AAACAGACCTTCCCCTCCACACCAATCACGAGTTTTTCTCCATTCCTCTC 4097

LsalCGN5271_CDS 4048 AAACAGACCTTCCCCTCCACACCAATCACGAGTTTTTCTCCATTCCTCTC 4097

LsatLACWENDEL_C 4065 ATATATCGTCGTAACGCCCTTAATGCTAGGTTTTGAAAAAGACTTTTCAT 4114

LsatSalinas_CDS 4065 ATATATCGTCGTAACGCCCTTAATGCTAGGTTTTGAAAAAGACTTTTCAT 4114

LserUS96UC23_CD 4065 ATATATCGTCGTAACGCCCTTAATGCTAGGTTTTGAAAAAGACTTTTCAT 4114

LvirLAC006941_C 4080 ATATATCGTCGTAACGCCCTTAATGCTAGGTTTTGAAAAAGACTTTTCAT 4129

LserLAC005780_C 4098 ATATATCGTCGTAACGCCCTTAATGCTAGGTTTTGAAAAAGACTTTTCAT 4147

LsalLAC008020_C 4098 ATATATCGTCGTAACGCCCTTAATGCTAGGTTTTGAAAAAGACTTTTCAT 4147

LsalCGN5271_CDS 4098 ATATATCGTCGTAACGCCCTTAATGCTAGGTTTTGAAAAAGACTTTTCAT 4147

LsatLACWENDEL_C 4115 GTCATTCCCATTTAGGTCCGATTCGGATCCCTCCGTTGTTTCCTTTTCCT 4164

LsatSalinas_CDS 4115 GTCATTCCCATTTAGGTCCGATTCGGATCCCTCCGTTGTTTCCTTTTCCT 4164

LserUS96UC23_CD 4115 GTCATTCCCATTTAGGTCCGATTCGGATCCCTCCGTTGTTTCCTTTTCCT 4164

LvirLAC006941_C 4130 GTCATTCCCATTTAGGTCCGATTCGGATCCCTCCGTTGTTTCCTTTTCCT 4179

LserLAC005780_C 4148 GTCATTCCCATTTAGGTCCGATTCGGATCCCTCCGTTGTTTCCTTTTCCT 4197

LsalLAC008020_C 4148 GTCATTCCCATTTAGGTCCGATTCGGATCCCTCCGTTGTTTCCTTTTCCT 4197

LsalCGN5271_CDS 4148 GTCATTCCCATTTAGGTCCGATTCGGATCCCTCCGTTGTTTCCTTTTCCT 4197

LsatLACWENDEL_C 4165 TCCGCACCTTTTCCTCGAAATGAGAAAGAAGATGGTACACTTGAATTGTA 4214

LsatSalinas_CDS 4165 TCCGCACCTTTTCCTCGAAATGAGAAAGAAGATGGTACACTTGAATTGTA 4214

LserUS96UC23_CD 4165 TCCGCACCTTTTCCTCGAAATGAGAAAGAAGATGGTACACTTGAATTGTA 4214

LvirLAC006941_C 4180 TCCGCACCTTTTCCTCGAAATGAGAAAGAAGATGGTACACTTGAATTGTA 4229

LserLAC005780_C 4198 TCCGCACCTTTTCCTCGAAATGAGAAAGAAGATGGTACACTTGAATTGTA 4247

LsalLAC008020_C 4198 TCCGCACCTTTTCCTCGAAATGAGAAAGAAGATGGTACACTTGAATTGTA 4247

LsalCGN5271_CDS 4198 TCCGCACCTTTTCCTCGAAATGAGAAAGAAGATGGTACACTTGAATTGTA 4247

LsatLACWENDEL_C 4215 TTATTTAAGTGCTTATTGCTTGCCAAAAATCCTACTTCTACAATTGGTAG 4264

LsatSalinas_CDS 4215 TTATTTAAGTGCTTATTGCTTGCCAAAAATCCTACTTCTACAATTGGTAG 4264

LserUS96UC23_CD 4215 TTATTTAAGTGCTTATTGCTTGCCAAAAATCCTACTTCTACAATTGGTAG 4264

LvirLAC006941_C 4230 TTATTTAAGTGCTTATTGCTTGCCAAAAATCCTACTTCTACAATTGGTAG 4279

LserLAC005780_C 4248 TTATTTAAGTGCTTATTGCTTGCCAAAAATCCTACTTCTACAATTGGTAG 4297

LsalLAC008020_C 4248 TTATTTAAGTGCTTATTGCTTGCCAAAAATCCTACTTCTACAATTGGTAG 4297

LsalCGN5271_CDS 4248 TTATTTAAGTGCTTATTGCTTGCCAAAAATCCTACTTCTACAATTGGTAG 4297

LsatLACWENDEL_C 4265 GTCACCGGGTTATTCAAATAAGTCGTGTTTTCCGTGGTTTTCCCATGTTA 4314

LsatSalinas_CDS 4265 GTCACCGGGTTATTCAAATAAGTCGTGTTTTCCGTGGTTTTCCCATGTTA 4314

LserUS96UC23_CD 4265 GTCACCGGGTTATTCAAATAAGTCGTGTTTTCCGTGGTTTTCCCATGTTA 4314

LvirLAC006941_C 4280 GTCACCGGGTTATTCAAATAAGTCGTGTTTTCCGTGGTTTTCCCATGTTA 4329

LserLAC005780_C 4298 GTCACCGGGTTATTCAAATAAGTCGTGTTTTCCGTGGTTTTCCCATGTTA 4347

LsalLAC008020_C 4298 GTCACCGGGTTATTCAAATAAGTCGTGTTTTCCGTGGTTTTCCCATGTTA 4347

LsalCGN5271_CDS 4298 GTCACCGGGTTATTCAAATAAGTCGTGTTTTCCGTGGTTTTCCCATGTTA 4347

LsatLACWENDEL_C 4315 CAACTTCCGTACCAATTCGGTCGATCCGGAATGGATCGGTTAAACATTCC 4364

LsatSalinas_CDS 4315 CAACTTCCGTACCAATTCGGTCGATCCGGAATGGATCGGTTAAACATTCC 4364

LserUS96UC23_CD 4315 CAACTTCCGTACCAATTCGGTCGATCCGGAATGGATCGGTTAAACATTCC 4364

LvirLAC006941_C 4330 CAACTTCCGTACCAATTCGGTCGATCCGGAATGGATCGGTTAAACATTCC 4379

LserLAC005780_C 4348 CAACTTCCGTACCAATTCGGTCGATCCGGAATGGATCGGTTAAACATTCC 4397

LsalLAC008020_C 4348 CAACTTCCGTACCAATTCGGTCGATCCGGAATGGATCGGTTAAACATTCC 4397

LsalCGN5271_CDS 4348 CAACTTCCGTACCAATTCGGTCGATCCGGAATGGATCGGTTAAACATTCC 4397

LsatLACWENDEL_C 4365 ATTAGGGAGCCTGGTCTTGACTTTTCTGTGTGGTATTCATTCTCGTTCGG 4414

LsatSalinas_CDS 4365 ATTAGGGAGCCTGGTCTTGACTTTTCTGTGTGGTATTCATTCTCGTTCGG 4414

LserUS96UC23_CD 4365 ATTAGGGAGCCTGGTCTTGACTTTTCTGTGTGGTATTCATTCTCGTTCGG 4414

LvirLAC006941_C 4380 ATTAGGGAGCCTGGTCTTGACTTTTCTGTGTGGTATTCATTCTCGTTCGG 4429

LserLAC005780_C 4398 ATTAGGGAGCCTGGTCTTGACTTTTCTGTGTGGTATTCATTCTCGTTCGG 4447

LsalLAC008020_C 4398 ATTAGGGAGCCTGGTCTTGACTTTTCTGTGTGGTATTCATTCTCGTTCGG 4447

LsalCGN5271_CDS 4398 ATTAGGGAGCCTGGTCTTGACTTTTCTGTGTGGTATTCATTCTCGTTCGG 4447

LsatLACWENDEL_C 4415 CTCTTGGAATCACATCCAGCAGTGGTGGGAACAGCTCGCAAAATCCAACC 4464

LsatSalinas_CDS 4415 CTCTTGGAATCACATCCAGCAGTGGTGGGAACAGCTCGCAAAATCCAACC 4464

LserUS96UC23_CD 4415 CTCTTGGAATCACATCCAGCAGTGGTGGGAACAGCTCGCAAAATCCAACC 4464

LvirLAC006941_C 4430 CTCTTGGAATCACATCCAGCAGTGGTGGGAACAGCTCGCAAAATCCAACC 4479

LserLAC005780_C 4448 CTCTTGGAATCACATCCAGCAGTGGTGGGAACAGCTCGCAAAATCCAACC 4497

LsalLAC008020_C 4448 CTCTTGGAATCACATCCAGCAGTGGTGGGAACAGCTCGCAAAATCCAACC 4497

LsalCGN5271_CDS 4448 CTCTTGGAATCACATCCAGCAGTGGTGGGAACAGCTCGCAAAATCCAACC 4497

LsatLACWENDEL_C 4465 ACTTCACCTACTTCATTGCCCCCAACCCTTTCTCGTACCTCTATTGAAAC 4514

LsatSalinas_CDS 4465 ACTTCACCTACTTCATTGCCCCCAACCCTTTCTCGTACCTCTATTGAAAC 4514

LserUS96UC23_CD 4465 ACTTCACCTACTTCATTGCCCCCAACCCTTTCTCGTACCTCTATTGAAAC 4514

LvirLAC006941_C 4480 ACTTCACCTACTTCATTGCCCCCAACCCTTTCTCGTACCTCTATTGAAAC 4529

LserLAC005780_C 4498 ACTTCACCTACTTCATTGCCCCCAACCCTTTCTCGTACCTCTATTGAAAC 4547

LsalLAC008020_C 4498 ACTTCACCTACTTCATTGCCCCCAACCCTTTCTCGTACCTCTATTGAAAC 4547

LsalCGN5271_CDS 4498 ACTTCACCTACTTCATTGCCCCCAACCCTTTCTCGTACCTCTATTGAAAC 4547

LsatLACWENDEL_C 4515 AGAATGGTTTCATGTTCTTTCATCGATTGGTTATTCCTTTCCGTTCGTAT 4564

LsatSalinas_CDS 4515 AGAATGGTTTCATGTTCTTTCATCGATTGGTTATTCCTTTCCGTTCGTAT 4564

LserUS96UC23_CD 4515 AGAATGGTTTCATGTTCTTTCATCGATTGGTTATTCCTTTCCGTTCGTAT 4564

LvirLAC006941_C 4530 AGAATGGTTTCATGTTCTTTCATCGATTGGTTATTCCTTTCCGTTCGTAT 4579

LserLAC005780_C 4548 AGAATGGTTTCATGTTCTTTCATCGATTGGTTATTCCTTTCCGTTCGTAT 4597

LsalLAC008020_C 4548 AGAATGGTTTCATGTTCTTTCATCGATTGGTTATTCCTTTCCGTTCGTAT 4597

LsalCGN5271_CDS 4548 AGAATGGTTTCATGTTCTTTCATCGATTGGTTATTCCTTTCCGTTCGTAT 4597

LsatLACWENDEL_C 4565 CTCTTTCTCCAATTTCGGTCTCGATTAGTTCACAAGATTGAATGTCCCTT 4614

LsatSalinas_CDS 4565 CTCTTTCTCCAATTTCGGTCTCGATTAGTTCACAAGATTGAATGTCCCTT 4614

LserUS96UC23_CD 4565 CTCTTTCTCCAATTTCGGTCTCGATTAGTTCACAAGATTGAATGTCCCTT 4614

LvirLAC006941_C 4580 CTCTTTCTCCAATTTCGGTCTCGATTAGTTCACAAGATTGAATGTCCCTT 4629

LserLAC005780_C 4598 CTCTTTCTCCAATTTCGGTCTCGATTAGTTCACAAGATTGAATGTCCCTT 4647

LsalLAC008020_C 4598 CTCTTTCTCCAATTTCGGTCTCGATTAGTTCACAAGATTGAATGTCCCTT 4647

LsalCGN5271_CDS 4598 CTCTTTCTCCAATTTCGGTCTCGATTAGTTCACAAGATTGAATGTCCCTT 4647

LsatLACWENDEL_C 4615 GCATTATTACAACCTTCTTTTTTGATGTCAAAGACCAGAAGCTACGCGAA 4664

LsatSalinas_CDS 4615 GCATTATTACAACCTTCTTTTTTGATGTCAAAGACCAGAAGCTACGCGAA 4664

LserUS96UC23_CD 4615 GCATTATTACAACCTTCTTTTTTGATGTCAAAGACCAGAAGCTACGCGAA 4664

LvirLAC006941_C 4630 GCATTATTACAACCTTCTTTTTTGATGTCAAAGACCAGAAGCTACGCGAA 4679

LserLAC005780_C 4648 GCATTATTACAACCTTCTTTTTTGATGTCAAAGACCAGAAGCTACGCGAA 4697

LsalLAC008020_C 4648 GCATTATTACAACCTTCTTTTTTGATGTCAAAGACCAGAAGCTACGCGAA 4697

LsalCGN5271_CDS 4648 GCATTATTACAACCTTCTTTTTTGATGTCAAAGACCAGAAGCTACGCGAA 4697

LsatLACWENDEL_C 4665 AATTTTCATTGGATCTCGGTTGTTCTTAACAGCGATGGCTATTCATTTAA 4714

LsatSalinas_CDS 4665 AATTTTCATTGGATCTCGGTTGTTCTTAACAGCGATGGCTATTCATTTAA 4714

LserUS96UC23_CD 4665 AATTTTCATTGGATCTCGGTTGTTCTTAACAGCGATGGCTATTCATTTAA 4714

LvirLAC006941_C 4680 AATTTTCATTGGATCTCGGTTGTTCTTAACAGCGATGGCTATTCATTTAA 4729

LserLAC005780_C 4698 AATTTTCATTGGATCTCGGTTGTTCTTAACAGCGATGGCTATTCATTTAA 4747

LsalLAC008020_C 4698 AATTTTCATTGGATCTCGGTTGTTCTTAACAGCGATGGCTATTCATTTAA 4747

LsalCGN5271_CDS 4698 AATTTTCATTGGATCTCGGTTGTTCTTAACAGCGATGGCTATTCATTTAA 4747

LsatLACWENDEL_C 4715 GTCTTCGGGTAGCACCACTAGACCTTCAACAAGGTGGAAATTCTCGTATT 4764

LsatSalinas_CDS 4715 GTCTTCGGGTAGCACCACTAGACCTTCAACAAGGTGGAAATTCTCGTATT 4764

LserUS96UC23_CD 4715 GTCTTCGGGTAGCACCACTAGACCTTCAACAAGGTGGAAATTCTCGTATT 4764

LvirLAC006941_C 4730 GTCTTCGGGTAGCACCACTAGACCTTCAACAAGGTGGAAATTCTCGTATT 4779

LserLAC005780_C 4748 GTCTTCGGGTAGCACCACTAGACCTTCAACAAGGTGGAAATTCTCGTATT 4797

LsalLAC008020_C 4748 GTCTTCGGGTAGCACCACTAGACCTTCAACAAGGTGGAAATTCTCGTATT 4797

LsalCGN5271_CDS 4748 GTCTTCGGGTAGCACCACTAGACCTTCAACAAGGTGGAAATTCTCGTATT 4797

LsatLACWENDEL_C 4765 CCTTATGTACACGTTCCTGCGGCTCGGATGAGTATTCTTGTTTATATCGC 4814

LsatSalinas_CDS 4765 CCTTATGTACACGTTCCTGCGGCTCGGATGAGTATTCTTGTTTATATCGC 4814

LserUS96UC23_CD 4765 CCTTATGTACACGTTCCTGCGGCTCGGATGAGTATTCTTGTTTATATCGC 4814

LvirLAC006941_C 4780 CCTTATGTACACGTTCCTGCGGCTCGGATGAGTATTCTTGTTTATATCGC 4829

LserLAC005780_C 4798 CCTTATGTACACGTTCCTGCGGCTCGGATGAGTATTCTTGTTTATATCGC 4847

LsalLAC008020_C 4798 CCTTATGTACACGTTCCTGCGGCTCGGATGAGTATTCTTGTTTATATCGC 4847

LsalCGN5271_CDS 4798 CCTTATGTACACGTTCCTGCGGCTCGGATGAGTATTCTTGTTTATATCGC 4847

LsatLACWENDEL_C 4815 TACGGCTATAAACACGTTCTTGTTCCTATTAACAAAACATCCCCTCTTTC 4864

LsatSalinas_CDS 4815 TACGGCTATAAACACGTTCTTGTTCCTATTAACAAAACATCCCCTCTTTC 4864

LserUS96UC23_CD 4815 TACGGCTATAAACACGTTCTTGTTCCTATTAACAAAACATCCCCTCTTTC 4864

LvirLAC006941_C 4830 TACGGCTATAAACACGTTCTTGTTCCTATTAACAAAACATCCCCTCTTTC 4879

LserLAC005780_C 4848 TACGGCTATAAACACGTTCTTGTTCCTATTAACAAAACATCCCCTCTTTC 4897

LsalLAC008020_C 4848 TACGGCTATAAACACGTTCTTGTTCCTATTAACAAAACATCCCCTCTTTC 4897

LsalCGN5271_CDS 4848 TACGGCTATAAACACGTTCTTGTTCCTATTAACAAAACATCCCCTCTTTC 4897

LsatLACWENDEL_C 4865 TTCGCTCTTTCGGAACCGGTACAGAAATGGGTGCTTTTTCTACGTTGTTT 4914

LsatSalinas_CDS 4865 TTCGCTCTTTCGGAACCGGTACAGAAATGGGTGCTTTTTCTACGTTGTTT 4914

LserUS96UC23_CD 4865 TTCGCTCTTTCGGAACCGGTACAGAAATGGGTGCTTTTTCTACGTTGTTT 4914

LvirLAC006941_C 4880 TTCGCTCTTTCGGAACCGGTACAGAAATGGGTGCTTTTTCTACGTTGTTT 4929

LserLAC005780_C 4898 TTCGCTCTTTCGGAACCGGTACAGAAATGGGTGCTTTTTCTACGTTGTTT 4947

LsalLAC008020_C 4898 TTCGCTCTTTCGGAACCGGTACAGAAATGGGTGCTTTTTCTACGTTGTTT 4947

LsalCGN5271_CDS 4898 TTCGCTCTTTCGGAACCGGTACAGAAATGGGTGCTTTTTCTACGTTGTTT 4947

LsatLACWENDEL_C 4915 ACCTTAGTTACTGGGGGGTTTCGGGGAAGACCTATGTGGGGCACCTTTTG 4964

LsatSalinas_CDS 4915 ACCTTAGTTACTGGGGGGTTTCGGGGAAGACCTATGTGGGGCACCTTTTG 4964

LserUS96UC23_CD 4915 ACCTTAGTTACTGGGGGGTTTCGGGGAAGACCTATGTGGGGCACCTTTTG 4964

LvirLAC006941_C 4930 ACCTTAGTTACTGGGGGGTTTCGGGGAAGACCTATGTGGGGCACCTTTTG 4979

LserLAC005780_C 4948 ACCTTAGTTACTGGGGGGTTTCGGGGAAGACCTATGTGGGGCACCTTTTG 4997

LsalLAC008020_C 4948 ACCTTAGTTACTGGGGGGTTTCGGGGAAGACCTATGTGGGGCACCTTTTG 4997

LsalCGN5271_CDS 4948 ACCTTAGTTACTGGGGGGTTTCGGGGAAGACCTATGTGGGGCACCTTTTG 4997

LsatLACWENDEL_C 4965 GGTGTGGGATGCTCGTTTAACCTCTGTATTAATCTCGTTCCTTATTTACA 5014

LsatSalinas_CDS 4965 GGTGTGGGATGCTCGTTTAACCTCTGTATTAATCTCGTTCCTTATTTACA 5014

LserUS96UC23_CD 4965 GGTGTGGGATGCTCGTTTAACCTCTGTATTAATCTCGTTCCTTATTTACA 5014

LvirLAC006941_C 4980 GGTGTGGGATGCTCGTTTAACCTCTGTATTAATCTCGTTCCTTATTTACA 5029

LserLAC005780_C 4998 GGTGTGGGATGCTCGTTTAACCTCTGTATTAATCTCGTTCCTTATTTACA 5047

LsalLAC008020_C 4998 GGTGTGGGATGCTCGTTTAACCTCTGTATTAATCTCGTTCCTTATTTACA 5047

LsalCGN5271_CDS 4998 GGTGTGGGATGCTCGTTTAACCTCTGTATTAATCTCGTTCCTTATTTACA 5047

LsatLACWENDEL_C 5015 TGGGTGCACTGCGTTTTCAAAAGCTTCCTATCGAACCGGCTCCTATTTCA 5064

LsatSalinas_CDS 5015 TGGGTGCACTGCGTTTTCAAAAGCTTCCTATCGAACCGGCTCCTATTTCA 5064

LserUS96UC23_CD 5015 TGGGTGCACTGCGTTTTCAAAAGCTTCCTATCGAACCGGCTCCTATTTCA 5064

LvirLAC006941_C 5030 TGGGTGCACTGCGTTTTCAAAAGCTTCCTATCGAACCGGCTCCTATTTCA 5079

LserLAC005780_C 5048 TGGGTGCACTGCGTTTTCAAAAGCTTCCTATCGAACCGGCTCCTATTTCA 5097

LsalLAC008020_C 5048 TGGGTGCACTGCGTTTTCAAAAGCTTCCTATCGAACCGGCTCCTATTTCA 5097

LsalCGN5271_CDS 5048 TGGGTGCACTGCGTTTTCAAAAGCTTCCTATCGAACCGGCTCCTATTTCA 5097

LsatLACWENDEL_C 5065 ATCCGTGCTGGACCGATCGATATACCAATAATCAAGTCTTCAGTCAACTG 5114

LsatSalinas_CDS 5065 ATCCGTGCTGGACCGATCGATATACCAATAATCAAGTCTTCAGTCAACTG 5114

LserUS96UC23_CD 5065 ATCCGTGCTGGACCGATCGATATACCAATAATCAAGTCTTCAGTCAACTG 5114

LvirLAC006941_C 5080 ATCCGTGCTGGACCGATCGATATACCAATAATCAAGTCTTCAGTCAACTG 5129

LserLAC005780_C 5098 ATCCGTGCTGGACCGATCGATATACCAATAATCAAGTCTTCAGTCAACTG 5147

LsalLAC008020_C 5098 ATCCGTGCTGGACCGATCGATATACCAATAATCAAGTCTTCAGTCAACTG 5147

LsalCGN5271_CDS 5098 ATCCGTGCTGGACCGATCGATATACCAATAATCAAGTCTTCAGTCAACTG 5147

LsatLACWENDEL_C 5115 GTGGAATACATCGCATCAACCTGGGAGCATTAGCCGATATGGTACATCAA 5164

LsatSalinas_CDS 5115 GTGGAATACATCGCATCAACCTGGGAGCATTAGCCGATATGGTACATCAA 5164

LserUS96UC23_CD 5115 GTGGAATACATCGCATCAACCTGGGAGCATTAGCCGATATGGTACATCAA 5164

LvirLAC006941_C 5130 GTGGAATACATCGCATCAACCTGGGAGCATTAGCCGATATGGTACATCAA 5179

LserLAC005780_C 5148 GTGGAATACATCGCATCAACCTGGGAGCATTAGCCGATATGGTACATCAA 5197

LsalLAC008020_C 5148 GTGGAATACATCGCATCAACCTGGGAGCATTAGCCGATATGGTACATCAA 5197

LsalCGN5271_CDS 5148 GTGGAATACATCGCATCAACCTGGGAGCATTAGCCGATATGGTACATCAA 5197

LsatLACWENDEL_C 5165 TACATGTTCCTATGCCCATTCCAATCTTGTCTAACTTTGCTAACTCCCCC 5214

LsatSalinas_CDS 5165 TACATGTTCCTATGCCCATTCCAATCTTGTCTAACTTTGCTAACTCCCCC 5214

LserUS96UC23_CD 5165 TACATGTTCCTATGCCCATTCCAATCTTGTCTAACTTTGCTAACTCCCCC 5214

LvirLAC006941_C 5180 TACATGTTCCTATGCCCATTCCAATCTTGTCTAACTTTGCTAACTCCCCC 5229

LserLAC005780_C 5198 TACATGTTCCTATGCCCATTCCAATCTTGTCTAACTTTGCTAACTCCCCC 5247

LsalLAC008020_C 5198 TACATGTTCCTATGCCCATTCCAATCTTGTCTAACTTTGCTAACTCCCCC 5247

LsalCGN5271_CDS 5198 TACATGTTCCTATGCCCATTCCAATCTTGTCTAACTTTGCTAACTCCCCC 5247

LsatLACWENDEL_C 5215 TTCTCAACCCGTATCTTGTTCGTTCTGGAAACACGTCTTCTTATTCCATC 5264

LsatSalinas_CDS 5215 TTCTCAACCCGTATCTTGTTCGTTCTGGAAACACGTCTTCTTATTCCATC 5264

LserUS96UC23_CD 5215 TTCTCAACCCGTATCTTGTTCGTTCTGGAAACACGTCTTCTTATTCCATC 5264

LvirLAC006941_C 5230 TTCTCAACCCGTATCTTGTTCGTTCTGGAAACACGTCTTCTTATTCCATC 5279

LserLAC005780_C 5248 TTCTCAACCCGTATCTTGTTCGTTCTGGAAACACGTCTTCTTATTCCATC 5297

LsalLAC008020_C 5248 TTCTCAACCCGTATCTTGTTCGTTCTGGAAACACGTCTTCTTATTCCATC 5297

LsalCGN5271_CDS 5248 TTCTCAACCCGTATCTTGTTCGTTCTGGAAACACGTCTTCTTATTCCATC 5297

LsatLACWENDEL_C 5265 TTTTCTCGAATCTCCTTTAACGGAAGAAATAGAAGCTCAAGAAGGAATAC 5314

LsatSalinas_CDS 5265 TTTTCTCGAATCTCCTTTAACGGAAGAAATAGAAGCTCAAGAAGGAATAC 5314

LserUS96UC23_CD 5265 TTTTCTCGAATCTCCTTTAACGGAAGAAATAGAAGCTCAAGAAGGAATAC 5314

LvirLAC006941_C 5280 TTTTCTCGAATCTCCTTTAACGGAAGAAATAGAAGCTCAAGAAGGAATAC 5329

LserLAC005780_C 5298 TTTTCTCGAATCTCCTTTAACGGAAGAAATAGAAGCTCAAGAAGGAATAC 5347

LsalLAC008020_C 5298 TTTTCTCGAATCTCCTTTAACGGAAGAAATAGAAGCTCAAGAAGGAATAC 5347

LsalCGN5271_CDS 5298 TTTTCTCGAATCTCCTTTAACGGAAGAAATAGAAGCTCAAGAAGGAATAC 5347

LsatLACWENDEL_C 5315 CAAAACCTAGTTCACTCGCTGAGTCTCTTTGCATCCATGGCTGAATGGTC 5364

LsatSalinas_CDS 5315 CAAAACCTAGTTCACTCGCTGAGTCTCTTTGCATCCATGGCTGAATGGTC 5364

LserUS96UC23_CD 5315 CAAAACCTAGTTCACTCGCTGAGTCTCTTTGCATCCATGGCTGAATGGTC 5364

LvirLAC006941_C 5330 CAAAACCTAGTTCACTCGCTGAGTCTCTTTGCATCCATGGCTGAATGGTC 5379

LserLAC005780_C 5348 CAAAACCTAGTTCACTCGCTGAGTCTCTTTGCATCCATGGCTGAATGGTC 5397

LsalLAC008020_C 5348 CAAAACCTAGTTCACTCGCTGAGTCTCTTTGCATCCATGGCTGAATGGTC 5397

LsalCGN5271_CDS 5348 CAAAACCTAGTTCACTCGCTGAGTCTCTTTGCATCCATGGCTGAATGGTC 5397

LsatLACWENDEL_C 5365 CAACTACAAAACTTTTTCTTTTTCATTACTTCCATGGTCGTGCCTCGTGG 5414

LsatSalinas_CDS 5365 CAACTACAAAACTTTTTCTTTTTCATTACTTCCATGGTCGTGCCTCGTGG 5414

LserUS96UC23_CD 5365 CAACTACAAAACTTTTTCTTTTTCATTACTTCCATGGTCGTGCCTCGTGG 5414

LvirLAC006941_C 5380 CAACTACAAAACTTTTTCTTTTTCATTACTTCCATGGTCGTGCCTCGTGG 5429

LserLAC005780_C 5398 CAACTACAAAACTTTTTCTTTTTCATTACTTCCATGGTCGTGCCTCGTGG 5447

LsalLAC008020_C 5398 CAACTACAAAACTTTTTCTTTTTCATTACTTCCATGGTCGTGCCTCGTGG 5447

LsalCGN5271_CDS 5398 CAACTACAAAACTTTTTCTTTTTCATTACTTCCATGGTCGTGCCTCGTGG 5447

LsatLACWENDEL_C 5415 CACGGCAGCACCCGTACTATTGAAATGGTTCGTCAGTAGAGATGTTCCCA 5464

LsatSalinas_CDS 5415 CACGGCAGCACCCGTACTATTGAAATGGTTCGTCAGTAGAGATGTTCCCA 5464

LserUS96UC23_CD 5415 CACGGCAGCACCCGTACTATTGAAATGGTTCGTCAGTAGAGATGTTCCCA 5464

LvirLAC006941_C 5430 CACGGCAGCACCCGTACTATTGAAATGGTTCGTCAGTAGAGATGTTCCCA 5479

LserLAC005780_C 5448 CACGGCAGCACCCGTACTATTGAAATGGTTCGTCAGTAGAGATGTTCCCA 5497

LsalLAC008020_C 5448 CACGGCAGCACCCGTACTATTGAAATGGTTCGTCAGTAGAGATGTTCCCA 5497

LsalCGN5271_CDS 5448 CACGGCAGCACCCGTACTATTGAAATGGTTCGTCAGTAGAGATGTTCCCA 5497

LsatLACWENDEL_C 5465 CAGGTGCCCCTTCTTCTAATGGTACTATAATTCCTATTCCTATCCCTTCA 5514

LsatSalinas_CDS 5465 CAGGTGCCCCTTCTTCTAATGGTACTATAATTCCTATTCCTATCCCTTCA 5514

LserUS96UC23_CD 5465 CAGGTGCCCCTTCTTCTAATGGTACTATAATTCCTATTCCTATCCCTTCA 5514

LvirLAC006941_C 5480 CAGGTGCCCCTTCTTCTAATGGTACTATAATTCCTATTCCTATCCCTTCA 5529

LserLAC005780_C 5498 CAGGTGCCCCTTCTTCTAATGGTACTATAATTCCTATTCCTATCCCTTCA 5547

LsalLAC008020_C 5498 CAGGTGCCCCTTCTTCTAATGGTACTATAATTCCTATTCCTATCCCTTCA 5547

LsalCGN5271_CDS 5498 CAGGTGCCCCTTCTTCTAATGGTACTATAATTCCTATTCCTATCCCTTCA 5547

LsatLACWENDEL_C 5515 TTCCCTCTTTTGGTCTATCTACATTCCAGGAAATTCATACGCTCCACGGA 5564

LsatSalinas_CDS 5515 TTCCCTCTTTTGGTCTATCTACATTCCAGGAAATTCATACGCTCCACGGA 5564

LserUS96UC23_CD 5515 TTCCCTCTTTTGGTCTATCTACATTCCAGGAAATTCATACGCTCCACGGA 5564

LvirLAC006941_C 5530 TTCCCTCTTTTGGTCTATCTACATTCCAGGAAATTCATACGCTCCACGGA 5579

LserLAC005780_C 5548 TTCCCTCTTTTGGTCTATCTACATTCCAGGAAATTCATACGCTCCACGGA 5597

LsalLAC008020_C 5548 TTCCCTCTTTTGGTCTATCTACATTCCAGGAAATTCATACGCTCCACGGA 5597

LsalCGN5271_CDS 5548 TTCCCTCTTTTGGTCTATCTACATTCCAGGAAATTCATACGCTCCACGGA 5597

LsatLACWENDEL_C 5565 CGGAGCAAAAAGCGGAGTCTTGGTCAGAGCAAGCCGACCTATTCTATTAC 5614

LsatSalinas_CDS 5565 CGGAGCAAAAAGCGGAGTCTTGGTCAGAGCAAGCCGACCTATTCTATTAC 5614

LserUS96UC23_CD 5565 CGGAGCAAAAAGCGGAGTCTTGGTCAGAGCAAGCCGACCTATTCTATTAC 5614

LvirLAC006941_C 5580 CGGAGCAAAAAGCGGAGTCTTGGTCAGAGCAAGCCGACCTATTCTATTAC 5629

LserLAC005780_C 5598 CGGAGCAAAAAGCGGAGTCTTGGTCAGAGCAAGCCGACCTATTCTATTAC 5647

LsalLAC008020_C 5598 CGGAGCAAAAAGCGGAGTCTTGGTCAGAGCAAGCCGACCTATTCTATTAC 5647

LsalCGN5271_CDS 5598 CGGAGCAAAAAGCGGAGTCTTGGTCAGAGCAAGCCGACCTATTCTATTAC 5647

LsatLACWENDEL_C 5615 CAGACATAATTGGGGGAAGCTCATCCGAAACTAGAGCTAGAAAGGCCTTA 5664

LsatSalinas_CDS 5615 CAGACATAATTGGGGGAAGCTCATCCGAAACTAGAGCTAGAAAGGCCTTA 5664

LserUS96UC23_CD 5615 CAGACATAATTGGGGGAAGCTCATCCGAAACTAGAGCTAGAAAGGCCTTA 5664

LvirLAC006941_C 5630 CAGACATAATTGGGGGAAGCTCATCCGAAACTAGAGCTAGAAAGGCCTTA 5679

LserLAC005780_C 5648 CAGACATAATTGGGGGAAGCTCATCCGAAACTAGAGCTAGAAAGGCCTTA 5697

LsalLAC008020_C 5648 CAGACATAATTGGGGGAAGCTCATCCGAAACTAGAGCTAGAAAGGCCTTA 5697

LsalCGN5271_CDS 5648 CAGACATAATTGGGGGAAGCTCATCCGAAACTAGAGCTAGAAAGGCCTTA 5697

LsatLACWENDEL_C 5665 TTTCGTTTCGTTCCTGTTCTTCATTTCCTTCTTATCGAAAAAAAGGGGGA 5714

LsatSalinas_CDS 5665 TTTCGTTTCGTTCCTGTTCTTCATTTCCTTCTTATCGAAAAAAAGGGGGA 5714

LserUS96UC23_CD 5665 TTTCGTTTCGTTCCTGTTCTTCATTTCCTTCTTATCGAAAAAAAGGGGGA 5714

LvirLAC006941_C 5680 TTTCGTTTCGTTCCTGTTCTTCATTTCCTTCTTATCGAAAAAAAGGGGGA 5729

LserLAC005780_C 5698 TTTCGTTTCGTTCCTGTTCTTCATTTCCTTCTTATCGAAAAAAAGGGGGA 5747

LsalLAC008020_C 5698 TTTCGTTTCGTTCCTGTTCTTCATTTCCTTCTTATCGAAAAAAAGGGGGA 5747

LsalCGN5271_CDS 5698 TTTCGTTTCGTTCCTGTTCTTCATTTCCTTCTTATCGAAAAAAAGGGGGA 5747

LsatLACWENDEL_C 5715 TTTCTCATATTTAGAATCTTTCTGCGGTGTGCTCCGTTTACTATTCTTTC 5764

LsatSalinas_CDS 5715 TTTCTCATATTTAGAATCTTTCTGCGGTGTGCTCCGTTTACTATTCTTTC 5764

LserUS96UC23_CD 5715 TTTCTCATATTTAGAATCTTTCTGCGGTGTGCTCCGTTTACTATTCTTTC 5764

LvirLAC006941_C 5730 TTTCTCATATTTAGAATCTTTCTGCGGTGTGCTCCGTTTACTATTCTTTC 5779

LserLAC005780_C 5748 TTTCTCATATTTAGAATCTTTCTGCGGTGTGCTCCGTTTACTATTCTTTC 5797

LsalLAC008020_C 5748 TTTCTCATATTTAGAATCTTTCTGCGGTGTGCTCCGTTTACTATTCTTTC 5797

LsalCGN5271_CDS 5748 TTTCTCATATTTAGAATCTTTCTGCGGTGTGCTCCGTTTACTATTCTTTC 5797

LsatLACWENDEL_C 5765 GTACTTTCTTCTCTTTACCACGCGATAGGTCAGCGAAGCGTGAGCGGGCG 5814

LsatSalinas_CDS 5765 GTACTTTCTTCTCTTTACCACGCGATAGGTCAGCGAAGCGTGAGCGGGCG 5814

LserUS96UC23_CD 5765 GTACTTTCTTCTCTTTACCACGCGATAGGTCAGCGAAGCGTGAGCGGGCG 5814

LvirLAC006941_C 5780 GTACTTTCTTCTCTTTACCACGCGATAGGTCAGCGAAGCGTGAGCGGGCG 5829

LserLAC005780_C 5798 GTACTTTCTTCTCTTTACCACGCGATAGGTCAGCGAAGCGTGAGCGGGCG 5847

LsalLAC008020_C 5798 GTACTTTCTTCTCTTTACCACGCGATAGGTCAGCGAAGCGTGAGCGGGCG 5847

LsalCGN5271_CDS 5798 GTACTTTCTTCTCTTTACCACGCGATAGGTCAGCGAAGCGTGAGCGGGCG 5847

LsatLACWENDEL_C 5815 CGGAGAAGGAAAGGCCAAAGACTTCAGCCTAACGGGAATGAGCAAGGACG 5864

LsatSalinas_CDS 5815 CGGAGAAGGAAAGGCCAAAGACTTCAGCCTAACGGGAATGAGCAAGGACG 5864

LserUS96UC23_CD 5815 CGGAGAAGGAAAGGCCAAAGACTTCAGCCTAACGGGAATGAGCAAGGACG 5864

LvirLAC006941_C 5830 CGGAGAAGGAAAGGCCAAAGACTTCAGCCTAACGGGAATGAGCAAGGACG 5879

LserLAC005780_C 5848 CGGAGAAGGAAAGGCCAAAGACTTCAGCCTAACGGGAATGAGCAAGGACG 5897

LsalLAC008020_C 5848 CGGAGAAGGAAAGGCCAAAGACTTCAGCCTAACGGGAATGAGCAAGGACG 5897

LsalCGN5271_CDS 5848 CGGAGAAGGAAAGGCCAAAGACTTCAGCCTAACGGGAATGAGCAAGGACG 5897

LsatLACWENDEL_C 5865 AAATGACAAGATGAGGCGCCCTGGGCACCCCCATTTAGAAAGAAGGGCCG 5914

LsatSalinas_CDS 5865 AAATGACAAGATGAGGCGCCCTGGGCACCCCCATTTAGAAAGAAGGGCCG 5914

LserUS96UC23_CD 5865 AAATGACAAGATGAGGCGCCCTGGGCACCCCCATTTAGAAAGAAGGGCCG 5914

LvirLAC006941_C 5880 AAATGACAAGATGAGGCGCCCTGGGCACCCCCATTTAGAAAGAAGGGCCG 5929

LserLAC005780_C 5898 AAATGACAAGATGAGGCGCCCTGGGCACCCCCATTTAGAAAGAAGGGCCG 5947

LsalLAC008020_C 5898 AAATGACAAGATGAGGCGCCCTGGGCACCCCCATTTAGAAAGAAGGGCCG 5947

LsalCGN5271_CDS 5898 AAATGACAAGATGAGGCGCCCTGGGCACCCCCATTTAGAAAGAAGGGCCG 5947

LsatLACWENDEL_C 5915 AAGTTTTTGGGCCTGTAGCTTTACCCGTCCCCCCTGAGTCGGGCGGTGCT 5964

LsatSalinas_CDS 5915 AAGTTTTTGGGCCTGTAGCTTTACCCGTCCCCCCTGAGTCGGGCGGTGCT 5964

LserUS96UC23_CD 5915 AAGTTTTTGGGCCTGTAGCTTTACCCGTCCCCCCTGAGTCGGGCGGTGCT 5964

LvirLAC006941_C 5930 AAGTTTTTGGGCCTGTAGCTTTACCCGTCCCCCCTGAGTCGGGCGGTGCT 5979

LserLAC005780_C 5948 AAGTTTTTGGGCCTGTAGCTTTACCCGTCCCCCCTGAGTCGGGCGGTGCT 5997

LsalLAC008020_C 5948 AAGTTTTTGGGCCTGTAGCTTTACCCGTCCCCCCTGAGTCGGGCGGTGCT 5997

LsalCGN5271_CDS 5948 AAGTTTTTGGGCCTGTAGCTTTACCCGTCCCCCCTGAGTCGGGCGGTGCT 5997

LsatLACWENDEL_C 5965 TGTGTGGGGGGTGCGCCACCAGAAATCGGGCTTGAAGCTCTCACCTTACC 6014

LsatSalinas_CDS 5965 TGTGTGGGGGGTGCGCCACCAGAAATCGGGCTTGAAGCTCTCACCTTACC 6014

LserUS96UC23_CD 5965 TGTGTGGGGGGTGCGCCACCAGAAATCGGGCTTGAAGCTCTCACCTTACC 6014

LvirLAC006941_C 5980 TGTGTGGGGGGTGCGCCACCAGAAATCGGGCTTGAAGCTCTCACCTTACC 6029

LserLAC005780_C 5998 TGTGTGGGGGGTGCGCCACCAGAAATCGGGCTTGAAGCTCTCACCTTACC 6047

LsalLAC008020_C 5998 TGTGTGGGGGGTGCGCCACCAGAAATCGGGCTTGAAGCTCTCACCTTACC 6047

LsalCGN5271_CDS 5998 TGTGTGGGGGGTGCGCCACCAGAAATCGGGCTTGAAGCTCTCACCTTACC 6047

LsatLACWENDEL_C 6015 AACGAGCCGACAGCTGATGGCTGTTGGTCACGACTACTACAAAAAAGCCC 6064

LsatSalinas_CDS 6015 AACGAGCCGACAGCTGATGGCTGTTGGTCACGACTACTACAAAAAAGCCC 6064

LserUS96UC23_CD 6015 AACGAGCCGACAGCTGATGGCTGTTGGTCACGACTACTACAAAAAAGCCC 6064

LvirLAC006941_C 6030 AACGAGCCGACAGCTGATGGCTGTTGGTCACGACTACTACAAAAAAGCCC 6079

LserLAC005780_C 6048 AACGAGCCGACAGCTGATGGCTGTTGGTCACGACTACTACAAAAAAGCCC 6097

LsalLAC008020_C 6048 AACGAGCCGACAGCTGATGGCTGTTGGTCACGACTACTACAAAAAAGCCC 6097

LsalCGN5271_CDS 6048 AACGAGCCGACAGCTGATGGCTGTTGGTCACGACTACTACAAAAAAGCCC 6097

LsatLACWENDEL_C 6065 CAATGAAGATGAATATTTCACATGGAGGAGTGTGCATCTTTATGTTGGGT 6114

LsatSalinas_CDS 6065 CAATGAAGATGAATATTTCACATGGAGGAGTGTGCATCTTTATGTTGGGT 6114

LserUS96UC23_CD 6065 CAATGAAGATGAATATTTCACATGGAGGAGTGTGCATCTTTATGTTGGGT 6114

LvirLAC006941_C 6080 CAATGAAGATGAATATTTCACATGGAGGAGTGTGCATCTTTATGTTGGGT 6129

LserLAC005780_C 6098 CAATGAAGATGAATATTTCACATGGAGGAGTGTGCATCTTTATGTTGGGT 6147

LsalLAC008020_C 6098 CAATGAAGATGAATATTTCACATGGAGGAGTGTGCATCTTTATGTTGGGT 6147

LsalCGN5271_CDS 6098 CAATGAAGATGAATATTTCACATGGAGGAGTGTGCATCTTTATGTTGGGT 6147

LsatLACWENDEL_C 6115 GTTCTTCTGTCTAACACAAAGAAGATACAGTTCACTCAACGATTGCCTTT 6164

LsatSalinas_CDS 6115 GTTCTTCTGTCTAACACAAAGAAGATACAGTTCACTCAACGATTGCCTTT 6164

LserUS96UC23_CD 6115 GTTCTTCTGTCTAACACAAAGAAGATACAGTTCACTCAACGATTGCCTTT 6164

LvirLAC006941_C 6130 GTTCTTCTGTCTAACACAAAGAAGATACAGTTCACTCAACGATTGCCTTT 6179

LserLAC005780_C 6148 GTTCTTCTGTCTAACACAAAGAAGATACAGTTCACTCAACGATTGCCTTT 6197

LsalLAC008020_C 6148 GTTCTTCTGTCTAACACAAAGAAGATACAGTTCACTCAACGATTGCCTTT 6197

LsalCGN5271_CDS 6148 GTTCTTCTGTCTAACACAAAGAAGATACAGTTCACTCAACGATTGCCTTT 6197

LsatLACWENDEL_C 6165 GGGTTCCGAACTCCATATGGGGAAGGAGCGTTGTTGTTTGCAAGGTCTCG 6214

LsatSalinas_CDS 6165 GGGTTCCGAACTCCATATGGGGAAGGAGCGTTGTTGTTTGCAAGGTCTCG 6214

LserUS96UC23_CD 6165 GGGTTCCGAACTCCATATGGGGAAGGAGCGTTGTTGTTTGCAAGGTCTCG 6214

LvirLAC006941_C 6180 GGGTTCCGAACTCCATATGGGGAAGGAGCGTTGTTGTTTGCAAGGTCTCG 6229

LserLAC005780_C 6198 GGGTTCCGAACTCCATATGGGGAAGGAGCGTTGTTGTTTGCAAGGTCTCG 6247

LsalLAC008020_C 6198 GGGTTCCGAACTCCATATGGGGAAGGAGCGTTGTTGTTTGCAAGGTCTCG 6247

LsalCGN5271_CDS 6198 GGGTTCCGAACTCCATATGGGGAAGGAGCGTTGTTGTTTGCAAGGTCTCG 6247

LsatLACWENDEL_C 6215 ATTATTTACATGGACCCACTTCTCATTCAATTTGTGGGAATTTTCTGATC 6264

LsatSalinas_CDS 6215 ATTATTTACATGGACCCACTTCTCATTCAATTTGTGGGAATTTTCTGATC 6264

LserUS96UC23_CD 6215 ATTATTTACATGGACCCACTTCTCATTCAATTTGTGGGAATTTTCTGATC 6264

LvirLAC006941_C 6230 ATTATTTACATGGACCCACTTCTCATTCAATTTGTGGGAATTTTCTGATC 6279

LserLAC005780_C 6248 ATTATTTACATGGACCCACTTCTCATTCAATTTGTGGGAATTTTCTGATC 6297

LsalLAC008020_C 6248 ATTATTTACATGGACCCACTTCTCATTCAATTTGTGGGAATTTTCTGATC 6297

LsalCGN5271_CDS 6248 ATTATTTACATGGACCCACTTCTCATTCAATTTGTGGGAATTTTCTGATC 6297

LsatLACWENDEL_C 6265 TATAAACCGTCCCTAACTAACGATCGGCTCATGTTTGAGGATGATGAATC 6314

LsatSalinas_CDS 6265 TATAAACCGTCCCTAACTAACGATCGGCTCATGTTTGAGGATGATGAATC 6314

LserUS96UC23_CD 6265 TATAAACCGTCCCTAACTAACGATCGGCTCATGTTTGAGGATGATGAATC 6314

LvirLAC006941_C 6280 TATAAACCGTCCCTAACTAACGATCGGCTCATGTTTGAGGATGATGAATC 6329

LserLAC005780_C 6298 TATAAACCGTCCCTAACTAACGATCGGCTCATGTTTGAGGATGATGAATC 6347

LsalLAC008020_C 6298 TATAAACCGTCCCTAACTAACGATCGGCTCATGTTTGAGGATGATGAATC 6347

LsalCGN5271_CDS 6298 TATAAACCGTCCCTAACTAACGATCGGCTCATGTTTGAGGATGATGAATC 6347

LsatLACWENDEL_C 6315 ACTTCGTGTCGACCTGTTGCCAATAAACTTTCCGGCCTCATATGAGAATG 6364

LsatSalinas_CDS 6315 ACTTCGTGTCGACCTGTTGCCAATAAACTTTCCGGCCTCATATGAGAATG 6364

LserUS96UC23_CD 6315 ACTTCGTGTCGACCTGTTGCCAATAAACTTTCCGGCCTCATATGAGAATG 6364

LvirLAC006941_C 6330 ACTTCGTGTCGACCTGTTGCCAATAAACTTTCCGGCCTCATATGAGAATG 6379

LserLAC005780_C 6348 ACTTCGTGTCGACCTGTTGCCAATAAACTTTCCGGCCTCATATGAGAATG 6397

LsalLAC008020_C 6348 ACTTCGTGTCGACCTGTTGCCAATAAACTTTCCGGCCTCATATGAGAATG 6397

LsalCGN5271_CDS 6348 ACTTCGTGTCGACCTGTTGCCAATAAACTTTCCGGCCTCATATGAGAATG 6397

LsatLACWENDEL_C 6365 GAAAACTGGAGCATTTTCTGCATCGGTGGATGAAGAATCGCGAACATAAG 6414

LsatSalinas_CDS 6365 GAAAACTGGAGCATTTTCTGCATCGGTGGATGAAGAATCGCGAACATAAG 6414

LserUS96UC23_CD 6365 GAAAACTGGAGCATTTTCTGCATCGGTGGATGAAGAATCGCGAACATAAG 6414

LvirLAC006941_C 6380 GAAAACTGGAGCATTTTCTGCATCGGTGGATGAAGAATCGCGAACATAAG 6429

LserLAC005780_C 6398 GAAAACTGGAGCATTTTCTGCATCGGTGGATGAAGAATCGCGAACATAAG 6447

LsalLAC008020_C 6398 GAAAACTGGAGCATTTTCTGCATCGGTGGATGAAGAATCGCGAACATAAG 6447

LsalCGN5271_CDS 6398 GAAAACTGGAGCATTTTCTGCATCGGTGGATGAAGAATCGCGAACATAAG 6447

LsatLACWENDEL_C 6415 AATTTCTGGTTGACCATGTTCCCAGAAAAAAGATACTTTCAAGAAACGAC 6464

LsatSalinas_CDS 6415 AATTTCTGGTTGACCATGTTCCCAGAAAAAAGATACTTTCAAGAAACGAC 6464

LserUS96UC23_CD 6415 AATTTCTGGTTGACCATGTTCCCAGAAAAAAGATACTTTCAAGAAACGAC 6464

LvirLAC006941_C 6430 AATTTCTGGTTGACCATGTTCCCAGAAAAAAGATACTTTCAAGAAACGAC 6479

LserLAC005780_C 6448 AATTTCTGGTTGACCATGTTCCCAGAAAAAAGATACTTTCAAGAAACGAC 6497

LsalLAC008020_C 6448 AATTTCTGGTTGACCATGTTCCCAGAAAAAAGATACTTTCAAGAAACGAC 6497

LsalCGN5271_CDS 6448 AATTTCTGGTTGACCATGTTCCCAGAAAAAAGATACTTTCAAGAAACGAC 6497

LsatLACWENDEL_C 6465 GAACACGACTGAAGTGGCTATACATACAAATCCATTTACGGATCTATATG 6514

LsatSalinas_CDS 6465 GAACACGACTGAAGTGGCTATACATACAAATCCATTTACGGATCTATATG 6514

LserUS96UC23_CD 6465 GAACACGACTGAAGTGGCTATACATACAAATCCATTTACGGATCTATATG 6514

LvirLAC006941_C 6480 GAACACGACTGAAGTGGCTATACATACAAATCCATTTACGGATCTATATG 6529

LserLAC005780_C 6498 GAACACGACTGAAGTGGCTATACATACAAATCCATTTACGGATCTATATG 6547

LsalLAC008020_C 6498 GAACACGACTGAAGTGGCTATACATACAAATCCATTTACGGATCTATATG 6547

LsalCGN5271_CDS 6498 GAACACGACTGAAGTGGCTATACATACAAATCCATTTACGGATCTATATG 6547

LsatLACWENDEL_C 6515 CTCCGATTGGAACTTCCAGTTCCAGAACAGGTGGCTGGTATACTACCATA 6564

LsatSalinas_CDS 6515 CTCCGATTGGAACTTCCAGTTCCAGAACAGGTGGCTGGTATACTACCATA 6564

LserUS96UC23_CD 6515 CTCCGATTGGAACTTCCAGTTCCAGAACAGGTGGCTGGTATACTACCATA 6564

LvirLAC006941_C 6530 CTCCGATTGGAACTTCCAGTTCCAGAACAGGTGGCTGGTATACTACCATA 6579

LserLAC005780_C 6548 CTCCGATTGGAACTTCCAGTTCCAGAACAGGTGGCTGGTATACTACCATA 6597

LsalLAC008020_C 6548 CTCCGATTGGAACTTCCAGTTCCAGAACAGGTGGCTGGTATACTACCATA 6597

LsalCGN5271_CDS 6548 CTCCGATTGGAACTTCCAGTTCCAGAACAGGTGGCTGGTATACTACCATA 6597

LsatLACWENDEL_C 6565 ATGAAACTGCCTTTTATTTTTTTTATTCGGATCGGATTTATGTTGGCTTC 6614

LsatSalinas_CDS 6565 ATGAAACTGCCTTTTATTTTTTTTATTCGGATCGGATTTATGTTGGCTTC 6614

LserUS96UC23_CD 6565 ATGAAACTGCCTTTTATTTTTTTTATTCGGATCGGATTTATGTTGGCTTC 6614

LvirLAC006941_C 6580 ATGAAACTGCCTTTTATTTTTTTTATTCGGATCGGATTTATGTTGGCTTC 6629

LserLAC005780_C 6598 ATGAAACTGCCTTTTATTTTTTTTATTCGGATCGGATTTATGTTGGCTTC 6647

LsalLAC008020_C 6598 ATGAAACTGCCTTTTATTTTTTTTATTCGGATCGGATTTATGTTGGCTTC 6647

LsalCGN5271_CDS 6598 ATGAAACTGCCTTTTATTTTTTTTATTCGGATCGGATTTATGTTGGCTTC 6647

LsatLACWENDEL_C 6615 GTTGGGAGGATCGCGTAGTTTGTTACGTCAGCTCAAAAAGGATAAGTTGC 6664

LsatSalinas_CDS 6615 GTTGGGAGGATCGCGTAGTTTGTTACGTCAGCTCAAAAAGGATAAGTTGC 6664

LserUS96UC23_CD 6615 GTTGGGAGGATCGCGTAGTTTGTTACGTCAGCTCAAAAAGGATAAGTTGC 6664

LvirLAC006941_C 6630 GTTGGGAGGATCGCGTAGTTTGTTACGTCAGCTCAAAAAGGATAAGTTGC 6679

LserLAC005780_C 6648 GTTGGGAGGATCGCGTAGTTTGTTACGTCAGCTCAAAAAGGATAAGTTGC 6697

LsalLAC008020_C 6648 GTTGGGAGGATCGCGTAGTTTGTTACGTCAGCTCAAAAAGGATAAGTTGC 6697

LsalCGN5271_CDS 6648 GTTGGGAGGATCGCGTAGTTTGTTACGTCAGCTCAAAAAGGATAAGTTGC 6697

LsatLACWENDEL_C 6665 CTTGGAATCGAGAAAGTTACGTGGAGTTCATAATTGCATAAATGAGCACG 6714

LsatSalinas_CDS 6665 CTTGGAATCGAGAAAGTTACGTGGAGTTCATAATTGCATAAATGAGCACG 6714

LserUS96UC23_CD 6665 CTTGGAATCGAGAAAGTTACGTGGAGTTCATAATTGCATAAATGAGCACG 6714

LvirLAC006941_C 6680 CTTGGAATCGAGAAAGTTACGTGGAGTTCATAATTGCATAAATGAGCACG 6729

LserLAC005780_C 6698 CTTGGAATCGAGAAAGTTACGTGGAGTTCATAATTGCATAAATGAGCACG 6747

LsalLAC008020_C 6698 CTTGGAATCGAGAAAGTTACGTGGAGTTCATAATTGCATAAATGAGCACG 6747

LsalCGN5271_CDS 6698 CTTGGAATCGAGAAAGTTACGTGGAGTTCATAATTGCATAAATGAGCACG 6747

LsatLACWENDEL_C 6715 TCAATATATGAATTGTTTCATTATTCGTTATTTCCGGGTCTTTTCGTTGC 6764

LsatSalinas_CDS 6715 TCAATATATGAATTGTTTCATTATTCGTTATTTCCGGGTCTTTTCGTTGC 6764

LserUS96UC23_CD 6715 TCAATATATGAATTGTTTCATTATTCGTTATTTCCGGGTCTTTTCGTTGC 6764

LvirLAC006941_C 6730 TCAATATATGAATTGTTTCATTATTCGTTATTTCCGGGTCTTTTCGTTGC 6779

LserLAC005780_C 6748 TCAATATATGAATTGTTTCATTATTCGTTATTTCCGGGTCTTTTCGTTGC 6797

LsalLAC008020_C 6748 TCAATATATGAATTGTTTCATTATTCGTTATTTCCGGGTCTTTTCGTTGC 6797

LsalCGN5271_CDS 6748 TCAATATATGAATTGTTTCATTATTCGTTATTTCCGGGTCTTTTCGTTGC 6797

LsatLACWENDEL_C 6765 ATTCACTTACAACAAGAAAGAACCACCAGTGTTTGGTGCAGCACCTGCAT 6814

LsatSalinas_CDS 6765 ATTCACTTACAACAAGAAAGAACCACCAGTGTTTGGTGCAGCACCTGCAT 6814

LserUS96UC23_CD 6765 ATTCACTTACAACAAGAAAGAACCACCAGTGTTTGGTGCAGCACCTGCAT 6814

LvirLAC006941_C 6780 ATTCACTTACAACAAGAAAGAACCACCAGTGTTTGGTGCAGCACCTGCAT 6829

LserLAC005780_C 6798 ATTCACTTACAACAAGAAAGAACCACCAGTGTTTGGTGCAGCACCTGCAT 6847

LsalLAC008020_C 6798 ATTCACTTACAACAAGAAAGAACCACCAGTGTTTGGTGCAGCACCTGCAT 6847

LsalCGN5271_CDS 6798 ATTCACTTACAACAAGAAAGAACCACCAGTGTTTGGTGCAGCACCTGCAT 6847

LsatLACWENDEL_C 6815 TTTGGTGCATTCTTCTTTCTTTCCTTGGTCTTCCGTTCCGTCATATTCCT 6864

LsatSalinas_CDS 6815 TTTGGTGCATTCTTCTTTCTTTCCTTGGTCTTCCGTTCCGTCATATTCCT 6864

LserUS96UC23_CD 6815 TTTGGTGCATTCTTCTTTCTTTCCTTGGTCTTCCGTTCCGTCATATTCCT 6864

LvirLAC006941_C 6830 TTTGGTGCATTCTTCTTTCTTTCCTTGGTCTTCCGTTCCGTCATATTCCT 6879

LserLAC005780_C 6848 TTTGGTGCATTCTTCTTTCTTTCCTTGGTCTTCCGTTCCGTCATATTCCT 6897

LsalLAC008020_C 6848 TTTGGTGCATTCTTCTTTCTTTCCTTGGTCTTCCGTTCCGTCATATTCCT 6897

LsalCGN5271_CDS 6848 TTTGGTGCATTCTTCTTTCTTTCCTTGGTCTTCCGTTCCGTCATATTCCT 6897

LsatLACWENDEL_C 6865 AATAACTTATCCAATGACAACGAATTAACCGCAAATGCACCTTTCTTTTA 6914

LsatSalinas_CDS 6865 AATAACTTATCCAATGACAACGAATTAACCGCAAATGCACCTTTCTTTTA 6914

LserUS96UC23_CD 6865 AATAACTTATCCAATGACAACGAATTAACCGCAAATGCACCTTTCTTTTA 6914

LvirLAC006941_C 6880 AATAACTTATCCAATGACAACGAATTAACCGCAAATGCACCTTTCTTTTA 6929

LserLAC005780_C 6898 AATAACTTATCCAATGACAACGAATTAACCGCAAATGCACCTTTCTTTTA 6947

LsalLAC008020_C 6898 AATAACTTATCCAATGACAACGAATTAACCGCAAATGCACCTTTCTTTTA 6947

LsalCGN5271_CDS 6898 AATAACTTATCCAATGACAACGAATTAACCGCAAATGCACCTTTCTTTTA 6947

LsatLACWENDEL_C 6915 TCAAATCTCAGGGACATGGTCTAATCATGAGGGTAGTATTTTATCATGGT 6964

LsatSalinas_CDS 6915 TCAAATCTCAGGGACATGGTCTAATCATGAGGGTAGTATTTTATCATGGT 6964

LserUS96UC23_CD 6915 TCAAATCTCAGGGACATGGTCTAATCATGAGGGTAGTATTTTATCATGGT 6964

LvirLAC006941_C 6930 TCAAATCTCAGGGACATGGTCTAATCATGAGGGTAGTATTTTATCATGGT 6979

LserLAC005780_C 6948 TCAAATCTCAGGGACATGGTCTAATCATGAGGGTAGTATTTTATCATGGT 6997

LsalLAC008020_C 6948 TCAAATCTCAGGGACATGGTCTAATCATGAGGGTAGTATTTTATCATGGT 6997

LsalCGN5271_CDS 6948 TCAAATCTCAGGGACATGGTCTAATCATGAGGGTAGTATTTTATCATGGT 6997

LsatLACWENDEL_C 6965 GTCGGATCCTAAGTTTTTATGGATTCCTTCTTTGTTACCGGGGTCGACCC 7014

LsatSalinas_CDS 6965 GTCGGATCCTAAGTTTTTATGGATTCCTTCTTTGTTACCGGGGTCGACCC 7014

LserUS96UC23_CD 6965 GTCGGATCCTAAGTTTTTATGGATTCCTTCTTTGTTACCGGGGTCGACCC 7014

LvirLAC006941_C 6980 GTCGGATCCTAAGTTTTTATGGATTCCTTCTTTGTTACCGGGGTCGACCC 7029

LserLAC005780_C 6998 GTCGGATCCTAAGTTTTTATGGATTCCTTCTTTGTTACCGGGGTCGACCC 7047

LsalLAC008020_C 6998 GTCGGATCCTAAGTTTTTATGGATTCCTTCTTTGTTACCGGGGTCGACCC 7047

LsalCGN5271_CDS 6998 GTCGGATCCTAAGTTTTTATGGATTCCTTCTTTGTTACCGGGGTCGACCC 7047

LsatLACWENDEL_C 7015 CAAAGCCATAATGTCTCAAAACGAGGAGGCCATAGAGAAAGTCTTTTTTA 7064

LsatSalinas_CDS 7015 CAAAGCCATAATGTCTCAAAACGAGGAGGCCATAGAGAAAGTCTTTTTTA 7064

LserUS96UC23_CD 7015 CAAAGCCATAATGTCTCAAAACGAGGAGGCCATAGAGAAAGTCTTTTTTA 7064

LvirLAC006941_C 7030 CAAAGCCATAATGTCTCAAAACGAGGAGGCCATAGAGAAAGTCTTTTTTA 7079

LserLAC005780_C 7048 CAAAGCCATAATGTCTCAAAACGAGGAGGCCATAGAGAAAGTCTTTTTTA 7097

LsalLAC008020_C 7048 CAAAGCCATAATGTCTCAAAACGAGGAGGCCATAGAGAAAGTCTTTTTTA 7097

LsalCGN5271_CDS 7048 CAAAGCCATAATGTCTCAAAACGAGGAGGCCATAGAGAAAGTCTTTTTTA 7097

LsatLACWENDEL_C 7065 TTCCTTTGTCTCAAACTTCGTGAAGAACTCCATTCTATCTCTCCCTAGTT 7114

LsatSalinas_CDS 7065 TTCCTTTGTCTCAAACTTCGTGAAGAACTCCATTCTATCTCTCCCTAGTT 7114

LserUS96UC23_CD 7065 TTCCTTTGTCTCAAACTTCGTGAAGAACTCCATTCTATCTCTCCCTAGTT 7114

LvirLAC006941_C 7080 TTCCTTTGTCTCAAACTTCGTGAAGAACTCCATTCTATCTCTCCCTAGTT 7129

LserLAC005780_C 7098 TTCCTTTGTCTCAAACTTCGTGAAGAACTCCATTCTATCTCTCCCTAGTT 7147

LsalLAC008020_C 7098 TTCCTTTGTCTCAAACTTCGTGAAGAACTCCATTCTATCTCTCCCTAGTT 7147

LsalCGN5271_CDS 7098 TTCCTTTGTCTCAAACTTCGTGAAGAACTCCATTCTATCTCTCCCTAGTT 7147

LsatLACWENDEL_C 7115 ACGAACAAAAAACTGGGGCTACGACCCAGTTGTACACTCCCTTCGTTCTA 7164

LsatSalinas_CDS 7115 ACGAACAAAAAACTGGGGCTACGACCCAGTTGTACACTCCCTTCGTTCTA 7164

LserUS96UC23_CD 7115 ACGAACAAAAAACTGGGGCTACGACCCAGTTGTACACTCCCTTCGTTCTA 7164

LvirLAC006941_C 7130 ACGAACAAAAAACTGGGGCTACGACCCAGTTGTACACTCCCTTCGTTCTA 7179

LserLAC005780_C 7148 ACGAACAAAAAACTGGGGCTACGACCCAGTTGTACACTCCCTTCGTTCTA 7197

LsalLAC008020_C 7148 ACGAACAAAAAACTGGGGCTACGACCCAGTTGTACACTCCCTTCGTTCTA 7197

LsalCGN5271_CDS 7148 ACGAACAAAAAACTGGGGCTACGACCCAGTTGTACACTCCCTTCGTTCTA 7197

LsatLACWENDEL_C 7165 CGAACCCTTGTTGATTCTGAACTTCGTTCGCGAAGGCGCCGGACTTTTGA 7214

LsatSalinas_CDS 7165 CGAACCCTTGTTGATTCTGAACTTCGTTCGCGAAGGCGCCGGACTTTTGA 7214

LserUS96UC23_CD 7165 CGAACCCTTGTTGATTCTGAACTTCGTTCGCGAAGGCGCCGGACTTTTGA 7214

LvirLAC006941_C 7180 CGAACCCTTGTTGATTCTGAACTTCGTTCGCGAAGGCGCCGGACTTTTGA 7229

LserLAC005780_C 7198 CGAACCCTTGTTGATTCTGAACTTCGTTCGCGAAGGCGCCGGACTTTTGA 7247

LsalLAC008020_C 7198 CGAACCCTTGTTGATTCTGAACTTCGTTCGCGAAGGCGCCGGACTTTTGA 7247

LsalCGN5271_CDS 7198 CGAACCCTTGTTGATTCTGAACTTCGTTCGCGAAGGCGCCGGACTTTTGA 7247

LsatLACWENDEL_C 7215 CGGGCCAGCCCTTTTTTATGCGCTGTTTTCCCAAAGAAGCTTTGCTCCTC 7264

LsatSalinas_CDS 7215 CGGGCCAGCCCTTTTTTATGCGCTGTTTTCCCAAAGAAGCTTTGCTCCTC 7264

LserUS96UC23_CD 7215 CGGGCCAGCCCTTTTTTATGCGCTGTTTTCCCAAAGAAGCTTTGCTCCTC 7264

LvirLAC006941_C 7230 CGGGCCAGCCCTTTTTTATGCGCTGTTTTCCCAAAGAAGCTTTGCTCCTC 7279

LserLAC005780_C 7248 CGGGCCAGCCCTTTTTTATGCGCTGTTTTCCCAAAGAAGCTTTGCTCCTC 7297

LsalLAC008020_C 7248 CGGGCCAGCCCTTTTTTATGCGCTGTTTTCCCAAAGAAGCTTTGCTCCTC 7297

LsalCGN5271_CDS 7248 CGGGCCAGCCCTTTTTTATGCGCTGTTTTCCCAAAGAAGCTTTGCTCCTC 7297

LsatLACWENDEL_C 7265 TGGGTGCTAGGCGCTTCCGTGGTTCGCGAGAAGGAAAAAGGACTCATCCT 7314

LsatSalinas_CDS 7265 TGGGTGCTAGGCGCTTCCGTGGTTCGCGAGAAGGAAAAAGGACTCATCCT 7314

LserUS96UC23_CD 7265 TGGGTGCTAGGCGCTTCCGTGGTTCGCGAGAAGGAAAAAGGACTCATCCT 7314

LvirLAC006941_C 7280 TGGGTGCTAGGCGCTTCCGTGGTTCGCGAGAAGGAAAAAGGACTCATCCT 7329

LserLAC005780_C 7298 TGGGTGCTAGGCGCTTCCGTGGTTCGCGAGAAGGAAAAAGGACTCATCCT 7347

LsalLAC008020_C 7298 TGGGTGCTAGGCGCTTCCGTGGTTCGCGAGAAGGAAAAAGGACTCATCCT 7347

LsalCGN5271_CDS 7298 TGGGTGCTAGGCGCTTCCGTGGTTCGCGAGAAGGAAAAAGGACTCATCCT 7347

LsatLACWENDEL_C 7315 TTGTTGCATCTGGCGCGAGATGATAAAGAGAGAGCTTCGTCTATCGATGA 7364

LsatSalinas_CDS 7315 TTGTTGCATCTGGCGCGAGATGATAAAGAGAGAGCTTCGTCTATCGATGA 7364

LserUS96UC23_CD 7315 TTGTTGCATCTGGCGCGAGATGATAAAGAGAGAGCTTCGTCTATCGATGA 7364

LvirLAC006941_C 7330 TTGTTGCATCTGGCGCGAGATGATAAAGAGAGAGCTTCGTCTATCGATGA 7379

LserLAC005780_C 7348 TTGTTGCATCTGGCGCGAGATGATAAAGAGAGAGCTTCGTCTATCGATGA 7397

LsalLAC008020_C 7348 TTGTTGCATCTGGCGCGAGATGATAAAGAGAGAGCTTCGTCTATCGATGA 7397

LsalCGN5271_CDS 7348 TTGTTGCATCTGGCGCGAGATGATAAAGAGAGAGCTTCGTCTATCGATGA 7397

LsatLACWENDEL_C 7365 ACAGCGGATTGACGGAGCTCTTGGCATTGCTTTGTTTTTCTCTCCTTTCC 7414

LsatSalinas_CDS 7365 ACAGCGGATTGACGGAGCTCTTGGCATTGCTTTGTTTTTCTCTCCTTTCC 7414

LserUS96UC23_CD 7365 ACAGCGGATTGACGGAGCTCTTGGCATTGCTTTGTTTTTCTCTCCTTTCC 7414

LvirLAC006941_C 7380 ACAGCGGATTGACGGAGCTCTTGGCATTGCTTTGTTTTTCTCTCCTTTCC 7429

LserLAC005780_C 7398 ACAGCGGATTGACGGAGCTCTTGGCATTGCTTTGTTTTTCTCTCCTTTCC 7447

LsalLAC008020_C 7398 ACAGCGGATTGACGGAGCTCTTGGCATTGCTTTGTTTTTCTCTCCTTTCC 7447

LsalCGN5271_CDS 7398 ACAGCGGATTGACGGAGCTCTTGGCATTGCTTTGTTTTTCTCTCCTTTCC 7447

LsatLACWENDEL_C 7415 TATCGGCGAGTTCCGACCCTTTTGTTCGAAATTTCTTCGTTCGTACCGAA 7464

LsatSalinas_CDS 7415 TATCGGCGAGTTCCGACCCTTTTGTTCGAAATTTCTTCGTTCGTACCGAA 7464

LserUS96UC23_CD 7415 TATCGGCGAGTTCCGACCCTTTTGTTCGAAATTTCTTCGTTCGTACCGAA 7464

LvirLAC006941_C 7430 TATCGGCGAGTTCCGACCCTTTTGTTCGAAATTTCTTCGTTCGTACCGAA 7479

LserLAC005780_C 7448 TATCGGCGAGTTCCGACCCTTTTGTTCGAAATTTCTTCGTTCGTACCGAA 7497

LsalLAC008020_C 7448 TATCGGCGAGTTCCGACCCTTTTGTTCGAAATTTCTTCGTTCGTACCGAA 7497

LsalCGN5271_CDS 7448 TATCGGCGAGTTCCGACCCTTTTGTTCGAAATTTCTTCGTTCGTACCGAA 7497

LsatLACWENDEL_C 7465 CCGCTTGCAGAATCAAATCCTGTTCCACAAGATCCTATATCAGCTATACA 7514

LsatSalinas_CDS 7465 CCGCTTGCAGAATCAAATCCTGTTCCACAAGATCCTATATCAGCTATACA 7514

LserUS96UC23_CD 7465 CCGCTTGCAGAATCAAATCCTGTTCCACAAGATCCTATATCAGCTATACA 7514

LvirLAC006941_C 7480 CCGCTTGCAGAATCAAATCCTGTTCCACAAGATCCTATATCAGCTATACA 7529

LserLAC005780_C 7498 CCGCTTGCAGAATCAAATCCTGTTCCACAAGATCCTATATCAGCTATACA 7547

LsalLAC008020_C 7498 CCGCTTGCAGAATCAAATCCTGTTCCACAAGATCCTATATCAGCTATACA 7547

LsalCGN5271_CDS 7498 CCGCTTGCAGAATCAAATCCTGTTCCACAAGATCCTATATCAGCTATACA 7547

LsatLACWENDEL_C 7515 TCCTCCTTGCATTTATGCCGGAGACGTCGCCAGTGCTATGGGCTTTGGCT 7564

LsatSalinas_CDS 7515 TCCTCCTTGCATTTATGCCGGAGACGTCGCCAGTGCTATGGGCTTTGGCT 7564

LserUS96UC23_CD 7515 TCCTCCTTGCATTTATGCCGGAGACGTCGCCAGTGCTATGGGCTTTGGCT 7564

LvirLAC006941_C 7530 TCCTCCTTGCATTTATGCCGGAGACGTCGCCAGTGCTATGGGCTTTGGCT 7579

LserLAC005780_C 7548 TCCTCCTTGCATTTATGCCGGAGACGTCGCCAGTGCTATGGGCTTTGGCT 7597

LsalLAC008020_C 7548 TCCTCCTTGCATTTATGCCGGAGACGTCGCCAGTGCTATGGGCTTTGGCT 7597

LsalCGN5271_CDS 7548 TCCTCCTTGCATTTATGCCGGAGACGTCGCCAGTGCTATGGGCTTTGGCT 7597

LsatLACWENDEL_C 7565 TATGTAGATCAAAAATGATGAATGGGATTGTGGCACTCCACTCGCCGCCA 7614

LsatSalinas_CDS 7565 TATGTAGATCAAAAATGATGAATGGGATTGTGGCACTCCACTCGCCGCCA 7614

LserUS96UC23_CD 7565 TATGTAGATCAAAAATGATGAATGGGATTGTGGCACTCCACTCGCCGCCA 7614

LvirLAC006941_C 7580 TATGTAGATCAAAAATGATGAATGGGATTGTGGCACTCCACTCGCCGCCA 7629

LserLAC005780_C 7598 TATGTAGATCAAAAATGATGAATGGGATTGTGGCACTCCACTCGCCGCCA 7647

LsalLAC008020_C 7598 TATGTAGATCAAAAATGATGAATGGGATTGTGGCACTCCACTCGCCGCCA 7647

LsalCGN5271_CDS 7598 TATGTAGATCAAAAATGATGAATGGGATTGTGGCACTCCACTCGCCGCCA 7647

LsatLACWENDEL_C 7615 ATGCGGAAGGATGACGCCGAAAAGAATGGAAGGCTCTTTCGCTCTGCTGC 7664

LsatSalinas_CDS 7615 ATGCGGAAGGATGACGCCGAAAAGAATGGAAGGCTCTTTCGCTCTGCTGC 7664

LserUS96UC23_CD 7615 ATGCGGAAGGATGACGCCGAAAAGAATGGAAGGCTCTTTCGCTCTGCTGC 7664

LvirLAC006941_C 7630 ATGCGGAAGGATGACGCCGAAAAGAATGGAAGGCTCTTTCGCTCTGCTGC 7679

LserLAC005780_C 7648 ATGCGGAAGGATGACGCCGAAAAGAATGGAAGGCTCTTTCGCTCTGCTGC 7697

LsalLAC008020_C 7648 ATGCGGAAGGATGACGCCGAAAAGAATGGAAGGCTCTTTCGCTCTGCTGC 7697

LsalCGN5271_CDS 7648 ATGCGGAAGGATGACGCCGAAAAGAATGGAAGGCTCTTTCGCTCTGCTGC 7697

LsatLACWENDEL_C 7665 ATGCGTCGGATCCCGTAGAACAAGCAAGCTCTTTACCCTAAAATTAAAAC 7714

LsatSalinas_CDS 7665 ATGCGTCGGATCCCGTAGAACAAGCAAGCTCTTTACCCTAAAATTAAAAC 7714

LserUS96UC23_CD 7665 ATGCGTCGGATCCCGTAGAACAAGCAAGCTCTTTACCCTAAAATTAAAAC 7714

LvirLAC006941_C 7680 ATGCGTCGGATCCCGTAGAACAAGCAAGCTCTTTACCCTAAAATTAAAAC 7729

LserLAC005780_C 7698 ATGCGTCGGATCCCGTAGAACAAGCAAGCTCTTTACCCTAAAATTAAAAC 7747

LsalLAC008020_C 7698 ATGCGTCGGATCCCGTAGAACAAGCAAGCTCTTTACCCTAAAATTAAAAC 7747

LsalCGN5271_CDS 7698 ATGCGTCGGATCCCGTAGAACAAGCAAGCTCTTTACCCTAAAATTAAAAC 7747

LsatLACWENDEL_C 7715 ATATGGGCGCAAAAGGCTATCCTGCTCTATTGTTACGTAGCAATAGAAGC 7764

LsatSalinas_CDS 7715 ATATGGGCGCAAAAGGCTATCCTGCTCTATTGTTACGTAGCAATAGAAGC 7764

LserUS96UC23_CD 7715 ATATGGGCGCAAAAGGCTATCCTGCTCTATTGTTACGTAGCAATAGAAGC 7764

LvirLAC006941_C 7730 ATATGGGCGCAAAAGGCTATCCTGCTCTATTGTTACGTAGCAATAGAAGC 7779

LserLAC005780_C 7748 ATATGGGCGCAAAAGGCTATCCTGCTCTATTGTTACGTAGCAATAGAAGC 7797

LsalLAC008020_C 7748 ATATGGGCGCAAAAGGCTATCCTGCTCTATTGTTACGTAGCAATAGAAGC 7797

LsalCGN5271_CDS 7748 ATATGGGCGCAAAAGGCTATCCTGCTCTATTGTTACGTAGCAATAGAAGC 7797

LsatLACWENDEL_C 7765 CTGCTTATGTTGCTTCGGCGGCGTTTCGCCTTCTCTTCGCTCTGGACAGG 7814

LsatSalinas_CDS 7765 CTGCTTATGTTGCTTCGGCGGCGTTTCGCCTTCTCTTCGCTCTGGACAGG 7814

LserUS96UC23_CD 7765 CTGCTTATGTTGCTTCGGCGGCGTTTCGCCTTCTCTTCGCTCTGGACAGG 7814

LvirLAC006941_C 7780 CTGCTTATGTTGCTTCGGCGGCGTTTCGCCTTCTCTTCGCTCTGGACAGG 7829

LserLAC005780_C 7798 CTGCTTATGTTGCTTCGGCGGCGTTTCGCCTTCTCTTCGCTCTGGACAGG 7847

LsalLAC008020_C 7798 CTGCTTATGTTGCTTCGGCGGCGTTTCGCCTTCTCTTCGCTCTGGACAGG 7847

LsalCGN5271_CDS 7798 CTGCTTATGTTGCTTCGGCGGCGTTTCGCCTTCTCTTCGCTCTGGACAGG 7847

LsatLACWENDEL_C 7815 AGCGCTAGTGGACACGGGGAGGGAGCAGGCGAAGCGTGTCGTTCGTAATG 7864

LsatSalinas_CDS 7815 AGCGCTAGTGGACACGGGGAGGGAGCAGGCGAAGCGTGTCGTTCGTAATG 7864

LserUS96UC23_CD 7815 AGCGCTAGTGGACACGGGGAGGGAGCAGGCGAAGCGTGTCGTTCGTAATG 7864

LvirLAC006941_C 7830 AGCGCTAGTGGACACGGGGAGGGAGCAGGCGAAGCGTGTCGTTCGTAATG 7879

LserLAC005780_C 7848 AGCGCTAGTGGACACGGGGAGGGAGCAGGCGAAGCGTGTCGTTCGTAATG 7897

LsalLAC008020_C 7848 AGCGCTAGTGGACACGGGGAGGGAGCAGGCGAAGCGTGTCGTTCGTAATG 7897

LsalCGN5271_CDS 7848 AGCGCTAGTGGACACGGGGAGGGAGCAGGCGAAGCGTGTCGTTCGTAATG 7897

LsatLACWENDEL_C 7865 GAAAGAAAGAGACCACTACTTCGCTTCTTTGTTGGATCGCCGGCGCGAAC 7914

LsatSalinas_CDS 7865 GAAAGAAAGAGACCACTACTTCGCTTCTTTGTTGGATCGCCGGCGCGAAC 7914

LserUS96UC23_CD 7865 GAAAGAAAGAGACCACTACTTCGCTTCTTTGTTGGATCGCCGGCGCGAAC 7914

LvirLAC006941_C 7880 GAAAGAAAGAGACCACTACTTCGCTTCTTTGTTGGATCGCCGGCGCGAAC 7929

LserLAC005780_C 7898 GAAAGAAAGAGACCACTACTTCGCTTCTTTGTTGGATCGCCGGCGCGAAC 7947

LsalLAC008020_C 7898 GAAAGAAAGAGACCACTACTTCGCTTCTTTGTTGGATCGCCGGCGCGAAC 7947

LsalCGN5271_CDS 7898 GAAAGAAAGAGACCACTACTTCGCTTCTTTGTTGGATCGCCGGCGCGAAC 7947

LsatLACWENDEL_C 7915 ACAGTGGTCTCTGACCAGGACCAGGAACCAATTCGAATTTTGATCTTGAC 7964

LsatSalinas_CDS 7915 ACAGTGGTCTCTGACCAGGACCAGGAACCAATTCGAATTTTGATCTTGAC 7964

LserUS96UC23_CD 7915 ACAGTGGTCTCTGACCAGGACCAGGAACCAATTCGAATTTTGATCTTGAC 7964

LvirLAC006941_C 7930 ACAGTGGTCTCTGACCAGGACCAGGAACCAATTCGAATTTTGATCTTGAC 7979

LserLAC005780_C 7948 ACAGTGGTCTCTGACCAGGACCAGGAACCAATTCGAATTTTGATCTTGAC 7997

LsalLAC008020_C 7948 ACAGTGGTCTCTGACCAGGACCAGGAACCAATTCGAATTTTGATCTTGAC 7997

LsalCGN5271_CDS 7948 ACAGTGGTCTCTGACCAGGACCAGGAACCAATTCGAATTTTGATCTTGAC 7997

LsatLACWENDEL_C 7965 ATGTCGGTGGTTTTTAACCGTAGGCATTTTGCCAGGAAGTTGGTGGGCTC 8014

LsatSalinas_CDS 7965 ATGTCGGTGGTTTTTAACCGTAGGCATTTTGCCAGGAAGTTGGTGGGCTC 8014

LserUS96UC23_CD 7965 ATGTCGGTGGTTTTTAACCGTAGGCATTTTGCCAGGAAGTTGGTGGGCTC 8014

LvirLAC006941_C 7980 ATGTCGGTGGTTTTTAACCGTAGGCATTTTGCCAGGAAGTTGGTGGGCTC 8029

LserLAC005780_C 7998 ATGTCGGTGGTTTTTAACCGTAGGCATTTTGCCAGGAAGTTGGTGGGCTC 8047

LsalLAC008020_C 7998 ATGTCGGTGGTTTTTAACCGTAGGCATTTTGCCAGGAAGTTGGTGGGCTC 8047

LsalCGN5271_CDS 7998 ATGTCGGTGGTTTTTAACCGTAGGCATTTTGCCAGGAAGTTGGTGGGCTC 8047

LsatLACWENDEL_C 8015 ATCATGAATTAGGTCGGGGTGGCTGGTGGTTTCGGGATCCCGTGGAAAAT 8064

LsatSalinas_CDS 8015 ATCATGAATTAGGTCGGGGTGGCTGGTGGTTTCGGGATCCCGTGGAAAAT 8064

LserUS96UC23_CD 8015 ATCATGAATTAGGTCGGGGTGGCTGGTGGTTTCGGGATCCCGTGGAAAAT 8064

LvirLAC006941_C 8030 ATCATGAATTAGGTCGGGGTGGCTGGTGGTTTCGGGATCCCGTGGAAAAT 8079

LserLAC005780_C 8048 ATCATGAATTAGGTCGGGGTGGCTGGTGGTTTCGGGATCCCGTGGAAAAT 8097

LsalLAC008020_C 8048 ATCATGAATTAGGTCGGGGTGGCTGGTGGTTTCGGGATCCCGTGGAAAAT 8097

LsalCGN5271_CDS 8048 ATCATGAATTAGGTCGGGGTGGCTGGTGGTTTCGGGATCCCGTGGAAAAT 8097

LsatLACWENDEL_C 8065 GCTTCTTTTATGCCTCGGGTATTAGCTACAGCTCGTATTCATTCAGTAAT 8114

LsatSalinas_CDS 8065 GCTTCTTTTATGCCTCGGGTATTAGCTACAGCTCGTATTCATTCAGTAAT 8114

LserUS96UC23_CD 8065 GCTTCTTTTATGCCTCGGGTATTAGCTACAGCTCGTATTCATTCAGTAAT 8114

LvirLAC006941_C 8080 GCTTCTTTTATGCCTCGGGTATTAGCTACAGCTCGTATTCATTCAGTAAT 8129

LserLAC005780_C 8098 GCTTCTTTTATGCCTCGGGTATTAGCTACAGCTCGTATTCATTCAGTAAT 8147

LsalLAC008020_C 8098 GCTTCTTTTATGCCTCGGGTATTAGCTACAGCTCGTATTCATTCAGTAAT 8147

LsalCGN5271_CDS 8098 GCTTCTTTTATGCCTCGGGTATTAGCTACAGCTCGTATTCATTCAGTAAT 8147

LsatLACWENDEL_C 8115 TCTACCCCTTCTTCATTCTTGGACCTCGTTTCTGAATATTGTGACTCTTC 8164

LsatSalinas_CDS 8115 TCTACCCCTTCTTCATTCTTGGACCTCGTTTCTGAATATTGTGACTCTTC 8164

LserUS96UC23_CD 8115 TCTACCCCTTCTTCATTCTTGGACCTCGTTTCTGAATATTGTGACTCTTC 8164

LvirLAC006941_C 8130 TCTACCCCTTCTTCATTCTTGGACCTCGTTTCTGAATATTGTGACTCTTC 8179

LserLAC005780_C 8148 TCTACCCCTTCTTCATTCTTGGACCTCGTTTCTGAATATTGTGACTCTTC 8197

LsalLAC008020_C 8148 TCTACCCCTTCTTCATTCTTGGACCTCGTTTCTGAATATTGTGACTCTTC 8197

LsalCGN5271_CDS 8148 TCTACCCCTTCTTCATTCTTGGACCTCGTTTCTGAATATTGTGACTCTTC 8197

LsatLACWENDEL_C 8165 CATGCTGTGTCTCAGGAACCTCTTCAATACGGTCCGGATTGCTAGCTCCC 8214

LsatSalinas_CDS 8165 CATGCTGTGTCTCAGGAACCTCTTCAATACGGTCCGGATTGCTAGCTCCC 8214

LserUS96UC23_CD 8165 CATGCTGTGTCTCAGGAACCTCTTCAATACGGTCCGGATTGCTAGCTCCC 8214

LvirLAC006941_C 8180 CATGCTGTGTCTCAGGAACCTCTTCAATACGGTCCGGATTGCTAGCTCCC 8229

LserLAC005780_C 8198 CATGCTGTGTCTCAGGAACCTCTTCAATACGGTCCGGATTGCTAGCTCCC 8247

LsalLAC008020_C 8198 CATGCTGTGTCTCAGGAACCTCTTCAATACGGTCCGGATTGCTAGCTCCC 8247

LsalCGN5271_CDS 8198 CATGCTGTGTCTCAGGAACCTCTTCAATACGGTCCGGATTGCTAGCTCCC 8247

LsatLACWENDEL_C 8215 GTTCATAATTTTGCTACAGATGATACACGAGGAATCTTTTTATGGTGGTT 8264

LsatSalinas_CDS 8215 GTTCATAATTTTGCTACAGATGATACACGAGGAATCTTTTTATGGTGGTT 8264

LserUS96UC23_CD 8215 GTTCATAATTTTGCTACAGATGATACACGAGGAATCTTTTTATGGTGGTT 8264

LvirLAC006941_C 8230 GTTCATAATTTTGCTACAGATGATACACGAGGAATCTTTTTATGGTGGTT 8279

LserLAC005780_C 8248 GTTCATAATTTTGCTACAGATGATACACGAGGAATCTTTTTATGGTGGTT 8297

LsalLAC008020_C 8248 GTTCATAATTTTGCTACAGATGATACACGAGGAATCTTTTTATGGTGGTT 8297

LsalCGN5271_CDS 8248 GTTCATAATTTTGCTACAGATGATACACGAGGAATCTTTTTATGGTGGTT 8297

LsatLACWENDEL_C 8265 CTTCCTTCTAATGACCGGCATATCTATGATTATTTTCTCCCAGATGAAGC 8314

LsatSalinas_CDS 8265 CTTCCTTCTAATGACCGGCATATCTATGATTATTTTCTCCCAGATGAAGC 8314

LserUS96UC23_CD 8265 CTTCCTTCTAATGACCGGCATATCTATGATTATTTTCTCCCAGATGAAGC 8314

LvirLAC006941_C 8280 CTTCCTTCTAATGACCGGCATATCTATGATTATTTTCTCCCAGATGAAGC 8329

LserLAC005780_C 8298 CTTCCTTCTAATGACCGGCATATCTATGATTATTTTCTCCCAGATGAAGC 8347

LsalLAC008020_C 8298 CTTCCTTCTAATGACCGGCATATCTATGATTATTTTCTCCCAGATGAAGC 8347

LsalCGN5271_CDS 8298 CTTCCTTCTAATGACCGGCATATCTATGATTATTTTCTCCCAGATGAAGC 8347

LsatLACWENDEL_C 8315 AGCAGGCATCGGTCCGTAGAACCTATAAAAAAGAGATGGTTGTGGCGCGA 8364

LsatSalinas_CDS 8315 AGCAGGCATCGGTCCGTAGAACCTATAAAAAAGAGATGGTTGTGGCGCGA 8364

LserUS96UC23_CD 8315 AGCAGGCATCGGTCCGTAGAACCTATAAAAAAGAGATGGTTGTGGCGCGA 8364

LvirLAC006941_C 8330 AGCAGGCATCGGTCCGTAGAACCTATAAAAAAGAGATGGTTGTGGCGCGA 8379

LserLAC005780_C 8348 AGCAGGCATCGGTCCGTAGAACCTATAAAAAAGAGATGGTTGTGGCGCGA 8397

LsalLAC008020_C 8348 AGCAGGCATCGGTCCGTAGAACCTATAAAAAAGAGATGGTTGTGGCGCGA 8397

LsalCGN5271_CDS 8348 AGCAGGCATCGGTCCGTAGAACCTATAAAAAAGAGATGGTTGTGGCGCGA 8397

LsatLACWENDEL_C 8365 AGTACTCTTGTGCACCTACGTCACTCGGCTCGCGCGCAACCCCGCCCCGT 8414

LsatSalinas_CDS 8365 AGTACTCTTGTGCACCTACGTCACTCGGCTCGCGCGCAACCCCGCCCCGT 8414

LserUS96UC23_CD 8365 AGTACTCTTGTGCACCTACGTCACTCGGCTCGCGCGCAACCCCGCCCCGT 8414

LvirLAC006941_C 8380 AGTACTCTTGTGCACCTACGTCACTCGGCTCGCGCGCAACCCCGCCCCGT 8429

LserLAC005780_C 8398 AGTACTCTTGTGCACCTACGTCACTCGGCTCGCGCGCAACCCCGCCCCGT 8447

LsalLAC008020_C 8398 AGTACTCTTGTGCACCTACGTCACTCGGCTCGCGCGCAACCCCGCCCCGT 8447

LsalCGN5271_CDS 8398 AGTACTCTTGTGCACCTACGTCACTCGGCTCGCGCGCAACCCCGCCCCGT 8447

LsatLACWENDEL_C 8415 TATGTTATAGATGACTATAAGGAACCAACGATTATCGATTCTTAAACAAC 8464

LsatSalinas_CDS 8415 TATGTTATAGATGACTATAAGGAACCAACGATTATCGATTCTTAAACAAC 8464

LserUS96UC23_CD 8415 TATGTTATAGATGACTATAAGGAACCAACGATTATCGATTCTTAAACAAC 8464

LvirLAC006941_C 8430 TATGTTATAGATGACTATAAGGAACCAACGATTATCGATTCTTAAACAAC 8479

LserLAC005780_C 8448 TATGTTATAGATGACTATAAGGAACCAACGATTATCGATTCTTAAACAAC 8497

LsalLAC008020_C 8448 TATGTTATAGATGACTATAAGGAACCAACGATTATCGATTCTTAAACAAC 8497

LsalCGN5271_CDS 8448 TATGTTATAGATGACTATAAGGAACCAACGATTATCGATTCTTAAACAAC 8497

LsatLACWENDEL_C 8465 CTATATTCTCCACACTTAATCAGCATTTGATAGATTATCCAACCCCGAGC 8514

LsatSalinas_CDS 8465 CTATATTCTCCACACTTAATCAGCATTTGATAGATTATCCAACCCCGAGC 8514

LserUS96UC23_CD 8465 CTATATTCTCCACACTTAATCAGCATTTGATAGATTATCCAACCCCGAGC 8514

LvirLAC006941_C 8480 CTATATTCTCCACACTTAATCAGCATTTGATAGATTATCCAACCCCGAGC 8529

LserLAC005780_C 8498 CTATATTCTCCACACTTAATCAGCATTTGATAGATTATCCAACCCCGAGC 8547

LsalLAC008020_C 8498 CTATATTCTCCACACTTAATCAGCATTTGATAGATTATCCAACCCCGAGC 8547

LsalCGN5271_CDS 8498 CTATATTCTCCACACTTAATCAGCATTTGATAGATTATCCAACCCCGAGC 8547

LsatLACWENDEL_C 8515 AATCTTAGTTATTGGTGGGGGTTCGGTCCGTTAGCTGGTATTTGTTTAGT 8564

LsatSalinas_CDS 8515 AATCTTAGTTATTGGTGGGGGTTCGGTCCGTTAGCTGGTATTTGTTTAGT 8564

LserUS96UC23_CD 8515 AATCTTAGTTATTGGTGGGGGTTCGGTCCGTTAGCTGGTATTTGTTTAGT 8564

LvirLAC006941_C 8530 AATCTTAGTTATTGGTGGGGGTTCGGTCCGTTAGCTGGTATTTGTTTAGT 8579

LserLAC005780_C 8548 AATCTTAGTTATTGGTGGGGGTTCGGTCCGTTAGCTGGTATTTGTTTAGT 8597

LsalLAC008020_C 8548 AATCTTAGTTATTGGTGGGGGTTCGGTCCGTTAGCTGGTATTTGTTTAGT 8597

LsalCGN5271_CDS 8548 AATCTTAGTTATTGGTGGGGGTTCGGTCCGTTAGCTGGTATTTGTTTAGT 8597

LsatLACWENDEL_C 8565 CATTCAGATAGTGACTGGCGTTTTTTTAGCTATGCATTACACACCTCATG 8614

LsatSalinas_CDS 8565 CATTCAGATAGTGACTGGCGTTTTTTTAGCTATGCATTACACACCTCATG 8614

LserUS96UC23_CD 8565 CATTCAGATAGTGACTGGCGTTTTTTTAGCTATGCATTACACACCTCATG 8614

LvirLAC006941_C 8580 CATTCAGATAGTGACTGGCGTTTTTTTAGCTATGCATTACACACCTCATG 8629

LserLAC005780_C 8598 CATTCAGATAGTGACTGGCGTTTTTTTAGCTATGCATTACACACCTCATG 8647

LsalLAC008020_C 8598 CATTCAGATAGTGACTGGCGTTTTTTTAGCTATGCATTACACACCTCATG 8647

LsalCGN5271_CDS 8598 CATTCAGATAGTGACTGGCGTTTTTTTAGCTATGCATTACACACCTCATG 8647

LsatLACWENDEL_C 8615 TGGATCTAGCTTTCAACAGCGTAGAACACATTATGAGAGATGTTGAAGGG 8664

LsatSalinas_CDS 8615 TGGATCTAGCTTTCAACAGCGTAGAACACATTATGAGAGATGTTGAAGGG 8664

LserUS96UC23_CD 8615 TGGATCTAGCTTTCAACAGCGTAGAACACATTATGAGAGATGTTGAAGGG 8664

LvirLAC006941_C 8630 TGGATCTAGCTTTCAACAGCGTAGAACACATTATGAGAGATGTTGAAGGG 8679

LserLAC005780_C 8648 TGGATCTAGCTTTCAACAGCGTAGAACACATTATGAGAGATGTTGAAGGG 8697

LsalLAC008020_C 8648 TGGATCTAGCTTTCAACAGCGTAGAACACATTATGAGAGATGTTGAAGGG 8697

LsalCGN5271_CDS 8648 TGGATCTAGCTTTCAACAGCGTAGAACACATTATGAGAGATGTTGAAGGG 8697

LsatLACWENDEL_C 8665 GGCTGGTTGCTCCGTTATATGCATGCTAATGGGGCAAGTATGTTTCTCAT 8714

LsatSalinas_CDS 8665 GGCTGGTTGCTCCGTTATATGCATGCTAATGGGGCAAGTATGTTTCTCAT 8714

LserUS96UC23_CD 8665 GGCTGGTTGCTCCGTTATATGCATGCTAATGGGGCAAGTATGTTTCTCAT 8714

LvirLAC006941_C 8680 GGCTGGTTGCTCCGTTATATGCATGCTAATGGGGCAAGTATGTTTCTCAT 8729

LserLAC005780_C 8698 GGCTGGTTGCTCCGTTATATGCATGCTAATGGGGCAAGTATGTTTCTCAT 8747

LsalLAC008020_C 8698 GGCTGGTTGCTCCGTTATATGCATGCTAATGGGGCAAGTATGTTTCTCAT 8747

LsalCGN5271_CDS 8698 GGCTGGTTGCTCCGTTATATGCATGCTAATGGGGCAAGTATGTTTCTCAT 8747

LsatLACWENDEL_C 8715 TGTGGTTCACCTTCATATTTTTCGTGGTCTATATCATGCGAGTTATAGCA 8764

LsatSalinas_CDS 8715 TGTGGTTCACCTTCATATTTTTCGTGGTCTATATCATGCGAGTTATAGCA 8764

LserUS96UC23_CD 8715 TGTGGTTCACCTTCATATTTTTCGTGGTCTATATCATGCGAGTTATAGCA 8764

LvirLAC006941_C 8730 TGTGGTTCACCTTCATATTTTTCGTGGTCTATATCATGCGAGTTATAGCA 8779

LserLAC005780_C 8748 TGTGGTTCACCTTCATATTTTTCGTGGTCTATATCATGCGAGTTATAGCA 8797

LsalLAC008020_C 8748 TGTGGTTCACCTTCATATTTTTCGTGGTCTATATCATGCGAGTTATAGCA 8797

LsalCGN5271_CDS 8748 TGTGGTTCACCTTCATATTTTTCGTGGTCTATATCATGCGAGTTATAGCA 8797

LsatLACWENDEL_C 8765 GTCCTAGGGAATTTGTTCGGTGTCTCGGAGTTGTAATCTTCCTATTAATG 8814

LsatSalinas_CDS 8765 GTCCTAGGGAATTTGTTCGGTGTCTCGGAGTTGTAATCTTCCTATTAATG 8814

LserUS96UC23_CD 8765 GTCCTAGGGAATTTGTTCGGTGTCTCGGAGTTGTAATCTTCCTATTAATG 8814

LvirLAC006941_C 8780 GTCCTAGGGAATTTGTTCGGTGTCTCGGAGTTGTAATCTTCCTATTAATG 8829

LserLAC005780_C 8798 GTCCTAGGGAATTTGTTCGGTGTCTCGGAGTTGTAATCTTCCTATTAATG 8847

LsalLAC008020_C 8798 GTCCTAGGGAATTTGTTCGGTGTCTCGGAGTTGTAATCTTCCTATTAATG 8847

LsalCGN5271_CDS 8798 GTCCTAGGGAATTTGTTCGGTGTCTCGGAGTTGTAATCTTCCTATTAATG 8847

LsatLACWENDEL_C 8815 ATTGTGACAGCTTTTATAGGATACGTACCGCCTTGGGGTCAGATGAGCTT 8864

LsatSalinas_CDS 8815 ATTGTGACAGCTTTTATAGGATACGTACCGCCTTGGGGTCAGATGAGCTT 8864

LserUS96UC23_CD 8815 ATTGTGACAGCTTTTATAGGATACGTACCGCCTTGGGGTCAGATGAGCTT 8864

LvirLAC006941_C 8830 ATTGTGACAGCTTTTATAGGATACGTACCGCCTTGGGGTCAGATGAGCTT 8879

LserLAC005780_C 8848 ATTGTGACAGCTTTTATAGGATACGTACCACCTTGGGGTCAGATGAGCTT 8897

LsalLAC008020_C 8848 ATTGTGACAGCTTTTATAGGATACGTACCACCTTGGGGTCAGATGAGCTT 8897

LsalCGN5271_CDS 8848 ATTGTGACAGCTTTTATAGGATACGTACCACCTTGGGGTCAGATGAGCTT 8897

LsatLACWENDEL_C 8865 TTGGGGGGCTACAGTAATTACAAGCTTAGCTAGCGCCATACCCGTAGTAG 8914

LsatSalinas_CDS 8865 TTGGGGGGCTACAGTAATTACAAGCTTAGCTAGCGCCATACCCGTAGTAG 8914

LserUS96UC23_CD 8865 TTGGGGGGCTACAGTAATTACAAGCTTAGCTAGCGCCATACCCGTAGTAG 8914

LvirLAC006941_C 8880 TTGGGGGGCTACAGTAATTACAAGCTTAGCTAGCGCCATACCCGTAGTAG 8929

LserLAC005780_C 8898 TTGGGGGGCTACAGTAATTACAAGCTTAGCTAGCGCCATACCCGTAGTAG 8947

LsalLAC008020_C 8898 TTGGGGGGCTACAGTAATTACAAGCTTAGCTAGCGCCATACCCGTAGTAG 8947

LsalCGN5271_CDS 8898 TTGGGGGGCTACAGTAATTACAAGCTTAGCTAGCGCCATACCCGTAGTAG 8947

LsatLACWENDEL_C 8915 GAGATACCATAGTGACTTGGCTTTGGGGTGGTTTCTCCGTGGACAATGCC 8964

LsatSalinas_CDS 8915 GAGATACCATAGTGACTTGGCTTTGGGGTGGTTTCTCCGTGGACAATGCC 8964

LserUS96UC23_CD 8915 GAGATACCATAGTGACTTGGCTTTGGGGTGGTTTCTCCGTGGACAATGCC 8964

LvirLAC006941_C 8930 GAGATACCATAGTGACTTGGCTTTGGGGTGGTTTCTCCGTGGACAATGCC 8979

LserLAC005780_C 8948 GAGATACCATAGTGACTTGGCTTTGGGGTGGTTTCTCCGTGGACAATGCC 8997

LsalLAC008020_C 8948 GAGATACCATAGTGACTTGGCTTTGGGGTGGTTTCTCCGTGGACAATGCC 8997

LsalCGN5271_CDS 8948 GAGATACCATAGTGACTTGGCTTTGGGGTGGTTTCTCCGTGGACAATGCC 8997

LsatLACWENDEL_C 8965 ACCTTAAATCGTTTTTTTAGTCTTCATCATTTACTCCCCTTTCTTTTAGT 9014

LsatSalinas_CDS 8965 ACCTTAAATCGTTTTTTTAGTCTTCATCATTTACTCCCCTTTCTTTTAGT 9014

LserUS96UC23_CD 8965 ACCTTAAATCGTTTTTTTAGTCTTCATCATTTACTCCCCTTTCTTTTAGT 9014

LvirLAC006941_C 8980 ACCTTAAATCGTTTTTTTAGTCTTCATCATTTACTCCCCTTTCTTTTAGT 9029

LserLAC005780_C 8998 ACCTTAAATCGTTTTTTTAGTCTTCATCATTTACTCCCCTTTCTTTTAGT 9047

LsalLAC008020_C 8998 ACCTTAAATCGTTTTTTTAGTCTTCATCATTTACTCCCCTTTCTTTTAGT 9047

LsalCGN5271_CDS 8998 ACCTTAAATCGTTTTTTTAGTCTTCATCATTTACTCCCCTTTCTTTTAGT 9047

LsatLACWENDEL_C 9015 AGGCGCCAGTCTTCTTCATCTGGCCGCATTGCATCAATATGGATCAAATA 9064

LsatSalinas_CDS 9015 AGGCGCCAGTCTTCTTCATCTGGCCGCATTGCATCAATATGGATCAAATA 9064

LserUS96UC23_CD 9015 AGGCGCCAGTCTTCTTCATCTGGCCGCATTGCATCAATATGGATCAAATA 9064

LvirLAC006941_C 9030 AGGCGCCAGTCTTCTTCATCTGGCCGCATTGCATCAATATGGATCAAATA 9079

LserLAC005780_C 9048 AGGCGCCAGTCTTCTTCATCTGGCCGCATTGCATCAATATGGATCAAATA 9097

LsalLAC008020_C 9048 AGGCGCCAGTCTTCTTCATCTGGCCGCATTGCATCAATATGGATCAAATA 9097

LsalCGN5271_CDS 9048 AGGCGCCAGTCTTCTTCATCTGGCCGCATTGCATCAATATGGATCAAATA 9097

LsatLACWENDEL_C 9065 ATCCATTGGGTGTACATTCAGAGATGGATAAAATTGCTTCTTACCCTTAT 9114

LsatSalinas_CDS 9065 ATCCATTGGGTGTACATTCAGAGATGGATAAAATTGCTTCTTACCCTTAT 9114

LserUS96UC23_CD 9065 ATCCATTGGGTGTACATTCAGAGATGGATAAAATTGCTTCTTACCCTTAT 9114

LvirLAC006941_C 9080 ATCCATTGGGTGTACATTCAGAGATGGATAAAATTGCTTCTTACCCTTAT 9129

LserLAC005780_C 9098 ATCCATTGGGTGTACATTCAGAGATGGATAAAATTGCTTCTTACCCTTAT 9147

LsalLAC008020_C 9098 ATCCATTGGGTGTACATTCAGAGATGGATAAAATTGCTTCTTACCCTTAT 9147

LsalCGN5271_CDS 9098 ATCCATTGGGTGTACATTCAGAGATGGATAAAATTGCTTCTTACCCTTAT 9147

LsatLACWENDEL_C 9115 TTTTATGTAAAGGATCTAGTAGGTTGGGTAGCTTTTGCTATCTTTTCTTC 9164

LsatSalinas_CDS 9115 TTTTATGTAAAGGATCTAGTAGGTTGGGTAGCTTTTGCTATCTTTTCTTC 9164

LserUS96UC23_CD 9115 TTTTATGTAAAGGATCTAGTAGGTTGGGTAGCTTTTGCTATCTTTTCTTC 9164

LvirLAC006941_C 9130 TTTTATGTAAAGGATCTAGTAGGTTGGGTAGCTTTTGCTATCTTTTCTTC 9179

LserLAC005780_C 9148 TTTTATGTAAAGGATCTAGTAGGTTGGGTAGCTTTTGCTATCTTTTCTTC 9197

LsalLAC008020_C 9148 TTTTATGTAAAGGATCTAGTAGGTTGGGTAGCTTTTGCTATCTTTTCTTC 9197

LsalCGN5271_CDS 9148 TTTTATGTAAAGGATCTAGTAGGTTGGGTAGCTTTTGCTATCTTTTCTTC 9197

LsatLACWENDEL_C 9165 CATTTTTATTTTTTATGCTCCTAATGTTTTGGGGCATCCCGACAATTATA 9214

LsatSalinas_CDS 9165 CATTTTTATTTTTTATGCTCCTAATGTTTTGGGGCATCCCGACAATTATA 9214

LserUS96UC23_CD 9165 CATTTTTATTTTTTATGCTCCTAATGTTTTGGGGCATCCCGACAATTATA 9214

LvirLAC006941_C 9180 CATTTTTATTTTTTATGCTCCTAATGTTTTGGGGCATCCCGACAATTATA 9229

LserLAC005780_C 9198 CATTTTTATTTTTTATGCTCCTAATGTTTTGGGGCATCCCGACAATTATA 9247

LsalLAC008020_C 9198 CATTTTTATTTTTTATGCTCCTAATGTTTTGGGGCATCCCGACAATTATA 9247

LsalCGN5271_CDS 9198 CATTTTTATTTTTTATGCTCCTAATGTTTTGGGGCATCCCGACAATTATA 9247

LsatLACWENDEL_C 9215 TACCTGCTAATCCGATGCCCACCCCGCCTCATATTGTACCGGAATGGTAT 9264

LsatSalinas_CDS 9215 TACCTGCTAATCCGATGCCCACCCCGCCTCATATTGTACCGGAATGGTAT 9264

LserUS96UC23_CD 9215 TACCTGCTAATCCGATGCCCACCCCGCCTCATATTGTACCGGAATGGTAT 9264

LvirLAC006941_C 9230 TACCTGCTAATCCGATGCCCACCCCGCCTCATATTGTACCGGAATGGTAT 9279

LserLAC005780_C 9248 TACCTGCTAATCCGATGCCCACCCCGCCTCATATTGTACCGGAATGGTAT 9297

LsalLAC008020_C 9248 TACCTGCTAATCCGATGCCCACCCCGCCTCATATTGTACCGGAATGGTAT 9297

LsalCGN5271_CDS 9248 TACCTGCTAATCCGATGCCCACCCCGCCTCATATTGTACCGGAATGGTAT 9297

LsatLACWENDEL_C 9265 TTCCTACCGATCCATGCCATTCTTCGTAGTATACCTGACAAAGCGGGAGG 9314

LsatSalinas_CDS 9265 TTCCTACCGATCCATGCCATTCTTCGTAGTATACCTGACAAAGCGGGAGG 9314

LserUS96UC23_CD 9265 TTCCTACCGATCCATGCCATTCTTCGTAGTATACCTGACAAAGCGGGAGG 9314

LvirLAC006941_C 9280 TTCCTACCGATCCATGCCATTCTTCGTAGTATACCTGACAAAGCGGGAGG 9329

LserLAC005780_C 9298 TTCCTACCGATCCATGCCATTCTTCGTAGTATACCTGACAAAGCGGGAGG 9347

LsalLAC008020_C 9298 TTCCTACCGATCCATGCCATTCTTCGTAGTATACCTGACAAAGCGGGAGG 9347

LsalCGN5271_CDS 9298 TTCCTACCGATCCATGCCATTCTTCGTAGTATACCTGACAAAGCGGGAGG 9347

LsatLACWENDEL_C 9315 TGTAGCCGCAATAGCACCAGTTTTTATATGTCTGTTGGCTTTACCTTTTT 9364

LsatSalinas_CDS 9315 TGTAGCCGCAATAGCACCAGTTTTTATATGTCTGTTGGCTTTACCTTTTT 9364

LserUS96UC23_CD 9315 TGTAGCCGCAATAGCACCAGTTTTTATATGTCTGTTGGCTTTACCTTTTT 9364

LvirLAC006941_C 9330 TGTAGCCGCAATAGCACCAGTTTTTATATGTCTGTTGGCTTTACCTTTTT 9379

LserLAC005780_C 9348 TGTAGCCGCAATAGCACCAGTTTTTATATGTCTGTTGGCTTTACCTTTTT 9397

LsalLAC008020_C 9348 TGTAGCCGCAATAGCACCAGTTTTTATATGTCTGTTGGCTTTACCTTTTT 9397

LsalCGN5271_CDS 9348 TGTAGCCGCAATAGCACCAGTTTTTATATGTCTGTTGGCTTTACCTTTTT 9397

LsatLACWENDEL_C 9365 TTAAAAGTATGTATGTACGTAGTTCAAGTTTTCGCCCGATTCACCAAGGA 9414

LsatSalinas_CDS 9365 TTAAAAGTATGTATGTACGTAGTTCAAGTTTTCGCCCGATTCACCAAGGA 9414

LserUS96UC23_CD 9365 TTAAAAGTATGTATGTACGTAGTTCAAGTTTTCGCCCGATTCACCAAGGA 9414

LvirLAC006941_C 9380 TTAAAAGTATGTATGTACGTAGTTCAAGTTTTCGCCCGATTCACCAAGGA 9429

LserLAC005780_C 9398 TTAAAAGTATGTATGTACGTAGTTCAAGTTTTCGCCCGATTCACCAAGGA 9447

LsalLAC008020_C 9398 TTAAAAGTATGTATGTACGTAGTTCAAGTTTTCGCCCGATTCACCAAGGA 9447

LsalCGN5271_CDS 9398 TTAAAAGTATGTATGTACGTAGTTCAAGTTTTCGCCCGATTCACCAAGGA 9447

LsatLACWENDEL_C 9415 ATATTTTGGTTGCTTTTGGCGGATCGCTTACTACTAGGTTGGATCGGATG 9464

LsatSalinas_CDS 9415 ATATTTTGGTTGCTTTTGGCGGATCGCTTACTACTAGGTTGGATCGGATG 9464

LserUS96UC23_CD 9415 ATATTTTGGTTGCTTTTGGCGGATCGCTTACTACTAGGTTGGATCGGATG 9464

LvirLAC006941_C 9430 ATATTTTGGTTGCTTTTGGCGGATCGCTTACTACTAGGTTGGATCGGATG 9479

LserLAC005780_C 9448 ATATTTTGGTTGCTTTTGGCGGATCGCTTACTACTAGGTTGGATCGGATG 9497

LsalLAC008020_C 9448 ATATTTTGGTTGCTTTTGGCGGATCGCTTACTACTAGGTTGGATCGGATG 9497

LsalCGN5271_CDS 9448 ATATTTTGGTTGCTTTTGGCGGATCGCTTACTACTAGGTTGGATCGGATG 9497

LsatLACWENDEL_C 9465 TCAACCTGTGGAGGCACCATTTGTGACTATTGGACAAATTTCCCCTTTTG 9514

LsatSalinas_CDS 9465 TCAACCTGTGGAGGCACCATTTGTGACTATTGGACAAATTTCCCCTTTTG 9514

LserUS96UC23_CD 9465 TCAACCTGTGGAGGCACCATTTGTGACTATTGGACAAATTTCCCCTTTTG 9514

LvirLAC006941_C 9480 TCAACCTGTGGAGGCACCATTTGTGACTATTGGACAAATTTCCCCTTTTG 9529

LserLAC005780_C 9498 TCAACCTGTGGAGGCACCATTTGTGACTATTGGACAAATTTCCCCTTTTG 9547

LsalLAC008020_C 9498 TCAACCTGTGGAGGCACCATTTGTGACTATTGGACAAATTTCCCCTTTTG 9547

LsalCGN5271_CDS 9498 TCAACCTGTGGAGGCACCATTTGTGACTATTGGACAAATTTCCCCTTTTG 9547

LsatLACWENDEL_C 9515 TTTTCTTCTTGTTCTTTGCCATAACGCCCATTCTGGGACGAGTTGGAAGA 9564

LsatSalinas_CDS 9515 TTTTCTTCTTGTTCTTTGCCATAACGCCCATTCTGGGACGAGTTGGAAGA 9564

LserUS96UC23_CD 9515 TTTTCTTCTTGTTCTTTGCCATAACGCCCATTCTGGGACGAGTTGGAAGA 9564

LvirLAC006941_C 9530 TTTTCTTCTTGTTCTTTGCCATAACGCCCATTCTGGGACGAGTTGGAAGA 9579

LserLAC005780_C 9548 TTTTCTTCTTGTTCTTTGCCATAACGCCCATTCTGGGACGAGTTGGAAGA 9597

LsalLAC008020_C 9548 TTTTCTTCTTGTTCTTTGCCATAACGCCCATTCTGGGACGAGTTGGAAGA 9597

LsalCGN5271_CDS 9548 TTTTCTTCTTGTTCTTTGCCATAACGCCCATTCTGGGACGAGTTGGAAGA 9597

LsatLACWENDEL_C 9565 GGAATTCCTAATTCTTACACGGATGAGACTGCCTGAACGACAAATCCGGT 9614

LsatSalinas_CDS 9565 GGAATTCCTAATTCTTACACGGATGAGACTGCCTGAACGACAAATCCGGT 9614

LserUS96UC23_CD 9565 GGAATTCCTAATTCTTACACGGATGAGACTGCCTGAACGACAAATCCGGT 9614

LvirLAC006941_C 9580 GGAATTCCTAATTCTTACACGGATGAGACTGCCTGAACGACAAATCCGGT 9629

LserLAC005780_C 9598 GGAATTCCTAATTCTTACACGGATGAGACTGCCTGAACGACAAATCCGGT 9647

LsalLAC008020_C 9598 GGAATTCCTAATTCTTACACGGATGAGACTGCCTGAACGACAAATCCGGT 9647

LsalCGN5271_CDS 9598 GGAATTCCTAATTCTTACACGGATGAGACTGCCTGAACGACAAATCCGGT 9647

LsatLACWENDEL_C 9615 CCGATGGCTGTTCTCCACTAACCACAAGGATATAGGGACTCTATATTTCA 9664

LsatSalinas_CDS 9615 CCGATGGCTGTTCTCCACTAACCACAAGGATATAGGGACTCTATATTTCA 9664

LserUS96UC23_CD 9615 CCGATGGCTGTTCTCCACTAACCACAAGGATATAGGGACTCTATATTTCA 9664

LvirLAC006941_C 9630 CCGATGGCTGTTCTCCACTAACCACAAGGATATAGGGACTCTATATTTCA 9679

LserLAC005780_C 9648 CCGATGGCTGTTCTCCACTAACCACAAGGATATAGGGACTCTATATTTCA 9697

LsalLAC008020_C 9648 CCGATGGCTGTTCTCCACTAACCACAAGGATATAGGGACTCTATATTTCA 9697

LsalCGN5271_CDS 9648 CCGATGGCTGTTCTCCACTAACCACAAGGATATAGGGACTCTATATTTCA 9697

LsatLACWENDEL_C 9665 TCTTTGGTGCCATTGCTGGAGTGATGGGCACATGCTTCTCAGTACTGATT 9714

LsatSalinas_CDS 9665 TCTTTGGTGCCATTGCTGGAGTGATGGGCACATGCTTCTCAGTACTGATT 9714

LserUS96UC23_CD 9665 TCTTTGGTGCCATTGCTGGAGTGATGGGCACATGCTTCTCAGTACTGATT 9714

LvirLAC006941_C 9680 TCTTTGGTGCCATTGCTGGAGTGATGGGCACATGCTTCTCAGTACTGATT 9729

LserLAC005780_C 9698 TCTTTGGTGCCATTGCTGGAGTGATGGGCACATGCTTCTCAGTACTGATT 9747

LsalLAC008020_C 9698 TCTTTGGTGCCATTGCTGGAGTGATGGGCACATGCTTCTCAGTACTGATT 9747

LsalCGN5271_CDS 9698 TCTTTGGTGCCATTGCTGGAGTGATGGGCACATGCTTCTCAGTACTGATT 9747

LsatLACWENDEL_C 9715 CGTATGGAATTAGCACGACCCGGCGATCAAATTCTTGGTGGGAATCATCA 9764

LsatSalinas_CDS 9715 CGTATGGAATTAGCACGACCCGGCGATCAAATTCTTGGTGGGAATCATCA 9764

LserUS96UC23_CD 9715 CGTATGGAATTAGCACGACCCGGCGATCAAATTCTTGGTGGGAATCATCA 9764

LvirLAC006941_C 9730 CGTATGGAATTAGCACGACCCGGCGATCAAATTCTTGGTGGGAATCATCA 9779

LserLAC005780_C 9748 CGTATGGAATTAGCACGACCCGGCGATCAAATTCTTGGTGGGAATCATCA 9797

LsalLAC008020_C 9748 CGTATGGAATTAGCACGACCCGGCGATCAAATTCTTGGTGGGAATCATCA 9797

LsalCGN5271_CDS 9748 CGTATGGAATTAGCACGACCCGGCGATCAAATTCTTGGTGGGAATCATCA 9797

LsatLACWENDEL_C 9765 ACTTTATAATGTTTTAATAACGGCTCACGCTTTTTTAATGATATTTTTTA 9814

LsatSalinas_CDS 9765 ACTTTATAATGTTTTAATAACGGCTCACGCTTTTTTAATGATATTTTTTA 9814

LserUS96UC23_CD 9765 ACTTTATAATGTTTTAATAACGGCTCACGCTTTTTTAATGATATTTTTTA 9814

LvirLAC006941_C 9780 ACTTTATAATGTTTTAATAACGGCTCACGCTTTTTTAATGATATTTTTTA 9829

LserLAC005780_C 9798 ACTTTATAATGTTTTAATAACGGCTCACGCTTTTTTAATGATATTTTTTA 9847

LsalLAC008020_C 9798 ACTTTATAATGTTTTAATAACGGCTCACGCTTTTTTAATGATATTTTTTA 9847

LsalCGN5271_CDS 9798 ACTTTATAATGTTTTAATAACGGCTCACGCTTTTTTAATGATATTTTTTA 9847

LsatLACWENDEL_C 9815 TGGTTATGCCGGCGATGATAGGTGGATCTGGTAATTGGTCTGTTCCGATT 9864

LsatSalinas_CDS 9815 TGGTTATGCCGGCGATGATAGGTGGATCTGGTAATTGGTCTGTTCCGATT 9864

LserUS96UC23_CD 9815 TGGTTATGCCGGCGATGATAGGTGGATCTGGTAATTGGTCTGTTCCGATT 9864

LvirLAC006941_C 9830 TGGTTATGCCGGCGATGATAGGTGGATCTGGTAATTGGTCTGTTCCGATT 9879

LserLAC005780_C 9848 TGGTTATGCCGGCGATGATAGGTGGATCTGGTAATTGGTCTGTTCCGATT 9897

LsalLAC008020_C 9848 TGGTTATGCCGGCGATGATAGGTGGATCTGGTAATTGGTCTGTTCCGATT 9897

LsalCGN5271_CDS 9848 TGGTTATGCCGGCGATGATAGGTGGATCTGGTAATTGGTCTGTTCCGATT 9897

LsatLACWENDEL_C 9865 CTGATAGGTGCGCCTGACATGGCATTTCCACGATTAAATAATATTTCATT 9914

LsatSalinas_CDS 9865 CTGATAGGTGCGCCTGACATGGCATTTCCACGATTAAATAATATTTCATT 9914

LserUS96UC23_CD 9865 CTGATAGGTGCGCCTGACATGGCATTTCCACGATTAAATAATATTTCATT 9914

LvirLAC006941_C 9880 CTGATAGGTGCGCCTGACATGGCATTTCCACGATTAAATAATATTTCATT 9929

LserLAC005780_C 9898 CTGATAGGTGCGCCTGACATGGCATTTCCACGATTAAATAATATTTCATT 9947

LsalLAC008020_C 9898 CTGATAGGTGCGCCTGACATGGCATTTCCACGATTAAATAATATTTCATT 9947

LsalCGN5271_CDS 9898 CTGATAGGTGCGCCTGACATGGCATTTCCACGATTAAATAATATTTCATT 9947

LsatLACWENDEL_C 9915 CTGGTTGTTGCCACCAAGTCTCTTGCTCCTATTAAGCCCAGCCTTAGTAG 9964

LsatSalinas_CDS 9915 CTGGTTGTTGCCACCAAGTCTCTTGCTCCTATTAAGCCCAGCCTTAGTAG 9964

LserUS96UC23_CD 9915 CTGGTTGTTGCCACCAAGTCTCTTGCTCCTATTAAGCCCAGCCTTAGTAG 9964

LvirLAC006941_C 9930 CTGGTTGTTGCCACCAAGTCTCTTGCTCCTATTAAGCCCAGCCTTAGTAG 9979

LserLAC005780_C 9948 CTGGTTGTTGCCACCAAGTCTCTTGCTCCTATTAAGCCCAGCCTTAGTAG 9997

LsalLAC008020_C 9948 CTGGTTGTTGCCACCAAGTCTCTTGCTCCTATTAAGCCCAGCCTTAGTAG 9997

LsalCGN5271_CDS 9948 CTGGTTGTTGCCACCAAGTCTCTTGCTCCTATTAAGCCCAGCCTTAGTAG 9997

LsatLACWENDEL_C 9965 AAGTGGGTAGTGGCACCGGGTGGACGGTCTATCCGCCCTTAAGTGGTATT 10014

LsatSalinas_CDS 9965 AAGTGGGTAGTGGCACCGGGTGGACGGTCTATCCGCCCTTAAGTGGTATT 10014

LserUS96UC23_CD 9965 AAGTGGGTAGTGGCACCGGGTGGACGGTCTATCCGCCCTTAAGTGGTATT 10014

LvirLAC006941_C 9980 AAGTGGGTAGTGGCACCGGGTGGACGGTCTATCCGCCCTTAAGTGGTATT 10029

LserLAC005780_C 9998 AAGTGGGTAGTGGCACCGGGTGGACGGTCTATCCGCCCTTAAGTGGTATT 10047

LsalLAC008020_C 9998 AAGTGGGTAGTGGCACCGGGTGGACGGTCTATCCGCCCTTAAGTGGTATT 10047

LsalCGN5271_CDS 9998 AAGTGGGTAGTGGCACCGGGTGGACGGTCTATCCGCCCTTAAGTGGTATT 10047

LsatLACWENDEL_C 10015 ACCAGCCATTCTGGAGGAGCAGTTGATTCAGCAATTTCTAGTCCTCATCT 10064

LsatSalinas_CDS 10015 ACCAGCCATTCTGGAGGAGCAGTTGATTCAGCAATTTCTAGTCCTCATCT 10064

LserUS96UC23_CD 10015 ACCAGCCATTCTGGAGGAGCAGTTGATTCAGCAATTTCTAGTCCTCATCT 10064

LvirLAC006941_C 10030 ACCAGCCATTCTGGAGGAGCAGTTGATTCAGCAATTTCTAGTCCTCATCT 10079

LserLAC005780_C 10048 ACCAGCCATTCTGGAGGAGCAGTTGATTCAGCAATTTCTAGTCCTCATCT 10097

LsalLAC008020_C 10048 ACCAGCCATTCTGGAGGAGCAGTTGATTCAGCAATTTCTAGTCCTCATCT 10097

LsalCGN5271_CDS 10048 ACCAGCCATTCTGGAGGAGCAGTTGATTCAGCAATTTCTAGTCCTCATCT 10097

LsatLACWENDEL_C 10065 ATCTGGTATTTCATCCATTTTAGGTTCTATCAATTTTATAACAACTATCT 10114

LsatSalinas_CDS 10065 ATCTGGTATTTCATCCATTTTAGGTTCTATCAATTTTATAACAACTATCT 10114

LserUS96UC23_CD 10065 ATCTGGTATTTCATCCATTTTAGGTTCTATCAATTTTATAACAACTATCT 10114

LvirLAC006941_C 10080 ATCTGGTATTTCATCCATTTTAGGTTCTATCAATTTTATAACAACTATCT 10129

LserLAC005780_C 10098 ATCTGGTATTTCATCCATTTTAGGTTCTATCAATTTTATAACAACTATCT 10147

LsalLAC008020_C 10098 ATCTGGTATTTCATCCATTTTAGGTTCTATCAATTTTATAACAACTATCT 10147

LsalCGN5271_CDS 10098 ATCTGGTATTTCATCCATTTTAGGTTCTATCAATTTTATAACAACTATCT 10147

LsatLACWENDEL_C 10115 CCAACATGCGTGGACCTGGAATGACTATGCATAGATCACCCCTATTTGTG 10164

LsatSalinas_CDS 10115 CCAACATGCGTGGACCTGGAATGACTATGCATAGATCACCCCTATTTGTG 10164

LserUS96UC23_CD 10115 CCAACATGCGTGGACCTGGAATGACTATGCATAGATCACCCCTATTTGTG 10164

LvirLAC006941_C 10130 CCAACATGCGTGGACCTGGAATGACTATGCATAGATCACCCCTATTTGTG 10179

LserLAC005780_C 10148 CCAACATGCGTGGACCTGGAATGACTATGCATAGATCACCCCTATTTGTG 10197

LsalLAC008020_C 10148 CCAACATGCGTGGACCTGGAATGACTATGCATAGATCACCCCTATTTGTG 10197

LsalCGN5271_CDS 10148 CCAACATGCGTGGACCTGGAATGACTATGCATAGATCACCCCTATTTGTG 10197

LsatLACWENDEL_C 10165 TGGTCCGTTCTAGTGACAGCATTCCCACTTTTATTATCACTTCCGGTACT 10214

LsatSalinas_CDS 10165 TGGTCCGTTCTAGTGACAGCATTCCCACTTTTATTATCACTTCCGGTACT 10214

LserUS96UC23_CD 10165 TGGTCCGTTCTAGTGACAGCATTCCCACTTTTATTATCACTTCCGGTACT 10214

LvirLAC006941_C 10180 TGGTCCGTTCTAGTGACAGCATTCCCACTTTTATTATCACTTCCGGTACT 10229

LserLAC005780_C 10198 TGGTCCGTTCTAGTGACAGCATTCCCACTTTTATTATCACTTCCGGTACT 10247

LsalLAC008020_C 10198 TGGTCCGTTCTAGTGACAGCATTCCCACTTTTATTATCACTTCCGGTACT 10247

LsalCGN5271_CDS 10198 TGGTCCGTTCTAGTGACAGCATTCCCACTTTTATTATCACTTCCGGTACT 10247

LsatLACWENDEL_C 10215 GGCAGGGGCAATTACCATGTTATTAACCGATCGAAACTTTAATACAACCT 10264

LsatSalinas_CDS 10215 GGCAGGGGCAATTACCATGTTATTAACCGATCGAAACTTTAATACAACCT 10264

LserUS96UC23_CD 10215 GGCAGGGGCAATTACCATGTTATTAACCGATCGAAACTTTAATACAACCT 10264

LvirLAC006941_C 10230 GGCAGGGGCAATTACCATGTTATTAACCGATCGAAACTTTAATACAACCT 10279

LserLAC005780_C 10248 GGCAGGGGCAATTACCATGTTATTAACCGATCGAAACTTTAATACAACCT 10297

LsalLAC008020_C 10248 GGCAGGGGCAATTACCATGTTATTAACCGATCGAAACTTTAATACAACCT 10297

LsalCGN5271_CDS 10248 GGCAGGGGCAATTACCATGTTATTAACCGATCGAAACTTTAATACAACCT 10297

LsatLACWENDEL_C 10265 TTTCTGATCCCGCTGGAGGGGGAGACCCCATATTATACCAGCATCTCTTT 10314

LsatSalinas_CDS 10265 TTTCTGATCCCGCTGGAGGGGGAGACCCCATATTATACCAGCATCTCTTT 10314

LserUS96UC23_CD 10265 TTTCTGATCCCGCTGGAGGGGGAGACCCCATATTATACCAGCATCTCTTT 10314

LvirLAC006941_C 10280 TTTCTGATCCCGCTGGAGGGGGAGACCCCATATTATACCAGCATCTCTTT 10329

LserLAC005780_C 10298 TTTCTGATCCCGCTGGAGGGGGAGACCCCATATTATACCAGCATCTCTTT 10347

LsalLAC008020_C 10298 TTTCTGATCCCGCTGGAGGGGGAGACCCCATATTATACCAGCATCTCTTT 10347

LsalCGN5271_CDS 10298 TTTCTGATCCCGCTGGAGGGGGAGACCCCATATTATACCAGCATCTCTTT 10347

LsatLACWENDEL_C 10315 CGGTTCTTCGGTCATCCAGAGGTGTATATTCCCATTCTGCCTGGATCCGG 10364

LsatSalinas_CDS 10315 CGGTTCTTCGGTCATCCAGAGGTGTATATTCCCATTCTGCCTGGATCCGG 10364

LserUS96UC23_CD 10315 CGGTTCTTCGGTCATCCAGAGGTGTATATTCCCATTCTGCCTGGATCCGG 10364

LvirLAC006941_C 10330 CGGTTCTTCGGTCATCCAGAGGTGTATATTCCCATTCTGCCTGGATCCGG 10379

LserLAC005780_C 10348 CGGTTCTTCGGTCATCCAGAGGTGTATATTCCCATTCTGCCTGGATCCGG 10397

LsalLAC008020_C 10348 CGGTTCTTCGGTCATCCAGAGGTGTATATTCCCATTCTGCCTGGATCCGG 10397

LsalCGN5271_CDS 10348 CGGTTCTTCGGTCATCCAGAGGTGTATATTCCCATTCTGCCTGGATCCGG 10397

LsatLACWENDEL_C 10365 TATCATAAGTCATATCGTTTCTACTTTTTCGGGAAAACCGGTCTTCGGGT 10414

LsatSalinas_CDS 10365 TATCATAAGTCATATCGTTTCTACTTTTTCGGGAAAACCGGTCTTCGGGT 10414

LserUS96UC23_CD 10365 TATCATAAGTCATATCGTTTCTACTTTTTCGGGAAAACCGGTCTTCGGGT 10414

LvirLAC006941_C 10380 TATCATAAGTCATATCGTTTCTACTTTTTCGGGAAAACCGGTCTTCGGGT 10429

LserLAC005780_C 10398 TATCATAAGTCATATCGTTTCTACTTTTTCGGGAAAACCGGTCTTCGGGT 10447

LsalLAC008020_C 10398 TATCATAAGTCATATCGTTTCTACTTTTTCGGGAAAACCGGTCTTCGGGT 10447

LsalCGN5271_CDS 10398 TATCATAAGTCATATCGTTTCTACTTTTTCGGGAAAACCGGTCTTCGGGT 10447

LsatLACWENDEL_C 10415 ATCTAGGCATGGTTTATGCCATGATCAGTATAGGTGTTCCTGGATCTCTT 10464

LsatSalinas_CDS 10415 ATCTAGGCATGGTTTATGCCATGATCAGTATAGGTGTTCCTGGATCTCTT 10464

LserUS96UC23_CD 10415 ATCTAGGCATGGTTTATGCCATGATCAGTATAGGTGTTCCTGGATCTCTT 10464

LvirLAC006941_C 10430 ATCTAGGCATGGTTTATGCCATGATCAGTATAGGTGTTCCTGGATCTCTT 10479

LserLAC005780_C 10448 ATCTAGGCATGGTTTATGCCATGATCAGTATAGGTGTTCCTGGATCTCTT 10497

LsalLAC008020_C 10448 ATCTAGGCATGGTTTATGCCATGATCAGTATAGGTGTTCCTGGATCTCTT 10497

LsalCGN5271_CDS 10448 ATCTAGGCATGGTTTATGCCATGATCAGTATAGGTGTTCCTGGATCTCTT 10497

LsatLACWENDEL_C 10465 GTTTGGGCTCATCATATGTTTACTGTGGGCTTAGACGTTGATACCCGTGC 10514

LsatSalinas_CDS 10465 GTTTGGGCTCATCATATGTTTACTGTGGGCTTAGACGTTGATACCCGTGC 10514

LserUS96UC23_CD 10465 GTTTGGGCTCATCATATGTTTACTGTGGGCTTAGACGTTGATACCCGTGC 10514

LvirLAC006941_C 10480 GTTTGGGCTCATCATATGTTTACTGTGGGCTTAGACGTTGATACCCGTGC 10529

LserLAC005780_C 10498 GTTTGGGCTCATCATATGTTTACTGTGGGCTTAGACGTTGATACCCGTGC 10547

LsalLAC008020_C 10498 GTTTGGGCTCATCATATGTTTACTGTGGGCTTAGACGTTGATACCCGTGC 10547

LsalCGN5271_CDS 10498 GTTTGGGCTCATCATATGTTTACTGTGGGCTTAGACGTTGATACCCGTGC 10547

LsatLACWENDEL_C 10515 CTACTTCACCGCAGCTACCATGATCATAGCTGTCCCCACTGGAATCAAAA 10564

LsatSalinas_CDS 10515 CTACTTCACCGCAGCTACCATGATCATAGCTGTCCCCACTGGAATCAAAA 10564

LserUS96UC23_CD 10515 CTACTTCACCGCAGCTACCATGATCATAGCTGTCCCCACTGGAATCAAAA 10564

LvirLAC006941_C 10530 CTACTTCACCGCAGCTACCATGATCATAGCTGTCCCCACTGGAATCAAAA 10579

LserLAC005780_C 10548 CTACTTCACCGCAGCTACCATGATCATAGCTGTCCCCACTGGAATCAAAA 10597

LsalLAC008020_C 10548 CTACTTCACCGCAGCTACCATGATCATAGCTGTCCCCACTGGAATCAAAA 10597

LsalCGN5271_CDS 10548 CTACTTCACCGCAGCTACCATGATCATAGCTGTCCCCACTGGAATCAAAA 10597

LsatLACWENDEL_C 10565 TCTTTAGTTGGATCGCTACCATGTGGGGGGGTTCGATACAATACAAAACA 10614

LsatSalinas_CDS 10565 TCTTTAGTTGGATCGCTACCATGTGGGGGGGTTCGATACAATACAAAACA 10614

LserUS96UC23_CD 10565 TCTTTAGTTGGATCGCTACCATGTGGGGGGGTTCGATACAATACAAAACA 10614

LvirLAC006941_C 10580 TCTTTAGTTGGATCGCTACCATGTGGGGGGGTTCGATACAATACAAAACA 10629

LserLAC005780_C 10598 TCTTTAGTTGGATCGCTACCATGTGGGGGGGTTCGATACAATACAAAACA 10647

LsalLAC008020_C 10598 TCTTTAGTTGGATCGCTACCATGTGGGGGGGTTCGATACAATACAAAACA 10647

LsalCGN5271_CDS 10598 TCTTTAGTTGGATCGCTACCATGTGGGGGGGTTCGATACAATACAAAACA 10647

LsatLACWENDEL_C 10615 CCCATGTTATTTGCTGTAGGGTCCATATTTTTGTTCACCATAGGGGGACT 10664

LsatSalinas_CDS 10615 CCCATGTTATTTGCTGTAGGGTCCATATTTTTGTTCACCATAGGGGGACT 10664

LserUS96UC23_CD 10615 CCCATGTTATTTGCTGTAGGGTCCATATTTTTGTTCACCATAGGGGGACT 10664

LvirLAC006941_C 10630 CCCATGTTATTTGCTGTAGGGTCCATATTTTTGTTCACCATAGGGGGACT 10679

LserLAC005780_C 10648 CCCATGTTATTTGCTGTAGGGTCCATATTTTTGTTCACCATAGGGGGACT 10697

LsalLAC008020_C 10648 CCCATGTTATTTGCTGTAGGGTCCATATTTTTGTTCACCATAGGGGGACT 10697

LsalCGN5271_CDS 10648 CCCATGTTATTTGCTGTAGGGTCCATATTTTTGTTCACCATAGGGGGACT 10697

LsatLACWENDEL_C 10665 CACTGGAATCGTTCCGGCAAATTCTGGGCTAGACATTGCTCTACATGATA 10714

LsatSalinas_CDS 10665 CACTGGAATCGTTCCGGCAAATTCTGGGCTAGACATTGCTCTACATGATA 10714

LserUS96UC23_CD 10665 CACTGGAATCGTTCCGGCAAATTCTGGGCTAGACATTGCTCTACATGATA 10714

LvirLAC006941_C 10680 CACTGGAATCGTTCCGGCAAATTCTGGGCTAGACATTGCTCTACATGATA 10729

LserLAC005780_C 10698 CACTGGAATCGTTCCGGCAAATTCTGGGCTAGACATTGCTCTACATGATA 10747

LsalLAC008020_C 10698 CACTGGAATCGTTCCGGCAAATTCTGGGCTAGACATTGCTCTACATGATA 10747

LsalCGN5271_CDS 10698 CACTGGAATCGTTCCGGCAAATTCTGGGCTAGACATTGCTCTACATGATA 10747

LsatLACWENDEL_C 10715 CTTATTATGTGGTTGCACATTTCCATTATGTACTTTCTATGGGAGCCGTT 10764

LsatSalinas_CDS 10715 CTTATTATGTGGTTGCACATTTCCATTATGTACTTTCTATGGGAGCCGTT 10764

LserUS96UC23_CD 10715 CTTATTATGTGGTTGCACATTTCCATTATGTACTTTCTATGGGAGCCGTT 10764

LvirLAC006941_C 10730 CTTATTATGTGGTTGCACATTTCCATTATGTACTTTCTATGGGAGCCGTT 10779

LserLAC005780_C 10748 CTTATTATGTGGTTGCACATTTCCATTATGTACTTTCTATGGGAGCCGTT 10797

LsalLAC008020_C 10748 CTTATTATGTGGTTGCACATTTCCATTATGTACTTTCTATGGGAGCCGTT 10797

LsalCGN5271_CDS 10748 CTTATTATGTGGTTGCACATTTCCATTATGTACTTTCTATGGGAGCCGTT 10797

LsatLACWENDEL_C 10765 TTTGCTTTATTTGCAGGATTTCACTATTGGGTAGGTAAAATCTTTGGTCG 10814

LsatSalinas_CDS 10765 TTTGCTTTATTTGCAGGATTTCACTATTGGGTAGGTAAAATCTTTGGTCG 10814

LserUS96UC23_CD 10765 TTTGCTTTATTTGCAGGATTTCACTATTGGGTAGGTAAAATCTTTGGTCG 10814

LvirLAC006941_C 10780 TTTGCTTTATTTGCAGGATTTCACTATTGGGTAGGTAAAATCTTTGGTCG 10829

LserLAC005780_C 10798 TTTGCTTTATTTGCAGGATTTCACTATTGGGTAGGTAAAATCTTTGGTCG 10847

LsalLAC008020_C 10798 TTTGCTTTATTTGCAGGATTTCACTATTGGGTAGGTAAAATCTTTGGTCG 10847

LsalCGN5271_CDS 10798 TTTGCTTTATTTGCAGGATTTCACTATTGGGTAGGTAAAATCTTTGGTCG 10847

LsatLACWENDEL_C 10815 AACATATCCTGAAACTTTAGGTCAAATCCATTTTTGGATCACTTTTTTCG 10864

LsatSalinas_CDS 10815 AACATATCCTGAAACTTTAGGTCAAATCCATTTTTGGATCACTTTTTTCG 10864

LserUS96UC23_CD 10815 AACATATCCTGAAACTTTAGGTCAAATCCATTTTTGGATCACTTTTTTCG 10864

LvirLAC006941_C 10830 AACATATCCTGAAACTTTAGGTCAAATCCATTTTTGGATCACTTTTTTCG 10879

LserLAC005780_C 10848 AACATATCCTGAAACTTTAGGTCAAATCCATTTTTGGATCACTTTTTTCG 10897

LsalLAC008020_C 10848 AACATATCCTGAAACTTTAGGTCAAATCCATTTTTGGATCACTTTTTTCG 10897

LsalCGN5271_CDS 10848 AACATATCCTGAAACTTTAGGTCAAATCCATTTTTGGATCACTTTTTTCG 10897

LsatLACWENDEL_C 10865 GGGTTAATCCGACCTTCTTTCCCATGCATTTCTTAGGGCTTTCGGGTATG 10914

LsatSalinas_CDS 10865 GGGTTAATCCGACCTTCTTTCCCATGCATTTCTTAGGGCTTTCGGGTATG 10914

LserUS96UC23_CD 10865 GGGTTAATCCGACCTTCTTTCCCATGCATTTCTTAGGGCTTTCGGGTATG 10914

LvirLAC006941_C 10880 GGGTTAATCCGACCTTCTTTCCCATGCATTTCTTAGGGCTTTCGGGTATG 10929

LserLAC005780_C 10898 GGGTTAATCCGACCTTCTTTCCCATGCATTTCTTAGGGCTTTCGGGTATG 10947

LsalLAC008020_C 10898 GGGTTAATCCGACCTTCTTTCCCATGCATTTCTTAGGGCTTTCGGGTATG 10947

LsalCGN5271_CDS 10898 GGGTTAATCCGACCTTCTTTCCCATGCATTTCTTAGGGCTTTCGGGTATG 10947

LsatLACWENDEL_C 10915 CCACGTCGCATTCCAGATTATCCAGATGCTTACGCTGGATGGAATGCCCT 10964

LsatSalinas_CDS 10915 CCACGTCGCATTCCAGATTATCCAGATGCTTACGCTGGATGGAATGCCCT 10964

LserUS96UC23_CD 10915 CCACGTCGCATTCCAGATTATCCAGATGCTTACGCTGGATGGAATGCCCT 10964

LvirLAC006941_C 10930 CCACGTCGCATTCCAGATTATCCAGATGCTTACGCTGGATGGAATGCCCT 10979

LserLAC005780_C 10948 CCACGTCGCATTCCAGATTATCCAGATGCTTACGCTGGATGGAATGCCCT 10997

LsalLAC008020_C 10948 CCACGTCGCATTCCAGATTATCCAGATGCTTACGCTGGATGGAATGCCCT 10997

LsalCGN5271_CDS 10948 CCACGTCGCATTCCAGATTATCCAGATGCTTACGCTGGATGGAATGCCCT 10997

LsatLACWENDEL_C 10965 TAGCAGTTCTGGCTCTTATATATCTGTAGTTGGGATTTGTCGTTTCTTCG 11014

LsatSalinas_CDS 10965 TAGCAGTTCTGGCTCTTATATATCTGTAGTTGGGATTTGTCGTTTCTTCG 11014

LserUS96UC23_CD 10965 TAGCAGTTCTGGCTCTTATATATCTGTAGTTGGGATTTGTCGTTTCTTCG 11014

LvirLAC006941_C 10980 TAGCAGTTCTGGCTCTTATATATCTGTAGTTGGGATTTGTCGTTTCTTCG 11029

LserLAC005780_C 10998 TAGCAGTTCTGGCTCTTATATATCTGTAGTTGGGATTTGTCGTTTCTTCG 11047

LsalLAC008020_C 10998 TAGCAGTTCTGGCTCTTATATATCTGTAGTTGGGATTTGTCGTTTCTTCG 11047

LsalCGN5271_CDS 10998 TAGCAGTTCTGGCTCTTATATATCTGTAGTTGGGATTTGTCGTTTCTTCG 11047

LsatLACWENDEL_C 11015 TGGTCGTAACAATCACTTCAAGCAGTGGAAACAACAAAAGATGCGCTCCA 11064

LsatSalinas_CDS 11015 TGGTCGTAACAATCACTTCAAGCAGTGGAAACAACAAAAGATGCGCTCCA 11064

LserUS96UC23_CD 11015 TGGTCGTAACAATCACTTCAAGCAGTGGAAACAACAAAAGATGCGCTCCA 11064

LvirLAC006941_C 11030 TGGTCGTAACAATCACTTCAAGCAGTGGAAACAACAAAAGATGCGCTCCA 11079

LserLAC005780_C 11048 TGGTCGTAACAATCACTTCAAGCAGTGGAAACAACAAAAGATGCGCTCCA 11097

LsalLAC008020_C 11048 TGGTCGTAACAATCACTTCAAGCAGTGGAAACAACAAAAGATGCGCTCCA 11097

LsalCGN5271_CDS 11048 TGGTCGTAACAATCACTTCAAGCAGTGGAAACAACAAAAGATGCGCTCCA 11097

LsatLACWENDEL_C 11065 AGTCCTTGGGCTGTTGAACAGAATCCAACCACACCGGAATGGATGGTACA 11114

LsatSalinas_CDS 11065 AGTCCTTGGGCTGTTGAACAGAATCCAACCACACCGGAATGGATGGTACA 11114

LserUS96UC23_CD 11065 AGTCCTTGGGCTGTTGAACAGAATCCAACCACACCGGAATGGATGGTACA 11114

LvirLAC006941_C 11080 AGTCCTTGGGCTGTTGAACAGAATCCAACCACACCGGAATGGATGGTACA 11129

LserLAC005780_C 11098 AGTCCTTGGGCTGTTGAACAGAATCCAACCACACCGGAATGGATGGTACA 11147

LsalLAC008020_C 11098 AGTCCTTGGGCTGTTGAACAGAATCCAACCACACCGGAATGGATGGTACA 11147

LsalCGN5271_CDS 11098 AGTCCTTGGGCTGTTGAACAGAATCCAACCACACCGGAATGGATGGTACA 11147

LsatLACWENDEL_C 11115 AAGTCCTCCAGCTTTTCATACTTTTGGAGAACTTCCAGCTATCAAGGAGA 11164

LsatSalinas_CDS 11115 AAGTCCTCCAGCTTTTCATACTTTTGGAGAACTTCCAGCTATCAAGGAGA 11164

LserUS96UC23_CD 11115 AAGTCCTCCAGCTTTTCATACTTTTGGAGAACTTCCAGCTATCAAGGAGA 11164

LvirLAC006941_C 11130 AAGTCCTCCAGCTTTTCATACTTTTGGAGAACTTCCAGCTATCAAGGAGA 11179

LserLAC005780_C 11148 AAGTCCTCCAGCTTTTCATACTTTTGGAGAACTTCCAGCTATCAAGGAGA 11197

LsalLAC008020_C 11148 AAGTCCTCCAGCTTTTCATACTTTTGGAGAACTTCCAGCTATCAAGGAGA 11197

LsalCGN5271_CDS 11148 AAGTCCTCCAGCTTTTCATACTTTTGGAGAACTTCCAGCTATCAAGGAGA 11197

LsatLACWENDEL_C 11165 CGAAAAGCTCTGTGAAGTAAATGATTGTTCTAGAATGGCTATTTCTCACA 11214

LsatSalinas_CDS 11165 CGAAAAGCTCTGTGAAGTAAATGATTGTTCTAGAATGGCTATTTCTCACA 11214

LserUS96UC23_CD 11165 CGAAAAGCTCTGTGAAGTAAATGATTGTTCTAGAATGGCTATTTCTCACA 11214

LvirLAC006941_C 11180 CGAAAAGCTCTGTGAAGTAAATGATTGTTCTAGAATGGCTATTTCTCACA 11229

LserLAC005780_C 11198 CGAAAAGCTCTGTGAAGTAAATGATTGTTCTAGAATGGCTATTTCTCACA 11247

LsalLAC008020_C 11198 CGAAAAGCTCTGTGAAGTAAATGATTGTTCTAGAATGGCTATTTCTCACA 11247

LsalCGN5271_CDS 11198 CGAAAAGCTCTGTGAAGTAAATGATTGTTCTAGAATGGCTATTTCTCACA 11247

LsatLACWENDEL_C 11215 ATTGCTCCTTGTGATGCAGCGGAACCATGGCAATTAGGATCTCAAGACGC 11264

LsatSalinas_CDS 11215 ATTGCTCCTTGTGATGCAGCGGAACCATGGCAATTAGGATCTCAAGACGC 11264

LserUS96UC23_CD 11215 ATTGCTCCTTGTGATGCAGCGGAACCATGGCAATTAGGATCTCAAGACGC 11264

LvirLAC006941_C 11230 ATTGCTCCTTGTGATGCAGCGGAACCATGGCAATTAGGATCTCAAGACGC 11279

LserLAC005780_C 11248 ATTGCTCCTTGTGATGCAGCGGAACCATGGCAATTAGGATCTCAAGACGC 11297

LsalLAC008020_C 11248 ATTGCTCCTTGTGATGCAGCGGAACCATGGCAATTAGGATCTCAAGACGC 11297

LsalCGN5271_CDS 11248 ATTGCTCCTTGTGATGCAGCGGAACCATGGCAATTAGGATCTCAAGACGC 11297

LsatLACWENDEL_C 11265 AGCAACACCTATGATGCAAGGAATAATAGACTTACATCACGATATCTTTT 11314

LsatSalinas_CDS 11265 AGCAACACCTATGATGCAAGGAATAATAGACTTACATCACGATATCTTTT 11314

LserUS96UC23_CD 11265 AGCAACACCTATGATGCAAGGAATAATAGACTTACATCACGATATCTTTT 11314

LvirLAC006941_C 11280 AGCAACACCTATGATGCAAGGAATAATAGACTTACATCACGATATCTTTT 11329

LserLAC005780_C 11298 AGCAACACCTATGATGCAAGGAATAATAGACTTACATCACGATATCTTTT 11347

LsalLAC008020_C 11298 AGCAACACCTATGATGCAAGGAATAATAGACTTACATCACGATATCTTTT 11347

LsalCGN5271_CDS 11298 AGCAACACCTATGATGCAAGGAATAATAGACTTACATCACGATATCTTTT 11347

LsatLACWENDEL_C 11315 TCTTCCTCATTCTTATTTTGGTTTTCGTCTCACGGATCTTGGTTCGCGCT 11364

LsatSalinas_CDS 11315 TCTTCCTCATTCTTATTTTGGTTTTCGTCTCACGGATCTTGGTTCGCGCT 11364

LserUS96UC23_CD 11315 TCTTCCTCATTCTTATTTTGGTTTTCGTCTCACGGATCTTGGTTCGCGCT 11364

LvirLAC006941_C 11330 TCTTCCTCATTCTTATTTTGGTTTTCGTCTCACGGATCTTGGTTCGCGCT 11379

LserLAC005780_C 11348 TCTTCCTCATTCTTATTTTGGTTTTCGTCTCACGGATCTTGGTTCGCGCT 11397

LsalLAC008020_C 11348 TCTTCCTCATTCTTATTTTGGTTTTCGTCTCACGGATCTTGGTTCGCGCT 11397

LsalCGN5271_CDS 11348 TCTTCCTCATTCTTATTTTGGTTTTCGTCTCACGGATCTTGGTTCGCGCT 11397

LsatLACWENDEL_C 11365 TTATGGCATTTCCAAAAAGAAAAAAATCCAATCCCGCAAAGGATTGTTCA 11414

LsatSalinas_CDS 11365 TTATGGCATTTCCAAAAAGAAAAAAATCCAATCCCGCAAAGGATTGTTCA 11414

LserUS96UC23_CD 11365 TTATGGCATTTCCAAAAAGAAAAAAATCCAATCCCGCAAAGGATTGTTCA 11414

LvirLAC006941_C 11380 TTATGGCATTTCCAAAAAGAAAAAAATCCAATCCCGCAAAGGATTGTTCA 11429

LserLAC005780_C 11398 TTATGGCATTTCCAAAAAGAAAAAAATCCAATCCCGCAAAGGATTGTTCA 11447

LsalLAC008020_C 11398 TTATGGCATTTCCAAAAAGAAAAAAATCCAATCCCGCAAAGGATTGTTCA 11447

LsalCGN5271_CDS 11398 TTATGGCATTTCCAAAAAGAAAAAAATCCAATCCCGCAAAGGATTGTTCA 11447

LsatLACWENDEL_C 11415 TGGAACTACTATCGAGATTCTTCGGACCATATTTCCTAGTATCATCCCGA 11464

LsatSalinas_CDS 11415 TGGAACTACTATCGAGATTCTTCGGACCATATTTCCTAGTATCATCCCGA 11464

LserUS96UC23_CD 11415 TGGAACTACTATCGAGATTCTTCGGACCATATTTCCTAGTATCATCCCGA 11464

LvirLAC006941_C 11430 TGGAACTACTATCGAGATTCTTCGGACCATATTTCCTAGTATCATCCCGA 11479

LserLAC005780_C 11448 TGGAACTACTATCGAGATTCTTCGGACCATATTTCCTAGTATCATCCCGA 11497

LsalLAC008020_C 11448 TGGAACTACTATCGAGATTCTTCGGACCATATTTCCTAGTATCATCCCGA 11497

LsalCGN5271_CDS 11448 TGGAACTACTATCGAGATTCTTCGGACCATATTTCCTAGTATCATCCCGA 11497

LsatLACWENDEL_C 11465 TGTTCATTGCTATACCATCATTTGCTCTCTTATACTCAATGGACGAGTTA 11514

LsatSalinas_CDS 11465 TGTTCATTGCTATACCATCATTTGCTCTCTTATACTCAATGGACGAGTTA 11514

LserUS96UC23_CD 11465 TGTTCATTGCTATACCATCATTTGCTCTCTTATACTCAATGGACGAGTTA 11514

LvirLAC006941_C 11480 TGTTCATTGCTATACCATCATTTGCTCTCTTATACTCAATGGACGAGTTA 11529

LserLAC005780_C 11498 TGTTCATTGCTATACCATCATTTGCTCTCTTATACTCAATGGACGAGGTA 11547

LsalLAC008020_C 11498 TGTTCATTGCTATACCATCATTTGCTCTCTTATACTCAATGGACGAGGTA 11547

LsalCGN5271_CDS 11498 TGTTCATTGCTATACCATCATTTGCTCTCTTATACTCAATGGACGAGGTA 11547

LsatLACWENDEL_C 11515 GTAGTAAATCCAGCCATTACTATCAAAGCTATTGGACATCAATGGTATCG 11564

LsatSalinas_CDS 11515 GTAGTAAATCCAGCCATTACTATCAAAGCTATTGGACATCAATGGTATCG 11564

LserUS96UC23_CD 11515 GTAGTAAATCCAGCCATTACTATCAAAGCTATTGGACATCAATGGTATCG 11564

LvirLAC006941_C 11530 GTAGTAAATCCAGCCATTACTATCAAAGCTATTGGACATCAATGGTATCG 11579

LserLAC005780_C 11548 GTAGTAAATCCAGCCATTACTATCAAAGCTATTGGACATCAATGGTATCG 11597

LsalLAC008020_C 11548 GTAGTAAATCCAGCCATTACTATCAAAGCTATTGGACATCAATGGTATCG 11597

LsalCGN5271_CDS 11548 GTAGTAAATCCAGCCATTACTATCAAAGCTATTGGACATCAATGGTATCG 11597

LsatLACWENDEL_C 11565 GAGTTATGAGTATTCGGACTATAACAGTTCCGATGAACAGTCACTCACTT 11614

LsatSalinas_CDS 11565 GAGTTATGAGTATTCGGACTATAACAGTTCCGATGAACAGTCACTCACTT 11614

LserUS96UC23_CD 11565 GAGTTATGAGTATTCGGACTATAACAGTTCCGATGAACAGTCACTCACTT 11614

LvirLAC006941_C 11580 GAGTTATGAGTATTCGGACTATAACAGTTCCGATGAACAGTCACTCACTT 11629

LserLAC005780_C 11598 GAGTTATGAGTATTCGGACTATAACAGTTCCGATGAACAGTCACTCACTT 11647

LsalLAC008020_C 11598 GAGTTATGAGTATTCGGACTATAACAGTTCCGATGAACAGTCACTCACTT 11647

LsalCGN5271_CDS 11598 GAGTTATGAGTATTCGGACTATAACAGTTCCGATGAACAGTCACTCACTT 11647

LsatLACWENDEL_C 11615 TTGACAGTTATACGATTCCAGAAGATGATCTAGAATTGGGTCAATTACGT 11664

LsatSalinas_CDS 11615 TTGACAGTTATACGATTCCAGAAGATGATCTAGAATTGGGTCAATTACGT 11664

LserUS96UC23_CD 11615 TTGACAGTTATACGATTCCAGAAGATGATCTAGAATTGGGTCAATTACGT 11664

LvirLAC006941_C 11630 TTGACAGTTATACGATTCCAGAAGATGATCTAGAATTGGGTCAATTACGT 11679

LserLAC005780_C 11648 TTGACAGTTATACGATTCCAGAAGATGATCTAGAATTGGGTCAATTACGT 11697

LsalLAC008020_C 11648 TTGACAGTTATACGATTCCAGAAGATGATCTAGAATTGGGTCAATTACGT 11697

LsalCGN5271_CDS 11648 TTGACAGTTATACGATTCCAGAAGATGATCTAGAATTGGGTCAATTACGT 11697

LsatLACWENDEL_C 11665 TTATTAGAAGTGGACAATCGAGTGGTTGTACCAGCCAAAAGTCATCTACG 11714

LsatSalinas_CDS 11665 TTATTAGAAGTGGACAATCGAGTGGTTGTACCAGCCAAAAGTCATCTACG 11714

LserUS96UC23_CD 11665 TTATTAGAAGTGGACAATCGAGTGGTTGTACCAGCCAAAAGTCATCTACG 11714

LvirLAC006941_C 11680 TTATTAGAAGTGGACAATCGAGTGGTTGTACCAGCCAAAAGTCATCTACG 11729

LserLAC005780_C 11698 TTATTAGAAGTGGACAATCGAGTGGTTGTACCAGCCAAAAGTCATCTACG 11747

LsalLAC008020_C 11698 TTATTAGAAGTGGACAATCGAGTGGTTGTACCAGCCAAAAGTCATCTACG 11747

LsalCGN5271_CDS 11698 TTATTAGAAGTGGACAATCGAGTGGTTGTACCAGCCAAAAGTCATCTACG 11747

LsatLACWENDEL_C 11715 TATTATTGTAACATCTGCTGATGTACTTCATAGTTGGGCTGTACCTTCCC 11764

LsatSalinas_CDS 11715 TATTATTGTAACATCTGCTGATGTACTTCATAGTTGGGCTGTACCTTCCC 11764

LserUS96UC23_CD 11715 TATTATTGTAACATCTGCTGATGTACTTCATAGTTGGGCTGTACCTTCCC 11764

LvirLAC006941_C 11730 TATTATTGTAACATCTGCTGATGTACTTCATAGTTGGGCTGTACCTTCCC 11779

LserLAC005780_C 11748 TATTATTGTAACATCTGCTGATGTACTTCATAGTTGGGCTGTACCTTCCC 11797

LsalLAC008020_C 11748 TATTATTGTAACATCTGCTGATGTACTTCATAGTTGGGCTGTACCTTCCC 11797

LsalCGN5271_CDS 11748 TATTATTGTAACATCTGCTGATGTACTTCATAGTTGGGCTGTACCTTCCC 11797

LsatLACWENDEL_C 11765 CAGGTGTAAAATGTGATGCTGTACCTGGTCGTTTAAATCAAACCTCTATT 11814

LsatSalinas_CDS 11765 CAGGTGTAAAATGTGATGCTGTACCTGGTCGTTTAAATCAAACCTCTATT 11814

LserUS96UC23_CD 11765 CAGGTGTAAAATGTGATGCTGTACCTGGTCGTTTAAATCAAACCTCTATT 11814

LvirLAC006941_C 11780 CAGGTGTAAAATGTGATGCTGTACCTGGTCGTTTAAATCAAACCTCTATT 11829

LserLAC005780_C 11798 CAGGTGTAAAATGTGATGCTGTACCTGGTCGTTTAAATCAAACCTCTATT 11847

LsalLAC008020_C 11798 CAGGTGTAAAATGTGATGCTGTACCTGGTCGTTTAAATCAAACCTCTATT 11847

LsalCGN5271_CDS 11798 CAGGTGTAAAATGTGATGCTGTACCTGGTCGTTTAAATCAAACCTCTATT 11847

LsatLACWENDEL_C 11815 TCGGTACAACGAGAAGGAGTTTACTATGGTCAGTGCAGTGAGATTTGTGG 11864

LsatSalinas_CDS 11815 TCGGTACAACGAGAAGGAGTTTACTATGGTCAGTGCAGTGAGATTTGTGG 11864

LserUS96UC23_CD 11815 TCGGTACAACGAGAAGGAGTTTACTATGGTCAGTGCAGTGAGATTTGTGG 11864

LvirLAC006941_C 11830 TCGGTACAACGAGAAGGAGTTTACTATGGTCAGTGCAGTGAGATTTGTGG 11879

LserLAC005780_C 11848 TCGGTACAACGAGAAGGAGTTTACTATGGTCAGTGCAGTGAGATTTGTGG 11897

LsalLAC008020_C 11848 TCGGTACAACGAGAAGGAGTTTACTATGGTCAGTGCAGTGAGATTTGTGG 11897

LsalCGN5271_CDS 11848 TCGGTACAACGAGAAGGAGTTTACTATGGTCAGTGCAGTGAGATTTGTGG 11897

LsatLACWENDEL_C 11865 AACTAATCATGCCTTTATGCGTGCGCCCGGAAAGATAGGCCGACTGCTGA 11914

LsatSalinas_CDS 11865 AACTAATCATGCCTTTATGCGTGCGCCCGGAAAGATAGGCCGACTGCTGA 11914

LserUS96UC23_CD 11865 AACTAATCATGCCTTTATGCGTGCGCCCGGAAAGATAGGCCGACTGCTGA 11914

LvirLAC006941_C 11880 AACTAATCATGCCTTTATGCGTGCGCCCGGAAAGATAGGCCGACTGCTGA 11929

LserLAC005780_C 11898 AACTAATCATGCCTTTATGCGTGCGCCCGGAAAGATAGGCCGACTGCTGA 11947

LsalLAC008020_C 11898 AACTAATCATGCCTTTATGCGTGCGCCCGGAAAGATAGGCCGACTGCTGA 11947

LsalCGN5271_CDS 11898 AACTAATCATGCCTTTATGCGTGCGCCCGGAAAGATAGGCCGACTGCTGA 11947

LsatLACWENDEL_C 11915 GCCCACTCTGGCTCAGCCGCACCACCCAGGGTGCGAGCCACCCGAGAAGC 11964

LsatSalinas_CDS 11915 GCCCACTCTGGCTCAGCCGCACCACCCAGGGTGCGAGCCACCCGAGAAGC 11964

LserUS96UC23_CD 11915 GCCCACTCTGGCTCAGCCGCACCACCCAGGGTGCGAGCCACCCGAGAAGC 11964

LvirLAC006941_C 11930 GCCCACTCTGGCTCAGCCGCACCACCCAGGGTGCGAGCCACCCGAGAAGC 11979

LserLAC005780_C 11948 GCCCACTCTGGCTCAGCCGCACCACCCAGGGTGCGAGCCACCCGAGAAGC 11997

LsalLAC008020_C 11948 GCCCACTCTGGCTCAGCCGCACCACCCAGGGTGCGAGCCACCCGAGAAGC 11997

LsalCGN5271_CDS 11948 GCCCACTCTGGCTCAGCCGCACCACCCAGGGTGCGAGCCACCCGAGAAGC 11997

LsatLACWENDEL_C 11965 AAGCTATTACAGCGAGCGGCTGGAGCAGTATGGAGCCGAGGAGCAAGGCA 12014

LsatSalinas_CDS 11965 AAGCTATTACAGCGAGCGGCTGGAGCAGTATGGAGCCGAGGAGCAAGGCA 12014

LserUS96UC23_CD 11965 AAGCTATTACAGCGAGCGGCTGGAGCAGTATGGAGCCGAGGAGCAAGGCA 12014

LvirLAC006941_C 11980 AAGCTATTACAGCGAGCGGCTGGAGCAGTATGGAGCCGAGGAGCAAGGCA 12029

LserLAC005780_C 11998 AAGCTATTACAGCGAGCGGCTGGAGCAGTATGGAGCCGAGGAGCAAGGCA 12047

LsalLAC008020_C 11998 AAGCTATTACAGCGAGCGGCTGGAGCAGTATGGAGCCGAGGAGCAAGGCA 12047

LsalCGN5271_CDS 11998 AAGCTATTACAGCGAGCGGCTGGAGCAGTATGGAGCCGAGGAGCAAGGCA 12047

LsatLACWENDEL_C 12015 GTAGATGATTGAATCTCAGAGGCACTCTTATCATTTGGTAGATCCAAGTC 12064

LsatSalinas_CDS 12015 GTAGATGATTGAATCTCAGAGGCACTCTTATCATTTGGTAGATCCAAGTC 12064

LserUS96UC23_CD 12015 GTAGATGATTGAATCTCAGAGGCACTCTTATCATTTGGTAGATCCAAGTC 12064

LvirLAC006941_C 12030 GTAGATGATTGAATCTCAGAGGCACTCTTATCATTTGGTAGATCCAAGTC 12079

LserLAC005780_C 12048 GTAGATGATTGAATCTCAGAGGCACTCTTATCATTTGGTAGATCCAAGTC 12097

LsalLAC008020_C 12048 GTAGATGATTGAATCTCAGAGGCACTCTTATCATTTGGTAGATCCAAGTC 12097

LsalCGN5271_CDS 12048 GTAGATGATTGAATCTCAGAGGCACTCTTATCATTTGGTAGATCCAAGTC 12097

LsatLACWENDEL_C 12065 CATGGCCTATTTCGGGTTCACTCGGAGCTTTGGCAACCACCGTAGGAGGT 12114

LsatSalinas_CDS 12065 CATGGCCTATTTCGGGTTCACTCGGAGCTTTGGCAACCACCGTAGGAGGT 12114

LserUS96UC23_CD 12065 CATGGCCTATTTCGGGTTCACTCGGAGCTTTGGCAACCACCGTAGGAGGT 12114

LvirLAC006941_C 12080 CATGGCCTATTTCGGGTTCACTCGGAGCTTTGGCAACCACCGTAGGAGGT 12129

LserLAC005780_C 12098 CATGGCCTATTTCGGGTTCACTCGGAGCTTTGGCAACCACCGTAGGAGGT 12147

LsalLAC008020_C 12098 CATGGCCTATTTCGGGTTCACTCGGAGCTTTGGCAACCACCGTAGGAGGT 12147

LsalCGN5271_CDS 12098 CATGGCCTATTTCGGGTTCACTCGGAGCTTTGGCAACCACCGTAGGAGGT 12147

LsatLACWENDEL_C 12115 GTGATGTACATGCACTCATTTCAAGGGGGTGCAACACTTCTCAGTTTGGG 12164

LsatSalinas_CDS 12115 GTGATGTACATGCACTCATTTCAAGGGGGTGCAACACTTCTCAGTTTGGG 12164

LserUS96UC23_CD 12115 GTGATGTACATGCACTCATTTCAAGGGGGTGCAACACTTCTCAGTTTGGG 12164

LvirLAC006941_C 12130 GTGATGTACATGCACTCATTTCAAGGGGGTGCAACACTTCTCAGTTTGGG 12179

LserLAC005780_C 12148 GTGATGTACATGCACTCATTTCAAGGGGGTGCAACACTTCTCAGTTTGGG 12197

LsalLAC008020_C 12148 GTGATGTACATGCACTCATTTCAAGGGGGTGCAACACTTCTCAGTTTGGG 12197

LsalCGN5271_CDS 12148 GTGATGTACATGCACTCATTTCAAGGGGGTGCAACACTTCTCAGTTTGGG 12197

LsatLACWENDEL_C 12165 CCTAATCTTTATCCTATATACCATGTTCGTATGGTGGCGCGATGTTCTAC 12214

LsatSalinas_CDS 12165 CCTAATCTTTATCCTATATACCATGTTCGTATGGTGGCGCGATGTTCTAC 12214

LserUS96UC23_CD 12165 CCTAATCTTTATCCTATATACCATGTTCGTATGGTGGCGCGATGTTCTAC 12214

LvirLAC006941_C 12180 CCTAATCTTTATCCTATATACCATGTTCGTATGGTGGCGCGATGTTCTAC 12229

LserLAC005780_C 12198 CCTAATCTTTATCCTATATACCATGTTCGTATGGTGGCGCGATGTTCTAC 12247

LsalLAC008020_C 12198 CCTAATCTTTATCCTATATACCATGTTCGTATGGTGGCGCGATGTTCTAC 12247

LsalCGN5271_CDS 12198 CCTAATCTTTATCCTATATACCATGTTCGTATGGTGGCGCGATGTTCTAC 12247

LsatLACWENDEL_C 12215 GTGAATCCACGTTGGAAGGACATCATACCAAAGTCGTACAATTAGGACCT 12264

LsatSalinas_CDS 12215 GTGAATCCACGTTGGAAGGACATCATACCAAAGTCGTACAATTAGGACCT 12264

LserUS96UC23_CD 12215 GTGAATCCACGTTGGAAGGACATCATACCAAAGTCGTACAATTAGGACCT 12264

LvirLAC006941_C 12230 GTGAATCCACGTTGGAAGGACATCATACCAAAGTCGTACAATTAGGACCT 12279

LserLAC005780_C 12248 GTGAATCCACGTTGGAAGGACATCATACCAAAGTCGTACAATTAGGACCT 12297

LsalLAC008020_C 12248 GTGAATCCACGTTGGAAGGACATCATACCAAAGTCGTACAATTAGGACCT 12297

LsalCGN5271_CDS 12248 GTGAATCCACGTTGGAAGGACATCATACCAAAGTCGTACAATTAGGACCT 12297

LsatLACWENDEL_C 12265 CGATATGGGTTTATTCTGTTTATCGTTTCGGAGGTTATGTTCCTTTTTGC 12314

LsatSalinas_CDS 12265 CGATATGGGTTTATTCTGTTTATCGTTTCGGAGGTTATGTTCCTTTTTGC 12314

LserUS96UC23_CD 12265 CGATATGGGTTTATTCTGTTTATCGTTTCGGAGGTTATGTTCCTTTTTGC 12314

LvirLAC006941_C 12280 CGATATGGGTTTATTCTGTTTATCGTTTCGGAGGTTATGTTCCTTTTTGC 12329

LserLAC005780_C 12298 CGATATGGGTTTATTCTGTTTATCGTTTCGGAGGTTATGTTCCTTTTTGC 12347

LsalLAC008020_C 12298 CGATATGGGTTTATTCTGTTTATCGTTTCGGAGGTTATGTTCCTTTTTGC 12347

LsalCGN5271_CDS 12298 CGATATGGGTTTATTCTGTTTATCGTTTCGGAGGTTATGTTCCTTTTTGC 12347

LsatLACWENDEL_C 12315 TCTTTTTCGGGCTTCTTCTCATTCTTCTTTGGCACCTACGGTAGAGATCG 12364

LsatSalinas_CDS 12315 TCTTTTTCGGGCTTCTTCTCATTCTTCTTTGGCACCTACGGTAGAGATCG 12364

LserUS96UC23_CD 12315 TCTTTTTCGGGCTTCTTCTCATTCTTCTTTGGCACCTACGGTAGAGATCG 12364

LvirLAC006941_C 12330 TCTTTTTCGGGCTTCTTCTCATTCTTCTTTGGCACCTACGGTAGAGATCG 12379

LserLAC005780_C 12348 TCTTTTTCGGGCTTCTTCTCATTCTTCTTTGGCACCTACGGTAGAGATCG 12397

LsalLAC008020_C 12348 TCTTTTTCGGGCTTCTTCTCATTCTTCTTTGGCACCTACGGTAGAGATCG 12397

LsalCGN5271_CDS 12348 TCTTTTTCGGGCTTCTTCTCATTCTTCTTTGGCACCTACGGTAGAGATCG 12397

LsatLACWENDEL_C 12365 GAGGTATTTGGCCCCCAAAAGGGATTGCGGTTTTAGATCCTCGGGAAATC 12414

LsatSalinas_CDS 12365 GAGGTATTTGGCCCCCAAAAGGGATTGCGGTTTTAGATCCTCGGGAAATC 12414

LserUS96UC23_CD 12365 GAGGTATTTGGCCCCCAAAAGGGATTGCGGTTTTAGATCCTCGGGAAATC 12414

LvirLAC006941_C 12380 GAGGTATTTGGCCCCCAAAAGGGATTGCGGTTTTAGATCCTCGGGAAATC 12429

LserLAC005780_C 12398 GAGGTATTTGGCCCCCAAAAGGGATTGCGGTTTTAGATCCTCGGGAAATC 12447

LsalLAC008020_C 12398 GAGGTATTTGGCCCCCAAAAGGGATTGCGGTTTTAGATCCTCGGGAAATC 12447

LsalCGN5271_CDS 12398 GAGGTATTTGGCCCCCAAAAGGGATTGCGGTTTTAGATCCTCGGGAAATC 12447

LsatLACWENDEL_C 12415 CCTTTTCTTAATACTCTTATTCCCCTTTCATCCGGAGCTGCCGTAACTTG 12464

LsatSalinas_CDS 12415 CCTTTTCTTAATACTCTTATTCCCCTTTCATCCGGAGCTGCCGTAACTTG 12464

LserUS96UC23_CD 12415 CCTTTTCTTAATACTCTTATTCCCCTTTCATCCGGAGCTGCCGTAACTTG 12464

LvirLAC006941_C 12430 CCTTTTCTTAATACTCTTATTCCCCTTTCATCCGGAGCTGCCGTAACTTG 12479

LserLAC005780_C 12448 CCTTTTCTTAATACTCTTATTCCCCTTTCATCCGGAGCTGCCGTAACTTG 12497

LsalLAC008020_C 12448 CCTTTTCTTAATACTCTTATTCCCCTTTCATCCGGAGCTGCCGTAACTTG 12497

LsalCGN5271_CDS 12448 CCTTTTCTTAATACTCTTATTCCCCTTTCATCCGGAGCTGCCGTAACTTG 12497

LsatLACWENDEL_C 12465 GGCTCATCATGCTATACTCGCGGGGAAGGAAAAACGAGCAGTTTACGCTT 12514

LsatSalinas_CDS 12465 GGCTCATCATGCTATACTCGCGGGGAAGGAAAAACGAGCAGTTTACGCTT 12514

LserUS96UC23_CD 12465 GGCTCATCATGCTATACTCGCGGGGAAGGAAAAACGAGCAGTTTACGCTT 12514

LvirLAC006941_C 12480 GGCTCATCATGCTATACTCGCGGGGAAGGAAAAACGAGCAGTTTACGCTT 12529

LserLAC005780_C 12498 GGCTCATCATGCTATACTCGCGGGGAAGGAAAAACGAGCAGTTTACGCTT 12547

LsalLAC008020_C 12498 GGCTCATCATGCTATACTCGCGGGGAAGGAAAAACGAGCAGTTTACGCTT 12547

LsalCGN5271_CDS 12498 GGCTCATCATGCTATACTCGCGGGGAAGGAAAAACGAGCAGTTTACGCTT 12547

LsatLACWENDEL_C 12515 TAGTAGCTACCGTTTCACTGGCTCTAGTATTCACCGCCTTTCAAGGAATG 12564

LsatSalinas_CDS 12515 TAGTAGCTACCGTTTCACTGGCTCTAGTATTCACCGCCTTTCAAGGAATG 12564

LserUS96UC23_CD 12515 TAGTAGCTACCGTTTCACTGGCTCTAGTATTCACCGCCTTTCAAGGAATG 12564

LvirLAC006941_C 12530 TAGTAGCTACCGTTTCACTGGCTCTAGTATTCACCGCCTTTCAAGGAATG 12579

LserLAC005780_C 12548 TAGTAGCTACCGTTTCACTGGCTCTAGTATTCACCGCCTTTCAAGGAATG 12597

LsalLAC008020_C 12548 TAGTAGCTACCGTTTCACTGGCTCTAGTATTCACCGCCTTTCAAGGAATG 12597

LsalCGN5271_CDS 12548 TAGTAGCTACCGTTTCACTGGCTCTAGTATTCACCGCCTTTCAAGGAATG 12597

LsatLACWENDEL_C 12565 GAATATTATCAAGCGCCCCCCACAATTTCGGATAGTATTTATGGTTCTAC 12614

LsatSalinas_CDS 12565 GAATATTATCAAGCGCCCCCCACAATTTCGGATAGTATTTATGGTTCTAC 12614

LserUS96UC23_CD 12565 GAATATTATCAAGCGCCCCCCACAATTTCGGATAGTATTTATGGTTCTAC 12614

LvirLAC006941_C 12580 GAATATTATCAAGCGCCCCCCACAATTTCGGATAGTATTTATGGTTCTAC 12629

LserLAC005780_C 12598 GAATATTATCAAGCGCCCCCCACAATTTCGGATAGTATTTATGGTTCTAC 12647

LsalLAC008020_C 12598 GAATATTATCAAGCGCCCCCCACAATTTCGGATAGTATTTATGGTTCTAC 12647

LsalCGN5271_CDS 12598 GAATATTATCAAGCGCCCCCCACAATTTCGGATAGTATTTATGGTTCTAC 12647

LsatLACWENDEL_C 12615 CTTTTTCTTAGCAACTGGCTTTCATGGTTTTCATGTGATTATAGGTACTC 12664

LsatSalinas_CDS 12615 CTTTTTCTTAGCAACTGGCTTTCATGGTTTTCATGTGATTATAGGTACTC 12664

LserUS96UC23_CD 12615 CTTTTTCTTAGCAACTGGCTTTCATGGTTTTCATGTGATTATAGGTACTC 12664

LvirLAC006941_C 12630 CTTTTTCTTAGCAACTGGCTTTCATGGTTTTCATGTGATTATAGGTACTC 12679

LserLAC005780_C 12648 CTTTTTCTTAGCAACTGGCTTTCATGGTTTTCATGTGATTATAGGTACTC 12697

LsalLAC008020_C 12648 CTTTTTCTTAGCAACTGGCTTTCATGGTTTTCATGTGATTATAGGTACTC 12697

LsalCGN5271_CDS 12648 CTTTTTCTTAGCAACTGGCTTTCATGGTTTTCATGTGATTATAGGTACTC 12697

LsatLACWENDEL_C 12665 TTTTCTCGATCATATGTGGTATTCGCCAATATCTTGGTCATCTGACCAAG 12714

LsatSalinas_CDS 12665 TTTTCTCGATCATATGTGGTATTCGCCAATATCTTGGTCATCTGACCAAG 12714

LserUS96UC23_CD 12665 TTTTCTCGATCATATGTGGTATTCGCCAATATCTTGGTCATCTGACCAAG 12714

LvirLAC006941_C 12680 TTTTCTCGATCATATGTGGTATTCGCCAATATCTTGGTCATCTGACCAAG 12729

LserLAC005780_C 12698 TTTTCTCGATCATATGTGGTATTCGCCAATATCTTGGTCATCTGACCAAG 12747

LsalLAC008020_C 12698 TTTTCTCGATCATATGTGGTATTCGCCAATATCTTGGTCATCTGACCAAG 12747

LsalCGN5271_CDS 12698 TTTTCTCGATCATATGTGGTATTCGCCAATATCTTGGTCATCTGACCAAG 12747

LsatLACWENDEL_C 12715 GAGCATCACGTTGGCTTTGAAGCAGCTGCATGGTACTGGCATTTTGTAGA 12764

LsatSalinas_CDS 12715 GAGCATCACGTTGGCTTTGAAGCAGCTGCATGGTACTGGCATTTTGTAGA 12764

LserUS96UC23_CD 12715 GAGCATCACGTTGGCTTTGAAGCAGCTGCATGGTACTGGCATTTTGTAGA 12764

LvirLAC006941_C 12730 GAGCATCACGTTGGCTTTGAAGCAGCTGCATGGTACTGGCATTTTGTAGA 12779

LserLAC005780_C 12748 GAGCATCACGTTGGCTTTGAAGCAGCTGCATGGTACTGGCATTTTGTAGA 12797

LsalLAC008020_C 12748 GAGCATCACGTTGGCTTTGAAGCAGCTGCATGGTACTGGCATTTTGTAGA 12797

LsalCGN5271_CDS 12748 GAGCATCACGTTGGCTTTGAAGCAGCTGCATGGTACTGGCATTTTGTAGA 12797

LsatLACWENDEL_C 12765 CGTGGTTCGGTTATTCCCATTTGTCTCTATCTATTGGTGGGGAGGTATAT 12814

LsatSalinas_CDS 12765 CGTGGTTCGGTTATTCCCATTTGTCTCTATCTATTGGTGGGGAGGTATAT 12814

LserUS96UC23_CD 12765 CGTGGTTCGGTTATTCCCATTTGTCTCTATCTATTGGTGGGGAGGTATAT 12814

LvirLAC006941_C 12780 CGTGGTTCGGTTATTCCCATTTGTCTCTATCTATTGGTGGGGAGGTATAT 12829

LserLAC005780_C 12798 CGTGGTTCGGTTATTCCCATTTGTCTCTATCTATTGGTGGGGAGGTATAT 12847

LsalLAC008020_C 12798 CGTGGTTCGGTTATTCCCATTTGTCTCTATCTATTGGTGGGGAGGTATAT 12847

LsalCGN5271_CDS 12798 CGTGGTTCGGTTATTCCCATTTGTCTCTATCTATTGGTGGGGAGGTATAT 12847

LsatLACWENDEL_C 12815 GAATGAAAGAGGCGATCAGAATGGTACTCGAATCCATTTACGATCCCGAG 12864

LsatSalinas_CDS 12815 GAATGAAAGAGGCGATCAGAATGGTACTCGAATCCATTTACGATCCCGAG 12864

LserUS96UC23_CD 12815 GAATGAAAGAGGCGATCAGAATGGTACTCGAATCCATTTACGATCCCGAG 12864

LvirLAC006941_C 12830 GAATGAAAGAGGCGATCAGAATGGTACTCGAATCCATTTACGATCCCGAG 12879

LserLAC005780_C 12848 GAATGAAAGAGGCGATCAGAATGGTACTCGAATCCATTTACGATCCCGAG 12897

LsalLAC008020_C 12848 GAATGAAAGAGGCGATCAGAATGGTACTCGAATCCATTTACGATCCCGAG 12897

LsalCGN5271_CDS 12848 GAATGAAAGAGGCGATCAGAATGGTACTCGAATCCATTTACGATCCCGAG 12897

LsatLACWENDEL_C 12865 TTTCCAGACACATCGCACTTCCGCTCGGGTCGAGGCTGCCACTCGGCCCT 12914

LsatSalinas_CDS 12865 TTTCCAGACACATCGCACTTCCGCTCGGGTCGAGGCTGCCACTCGGCCCT 12914

LserUS96UC23_CD 12865 TTTCCAGACACATCGCACTTCCGCTCGGGTCGAGGCTGCCACTCGGCCCT 12914

LvirLAC006941_C 12880 TTTCCAGACACATCGCACTTCCGCTCGGGTCGAGGCTGCCACTCGGCCCT 12929

LserLAC005780_C 12898 TTTCCAGACACATCGCACTTCCGCTCGGGTCGAGGCTGCCACTCGGCCCT 12947

LsalLAC008020_C 12898 TTTCCAGACACATCGCACTTCCGCTCGGGTCGAGGCTGCCACTCGGCCCT 12947

LsalCGN5271_CDS 12898 TTTCCAGACACATCGCACTTCCGCTCGGGTCGAGGCTGCCACTCGGCCCT 12947

LsatLACWENDEL_C 12915 AAGACGGATCAAAGAAGAGTGGGGAACCTCTCGCTGGTTTTTGGAATTCG 12964

LsatSalinas_CDS 12915 AAGACGGATCAAAGAAGAGTGGGGAACCTCTCGCTGGTTTTTGGAATTCG 12964

LserUS96UC23_CD 12915 AAGACGGATCAAAGAAGAGTGGGGAACCTCTCGCTGGTTTTTGGAATTCG 12964

LvirLAC006941_C 12930 AAGACGGATCAAAGAAGAGTGGGGAACCTCTCGCTGGTTTTTGGAATTCG 12979

LserLAC005780_C 12948 AAGACGGATCAAAGAAGAGTGGGGAACCTCTCGCTGGTTTTTGGAATTCG 12997

LsalLAC008020_C 12948 AAGACGGATCAAAGAAGAGTGGGGAACCTCTCGCTGGTTTTTGGAATTCG 12997

LsalCGN5271_CDS 12948 AAGACGGATCAAAGAAGAGTGGGGAACCTCTCGCTGGTTTTTGGAATTCG 12997

LsatLACWENDEL_C 12965 ACATCAGGAAGTGTTTTCACACCATCGACCGACATCGACTCATCCCCATC 13014

LsatSalinas_CDS 12965 ACATCAGGAAGTGTTTTCACACCATCGACCGACATCGACTCATCCCCATC 13014

LserUS96UC23_CD 12965 ACATCAGGAAGTGTTTTCACACCATCGACCGACATCGACTCATCCCCATC 13014

LvirLAC006941_C 12980 ACATCAGGAAGTGTTTTCACACCATCGACCGACATCGACTCATCCCCATC 13029

LserLAC005780_C 12998 ACATCAGGAAGTGTTTTCACACCATCGACCGACATCGACTCATCCCCATC 13047

LsalLAC008020_C 12998 ACATCAGGAAGTGTTTTCACACCATCGACCGACATCGACTCATCCCCATC 13047

LsalCGN5271_CDS 12998 ACATCAGGAAGTGTTTTCACACCATCGACCGACATCGACTCATCCCCATC 13047

LsatLACWENDEL_C 13015 TTTAAGGAAGAGATCGGCGACCCCAAGTTCTTTTACCCCATTCAGAAAGT 13064

LsatSalinas_CDS 13015 TTTAAGGAAGAGATCGGCGACCCCAAGTTCTTTTACCCCATTCAGAAAGT 13064

LserUS96UC23_CD 13015 TTTAAGGAAGAGATCGGCGACCCCAAGTTCTTTTACCCCATTCAGAAAGT 13064

LvirLAC006941_C 13030 TTTAAGGAAGAGATCGGCGACCCCAAGTTCTTTTACCCCATTCAGAAAGT 13079

LserLAC005780_C 13048 TTTAAGGAAGAGATCGGCGACCCCAAGTTCTTTTACCCCATTCAGAAAGT 13097

LsalLAC008020_C 13048 TTTAAGGAAGAGATCGGCGACCCCAAGTTCTTTTACCCCATTCAGAAAGT 13097

LsalCGN5271_CDS 13048 TTTAAGGAAGAGATCGGCGACCCCAAGTTCTTTTACCCCATTCAGAAAGT 13097

LsatLACWENDEL_C 13065 ATTTTCTGCCGGACGACTCGTAGGGGCGGAGAAGGGCCCTTACTCCGTCC 13114

LsatSalinas_CDS 13065 ATTTTCTGCCGGACGACTCGTAGGGGCGGAGAAGGGCCCTTACTCCGTCC 13114

LserUS96UC23_CD 13065 ATTTTCTGCCGGACGACTCGTAGGGGCGGAGAAGGGCCCTTACTCCGTCC 13114

LvirLAC006941_C 13080 ATTTTCTGCCGGACGACTCGTAGGGGCGGAGAAGGGCCCTTACTCCGTCC 13129

LserLAC005780_C 13098 ATTTTCTGCCGGACGACTCGTAGGGGCGGAGAAGGGCCCTTACTCCGTCC 13147

LsalLAC008020_C 13098 ATTTTCTGCCGGACGACTCGTAGGGGCGGAGAAGGGCCCTTACTCCGTCC 13147

LsalCGN5271_CDS 13098 ATTTTCTGCCGGACGACTCGTAGGGGCGGAGAAGGGCCCTTACTCCGTCC 13147

LsatLACWENDEL_C 13115 CACACAGTGTATTACTATCGGCCCTACCAGACAACATCTACCTACACAAG 13164

LsatSalinas_CDS 13115 CACACAGTGTATTACTATCGGCCCTACCAGACAACATCTACCTACACAAG 13164

LserUS96UC23_CD 13115 CACACAGTGTATTACTATCGGCCCTACCAGACAACATCTACCTACACAAG 13164

LvirLAC006941_C 13130 CACACAGTGTATTACTATCGGCCCTACCAGACAACATCTACCTACACAAG 13179

LserLAC005780_C 13148 CACACAGTGTATTACTATCGGCCCTACCAGACAACATCTACCTACACAAG 13197

LsalLAC008020_C 13148 CACACAGTGTATTACTATCGGCCCTACCAGACAACATCTACCTACACAAG 13197

LsalCGN5271_CDS 13148 CACACAGTGTATTACTATCGGCCCTACCAGACAACATCTACCTACACAAG 13197

LsatLACWENDEL_C 13165 CTCGATCAGGAGATAGGGAGGATCCAACAGAAGTACGAAATTCCGATTGT 13214

LsatSalinas_CDS 13165 CTCGATCAGGAGATAGGGAGGATCCAACAGAAGTACGAAATTCCGATTGT 13214

LserUS96UC23_CD 13165 CTCGATCAGGAGATAGGGAGGATCCAACAGAAGTACGAAATTCCGATTGT 13214

LvirLAC006941_C 13180 CTCGATCAGGAGATAGGGAGGATCCAACAGAAGTACGAAATTCCGATTGT 13229

LserLAC005780_C 13198 CTCGATCAGGAGATAGGGAGGATCCAACAGAAGTACGAAATTCCGATTGT 13247

LsalLAC008020_C 13198 CTCGATCAGGAGATAGGGAGGATCCAACAGAAGTACGAAATTCCGATTGT 13247

LsalCGN5271_CDS 13198 CTCGATCAGGAGATAGGGAGGATCCAACAGAAGTACGAAATTCCGATTGT 13247

LsatLACWENDEL_C 13215 TCAGAGAATAAGATCGGTTCTATTAAGGACAGGTCGTATTGATGACCAAG 13264

LsatSalinas_CDS 13215 TCAGAGAATAAGATCGGTTCTATTAAGGACAGGTCGTATTGATGACCAAG 13264

LserUS96UC23_CD 13215 TCAGAGAATAAGATCGGTTCTATTAAGGACAGGTCGTATTGATGACCAAG 13264

LvirLAC006941_C 13230 TCAGAGAATAAGATCGGTTCTATTAAGGACAGGTCGTATTGATGACCAAG 13279

LserLAC005780_C 13248 TCAGAGAATAAGATCGGTTCTATTAAGGACAGGTCGTATTGATGACCAAG 13297

LsalLAC008020_C 13248 TCAGAGAATAAGATCGGTTCTATTAAGGACAGGTCGTATTGATGACCAAG 13297

LsalCGN5271_CDS 13248 TCAGAGAATAAGATCGGTTCTATTAAGGACAGGTCGTATTGATGACCAAG 13297

LsatLACWENDEL_C 13265 AAGACTCTGGAGAAGAAGCAAGCTTCAACCCTCCCCAAGACAACAGAGCC 13314

LsatSalinas_CDS 13265 AAGACTCTGGAGAAGAAGCAAGCTTCAACCCTCCCCAAGACAACAGAGCC 13314

LserUS96UC23_CD 13265 AAGACTCTGGAGAAGAAGCAAGCTTCAACCCTCCCCAAGACAACAGAGCC 13314

LvirLAC006941_C 13280 AAGACTCTGGAGAAGAAGCAAGCTTCAACCCTCCCCAAGACAACAGAGCC 13329

LserLAC005780_C 13298 AAGACTCTGGAGAAGAAGCAAGCTTCAACCCTCCCCAAGACAACAGAGCC 13347

LsalLAC008020_C 13298 AAGACTCTGGAGAAGAAGCAAGCTTCAACCCTCCCCAAGACAACAGAGCC 13347

LsalCGN5271_CDS 13298 AAGACTCTGGAGAAGAAGCAAGCTTCAACCCTCCCCAAGACAACAGAGCC 13347

LsatLACWENDEL_C 13315 ATCATTGTGGGGAGGGTAAAGAGCATCCAACGCAAAGCGGCCTTTCATTC 13364

LsatSalinas_CDS 13315 ATCATTGTGGGGAGGGTAAAGAGCATCCAACGCAAAGCGGCCTTTCATTC 13364

LserUS96UC23_CD 13315 ATCATTGTGGGGAGGGTAAAGAGCATCCAACGCAAAGCGGCCTTTCATTC 13364

LvirLAC006941_C 13330 ATCATTGTGGGGAGGGTAAAGAGCATCCAACGCAAAGCGGCCTTTCATTC 13379

LserLAC005780_C 13348 ATCATTGTGGGGAGGGTAAAGAGCATCCAACGCAAAGCGGCCTTTCATTC 13397

LsalLAC008020_C 13348 ATCATTGTGGGGAGGGTAAAGAGCATCCAACGCAAAGCGGCCTTTCATTC 13397

LsalCGN5271_CDS 13348 ATCATTGTGGGGAGGGTAAAGAGCATCCAACGCAAAGCGGCCTTTCATTC 13397

LsatLACWENDEL_C 13365 CCTTGTTTCGTCGTGGCACACCCCTCCCACAAGCACCCCCCGGCTCAGGG 13414

LsatSalinas_CDS 13365 CCTTGTTTCGTCGTGGCACACCCCTCCCACAAGCACCCCCCGGCTCAGGG 13414

LserUS96UC23_CD 13365 CCTTGTTTCGTCGTGGCACACCCCTCCCACAAGCACCCCCCGGCTCAGGG 13414

LvirLAC006941_C 13380 CCTTGTTTCGTCGTGGCACACCCCTCCCACAAGCACCCCCCGGCTCAGGG 13429

LserLAC005780_C 13398 CCTTGTTTCGTCGTGGCACACCCCTCCCACAAGCACCCCCCGGCTCAGGG 13447

LsalLAC008020_C 13398 CCTTGTTTCGTCGTGGCACACCCCTCCCACAAGCACCCCCCGGCTCAGGG 13447

LsalCGN5271_CDS 13398 CCTTGTTTCGTCGTGGCACACCCCTCCCACAAGCACCCCCCGGCTCAGGG 13447

LsatLACWENDEL_C 13415 GGGACCAGAAAAGGTCTTTCGTTTTCCCCCCTGAGTCGGCCCTTGCCGCC 13464

LsatSalinas_CDS 13415 GGGACCAGAAAAGGTCTTTCGTTTTCCCCCCTGAGTCGGCCCTTGCCGCC 13464

LserUS96UC23_CD 13415 GGGACCAGAAAAGGTCTTTCGTTTTCCCCCCTGAGTCGGCCCTTGCCGCC 13464

LvirLAC006941_C 13430 GGGACCAGAAAAGGTCTTTCGTTTTCCCCCCTGAGTCGGCCCTTGCCGCC 13479

LserLAC005780_C 13448 GGGACCAGAAAAGGTCTTTCGTTTTCCCCCCTGAGTCGGCCCTTGCCGCC 13497

LsalLAC008020_C 13448 GGGACCAGAAAAGGTCTTTCGTTTTCCCCCCTGAGTCGGCCCTTGCCGCC 13497

LsalCGN5271_CDS 13448 GGGACCAGAAAAGGTCTTTCGTTTTCCCCCCTGAGTCGGCCCTTGCCGCC 13497

LsatLACWENDEL_C 13465 TTCCTTAACAAGCCCTCGAGCCTCCTTTGCGCCGCCTTCCTAATAGAAGC 13514

LsatSalinas_CDS 13465 TTCCTTAACAAGCCCTCGAGCCTCCTTTGCGCCGCCTTCCTAATAGAAGC 13514

LserUS96UC23_CD 13465 TTCCTTAACAAGCCCTCGAGCCTCCTTTGCGCCGCCTTCCTAATAGAAGC 13514

LvirLAC006941_C 13480 TTCCTTAACAAGCCCTCGAGCCTCCTTTGCGCCGCCTTCCTAATAGAAGC 13529

LserLAC005780_C 13498 TTCCTTAACAAGCCCTCGAGCCTCCTTTGCGCCGCCTTCCTAATAGAAGC 13547

LsalLAC008020_C 13498 TTCCTTAACAAGCCCTCGAGCCTCCTTTGCGCCGCCTTCCTAATAGAAGC 13547

LsalCGN5271_CDS 13498 TTCCTTAACAAGCCCTCGAGCCTCCTTTGCGCCGCCTTCCTAATAGAAGC 13547

LsatLACWENDEL_C 13515 CGCCGAGTTGACCCCGAAGGCCGAATTCTATGGTAGAGAACGCTGTAATA 13564

LsatSalinas_CDS 13515 CGCCGAGTTGACCCCGAAGGCCGAATTCTATGGTAGAGAACGCTGTAATA 13564

LserUS96UC23_CD 13515 CGCCGAGTTGACCCCGAAGGCCGAATTCTATGGTAGAGAACGCTGTAATA 13564

LvirLAC006941_C 13530 CGCCGAGTTGACCCCGAAGGCCGAATTCTATGGTAGAGAACGCTGTAATA 13579

LserLAC005780_C 13548 CGCCGAGTTGACCCCGAAGGCCGAATTCTATGGTAGAGAACGCTGTAATA 13597

LsalLAC008020_C 13548 CGCCGAGTTGACCCCGAAGGCCGAATTCTATGGTAGAGAACGCTGTAATA 13597

LsalCGN5271_CDS 13548 CGCCGAGTTGACCCCGAAGGCCGAATTCTATGGTAGAGAACGCTGTAATA 13597

LsatLACWENDEL_C 13565 ATAATTGGGCCATGAGAGACTTTTTTAAGTATTGCAAAAGAAAGGGCCTG 13614

LsatSalinas_CDS 13565 ATAATTGGGCCATGAGAGACTTTTTTAAGTATTGCAAAAGAAAGGGCCTG 13614

LserUS96UC23_CD 13565 ATAATTGGGCCATGAGAGACTTTTTTAAGTATTGCAAAAGAAAGGGCCTG 13614

LvirLAC006941_C 13580 ATAATTGGGCCATGAGAGACTTTTTTAAGTATTGCAAAAGAAAGGGCCTG 13629

LserLAC005780_C 13598 ATAATTGGGCCATGAGAGACTTTTTTAAGTATTGCAAAAGAAAGGGCCTG 13647

LsalLAC008020_C 13598 ATAATTGGGCCATGAGAGACTTTTTTAAGTATTGCAAAAGAAAGGGCCTG 13647

LsalCGN5271_CDS 13598 ATAATTGGGCCATGAGAGACTTTTTTAAGTATTGCAAAAGAAAGGGCCTG 13647

LsatLACWENDEL_C 13615 CTGATAGAGCTGGGCGGGGAGGCGATACTAGTTATCAGGTCAGAGAGAGG 13664

LsatSalinas_CDS 13615 CTGATAGAGCTGGGCGGGGAGGCGATACTAGTTATCAGGTCAGAGAGAGG 13664

LserUS96UC23_CD 13615 CTGATAGAGCTGGGCGGGGAGGCGATACTAGTTATCAGGTCAGAGAGAGG 13664

LvirLAC006941_C 13630 CTGATAGAGCTGGGCGGGGAGGCGATACTAGTTATCAGGTCAGAGAGAGG 13679

LserLAC005780_C 13648 CTGATAGAGCTGGGCGGGGAGGCGATACTAGTTATCAGGTCAGAGAGAGG 13697

LsalLAC008020_C 13648 CTGATAGAGCTGGGCGGGGAGGCGATACTAGTTATCAGGTCAGAGAGAGG 13697

LsalCGN5271_CDS 13648 CTGATAGAGCTGGGCGGGGAGGCGATACTAGTTATCAGGTCAGAGAGAGG 13697

LsatLACWENDEL_C 13665 CCTGGCCCGTAAGCTGGCCCCCTTAAAAAGCCATTACTTAATAAGGATTT 13714

LsatSalinas_CDS 13665 CCTGGCCCGTAAGCTGGCCCCCTTAAAAAGCCATTACTTAATAAGGATTT 13714

LserUS96UC23_CD 13665 CCTGGCCCGTAAGCTGGCCCCCTTAAAAAGCCATTACTTAATAAGGATTT 13714

LvirLAC006941_C 13680 CCTGGCCCGTAAGCTGGCCCCCTTAAAAAGCCATTACTTAATAAGGATTT 13729

LserLAC005780_C 13698 CCTGGCCCGTAAGCTGGCCCCCTTAAAAAGCCATTACTTAATAAGGATTT 13747

LsalLAC008020_C 13698 CCTGGCCCGTAAGCTGGCCCCCTTAAAAAGCCATTACTTAATAAGGATTT 13747

LsalCGN5271_CDS 13698 CCTGGCCCGTAAGCTGGCCCCCTTAAAAAGCCATTACTTAATAAGGATTT 13747

LsatLACWENDEL_C 13715 GTTACGCGCGATATGCCGACGACTTACTACTGGGAATCGTGGGTGCCGTA 13764

LsatSalinas_CDS 13715 GTTACGCGCGATATGCCGACGACTTACTACTGGGAATCGTGGGTGCCGTA 13764

LserUS96UC23_CD 13715 GTTACGCGCGATATGCCGACGACTTACTACTGGGAATCGTGGGTGCCGTA 13764

LvirLAC006941_C 13730 GTTACGCGCGATATGCCGACGACTTACTACTGGGAATCGTGGGTGCCGTA 13779

LserLAC005780_C 13748 GTTACGCGCGATATGCCGACGACTTACTACTGGGAATCGTGGGTGCCGTA 13797

LsalLAC008020_C 13748 GTTACGCGCGATATGCCGACGACTTACTACTGGGAATCGTGGGTGCCGTA 13797

LsalCGN5271_CDS 13748 GTTACGCGCGATATGCCGACGACTTACTACTGGGAATCGTGGGTGCCGTA 13797

LsatLACWENDEL_C 13765 GAGCTTCTCATAGAAATAAAAAAACTTCTCGCCCACTTCCTACAATCCGG 13814

LsatSalinas_CDS 13765 GAGCTTCTCATAGAAATAAAAAAACTTCTCGCCCACTTCCTACAATCCGG 13814

LserUS96UC23_CD 13765 GAGCTTCTCATAGAAATAAAAAAACTTCTCGCCCACTTCCTACAATCCGG 13814

LvirLAC006941_C 13780 GAGCTTCTCATAGAAATAAAAAAACTTCTCGCCCACTTCCTACAATCCGG 13829

LserLAC005780_C 13798 GAGCTTCTCATAGAAATAAAAAAACTTCTCGCCCACTTCCTACAATCCGG 13847

LsalLAC008020_C 13798 GAGCTTCTCATAGAAATAAAAAAACTTCTCGCCCACTTCCTACAATCCGG 13847

LsalCGN5271_CDS 13798 GAGCTTCTCATAGAAATAAAAAAACTTCTCGCCCACTTCCTACAATCCGG 13847

LsatLACWENDEL_C 13815 CCTGAACCTTTGGGTAGGCTCTGCAGGATCAACAACAATAGCTGCACGGA 13864

LsatSalinas_CDS 13815 CCTGAACCTTTGGGTAGGCTCTGCAGGATCAACAACAATAGCTGCACGGA 13864

LserUS96UC23_CD 13815 CCTGAACCTTTGGGTAGGCTCTGCAGGATCAACAACAATAGCTGCACGGA 13864

LvirLAC006941_C 13830 CCTGAACCTTTGGGTAGGCTCTGCAGGATCAACAACAATAGCTGCACGGA 13879

LserLAC005780_C 13848 CCTGAACCTTTGGGTAGGCTCTGCAGGATCAACAACAATAGCTGCACGGA 13897

LsalLAC008020_C 13848 CCTGAACCTTTGGGTAGGCTCTGCAGGATCAACAACAATAGCTGCACGGA 13897

LsalCGN5271_CDS 13848 CCTGAACCTTTGGGTAGGCTCTGCAGGATCAACAACAATAGCTGCACGGA 13897

LsatLACWENDEL_C 13865 GTACGGTAGAATTCCTCGGTACGGTCATTCGGGAAGTCCCTCCGAGGACG 13914

LsatSalinas_CDS 13865 GTACGGTAGAATTCCTCGGTACGGTCATTCGGGAAGTCCCTCCGAGGACG 13914

LserUS96UC23_CD 13865 GTACGGTAGAATTCCTCGGTACGGTCATTCGGGAAGTCCCTCCGAGGACG 13914

LvirLAC006941_C 13880 GTACGGTAGAATTCCTCGGTACGGTCATTCGGGAAGTCCCTCCGAGGACG 13929

LserLAC005780_C 13898 GTACGGTAGAATTCCTCGGTACGGTCATTCGGGAAGTCCCTCCGAGGACG 13947

LsalLAC008020_C 13898 GTACGGTAGAATTCCTCGGTACGGTCATTCGGGAAGTCCCTCCGAGGACG 13947

LsalCGN5271_CDS 13898 GTACGGTAGAATTCCTCGGTACGGTCATTCGGGAAGTCCCTCCGAGGACG 13947

LsatLACWENDEL_C 13915 ACTCCCATACAATTCTTGCGAGAGCTGGAGAAGCGTCTACGAGTAAAGCA 13964

LsatSalinas_CDS 13915 ACTCCCATACAATTCTTGCGAGAGCTGGAGAAGCGTCTACGAGTAAAGCA 13964

LserUS96UC23_CD 13915 ACTCCCATACAATTCTTGCGAGAGCTGGAGAAGCGTCTACGAGTAAAGCA 13964

LvirLAC006941_C 13930 ACTCCCATACAATTCTTGCGAGAGCTGGAGAAGCGTCTACGAGTAAAGCA 13979

LserLAC005780_C 13948 ACTCCCATACAATTCTTGCGAGAGCTGGAGAAGCGTCTACGAGTAAAGCA 13997

LsalLAC008020_C 13948 ACTCCCATACAATTCTTGCGAGAGCTGGAGAAGCGTCTACGAGTAAAGCA 13997

LsalCGN5271_CDS 13948 ACTCCCATACAATTCTTGCGAGAGCTGGAGAAGCGTCTACGAGTAAAGCA 13997

LsatLACWENDEL_C 13965 CCGTATACATATAACTGCTTGCCACCTACGCTCCGCCATCCATTCCAAGT 14014

LsatSalinas_CDS 13965 CCGTATACATATAACTGCTTGCCACCTACGCTCCGCCATCCATTCCAAGT 14014

LserUS96UC23_CD 13965 CCGTATACATATAACTGCTTGCCACCTACGCTCCGCCATCCATTCCAAGT 14014

LvirLAC006941_C 13980 CCGTATACATATAACTGCTTGCCACCTACGCTCCGCCATCCATTCCAAGT 14029

LserLAC005780_C 13998 CCGTATACATATAACTGCTTGCCACCTACGCTCCGCCATCCATTCCAAGT 14047

LsalLAC008020_C 13998 CCGTATACATATAACTGCTTGCCACCTACGCTCCGCCATCCATTCCAAGT 14047

LsalCGN5271_CDS 13998 CCGTATACATATAACTGCTTGCCACCTACGCTCCGCCATCCATTCCAAGT 14047

LsatLACWENDEL_C 14015 TTAGGAACCTAGGTAATAGTATCCCGATCAAAGAGCTGACGAAGGGGATG 14064

LsatSalinas_CDS 14015 TTAGGAACCTAGGTAATAGTATCCCGATCAAAGAGCTGACGAAGGGGATG 14064

LserUS96UC23_CD 14015 TTAGGAACCTAGGTAATAGTATCCCGATCAAAGAGCTGACGAAGGGGATG 14064

LvirLAC006941_C 14030 TTAGGAACCTAGGTAATAGTATCCCGATCAAAGAGCTGACGAAGGGGATG 14079

LserLAC005780_C 14048 TTAGGAACCTAGGTAATAGTATCCCGATCAAAGAGCTGACGAAGGGGATG 14097

LsalLAC008020_C 14048 TTAGGAACCTAGGTAATAGTATCCCGATCAAAGAGCTGACGAAGGGGATG 14097

LsalCGN5271_CDS 14048 TTAGGAACCTAGGTAATAGTATCCCGATCAAAGAGCTGACGAAGGGGATG 14097

LsatLACWENDEL_C 14065 AGCGGAACAGGGAGTCTACTGGACGCGGTTCAACTAGCGGACACTCTTGG 14114

LsatSalinas_CDS 14065 AGCGGAACAGGGAGTCTACTGGACGCGGTTCAACTAGCGGACACTCTTGG 14114

LserUS96UC23_CD 14065 AGCGGAACAGGGAGTCTACTGGACGCGGTTCAACTAGCGGACACTCTTGG 14114

LvirLAC006941_C 14080 AGCGGAACAGGGAGTCTACTGGACGCGGTTCAACTAGCGGACACTCTTGG 14129

LserLAC005780_C 14098 AGCGGAACAGGGAGTCTACTGGACGCGGTTCAACTAGCGGACACTCTTGG 14147

LsalLAC008020_C 14098 AGCGGAACAGGGAGTCTACTGGACGCGGTTCAACTAGCGGACACTCTTGG 14147

LsalCGN5271_CDS 14098 AGCGGAACAGGGAGTCTACTGGACGCGGTTCAACTAGCGGACACTCTTGG 14147

LsatLACWENDEL_C 14115 AACAGCTGGAGTAAGAAGTCCCCAAGTGAGCGTATTATGGGGGACCGTCA 14164

LsatSalinas_CDS 14115 AACAGCTGGAGTAAGAAGTCCCCAAGTGAGCGTATTATGGGGGACCGTCA 14164

LserUS96UC23_CD 14115 AACAGCTGGAGTAAGAAGTCCCCAAGTGAGCGTATTATGGGGGACCGTCA 14164

LvirLAC006941_C 14130 AACAGCTGGAGTAAGAAGTCCCCAAGTGAGCGTATTATGGGGGACCGTCA 14179

LserLAC005780_C 14148 AACAGCTGGAGTAAGAAGTCCCCAAGTGAGCGTATTATGGGGGACCGTCA 14197

LsalLAC008020_C 14148 AACAGCTGGAGTAAGAAGTCCCCAAGTGAGCGTATTATGGGGGACCGTCA 14197

LsalCGN5271_CDS 14148 AACAGCTGGAGTAAGAAGTCCCCAAGTGAGCGTATTATGGGGGACCGTCA 14197

LsatLACWENDEL_C 14165 AGCACATCCGGCAAGGATCAAGGGCGATCTCGTTGTTGCATAGCTCAGGT 14214

LsatSalinas_CDS 14165 AGCACATCCGGCAAGGATCAAGGGCGATCTCGTTGTTGCATAGCTCAGGT 14214

LserUS96UC23_CD 14165 AGCACATCCGGCAAGGATCAAGGGCGATCTCGTTGTTGCATAGCTCAGGT 14214

LvirLAC006941_C 14180 AGCACATCCGGCAAGGATCAAGGGCGATCTCGTTGTTGCATAGCTCAGGT 14229

LserLAC005780_C 14198 AGCACATCCGGCAAGGATCAAGGGCGATCTCGTTGTTGCATAGCTCAGGT 14247

LsalLAC008020_C 14198 AGCACATCCGGCAAGGATCAAGGGCGATCTCGTTGTTGCATAGCTCAGGT 14247

LsalCGN5271_CDS 14198 AGCACATCCGGCAAGGATCAAGGGCGATCTCGTTGTTGCATAGCTCAGGT 14247

LsatLACWENDEL_C 14215 CGGAGTAAGGTGCCATCGGACGTCCAACAGGCAGTCTCACGATCGGGCAT 14264

LsatSalinas_CDS 14215 CGGAGTAAGGTGCCATCGGACGTCCAACAGGCAGTCTCACGATCGGGCAT 14264

LserUS96UC23_CD 14215 CGGAGTAAGGTGCCATCGGACGTCCAACAGGCAGTCTCACGATCGGGCAT 14264

LvirLAC006941_C 14230 CGGAGTAAGGTGCCATCGGACGTCCAACAGGCAGTCTCACGATCGGGCAT 14279

LserLAC005780_C 14248 CGGAGTAAGGTGCCATCGGACGTCCAACAGGCAGTCTCACGATCGGGCAT 14297

LsalLAC008020_C 14248 CGGAGTAAGGTGCCATCGGACGTCCAACAGGCAGTCTCACGATCGGGCAT 14297

LsalCGN5271_CDS 14248 CGGAGTAAGGTGCCATCGGACGTCCAACAGGCAGTCTCACGATCGGGCAT 14297

LsatLACWENDEL_C 14265 GAGTGTCCGGAAGTTGTCATTGTATACTCCCGCGGGTCGGAAGGCGGCGG 14314

LsatSalinas_CDS 14265 GAGTGTCCGGAAGTTGTCATTGTATACTCCCGCGGGTCGGAAGGCGGCGG 14314

LserUS96UC23_CD 14265 GAGTGTCCGGAAGTTGTCATTGTATACTCCCGCGGGTCGGAAGGCGGCGG 14314

LvirLAC006941_C 14280 GAGTGTCCGGAAGTTGTCATTGTATACTCCCGCGGGTCGGAAGGCGGCGG 14329

LserLAC005780_C 14298 GAGTGTCCGGAAGTTGTCATTGTATACTCCCGCGGGTCGGAAGGCGGCGG 14347

LsalLAC008020_C 14298 GAGTGTCCGGAAGTTGTCATTGTATACTCCCGCGGGTCGGAAGGCGGCGG 14347

LsalCGN5271_CDS 14298 GAGTGTCCGGAAGTTGTCATTGTATACTCCCGCGGGTCGGAAGGCGGCGG 14347

LsatLACWENDEL_C 14315 GGGAAGGAGAGGGAGACTGGGCGAGATCTATCAGAAGGGAATTCCCCATA 14364

LsatSalinas_CDS 14315 GGGAAGGAGAGGGAGACTGGGCGAGATCTATCAGAAGGGAATTCCCCATA 14364

LserUS96UC23_CD 14315 GGGAAGGAGAGGGAGACTGGGCGAGATCTATCAGAAGGGAATTCCCCATA 14364

LvirLAC006941_C 14330 GGGAAGGAGAGGGAGACTGGGCGAGATCTATCAGAAGGGAATTCCCCATA 14379

LserLAC005780_C 14348 GGGAAGGAGAGGGAGACTGGGCGAGATCTATCAGAAGGGAATTCCCCATA 14397

LsalLAC008020_C 14348 GGGAAGGAGAGGGAGACTGGGCGAGATCTATCAGAAGGGAATTCCCCATA 14397

LsalCGN5271_CDS 14348 GGGAAGGAGAGGGAGACTGGGCGAGATCTATCAGAAGGGAATTCCCCATA 14397

LsatLACWENDEL_C 14365 CAAATAGAGGCGCCTATCAAAAAGATACTCCGAAGGCTTCGGGATCGAGG 14414

LsatSalinas_CDS 14365 CAAATAGAGGCGCCTATCAAAAAGATACTCCGAAGGCTTCGGGATCGAGG 14414

LserUS96UC23_CD 14365 CAAATAGAGGCGCCTATCAAAAAGATACTCCGAAGGCTTCGGGATCGAGG 14414

LvirLAC006941_C 14380 CAAATAGAGGCGCCTATCAAAAAGATACTCCGAAGGCTTCGGGATCGAGG 14429

LserLAC005780_C 14398 CAAATAGAGGCGCCTATCAAAAAGATACTCCGAAGGCTTCGGGATCGAGG 14447

LsalLAC008020_C 14398 CAAATAGAGGCGCCTATCAAAAAGATACTCCGAAGGCTTCGGGATCGAGG 14447

LsalCGN5271_CDS 14398 CAAATAGAGGCGCCTATCAAAAAGATACTCCGAAGGCTTCGGGATCGAGG 14447

LsatLACWENDEL_C 14415 TCTCATTAGCCGAAGAAGACCCTGGCCAATCCACGTGGCCTGCTTGACGA 14464

LsatSalinas_CDS 14415 TCTCATTAGCCGAAGAAGACCCTGGCCAATCCACGTGGCCTGCTTGACGA 14464

LserUS96UC23_CD 14415 TCTCATTAGCCGAAGAAGACCCTGGCCAATCCACGTGGCCTGCTTGACGA 14464

LvirLAC006941_C 14430 TCTCATTAGCCGAAGAAGACCCTGGCCAATCCACGTGGCCTGCTTGACGA 14479

LserLAC005780_C 14448 TCTCATTAGCCGAAGAAGACCCTGGCCAATCCACGTGGCCTGCTTGACGA 14497

LsalLAC008020_C 14448 TCTCATTAGCCGAAGAAGACCCTGGCCAATCCACGTGGCCTGCTTGACGA 14497

LsalCGN5271_CDS 14448 TCTCATTAGCCGAAGAAGACCCTGGCCAATCCACGTGGCCTGCTTGACGA 14497

LsatLACWENDEL_C 14465 ACGTCAGCGACGGAGACATCGTAAATTGGTCCGCGGGCATCGCGATAAGT 14514

LsatSalinas_CDS 14465 ACGTCAGCGACGGAGACATCGTAAATTGGTCCGCGGGCATCGCGATAAGT 14514

LserUS96UC23_CD 14465 ACGTCAGCGACGGAGACATCGTAAATTGGTCCGCGGGCATCGCGATAAGT 14514

LvirLAC006941_C 14480 ACGTCAGCGACGGAGACATCGTAAATTGGTCCGCGGGCATCGCGATAAGT 14529

LserLAC005780_C 14498 ACGTCAGCGACGGAGACATCGTAAATTGGTCCGCGGGCATCGCGATAAGT 14547

LsalLAC008020_C 14498 ACGTCAGCGACGGAGACATCGTAAATTGGTCCGCGGGCATCGCGATAAGT 14547

LsalCGN5271_CDS 14498 ACGTCAGCGACGGAGACATCGTAAATTGGTCCGCGGGCATCGCGATAAGT 14547

LsatLACWENDEL_C 14515 CCTCTGTCCTACTACAGGTGCCGCGACAACCTTTACCAAGTCCGAACGAT 14564

LsatSalinas_CDS 14515 CCTCTGTCCTACTACAGGTGCCGCGACAACCTTTACCAAGTCCGAACGAT 14564

LserUS96UC23_CD 14515 CCTCTGTCCTACTACAGGTGCCGCGACAACCTTTACCAAGTCCGAACGAT 14564

LvirLAC006941_C 14530 CCTCTGTCCTACTACAGGTGCCGCGACAACCTTTACCAAGTCCGAACGAT 14579

LserLAC005780_C 14548 CCTCTGTCCTACTACAGGTGCCGCGACAACCTTTACCAAGTCCGAACGAT 14597

LsalLAC008020_C 14548 CCTCTGTCCTACTACAGGTGCCGCGACAACCTTTACCAAGTCCGAACGAT 14597

LsalCGN5271_CDS 14548 CCTCTGTCCTACTACAGGTGCCGCGACAACCTTTACCAAGTCCGAACGAT 14597

LsatLACWENDEL_C 14565 TGTCGACCACCAGATCCGCTGGTCTGCAATATTCACCCCGGCCCACAAGC 14614

LsatSalinas_CDS 14565 TGTCGACCACCAGATCCGCTGGTCTGCAATATTCACCCCGGCCCACAAGC 14614

LserUS96UC23_CD 14565 TGTCGACCACCAGATCCGCTGGTCTGCAATATTCACCCCGGCCCACAAGC 14614

LvirLAC006941_C 14580 TGTCGACCACCAGATCCGCTGGTCTGCAATATTCACCCCGGCCCACAAGC 14629

LserLAC005780_C 14598 TGTCGACCACCAGATCCGCTGGTCTGCAATATTCACCCCGGCCCACAAGC 14647

LsalLAC008020_C 14598 TGTCGACCACCAGATCCGCTGGTCTGCAATATTCACCCCGGCCCACAAGC 14647

LsalCGN5271_CDS 14598 TGTCGACCACCAGATCCGCTGGTCTGCAATATTCACCCCGGCCCACAAGC 14647

LsatLACWENDEL_C 14615 ACAAATCCTCGGCGCGGAATATAATCCCAAAGTACTCCAAAGACTCAAAT 14664

LsatSalinas_CDS 14615 ACAAATCCTCGGCGCGGAATATAATCCCAAAGTACTCCAAAGACTCAAAT 14664

LserUS96UC23_CD 14615 ACAAATCCTCGGCGCGGAATATAATCCCAAAGTACTCCAAAGACTCAAAT 14664

LvirLAC006941_C 14630 ACAAATCCTCGGCGCGGAATATAATCCCAAAGTACTCCAAAGACTCAAAT 14679

LserLAC005780_C 14648 ACAAATCCTCGGCGCGGAATATAATCCCAAAGTACTCCAAAGACTCAAAT 14697

LsalLAC008020_C 14648 ACAAATCCTCGGCGCGGAATATAATCCCAAAGTACTCCAAAGACTCAAAT 14697

LsalCGN5271_CDS 14648 ACAAATCCTCGGCGCGGAATATAATCCCAAAGTACTCCAAAGACTCAAAT 14697

LsatLACWENDEL_C 14665 ATAGTAAATAAAGAAGGTGGTAAGACCCTTGCAGAGTTCCCCAACAGCAT 14714

LsatSalinas_CDS 14665 ATAGTAAATAAAGAAGGTGGTAAGACCCTTGCAGAGTTCCCCAACAGCAT 14714

LserUS96UC23_CD 14665 ATAGTAAATAAAGAAGGTGGTAAGACCCTTGCAGAGTTCCCCAACAGCAT 14714

LvirLAC006941_C 14680 ATAGTAAATAAAGAAGGTGGTAAGACCCTTGCAGAGTTCCCCAACAGCAT 14729

LserLAC005780_C 14698 ATAGTAAATAAAGAAGGTGGTAAGACCCTTGCAGAGTTCCCCAACAGCAT 14747

LsalLAC008020_C 14698 ATAGTAAATAAAGAAGGTGGTAAGACCCTTGCAGAGTTCCCCAACAGCAT 14747

LsalCGN5271_CDS 14698 ATAGTAAATAAAGAAGGTGGTAAGACCCTTGCAGAGTTCCCCAACAGCAT 14747

LsatLACWENDEL_C 14715 AGAGCTTGGGAAGCTCGGACCCGGTCAAGATCCGAACAACAAGGAGCACT 14764

LsatSalinas_CDS 14715 AGAGCTTGGGAAGCTCGGACCCGGTCAAGATCCGAACAACAAGGAGCACT 14764

LserUS96UC23_CD 14715 AGAGCTTGGGAAGCTCGGACCCGGTCAAGATCCGAACAACAAGGAGCACT 14764

LvirLAC006941_C 14730 AGAGCTTGGGAAGCTCGGACCCGGTCAAGATCCGAACAACAAGGAGCACT 14779

LserLAC005780_C 14748 AGAGCTTGGGAAGCTCGGACCCGGTCAAGATCCGAACAACAAGGAGCACT 14797

LsalLAC008020_C 14748 AGAGCTTGGGAAGCTCGGACCCGGTCAAGATCCGAACAACAAGGAGCACT 14797

LsalCGN5271_CDS 14748 AGAGCTTGGGAAGCTCGGACCCGGTCAAGATCCGAACAACAAGGAGCACT 14797

LsatLACWENDEL_C 14765 CAACTACTAGTCTAGTCTAGATTGTGATACATAATCAATCCTATTTGTTT 14814

LsatSalinas_CDS 14765 CAACTACTAGTCTAGTCTAGATTGTGATACATAATCAATCCTATTTGTTT 14814

LserUS96UC23_CD 14765 CAACTACTAGTCTAGTCTAGATTGTGATACATAATCAATCCTATTTGTTT 14814

LvirLAC006941_C 14780 CAACTACTAGTCTAGTCTAGATTGTGATACATAATCAATCCTATTTGTTT 14829

LserLAC005780_C 14798 CAACTACTAGTCTAGTCTAGATTGTGATACATAATCAATCCTATTTGTTT 14847

LsalLAC008020_C 14798 CAACTACTAGTCTAGTCTAGATTGTGATACATAATCAATCCTATTTGTTT 14847

LsalCGN5271_CDS 14798 CAACTACTAGTCTAGTCTAGATTGTGATACATAATCAATCCTATTTGTTT 14847

LsatLACWENDEL_C 14815 TGTTCATTACTAAAGAATCCTATAAAATCTTTCTCTTTATCCTTTGAATT 14864

LsatSalinas_CDS 14815 TGTTCATTACTAAAGAATCCTATAAAATCTTTCTCTTTATCCTTTGAATT 14864

LserUS96UC23_CD 14815 TGTTCATTACTAAAGAATCCTATAAAATCTTTCTCTTTATCCTTTGAATT 14864

LvirLAC006941_C 14830 TGTTCATTACTAAAGAATCCTATAAAATCTTTCTCTTTATCCTTTGAATT 14879

LserLAC005780_C 14848 TGTTCATTACTAAAGAATCCTATAAAATCTTTCTCTTTATCCTTTGAATT 14897

LsalLAC008020_C 14848 TGTTCATTACTAAAGAATCCTATAAAATCTTTCTCTTTATCCTTTGAATT 14897

LsalCGN5271_CDS 14848 TGTTCATTACTAAAGAATCCTATAAAATCTTTCTCTTTATCCTTTGAATT 14897

LsatLACWENDEL_C 14865 ACTCATATATATATCCTATGAATTTCATTTCGCACCGGAAACTATTCTAG 14914

LsatSalinas_CDS 14865 ACTCATATATATATCCTATGAATTTCATTTCGCACCGGAAACTATTCTAG 14914

LserUS96UC23_CD 14865 ACTCATATATATATCCTATGAATTTCATTTCGCACCGGAAACTATTCTAG 14914

LvirLAC006941_C 14880 ACTCATATATATATCCTATGAATTTCATTTCGCACCGGAAACTATTCTAG 14929

LserLAC005780_C 14898 ACTCATATATATATCCTATGAATTTCATTTCGCACCGGAAACTATTCTAG 14947

LsalLAC008020_C 14898 ACTCATATATATATCCTATGAATTTCATTTCGCACCGGAAACTATTCTAG 14947

LsalCGN5271_CDS 14898 ACTCATATATATATCCTATGAATTTCATTTCGCACCGGAAACTATTCTAG 14947

LsatLACWENDEL_C 14915 GAGAAGTTCGAATCCGTTCCGTTCGGATATTGATCGGTCTTGGTTTGACA 14964

LsatSalinas_CDS 14915 GAGAAGTTCGAATCCGTTCCGTTCGGATATTGATCGGTCTTGGTTTGACA 14964

LserUS96UC23_CD 14915 GAGAAGTTCGAATCCGTTCCGTTCGGATATTGATCGGTCTTGGTTTGACA 14964

LvirLAC006941_C 14930 GAGAAGTTCGAATCCGTTCCGTTCGGATATTGATCGGTCTTGGTTTGACA 14979

LserLAC005780_C 14948 GAGAAGTTCGAATCCGTTCCGTTCGGATATTGATCGGTCTTGGTTTGACA 14997

LsalLAC008020_C 14948 GAGAAGTTCGAATCCGTTCCGTTCGGATATTGATCGGTCTTGGTTTGACA 14997

LsalCGN5271_CDS 14948 GAGAAGTTCGAATCCGTTCCGTTCGGATATTGATCGGTCTTGGTTTGACA 14997

LsatLACWENDEL_C 14965 TGGTTTACGCGTTACTGGTTCCCGGAAGAGTTAATATCTCCATTAGCTAA 15014

LsatSalinas_CDS 14965 TGGTTTACGCGTTACTGGTTCCCGGAAGAGTTAATATCTCCATTAGCTAA 15014

LserUS96UC23_CD 14965 TGGTTTACGCGTTACTGGTTCCCGGAAGAGTTAATATCTCCATTAGCTAA 15014

LvirLAC006941_C 14980 TGGTTTACGCGTTACTGGTTCCCGGAAGAGTTAATATCTCCATTAGCTAA 15029

LserLAC005780_C 14998 TGGTTTACGCGTTACTGGTTCCCGGAAGAGTTAATATCTCCATTAGCTAA 15047

LsalLAC008020_C 14998 TGGTTTACGCGTTACTGGTTCCCGGAAGAGTTAATATCTCCATTAGCTAA 15047

LsalCGN5271_CDS 14998 TGGTTTACGCGTTACTGGTTCCCGGAAGAGTTAATATCTCCATTAGCTAA 15047

LsatLACWENDEL_C 15015 ACCCTTTCTTATTCTTACCTTGCCTTTGGACTCGTATTTTGTTCGTACAC 15064

LsatSalinas_CDS 15015 ACCCTTTCTTATTCTTACCTTGCCTTTGGACTCGTATTTTGTTCGTACAC 15064

LserUS96UC23_CD 15015 ACCCTTTCTTATTCTTACCTTGCCTTTGGACTCGTATTTTGTTCGTACAC 15064

LvirLAC006941_C 15030 ACCCTTTCTTATTCTTACCTTGCCTTTGGACTCGTATTTTGTTCGTACAC 15079

LserLAC005780_C 15048 ACCCTTTCTTATTCTTACCTTGCCTTTGGACTCGTATTTTGTTCGTACAC 15097

LsalLAC008020_C 15048 ACCCTTTCTTATTCTTACCTTGCCTTTGGACTCGTATTTTGTTCGTACAC 15097

LsalCGN5271_CDS 15048 ACCCTTTCTTATTCTTACCTTGCCTTTGGACTCGTATTTTGTTCGTACAC 15097

LsatLACWENDEL_C 15065 AATCAACGGAGGCCTCCCCGACATATCTTGCAACGTCTTCAATAGCATGC 15114

LsatSalinas_CDS 15065 AATCAACGGAGGCCTCCCCGACATATCTTGCAACGTCTTCAATAGCATGC 15114

LserUS96UC23_CD 15065 AATCAACGGAGGCCTCCCCGACATATCTTGCAACGTCTTCAATAGCATGC 15114

LvirLAC006941_C 15080 AATCAACGGAGGCCTCCCCGACATATCTTGCAACGTCTTCAATAGCATGC 15129

LserLAC005780_C 15098 AATCAACGGAGGCCTCCCCGACATATCTTGCAACGTCTTCAATAGCATGC 15147

LsalLAC008020_C 15098 AATCAACGGAGGCCTCCCCGACATATCTTGCAACGTCTTCAATAGCATGC 15147

LsalCGN5271_CDS 15098 AATCAACGGAGGCCTCCCCGACATATCTTGCAACGTCTTCAATAGCATGC 15147

LsatLACWENDEL_C 15115 TCTTACTTCGTCTTTCCCTTAATAAGTCATCAAATTTGGTGCTTTTTGAT 15164

LsatSalinas_CDS 15115 TCTTACTTCGTCTTTCCCTTAATAAGTCATCAAATTTGGTGCTTTTTGAT 15164

LserUS96UC23_CD 15115 TCTTACTTCGTCTTTCCCTTAATAAGTCATCAAATTTGGTGCTTTTTGAT 15164

LvirLAC006941_C 15130 TCTTACTTCGTCTTTCCCTTAATAAGTCATCAAATTTGGTGCTTTTTGAT 15179

LserLAC005780_C 15148 TCTTACTTCGTCTTTCCCTTAATAAGTCATCAAATTTGGTGCTTTTTGAT 15197

LsalLAC008020_C 15148 TCTTACTTCGTCTTTCCCTTAATAAGTCATCAAATTTGGTGCTTTTTGAT 15197

LsalCGN5271_CDS 15148 TCTTACTTCGTCTTTCCCTTAATAAGTCATCAAATTTGGTGCTTTTTGAT 15197

LsatLACWENDEL_C 15165 CCCCAGTTGCTATGGGGAACAAAGGACGAAATACAATCGATTCCTCCATT 15214

LsatSalinas_CDS 15165 CCCCAGTTGCTATGGGGAACAAAGGACGAAATACAATCGATTCCTCCATT 15214

LserUS96UC23_CD 15165 CCCCAGTTGCTATGGGGAACAAAGGACGAAATACAATCGATTCCTCCATT 15214

LvirLAC006941_C 15180 CCCCAGTTGCTATGGGGAACAAAGGACGAAATACAATCGATTCCTCCATT 15229

LserLAC005780_C 15198 CCCCAGTTGCTATGGGGAACAAAGGACGAAATACAATCGATTCCTCCATT 15247

LsalLAC008020_C 15198 CCCCAGTTGCTATGGGGAACAAAGGACGAAATACAATCGATTCCTCCATT 15247

LsalCGN5271_CDS 15198 CCCCAGTTGCTATGGGGAACAAAGGACGAAATACAATCGATTCCTCCATT 15247

LsatLACWENDEL_C 15215 TAAGTGGTTCTCGCTTCTCCTTGTTCCTGTTCCTAACTCCTCCCCGGGTA 15264

LsatSalinas_CDS 15215 TAAGTGGTTCTCGCTTCTCCTTGTTCCTGTTCCTAACTCCTCCCCGGGTA 15264

LserUS96UC23_CD 15215 TAAGTGGTTCTCGCTTCTCCTTGTTCCTGTTCCTAACTCCTCCCCGGGTA 15264

LvirLAC006941_C 15230 TAAGTGGTTCTCGCTTCTCCTTGTTCCTGTTCCTAACTCCTCCCCGGGTA 15279

LserLAC005780_C 15248 TAAGTGGTTCTCGCTTCTCCTTGTTCCTGTTCCTAACTCCTCCCCGGGTA 15297

LsalLAC008020_C 15248 TAAGTGGTTCTCGCTTCTCCTTGTTCCTGTTCCTAACTCCTCCCCGGGTA 15297

LsalCGN5271_CDS 15248 TAAGTGGTTCTCGCTTCTCCTTGTTCCTGTTCCTAACTCCTCCCCGGGTA 15297

LsatLACWENDEL_C 15265 GTTCCCAATGTTTGGCACTTTCCATACTTGATGGGTGCAACATCAACAAA 15314

LsatSalinas_CDS 15265 GTTCCCAATGTTTGGCACTTTCCATACTTGATGGGTGCAACATCAACAAA 15314

LserUS96UC23_CD 15265 GTTCCCAATGTTTGGCACTTTCCATACTTGATGGGTGCAACATCAACAAA 15314

LvirLAC006941_C 15280 GTTCCCAATGTTTGGCACTTTCCATACTTGATGGGTGCAACATCAACAAA 15329

LserLAC005780_C 15298 GTTCCCAATGTTTGGCACTTTCCATACTTGATGGGTGCAACATCAACAAA 15347

LsalLAC008020_C 15298 GTTCCCAATGTTTGGCACTTTCCATACTTGATGGGTGCAACATCAACAAA 15347

LsalCGN5271_CDS 15298 GTTCCCAATGTTTGGCACTTTCCATACTTGATGGGTGCAACATCAACAAA 15347

LsatLACWENDEL_C 15315 TTCGCTCATGATCAAGTTACAACCTAAGATCTATGACCATATTATGTTAA 15364

LsatSalinas_CDS 15315 TTCGCTCATGATCAAGTTACAACCTAAGATCTATGACCATATTATGTTAA 15364

LserUS96UC23_CD 15315 TTCGCTCATGATCAAGTTACAACCTAAGATCTATGACCATATTATGTTAA 15364

LvirLAC006941_C 15330 TTCGCTCATGATCAAGTTACAACCTAAGATCTATGACCATATTATGTTAA 15379

LserLAC005780_C 15348 TTCGCTCATGATCAAGTTACAACCTAAGATCTATGACCATATTATGTTAA 15397

LsalLAC008020_C 15348 TTCGCTCATGATCAAGTTACAACCTAAGATCTATGACCATATTATGTTAA 15397

LsalCGN5271_CDS 15348 TTCGCTCATGATCAAGTTACAACCTAAGATCTATGACCATATTATGTTAA 15397

LsatLACWENDEL_C 15365 CTGTTCGTATTTCGTTCATTCCATCGGTATGCTCCCAGGTACCTGTAATT 15414

LsatSalinas_CDS 15365 CTGTTCGTATTTCGTTCATTCCATCGGTATGCTCCCAGGTACCTGTAATT 15414

LserUS96UC23_CD 15365 CTGTTCGTATTTCGTTCATTCCATCGGTATGCTCCCAGGTACCTGTAATT 15414

LvirLAC006941_C 15380 CTGTTCGTATTTCGTTCATTCCATCGGTATGCTCCCAGGTACCTGTAATT 15429

LserLAC005780_C 15398 CTGTTCGTATTTCGTTCATTCCATCGGTATGCTCCCAGGTACCTGTAATT 15447

LsalLAC008020_C 15398 CTGTTCGTATTTCGTTCATTCCATCGGTATGCTCCCAGGTACCTGTAATT 15447

LsalCGN5271_CDS 15398 CTGTTCGTATTTCGTTCATTCCATCGGTATGCTCCCAGGTACCTGTAATT 15447

LsatLACWENDEL_C 15415 GTGATCCGTTTGCCAGAACCAAGGGGTCTTTCTGTGGAAACCTCCACGAA 15464

LsatSalinas_CDS 15415 GTGATCCGTTTGCCAGAACCAAGGGGTCTTTCTGTGGAAACCTCCACGAA 15464

LserUS96UC23_CD 15415 GTGATCCGTTTGCCAGAACCAAGGGGTCTTTCTGTGGAAACCTCCACGAA 15464

LvirLAC006941_C 15430 GTGATCCGTTTGCCAGAACCAAGGGGTCTTTCTGTGGAAACCTCCACGAA 15479

LserLAC005780_C 15448 GTGATCCGTTTGCCAGAACCAAGGGGTCTTTCTGTGGAAACCTCCACGAA 15497

LsalLAC008020_C 15448 GTGATCCGTTTGCCAGAACCAAGGGGTCTTTCTGTGGAAACCTCCACGAA 15497

LsalCGN5271_CDS 15448 GTGATCCGTTTGCCAGAACCAAGGGGTCTTTCTGTGGAAACCTCCACGAA 15497

LsatLACWENDEL_C 15465 CAATCGTCGTTTTTTGATGGTTTTTCCGCTTCTCACAGCTGCTCTTTCCA 15514

LsatSalinas_CDS 15465 CAATCGTCGTTTTTTGATGGTTTTTCCGCTTCTCACAGCTGCTCTTTCCA 15514

LserUS96UC23_CD 15465 CAATCGTCGTTTTTTGATGGTTTTTCCGCTTCTCACAGCTGCTCTTTCCA 15514

LvirLAC006941_C 15480 CAATCGTCGTTTTTTGATGGTTTTTCCGCTTCTCACAGCTGCTCTTTCCA 15529

LserLAC005780_C 15498 CAATCGTCGTTTTTTGATGGTTTTTCCGCTTCTCACAGCTGCTCTTTCCA 15547

LsalLAC008020_C 15498 CAATCGTCGTTTTTTGATGGTTTTTCCGCTTCTCACAGCTGCTCTTTCCA 15547

LsalCGN5271_CDS 15498 CAATCGTCGTTTTTTGATGGTTTTTCCGCTTCTCACAGCTGCTCTTTCCA 15547

LsatLACWENDEL_C 15515 CACCTCCGGATATCTGGTGCCAAATCGTCGCCCCTTTCCTTATTTTTTCG 15564

LsatSalinas_CDS 15515 CACCTCCGGATATCTGGTGCCAAATCGTCGCCCCTTTCCTTATTTTTTCG 15564

LserUS96UC23_CD 15515 CACCTCCGGATATCTGGTGCCAAATCGTCGCCCCTTTCCTTATTTTTTCG 15564

LvirLAC006941_C 15530 CACCTCCGGATATCTGGTGCCAAATCGTCGCCCCTTTCCTTATTTTTTCG 15579

LserLAC005780_C 15548 CACCTCCGGATATCTGGTGCCAAATCGTCGCCCCTTTCCTTATTTTTTCG 15597

LsalLAC008020_C 15548 CACCTCCGGATATCTGGTGCCAAATCGTCGCCCCTTTCCTTATTTTTTCG 15597

LsalCGN5271_CDS 15548 CACCTCCGGATATCTGGTGCCAAATCGTCGCCCCTTTCCTTATTTTTTCG 15597

LsatLACWENDEL_C 15565 ATAATAGAGTTGGCTATCTCTGTGGCATCGATTGTACAAGTTCGTGAAGA 15614

LsatSalinas_CDS 15565 ATAATAGAGTTGGCTATCTCTGTGGCATCGATTGTACAAGTTCGTGAAGA 15614

LserUS96UC23_CD 15565 ATAATAGAGTTGGCTATCTCTGTGGCATCGATTGTACAAGTTCGTGAAGA 15614

LvirLAC006941_C 15580 ATAATAGAGTTGGCTATCTCTGTGGCATCGATTGTACAAGTTCGTGAAGA 15629

LserLAC005780_C 15598 ATAATAGAGTTGGCTATCTCTGTGGCATCGATTGTACAAGTTCGTGAAGA 15647

LsalLAC008020_C 15598 ATAATAGAGTTGGCTATCTCTGTGGCATCGATTGTACAAGTTCGTGAAGA 15647

LsalCGN5271_CDS 15598 ATAATAGAGTTGGCTATCTCTGTGGCATCGATTGTACAAGTTCGTGAAGA 15647

LsatLACWENDEL_C 15615 GGGCTGGACGAGTGGAATGAGGGAGAGCGGCTCGATCGACAAAAAAGAAT 15664

LsatSalinas_CDS 15615 GGGCTGGACGAGTGGAATGAGGGAGAGCGGCTCGATCGACAAAAAAGAAT 15664

LserUS96UC23_CD 15615 GGGCTGGACGAGTGGAATGAGGGAGAGCGGCTCGATCGACAAAAAAGAAT 15664

LvirLAC006941_C 15630 GGGCTGGACGAGTGGAATGAGGGAGAGCGGCTCGATCGACAAAAAAGAAT 15679

LserLAC005780_C 15648 GGGCTGGACGAGTGGAATGAGGGAGAGCGGCTCGATCGACAAAAAAGAAT 15697

LsalLAC008020_C 15648 GGGCTGGACGAGTGGAATGAGGGAGAGCGGCTCGATCGACAAAAAAGAAT 15697

LsalCGN5271_CDS 15648 GGGCTGGACGAGTGGAATGAGGGAGAGCGGCTCGATCGACAAAAAAGAAT 15697

LsatLACWENDEL_C 15665 AGATGTACATAGCAGTTCCAGCTGAAATACTTGGAATAATTCTACCACTT 15714

LsatSalinas_CDS 15665 AGATGTACATAGCAGTTCCAGCTGAAATACTTGGAATAATTCTACCACTT 15714

LserUS96UC23_CD 15665 AGATGTACATAGCAGTTCCAGCTGAAATACTTGGAATAATTCTACCACTT 15714

LvirLAC006941_C 15680 AGATGTACATAGCAGTTCCAGCTGAAATACTTGGAATAATTCTACCACTT 15729

LserLAC005780_C 15698 AGATGTACATAGCAGTTCCAGCTGAAATACTTGGAATAATTCTACCACTT 15747

LsalLAC008020_C 15698 AGATGTACATAGCAGTTCCAGCTGAAATACTTGGAATAATTCTACCACTT 15747

LsalCGN5271_CDS 15698 AGATGTACATAGCAGTTCCAGCTGAAATACTTGGAATAATTCTACCACTT 15747

LsatLACWENDEL_C 15715 CTACTAGGAGTAGCCTTTTTAGTGCTAGCTGAACGTAAAGTAATGGCTTT 15764

LsatSalinas_CDS 15715 CTACTAGGAGTAGCCTTTTTAGTGCTAGCTGAACGTAAAGTAATGGCTTT 15764

LserUS96UC23_CD 15715 CTACTAGGAGTAGCCTTTTTAGTGCTAGCTGAACGTAAAGTAATGGCTTT 15764

LvirLAC006941_C 15730 CTACTAGGAGTAGCCTTTTTAGTGCTAGCTGAACGTAAAGTAATGGCTTT 15779

LserLAC005780_C 15748 CTACTAGGAGTAGCCTTTTTAGTGCTAGCTGAACGTAAAGTAATGGCTTT 15797

LsalLAC008020_C 15748 CTACTAGGAGTAGCCTTTTTAGTGCTAGCTGAACGTAAAGTAATGGCTTT 15797

LsalCGN5271_CDS 15748 CTACTAGGAGTAGCCTTTTTAGTGCTAGCTGAACGTAAAGTAATGGCTTT 15797

LsatLACWENDEL_C 15765 TGTGCAACGTCGAAAGGGTCCTGATGTAGTGGGATCATTTGGATTGTTAC 15814

LsatSalinas_CDS 15765 TGTGCAACGTCGAAAGGGTCCTGATGTAGTGGGATCATTTGGATTGTTAC 15814

LserUS96UC23_CD 15765 TGTGCAACGTCGAAAGGGTCCTGATGTAGTGGGATCATTTGGATTGTTAC 15814

LvirLAC006941_C 15780 TGTGCAACGTCGAAAGGGTCCTGATGTAGTGGGATCATTTGGATTGTTAC 15829

LserLAC005780_C 15798 TGTGCAACGTCGAAAGGGTCCTGATGTAGTGGGATCATTTGGATTGTTAC 15847

LsalLAC008020_C 15798 TGTGCAACGTCGAAAGGGTCCTGATGTAGTGGGATCATTTGGATTGTTAC 15847

LsalCGN5271_CDS 15798 TGTGCAACGTCGAAAGGGTCCTGATGTAGTGGGATCATTTGGATTGTTAC 15847

LsatLACWENDEL_C 15815 AACCTCTAGCAGATGGTTTTAAATTGATTATAAAAGAACCTATTTCACCA 15864

LsatSalinas_CDS 15815 AACCTCTAGCAGATGGTTTTAAATTGATTATAAAAGAACCTATTTCACCA 15864

LserUS96UC23_CD 15815 AACCTCTAGCAGATGGTTTTAAATTGATTATAAAAGAACCTATTTCACCA 15864

LvirLAC006941_C 15830 AACCTCTAGCAGATGGTTTTAAATTGATTATAAAAGAACCTATTTCACCA 15879

LserLAC005780_C 15848 AACCTCTAGCAGATGGTTTTAAATTGATTATAAAAGAACCTATTTCACCA 15897

LsalLAC008020_C 15848 AACCTCTAGCAGATGGTTTTAAATTGATTATAAAAGAACCTATTTCACCA 15897

LsalCGN5271_CDS 15848 AACCTCTAGCAGATGGTTTTAAATTGATTATAAAAGAACCTATTTCACCA 15897

LsatLACWENDEL_C 15865 AGTAGTGCAAATTTCTCCCTTTTTAGAATGGCTCCAGTGGCTACATTTAT 15914

LsatSalinas_CDS 15865 AGTAGTGCAAATTTCTCCCTTTTTAGAATGGCTCCAGTGGCTACATTTAT 15914

LserUS96UC23_CD 15865 AGTAGTGCAAATTTCTCCCTTTTTAGAATGGCTCCAGTGGCTACATTTAT 15914

LvirLAC006941_C 15880 AGTAGTGCAAATTTCTCCCTTTTTAGAATGGCTCCAGTGGCTACATTTAT 15929

LserLAC005780_C 15898 AGTAGTGCAAATTTCTCCCTTTTTAGAATGGCTCCAGTGGCTACATTTAT 15947

LsalLAC008020_C 15898 AGTAGTGCAAATTTCTCCCTTTTTAGAATGGCTCCAGTGGCTACATTTAT 15947

LsalCGN5271_CDS 15898 AGTAGTGCAAATTTCTCCCTTTTTAGAATGGCTCCAGTGGCTACATTTAT 15947

LsatLACWENDEL_C 15915 GTTAAGTCTGGTCGCTCGGGCCGTTGTACCTTTTGATTATGGTATGGTAT 15964

LsatSalinas_CDS 15915 GTTAAGTCTGGTCGCTCGGGCCGTTGTACCTTTTGATTATGGTATGGTAT 15964

LserUS96UC23_CD 15915 GTTAAGTCTGGTCGCTCGGGCCGTTGTACCTTTTGATTATGGTATGGTAT 15964

LvirLAC006941_C 15930 GTTAAGTCTGGTCGCTCGGGCCGTTGTACCTTTTGATTATGGTATGGTAT 15979

LserLAC005780_C 15948 GTTAAGTCTGGTCGCTCGGGCCGTTGTACCTTTTGATTATGGTATGGTAT 15997

LsalLAC008020_C 15948 GTTAAGTCTGGTCGCTCGGGCCGTTGTACCTTTTGATTATGGTATGGTAT 15997

LsalCGN5271_CDS 15948 GTTAAGTCTGGTCGCTCGGGCCGTTGTACCTTTTGATTATGGTATGGTAT 15997

LsatLACWENDEL_C 15965 TGTCAGATCCGAACATAGGGCTACTTTATTTGTTTGCCATATCTTCGCTA 16014

LsatSalinas_CDS 15965 TGTCAGATCCGAACATAGGGCTACTTTATTTGTTTGCCATATCTTCGCTA 16014

LserUS96UC23_CD 15965 TGTCAGATCCGAACATAGGGCTACTTTATTTGTTTGCCATATCTTCGCTA 16014

LvirLAC006941_C 15980 TGTCAGATCCGAACATAGGGCTACTTTATTTGTTTGCCATATCTTCGCTA 16029

LserLAC005780_C 15998 TGTCAGATCCGAACATAGGGCTACTTTATTTGTTTGCCATATCTTCGCTA 16047

LsalLAC008020_C 15998 TGTCAGATCCGAACATAGGGCTACTTTATTTGTTTGCCATATCTTCGCTA 16047

LsalCGN5271_CDS 15998 TGTCAGATCCGAACATAGGGCTACTTTATTTGTTTGCCATATCTTCGCTA 16047

LsatLACWENDEL_C 16015 GGTGTTTATGGAATTATTATAGCAGGTCGGTCTAGTAATTCTTCAATGGG 16064

LsatSalinas_CDS 16015 GGTGTTTATGGAATTATTATAGCAGGTCGGTCTAGTAATTCTTCAATGGG 16064

LserUS96UC23_CD 16015 GGTGTTTATGGAATTATTATAGCAGGTCGGTCTAGTAATTCTTCAATGGG 16064

LvirLAC006941_C 16030 GGTGTTTATGGAATTATTATAGCAGGTCGGTCTAGTAATTCTTCAATGGG 16079

LserLAC005780_C 16048 GGTGTTTATGGAATTATTATAGCAGGTCGGTCTAGTAATTCTTCAATGGG 16097

LsalLAC008020_C 16048 GGTGTTTATGGAATTATTATAGCAGGTCGGTCTAGTAATTCTTCAATGGG 16097

LsalCGN5271_CDS 16048 GGTGTTTATGGAATTATTATAGCAGGTCGGTCTAGTAATTCTTCAATGGG 16097

LsatLACWENDEL_C 16065 GTCTGCTCTTTCTTTTTTGGGAGAGTATGCCAATATGATCTTAATGAGTC 16114

LsatSalinas_CDS 16065 GTCTGCTCTTTCTTTTTTGGGAGAGTATGCCAATATGATCTTAATGAGTC 16114

LserUS96UC23_CD 16065 GTCTGCTCTTTCTTTTTTGGGAGAGTATGCCAATATGATCTTAATGAGTC 16114

LvirLAC006941_C 16080 GTCTGCTCTTTCTTTTTTGGGAGAGTATGCCAATATGATCTTAATGAGTC 16129

LserLAC005780_C 16098 GTCTGCTCTTTCTTTTTTGGGAGAGTATGCCAATATGATCTTAATGAGTC 16147

LsalLAC008020_C 16098 GTCTGCTCTTTCTTTTTTGGGAGAGTATGCCAATATGATCTTAATGAGTC 16147

LsalCGN5271_CDS 16098 GTCTGCTCTTTCTTTTTTGGGAGAGTATGCCAATATGATCTTAATGAGTC 16147

LsatLACWENDEL_C 16115 GAAATATGCCTTTCTAGGAGCATTACGATCTGCAGCTCAAATGGTCCCTT 16164

LsatSalinas_CDS 16115 GAAATATGCCTTTCTAGGAGCATTACGATCTGCAGCTCAAATGGTCCCTT 16164

LserUS96UC23_CD 16115 GAAATATGCCTTTCTAGGAGCATTACGATCTGCAGCTCAAATGGTCCCTT 16164

LvirLAC006941_C 16130 GAAATATGCCTTTCTAGGAGCATTACGATCTGCAGCTCAAATGGTCCCTT 16179

LserLAC005780_C 16148 GAAATATGCCTTTCTAGGAGCATTACGATCTGCAGCTCAAATGGTCCCTT 16197

LsalLAC008020_C 16148 GAAATATGCCTTTCTAGGAGCATTACGATCTGCAGCTCAAATGGTCCCTT 16197

LsalCGN5271_CDS 16148 GAAATATGCCTTTCTAGGAGCATTACGATCTGCAGCTCAAATGGTCCCTT 16197

LsatLACWENDEL_C 16165 ATGAAGTCTCTATTGGTCTTATTCTTATTACTGTACTAATATGTGTAGGT 16214

LsatSalinas_CDS 16165 ATGAAGTCTCTATTGGTCTTATTCTTATTACTGTACTAATATGTGTAGGT 16214

LserUS96UC23_CD 16165 ATGAAGTCTCTATTGGTCTTATTCTTATTACTGTACTAATATGTGTAGGT 16214

LvirLAC006941_C 16180 ATGAAGTCTCTATTGGTCTTATTCTTATTACTGTACTAATATGTGTAGGT 16229

LserLAC005780_C 16198 ATGAAGTCTCTATTGGTCTTATTCTTATTACTGTACTAATATGTGTAGGT 16247

LsalLAC008020_C 16198 ATGAAGTCTCTATTGGTCTTATTCTTATTACTGTACTAATATGTGTAGGT 16247

LsalCGN5271_CDS 16198 ATGAAGTCTCTATTGGTCTTATTCTTATTACTGTACTAATATGTGTAGGT 16247

LsatLACWENDEL_C 16215 CCCCGTAATTCGAGTGAGATTGTCATGGCGCAAAAGCAGATATGGTCCGG 16264

LsatSalinas_CDS 16215 CCCCGTAATTCGAGTGAGATTGTCATGGCGCAAAAGCAGATATGGTCCGG 16264

LserUS96UC23_CD 16215 CCCCGTAATTCGAGTGAGATTGTCATGGCGCAAAAGCAGATATGGTCCGG 16264

LvirLAC006941_C 16230 CCCCGTAATTCGAGTGAGATTGTCATGGCGCAAAAGCAGATATGGTCCGG 16279

LserLAC005780_C 16248 CCCCGTAATTCGAGTGAGATTGTCATGGCGCAAAAGCAGATATGGTCCGG 16297

LsalLAC008020_C 16248 CCCCGTAATTCGAGTGAGATTGTCATGGCGCAAAAGCAGATATGGTCCGG 16297

LsalCGN5271_CDS 16248 CCCCGTAATTCGAGTGAGATTGTCATGGCGCAAAAGCAGATATGGTCCGG 16297

LsatLACWENDEL_C 16265 TATTCCCTTGTTCCCTGTATTGGTTATGTTCTTTATTTCTTGTCTAGCAG 16314

LsatSalinas_CDS 16265 TATTCCCTTGTTCCCTGTATTGGTTATGTTCTTTATTTCTTGTCTAGCAG 16314

LserUS96UC23_CD 16265 TATTCCCTTGTTCCCTGTATTGGTTATGTTCTTTATTTCTTGTCTAGCAG 16314

LvirLAC006941_C 16280 TATTCCCTTGTTCCCTGTATTGGTTATGTTCTTTATTTCTTGTCTAGCAG 16329

LserLAC005780_C 16298 TATTCCCTTGTTCCCTGTATTGGTTATGTTCTTTATTTCTTGTCTAGCAG 16347

LsalLAC008020_C 16298 TATTCCCTTGTTCCCTGTATTGGTTATGTTCTTTATTTCTTGTCTAGCAG 16347

LsalCGN5271_CDS 16298 TATTCCCTTGTTCCCTGTATTGGTTATGTTCTTTATTTCTTGTCTAGCAG 16347

LsatLACWENDEL_C 16315 AAACTAATCGAGCTCCGTTTGATCTCCCAGAAGCGGAAGCTGAATCAGTT 16364

LsatSalinas_CDS 16315 AAACTAATCGAGCTCCGTTTGATCTCCCAGAAGCGGAAGCTGAATCAGTT 16364

LserUS96UC23_CD 16315 AAACTAATCGAGCTCCGTTTGATCTCCCAGAAGCGGAAGCTGAATCAGTT 16364

LvirLAC006941_C 16330 AAACTAATCGAGCTCCGTTTGATCTCCCAGAAGCGGAAGCTGAATCAGTT 16379

LserLAC005780_C 16348 AAACTAATCGAGCTCCGTTTGATCTCCCAGAAGCGGAAGCTGAATCAGTT 16397

LsalLAC008020_C 16348 AAACTAATCGAGCTCCGTTTGATCTCCCAGAAGCGGAAGCTGAATCAGTT 16397

LsalCGN5271_CDS 16348 AAACTAATCGAGCTCCGTTTGATCTCCCAGAAGCGGAAGCTGAATCAGTT 16397

LsatLACWENDEL_C 16365 GCAGGCTATAATGTAGAATATATGTCAGAATTTGCACCTATTTTTATCTA 16414

LsatSalinas_CDS 16365 GCAGGCTATAATGTAGAATATATGTCAGAATTTGCACCTATTTTTATCTA 16414

LserUS96UC23_CD 16365 GCAGGCTATAATGTAGAATATATGTCAGAATTTGCACCTATTTTTATCTA 16414

LvirLAC006941_C 16380 GCAGGCTATAATGTAGAATATATGTCAGAATTTGCACCTATTTTTATCTA 16429

LserLAC005780_C 16398 GCAGGCTATAATGTAGAATATATGTCAGAATTTGCACCTATTTTTATCTA 16447

LsalLAC008020_C 16398 GCAGGCTATAATGTAGAATATATGTCAGAATTTGCACCTATTTTTATCTA 16447

LsalCGN5271_CDS 16398 GCAGGCTATAATGTAGAATATATGTCAGAATTTGCACCTATTTTTATCTA 16447

LsatLACWENDEL_C 16415 TTTAGTGATCAGTCCGCTAGTTTCTTTGATCCCACTCGGTGTTCCTTTTC 16464

LsatSalinas_CDS 16415 TTTAGTGATCAGTCCGCTAGTTTCTTTGATCCCACTCGGTGTTCCTTTTC 16464

LserUS96UC23_CD 16415 TTTAGTGATCAGTCCGCTAGTTTCTTTGATCCCACTCGGTGTTCCTTTTC 16464

LvirLAC006941_C 16430 TTTAGTGATCAGTCCGCTAGTTTCTTTGATCCCACTCGGTGTTCCTTTTC 16479

LserLAC005780_C 16448 TTTAGTGATCAGTCCGCTAGTTTCTTTGATCCCACTCGGTGTTCCTTTTC 16497

LsalLAC008020_C 16448 TTTAGTGATCAGTCCGCTAGTTTCTTTGATCCCACTCGGTGTTCCTTTTC 16497

LsalCGN5271_CDS 16448 TTTAGTGATCAGTCCGCTAGTTTCTTTGATCCCACTCGGTGTTCCTTTTC 16497

LsatLACWENDEL_C 16465 CATTTGCTTCCAATAGTTCGACCTACCCAGAAAAATTGTCGGCCTACGAA 16514

LsatSalinas_CDS 16465 CATTTGCTTCCAATAGTTCGACCTACCCAGAAAAATTGTCGGCCTACGAA 16514

LserUS96UC23_CD 16465 CATTTGCTTCCAATAGTTCGACCTACCCAGAAAAATTGTCGGCCTACGAA 16514

LvirLAC006941_C 16480 CATTTGCTTCCAATAGTTCGACCTACCCAGAAAAATTGTCGGCCTACGAA 16529

LserLAC005780_C 16498 CATTTGCTTCCAATAGTTCGACCTACCCAGAAAAATTGTCGGCCTACGAA 16547

LsalLAC008020_C 16498 CATTTGCTTCCAATAGTTCGACCTACCCAGAAAAATTGTCGGCCTACGAA 16547

LsalCGN5271_CDS 16498 CATTTGCTTCCAATAGTTCGACCTACCCAGAAAAATTGTCGGCCTACGAA 16547

LsatLACWENDEL_C 16515 TGTGGTTTCGATCCTTTCGGTGATGCCAGAAGTCGTTTTGATATACGATT 16564

LsatSalinas_CDS 16515 TGTGGTTTCGATCCTTTCGGTGATGCCAGAAGTCGTTTTGATATACGATT 16564

LserUS96UC23_CD 16515 TGTGGTTTCGATCCTTTCGGTGATGCCAGAAGTCGTTTTGATATACGATT 16564

LvirLAC006941_C 16530 TGTGGTTTCGATCCTTTCGGTGATGCCAGAAGTCGTTTTGATATACGATT 16579

LserLAC005780_C 16548 TGTGGTTTCGATCCTTTCGGTGATGCCAGAAGTCGTTTTGATATACGATT 16597

LsalLAC008020_C 16548 TGTGGTTTCGATCCTTTCGGTGATGCCAGAAGTCGTTTTGATATACGATT 16597

LsalCGN5271_CDS 16548 TGTGGTTTCGATCCTTTCGGTGATGCCAGAAGTCGTTTTGATATACGATT 16597

LsatLACWENDEL_C 16565 TTATCTTGTTTCTATTTTATTTATTATCCCTGATCCGGAAGTCACCTTTT 16614

LsatSalinas_CDS 16565 TTATCTTGTTTCTATTTTATTTATTATCCCTGATCCGGAAGTCACCTTTT 16614

LserUS96UC23_CD 16565 TTATCTTGTTTCTATTTTATTTATTATCCCTGATCCGGAAGTCACCTTTT 16614

LvirLAC006941_C 16580 TTATCTTGTTTCTATTTTATTTATTATCCCTGATCCGGAAGTCACCTTTT 16629

LserLAC005780_C 16598 TTATCTTGTTTCTATTTTATTTATTATCCCTGATCCGGAAGTCACCTTTT 16647

LsalLAC008020_C 16598 TTATCTTGTTTCTATTTTATTTATTATCCCTGATCCGGAAGTCACCTTTT 16647

LsalCGN5271_CDS 16598 TTATCTTGTTTCTATTTTATTTATTATCCCTGATCCGGAAGTCACCTTTT 16647

LsatLACWENDEL_C 16615 CCTTTCCTTGGGCAGTACCTCCCAACAAGATTGATCCCTTTGGATCTTGG 16664

LsatSalinas_CDS 16615 CCTTTCCTTGGGCAGTACCTCCCAACAAGATTGATCCCTTTGGATCTTGG 16664

LserUS96UC23_CD 16615 CCTTTCCTTGGGCAGTACCTCCCAACAAGATTGATCCCTTTGGATCTTGG 16664

LvirLAC006941_C 16630 CCTTTCCTTGGGCAGTACCTCCCAACAAGATTGATCCCTTTGGATCTTGG 16679

LserLAC005780_C 16648 CCTTTCCTTGGGCAGTACCTCCCAACAAGATTGATCCCTTTGGATCTTGG 16697

LsalLAC008020_C 16648 CCTTTCCTTGGGCAGTACCTCCCAACAAGATTGATCCCTTTGGATCTTGG 16697

LsalCGN5271_CDS 16648 CCTTTCCTTGGGCAGTACCTCCCAACAAGATTGATCCCTTTGGATCTTGG 16697

LsatLACWENDEL_C 16665 TCCATGATGGCCTTTTTATTGATTTTGACGATTGGATCTCTCTATGAATG 16714

LsatSalinas_CDS 16665 TCCATGATGGCCTTTTTATTGATTTTGACGATTGGATCTCTCTATGAATG 16714

LserUS96UC23_CD 16665 TCCATGATGGCCTTTTTATTGATTTTGACGATTGGATCTCTCTATGAATG 16714

LvirLAC006941_C 16680 TCCATGATGGCCTTTTTATTGATTTTGACGATTGGATCTCTCTATGAATG 16729

LserLAC005780_C 16698 TCCATGATGGCCTTTTTATTGATTTTGACGATTGGATCTCTCTATGAATG 16747

LsalLAC008020_C 16698 TCCATGATGGCCTTTTTATTGATTTTGACGATTGGATCTCTCTATGAATG 16747

LsalCGN5271_CDS 16698 TCCATGATGGCCTTTTTATTGATTTTGACGATTGGATCTCTCTATGAATG 16747

LsatLACWENDEL_C 16715 GAAAAGGGGTGCTTCGGATCGGGAGTAAATGTTAGAACATTTCTGTGAAT 16764

LsatSalinas_CDS 16715 GAAAAGGGGTGCTTCGGATCGGGAGTAAATGTTAGAACATTTCTGTGAAT 16764

LserUS96UC23_CD 16715 GAAAAGGGGTGCTTCGGATCGGGAGTAAATGTTAGAACATTTCTGTGAAT 16764

LvirLAC006941_C 16730 GAAAAGGGGTGCTTCGGATCGGGAGTAAATGTTAGAACATTTCTGTGAAT 16779

LserLAC005780_C 16748 GAAAAGGGGTGCTTCGGATCGGGAGTAAATGTTAGAACATTTCTGTGAAT 16797

LsalLAC008020_C 16748 GAAAAGGGGTGCTTCGGATCGGGAGTAAATGTTAGAACATTTCTGTGAAT 16797

LsalCGN5271_CDS 16748 GAAAAGGGGTGCTTCGGATCGGGAGTAAATGTTAGAACATTTCTGTGAAT 16797

LsatLACWENDEL_C 16765 GCTATTCTGATCTAAGTGGTCTTATTCTGTGTCCCGTGCTAGGAAGCATT 16814

LsatSalinas_CDS 16765 GCTATTCTGATCTAAGTGGTCTTATTCTGTGTCCCGTGCTAGGAAGCATT 16814

LserUS96UC23_CD 16765 GCTATTCTGATCTAAGTGGTCTTATTCTGTGTCCCGTGCTAGGAAGCATT 16814

LvirLAC006941_C 16780 GCTATTCTGATCTAAGTGGTCTTATTCTGTGTCCCGTGCTAGGAAGCATT 16829

LserLAC005780_C 16798 GCTATTCTGATCTAAGTGGTCTTATTCTGTGTCCCGTGCTAGGAAGCATT 16847

LsalLAC008020_C 16798 GCTATTCTGATCTAAGTGGTCTTATTCTGTGTCCCGTGCTAGGAAGCATT 16847

LsalCGN5271_CDS 16798 GCTATTCTGATCTAAGTGGTCTTATTCTGTGTCCCGTGCTAGGAAGCATT 16847

LsatLACWENDEL_C 16815 ACTCCTCTTTTCATTCCAAATTCAAGAATACGACCGATACGATTGATTGG 16864

LsatSalinas_CDS 16815 ACTCCTCTTTTCATTCCAAATTCAAGAATACGACCGATACGATTGATTGG 16864

LserUS96UC23_CD 16815 ACTCCTCTTTTCATTCCAAATTCAAGAATACGACCGATACGATTGATTGG 16864

LvirLAC006941_C 16830 ACTCCTCTTTTCATTCCAAATTCAAGAATACGACCGATACGATTGATTGG 16879

LserLAC005780_C 16848 ACTCCTCTTTTCATTCCAAATTCAAGAATACGACCGATACGATTGATTGG 16897

LsalLAC008020_C 16848 ACTCCTCTTTTCATTCCAAATTCAAGAATACGACCGATACGATTGATTGG 16897

LsalCGN5271_CDS 16848 ACTCCTCTTTTCATTCCAAATTCAAGAATACGACCGATACGATTGATTGG 16897

LsatLACWENDEL_C 16865 TCTGTGCGCCTCTCTTATTACTTTTTTGTATTCCCCTGTTCTTCGGATAC 16914

LsatSalinas_CDS 16865 TCTGTGCGCCTCTCTTATTACTTTTTTGTATTCCCCTGTTCTTCGGATAC 16914

LserUS96UC23_CD 16865 TCTGTGCGCCTCTCTTATTACTTTTTTGTATTCCCCTGTTCTTCGGATAC 16914

LvirLAC006941_C 16880 TCTGTGCGCCTCTCTTATTACTTTTTTGTATTCCCCTGTTCTTCGGATAC 16929

LserLAC005780_C 16898 TCTGTGCGCCTCTCTTATTACTTTTTTGTATTCCCCTGTTCTTCGGATAC 16947

LsalLAC008020_C 16898 TCTGTGCGCCTCTCTTATTACTTTTTTGTATTCCCCTGTTCTTCGGATAC 16947

LsalCGN5271_CDS 16898 TCTGTGCGCCTCTCTTATTACTTTTTTGTATTCCCCTGTTCTTCGGATAC 16947

LsatLACWENDEL_C 16915 AATTCGATCCTTCTACGGCCAAATCTCAATTTGTGGAAAGCCTTCGATGG 16964

LsatSalinas_CDS 16915 AATTCGATCCTTCTACGGCCAAATCTCAATTTGTGGAAAGCCTTCGATGG 16964

LserUS96UC23_CD 16915 AATTCGATCCTTCTACGGCCAAATCTCAATTTGTGGAAAGCCTTCGATGG 16964

LvirLAC006941_C 16930 AATTCGATCCTTCTACGGCCAAATCTCAATTTGTGGAAAGCCTTCGATGG 16979

LserLAC005780_C 16948 AATTCGATCCTTCTACGGCCAAATCTCAATTTGTGGAAAGCCTTCGATGG 16997

LsalLAC008020_C 16948 AATTCGATCCTTCTACGGCCAAATCTCAATTTGTGGAAAGCCTTCGATGG 16997

LsalCGN5271_CDS 16948 AATTCGATCCTTCTACGGCCAAATCTCAATTTGTGGAAAGCCTTCGATGG 16997

LsatLACWENDEL_C 16965 CTTCCTTATGAAAACATCCATTTTTATTTGGGTATAGACGGTATCTCTTT 17014

LsatSalinas_CDS 16965 CTTCCTTATGAAAACATCCATTTTTATTTGGGTATAGACGGTATCTCTTT 17014

LserUS96UC23_CD 16965 CTTCCTTATGAAAACATCCATTTTTATTTGGGTATAGACGGTATCTCTTT 17014

LvirLAC006941_C 16980 CTTCCTTATGAAAACATCCATTTTTATTTGGGTATAGACGGTATCTCTTT 17029

LserLAC005780_C 16998 CTTCCTTATGAAAACATCCATTTTTATTTGGGTATAGACGGTATCTCTTT 17047

LsalLAC008020_C 16998 CTTCCTTATGAAAACATCCATTTTTATTTGGGTATAGACGGTATCTCTTT 17047

LsalCGN5271_CDS 16998 CTTCCTTATGAAAACATCCATTTTTATTTGGGTATAGACGGTATCTCTTT 17047

LsatLACWENDEL_C 17015 ATTCTTCGTGATATTGACCACATTTCTGATCCCTATTTGCATTTTAGTGG 17064

LsatSalinas_CDS 17015 ATTCTTCGTGATATTGACCACATTTCTGATCCCTATTTGCATTTTAGTGG 17064

LserUS96UC23_CD 17015 ATTCTTCGTGATATTGACCACATTTCTGATCCCTATTTGCATTTTAGTGG 17064

LvirLAC006941_C 17030 ATTCTTCGTGATATTGACCACATTTCTGATCCCTATTTGCATTTTAGTGG 17079

LserLAC005780_C 17048 ATTCTTCGTGATATTGACCACATTTCTGATCCCTATTTGCATTTTAGTGG 17097

LsalLAC008020_C 17048 ATTCTTCGTGATATTGACCACATTTCTGATCCCTATTTGCATTTTAGTGG 17097

LsalCGN5271_CDS 17048 ATTCTTCGTGATATTGACCACATTTCTGATCCCTATTTGCATTTTAGTGG 17097

LsatLACWENDEL_C 17065 GTTGGTCTGGTATGAGAAGTTATGGGAAAGAGTATATTACAGCATCTCTA 17114

LsatSalinas_CDS 17065 GTTGGTCTGGTATGAGAAGTTATGGGAAAGAGTATATTACAGCATCTCTA 17114

LserUS96UC23_CD 17065 GTTGGTCTGGTATGAGAAGTTATGGGAAAGAGTATATTACAGCATCTCTA 17114

LvirLAC006941_C 17080 GTTGGTCTGGTATGAGAAGTTATGGGAAAGAGTATATTACAGCATCTCTA 17129

LserLAC005780_C 17098 GTTGGTCTGGTATGAGAAGTTATGGGAAAGAGTATATTACAGCATCTCTA 17147

LsalLAC008020_C 17098 GTTGGTCTGGTATGAGAAGTTATGGGAAAGAGTATATTACAGCATCTCTA 17147

LsalCGN5271_CDS 17098 GTTGGTCTGGTATGAGAAGTTATGGGAAAGAGTATATTACAGCATCTCTA 17147

LsatLACWENDEL_C 17115 ATTCGTGAATTTCTAATGATCGCCGTGTTCCGCATGCTGGATCCTCTACT 17164

LsatSalinas_CDS 17115 ATTCGTGAATTTCTAATGATCGCCGTGTTCCGCATGCTGGATCCTCTACT 17164

LserUS96UC23_CD 17115 ATTCGTGAATTTCTAATGATCGCCGTGTTCCGCATGCTGGATCCTCTACT 17164

LvirLAC006941_C 17130 ATTCGTGAATTTCTAATGATCGCCGTGTTCCGCATGCTGGATCCTCTACT 17179

LserLAC005780_C 17148 ATTCGTGAATTTCTAATGATCGCCGTGTTCCGCATGCTGGATCCTCTACT 17197

LsalLAC008020_C 17148 ATTCGTGAATTTCTAATGATCGCCGTGTTCCGCATGCTGGATCCTCTACT 17197

LsalCGN5271_CDS 17148 ATTCGTGAATTTCTAATGATCGCCGTGTTCCGCATGCTGGATCCTCTACT 17197

LsatLACWENDEL_C 17165 ATTCTATGTTCTTCCCGAAAGCGTGCTAATCCCTATGTTCATTATTATAG 17214

LsatSalinas_CDS 17165 ATTCTATGTTCTTCCCGAAAGCGTGCTAATCCCTATGTTCATTATTATAG 17214

LserUS96UC23_CD 17165 ATTCTATGTTCTTCCCGAAAGCGTGCTAATCCCTATGTTCATTATTATAG 17214

LvirLAC006941_C 17180 ATTCTATGTTCTTCCCGAAAGCGTGCTAATCCCTATGTTCATTATTATAG 17229

LserLAC005780_C 17198 ATTCTATGTTCTTCCCGAAAGCGTGCTAATCCCTATGTTCATTATTATAG 17247

LsalLAC008020_C 17198 ATTCTATGTTCTTCCCGAAAGCGTGCTAATCCCTATGTTCATTATTATAG 17247

LsalCGN5271_CDS 17198 ATTCTATGTTCTTCCCGAAAGCGTGCTAATCCCTATGTTCATTATTATAG 17247

LsatLACWENDEL_C 17215 GGGTATGGGGTTCGAGACAAAGAAAGATCAAGGCAGCATATCAGCTTTTC 17264

LsatSalinas_CDS 17215 GGGTATGGGGTTCGAGACAAAGAAAGATCAAGGCAGCATATCAGCTTTTC 17264

LserUS96UC23_CD 17215 GGGTATGGGGTTCGAGACAAAGAAAGATCAAGGCAGCATATCAGCTTTTC 17264

LvirLAC006941_C 17230 GGGTATGGGGTTCGAGACAAAGAAAGATCAAGGCAGCATATCAGCTTTTC 17279

LserLAC005780_C 17248 GGGTATGGGGTTCGAGACAAAGAAAGATCAAGGCAGCATATCAGCTTTTC 17297

LsalLAC008020_C 17248 GGGTATGGGGTTCGAGACAAAGAAAGATCAAGGCAGCATATCAGCTTTTC 17297

LsalCGN5271_CDS 17248 GGGTATGGGGTTCGAGACAAAGAAAGATCAAGGCAGCATATCAGCTTTTC 17297

LsatLACWENDEL_C 17265 CTTTATACTTTACTTGGATCTGTTTTTATGCTATTAGCTATTCTGTTGAT 17314

LsatSalinas_CDS 17265 CTTTATACTTTACTTGGATCTGTTTTTATGCTATTAGCTATTCTGTTGAT 17314

LserUS96UC23_CD 17265 CTTTATACTTTACTTGGATCTGTTTTTATGCTATTAGCTATTCTGTTGAT 17314

LvirLAC006941_C 17280 CTTTATACTTTACTTGGATCTGTTTTTATGCTATTAGCTATTCTGTTGAT 17329

LserLAC005780_C 17298 CTTTATACTTTACTTGGATCTGTTTTTATGCTATTAGCTATTCTGTTGAT 17347

LsalLAC008020_C 17298 CTTTATACTTTACTTGGATCTGTTTTTATGCTATTAGCTATTCTGTTGAT 17347

LsalCGN5271_CDS 17298 CTTTATACTTTACTTGGATCTGTTTTTATGCTATTAGCTATTCTGTTGAT 17347

LsatLACWENDEL_C 17315 TCTTTTCCAAACAGGAACCACCGATTTACAAATTTTATTAACCACAGAAT 17364

LsatSalinas_CDS 17315 TCTTTTCCAAACAGGAACCACCGATTTACAAATTTTATTAACCACAGAAT 17364

LserUS96UC23_CD 17315 TCTTTTCCAAACAGGAACCACCGATTTACAAATTTTATTAACCACAGAAT 17364

LvirLAC006941_C 17330 TCTTTTCCAAACAGGAACCACCGATTTACAAATTTTATTAACCACAGAAT 17379

LserLAC005780_C 17348 TCTTTTCCAAACAGGAACCACCGATTTACAAATTTTATTAACCACAGAAT 17397

LsalLAC008020_C 17348 TCTTTTCCAAACAGGAACCACCGATTTACAAATTTTATTAACCACAGAAT 17397

LsalCGN5271_CDS 17348 TCTTTTCCAAACAGGAACCACCGATTTACAAATTTTATTAACCACAGAAT 17397

LsatLACWENDEL_C 17365 TTAGTGAGCGGCGCCAAATCTTTCTATGGATTGCTTTTTTCGCCTCTTTC 17414

LsatSalinas_CDS 17365 TTAGTGAGCGGCGCCAAATCTTTCTATGGATTGCTTTTTTCGCCTCTTTC 17414

LserUS96UC23_CD 17365 TTAGTGAGCGGCGCCAAATCTTTCTATGGATTGCTTTTTTCGCCTCTTTC 17414

LvirLAC006941_C 17380 TTAGTGAGCGGCGCCAAATCTTTCTATGGATTGCTTTTTTCGCCTCTTTC 17429

LserLAC005780_C 17398 TTAGTGAGCGGCGCCAAATCTTTCTATGGATTGCTTTTTTCGCCTCTTTC 17447

LsalLAC008020_C 17398 TTAGTGAGCGGCGCCAAATCTTTCTATGGATTGCTTTTTTCGCCTCTTTC 17447

LsalCGN5271_CDS 17398 TTAGTGAGCGGCGCCAAATCTTTCTATGGATTGCTTTTTTCGCCTCTTTC 17447

LsatLACWENDEL_C 17415 GCCGTCAAAGTGCCTATGGTACCAGTTCATATTTGGTTACCCGAAGCTCA 17464

LsatSalinas_CDS 17415 GCCGTCAAAGTGCCTATGGTACCAGTTCATATTTGGTTACCCGAAGCTCA 17464

LserUS96UC23_CD 17415 GCCGTCAAAGTGCCTATGGTACCAGTTCATATTTGGTTACCCGAAGCTCA 17464

LvirLAC006941_C 17430 GCCGTCAAAGTGCCTATGGTACCAGTTCATATTTGGTTACCCGAAGCTCA 17479

LserLAC005780_C 17448 GCCGTCAAAGTGCCTATGGTACCAGTTCATATTTGGTTACCCGAAGCTCA 17497

LsalLAC008020_C 17448 GCCGTCAAAGTGCCTATGGTACCAGTTCATATTTGGTTACCCGAAGCTCA 17497

LsalCGN5271_CDS 17448 GCCGTCAAAGTGCCTATGGTACCAGTTCATATTTGGTTACCCGAAGCTCA 17497

LsatLACWENDEL_C 17465 TGTAGAGGCACCTACGGCAGGATCCGTCATCTTGGCGGGAATTCTTTTAA 17514

LsatSalinas_CDS 17465 TGTAGAGGCACCTACGGCAGGATCCGTCATCTTGGCGGGAATTCTTTTAA 17514

LserUS96UC23_CD 17465 TGTAGAGGCACCTACGGCAGGATCCGTCATCTTGGCGGGAATTCTTTTAA 17514

LvirLAC006941_C 17480 TGTAGAGGCACCTACGGCAGGATCCGTCATCTTGGCGGGAATTCTTTTAA 17529

LserLAC005780_C 17498 TGTAGAGGCACCTACGGCAGGATCCGTCATCTTGGCGGGAATTCTTTTAA 17547

LsalLAC008020_C 17498 TGTAGAGGCACCTACGGCAGGATCCGTCATCTTGGCGGGAATTCTTTTAA 17547

LsalCGN5271_CDS 17498 TGTAGAGGCACCTACGGCAGGATCCGTCATCTTGGCGGGAATTCTTTTAA 17547

LsatLACWENDEL_C 17515 AATTGGGAACCTACGGCTTTTTAAGATTTTCAATACCCATGTTTCCTGAA 17564

LsatSalinas_CDS 17515 AATTGGGAACCTACGGCTTTTTAAGATTTTCAATACCCATGTTTCCTGAA 17564

LserUS96UC23_CD 17515 AATTGGGAACCTACGGCTTTTTAAGATTTTCAATACCCATGTTTCCTGAA 17564

LvirLAC006941_C 17530 AATTGGGAACCTACGGCTTTTTAAGATTTTCAATACCCATGTTTCCTGAA 17579

LserLAC005780_C 17548 AATTGGGAACCTACGGCTTTTTAAGATTTTCAATACCCATGTTTCCTGAA 17597

LsalLAC008020_C 17548 AATTGGGAACCTACGGCTTTTTAAGATTTTCAATACCCATGTTTCCTGAA 17597

LsalCGN5271_CDS 17548 AATTGGGAACCTACGGCTTTTTAAGATTTTCAATACCCATGTTTCCTGAA 17597

LsatLACWENDEL_C 17565 GCGACACTTTGTTTCACTCCTTTCATTTATACTTTAAGCGCGATTGCTAT 17614

LsatSalinas_CDS 17565 GCGACACTTTGTTTCACTCCTTTCATTTATACTTTAAGCGCGATTGCTAT 17614

LserUS96UC23_CD 17565 GCGACACTTTGTTTCACTCCTTTCATTTATACTTTAAGCGCGATTGCTAT 17614

LvirLAC006941_C 17580 GCGACACTTTGTTTCACTCCTTTCATTTATACTTTAAGCGCGATTGCTAT 17629

LserLAC005780_C 17598 GCGACACTTTGTTTCACTCCTTTCATTTATACTTTAAGCGCGATTGCTAT 17647

LsalLAC008020_C 17598 GCGACACTTTGTTTCACTCCTTTCATTTATACTTTAAGCGCGATTGCTAT 17647

LsalCGN5271_CDS 17598 GCGACACTTTGTTTCACTCCTTTCATTTATACTTTAAGCGCGATTGCTAT 17647

LsatLACWENDEL_C 17615 AATATATACTTCCTTGACCACTTTAAGACAGATCGATCTTAAGAAGATCA 17664

LsatSalinas_CDS 17615 AATATATACTTCCTTGACCACTTTAAGACAGATCGATCTTAAGAAGATCA 17664

LserUS96UC23_CD 17615 AATATATACTTCCTTGACCACTTTAAGACAGATCGATCTTAAGAAGATCA 17664

LvirLAC006941_C 17630 AATATATACTTCCTTGACCACTTTAAGACAGATCGATCTTAAGAAGATCA 17679

LserLAC005780_C 17648 AATATATACTTCCTTGACCACTTTAAGACAGATCGATCTTAAGAAGATCA 17697

LsalLAC008020_C 17648 AATATATACTTCCTTGACCACTTTAAGACAGATCGATCTTAAGAAGATCA 17697

LsalCGN5271_CDS 17648 AATATATACTTCCTTGACCACTTTAAGACAGATCGATCTTAAGAAGATCA 17697

LsatLACWENDEL_C 17665 TTGCTTACTCCTCAGTAGCTCATATGAATCTGGTGACTATTGGTATGTTT 17714

LsatSalinas_CDS 17665 TTGCTTACTCCTCAGTAGCTCATATGAATCTGGTGACTATTGGTATGTTT 17714

LserUS96UC23_CD 17665 TTGCTTACTCCTCAGTAGCTCATATGAATCTGGTGACTATTGGTATGTTT 17714

LvirLAC006941_C 17680 TTGCTTACTCCTCAGTAGCTCATATGAATCTGGTGACTATTGGTATGTTT 17729

LserLAC005780_C 17698 TTGCTTACTCCTCAGTAGCTCATATGAATCTGGTGACTATTGGTATGTTT 17747

LsalLAC008020_C 17698 TTGCTTACTCCTCAGTAGCTCATATGAATCTGGTGACTATTGGTATGTTT 17747

LsalCGN5271_CDS 17698 TTGCTTACTCCTCAGTAGCTCATATGAATCTGGTGACTATTGGTATGTTT 17747

LsatLACWENDEL_C 17715 AGTCTGAACATACAGGGAATTGGAGGTAGCATTCTACTGATGTTAAGTCA 17764

LsatSalinas_CDS 17715 AGTCTGAACATACAGGGAATTGGAGGTAGCATTCTACTGATGTTAAGTCA 17764

LserUS96UC23_CD 17715 AGTCTGAACATACAGGGAATTGGAGGTAGCATTCTACTGATGTTAAGTCA 17764

LvirLAC006941_C 17730 AGTCTGAACATACAGGGAATTGGAGGTAGCATTCTACTGATGTTAAGTCA 17779

LserLAC005780_C 17748 AGTCTGAACATACAGGGAATTGGAGGTAGCATTCTACTGATGTTAAGTCA 17797

LsalLAC008020_C 17748 AGTCTGAACATACAGGGAATTGGAGGTAGCATTCTACTGATGTTAAGTCA 17797

LsalCGN5271_CDS 17748 AGTCTGAACATACAGGGAATTGGAGGTAGCATTCTACTGATGTTAAGTCA 17797

LsatLACWENDEL_C 17765 TGGACTGGTTTCTTCAGCCCTTTTTCTATGTGTTGGTGTTCTATATGACC 17814

LsatSalinas_CDS 17765 TGGACTGGTTTCTTCAGCCCTTTTTCTATGTGTTGGTGTTCTATATGACC 17814

LserUS96UC23_CD 17765 TGGACTGGTTTCTTCAGCCCTTTTTCTATGTGTTGGTGTTCTATATGACC 17814

LvirLAC006941_C 17780 TGGACTGGTTTCTTCAGCCCTTTTTCTATGTGTTGGTGTTCTATATGACC 17829

LserLAC005780_C 17798 TGGACTGGTTTCTTCAGCCCTTTTTCTATGTGTTGGTGTTCTATATGACC 17847

LsalLAC008020_C 17798 TGGACTGGTTTCTTCAGCCCTTTTTCTATGTGTTGGTGTTCTATATGACC 17847

LsalCGN5271_CDS 17798 TGGACTGGTTTCTTCAGCCCTTTTTCTATGTGTTGGTGTTCTATATGACC 17847

LsatLACWENDEL_C 17815 GACATAAGACTCGACTTGTTAGATATTACGGAGGTTTAGTGAGCACCATG 17864

LsatSalinas_CDS 17815 GACATAAGACTCGACTTGTTAGATATTACGGAGGTTTAGTGAGCACCATG 17864

LserUS96UC23_CD 17815 GACATAAGACTCGACTTGTTAGATATTACGGAGGTTTAGTGAGCACCATG 17864

LvirLAC006941_C 17830 GACATAAGACTCGACTTGTTAGATATTACGGAGGTTTAGTGAGCACCATG 17879

LserLAC005780_C 17848 GACATAAGACTCGACTTGTTAGATATTACGGAGGTTTAGTGAGCACCATG 17897

LsalLAC008020_C 17848 GACATAAGACTCGACTTGTTAGATATTACGGAGGTTTAGTGAGCACCATG 17897

LsalCGN5271_CDS 17848 GACATAAGACTCGACTTGTTAGATATTACGGAGGTTTAGTGAGCACCATG 17897

LsatLACWENDEL_C 17865 CCGAATTTCTCTACCATTTTCTTCTTTTTCACTTTGGCCAATATGAGTTT 17914

LsatSalinas_CDS 17865 CCGAATTTCTCTACCATTTTCTTCTTTTTCACTTTGGCCAATATGAGTTT 17914

LserUS96UC23_CD 17865 CCGAATTTCTCTACCATTTTCTTCTTTTTCACTTTGGCCAATATGAGTTT 17914

LvirLAC006941_C 17880 CCGAATTTCTCTACCATTTTCTTCTTTTTCACTTTGGCCAATATGAGTTT 17929

LserLAC005780_C 17898 CCGAATTTCTCTACCATTTTCTTCTTTTTCACTTTGGCCAATATGAGTTT 17947

LsalLAC008020_C 17898 CCGAATTTCTCTACCATTTTCTTCTTTTTCACTTTGGCCAATATGAGTTT 17947

LsalCGN5271_CDS 17898 CCGAATTTCTCTACCATTTTCTTCTTTTTCACTTTGGCCAATATGAGTTT 17947

LsatLACWENDEL_C 17915 ACCTGGTACTAGCAGCTTTATCGGGGAATTTCTCATCTTAGTAGGAGCTT 17964

LsatSalinas_CDS 17915 ACCTGGTACTAGCAGCTTTATCGGGGAATTTCTCATCTTAGTAGGAGCTT 17964

LserUS96UC23_CD 17915 ACCTGGTACTAGCAGCTTTATCGGGGAATTTCTCATCTTAGTAGGAGCTT 17964

LvirLAC006941_C 17930 ACCTGGTACTAGCAGCTTTATCGGGGAATTTCTCATCTTAGTAGGAGCTT 17979

LserLAC005780_C 17948 ACCTGGTACTAGCAGCTTTATCGGGGAATTTCTCATCTTAGTAGGAGCTT 17997

LsalLAC008020_C 17948 ACCTGGTACTAGCAGCTTTATCGGGGAATTTCTCATCTTAGTAGGAGCTT 17997

LsalCGN5271_CDS 17948 ACCTGGTACTAGCAGCTTTATCGGGGAATTTCTCATCTTAGTAGGAGCTT 17997

LsatLACWENDEL_C 17965 TCCAAAGAAATAGCTTAGTAGCCACATTAGCAGCGCTTGGGATGATTTTA 18014

LsatSalinas_CDS 17965 TCCAAAGAAATAGCTTAGTAGCCACATTAGCAGCGCTTGGGATGATTTTA 18014

LserUS96UC23_CD 17965 TCCAAAGAAATAGCTTAGTAGCCACATTAGCAGCGCTTGGGATGATTTTA 18014

LvirLAC006941_C 17980 TCCAAAGAAATAGCTTAGTAGCCACATTAGCAGCGCTTGGGATGATTTTA 18029

LserLAC005780_C 17998 TCCAAAGAAATAGCTTAGTAGCCACATTAGCAGCGCTTGGGATGATTTTA 18047

LsalLAC008020_C 17998 TCCAAAGAAATAGCTTAGTAGCCACATTAGCAGCGCTTGGGATGATTTTA 18047

LsalCGN5271_CDS 17998 TCCAAAGAAATAGCTTAGTAGCCACATTAGCAGCGCTTGGGATGATTTTA 18047

LsatLACWENDEL_C 18015 GGCGCGGCGTATTCCCTTTGGCTATATAATCGTGTGGTTTCTGGGAATTT 18064

LsatSalinas_CDS 18015 GGCGCGGCGTATTCCCTTTGGCTATATAATCGTGTGGTTTCTGGGAATTT 18064

LserUS96UC23_CD 18015 GGCGCGGCGTATTCCCTTTGGCTATATAATCGTGTGGTTTCTGGGAATTT 18064

LvirLAC006941_C 18030 GGCGCGGCGTATTCCCTTTGGCTATATAATCGTGTGGTTTCTGGGAATTT 18079

LserLAC005780_C 18048 GGCGCGGCGTATTCCCTTTGGCTATATAATCGTGTGGTTTCTGGGAATTT 18097

LsalLAC008020_C 18048 GGCGCGGCGTATTCCCTTTGGCTATATAATCGTGTGGTTTCTGGGAATTT 18097

LsalCGN5271_CDS 18048 GGCGCGGCGTATTCCCTTTGGCTATATAATCGTGTGGTTTCTGGGAATTT 18097

LsatLACWENDEL_C 18065 AAAACCGGATTTCCTCCATAAATTCTCCGATCTAAATGGTAGAGAAGTTT 18114

LsatSalinas_CDS 18065 AAAACCGGATTTCCTCCATAAATTCTCCGATCTAAATGGTAGAGAAGTTT 18114

LserUS96UC23_CD 18065 AAAACCGGATTTCCTCCATAAATTCTCCGATCTAAATGGTAGAGAAGTTT 18114

LvirLAC006941_C 18080 AAAACCGGATTTCCTCCATAAATTCTCCGATCTAAATGGTAGAGAAGTTT 18129

LserLAC005780_C 18098 AAAACCGGATTTCCTCCATAAATTCTCCGATCTAAATGGTAGAGAAGTTT 18147

LsalLAC008020_C 18098 AAAACCGGATTTCCTCCATAAATTCTCCGATCTAAATGGTAGAGAAGTTT 18147

LsalCGN5271_CDS 18098 AAAACCGGATTTCCTCCATAAATTCTCCGATCTAAATGGTAGAGAAGTTT 18147

LsatLACWENDEL_C 18115 TCATATTTATACCTTTTCTTGTTGGAGTTGTTTGGATGGGTGTTTACCCC 18164

LsatSalinas_CDS 18115 TCATATTTATACCTTTTCTTGTTGGAGTTGTTTGGATGGGTGTTTACCCC 18164

LserUS96UC23_CD 18115 TCATATTTATACCTTTTCTTGTTGGAGTTGTTTGGATGGGTGTTTACCCC 18164

LvirLAC006941_C 18130 TCATATTTATACCTTTTCTTGTTGGAGTTGTTTGGATGGGTGTTTACCCC 18179

LserLAC005780_C 18148 TCATATTTATACCTTTTCTTGTTGGAGTTGTTTGGATGGGTGTTTACCCC 18197

LsalLAC008020_C 18148 TCATATTTATACCTTTTCTTGTTGGAGTTGTTTGGATGGGTGTTTACCCC 18197

LsalCGN5271_CDS 18148 TCATATTTATACCTTTTCTTGTTGGAGTTGTTTGGATGGGTGTTTACCCC 18197

LsatLACWENDEL_C 18165 AAAGTGTTCCCGGACTGCATGCATACATCCGTAAGTAACTTAGTGCAACA 18214

LsatSalinas_CDS 18165 AAAGTGTTCCCGGACTGCATGCATACATCCGTAAGTAACTTAGTGCAACA 18214

LserUS96UC23_CD 18165 AAAGTGTTCCCGGACTGCATGCATACATCCGTAAGTAACTTAGTGCAACA 18214

LvirLAC006941_C 18180 AAAGTGTTCCCGGACTGCATGCATACATCCGTAAGTAACTTAGTGCAACA 18229

LserLAC005780_C 18198 AAAGTGTTCCCGGACTGCATGCATACATCCGTAAGTAACTTAGTGCAACA 18247

LsalLAC008020_C 18198 AAAGTGTTCCCGGACTGCATGCATACATCCGTAAGTAACTTAGTGCAACA 18247

LsalCGN5271_CDS 18198 AAAGTGTTCCCGGACTGCATGCATACATCCGTAAGTAACTTAGTGCAACA 18247

LsatLACWENDEL_C 18215 TGGAAAATTTCATTGAATGATCATCTCTATTTCAGGTATTCGGGGAATCC 18264

LsatSalinas_CDS 18215 TGGAAAATTTCATTGAATGATCATCTCTATTTCAGGTATTCGGGGAATCC 18264

LserUS96UC23_CD 18215 TGGAAAATTTCATTGAATGATCATCTCTATTTCAGGTATTCGGGGAATCC 18264

LvirLAC006941_C 18230 TGGAAAATTTCATTGAATGATCATCTCTATTTCAGGTATTCGGGGAATCC 18279

LserLAC005780_C 18248 TGGAAAATTTCATTGAATGATCATCTCTATTTCAGGTATTCGGGGAATCC 18297

LsalLAC008020_C 18248 TGGAAAATTTCATTGAATGATCATCTCTATTTCAGGTATTCGGGGAATCC 18297

LsalCGN5271_CDS 18248 TGGAAAATTTCATTGAATGATCATCTCTATTTCAGGTATTCGGGGAATCC 18297

LsatLACWENDEL_C 18265 TCCTTAATAGACGAAATATTCCTATTATGTCAATGCCAATTGAATCAATG 18314

LsatSalinas_CDS 18265 TCCTTAATAGACGAAATATTCCTATTATGTCAATGCCAATTGAATCAATG 18314

LserUS96UC23_CD 18265 TCCTTAATAGACGAAATATTCCTATTATGTCAATGCCAATTGAATCAATG 18314

LvirLAC006941_C 18280 TCCTTAATAGACGAAATATTCCTATTATGTCAATGCCAATTGAATCAATG 18329

LserLAC005780_C 18298 TCCTTAATAGACGAAATATTCCTATTATGTCAATGCCAATTGAATCAATG 18347

LsalLAC008020_C 18298 TCCTTAATAGACGAAATATTCCTATTATGTCAATGCCAATTGAATCAATG 18347

LsalCGN5271_CDS 18298 TCCTTAATAGACGAAATATTCCTATTATGTCAATGCCAATTGAATCAATG 18347

LsatLACWENDEL_C 18315 TTATTAGCTGTGAATTCGAACTTTTTGGTATTTTCCGTTTCTTCGGATGA 18364

LsatSalinas_CDS 18315 TTATTAGCTGTGAATTCGAACTTTTTGGTATTTTCCGTTTCTTCGGATGA 18364

LserUS96UC23_CD 18315 TTATTAGCTGTGAATTCGAACTTTTTGGTATTTTCCGTTTCTTCGGATGA 18364

LvirLAC006941_C 18330 TTATTAGCTGTGAATTCGAACTTTTTGGTATTTTCCGTTTCTTCGGATGA 18379

LserLAC005780_C 18348 TTATTAGCTGTGAATTCGAACTTTTTGGTATTTTCCGTTTCTTCGGATGA 18397

LsalLAC008020_C 18348 TTATTAGCTGTGAATTCGAACTTTTTGGTATTTTCCGTTTCTTCGGATGA 18397

LsalCGN5271_CDS 18348 TTATTAGCTGTGAATTCGAACTTTTTGGTATTTTCCGTTTCTTCGGATGA 18397

LsatLACWENDEL_C 18365 TATGATGGGTCAATCATTTGCTTCATTGGTTCCAACGGTGGCAGCTGCGG 18414

LsatSalinas_CDS 18365 TATGATGGGTCAATCATTTGCTTCATTGGTTCCAACGGTGGCAGCTGCGG 18414

LserUS96UC23_CD 18365 TATGATGGGTCAATCATTTGCTTCATTGGTTCCAACGGTGGCAGCTGCGG 18414

LvirLAC006941_C 18380 TATGATGGGTCAATCATTTGCTTCATTGGTTCCAACGGTGGCAGCTGCGG 18429

LserLAC005780_C 18398 TATGATGGGTCAATCATTTGCTTCATTGGTTCCAACGGTGGCAGCTGCGG 18447

LsalLAC008020_C 18398 TATGATGGGTCAATCATTTGCTTCATTGGTTCCAACGGTGGCAGCTGCGG 18447

LsalCGN5271_CDS 18398 TATGATGGGTCAATCATTTGCTTCATTGGTTCCAACGGTGGCAGCTGCGG 18447

LsatLACWENDEL_C 18415 AATCTGCTATTGGGTTAGCCATTTTCGTTATTACTTTCCGAGTCCGAGGG 18464

LsatSalinas_CDS 18415 AATCTGCTATTGGGTTAGCCATTTTCGTTATTACTTTCCGAGTCCGAGGG 18464

LserUS96UC23_CD 18415 AATCTGCTATTGGGTTAGCCATTTTCGTTATTACTTTCCGAGTCCGAGGG 18464

LvirLAC006941_C 18430 AATCTGCTATTGGGTTAGCCATTTTCGTTATTACTTTCCGAGTCCGAGGG 18479

LserLAC005780_C 18448 AATCTGCTATTGGGTTAGCCATTTTCGTTATTACTTTCCGAGTCCGAGGG 18497

LsalLAC008020_C 18448 AATCTGCTATTGGGTTAGCCATTTTCGTTATTACTTTCCGAGTCCGAGGG 18497

LsalCGN5271_CDS 18448 AATCTGCTATTGGGTTAGCCATTTTCGTTATTACTTTCCGAGTCCGAGGG 18497

LsatLACWENDEL_C 18465 ACTATTGCTGTAGAATCTATTAATAGCATTCAAGGTTAAATGTATCTACT 18514

LsatSalinas_CDS 18465 ACTATTGCTGTAGAATCTATTAATAGCATTCAAGGTTAAATGTATCTACT 18514

LserUS96UC23_CD 18465 ACTATTGCTGTAGAATCTATTAATAGCATTCAAGGTTAAATGTATCTACT 18514

LvirLAC006941_C 18480 ACTATTGCTGTAGAATCTATTAATAGCATTCAAGGTTAAATGTATCTACT 18529

LserLAC005780_C 18498 ACTATTGCTGTAGAATCTATTAATAGCATTCAAGGTTAAATGTATCTACT 18547

LsalLAC008020_C 18498 ACTATTGCTGTAGAATCTATTAATAGCATTCAAGGTTAAATGTATCTACT 18547

LsalCGN5271_CDS 18498 ACTATTGCTGTAGAATCTATTAATAGCATTCAAGGTTAAATGTATCTACT 18547

LsatLACWENDEL_C 18515 TATCATCTTTTTGCCCCTGCTCGGTAGTTCCGTAGCAGGTTTTTTCGGAC 18564

LsatSalinas_CDS 18515 TATCATCTTTTTGCCCCTGCTCGGTAGTTCCGTAGCAGGTTTTTTCGGAC 18564

LserUS96UC23_CD 18515 TATCATCTTTTTGCCCCTGCTCGGTAGTTCCGTAGCAGGTTTTTTCGGAC 18564

LvirLAC006941_C 18530 TATCATCTTTTTGCCCCTGCTCGGTAGTTCCGTAGCAGGTTTTTTCGGAC 18579

LserLAC005780_C 18548 TATCATCTTTTTGCCCCTGCTCGGTAGTTCCGTAGCAGGTTTTTTCGGAC 18597

LsalLAC008020_C 18548 TATCATCTTTTTGCCCCTGCTCGGTAGTTCCGTAGCAGGTTTTTTCGGAC 18597

LsalCGN5271_CDS 18548 TATCATCTTTTTGCCCCTGCTCGGTAGTTCCGTAGCAGGTTTTTTCGGAC 18597

LsatLACWENDEL_C 18565 GTTTTCTAGGATCAGAAGGAACCGCTATAATGACCACTACGTGCGTTTCA 18614

LsatSalinas_CDS 18565 GTTTTCTAGGATCAGAAGGAACCGCTATAATGACCACTACGTGCGTTTCA 18614

LserUS96UC23_CD 18565 GTTTTCTAGGATCAGAAGGAACCGCTATAATGACCACTACGTGCGTTTCA 18614

LvirLAC006941_C 18580 GTTTTCTAGGATCAGAAGGAACCGCTATAATGACCACTACGTGCGTTTCA 18629

LserLAC005780_C 18598 GTTTTCTAGGATCAGAAGGAACCGCTATAATGACCACTACGTGCGTTTCA 18647

LsalLAC008020_C 18598 GTTTTCTAGGATCAGAAGGAACCGCTATAATGACCACTACGTGCGTTTCA 18647

LsalCGN5271_CDS 18598 GTTTTCTAGGATCAGAAGGAACCGCTATAATGACCACTACGTGCGTTTCA 18647

LsatLACWENDEL_C 18615 TTCTCTTCGATCTTATCTTTGATTGCTTTTTATGAAGTCGCACCGGGAGC 18664

LsatSalinas_CDS 18615 TTCTCTTCGATCTTATCTTTGATTGCTTTTTATGAAGTCGCACCGGGAGC 18664

LserUS96UC23_CD 18615 TTCTCTTCGATCTTATCTTTGATTGCTTTTTATGAAGTCGCACCGGGAGC 18664

LvirLAC006941_C 18630 TTCTCTTCGATCTTATCTTTGATTGCTTTTTATGAAGTCGCACCGGGAGC 18679

LserLAC005780_C 18648 TTCTCTTCGATCTTATCTTTGATTGCTTTTTATGAAGTCGCACCGGGAGC 18697

LsalLAC008020_C 18648 TTCTCTTCGATCTTATCTTTGATTGCTTTTTATGAAGTCGCACCGGGAGC 18697

LsalCGN5271_CDS 18648 TTCTCTTCGATCTTATCTTTGATTGCTTTTTATGAAGTCGCACCGGGAGC 18697

LsatLACWENDEL_C 18665 TAGTGCTTGCTATCTAAGAATAGCTCCATGGATCTCATCGGAAATGTTTG 18714

LsatSalinas_CDS 18665 TAGTGCTTGCTATCTAAGAATAGCTCCATGGATCTCATCGGAAATGTTTG 18714

LserUS96UC23_CD 18665 TAGTGCTTGCTATCTAAGAATAGCTCCATGGATCTCATCGGAAATGTTTG 18714

LvirLAC006941_C 18680 TAGTGCTTGCTATCTAAGAATAGCTCCATGGATCTCATCGGAAATGTTTG 18729

LserLAC005780_C 18698 TAGTGCTTGCTATCTAAGAATAGCTCCATGGATCTCATCGGAAATGTTTG 18747

LsalLAC008020_C 18698 TAGTGCTTGCTATCTAAGAATAGCTCCATGGATCTCATCGGAAATGTTTG 18747

LsalCGN5271_CDS 18698 TAGTGCTTGCTATCTAAGAATAGCTCCATGGATCTCATCGGAAATGTTTG 18747

LsatLACWENDEL_C 18715 ATGCTTCTTGGGGCTTCGTGTTCGATAGCCCGACCGTAGTGATGTTAATT 18764

LsatSalinas_CDS 18715 ATGCTTCTTGGGGCTTCGTGTTCGATAGCCCGACCGTAGTGATGTTAATT 18764

LserUS96UC23_CD 18715 ATGCTTCTTGGGGCTTCGTGTTCGATAGCCCGACCGTAGTGATGTTAATT 18764

LvirLAC006941_C 18730 ATGCTTCTTGGGGCTTCGTGTTCGATAGCCCGACCGTAGTGATGTTAATT 18779

LserLAC005780_C 18748 ATGCTTCTTGGGGCTTCGTGTTCGATAGCCCGACCGTAGTGATGTTAATT 18797

LsalLAC008020_C 18748 ATGCTTCTTGGGGCTTCGTGTTCGATAGCCCGACCGTAGTGATGTTAATT 18797

LsalCGN5271_CDS 18748 ATGCTTCTTGGGGCTTCGTGTTCGATAGCCCGACCGTAGTGATGTTAATT 18797

LsatLACWENDEL_C 18765 GTGGTTACATCCATAAGTAGCTTGGTCCATCTTTATTCCATTTCATATAT 18814

LsatSalinas_CDS 18765 GTGGTTACATCCATAAGTAGCTTGGTCCATCTTTATTCCATTTCATATAT 18814

LserUS96UC23_CD 18765 GTGGTTACATCCATAAGTAGCTTGGTCCATCTTTATTCCATTTCATATAT 18814

LvirLAC006941_C 18780 GTGGTTACATCCATAAGTAGCTTGGTCCATCTTTATTCCATTTCATATAT 18829

LserLAC005780_C 18798 GTGGTTACATCCATAAGTAGCTTGGTCCATCTTTATTCCATTTCATATAT 18847

LsalLAC008020_C 18798 GTGGTTACATCCATAAGTAGCTTGGTCCATCTTTATTCCATTTCATATAT 18847

LsalCGN5271_CDS 18798 GTGGTTACATCCATAAGTAGCTTGGTCCATCTTTATTCCATTTCATATAT 18847

LsatLACWENDEL_C 18815 GTCTGAGGATCCGCATAGCCCTCGATTTATGTGTTATTTATCCATTCCTA 18864

LsatSalinas_CDS 18815 GTCTGAGGATCCGCATAGCCCTCGATTTATGTGTTATTTATCCATTCCTA 18864

LserUS96UC23_CD 18815 GTCTGAGGATCCGCATAGCCCTCGATTTATGTGTTATTTATCCATTCCTA 18864

LvirLAC006941_C 18830 GTCTGAGGATCCGCATAGCCCTCGATTTATGTGTTATTTATCCATTCCTA 18879

LserLAC005780_C 18848 GTCTGAGGATCCGCATAGCCCTCGATTTATGTGTTATTTATCCATTCCTA 18897

LsalLAC008020_C 18848 GTCTGAGGATCCGCATAGCCCTCGATTTATGTGTTATTTATCCATTCCTA 18897

LsalCGN5271_CDS 18848 GTCTGAGGATCCGCATAGCCCTCGATTTATGTGTTATTTATCCATTCCTA 18897

LsatLACWENDEL_C 18865 CTTTTTTTATGCCAATGTTGGTGACTGGAGATAACTCTCTTCAATTATTC 18914

LsatSalinas_CDS 18865 CTTTTTTTATGCCAATGTTGGTGACTGGAGATAACTCTCTTCAATTATTC 18914

LserUS96UC23_CD 18865 CTTTTTTTATGCCAATGTTGGTGACTGGAGATAACTCTCTTCAATTATTC 18914

LvirLAC006941_C 18880 CTTTTTTTATGCCAATGTTGGTGACTGGAGATAACTCTCTTCAATTATTC 18929

LserLAC005780_C 18898 CTTTTTTTATGCCAATGTTGGTGACTGGAGATAACTCTCTTCAATTATTC 18947

LsalLAC008020_C 18898 CTTTTTTTATGCCAATGTTGGTGACTGGAGATAACTCTCTTCAATTATTC 18947

LsalCGN5271_CDS 18898 CTTTTTTTATGCCAATGTTGGTGACTGGAGATAACTCTCTTCAATTATTC 18947

LsatLACWENDEL_C 18915 CTGGGATGGGAGGGAGTAGGTCTTGCTTCATATTTGTTAATTCATTTCTG 18964

LsatSalinas_CDS 18915 CTGGGATGGGAGGGAGTAGGTCTTGCTTCATATTTGTTAATTCATTTCTG 18964

LserUS96UC23_CD 18915 CTGGGATGGGAGGGAGTAGGTCTTGCTTCATATTTGTTAATTCATTTCTG 18964

LvirLAC006941_C 18930 CTGGGATGGGAGGGAGTAGGTCTTGCTTCATATTTGTTAATTCATTTCTG 18979

LserLAC005780_C 18948 CTGGGATGGGAGGGAGTAGGTCTTGCTTCATATTTGTTAATTCATTTCTG 18997

LsalLAC008020_C 18948 CTGGGATGGGAGGGAGTAGGTCTTGCTTCATATTTGTTAATTCATTTCTG 18997

LsalCGN5271_CDS 18948 CTGGGATGGGAGGGAGTAGGTCTTGCTTCATATTTGTTAATTCATTTCTG 18997

LsatLACWENDEL_C 18965 GTTTACACGACTTCAGGCAGATAAAGCAGCTATAAAAGCTATGCTTGTCA 19014

LsatSalinas_CDS 18965 GTTTACACGACTTCAGGCAGATAAAGCAGCTATAAAAGCTATGCTTGTCA 19014

LserUS96UC23_CD 18965 GTTTACACGACTTCAGGCAGATAAAGCAGCTATAAAAGCTATGCTTGTCA 19014

LvirLAC006941_C 18980 GTTTACACGACTTCAGGCAGATAAAGCAGCTATAAAAGCTATGCTTGTCA 19029

LserLAC005780_C 18998 GTTTACACGACTTCAGGCAGATAAAGCAGCTATAAAAGCTATGCTTGTCA 19047

LsalLAC008020_C 18998 GTTTACACGACTTCAGGCAGATAAAGCAGCTATAAAAGCTATGCTTGTCA 19047

LsalCGN5271_CDS 18998 GTTTACACGACTTCAGGCAGATAAAGCAGCTATAAAAGCTATGCTTGTCA 19047

LsatLACWENDEL_C 19015 ATCGAGTAGGTGATTTTGGATTAGCTCCTGGGATTTCGGGTTGTTTTACT 19064

LsatSalinas_CDS 19015 ATCGAGTAGGTGATTTTGGATTAGCTCCTGGGATTTCGGGTTGTTTTACT 19064

LserUS96UC23_CD 19015 ATCGAGTAGGTGATTTTGGATTAGCTCCTGGGATTTCGGGTTGTTTTACT 19064

LvirLAC006941_C 19030 ATCGAGTAGGTGATTTTGGATTAGCTCCTGGGATTTCGGGTTGTTTTACT 19079

LserLAC005780_C 19048 ATCGAGTAGGTGATTTTGGATTAGCTCCTGGGATTTCGGGTTGTTTTACT 19097

LsalLAC008020_C 19048 ATCGAGTAGGTGATTTTGGATTAGCTCCTGGGATTTCGGGTTGTTTTACT 19097

LsalCGN5271_CDS 19048 ATCGAGTAGGTGATTTTGGATTAGCTCCTGGGATTTCGGGTTGTTTTACT 19097

LsatLACWENDEL_C 19065 CTCTTTCAAACAGTAGACTTTTCCACCATTTTTGCTTGTGCTAGTGCCCC 19114

LsatSalinas_CDS 19065 CTCTTTCAAACAGTAGACTTTTCCACCATTTTTGCTTGTGCTAGTGCCCC 19114

LserUS96UC23_CD 19065 CTCTTTCAAACAGTAGACTTTTCCACCATTTTTGCTTGTGCTAGTGCCCC 19114

LvirLAC006941_C 19080 CTCTTTCAAACAGTAGACTTTTCCACCATTTTTGCTTGTGCTAGTGCCCC 19129

LserLAC005780_C 19098 CTCTTTCAAACAGTAGACTTTTCCACCATTTTTGCTTGTGCTAGTGCCCC 19147

LsalLAC008020_C 19098 CTCTTTCAAACAGTAGACTTTTCCACCATTTTTGCTTGTGCTAGTGCCCC 19147

LsalCGN5271_CDS 19098 CTCTTTCAAACAGTAGACTTTTCCACCATTTTTGCTTGTGCTAGTGCCCC 19147

LsatLACWENDEL_C 19115 CAGAAATTCTTGGATTTCTCGCAATATGAGATTGAATGCCATAACTCTTA 19164

LsatSalinas_CDS 19115 CAGAAATTCTTGGATTTCTCGCAATATGAGATTGAATGCCATAACTCTTA 19164

LserUS96UC23_CD 19115 CAGAAATTCTTGGATTTCTCGCAATATGAGATTGAATGCCATAACTCTTA 19164

LvirLAC006941_C 19130 CAGAAATTCTTGGATTTCTCGCAATATGAGATTGAATGCCATAACTCTTA 19179

LserLAC005780_C 19148 CAGAAATTCTTGGATTTCTCGCAATATGAGATTGAATGCCATAACTCTTA 19197

LsalLAC008020_C 19148 CAGAAATTCTTGGATTTCTCGCAATATGAGATTGAATGCCATAACTCTTA 19197

LsalCGN5271_CDS 19148 CAGAAATTCTTGGATTTCTCGCAATATGAGATTGAATGCCATAACTCTTA 19197

LsatLACWENDEL_C 19165 TTTGTATTTTACTTCTTATTGGTGCTGCTGGGAAATCTGCACAGATAGGA 19214

LsatSalinas_CDS 19165 TTTGTATTTTACTTCTTATTGGTGCTGCTGGGAAATCTGCACAGATAGGA 19214

LserUS96UC23_CD 19165 TTTGTATTTTACTTCTTATTGGTGCTGCTGGGAAATCTGCACAGATAGGA 19214

LvirLAC006941_C 19180 TTTGTATTTTACTTCTTATTGGTGCTGCTGGGAAATCTGCACAGATAGGA 19229

LserLAC005780_C 19198 TTTGTATTTTACTTCTTATTGGTGCTGCTGGGAAATCTGCACAGATAGGA 19247

LsalLAC008020_C 19198 TTTGTATTTTACTTCTTATTGGTGCTGCTGGGAAATCTGCACAGATAGGA 19247

LsalCGN5271_CDS 19198 TTTGTATTTTACTTCTTATTGGTGCTGCTGGGAAATCTGCACAGATAGGA 19247

LsatLACWENDEL_C 19215 TCGCATACTTGGTCACCCGATGCTATGGAGGGTCCCACTCCAGTATCCGC 19264

LsatSalinas_CDS 19215 TCGCATACTTGGTCACCCGATGCTATGGAGGGTCCCACTCCAGTATCCGC 19264

LserUS96UC23_CD 19215 TCGCATACTTGGTCACCCGATGCTATGGAGGGTCCCACTCCAGTATCCGC 19264

LvirLAC006941_C 19230 TCGCATACTTGGTCACCCGATGCTATGGAGGGTCCCACTCCAGTATCCGC 19279

LserLAC005780_C 19248 TCGCATACTTGGTCACCCGATGCTATGGAGGGTCCCACTCCAGTATCCGC 19297

LsalLAC008020_C 19248 TCGCATACTTGGTCACCCGATGCTATGGAGGGTCCCACTCCAGTATCCGC 19297

LsalCGN5271_CDS 19248 TCGCATACTTGGTCACCCGATGCTATGGAGGGTCCCACTCCAGTATCCGC 19297

LsatLACWENDEL_C 19265 TTTGATTCATGCAGCTACTATGGTAACAGCTGGCGTTTTCATGATAGCAA 19314

LsatSalinas_CDS 19265 TTTGATTCATGCAGCTACTATGGTAACAGCTGGCGTTTTCATGATAGCAA 19314

LserUS96UC23_CD 19265 TTTGATTCATGCAGCTACTATGGTAACAGCTGGCGTTTTCATGATAGCAA 19314

LvirLAC006941_C 19280 TTTGATTCATGCAGCTACTATGGTAACAGCTGGCGTTTTCATGATAGCAA 19329

LserLAC005780_C 19298 TTTGATTCATGCAGCTACTATGGTAACAGCTGGCGTTTTCATGATAGCAA 19347

LsalLAC008020_C 19298 TTTGATTCATGCAGCTACTATGGTAACAGCTGGCGTTTTCATGATAGCAA 19347

LsalCGN5271_CDS 19298 TTTGATTCATGCAGCTACTATGGTAACAGCTGGCGTTTTCATGATAGCAA 19347

LsatLACWENDEL_C 19315 GGTGCTCCCCTTTATTTGAATACCCACCTACGGCTTTGATTGTTATTACT 19364

LsatSalinas_CDS 19315 GGTGCTCCCCTTTATTTGAATACCCACCTACGGCTTTGATTGTTATTACT 19364

LserUS96UC23_CD 19315 GGTGCTCCCCTTTATTTGAATACCCACCTACGGCTTTGATTGTTATTACT 19364

LvirLAC006941_C 19330 GGTGCTCCCCTTTATTTGAATACCCACCTACGGCTTTGATTGTTATTACT 19379

LserLAC005780_C 19348 GGTGCTCCCCTTTATTTGAATACCCACCTACGGCTTTGATTGTTATTACT 19397

LsalLAC008020_C 19348 GGTGCTCCCCTTTATTTGAATACCCACCTACGGCTTTGATTGTTATTACT 19397

LsalCGN5271_CDS 19348 GGTGCTCCCCTTTATTTGAATACCCACCTACGGCTTTGATTGTTATTACT 19397

LsatLACWENDEL_C 19365 TTTGCAGGAGCTATGACGTCATTCCTTGCGGCAACCACTGGAATATTACA 19414

LsatSalinas_CDS 19365 TTTGCAGGAGCTATGACGTCATTCCTTGCGGCAACCACTGGAATATTACA 19414

LserUS96UC23_CD 19365 TTTGCAGGAGCTATGACGTCATTCCTTGCGGCAACCACTGGAATATTACA 19414

LvirLAC006941_C 19380 TTTGCAGGAGCTATGACGTCATTCCTTGCGGCAACCACTGGAATATTACA 19429

LserLAC005780_C 19398 TTTGCAGGAGCTATGACGTCATTCCTTGCGGCAACCACTGGAATATTACA 19447

LsalLAC008020_C 19398 TTTGCAGGAGCTATGACGTCATTCCTTGCGGCAACCACTGGAATATTACA 19447

LsalCGN5271_CDS 19398 TTTGCAGGAGCTATGACGTCATTCCTTGCGGCAACCACTGGAATATTACA 19447

LsatLACWENDEL_C 19415 GAACGATCTAAAGAGGGTCATAGCTTATTCAACTTGCAGTCAATTAGGCT 19464

LsatSalinas_CDS 19415 GAACGATCTAAAGAGGGTCATAGCTTATTCAACTTGCAGTCAATTAGGCT 19464

LserUS96UC23_CD 19415 GAACGATCTAAAGAGGGTCATAGCTTATTCAACTTGCAGTCAATTAGGCT 19464

LvirLAC006941_C 19430 GAACGATCTAAAGAGGGTCATAGCTTATTCAACTTGCAGTCAATTAGGCT 19479

LserLAC005780_C 19448 GAACGATCTAAAGAGGGTCATAGCTTATTCAACTTGCAGTCAATTAGGCT 19497

LsalLAC008020_C 19448 GAACGATCTAAAGAGGGTCATAGCTTATTCAACTTGCAGTCAATTAGGCT 19497

LsalCGN5271_CDS 19448 GAACGATCTAAAGAGGGTCATAGCTTATTCAACTTGCAGTCAATTAGGCT 19497

LsatLACWENDEL_C 19465 ATATGATCTTTGCTTGCGGCATCTCTAACTATTCGGTTAGCGTCTTTCAC 19514

LsatSalinas_CDS 19465 ATATGATCTTTGCTTGCGGCATCTCTAACTATTCGGTTAGCGTCTTTCAC 19514

LserUS96UC23_CD 19465 ATATGATCTTTGCTTGCGGCATCTCTAACTATTCGGTTAGCGTCTTTCAC 19514

LvirLAC006941_C 19480 ATATGATCTTTGCTTGCGGCATCTCTAACTATTCGGTTAGCGTCTTTCAC 19529

LserLAC005780_C 19498 ATATGATCTTTGCTTGCGGCATCTCTAACTATTCGGTTAGCGTCTTTCAC 19547

LsalLAC008020_C 19498 ATATGATCTTTGCTTGCGGCATCTCTAACTATTCGGTTAGCGTCTTTCAC 19547

LsalCGN5271_CDS 19498 ATATGATCTTTGCTTGCGGCATCTCTAACTATTCGGTTAGCGTCTTTCAC 19547

LsatLACWENDEL_C 19515 TTAATGAATCACGCCTTTTTCAAAGCATTACTATTCCTGAGTGCAGGTTC 19564

LsatSalinas_CDS 19515 TTAATGAATCACGCCTTTTTCAAAGCATTACTATTCCTGAGTGCAGGTTC 19564

LserUS96UC23_CD 19515 TTAATGAATCACGCCTTTTTCAAAGCATTACTATTCCTGAGTGCAGGTTC 19564

LvirLAC006941_C 19530 TTAATGAATCACGCCTTTTTCAAAGCATTACTATTCCTGAGTGCAGGTTC 19579

LserLAC005780_C 19548 TTAATGAATCACGCCTTTTTCAAAGCATTACTATTCCTGAGTGCAGGTTC 19597

LsalLAC008020_C 19548 TTAATGAATCACGCCTTTTTCAAAGCATTACTATTCCTGAGTGCAGGTTC 19597

LsalCGN5271_CDS 19548 TTAATGAATCACGCCTTTTTCAAAGCATTACTATTCCTGAGTGCAGGTTC 19597

LsatLACWENDEL_C 19565 GGTGATTCATGCCATGTCGGATGAGCAAGATATGCGGAAGATGGGGGGGC 19614

LsatSalinas_CDS 19565 GGTGATTCATGCCATGTCGGATGAGCAAGATATGCGGAAGATGGGGGGGC 19614

LserUS96UC23_CD 19565 GGTGATTCATGCCATGTCGGATGAGCAAGATATGCGGAAGATGGGGGGGC 19614

LvirLAC006941_C 19580 GGTGATTCATGCCATGTCGGATGAGCAAGATATGCGGAAGATGGGGGGGC 19629

LserLAC005780_C 19598 GGTGATTCATGCCATGTCGGATGAGCAAGATATGCGGAAGATGGGGGGGC 19647

LsalLAC008020_C 19598 GGTGATTCATGCCATGTCGGATGAGCAAGATATGCGGAAGATGGGGGGGC 19647

LsalCGN5271_CDS 19598 GGTGATTCATGCCATGTCGGATGAGCAAGATATGCGGAAGATGGGGGGGC 19647

LsatLACWENDEL_C 19615 TTGCCTCCTCATTCCCTTTTACCTATGCCATGATGCTCATGGGCAGCTTA 19664

LsatSalinas_CDS 19615 TTGCCTCCTCATTCCCTTTTACCTATGCCATGATGCTCATGGGCAGCTTA 19664

LserUS96UC23_CD 19615 TTGCCTCCTCATTCCCTTTTACCTATGCCATGATGCTCATGGGCAGCTTA 19664

LvirLAC006941_C 19630 TTGCCTCCTCATTCCCTTTTACCTATGCCATGATGCTCATGGGCAGCTTA 19679

LserLAC005780_C 19648 TTGCCTCCTCATTCCCTTTTACCTATGCCATGATGCTCATGGGCAGCTTA 19697

LsalLAC008020_C 19648 TTGCCTCCTCATTCCCTTTTACCTATGCCATGATGCTCATGGGCAGCTTA 19697

LsalCGN5271_CDS 19648 TTGCCTCCTCATTCCCTTTTACCTATGCCATGATGCTCATGGGCAGCTTA 19697

LsatLACWENDEL_C 19665 TCTCTAATTGGATTTCCTTTTCCAACTGGATTTTATTCCAAAGATGTGAT 19714

LsatSalinas_CDS 19665 TCTCTAATTGGATTTCCTTTTCCAACTGGATTTTATTCCAAAGATGTGAT 19714

LserUS96UC23_CD 19665 TCTCTAATTGGATTTCCTTTTCCAACTGGATTTTATTCCAAAGATGTGAT 19714

LvirLAC006941_C 19680 TCTCTAATTGGATTTCCTTTTCCAACTGGATTTTATTCCAAAGATGTGAT 19729

LserLAC005780_C 19698 TCTCTAATTGGATTTCCTTTTCCAACTGGATTTTATTCCAAAGATGTGAT 19747

LsalLAC008020_C 19698 TCTCTAATTGGATTTCCTTTTCCAACTGGATTTTATTCCAAAGATGTGAT 19747

LsalCGN5271_CDS 19698 TCTCTAATTGGATTTCCTTTTCCAACTGGATTTTATTCCAAAGATGTGAT 19747

LsatLACWENDEL_C 19715 CTTAGAGCTCGCTTACACTAAGTATACCATCAGTGGGAACTTTGCTTTCT 19764

LsatSalinas_CDS 19715 CTTAGAGCTCGCTTACACTAAGTATACCATCAGTGGGAACTTTGCTTTCT 19764

LserUS96UC23_CD 19715 CTTAGAGCTCGCTTACACTAAGTATACCATCAGTGGGAACTTTGCTTTCT 19764

LvirLAC006941_C 19730 CTTAGAGCTCGCTTACACTAAGTATACCATCAGTGGGAACTTTGCTTTCT 19779

LserLAC005780_C 19748 CTTAGAGCTCGCTTACACTAAGTATACCATCAGTGGGAACTTTGCTTTCT 19797

LsalLAC008020_C 19748 CTTAGAGCTCGCTTACACTAAGTATACCATCAGTGGGAACTTTGCTTTCT 19797

LsalCGN5271_CDS 19748 CTTAGAGCTCGCTTACACTAAGTATACCATCAGTGGGAACTTTGCTTTCT 19797

LsatLACWENDEL_C 19765 GGTTGGGAAGTGTCTCTGTCCTTTTCACTTCTTATTACTCTTTTCGTTCA 19814

LsatSalinas_CDS 19765 GGTTGGGAAGTGTCTCTGTCCTTTTCACTTCTTATTACTCTTTTCGTTCA 19814

LserUS96UC23_CD 19765 GGTTGGGAAGTGTCTCTGTCCTTTTCACTTCTTATTACTCTTTTCGTTCA 19814

LvirLAC006941_C 19780 GGTTGGGAAGTGTCTCTGTCCTTTTCACTTCTTATTACTCTTTTCGTTCA 19829

LserLAC005780_C 19798 GGTTGGGAAGTGTCTCTGTCCTTTTCACTTCTTATTACTCTTTTCGTTCA 19847

LsalLAC008020_C 19798 GGTTGGGAAGTGTCTCTGTCCTTTTCACTTCTTATTACTCTTTTCGTTCA 19847

LsalCGN5271_CDS 19798 GGTTGGGAAGTGTCTCTGTCCTTTTCACTTCTTATTACTCTTTTCGTTCA 19847

LsatLACWENDEL_C 19815 CTTTTTCTAACATTTCTAGGACCAACTAATTCATTCGGGCGAGACATCTT 19864

LsatSalinas_CDS 19815 CTTTTTCTAACATTTCTAGGACCAACTAATTCATTCGGGCGAGACATCTT 19864

LserUS96UC23_CD 19815 CTTTTTCTAACATTTCTAGGACCAACTAATTCATTCGGGCGAGACATCTT 19864

LvirLAC006941_C 19830 CTTTTTCTAACATTTCTAGGACCAACTAATTCATTCGGGCGAGACATCTT 19879

LserLAC005780_C 19848 CTTTTTCTAACATTTCTAGGACCAACTAATTCATTCGGGCGAGACATCTT 19897

LsalLAC008020_C 19848 CTTTTTCTAACATTTCTAGGACCAACTAATTCATTCGGGCGAGACATCTT 19897

LsalCGN5271_CDS 19848 CTTTTTCTAACATTTCTAGGACCAACTAATTCATTCGGGCGAGACATCTT 19897

LsatLACWENDEL_C 19865 ACGATGTCATGATGCGCCCATTCCTATGGCCATTCCTTTAATACTTCTGG 19914

LsatSalinas_CDS 19865 ACGATGTCATGATGCGCCCATTCCTATGGCCATTCCTTTAATACTTCTGG 19914

LserUS96UC23_CD 19865 ACGATGTCATGATGCGCCCATTCCTATGGCCATTCCTTTAATACTTCTGG 19914

LvirLAC006941_C 19880 ACGATGTCATGATGCGCCCATTCCTATGGCCATTCCTTTAATACTTCTGG 19929

LserLAC005780_C 19898 ACGATGTCATGATGCGCCCATTCCTATGGCCATTCCTTTAATACTTCTGG 19947

LsalLAC008020_C 19898 ACGATGTCATGATGCGCCCATTCCTATGGCCATTCCTTTAATACTTCTGG 19947

LsalCGN5271_CDS 19898 ACGATGTCATGATGCGCCCATTCCTATGGCCATTCCTTTAATACTTCTGG 19947

LsatLACWENDEL_C 19915 CTCTCGGGAGTCTCTTTGTAGGATACTTGGCCAAACCAATTTTTGGGCCA 19964

LsatSalinas_CDS 19915 CTCTCGGGAGTCTCTTTGTAGGATACTTGGCCAAACCAATTTTTGGGCCA 19964

LserUS96UC23_CD 19915 CTCTCGGGAGTCTCTTTGTAGGATACTTGGCCAAACCAATTTTTGGGCCA 19964

LvirLAC006941_C 19930 CTCTCGGGAGTCTCTTTGTAGGATACTTGGCCAAACCAATTTTTGGGCCA 19979

LserLAC005780_C 19948 CTCTCGGGAGTCTCTTTGTAGGATACTTGGCCAAACCAATTTTTGGGCCA 19997

LsalLAC008020_C 19948 CTCTCGGGAGTCTCTTTGTAGGATACTTGGCCAAACCAATTTTTGGGCCA 19997

LsalCGN5271_CDS 19948 CTCTCGGGAGTCTCTTTGTAGGATACTTGGCCAAACCAATTTTTGGGCCA 19997

LsatLACWENDEL_C 19965 ATTCCCCCTTCGTACTACCAAAAAATGAGATTCTTGCCGAATCCGAGTTT 20014

LsatSalinas_CDS 19965 ATTCCCCCTTCGTACTACCAAAAAATGAGATTCTTGCCGAATCCGAGTTT 20014

LserUS96UC23_CD 19965 ATTCCCCCTTCGTACTACCAAAAAATGAGATTCTTGCCGAATCCGAGTTT 20014

LvirLAC006941_C 19980 ATTCCCCCTTCGTACTACCAAAAAATGAGATTCTTGCCGAATCCGAGTTT 20029

LserLAC005780_C 19998 ATTCCCCCTTCGTACTACCAAAAAATGAGATTCTTGCCGAATCCGAGTTT 20047

LsalLAC008020_C 19998 ATTCCCCCTTCGTACTACCAAAAAATGAGATTCTTGCCGAATCCGAGTTT 20047

LsalCGN5271_CDS 19998 ATTCCCCCTTCGTACTACCAAAAAATGAGATTCTTGCCGAATCCGAGTTT 20047

LsatLACWENDEL_C 20015 GCTGCTCCAACCATTACCAAACTAATACCTATTCCGTTTAGTACTTCAGG 20064

LsatSalinas_CDS 20015 GCTGCTCCAACCATTACCAAACTAATACCTATTCCGTTTAGTACTTCAGG 20064

LserUS96UC23_CD 20015 GCTGCTCCAACCATTACCAAACTAATACCTATTCCGTTTAGTACTTCAGG 20064

LvirLAC006941_C 20030 GCTGCTCCAACCATTACCAAACTAATACCTATTCCGTTTAGTACTTCAGG 20079

LserLAC005780_C 20048 GCTGCTCCAACCATTACCAAACTAATACCTATTCCGTTTAGTACTTCAGG 20097

LsalLAC008020_C 20048 GCTGCTCCAACCATTACCAAACTAATACCTATTCCGTTTAGTACTTCAGG 20097

LsalCGN5271_CDS 20048 GCTGCTCCAACCATTACCAAACTAATACCTATTCCGTTTAGTACTTCAGG 20097

LsatLACWENDEL_C 20065 TGCTTCTGTTGCGTATAATGTAAATCCAGTAGCGGATCAATTCCAACGAG 20114

LsatSalinas_CDS 20065 TGCTTCTGTTGCGTATAATGTAAATCCAGTAGCGGATCAATTCCAACGAG 20114

LserUS96UC23_CD 20065 TGCTTCTGTTGCGTATAATGTAAATCCAGTAGCGGATCAATTCCAACGAG 20114

LvirLAC006941_C 20080 TGCTTCTGTTGCGTATAATGTAAATCCAGTAGCGGATCAATTCCAACGAG 20129

LserLAC005780_C 20098 TGCTTCTGTTGCGTATAATGTAAATCCAGTAGCGGATCAATTCCAACGAG 20147

LsalLAC008020_C 20098 TGCTTCTGTTGCGTATAATGTAAATCCAGTAGCGGATCAATTCCAACGAG 20147

LsalCGN5271_CDS 20098 TGCTTCTGTTGCGTATAATGTAAATCCAGTAGCGGATCAATTCCAACGAG 20147

LsatLACWENDEL_C 20115 CCTTTCAAACTAGTACTTTTTGTAATCGACTCTATAGCTTCTTCAATAAA 20164

LsatSalinas_CDS 20115 CCTTTCAAACTAGTACTTTTTGTAATCGACTCTATAGCTTCTTCAATAAA 20164

LserUS96UC23_CD 20115 CCTTTCAAACTAGTACTTTTTGTAATCGACTCTATAGCTTCTTCAATAAA 20164

LvirLAC006941_C 20130 CCTTTCAAACTAGTACTTTTTGTAATCGACTCTATAGCTTCTTCAATAAA 20179

LserLAC005780_C 20148 CCTTTCAAACTAGTACTTTTTGTAATCGACTCTATAGCTTCTTCAATAAA 20197

LsalLAC008020_C 20148 CCTTTCAAACTAGTACTTTTTGTAATCGACTCTATAGCTTCTTCAATAAA 20197

LsalCGN5271_CDS 20148 CCTTTCAAACTAGTACTTTTTGTAATCGACTCTATAGCTTCTTCAATAAA 20197

LsatLACWENDEL_C 20165 CGCTGGTTCTTCGATCAAGTTTTTAATGACTTTCTAGTCAGATCGTTCCT 20214

LsatSalinas_CDS 20165 CGCTGGTTCTTCGATCAAGTTTTTAATGACTTTCTAGTCAGATCGTTCCT 20214

LserUS96UC23_CD 20165 CGCTGGTTCTTCGATCAAGTTTTTAATGACTTTCTAGTCAGATCGTTCCT 20214

LvirLAC006941_C 20180 CGCTGGTTCTTCGATCAAGTTTTTAATGACTTTCTAGTCAGATCGTTCCT 20229

LserLAC005780_C 20198 CGCTGGTTCTTCGATCAAGTTTTTAATGACTTTCTAGTCAGATCGTTCCT 20247

LsalLAC008020_C 20198 CGCTGGTTCTTCGATCAAGTTTTTAATGACTTTCTAGTCAGATCGTTCCT 20247

LsalCGN5271_CDS 20198 CGCTGGTTCTTCGATCAAGTTTTTAATGACTTTCTAGTCAGATCGTTCCT 20247

LsatLACWENDEL_C 20215 GCGTTTCGGATATGAAGTTTCATTCGAAGCTTTAGACAAAGGTGCTATTG 20264

LsatSalinas_CDS 20215 GCGTTTCGGATATGAAGTTTCATTCGAAGCTTTAGACAAAGGTGCTATTG 20264

LserUS96UC23_CD 20215 GCGTTTCGGATATGAAGTTTCATTCGAAGCTTTAGACAAAGGTGCTATTG 20264

LvirLAC006941_C 20230 GCGTTTCGGATATGAAGTTTCATTCGAAGCTTTAGACAAAGGTGCTATTG 20279

LserLAC005780_C 20248 GCGTTTCGGATATGAAGTTTCATTCGAAGCTTTAGACAAAGGTGCTATTG 20297

LsalLAC008020_C 20248 GCGTTTCGGATATGAAGTTTCATTCGAAGCTTTAGACAAAGGTGCTATTG 20297

LsalCGN5271_CDS 20248 GCGTTTCGGATATGAAGTTTCATTCGAAGCTTTAGACAAAGGTGCTATTG 20297

LsatLACWENDEL_C 20265 AGATATTGGGCCCTTATGGTATCTCGTACACATTCCGACGATTGGCCGAG 20314

LsatSalinas_CDS 20265 AGATATTGGGCCCTTATGGTATCTCGTACACATTCCGACGATTGGCCGAG 20314

LserUS96UC23_CD 20265 AGATATTGGGCCCTTATGGTATCTCGTACACATTCCGACGATTGGCCGAG 20314

LvirLAC006941_C 20280 AGATATTGGGCCCTTATGGTATCTCGTACACATTCCGACGATTGGCCGAG 20329

LserLAC005780_C 20298 AGATATTGGGCCCTTATGGTATCTCGTACACATTCCGACGATTGGCCGAG 20347

LsalLAC008020_C 20298 AGATATTGGGCCCTTATGGTATCTCGTACACATTCCGACGATTGGCCGAG 20347

LsalCGN5271_CDS 20298 AGATATTGGGCCCTTATGGTATCTCGTACACATTCCGACGATTGGCCGAG 20347

LsatLACWENDEL_C 20315 CGAATAAGTCAACTTCAAAGTGGATTTGTTTATCATTATGCCTTTGCAAT 20364

LsatSalinas_CDS 20315 CGAATAAGTCAACTTCAAAGTGGATTTGTTTATCATTATGCCTTTGCAAT 20364

LserUS96UC23_CD 20315 CGAATAAGTCAACTTCAAAGTGGATTTGTTTATCATTATGCCTTTGCAAT 20364

LvirLAC006941_C 20330 CGAATAAGTCAACTTCAAAGTGGATTTGTTTATCATTATGCCTTTGCAAT 20379

LserLAC005780_C 20348 CGAATAAGTCAACTTCAAAGTGGATTTGTTTATCATTATGCCTTTGCAAT 20397

LsalLAC008020_C 20348 CGAATAAGTCAACTTCAAAGTGGATTTGTTTATCATTATGCCTTTGCAAT 20397

LsalCGN5271_CDS 20348 CGAATAAGTCAACTTCAAAGTGGATTTGTTTATCATTATGCCTTTGCAAT 20397

LsatLACWENDEL_C 20365 GTTACTTGGTTCAACTCTATTTGTGACCTTTTCTCGTATGTGGGACTCTC 20414

LsatSalinas_CDS 20365 GTTACTTGGTTCAACTCTATTTGTGACCTTTTCTCGTATGTGGGACTCTC 20414

LserUS96UC23_CD 20365 GTTACTTGGTTCAACTCTATTTGTGACCTTTTCTCGTATGTGGGACTCTC 20414

LvirLAC006941_C 20380 GTTACTTGGTTCAACTCTATTTGTGACCTTTTCTCGTATGTGGGACTCTC 20429

LserLAC005780_C 20398 GTTACTTGGTTCAACTCTATTTGTGACCTTTTCTCGTATGTGGGACTCTC 20447

LsalLAC008020_C 20398 GTTACTTGGTTCAACTCTATTTGTGACCTTTTCTCGTATGTGGGACTCTC 20447

LsalCGN5271_CDS 20398 GTTACTTGGTTCAACTCTATTTGTGACCTTTTCTCGTATGTGGGACTCTC 20447

LsatLACWENDEL_C 20415 TATCTTCTTGGGTAGATAATCGATCGTCTTTCATTTGGATAGTGAGTTGT 20464

LsatSalinas_CDS 20415 TATCTTCTTGGGTAGATAATCGATCGTCTTTCATTTGGATAGTGAGTTGT 20464

LserUS96UC23_CD 20415 TATCTTCTTGGGTAGATAATCGATCGTCTTTCATTTGGATAGTGAGTTGT 20464

LvirLAC006941_C 20430 TATCTTCTTGGGTAGATAATCGATCGTCTTTCATTTGGATAGTGAGTTGT 20479

LserLAC005780_C 20448 TATCTTCTTGGGTAGATAATCGATCGTCTTTCATTTGGATAGTGAGTTGT 20497

LsalLAC008020_C 20448 TATCTTCTTGGGTAGATAATCGATCGTCTTTCATTTGGATAGTGAGTTGT 20497

LsalCGN5271_CDS 20448 TATCTTCTTGGGTAGATAATCGATCGTCTTTCATTTGGATAGTGAGTTGT 20497

LsatLACWENDEL_C 20465 TTTTATAATAAGTCAAGTCAAGAATCATAAATGATACTTTCTGTTTTGTC 20514

LsatSalinas_CDS 20465 TTTTATAATAAGTCAAGTCAAGAATCATAAATGATACTTTCTGTTTTGTC 20514

LserUS96UC23_CD 20465 TTTTATAATAAGTCAAGTCAAGAATCATAAATGATACTTTCTGTTTTGTC 20514

LvirLAC006941_C 20480 TTTTATAATAAGTCAAGTCAAGAATCATAAATGATACTTTCTGTTTTGTC 20529

LserLAC005780_C 20498 TTTTATAATAAGTCAAGTCAAGAATCATAAATGATACTTTCTGTTTTGTC 20547

LsalLAC008020_C 20498 TTTTATAATAAGTCAAGTCAAGAATCATAAATGATACTTTCTGTTTTGTC 20547

LsalCGN5271_CDS 20498 TTTTATAATAAGTCAAGTCAAGAATCATAAATGATACTTTCTGTTTTGTC 20547

LsatLACWENDEL_C 20515 GAGCCTTGCTTTGGTCTCTGGTTTGATGGTTGTACGTGCTAAAAATCCGG 20564

LsatSalinas_CDS 20515 GAGCCTTGCTTTGGTCTCTGGTTTGATGGTTGTACGTGCTAAAAATCCGG 20564

LserUS96UC23_CD 20515 GAGCCTTGCTTTGGTCTCTGGTTTGATGGTTGTACGTGCTAAAAATCCGG 20564

LvirLAC006941_C 20530 GAGCCTTGCTTTGGTCTCTGGTTTGATGGTTGTACGTGCTAAAAATCCGG 20579

LserLAC005780_C 20548 GAGCCTTGCTTTGGTCTCTGGTTTGATGGTTGTACGTGCTAAAAATCCGG 20597

LsalLAC008020_C 20548 GAGCCTTGCTTTGGTCTCTGGTTTGATGGTTGTACGTGCTAAAAATCCGG 20597

LsalCGN5271_CDS 20548 GAGCCTTGCTTTGGTCTCTGGTTTGATGGTTGTACGTGCTAAAAATCCGG 20597

LsatLACWENDEL_C 20565 TACATTCCGTTTTGTTTCCCATCCCAGTCTTTCGCAACACTTCAGGTTTA 20614

LsatSalinas_CDS 20565 TACATTCCGTTTTGTTTCCCATCCCAGTCTTTCGCAACACTTCAGGTTTA 20614

LserUS96UC23_CD 20565 TACATTCCGTTTTGTTTCCCATCCCAGTCTTTCGCAACACTTCAGGTTTA 20614

LvirLAC006941_C 20580 TACATTCCGTTTTGTTTCCCATCCCAGTCTTTCGCAACACTTCAGGTTTA 20629

LserLAC005780_C 20598 TACATTCCGTTTTGTTTCCCATCCCAGTCTTTCGCAACACTTCAGGTTTA 20647

LsalLAC008020_C 20598 TACATTCCGTTTTGTTTCCCATCCCAGTCTTTCGCAACACTTCAGGTTTA 20647

LsalCGN5271_CDS 20598 TACATTCCGTTTTGTTTCCCATCCCAGTCTTTCGCAACACTTCAGGTTTA 20647

LsatLACWENDEL_C 20615 CTTCTTTTGTTAGGTCTCGACTTTTTCGCTATGATCTTCCCAGTAGTTCA 20664

LsatSalinas_CDS 20615 CTTCTTTTGTTAGGTCTCGACTTTTTCGCTATGATCTTCCCAGTAGTTCA 20664

LserUS96UC23_CD 20615 CTTCTTTTGTTAGGTCTCGACTTTTTCGCTATGATCTTCCCAGTAGTTCA 20664

LvirLAC006941_C 20630 CTTCTTTTGTTAGGTCTCGACTTTTTCGCTATGATCTTCCCAGTAGTTCA 20679

LserLAC005780_C 20648 CTTCTTTTGTTAGGTCTCGACTTTTTCGCTATGATCTTCCCAGTAGTTCA 20697

LsalLAC008020_C 20648 CTTCTTTTGTTAGGTCTCGACTTTTTCGCTATGATCTTCCCAGTAGTTCA 20697

LsalCGN5271_CDS 20648 CTTCTTTTGTTAGGTCTCGACTTTTTCGCTATGATCTTCCCAGTAGTTCA 20697

LsatLACWENDEL_C 20665 TATAGGAGCTATAGCCGTTTCATTCCTATTCGTTGTTATGATGTTCCATA 20714

LsatSalinas_CDS 20665 TATAGGAGCTATAGCCGTTTCATTCCTATTCGTTGTTATGATGTTCCATA 20714

LserUS96UC23_CD 20665 TATAGGAGCTATAGCCGTTTCATTCCTATTCGTTGTTATGATGTTCCATA 20714

LvirLAC006941_C 20680 TATAGGAGCTATAGCCGTTTCATTCCTATTCGTTGTTATGATGTTCCATA 20729

LserLAC005780_C 20698 TATAGGAGCTATAGCCGTTTCATTCCTATTCGTTGTTATGATGTTCCATA 20747

LsalLAC008020_C 20698 TATAGGAGCTATAGCCGTTTCATTCCTATTCGTTGTTATGATGTTCCATA 20747

LsalCGN5271_CDS 20698 TATAGGAGCTATAGCCGTTTCATTCCTATTCGTTGTTATGATGTTCCATA 20747

LsatLACWENDEL_C 20715 TTCAAATAGCGGAGATTCACGAAGAAGTATTGCGCTATTTACCAGTGAGT 20764

LsatSalinas_CDS 20715 TTCAAATAGCGGAGATTCACGAAGAAGTATTGCGCTATTTACCAGTGAGT 20764

LserUS96UC23_CD 20715 TTCAAATAGCGGAGATTCACGAAGAAGTATTGCGCTATTTACCAGTGAGT 20764

LvirLAC006941_C 20730 TTCAAATAGCGGAGATTCACGAAGAAGTATTGCGCTATTTACCAGTGAGT 20779

LserLAC005780_C 20748 TTCAAATAGCGGAGATTCACGAAGAAGTATTGCGCTATTTACCAGTGAGT 20797

LsalLAC008020_C 20748 TTCAAATAGCGGAGATTCACGAAGAAGTATTGCGCTATTTACCAGTGAGT 20797

LsalCGN5271_CDS 20748 TTCAAATAGCGGAGATTCACGAAGAAGTATTGCGCTATTTACCAGTGAGT 20797

LsatLACWENDEL_C 20765 GGTATTATTGGACTGATCTTTTGGTGGGAAATGTTCTTCATTTTAGATAA 20814

LsatSalinas_CDS 20765 GGTATTATTGGACTGATCTTTTGGTGGGAAATGTTCTTCATTTTAGATAA 20814

LserUS96UC23_CD 20765 GGTATTATTGGACTGATCTTTTGGTGGGAAATGTTCTTCATTTTAGATAA 20814

LvirLAC006941_C 20780 GGTATTATTGGACTGATCTTTTGGTGGGAAATGTTCTTCATTTTAGATAA 20829

LserLAC005780_C 20798 GGTATTATTGGACTGATCTTTTGGTGGGAAATGTTCTTCATTTTAGATAA 20847

LsalLAC008020_C 20798 GGTATTATTGGACTGATCTTTTGGTGGGAAATGTTCTTCATTTTAGATAA 20847

LsalCGN5271_CDS 20798 GGTATTATTGGACTGATCTTTTGGTGGGAAATGTTCTTCATTTTAGATAA 20847

LsatLACWENDEL_C 20815 TGAAAGCATTCCATTACTACCAACCCAAAGAAATACGACCTCTCTGAGAT 20864

LsatSalinas_CDS 20815 TGAAAGCATTCCATTACTACCAACCCAAAGAAATACGACCTCTCTGAGAT 20864

LserUS96UC23_CD 20815 TGAAAGCATTCCATTACTACCAACCCAAAGAAATACGACCTCTCTGAGAT 20864

LvirLAC006941_C 20830 TGAAAGCATTCCATTACTACCAACCCAAAGAAATACGACCTCTCTGAGAT 20879

LserLAC005780_C 20848 TGAAAGCATTCCATTACTACCAACCCAAAGAAATACGACCTCTCTGAGAT 20897

LsalLAC008020_C 20848 TGAAAGCATTCCATTACTACCAACCCAAAGAAATACGACCTCTCTGAGAT 20897

LsalCGN5271_CDS 20848 TGAAAGCATTCCATTACTACCAACCCAAAGAAATACGACCTCTCTGAGAT 20897

LsatLACWENDEL_C 20865 ATATGGTTTATGCCGGAAAGGTACGAAGTTGGACTAATTTGGAAACATTG 20914

LsatSalinas_CDS 20865 ATATGGTTTATGCCGGAAAGGTACGAAGTTGGACTAATTTGGAAACATTG 20914

LserUS96UC23_CD 20865 ATATGGTTTATGCCGGAAAGGTACGAAGTTGGACTAATTTGGAAACATTG 20914

LvirLAC006941_C 20880 ATATGGTTTATGCCGGAAAGGTACGAAGTTGGACTAATTTGGAAACATTG 20929

LserLAC005780_C 20898 ATATGGTTTATGCCGGAAAGGTACGAAGTTGGACTAATTTGGAAACATTG 20947

LsalLAC008020_C 20898 ATATGGTTTATGCCGGAAAGGTACGAAGTTGGACTAATTTGGAAACATTG 20947

LsalCGN5271_CDS 20898 ATATGGTTTATGCCGGAAAGGTACGAAGTTGGACTAATTTGGAAACATTG 20947

LsatLACWENDEL_C 20915 GGCAATTTACTTTATACCTACTATTCCGTCTGGTTTTTGGTTCCTAGTCT 20964

LsatSalinas_CDS 20915 GGCAATTTACTTTATACCTACTATTCCGTCTGGTTTTTGGTTCCTAGTCT 20964

LserUS96UC23_CD 20915 GGCAATTTACTTTATACCTACTATTCCGTCTGGTTTTTGGTTCCTAGTCT 20964

LvirLAC006941_C 20930 GGCAATTTACTTTATACCTACTATTCCGTCTGGTTTTTGGTTCCTAGTCT 20979

LserLAC005780_C 20948 GGCAATTTACTTTATACCTACTATTCCGTCTGGTTTTTGGTTCCTAGTCT 20997

LsalLAC008020_C 20948 GGCAATTTACTTTATACCTACTATTCCGTCTGGTTTTTGGTTCCTAGTCT 20997

LsalCGN5271_CDS 20948 GGCAATTTACTTTATACCTACTATTCCGTCTGGTTTTTGGTTCCTAGTCT 20997

LsatLACWENDEL_C 20965 GATTTTATTAGTAGCCATGATTGGGGCTATAGTACTGACTATGCATAGGA 21014

LsatSalinas_CDS 20965 GATTTTATTAGTAGCCATGATTGGGGCTATAGTACTGACTATGCATAGGA 21014

LserUS96UC23_CD 20965 GATTTTATTAGTAGCCATGATTGGGGCTATAGTACTGACTATGCATAGGA 21014

LvirLAC006941_C 20980 GATTTTATTAGTAGCCATGATTGGGGCTATAGTACTGACTATGCATAGGA 21029

LserLAC005780_C 20998 GATTTTATTAGTAGCCATGATTGGGGCTATAGTACTGACTATGCATAGGA 21047

LsalLAC008020_C 20998 GATTTTATTAGTAGCCATGATTGGGGCTATAGTACTGACTATGCATAGGA 21047

LsalCGN5271_CDS 20998 GATTTTATTAGTAGCCATGATTGGGGCTATAGTACTGACTATGCATAGGA 21047

LsatLACWENDEL_C 21015 CTACTAAGGTGAAAAGACAGGATGTATTCCGACGAAATGCTATTGATTCT 21064

LsatSalinas_CDS 21015 CTACTAAGGTGAAAAGACAGGATGTATTCCGACGAAATGCTATTGATTCT 21064

LserUS96UC23_CD 21015 CTACTAAGGTGAAAAGACAGGATGTATTCCGACGAAATGCTATTGATTCT 21064

LvirLAC006941_C 21030 CTACTAAGGTGAAAAGACAGGATGTATTCCGACGAAATGCTATTGATTCT 21079

LserLAC005780_C 21048 CTACTAAGGTGAAAAGACAGGATGTATTCCGACGAAATGCTATTGATTCT 21097

LsalLAC008020_C 21048 CTACTAAGGTGAAAAGACAGGATGTATTCCGACGAAATGCTATTGATTCT 21097

LsalCGN5271_CDS 21048 CTACTAAGGTGAAAAGACAGGATGTATTCCGACGAAATGCTATTGATTCT 21097

LsatLACWENDEL_C 21065 AGGAGGACTATAATGAGGGGGATGACAGATCTACTAAAGGAAAGTAGCTT 21114

LsatSalinas_CDS 21065 AGGAGGACTATAATGAGGGGGATGACAGATCTACTAAAGGAAAGTAGCTT 21114

LserUS96UC23_CD 21065 AGGAGGACTATAATGAGGGGGATGACAGATCTACTAAAGGAAAGTAGCTT 21114

LvirLAC006941_C 21080 AGGAGGACTATAATGAGGGGGATGACAGATCTACTAAAGGAAAGTAGCTT 21129

LserLAC005780_C 21098 AGGAGGACTATAATGAGGGGGATGACAGATCTACTAAAGGAAAGTAGCTT 21147

LsalLAC008020_C 21098 AGGAGGACTATAATGAGGGGGATGACAGATCTACTAAAGGAAAGTAGCTT 21147

LsalCGN5271_CDS 21098 AGGAGGACTATAATGAGGGGGATGACAGATCTACTAAAGGAAAGTAGCTT 21147

LsatLACWENDEL_C 21115 GATATTGGTTCGAATCCAATTCGTGAGATGGCCATCTTGGTCATATAGAT 21164

LsatSalinas_CDS 21115 GATATTGGTTCGAATCCAATTCGTGAGATGGCCATCTTGGTCATATAGAT 21164

LserUS96UC23_CD 21115 GATATTGGTTCGAATCCAATTCGTGAGATGGCCATCTTGGTCATATAGAT 21164

LvirLAC006941_C 21130 GATATTGGTTCGAATCCAATTCGTGAGATGGCCATCTTGGTCATATAGAT 21179

LserLAC005780_C 21148 GATATTGGTTCGAATCCAATTCGTGAGATGGCCATCTTGGTCATATAGAT 21197

LsalLAC008020_C 21148 GATATTGGTTCGAATCCAATTCGTGAGATGGCCATCTTGGTCATATAGAT 21197

LsalCGN5271_CDS 21148 GATATTGGTTCGAATCCAATTCGTGAGATGGCCATCTTGGTCATATAGAT 21197

LsatLACWENDEL_C 21165 GTCTTGACACCCTGATTTTCTTTTCTCATTTTCGAATGACTGTCCCATTC 21214

LsatSalinas_CDS 21165 GTCTTGACACCCTGATTTTCTTTTCTCATTTTCGAATGACTGTCCCATTC 21214

LserUS96UC23_CD 21165 GTCTTGACACCCTGATTTTCTTTTCTCATTTTCGAATGACTGTCCCATTC 21214

LvirLAC006941_C 21180 GTCTTGACACCCTGATTTTCTTTTCTCATTTTCGAATGACTGTCCCATTC 21229

LserLAC005780_C 21198 GTCTTGACACCCTGATTTTCTTTTCTCATTTTCGAATGACTGTCCCATTC 21247

LsalLAC008020_C 21198 GTCTTGACACCCTGATTTTATTTTCTCATTTTCGAATGACTGTCCCATTC 21247

LsalCGN5271_CDS 21198 GTCTTGACACCCTGATTTTATTTTCTCATTTTCGAATGACTGTCCCATTC 21247

LsatLACWENDEL_C 21215 CATTTTTGGTAGATGACGACTAGGAACGGGCAAATCAAAAATTTCACTTC 21264

LsatSalinas_CDS 21215 CATTTTTGGTAGATGACGACTAGGAACGGGCAAATCAAAAATTTCACTTC 21264

LserUS96UC23_CD 21215 CATTTTTGGTAGATGACGACTAGGAACGGGCAAATCAAAAATTTCACTTC 21264

LvirLAC006941_C 21230 CATTTTTGGTAGATGACGACTAGGAACGGGCAAATCAAAAATTTCACTTC 21279

LserLAC005780_C 21248 CATTTTTGGTAGATGACGACTAGGAACGGGCAAATCAAAAATTTCACTTC 21297

LsalLAC008020_C 21248 CATTTTTGGTAGATGACGACTAGGAACGGGCAAATCAAAAATTTCACTTC 21297

LsalCGN5271_CDS 21248 CATTTTTGGTAGATGACGACTAGGAACGGGCAAATCAAAAATTTCACTTC 21297

LsatLACWENDEL_C 21265 GAATTTCGGACCTCAACATCCTGCTGCTCATGGTGTTTCACGATCAGTAT 21314

LsatSalinas_CDS 21265 GAATTTCGGACCTCAACATCCTGCTGCTCATGGTGTTTCACGATCAGTAT 21314

LserUS96UC23_CD 21265 GAATTTCGGACCTCAACATCCTGCTGCTCATGGTGTTTCACGATCAGTAT 21314

LvirLAC006941_C 21280 GAATTTCGGACCTCAACATCCTGCTGCTCATGGTGTTTCACGATCAGTAT 21329

LserLAC005780_C 21298 GAATTTCGGACCTCAACATCCTGCTGCTCATGGTGTTTCACGATCAGTAT 21347

LsalLAC008020_C 21298 GAATTTCGGACCTCAACATCCTGCTGCTCATGGTGTTTCACGATCAGTAT 21347

LsalCGN5271_CDS 21298 GAATTTCGGACCTCAACATCCTGCTGCTCATGGTGTTTCACGATCAGTAT 21347

LsatLACWENDEL_C 21315 TGGAAATGAACGGAGAAGTGGTGGAACGTGCGGAACCACATATTGGATCA 21364

LsatSalinas_CDS 21315 TGGAAATGAACGGAGAAGTGGTGGAACGTGCGGAACCACATATTGGATCA 21364

LserUS96UC23_CD 21315 TGGAAATGAACGGAGAAGTGGTGGAACGTGCGGAACCACATATTGGATCA 21364

LvirLAC006941_C 21330 TGGAAATGAACGGAGAAGTGGTGGAACGTGCGGAACCACATATTGGATCA 21379

LserLAC005780_C 21348 TGGAAATGAACGGAGAAGTGGTGGAACGTGCGGAACCACATATTGGATCA 21397

LsalLAC008020_C 21348 TGGAAATGAACGGAGAAGTGGTGGAACGTGCGGAACCACATATTGGATCA 21397

LsalCGN5271_CDS 21348 TGGAAATGAACGGAGAAGTGGTGGAACGTGCGGAACCACATATTGGATCA 21397

LsatLACWENDEL_C 21365 CTCCAGAGAGGGACTGAGAAATTAATAGAGTACAAAACTTATCTTCAAGC 21414

LsatSalinas_CDS 21365 CTCCAGAGAGGGACTGAGAAATTAATAGAGTACAAAACTTATCTTCAAGC 21414

LserUS96UC23_CD 21365 CTCCAGAGAGGGACTGAGAAATTAATAGAGTACAAAACTTATCTTCAAGC 21414

LvirLAC006941_C 21380 CTCCAGAGAGGGACTGAGAAATTAATAGAGTACAAAACTTATCTTCAAGC 21429

LserLAC005780_C 21398 CTCCAGAGAGGGACTGAGAAATTAATAGAGTACAAAACTTATCTTCAAGC 21447

LsalLAC008020_C 21398 CTCCAGAGAGGGACTGAGAAATTAATAGAGTACAAAACTTATCTTCAAGC 21447

LsalCGN5271_CDS 21398 CTCCAGAGAGGGACTGAGAAATTAATAGAGTACAAAACTTATCTTCAAGC 21447

LsatLACWENDEL_C 21415 TTTACCTTATTCTGATCGTTCAGAGCCAGGGGTATGCTGGGATTCGCGAA 21464

LsatSalinas_CDS 21415 TTTACCTTATTCTGATCGTTCAGAGCCAGGGGTATGCTGGGATTCGCGAA 21464

LserUS96UC23_CD 21415 TTTACCTTATTCTGATCGTTCAGAGCCAGGGGTATGCTGGGATTCGCGAA 21464

LvirLAC006941_C 21430 TTTACCTTATTCTGATCGTTCAGAGCCAGGGGTATGCTGGGATTCGCGAA 21479

LserLAC005780_C 21448 TTTACCTTATTCTGATCGTTCAGAGCCAGGGGTATGCTGGGATTCGCGAA 21497

LsalLAC008020_C 21448 TTTACCTTATTCTGATCGTTCAGAGCCAGGGGTATGCTGGGATTCGCGAA 21497

LsalCGN5271_CDS 21448 TTTACCTTATTCTGATCGTTCAGAGCCAGGGGTATGCTGGGATTCGCGAA 21497

LsatLACWENDEL_C 21465 GAGCAGCACCTTACGATGTTCATGACCAATCGGATCCTGACGTACCAGTA 21514

LsatSalinas_CDS 21465 GAGCAGCACCTTACGATGTTCATGACCAATCGGATCCTGACGTACCAGTA 21514

LserUS96UC23_CD 21465 GAGCAGCACCTTACGATGTTCATGACCAATCGGATCCTGACGTACCAGTA 21514

LvirLAC006941_C 21480 GAGCAGCACCTTACGATGTTCATGACCAATCGGATCCTGACGTACCAGTA 21529

LserLAC005780_C 21498 GAGCAGCACCTTACGATGTTCATGACCAATCGGATCCTGACGTACCAGTA 21547

LsalLAC008020_C 21498 GAGCAGCACCTTACGATGTTCATGACCAATCGGATCCTGACGTACCAGTA 21547

LsalCGN5271_CDS 21498 GAGCAGCACCTTACGATGTTCATGACCAATCGGATCCTGACGTACCAGTA 21547

LsatLACWENDEL_C 21515 GGTACCAGAGGAGATCGCTATGATCGTTACTGTATTCGTATCGAAGAGAT 21564

LsatSalinas_CDS 21515 GGTACCAGAGGAGATCGCTATGATCGTTACTGTATTCGTATCGAAGAGAT 21564

LserUS96UC23_CD 21515 GGTACCAGAGGAGATCGCTATGATCGTTACTGTATTCGTATCGAAGAGAT 21564

LvirLAC006941_C 21530 GGTACCAGAGGAGATCGCTATGATCGTTACTGTATTCGTATCGAAGAGAT 21579

LserLAC005780_C 21548 GGTACCAGAGGAGATCGCTATGATCGTTACTGTATTCGTATCGAAGAGAT 21597

LsalLAC008020_C 21548 GGTACCAGAGGAGATCGCTATGATCGTTACTGTATTCGTATCGAAGAGAT 21597

LsalCGN5271_CDS 21548 GGTACCAGAGGAGATCGCTATGATCGTTACTGTATTCGTATCGAAGAGAT 21597

LsatLACWENDEL_C 21565 GCGACAAAGTGTTCGGATCATTGTGCAATGTCCTAATCAAATGCCTAGTG 21614

LsatSalinas_CDS 21565 GCGACAAAGTGTTCGGATCATTGTGCAATGTCCTAATCAAATGCCTAGTG 21614

LserUS96UC23_CD 21565 GCGACAAAGTGTTCGGATCATTGTGCAATGTCCTAATCAAATGCCTAGTG 21614

LvirLAC006941_C 21580 GCGACAAAGTGTTCGGATCATTGTGCAATGTCCTAATCAAATGCCTAGTG 21629

LserLAC005780_C 21598 GCGACAAAGTGTTCGGATCATTGTGCAATGTCCTAATCAAATGCCTAGTG 21647

LsalLAC008020_C 21598 GCGACAAAGTGTTCGGATCATTGTGCAATGTCCTAATCAAATGCCTAGTG 21647

LsalCGN5271_CDS 21598 GCGACAAAGTGTTCGGATCATTGTGCAATGTCCTAATCAAATGCCTAGTG 21647

LsatLACWENDEL_C 21615 GAATGATCAAAGCCGATGATCGTAAGCTATGTCCTCCATCACGATCTCGA 21664

LsatSalinas_CDS 21615 GAATGATCAAAGCCGATGATCGTAAGCTATGTCCTCCATCACGATCTCGA 21664

LserUS96UC23_CD 21615 GAATGATCAAAGCCGATGATCGTAAGCTATGTCCTCCATCACGATCTCGA 21664

LvirLAC006941_C 21630 GAATGATCAAAGCCGATGATCGTAAGCTATGTCCTCCATCACGATCTCGA 21679

LserLAC005780_C 21648 GAATGATCAAAGCCGATGATCGTAAGCTATGTCCTCCATCACGATCTCGA 21697

LsalLAC008020_C 21648 GAATGATCAAAGCCGATGATCGTAAGCTATGTCCTCCATCACGATCTCGA 21697

LsalCGN5271_CDS 21648 GAATGATCAAAGCCGATGATCGTAAGCTATGTCCTCCATCACGATCTCGA 21697

LsatLACWENDEL_C 21665 ATGAAACTATCCATGGAATCGTCAATTCACCATTTCGAACCTTATACAGA 21714

LsatSalinas_CDS 21665 ATGAAACTATCCATGGAATCGTCAATTCACCATTTCGAACCTTATACAGA 21714

LserUS96UC23_CD 21665 ATGAAACTATCCATGGAATCGTCAATTCACCATTTCGAACCTTATACAGA 21714

LvirLAC006941_C 21680 ATGAAACTATCCATGGAATCGTCAATTCACCATTTCGAACCTTATACAGA 21729

LserLAC005780_C 21698 ATGAAACTATCCATGGAATCGTCAATTCACCATTTCGAACCTTATACAGA 21747

LsalLAC008020_C 21698 ATGAAACTATCCATGGAATCGTCAATTCACCATTTCGAACCTTATACAGA 21747

LsalCGN5271_CDS 21698 ATGAAACTATCCATGGAATCGTCAATTCACCATTTCGAACCTTATACAGA 21747

LsatLACWENDEL_C 21715 AGGTTTTTCCGTACCAGCTCCTTCTACCTATACCGCAGTTGAAGCACCTA 21764

LsatSalinas_CDS 21715 AGGTTTTTCCGTACCAGCTCCTTCTACCTATACCGCAGTTGAAGCACCTA 21764

LserUS96UC23_CD 21715 AGGTTTTTCCGTACCAGCTCCTTCTACCTATACCGCAGTTGAAGCACCTA 21764

LvirLAC006941_C 21730 AGGTTTTTCCGTACCAGCTCCTTCTACCTATACCGCAGTTGAAGCACCTA 21779

LserLAC005780_C 21748 AGGTTTTTCCGTACCAGCTCCTTCTACCTATACCGCAGTTGAAGCACCTA 21797

LsalLAC008020_C 21748 AGGTTTTTCCGTACCAGCTCCTTCTACCTATACCGCAGTTGAAGCACCTA 21797

LsalCGN5271_CDS 21748 AGGTTTTTCCGTACCAGCTCCTTCTACCTATACCGCAGTTGAAGCACCTA 21797

LsatLACWENDEL_C 21765 AAGGAGAATTTGGTGTCTTTCTGGTCAGTAATGGAAGCAATCGTCCCTAC 21814

LsatSalinas_CDS 21765 AAGGAGAATTTGGTGTCTTTCTGGTCAGTAATGGAAGCAATCGTCCCTAC 21814

LserUS96UC23_CD 21765 AAGGAGAATTTGGTGTCTTTCTGGTCAGTAATGGAAGCAATCGTCCCTAC 21814

LvirLAC006941_C 21780 AAGGAGAATTTGGTGTCTTTCTGGTCAGTAATGGAAGCAATCGTCCCTAC 21829

LserLAC005780_C 21798 AAGGAGAATTTGGTGTCTTTCTGGTCAGTAATGGAAGCAATCGTCCCTAC 21847

LsalLAC008020_C 21798 AAGGAGAATTTGGTGTCTTTCTGGTCAGTAATGGAAGCAATCGTCCCTAC 21847

LsalCGN5271_CDS 21798 AAGGAGAATTTGGTGTCTTTCTGGTCAGTAATGGAAGCAATCGTCCCTAC 21847

LsatLACWENDEL_C 21815 CGTCGTAAAATAAGAGCACCTGGCTCTGCCCATTCACAAGGACTCGATTC 21864

LsatSalinas_CDS 21815 CGTCGTAAAATAAGAGCACCTGGCTCTGCCCATTCACAAGGACTCGATTC 21864

LserUS96UC23_CD 21815 CGTCGTAAAATAAGAGCACCTGGCTCTGCCCATTCACAAGGACTCGATTC 21864

LvirLAC006941_C 21830 CGTCGTAAAATAAGAGCACCTGGCTCTGCCCATTCACAAGGACTCGATTC 21879

LserLAC005780_C 21848 CGTCGTAAAATAAGAGCACCTGGCTCTGCCCATTCACAAGGACTCGATTC 21897

LsalLAC008020_C 21848 CGTCGTAAAATAAGAGCACCTGGCTCTGCCCATTCACAAGGACTCGATTC 21897

LsalCGN5271_CDS 21848 CGTCGTAAAATAAGAGCACCTGGCTCTGCCCATTCACAAGGACTCGATTC 21897

LsatLACWENDEL_C 21865 TATGTCCAAACATCACATGCCAGCAGATGTGGTCACCATCATAGGTACTC 21914

LsatSalinas_CDS 21865 TATGTCCAAACATCACATGCCAGCAGATGTGGTCACCATCATAGGTACTC 21914

LserUS96UC23_CD 21865 TATGTCCAAACATCACATGCCAGCAGATGTGGTCACCATCATAGGTACTC 21914

LvirLAC006941_C 21880 TATGTCCAAACATCACATGCCAGCAGATGTGGTCACCATCATAGGTACTC 21929

LserLAC005780_C 21898 TATGTCCAAACATCACATGCCAGCAGATGTGGTCACCATCATAGGTACTC 21947

LsalLAC008020_C 21898 TATGTCCAAACATCACATGCCAGCAGATGTGGTCACCATCATAGGTACTC 21947

LsalCGN5271_CDS 21898 TATGTCCAAACATCACATGCCAGCAGATGTGGTCACCATCATAGGTACTC 21947

LsatLACWENDEL_C 21915 AAGATATTGTGTCTGGAGAGGTGGATAGATAGATGGATAACCAATTCATT 21964

LsatSalinas_CDS 21915 AAGATATTGTGTCTGGAGAGGTGGATAGATAGATGGATAACCAATTCATT 21964

LserUS96UC23_CD 21915 AAGATATTGTGTCTGGAGAGGTGGATAGATAGATGGATAACCAATTCATT 21964

LvirLAC006941_C 21930 AAGATATTGTGTCTGGAGAGGTGGATAGATAGATGGATAACCAATTCATT 21979

LserLAC005780_C 21948 AAGATATTGTGTCTGGAGAGGTGGATAGATAGATGGATAACCAATTCATT 21997

LsalLAC008020_C 21948 AAGATATTGTGTCTGGAGAGGTGGATAGATAGATGGATAACCAATTCATT 21997

LsalCGN5271_CDS 21948 AAGATATTGTGTCTGGAGAGGTGGATAGATAGATGGATAACCAATTCATT 21997

LsatLACWENDEL_C 21965 TTCAAATATAGTTGGGAGACTTTACCCAAGAAATGGGTAAAAAAAATGGA 22014

LsatSalinas_CDS 21965 TTCAAATATAGTTGGGAGACTTTACCCAAGAAATGGGTAAAAAAAATGGA 22014

LserUS96UC23_CD 21965 TTCAAATATAGTTGGGAGACTTTACCCAAGAAATGGGTAAAAAAAATGGA 22014

LvirLAC006941_C 21980 TTCAAATATAGTTGGGAGACTTTACCCAAGAAATGGGTAAAAAAAATGGA 22029

LserLAC005780_C 21998 TTCAAATATAGTTGGGAGACTTTACCCAAGAAATGGGTAAAAAAAATGGA 22047

LsalLAC008020_C 21998 TTCAAATATAGTTGGGAGACTTTACCCAAGAAATGGGTAAAAAAAATGGA 22047

LsalCGN5271_CDS 21998 TTCAAATATAGTTGGGAGACTTTACCCAAGAAATGGGTAAAAAAAATGGA 22047

LsatLACWENDEL_C 22015 AAGATCGGAACATGGGAATAGCTCTGATACCAATACGGACTGCCCATTTC 22064

LsatSalinas_CDS 22015 AAGATCGGAACATGGGAATAGCTCTGATACCAATACGGACTGCCCATTTC 22064

LserUS96UC23_CD 22015 AAGATCGGAACATGGGAATAGCTCTGATACCAATACGGACTGCCCATTTC 22064

LvirLAC006941_C 22030 AAGATCGGAACATGGGAATAGCTCTGATACCAATACGGACTGCCCATTTC 22079

LserLAC005780_C 22048 AAGATCGGAACATGGGAATAGCTCTGATACCAATACGGACTGCCCATTTC 22097

LsalLAC008020_C 22048 AAGATCGGAACATGGGAATAGCTCTGATACCAATACGGACTGCCCATTTC 22097

LsalCGN5271_CDS 22048 AAGATCGGAACATGGGAATAGCTCTGATACCAATACGGACTGCCCATTTC 22097

LsatLACWENDEL_C 22065 AATTGTTGTGCTTTCTTAAATTGCATACCTATACAAGGGTTCAAGTTTCG 22114

LsatSalinas_CDS 22065 AATTGTTGTGCTTTCTTAAATTGCATACCTATACAAGGGTTCAAGTTTCG 22114

LserUS96UC23_CD 22065 AATTGTTGTGCTTTCTTAAATTGCATACCTATACAAGGGTTCAAGTTTCG 22114

LvirLAC006941_C 22080 AATTGTTGTGCTTTCTTAAATTGCATACCTATACAAGGGTTCAAGTTTCG 22129

LserLAC005780_C 22098 AATTGTTGTGCTTTCTTAAATTGCATACCTATACAAGGGTTCAAGTTTCG 22147

LsalLAC008020_C 22098 AATTGTTGTGCTTTCTTAAATTGCATACCTATACAAGGGTTCAAGTTTCG 22147

LsalCGN5271_CDS 22098 AATTGTTGTGCTTTCTTAAATTGCATACCTATACAAGGGTTCAAGTTTCG 22147

LsatLACWENDEL_C 22115 ATCGATATTTGCGGAGTTGATCATCCCTCTCGAAAACGAAGATTTGAAGT 22164

LsatSalinas_CDS 22115 ATCGATATTTGCGGAGTTGATCATCCCTCTCGAAAACGAAGATTTGAAGT 22164

LserUS96UC23_CD 22115 ATCGATATTTGCGGAGTTGATCATCCCTCTCGAAAACGAAGATTTGAAGT 22164

LvirLAC006941_C 22130 ATCGATATTTGCGGAGTTGATCATCCCTCTCGAAAACGAAGATTTGAAGT 22179

LserLAC005780_C 22148 ATCGATATTTGCGGAGTTGATCATCCCTCTCGAAAACGAAGATTTGAAGT 22197

LsalLAC008020_C 22148 ATCGATATTTGCGGAGTTGATCATCCCTCTCGAAAACGAAGATTTGAAGT 22197

LsalCGN5271_CDS 22148 ATCGATATTTGCGGAGTTGATCATCCCTCTCGAAAACGAAGATTTGAAGT 22197

LsatLACWENDEL_C 22165 GGTCTATAATTTACTGAGTACTCGGTATAACTCACGCATTCGTGTACAAA 22214

LsatSalinas_CDS 22165 GGTCTATAATTTACTGAGTACTCGGTATAACTCACGCATTCGTGTACAAA 22214

LserUS96UC23_CD 22165 GGTCTATAATTTACTGAGTACTCGGTATAACTCACGCATTCGTGTACAAA 22214

LvirLAC006941_C 22180 GGTCTATAATTTACTGAGTACTCGGTATAACTCACGCATTCGTGTACAAA 22229

LserLAC005780_C 22198 GGTCTATAATTTACTGAGTACTCGGTATAACTCACGCATTCGTGTACAAA 22247

LsalLAC008020_C 22198 GGTCTATAATTTACTGAGTACTCGGTATAACTCACGCATTCGTGTACAAA 22247

LsalCGN5271_CDS 22198 GGTCTATAATTTACTGAGTACTCGGTATAACTCACGCATTCGTGTACAAA 22247

LsatLACWENDEL_C 22215 CCAGTGCAGACGAAGTAACACGAATATCCCCGGTAGTCAGTCCATTTCCA 22264

LsatSalinas_CDS 22215 CCAGTGCAGACGAAGTAACACGAATATCCCCGGTAGTCAGTCCATTTCCA 22264

LserUS96UC23_CD 22215 CCAGTGCAGACGAAGTAACACGAATATCCCCGGTAGTCAGTCCATTTCCA 22264

LvirLAC006941_C 22230 CCAGTGCAGACGAAGTAACACGAATATCCCCGGTAGTCAGTCCATTTCCA 22279

LserLAC005780_C 22248 CCAGTGCAGACGAAGTAACACGAATATCCCCGGTAGTCAGTCCATTTCCA 22297

LsalLAC008020_C 22248 CCAGTGCAGACGAAGTAACACGAATATCCCCGGTAGTCAGTCCATTTCCA 22297

LsalCGN5271_CDS 22248 CCAGTGCAGACGAAGTAACACGAATATCCCCGGTAGTCAGTCCATTTCCA 22297

LsatLACWENDEL_C 22265 TCAGCCGGCCGGTGGGAGCGAGAAGTTTGGGATATGTTTGGTGTTTCTTC 22314

LsatSalinas_CDS 22265 TCAGCCGGCCGGTGGGAGCGAGAAGTTTGGGATATGTTTGGTGTTTCTTC 22314

LserUS96UC23_CD 22265 TCAGCCGGCCGGTGGGAGCGAGAAGTTTGGGATATGTTTGGTGTTTCTTC 22314

LvirLAC006941_C 22280 TCAGCCGGCCGGTGGGAGCGAGAAGTTTGGGATATGTTTGGTGTTTCTTC 22329

LserLAC005780_C 22298 TCAGCCGGCCGGTGGGAGCGAGAAGTTTGGGATATGTTTGGTGTTTCTTC 22347

LsalLAC008020_C 22298 TCAGCCGGCCGGTGGGAGCGAGAAGTTTGGGATATGTTTGGTGTTTCTTC 22347

LsalCGN5271_CDS 22298 TCAGCCGGCCGGTGGGAGCGAGAAGTTTGGGATATGTTTGGTGTTTCTTC 22347

LsatLACWENDEL_C 22315 CATCAATCATCCAGATCTACGCCGTATATCAACAGATTATGGTTTCGAGG 22364

LsatSalinas_CDS 22315 CATCAATCATCCAGATCTACGCCGTATATCAACAGATTATGGTTTCGAGG 22364

LserUS96UC23_CD 22315 CATCAATCATCCAGATCTACGCCGTATATCAACAGATTATGGTTTCGAGG 22364

LvirLAC006941_C 22330 CATCAATCATCCAGATCTACGCCGTATATCAACAGATTATGGTTTCGAGG 22379

LserLAC005780_C 22348 CATCAATCATCCAGATCTACGCCGTATATCAACAGATTATGGTTTCGAGG 22397

LsalLAC008020_C 22348 CATCAATCATCCAGATCTACGCCGTATATCAACAGATTATGGTTTCGAGG 22397

LsalCGN5271_CDS 22348 CATCAATCATCCAGATCTACGCCGTATATCAACAGATTATGGTTTCGAGG 22397

LsatLACWENDEL_C 22365 GTCATCCATTACGAAAAGACCTTCCTCTGAGTGGATATGTGGAAGTACGC 22414

LsatSalinas_CDS 22365 GTCATCCATTACGAAAAGACCTTCCTCTGAGTGGATATGTGGAAGTACGC 22414

LserUS96UC23_CD 22365 GTCATCCATTACGAAAAGACCTTCCTCTGAGTGGATATGTGGAAGTACGC 22414

LvirLAC006941_C 22380 GTCATCCATTACGAAAAGACCTTCCTCTGAGTGGATATGTGGAAGTACGC 22429

LserLAC005780_C 22398 GTCATCCATTACGAAAAGACCTTCCTCTGAGTGGATATGTGGAAGTACGC 22447

LsalLAC008020_C 22398 GTCATCCATTACGAAAAGACCTTCCTCTGAGTGGATATGTGGAAGTACGC 22447

LsalCGN5271_CDS 22398 GTCATCCATTACGAAAAGACCTTCCTCTGAGTGGATATGTGGAAGTACGC 22447

LsatLACWENDEL_C 22415 TATGATGATCCAGAGAAACGTGTGGTTTCTGAACCCATTGAGATGACCCA 22464

LsatSalinas_CDS 22415 TATGATGATCCAGAGAAACGTGTGGTTTCTGAACCCATTGAGATGACCCA 22464

LserUS96UC23_CD 22415 TATGATGATCCAGAGAAACGTGTGGTTTCTGAACCCATTGAGATGACCCA 22464

LvirLAC006941_C 22430 TATGATGATCCAGAGAAACGTGTGGTTTCTGAACCCATTGAGATGACCCA 22479

LserLAC005780_C 22448 TATGATGATCCAGAGAAACGTGTGGTTTCTGAACCCATTGAGATGACCCA 22497

LsalLAC008020_C 22448 TATGATGATCCAGAGAAACGTGTGGTTTCTGAACCCATTGAGATGACCCA 22497

LsalCGN5271_CDS 22448 TATGATGATCCAGAGAAACGTGTGGTTTCTGAACCCATTGAGATGACCCA 22497

LsatLACWENDEL_C 22465 AGAATTTCGCTATTTCGATTCTGCTAGTCCTTGGGAACAGCGTAGCGACG 22514

LsatSalinas_CDS 22465 AGAATTTCGCTATTTCGATTCTGCTAGTCCTTGGGAACAGCGTAGCGACG 22514

LserUS96UC23_CD 22465 AGAATTTCGCTATTTCGATTCTGCTAGTCCTTGGGAACAGCGTAGCGACG 22514

LvirLAC006941_C 22480 AGAATTTCGCTATTTCGATTCTGCTAGTCCTTGGGAACAGCGTAGCGACG 22529

LserLAC005780_C 22498 AGAATTTCGCTATTTCGATTCTGCTAGTCCTTGGGAACAGCGTAGCGACG 22547

LsalLAC008020_C 22498 AGAATTTCGCTATTTCGATTCTGCTAGTCCTTGGGAACAGCGTAGCGACG 22547

LsalCGN5271_CDS 22498 AGAATTTCGCTATTTCGATTCTGCTAGTCCTTGGGAACAGCGTAGCGACG 22547

LsatLACWENDEL_C 22515 GATAAATGCCATTCGGAAGAAGTCTTCTACAGAGGGAAAGCCTGTTACGA 22564

LsatSalinas_CDS 22515 GATAAATGCCATTCGGAAGAAGTCTTCTACAGAGGGAAAGCCTGTTACGA 22564

LserUS96UC23_CD 22515 GATAAATGCCATTCGGAAGAAGTCTTCTACAGAGGGAAAGCCTGTTACGA 22564

LvirLAC006941_C 22530 GATAAATGCCATTCGGAAGAAGTCTTCTACAGAGGGAAAGCCTGTTACGA 22579

LserLAC005780_C 22548 GATAAATGCCATTCGGAAGAAGTCTTCTACAGAGGGAAAGCCTGTTACGA 22597

LsalLAC008020_C 22548 GATAAATGCCATTCGGAAGAAGTCTTCTACAGAGGGAAAGCCTGTTACGA 22597

LsalCGN5271_CDS 22548 GATAAATGCCATTCGGAAGAAGTCTTCTACAGAGGGAAAGCCTGTTACGA 22597

LsatLACWENDEL_C 22565 GTAAGTGGAGAGGAAAGATCTCCAGAGATTCTTATCTCATTCCATTCCAG 22614

LsatSalinas_CDS 22565 GTAAGTGGAGAGGAAAGATCTCCAGAGATTCTTATCTCATTCCATTCCAG 22614

LserUS96UC23_CD 22565 GTAAGTGGAGAGGAAAGATCTCCAGAGATTCTTATCTCATTCCATTCCAG 22614

LvirLAC006941_C 22580 GTAAGTGGAGAGGAAAGATCTCCAGAGATTCTTATCTCATTCCATTCCAG 22629

LserLAC005780_C 22598 GTAAGTGGAGAGGAAAGATCTCCAGAGATTCTTATCTCATTCCATTCCAG 22647

LsalLAC008020_C 22598 GTAAGTGGAGAGGAAAGATCTCCAGAGATTCTTATCTCATTCCATTCCAG 22647

LsalCGN5271_CDS 22598 GTAAGTGGAGAGGAAAGATCTCCAGAGATTCTTATCTCATTCCATTCCAG 22647

LsatLACWENDEL_C 22615 TGGCTCAACCAGTAACCAATGGCGAAAACTAAAAAATCCATGGTTTCCCG 22664

LsatSalinas_CDS 22615 TGGCTCAACCAGTAACCAATGGCGAAAACTAAAAAATCCATGGTTTCCCG 22664

LserUS96UC23_CD 22615 TGGCTCAACCAGTAACCAATGGCGAAAACTAAAAAATCCATGGTTTCCCG 22664

LvirLAC006941_C 22630 TGGCTCAACCAGTAACCAATGGCGAAAACTAAAAAATCCATGGTTTCCCG 22679

LserLAC005780_C 22648 TGGCTCAACCAGTAACCAATGGCGAAAACTAAAAAATCCATGGTTTCCCG 22697

LsalLAC008020_C 22648 TGGCTCAACCAGTAACCAATGGCGAAAACTAAAAAATCCATGGTTTCCCG 22697

LsalCGN5271_CDS 22648 TGGCTCAACCAGTAACCAATGGCGAAAACTAAAAAATCCATGGTTTCCCG 22697

LsatLACWENDEL_C 22665 GTAGAACCCTATTTCGCCCAAGTTGTTTCAGAACCGGAAAAAAGAAGCGG 22714

LsatSalinas_CDS 22665 GTAGAACCCTATTTCGCCCAAGTTGTTTCAGAACCGGAAAAAAGAAGCGG 22714

LserUS96UC23_CD 22665 GTAGAACCCTATTTCGCCCAAGTTGTTTCAGAACCGGAAAAAAGAAGCGG 22714

LvirLAC006941_C 22680 GTAGAACCCTATTTCGCCCAAGTTGTTTCAGAACCGGAAAAAAGAAGCGG 22729

LserLAC005780_C 22698 GTAGAACCCTATTTCGCCCAAGTTGTTTCAGAACCGGAAAAAAGAAGCGG 22747

LsalLAC008020_C 22698 GTAGAACCCTATTTCGCCCAAGTTGTTTCAGAACCGGAAAAAAGAAGCGG 22747

LsalCGN5271_CDS 22698 GTAGAACCCTATTTCGCCCAAGTTGTTTCAGAACCGGAAAAAAGAAGCGG 22747

LsatLACWENDEL_C 22715 TTTTTCGCACAGCTTGCGCATAGTGCAGGTCCCACTTGTATATCGTATTT 22764

LsatSalinas_CDS 22715 TTTTTCGCACAGCTTGCGCATAGTGCAGGTCCCACTTGTATATCGTATTT 22764

LserUS96UC23_CD 22715 TTTTTCGCACAGCTTGCGCATAGTGCAGGTCCCACTTGTATATCGTATTT 22764

LvirLAC006941_C 22730 TTTTTCGCACAGCTTGCGCATAGTGCAGGTCCCACTTGTATATCGTATTT 22779

LserLAC005780_C 22748 TTTTTCGCACAGCTTGCGCATAGTGCAGGTCCCACTTGTATATCGTATTT 22797

LsalLAC008020_C 22748 TTTTTCGCACAGCTTGCGCATAGTGCAGGTCCCACTTGTATATCGTATTT 22797

LsalCGN5271_CDS 22748 TTTTTCGCACAGCTTGCGCATAGTGCAGGTCCCACTTGTATATCGTATTT 22797

LsatLACWENDEL_C 22765 GGCCGAAGAAGCATCGGACAGGTTGGAGTTCTTACCTTCTTGGGATTCCA 22814

LsatSalinas_CDS 22765 GGCCGAAGAAGCATCGGACAGGTTGGAGTTCTTACCTTCTTGGGATTCCA 22814

LserUS96UC23_CD 22765 GGCCGAAGAAGCATCGGACAGGTTGGAGTTCTTACCTTCTTGGGATTCCA 22814

LvirLAC006941_C 22780 GGCCGAAGAAGCATCGGACAGGTTGGAGTTCTTACCTTCTTGGGATTCCA 22829

LserLAC005780_C 22798 GGCCGAAGAAGCATCGGACAGGTTGGAGTTCTTACCTTCTTGGGATTCCA 22847

LsalLAC008020_C 22798 GGCCGAAGAAGCATCGGACAGGTTGGAGTTCTTACCTTCTTGGGATTCCA 22847

LsalCGN5271_CDS 22798 GGCCGAAGAAGCATCGGACAGGTTGGAGTTCTTACCTTCTTGGGATTCCA 22847

LsatLACWENDEL_C 22815 TGGACCAAGATCTGCTTTTATTATATGGGCAATACCGATCTACTTTAGTA 22864

LsatSalinas_CDS 22815 TGGACCAAGATCTGCTTTTATTATATGGGCAATACCGATCTACTTTAGTA 22864

LserUS96UC23_CD 22815 TGGACCAAGATCTGCTTTTATTATATGGGCAATACCGATCTACTTTAGTA 22864

LvirLAC006941_C 22830 TGGACCAAGATCTGCTTTTATTATATGGGCAATACCGATCTACTTTAGTA 22879

LserLAC005780_C 22848 TGGACCAAGATCTGCTTTTATTATATGGGCAATACCGATCTACTTTAGTA 22897

LsalLAC008020_C 22848 TGGACCAAGATCTGCTTTTATTATATGGGCAATACCGATCTACTTTAGTA 22897

LsalCGN5271_CDS 22848 TGGACCAAGATCTGCTTTTATTATATGGGCAATACCGATCTACTTTAGTA 22897

LsatLACWENDEL_C 22865 GATCATATGGATGTAGAAAAAGCTTCTCATTTTGATGAATTGGAAACATC 22914

LsatSalinas_CDS 22865 GATCATATGGATGTAGAAAAAGCTTCTCATTTTGATGAATTGGAAACATC 22914

LserUS96UC23_CD 22865 GATCATATGGATGTAGAAAAAGCTTCTCATTTTGATGAATTGGAAACATC 22914

LvirLAC006941_C 22880 GATCATATGGATGTAGAAAAAGCTTCTCATTTTGATGAATTGGAAACATC 22929

LserLAC005780_C 22898 GATCATATGGATGTAGAAAAAGCTTCTCATTTTGATGAATTGGAAACATC 22947

LsalLAC008020_C 22898 GATCATATGGATGTAGAAAAAGCTTCTCATTTTGATGAATTGGAAACATC 22947

LsalCGN5271_CDS 22898 GATCATATGGATGTAGAAAAAGCTTCTCATTTTGATGAATTGGAAACATC 22947

LsatLACWENDEL_C 22915 TCTTTTCCATTTCTATTTACCCAGTTCATATCTTTGTTTCGTGTGTTCCC 22964

LsatSalinas_CDS 22915 TCTTTTCCATTTCTATTTACCCAGTTCATATCTTTGTTTCGTGTGTTCCC 22964

LserUS96UC23_CD 22915 TCTTTTCCATTTCTATTTACCCAGTTCATATCTTTGTTTCGTGTGTTCCC 22964

LvirLAC006941_C 22930 TCTTTTCCATTTCTATTTACCCAGTTCATATCTTTGTTTCGTGTGTTCCC 22979

LserLAC005780_C 22948 TCTTTTCCATTTCTATTTACCCAGTTCATATCTTTGTTTCGTGTGTTCCC 22997

LsalLAC008020_C 22948 TCTTTTCCATTTCTATTTACCCAGTTCATATCTTTGTTTCGTGTGTTCCC 22997

LsalCGN5271_CDS 22948 TCTTTTCCATTTCTATTTACCCAGTTCATATCTTTGTTTCGTGTGTTCCC 22997

LsatLACWENDEL_C 22965 CGGAGGAATTCGATCTCTTCAATCTCGG---GATACCACCTAAATAAATG 23011

LsatSalinas_CDS 22965 CGGAGGAATTCGATCTCTTCAATCTCGG---GATACCACCTAAATAAATG 23011

LserUS96UC23_CD 22965 CGGAGGAATTCGATCTCTTCAATCTCGG---GATACCACCTAAATAAATG 23011

LvirLAC006941_C 22980 CGGAGGAATTCGATCTCTTCAATCTCGG---GATACCACCTAAATAAATG 23026

LserLAC005780_C 22998 CGGAGGAATTCGATCTCTTCAATCTCGG---GATACCACCTAAATAAATG 23044

LsalLAC008020_C 22998 CGGAGGAATTCGATCTCTTCAATCTCGGCGGGATACCACCTAAATAAATG 23047

LsalCGN5271_CDS 22998 CGGAGGAATTCGATCTCTTCAATCTCGGCGGGATACCACCTAAATAAATG 23047

LsatLACWENDEL_C 23012 TTTACACTCAATTTTCATTACGAAGATGTATCACGTCAGGATCCGTTGCT 23061

LsatSalinas_CDS 23012 TTTACACTCAATTTTCATTACGAAGATGTATCACGTCAGGATCCGTTGCT 23061

LserUS96UC23_CD 23012 TTTACACTCAATTTTCATTACGAAGATGTATCACGTCAGGATCCGTTGCT 23061

LvirLAC006941_C 23027 TTTACACTCAATTTTCATTACGAAGATGTATCACGTCAGGATCCGTTGCT 23076

LserLAC005780_C 23045 TTTACACTCAATTTTCATTACGAAGATGTATCACGTCAGGATCCGTTGCT 23094

LsalLAC008020_C 23048 TTTACACTCAATTTTCATTACGAAGATGTATCACGTCAGGATCCGTTGCT 23097

LsalCGN5271_CDS 23048 TTTACACTCAATTTTCATTACGAAGATGTATCACGTCAGGATCCGTTGCT 23097

LsatLACWENDEL_C 23062 CAAACCGAATCACGCCAACGTTATGGAAGTTCCTGGATCGTGTAAAATAA 23111

LsatSalinas_CDS 23062 CAAACCGAATCACGCCAACGTTATGGAAGTTCCTGGATCGTGTAAAATAA 23111

LserUS96UC23_CD 23062 CAAACCGAATCACGCCAACGTTATGGAAGTTCCTGGATCGTGTAAAATAA 23111

LvirLAC006941_C 23077 CAAACCGAATCACGCCAACGTTATGGAAGTTCCTGGATCGTGTAAAATAA 23126

LserLAC005780_C 23095 CAAACCGAATCACGCCAACGTTATGGAAGTTCCTGGATCGTGTAAAATAA 23144

LsalLAC008020_C 23098 CAAACCGAATCACGCCAACGTTATGGAAGTTCCTGGATCGTGTAAAATAA 23147

LsalCGN5271_CDS 23098 CAAACCGAATCACGCCAACGTTATGGAAGTTCCTGGATCGTGTAAAATAA 23147

LsatLACWENDEL_C 23112 GAGTAGTACCAAAGGCAGCACCCTCTGATTTCATAATAAAAAATGGAAAA 23161

LsatSalinas_CDS 23112 GAGTAGTACCAAAGGCAGCACCCTCTGATTTCATAATAAAAAATGGAAAA 23161

LserUS96UC23_CD 23112 GAGTAGTACCAAAGGCAGCACCCTCTGATTTCATAATAAAAAATGGAAAA 23161

LvirLAC006941_C 23127 GAGTAGTACCAAAGGCAGCACCCTCTGATTTCATAATAAAAAATGGAAAA 23176

LserLAC005780_C 23145 GAGTAGTACCAAAGGCAGCACCCTCTGATTTCATAATAAAAAATGGAAAA 23194

LsalLAC008020_C 23148 GAGTAGTACCAAAGGCAGCACCCTCTGATTTCATAATAAAAAATGGAAAA 23197

LsalCGN5271_CDS 23148 GAGTAGTACCAAAGGCAGCACCCTCTGATTTCATAATAAAAAATGGAAAA 23197

LsatLACWENDEL_C 23162 TTGGCTATGGAGATTCCGTGCGGTCAGAAATTAATACAGACACAAAGGGC 23211

LsatSalinas_CDS 23162 TTGGCTATGGAGATTCCGTGCGGTCAGAAATTAATACAGACACAAAGGGC 23211

LserUS96UC23_CD 23162 TTGGCTATGGAGATTCCGTGCGGTCAGAAATTAATACAGACACAAAGGGC 23211

LvirLAC006941_C 23177 TTGGCTATGGAGATTCCGTGCGGTCAGAAATTAATACAGACACAAAGGGC 23226

LserLAC005780_C 23195 TTGGCTATGGAGATTCCGTGCGGTCAGAAATTAATACAGACACAAAGGGC 23244

LsalLAC008020_C 23198 TTGGCTATGGAGATTCCGTGCGGTCAGAAATTAATACAGACACAAAGGGC 23247

LsalCGN5271_CDS 23198 TTGGCTATGGAGATTCCGTGCGGTCAGAAATTAATACAGACACAAAGGGC 23247

LsatLACWENDEL_C 23212 TTCGACAGGAAAGGAGTTTCGATCCAATCCATTCTTGGGAAAAAATCAAG 23261

LsatSalinas_CDS 23212 TTCGACAGGAAAGGAGTTTCGATCCAATCCATTCTTGGGAAAAAATCAAG 23261

LserUS96UC23_CD 23212 TTCGACAGGAAAGGAGTTTCGATCCAATCCATTCTTGGGAAAAAATCAAG 23261

LvirLAC006941_C 23227 TTCGACAGGAAAGGAGTTTCGATCCAATCCATTCTTGGGAAAAAATCAAG 23276

LserLAC005780_C 23245 TTCGACAGGAAAGGAGTTTCGATCCAATCCATTCTTGGGAAAAAATCAAG 23294

LsalLAC008020_C 23248 TTCGACAGGAAAGGAGTTTCGATCCAATCCATTCTTGGGAAAAAATCAAG 23297

LsalCGN5271_CDS 23248 TTCGACAGGAAAGGAGTTTCGATCCAATCCATTCTTGGGAAAAAATCAAG 23297

LsatLACWENDEL_C 23262 ACAAAAAAGGATATGTCAGTGACCTAGCACGGCAAAGCACTCTCCGAGGG 23311

LsatSalinas_CDS 23262 ACAAAAAAGGATATGTCAGTGACCTAGCACGGCAAAGCACTCTCCGAGGG 23311

LserUS96UC23_CD 23262 ACAAAAAAGGATATGTCAGTGACCTAGCACGGCAAAGCACTCTCCGAGGG 23311

LvirLAC006941_C 23277 ACAAAAAAGGATATGTCAGTGACCTAGCACGGCAAAGCACTCTCCGAGGG 23326

LserLAC005780_C 23295 ACAAAAAAGGATATGTCAGTGACCTAGCACGGCAAAGCACTCTCCGAGGG 23344

LsalLAC008020_C 23298 ACAAAAAAGGATATGTCAGTGACCTAGCACGGCAAAGCACTCTCCGAGGG 23347

LsalCGN5271_CDS 23298 ACAAAAAAGGATATGTCAGTGACCTAGCACGGCAAAGCACTCTCCGAGGG 23347

LsatLACWENDEL_C 23312 CATGGAATGTCTCATTTTTTGGTAAGAATCTCCGCAGTAATGTCTCTGTT 23361

LsatSalinas_CDS 23312 CATGGAATGTCTCATTTTTTGGTAAGAATCTCCGCAGTAATGTCTCTGTT 23361

LserUS96UC23_CD 23312 CATGGAATGTCTCATTTTTTGGTAAGAATCTCCGCAGTAATGTCTCTGTT 23361

LvirLAC006941_C 23327 CATGGAATGTCTCATTTTTTGGTAAGAATCTCCGCAGTAATGTCTCTGTT 23376

LserLAC005780_C 23345 CATGGAATGTCTCATTTTTTGGTAAGAATCTCCGCAGTAATGTCTCTGTT 23394

LsalLAC008020_C 23348 CATGGAATGTCTCATTTTTTGGTAAGAATCTCCGCAGTAATGTCTCTGTT 23397

LsalCGN5271_CDS 23348 CATGGAATGTCTCATTTTTTGGTAAGAATCTCCGCAGTAATGTCTCTGTT 23397

LsatLACWENDEL_C 23362 AGATTTTCCGGTCGAAATACGGGAAAAGTCCATTCAATTCTTGATGGAAA 23411

LsatSalinas_CDS 23362 AGATTTTCCGGTCGAAATACGGGAAAAGTCCATTCAATTCTTGATGGAAA 23411

LserUS96UC23_CD 23362 AGATTTTCCGGTCGAAATACGGGAAAAGTCCATTCAATTCTTGATGGAAA 23411

LvirLAC006941_C 23377 AGATTTTCCGGTCGAAATACGGGAAAAGTCCATTCAATTCTTGATGGAAA 23426

LserLAC005780_C 23395 AGATTTTCCGGTCGAAATACGGGAAAAGTCCATTCAATTCTTGATGGAAA 23444

LsalLAC008020_C 23398 AGATTTTCCGGTCGAAATACGGGAAAAGTCCATTCAATTCTTGATGGAAA 23447

LsalCGN5271_CDS 23398 AGATTTTCCGGTCGAAATACGGGAAAAGTCCATTCAATTCTTGATGGAAA 23447

LsatLACWENDEL_C 23412 TGGAGTTTTGCGAATTCTCCCCGGAACTGGAAGATCATTTCGAGATCTTC 23461

LsatSalinas_CDS 23412 TGGAGTTTTGCGAATTCTCCCCGGAACTGGAAGATCATTTCGAGATCTTC 23461

LserUS96UC23_CD 23412 TGGAGTTTTGCGAATTCTCCCCGGAACTGGAAGATCATTTCGAGATCTTC 23461

LvirLAC006941_C 23427 TGGAGTTTTGCGAATTCTCCCCGGAACTGGAAGATCATTTCGAGATCTTC 23476

LserLAC005780_C 23445 TGGAGTTTTGCGAATTCTCCCCGGAACTGGAAGATCATTTCGAGATCTTC 23494

LsalLAC008020_C 23448 TGGAGTTTTGCGAATTCTCCCCGGAACTGGAAGATCATTTCGAGATCTTC 23497

LsalCGN5271_CDS 23448 TGGAGTTTTGCGAATTCTCCCCGGAACTGGAAGATCATTTCGAGATCTTC 23497

LsatLACWENDEL_C 23462 GAACATATTCGAGGGTTCAATGTCACTATTGTAACTTCGGCCAACACACA 23511

LsatSalinas_CDS 23462 GAACATATTCGAGGGTTCAATGTCACTATTGTAACTTCGGCCAACACACA 23511

LserUS96UC23_CD 23462 GAACATATTCGAGGGTTCAATGTCACTATTGTAACTTCGGCCAACACACA 23511

LvirLAC006941_C 23477 GAACATATTCGAGGGTTCAATGTCACTATTGTAACTTCGGCCAACACACA 23526

LserLAC005780_C 23495 GAACATATTCGAGGGTTCAATGTCACTATTGTAACTTCGGCCAACACACA 23544

LsalLAC008020_C 23498 GAACATATTCGAGGGTTCAATGTCACTATTGTAACTTCGGCCAACACACA 23547

LsalCGN5271_CDS 23498 GAACATATTCGAGGGTTCAATGTCACTATTGTAACTTCGGCCAACACACA 23547

LsatLACWENDEL_C 23512 AGATGAGACTTTACCACCGTGGAGCGGCTTTTTTCAAAAAGATGAGGGGG 23561

LsatSalinas_CDS 23512 AGATGAGACTTTACCACCGTGGAGCGGCTTTTTTCAAAAAGATGAGGGGG 23561

LserUS96UC23_CD 23512 AGATGAGACTTTACCACCGTGGAGCGGCTTTTTTCAAAAAGATGAGGGGG 23561

LvirLAC006941_C 23527 AGATGAGACTTTACCACCGTGGAGCGGCTTTTTTCAAAAAGATGAGGGGG 23576

LserLAC005780_C 23545 AGATGAGACTTTACCACCGTGGAGCGGCTTTTTTCAAAAAGATGAGGGGG 23594

LsalLAC008020_C 23548 AGATGAGACTTTACCACCGTGGAGCGGCTTTTTTCAAAAAGATGAGGGGG 23597

LsalCGN5271_CDS 23548 AGATGAGACTTTACCACCGTGGAGCGGCTTTTTTCAAAAAGATGAGGGGG 23597

LsatLACWENDEL_C 23562 AAAGTCAGTAAATGCCTACATTAAATCAATTGATTCGTCATGGTAGAGAA 23611

LsatSalinas_CDS 23562 AAAGTCAGTAAATGCCTACATTAAATCAATTGATTCGTCATGGTAGAGAA 23611

LserUS96UC23_CD 23562 AAAGTCAGTAAATGCCTACATTAAATCAATTGATTCGTCATGGTAGAGAA 23611

LvirLAC006941_C 23577 AAAGTCAGTAAATGCCTACATTAAATCAATTGATTCGTCATGGTAGAGAA 23626

LserLAC005780_C 23595 AAAGTCAGTAAATGCCTACATTAAATCAATTGATTCGTCATGGTAGAGAA 23644

LsalLAC008020_C 23598 AAAGTCAGTAAATGCCTACATTAAATCAATTGATTCGTCATGGTAGAGAA 23647

LsalCGN5271_CDS 23598 AAAGTCAGTAAATGCCTACATTAAATCAATTGATTCGTCATGGTAGAGAA 23647

LsatLACWENDEL_C 23612 GAAAAACGGCGCACGGACCGTACTCGAGCTTCGGATCAATGTCCCCAGAA 23661

LsatSalinas_CDS 23612 GAAAAACGGCGCACGGACCGTACTCGAGCTTCGGATCAATGTCCCCAGAA 23661

LserUS96UC23_CD 23612 GAAAAACGGCGCACGGACCGTACTCGAGCTTCGGATCAATGTCCCCAGAA 23661

LvirLAC006941_C 23627 GAAAAACGGCGCACGGACCGTACTCGAGCTTCGGATCAATGTCCCCAGAA 23676

LserLAC005780_C 23645 GAAAAACGGCGCACGGACCGTACTCGAGCTTCGGATCAATGTCCCCAGAA 23694

LsalLAC008020_C 23648 GAAAAACGGCGCACGGACCGTACTCGAGCTTCGGATCAATGTCCCCAGAA 23697

LsalCGN5271_CDS 23648 GAAAAACGGCGCACGGACCGTACTCGAGCTTCGGATCAATGTCCCCAGAA 23697

LsatLACWENDEL_C 23662 GCAAGGAGTACGCCCGCGTGTACCAACGAGAACACCGAAAAAACCGAATT 23711

LsatSalinas_CDS 23662 GCAAGGAGTACGCCCGCGTGTACCAACGAGAACACCGAAAAAACCGAATT 23711

LserUS96UC23_CD 23662 GCAAGGAGTACGCCCGCGTGTACCAACGAGAACACCGAAAAAACCGAATT 23711

LvirLAC006941_C 23677 GCAAGGAGTACGCCCGCGTGTACCAACGAGAACACCGAAAAAACCGAATT 23726

LserLAC005780_C 23695 GCAAGGAGTACGCCCGCGTGTACCAACGAGAACACCGAAAAAACCGAATT 23744

LsalLAC008020_C 23698 GCAAGGAGTACGCCCGCGTGTACCAACGAGAACACCGAAAAAACCGAATT 23747

LsalCGN5271_CDS 23698 GCAAGGAGTACGCCCGCGTGTACCAACGAGAACACCGAAAAAACCGAATT 23747

LsatLACWENDEL_C 23712 CAGCTCCACGTAAGATAGCCAAAGTACGGTTGAGCAATCGACATGATATA 23761

LsatSalinas_CDS 23712 CAGCTCCACGTAAGATAGCCAAAGTACGGTTGAGCAATCGACATGATATA 23761

LserUS96UC23_CD 23712 CAGCTCCACGTAAGATAGCCAAAGTACGGTTGAGCAATCGACATGATATA 23761

LvirLAC006941_C 23727 CAGCTCCACGTAAGATAGCCAAAGTACGGTTGAGCAATCGACATGATATA 23776

LserLAC005780_C 23745 CAGCTCCACGTAAGATAGCCAAAGTACGGTTGAGCAATCGACATGATATA 23794

LsalLAC008020_C 23748 CAGCTCCACGTAAGATAGCCAAAGTACGGTTGAGCAATCGACATGATATA 23797

LsalCGN5271_CDS 23748 CAGCTCCACGTAAGATAGCCAAAGTACGGTTGAGCAATCGACATGATATA 23797

LsatLACWENDEL_C 23762 TTTGCTCACATTCCGGGGGAAGGTCATAATTCGCAGGAACATTCTATAGT 23811

LsatSalinas_CDS 23762 TTTGCTCACATTCCGGGGGAAGGTCATAATTCGCAGGAACATTCTATAGT 23811

LserUS96UC23_CD 23762 TTTGCTCACATTCCGGGGGAAGGTCATAATTCGCAGGAACATTCTATAGT 23811

LvirLAC006941_C 23777 TTTGCTCACATTCCGGGGGAAGGTCATAATTCGCAGGAACATTCTATAGT 23826

LserLAC005780_C 23795 TTTGCTCACATTCCGGGGGAAGGTCATAATTCGCAGGAACATTCTATAGT 23844

LsalLAC008020_C 23798 TTTGCTCACATTCCGGGGGAAGGTCATAATTCGCAGGAACATTCTATAGT 23847

LsalCGN5271_CDS 23798 TTTGCTCACATTCCGGGGGAAGGTCATAATTCGCAGGAACATTCTATAGT 23847

LsatLACWENDEL_C 23812 CTTAATAAGAGGAGGTAGAGTGAAAGATTCGCCAGGTGTGAAATCCCATT 23861

LsatSalinas_CDS 23812 CTTAATAAGAGGAGGTAGAGTGAAAGATTCGCCAGGTGTGAAATCCCATT 23861

LserUS96UC23_CD 23812 CTTAATAAGAGGAGGTAGAGTGAAAGATTCGCCAGGTGTGAAATCCCATT 23861

LvirLAC006941_C 23827 CTTAATAAGAGGAGGTAGAGTGAAAGATTCGCCAGGTGTGAAATCCCATT 23876

LserLAC005780_C 23845 CTTAATAAGAGGAGGTAGAGTGAAAGATTCGCCAGGTGTGAAATCCCATT 23894

LsalLAC008020_C 23848 CTTAATAAGAGGAGGTAGAGTGAAAGATTCGCCAGGTGTGAAATCCCATT 23897

LsalCGN5271_CDS 23848 CTTAATAAGAGGAGGTAGAGTGAAAGATTCGCCAGGTGTGAAATCCCATT 23897

LsatLACWENDEL_C 23862 GTATTCGAGGAGTCAAGGATTTGTTGGGAATTCCGGATCGAAGAAAAGGG 23911

LsatSalinas_CDS 23862 GTATTCGAGGAGTCAAGGATTTGTTGGGAATTCCGGATCGAAGAAAAGGG 23911

LserUS96UC23_CD 23862 GTATTCGAGGAGTCAAGGATTTGTTGGGAATTCCGGATCGAAGAAAAGGG 23911

LvirLAC006941_C 23877 GTATTCGAGGAGTCAAGGATTTGTTGGGAATTCCGGATCGAAGAAAAGGG 23926

LserLAC005780_C 23895 GTATTCGAGGAGTCAAGGATTTGTTGGGAATTCCGGATCGAAGAAAAGGG 23944

LsalLAC008020_C 23898 GTATTCGAGGAGTCAAGGATTTGTTGGGAATTCCGGATCGAAGAAAAGGG 23947

LsalCGN5271_CDS 23898 GTATTCGAGGAGTCAAGGATTTGTTGGGAATTCCGGATCGAAGAAAAGGG 23947

LsatLACWENDEL_C 23912 AGATCAAAATATGGTGCAGAAAAACCAAAATAGATGTTATATATTTCAGG 23961

LsatSalinas_CDS 23912 AGATCAAAATATGGTGCAGAAAAACCAAAATAGATGTTATATATTTCAGG 23961

LserUS96UC23_CD 23912 AGATCAAAATATGGTGCAGAAAAACCAAAATAGATGTTATATATTTCAGG 23961

LvirLAC006941_C 23927 AGATCAAAATATGGTGCAGAAAAACCAAAATAGATGTTATATATTTCAGG 23976

LserLAC005780_C 23945 AGATCAAAATATGGTGCAGAAAAACCAAAATAGATGTTATATATTTCAGG 23994

LsalLAC008020_C 23948 AGATCAAAATATGGTGCAGAAAAACCAAAATAGATGTTATATATTTCAGG 23997

LsalCGN5271_CDS 23948 AGATCAAAATATGGTGCAGAAAAACCAAAATAGATGTTATATATTTCAGG 23997

LsatLACWENDEL_C 23962 AGCTAGATCAGTTGCCGATGAACAAGTCAGAATTGCCTCAACAAAAATAG 24011

LsatSalinas_CDS 23962 AGCTAGATCAGTTGCCGATGAACAAGTCAGAATTGCCTCAACAAAAATAG 24011

LserUS96UC23_CD 23962 AGCTAGATCAGTTGCCGATGAACAAGTCAGAATTGCCTCAACAAAAATAG 24011

LvirLAC006941_C 23977 AGCTAGATCAGTTGCCGATGAACAAGTCAGAATTGCCTCAACAAAAATAG 24026

LserLAC005780_C 23995 AGCTAGATCAGTTGCCGATGAACAAGTCAGAATTGCCTCAACAAAAATAG 24044

LsalLAC008020_C 23998 AGCTAGATCAGTTGCCGATGAACAAGTCAGAATTGCCTCAACAAAAATAG 24047

LsalCGN5271_CDS 23998 AGCTAGATCAGTTGCCGATGAACAAGTCAGAATTGCCTCAACAAAAATAG 24047

LsatLACWENDEL_C 24012 ATGGAATTGGGCCTAAAAAAGCCATTCTGGTTCGTTATCGATTAGGTATC 24061

LsatSalinas_CDS 24012 ATGGAATTGGGCCTAAAAAAGCCATTCTGGTTCGTTATCGATTAGGTATC 24061

LserUS96UC23_CD 24012 ATGGAATTGGGCCTAAAAAAGCCATTCTGGTTCGTTATCGATTAGGTATC 24061

LvirLAC006941_C 24027 ATGGAATTGGGCCTAAAAAAGCCATTCTGGTTCGTTATCGATTAGGTATC 24076

LserLAC005780_C 24045 ATGGAATTGGGCCTAAAAAAGCCATTCTGGTTCGTTATCGATTAGGTATC 24094

LsalLAC008020_C 24048 ATGGAATTGGGCCTAAAAAAGCCATTCTGGTTCGTTATCGATTAGGTATC 24097

LsalCGN5271_CDS 24048 ATGGAATTGGGCCTAAAAAAGCCATTCTGGTTCGTTATCGATTAGGTATC 24097

LsatLACWENDEL_C 24062 AGTGGGAACATAAAGATAAAAGAATTAACTAAGTATCAGATCGACCAAAT 24111

LsatSalinas_CDS 24062 AGTGGGAACATAAAGATAAAAGAATTAACTAAGTATCAGATCGACCAAAT 24111

LserUS96UC23_CD 24062 AGTGGGAACATAAAGATAAAAGAATTAACTAAGTATCAGATCGACCAAAT 24111

LvirLAC006941_C 24077 AGTGGGAACATAAAGATAAAAGAATTAACTAAGTATCAGATCGACCAAAT 24126

LserLAC005780_C 24095 AGTGGGAACATAAAGATAAAAGAATTAACTAAGTATCAGATCGACCAAAT 24144

LsalLAC008020_C 24098 AGTGGGAACATAAAGATAAAAGAATTAACTAAGTATCAGATCGACCAAAT 24147

LsalCGN5271_CDS 24098 AGTGGGAACATAAAGATAAAAGAATTAACTAAGTATCAGATCGACCAAAT 24147

LsatLACWENDEL_C 24112 TGAACAAATGATAGGTCAAGATCATGTTGTTCATTGGGAATTGAAGAGGG 24161

LsatSalinas_CDS 24112 TGAACAAATGATAGGTCAAGATCATGTTGTTCATTGGGAATTGAAGAGGG 24161

LserUS96UC23_CD 24112 TGAACAAATGATAGGTCAAGATCATGTTGTTCATTGGGAATTGAAGAGGG 24161

LvirLAC006941_C 24127 TGAACAAATGATAGGTCAAGATCATGTTGTTCATTGGGAATTGAAGAGGG 24176

LserLAC005780_C 24145 TGAACAAATGATAGGTCAAGATCATGTTGTTCATTGGGAATTGAAGAGGG 24194

LsalLAC008020_C 24148 TGAACAAATGATAGGTCAAGATCATGTTGTTCATTGGGAATTGAAGAGGG 24197

LsalCGN5271_CDS 24148 TGAACAAATGATAGGTCAAGATCATGTTGTTCATTGGGAATTGAAGAGGG 24197

LsatLACWENDEL_C 24162 GAGAACGAGCAGACATCGAACGATTAATTTCTATTTCTTGTTATCGTGGA 24211

LsatSalinas_CDS 24162 GAGAACGAGCAGACATCGAACGATTAATTTCTATTTCTTGTTATCGTGGA 24211

LserUS96UC23_CD 24162 GAGAACGAGCAGACATCGAACGATTAATTTCTATTTCTTGTTATCGTGGA 24211

LvirLAC006941_C 24177 GAGAACGAGCAGACATCGAACGATTAATTTCTATTTCTTGTTATCGTGGA 24226

LserLAC005780_C 24195 GAGAACGAGCAGACATCGAACGATTAATTTCTATTTCTTGTTATCGTGGA 24244

LsalLAC008020_C 24198 GAGAACGAGCAGACATCGAACGATTAATTTCTATTTCTTGTTATCGTGGA 24247

LsalCGN5271_CDS 24198 GAGAACGAGCAGACATCGAACGATTAATTTCTATTTCTTGTTATCGTGGA 24247

LsatLACWENDEL_C 24212 ATTCGTCATCAAGATGGATCACCCTTACGCGGTCAACGAACTCATACTAA 24261

LsatSalinas_CDS 24212 ATTCGTCATCAAGATGGATCACCCTTACGCGGTCAACGAACTCATACTAA 24261

LserUS96UC23_CD 24212 ATTCGTCATCAAGATGGATCACCCTTACGCGGTCAACGAACTCATACTAA 24261

LvirLAC006941_C 24227 ATTCGTCATCAAGATGGATCACCCTTACGCGGTCAACGAACTCATACTAA 24276

LserLAC005780_C 24245 ATTCGTCATCAAGATGGATCACCCTTACGCGGTCAACGAACTCATACTAA 24294

LsalLAC008020_C 24248 ATTCGTCATCAAGATGGATCACCCTTACGCGGTCAACGAACTCATACTAA 24297

LsalCGN5271_CDS 24248 ATTCGTCATCAAGATGGATCACCCTTACGCGGTCAACGAACTCATACTAA 24297

LsatLACWENDEL_C 24262 TGCTAGGACTTGTCGCAAGCAAATTCGGAAATGAATGGCACGAAAAGGAA 24311

LsatSalinas_CDS 24262 TGCTAGGACTTGTCGCAAGCAAATTCGGAAATGAATGGCACGAAAAGGAA 24311

LserUS96UC23_CD 24262 TGCTAGGACTTGTCGCAAGCAAATTCGGAAATGAATGGCACGAAAAGGAA 24311

LvirLAC006941_C 24277 TGCTAGGACTTGTCGCAAGCAAATTCGGAAATGAATGGCACGAAAAGGAA 24326

LserLAC005780_C 24295 TGCTAGGACTTGTCGCAAGCAAATTCGGAAATGAATGGCACGAAAAGGAA 24344

LsalLAC008020_C 24298 TGCTAGGACTTGTCGCAAGCAAATTCGGAAATGAATGGCACGAAAAGGAA 24347

LsalCGN5271_CDS 24298 TGCTAGGACTTGTCGCAAGCAAATTCGGAAATGAATGGCACGAAAAGGAA 24347

LsatLACWENDEL_C 24312 ATCCAATTTCGGTAAGACTTGGTCTGAATCGTAGTTCAGATTCAAGTCGG 24361

LsatSalinas_CDS 24312 ATCCAATTTCGGTAAGACTTGGTCTGAATCGTAGTTCAGATTCAAGTCGG 24361

LserUS96UC23_CD 24312 ATCCAATTTCGGTAAGACTTGGTCTGAATCGTAGTTCAGATTCAAGTCGG 24361

LvirLAC006941_C 24327 ATCCAATTTCGGTAAGACTTGGTCTGAATCGTAGTTCAGATTCAAGTCGG 24376

LserLAC005780_C 24345 ATCCAATTTCGGTAAGACTTGGTCTGAATCGTAGTTCAGATTCAAGTCGG 24394

LsalLAC008020_C 24348 ATCCAATTTCGGTAAGACTTGGTCTGAATCGTAGTTCAGATTCAAGTCGG 24397

LsalCGN5271_CDS 24348 ATCCAATTTCGGTAAGACTTGGTCTGAATCGTAGTTCAGATTCAAGTCGG 24397

LsatLACWENDEL_C 24362 TTCAGTGATTATTATTATGGTAAATCAGTGTATCAAGATGTCAATCTTAG 24411

LsatSalinas_CDS 24362 TTCAGTGATTATTATTATGGTAAATCAGTGTATCAAGATGTCAATCTTAG 24411

LserUS96UC23_CD 24362 TTCAGTGATTATTATTATGGTAAATCAGTGTATCAAGATGTCAATCTTAG 24411

LvirLAC006941_C 24377 TTCAGTGATTATTATTATGGTAAATCAGTGTATCAAGATGTCAATCTTAG 24426

LserLAC005780_C 24395 TTCAGTGATTATTATTATGGTAAATCAGTGTATCAAGATGTCAATCTTAG 24444

LsalLAC008020_C 24398 TTCAGTGATTATTATTATGGTAAATCAGTGTATCAAGATGTCAATCTTAG 24447

LsalCGN5271_CDS 24398 TTCAGTGATTATTATTATGGTAAATCAGTGTATCAAGATGTCAATCTTAG 24447

LsatLACWENDEL_C 24412 ATCTTATTTCGGTTCGATACGTCCACCTACTAGACTCACCTTTGGCTTTC 24461

LsatSalinas_CDS 24412 ATCTTATTTCGGTTCGATACGTCCACCTACTAGACTCACCTTTGGCTTTC 24461

LserUS96UC23_CD 24412 ATCTTATTTCGGTTCGATACGTCCACCTACTAGACTCACCTTTGGCTTTC 24461

LvirLAC006941_C 24427 ATCTTATTTCGGTTCGATACGTCCACCTACTAGACTCACCTTTGGCTTTC 24476

LserLAC005780_C 24445 ATCTTATTTCGGTTCGATACGTCCACCTACTAGACTCACCTTTGGCTTTC 24494

LsalLAC008020_C 24448 ATCTTATTTCGGTTCGATACGTCCACCTACTAGACTCACCTTTGGCTTTC 24497

LsalCGN5271_CDS 24448 ATCTTATTTCGGTTCGATACGTCCACCTACTAGACTCACCTTTGGCTTTC 24497

LsatLACWENDEL_C 24462 GTCTCGGTAGGTGTATTATTATACATTTTCCCAAAAGAACATTCATTCAT 24511

LsatSalinas_CDS 24462 GTCTCGGTAGGTGTATTATTATACATTTTCCCAAAAGAACATTCATTCAT 24511

LserUS96UC23_CD 24462 GTCTCGGTAGGTGTATTATTATACATTTTCCCAAAAGAACATTCATTCAT 24511

LvirLAC006941_C 24477 GTCTCGGTAGGTGTATTATTATACATTTTCCCAAAAGAACATTCATTCAT 24526

LserLAC005780_C 24495 GTCTCGGTAGGTGTATTATTATACATTTTCCCAAAAGAACATTCATTCAT 24544

LsalLAC008020_C 24498 GTCTCGGTAGGTGTATTATTATACATTTTCCCAAAAGAACATTCATTCAT 24547

LsalCGN5271_CDS 24498 GTCTCGGTAGGTGTATTATTATACATTTTCCCAAAAGAACATTCATTCAT 24547

LsatLACWENDEL_C 24512 TTCTTTCTTCCCCGTCGACCACGACGACAGAAACGACGCGAAAAATCCAG 24561

LsatSalinas_CDS 24512 TTCTTTCTTCCCCGTCGACCACGACGACAGAAACGACGCGAAAAATCCAG 24561

LserUS96UC23_CD 24512 TTCTTTCTTCCCCGTCGACCACGACGACAGAAACGACGCGAAAAATCCAG 24561

LvirLAC006941_C 24527 TTCTTTCTTCCCCGTCGACCACGACGACAGAAACGACGCGAAAAATCCAG 24576

LserLAC005780_C 24545 TTCTTTCTTCCCCGTCGACCACGACGACAGAAACGACGCGAAAAATCCAG 24594

LsalLAC008020_C 24548 TTCTTTCTTCCCCGTCGACCACGACGACAGAAACGACGCGAAAAATCCAG 24597

LsalCGN5271_CDS 24548 TTCTTTCTTCCCCGTCGACCACGACGACAGAAACGACGCGAAAAATCCAG 24597

LsatLACWENDEL_C 24562 ACCCGGAAAGGGGAAGGGCCAGTGGTGGGCATTTGGGAAAGTCGGGCCGA 24611

LsatSalinas_CDS 24562 ACCCGGAAAGGGGAAGGGCCAGTGGTGGGCATTTGGGAAAGTCGGGCCGA 24611

LserUS96UC23_CD 24562 ACCCGGAAAGGGGAAGGGCCAGTGGTGGGCATTTGGGAAAGTCGGGCCGA 24611

LvirLAC006941_C 24577 ACCCGGAAAGGGGAAGGGCCAGTGGTGGGCATTTGGGAAAGTCGGGCCGA 24626

LserLAC005780_C 24595 ACCCGGAAAGGGGAAGGGCCAGTGGTGGGCATTTGGGAAAGTCGGGCCGA 24644

LsalLAC008020_C 24598 ACCCGGAAAGGGGAAGGGCCAGTGGTGGGCATTTGGGAAAGTCGGGCCGA 24647

LsalCGN5271_CDS 24598 ACCCGGAAAGGGGAAGGGCCAGTGGTGGGCATTTGGGAAAGTCGGGCCGA 24647

LsatLACWENDEL_C 24612 TCGGGTGTCTTCATTCAAGCGACGGTACAGAAGAAGAACGAAACGAAGTG 24661

LsatSalinas_CDS 24612 TCGGGTGTCTTCATTCAAGCGACGGTACAGAAGAAGAACGAAACGAAGTG 24661

LserUS96UC23_CD 24612 TCGGGTGTCTTCATTCAAGCGACGGTACAGAAGAAGAACGAAACGAAGTG 24661

LvirLAC006941_C 24627 TCGGGTGTCTTCATTCAAGCGACGGTACAGAAGAAGAACGAAACGAAGTG 24676

LserLAC005780_C 24645 TCGGGTGTCTTCATTCAAGCGACGGTACAGAAGAAGAACGAAACGAAGTG 24694

LsalLAC008020_C 24648 TCGGGTGTCTTCATTCAAGCGACGGTACAGAAGAAGAACGAAACGAAGTG 24697

LsalCGN5271_CDS 24648 TCGGGTGTCTTCATTCAAGCGACGGTACAGAAGAAGAACGAAACGAAGTG 24697

LsatLACWENDEL_C 24662 AGAGGCCGGGGGGCAGGGAAAAGAGTCGAGTCGATCAGGCTCGACGACCG 24711

LsatSalinas_CDS 24662 AGAGGCCGGGGGGCAGGGAAAAGAGTCGAGTCGATCAGGCTCGACGACCG 24711

LserUS96UC23_CD 24662 AGAGGCCGGGGGGCAGGGAAAAGAGTCGAGTCGATCAGGCTCGACGACCG 24711

LvirLAC006941_C 24677 AGAGGCCGGGGGGCAGGGAAAAGAGTCGAGTCGATCAGGCTCGACGACCG 24726

LserLAC005780_C 24695 AGAGGCCGGGGGGCAGGGAAAAGAGTCGAGTCGATCAGGCTCGACGACCG 24744

LsalLAC008020_C 24698 AGAGGCCGGGGGGCAGGGAAAAGAGTCGAGTCGATCAGGCTCGACGACCG 24747

LsalCGN5271_CDS 24698 AGAGGCCGGGGGGCAGGGAAAAGAGTCGAGTCGATCAGGCTCGACGACCG 24747

LsatLACWENDEL_C 24712 GGAGAAGCAAAACGAAATCCGGATTTGGCCGAAAAAGAAGCAACGCTATG 24761

LsatSalinas_CDS 24712 GGAGAAGCAAAACGAAATCCGGATTTGGCCGAAAAAGAAGCAACGCTATG 24761

LserUS96UC23_CD 24712 GGAGAAGCAAAACGAAATCCGGATTTGGCCGAAAAAGAAGCAACGCTATG 24761

LvirLAC006941_C 24727 GGAGAAGCAAAACGAAATCCGGATTTGGCCGAAAAAGAAGCAACGCTATG 24776

LserLAC005780_C 24745 GGAGAAGCAAAACGAAATCCGGATTTGGCCGAAAAAGAAGCAACGCTATG 24794

LsalLAC008020_C 24748 GGAGAAGCAAAACGAAATCCGGATTTGGCCGAAAAAGAAGCAACGCTATG 24797

LsalCGN5271_CDS 24748 GGAGAAGCAAAACGAAATCCGGATTTGGCCGAAAAAGAAGCAACGCTATG 24797

LsatLACWENDEL_C 24762 GATACCATGACCGATCACCATCGATAAAGAAGAATCTTTCTAAATCACTT 24811

LsatSalinas_CDS 24762 GATACCATGACCGATCACCATCGATAAAGAAGAATCTTTCTAAATCACTT 24811

LserUS96UC23_CD 24762 GATACCATGACCGATCACCATCGATAAAGAAGAATCTTTCTAAATCACTT 24811

LvirLAC006941_C 24777 GATACCATGACCGATCACCATCGATAAAGAAGAATCTTTCTAAATCACTT 24826

LserLAC005780_C 24795 GATACCATGACCGATCACCATCGATAAAGAAGAATCTTTCTAAATCACTT 24844

LsalLAC008020_C 24798 GATACCATGACCGATCACCATCGATAAAGAAGAATCTTTCTAAATCACTT 24847

LsalCGN5271_CDS 24798 GATACCATGACCGATCACCATCGATAAAGAAGAATCTTTCTAAATCACTT 24847

LsatLACWENDEL_C 24812 CGGGTCAGCGGGGCCTTCAAGCATCCGAAATACGCCGGGCTTGTAAATGA 24861

LsatSalinas_CDS 24812 CGGGTCAGCGGGGCCTTCAAGCATCCGAAATACGCCGGGCTTGTAAATGA 24861

LserUS96UC23_CD 24812 CGGGTCAGCGGGGCCTTCAAGCATCCGAAATACGCCGGGCTTGTAAATGA 24861

LvirLAC006941_C 24827 CGGGTCAGCGGGGCCTTCAAGCATCCGAAATACGCCGGGCTTGTAAATGA 24876

LserLAC005780_C 24845 CGGGTCAGCGGGGCCTTCAAGCATCCGAAATACGCCGGGCTTGTAAATGA 24894

LsalLAC008020_C 24848 CGGGTCAGCGGGGCCTTCAAGCATCCGAAATACGCCGGGCTTGTAAATGA 24897

LsalCGN5271_CDS 24848 CGGGTCAGCGGGGCCTTCAAGCATCCGAAATACGCCGGGCTTGTAAATGA 24897

LsatLACWENDEL_C 24862 CATAGCCCTCCTAAATGACGACTCCTTCAGAAAAACGAAGTTATTCAAGT 24911

LsatSalinas_CDS 24862 CATAGCCCTCCTAAATGACGACTCCTTCAGAAAAACGAAGTTATTCAAGT 24911

LserUS96UC23_CD 24862 CATAGCCCTCCTAAATGACGACTCCTTCAGAAAAACGAAGTTATTCAAGT 24911

LvirLAC006941_C 24877 CATAGCCCTCCTAAATGACGACTCCTTCAGAAAAACGAAGTTATTCAAGT 24926

LserLAC005780_C 24895 CATAGCCCTCCTAAATGACGACTCCTTCAGAAAAACGAAGTTATTCAAGT 24944

LsalLAC008020_C 24898 CATAGCCCTCCTAAATGACGACTCCTTCAGAAAAACGAAGTTATTCAAGT 24947

LsalCGN5271_CDS 24898 CATAGCCCTCCTAAATGACGACTCCTTCAGAAAAACGAAGTTATTCAAGT 24947

LsatLACWENDEL_C 24912 TCTTTTTCTCAAAGAAGTCCCCCTCCGACAGCCCGACGAGTCATCCACTT 24961

LsatSalinas_CDS 24912 TCTTTTTCTCAAAGAAGTCCCCCTCCGACAGCCCGACGAGTCATCCACTT 24961

LserUS96UC23_CD 24912 TCTTTTTCTCAAAGAAGTCCCCCTCCGACAGCCCGACGAGTCATCCACTT 24961

LvirLAC006941_C 24927 TCTTTTTCTCAAAGAAGTCCCCCTCCGACAGCCCGACGAGTCATCCACTT 24976

LserLAC005780_C 24945 TCTTTTTCTCAAAGAAGTCCCCCTCCGACAGCCCGACGAGTCATCCACTT 24994

LsalLAC008020_C 24948 TCTTTTTCTCAAAGAAGTCCCCCTCCGACAGCCCGACGAGTCATCCACTT 24997

LsalCGN5271_CDS 24948 TCTTTTTCTCAAAGAAGTCCCCCTCCGACAGCCCGACGAGTCATCCACTT 24997

LsatLACWENDEL_C 24962 AAAAGGACCCTCCCTGCGGTGCGCCCTTCCTTGAATTATTCGGTCATGCA 25011

LsatSalinas_CDS 24962 AAAAGGACCCTCCCTGCGGTGCGCCCTTCCTTGAATTATTCGGTCATGCA 25011

LserUS96UC23_CD 24962 AAAAGGACCCTCCCTGCGGTGCGCCCTTCCTTGAATTATTCGGTCATGCA 25011

LvirLAC006941_C 24977 AAAAGGACCCTCCCTGCGGTGCGCCCTTCCTTGAATTATTCGGTCATGCA 25026

LserLAC005780_C 24995 AAAAGGACCCTCCCTGCGGTGCGCCCTTCCTTGAATTATTCGGTCATGCA 25044

LsalLAC008020_C 24998 AAAAGGACCCTCCCTGCGGTGCGCCCTTCCTTGAATTATTCGGTCATGCA 25047

LsalCGN5271_CDS 24998 AAAAGGACCCTCCCTGCGGTGCGCCCTTCCTTGAATTATTCGGTCATGCA 25047

LsatLACWENDEL_C 25012 ATACTTATTGAAGACAAAGAACCAAATGCATTTCGACCCCGTCGTAGTTC 25061

LsatSalinas_CDS 25012 ATACTTATTGAAGACAAAGAACCAAATGCATTTCGACCCCGTCGTAGTTC 25061

LserUS96UC23_CD 25012 ATACTTATTGAAGACAAAGAACCAAATGCATTTCGACCCCGTCGTAGTTC 25061

LvirLAC006941_C 25027 ATACTTATTGAAGACAAAGAACCAAATGCATTTCGACCCCGTCGTAGTTC 25076

LserLAC005780_C 25045 ATACTTATTGAAGACAAAGAACCAAATGCATTTCGACCCCGTCGTAGTTC 25094

LsalLAC008020_C 25048 ATACTTATTGAAGACAAAGAACCAAATGCATTTCGACCCCGTCGTAGTTC 25097

LsalCGN5271_CDS 25048 ATACTTATTGAAGACAAAGAACCAAATGCATTTCGACCCCGTCGTAGTTC 25097

LsatLACWENDEL_C 25062 TCAATCATTTCGTGGAACCGGGCGTGGTTGAACCATCTACCATGGGGGGA 25111

LsatSalinas_CDS 25062 TCAATCATTTCGTGGAACCGGGCGTGGTTGAACCATCTACCATGGGGGGA 25111

LserUS96UC23_CD 25062 TCAATCATTTCGTGGAACCGGGCGTGGTTGAACCATCTACCATGGGGGGA 25111

LvirLAC006941_C 25077 TCAATCATTTCGTGGAACCGGGCGTGGTTGAACCATCTACCATGGGGGGA 25126

LserLAC005780_C 25095 TCAATCATTTCGTGGAACCGGGCGTGGTTGAACCATCTACCATGGGGGGA 25144

LsalLAC008020_C 25098 TCAATCATTTCGTGGAACCGGGCGTGGTTGAACCATCTACCATGGGGGGA 25147

LsalCGN5271_CDS 25098 TCAATCATTTCGTGGAACCGGGCGTGGTTGAACCATCTACCATGGGGGGA 25147

LsatLACWENDEL_C 25112 GCTAATGCACAGGGAAGAAGCTTAGATAAGAGAATACGTTTCGCTTTTTT 25161

LsatSalinas_CDS 25112 GCTAATGCACAGGGAAGAAGCTTAGATAAGAGAATACGTTTCGCTTTTTT 25161

LserUS96UC23_CD 25112 GCTAATGCACAGGGAAGAAGCTTAGATAAGAGAATACGTTTCGCTTTTTT 25161

LvirLAC006941_C 25127 GCTAATGCACAGGGAAGAAGCTTAGATAAGAGAATACGTTTCGCTTTTTT 25176

LserLAC005780_C 25145 GCTAATGCACAGGGAAGAAGCTTAGATAAGAGAATACGTTTCGCTTTTTT 25194

LsalLAC008020_C 25148 GCTAATGCACAGGGAAGAAGCTTAGATAAGAGAATACGTTTCGCTTTTTT 25197

LsalCGN5271_CDS 25148 GCTAATGCACAGGGAAGAAGCTTAGATAAGAGAATACGTTTCGCTTTTTT 25197

LsatLACWENDEL_C 25162 TGTCGAAAGCTCGACCAGCGAGAAAAAGTTTTTGGCCGAAGACAAAAAGT 25211

LsatSalinas_CDS 25162 TGTCGAAAGCTCGACCAGCGAGAAAAAGTTTTTGGCCGAAGACAAAAAGT 25211

LserUS96UC23_CD 25162 TGTCGAAAGCTCGACCAGCGAGAAAAAGTTTTTGGCCGAAGACAAAAAGT 25211

LvirLAC006941_C 25177 TGTCGAAAGCTCGACCAGCGAGAAAAAGTTTTTGGCCGAAGACAAAAAGT 25226

LserLAC005780_C 25195 TGTCGAAAGCTCGACCAGCGAGAAAAAGTTTTTGGCCGAAGACAAAAAGT 25244

LsalLAC008020_C 25198 TGTCGAAAGCTCGACCAGCGAGAAAAAGTTTTTGGCCGAAGACAAAAAGT 25247

LsalCGN5271_CDS 25198 TGTCGAAAGCTCGACCAGCGAGAAAAAGTTTTTGGCCGAAGACAAAAAGT 25247

LsatLACWENDEL_C 25212 TGACCCACTTCATTCGCTGGTCGGATCATCCTCGCTTCGCGGGAACAACA 25261

LsatSalinas_CDS 25212 TGACCCACTTCATTCGCTGGTCGGATCATCCTCGCTTCGCGGGAACAACA 25261

LserUS96UC23_CD 25212 TGACCCACTTCATTCGCTGGTCGGATCATCCTCGCTTCGCGGGAACAACA 25261

LvirLAC006941_C 25227 TGACCCACTTCATTCGCTGGTCGGATCATCCTCGCTTCGCGGGAACAACA 25276

LserLAC005780_C 25245 TGACCCACTTCATTCGCTGGTCGGATCATCCTCGCTTCGCGGGAACAACA 25294

LsalLAC008020_C 25248 TGACCCACTTCATTCGCTGGTCGGATCATCCTCGCTTCGCGGGAACAACA 25297

LsalCGN5271_CDS 25248 TGACCCACTTCATTCGCTGGTCGGATCATCCTCGCTTCGCGGGAACAACA 25297

LsatLACWENDEL_C 25262 AAAACCACCATCTCGCTCTTTCCTTTCTTCGGTGCTACCTTTTTCTTTCC 25311

LsatSalinas_CDS 25262 AAAACCACCATCTCGCTCTTTCCTTTCTTCGGTGCTACCTTTTTCTTTCC 25311

LserUS96UC23_CD 25262 AAAACCACCATCTCGCTCTTTCCTTTCTTCGGTGCTACCTTTTTCTTTCC 25311

LvirLAC006941_C 25277 AAAACCACCATCTCGCTCTTTCCTTTCTTCGGTGCTACCTTTTTCTTTCC 25326

LserLAC005780_C 25295 AAAACCACCATCTCGCTCTTTCCTTTCTTCGGTGCTACCTTTTTCTTTCC 25344

LsalLAC008020_C 25298 AAAACCACCATCTCGCTCTTTCCTTTCTTCGGTGCTACCTTTTTCTTTCC 25347

LsalCGN5271_CDS 25298 AAAACCACCATCTCGCTCTTTCCTTTCTTCGGTGCTACCTTTTTCTTTCC 25347

LsatLACWENDEL_C 25312 AAGGGACGGGGTTGGGGTGTATAAGAACCTTTTTTTTGAGTATGCCCGGG 25361

LsatSalinas_CDS 25312 AAGGGACGGGGTTGGGGTGTATAAGAACCTTTTTTTTGAGTATGCCCGGG 25361

LserUS96UC23_CD 25312 AAGGGACGGGGTTGGGGTGTATAAGAACCTTTTTTTTGAGTATGCCCGGG 25361

LvirLAC006941_C 25327 AAGGGACGGGGTTGGGGTGTATAAGAACCTTTTTTTTGAGTATGCCCGGG 25376

LserLAC005780_C 25345 AAGGGACGGGGTTGGGGTGTATAAGAACCTTTTTTTTGAGTATGCCCGGG 25394

LsalLAC008020_C 25348 AAGGGACGGGGTTGGGGTGTATAAGAACCTTTTTTTTGAGTATGCCCGGG 25397

LsalCGN5271_CDS 25348 AAGGGACGGGGTTGGGGTGTATAAGAACCTTTTTTTTGAGTATGCCCGGG 25397

LsatLACWENDEL_C 25362 AACAACTCCTACGAAAATTCAGGAAAAAATGTTGGAACCTCACGGCTAAG 25411

LsatSalinas_CDS 25362 AACAACTCCTACGAAAATTCAGGAAAAAATGTTGGAACCTCACGGCTAAG 25411

LserUS96UC23_CD 25362 AACAACTCCTACGAAAATTCAGGAAAAAATGTTGGAACCTCACGGCTAAG 25411

LvirLAC006941_C 25377 AACAACTCCTACGAAAATTCAGGAAAAAATGTTGGAACCTCACGGCTAAG 25426

LserLAC005780_C 25395 AACAACTCCTACGAAAATTCAGGAAAAAATGTTGGAACCTCACGGCTAAG 25444

LsalLAC008020_C 25398 AACAACTCCTACGAAAATTCAGGAAAAAATGTTGGAACCTCACGGCTAAG 25447

LsalCGN5271_CDS 25398 AACAACTCCTACGAAAATTCAGGAAAAAATGTTGGAACCTCACGGCTAAG 25447

LsatLACWENDEL_C 25412 GATAAGGTAATGGAATTGATAGAGAAATTCATAGACCTAGGTGGGATAGG 25461

LsatSalinas_CDS 25412 GATAAGGTAATGGAATTGATAGAGAAATTCATAGACCTAGGTGGGATAGG 25461

LserUS96UC23_CD 25412 GATAAGGTAATGGAATTGATAGAGAAATTCATAGACCTAGGTGGGATAGG 25461

LvirLAC006941_C 25427 GATAAGGTAATGGAATTGATAGAGAAATTCATAGACCTAGGTGGGATAGG 25476

LserLAC005780_C 25445 GATAAGGTAATGGAATTGATAGAGAAATTCATAGACCTAGGTGGGATAGG 25494

LsalLAC008020_C 25448 GATAAGGTAATGGAATTGATAGAGAAATTCATAGACCTAGGTGGGATAGG 25497

LsalCGN5271_CDS 25448 GATAAGGTAATGGAATTGATAGAGAAATTCATAGACCTAGGTGGGATAGG 25497

LsatLACWENDEL_C 25462 AGAATTGATAAAGGGAATAGAGATGATGATAGAGATCATACTGAGAAACA 25511

LsatSalinas_CDS 25462 AGAATTGATAAAGGGAATAGAGATGATGATAGAGATCATACTGAGAAACA 25511

LserUS96UC23_CD 25462 AGAATTGATAAAGGGAATAGAGATGATGATAGAGATCATACTGAGAAACA 25511

LvirLAC006941_C 25477 AGAATTGATAAAGGGAATAGAGATGATGATAGAGATCATACTGAGAAACA 25526

LserLAC005780_C 25495 AGAATTGATAAAGGGAATAGAGATGATGATAGAGATCATACTGAGAAACA 25544

LsalLAC008020_C 25498 AGAATTGATAAAGGGAATAGAGATGATGATAGAGATCATACTGAGAAACA 25547

LsalCGN5271_CDS 25498 AGAATTGATAAAGGGAATAGAGATGATGATAGAGATCATACTGAGAAACA 25547

LsatLACWENDEL_C 25512 GAAGAATTCCGTACGGGTACAACTTTTATTTGAACGAAGTGAAAAAAATG 25561

LsatSalinas_CDS 25512 GAAGAATTCCGTACGGGTACAACTTTTATTTGAACGAAGTGAAAAAAATG 25561

LserUS96UC23_CD 25512 GAAGAATTCCGTACGGGTACAACTTTTATTTGAACGAAGTGAAAAAAATG 25561

LvirLAC006941_C 25527 GAAGAATTCCGTACGGGTACAACTTTTATTTGAACGAAGTGAAAAAAATG 25576

LserLAC005780_C 25545 GAAGAATTCCGTACGGGTACAACTTTTATTTGAACGAAGTGAAAAAAATG 25594

LsalLAC008020_C 25548 GAAGAATTCCGTACGGGTACAACTTTTATTTGAACGAAGTGAAAAAAATG 25597

LsalCGN5271_CDS 25548 GAAGAATTCCGTACGGGTACAACTTTTATTTGAACGAAGTGAAAAAAATG 25597

LsatLACWENDEL_C 25562 CGATCTTTGTTGTCTAATAGAACAAAGACTAATACCTTAATTGAGTCGGT 25611

LsatSalinas_CDS 25562 CGATCTTTGTTGTCTAATAGAACAAAGACTAATACCTTAATTGAGTCGGT 25611

LserUS96UC23_CD 25562 CGATCTTTGTTGTCTAATAGAACAAAGACTAATACCTTAATTGAGTCGGT 25611

LvirLAC006941_C 25577 CGATCTTTGTTGTCTAATAGAACAAAGACTAATACCTTAATTGAGTCGGT 25626

LserLAC005780_C 25595 CGATCTTTGTTGTCTAATAGAACAAAGACTAATACCTTAATTGAGTCGGT 25644

LsalLAC008020_C 25598 CGATCTTTGTTGTCTAATAGAACAAAGACTAATACCTTAATTGAGTCGGT 25647

LsalCGN5271_CDS 25598 CGATCTTTGTTGTCTAATAGAACAAAGACTAATACCTTAATTGAGTCGGT 25647

LsatLACWENDEL_C 25612 CAAGATCAAATCTGTTTATCAAAGTGCTTCTCCGATTGCTCAAGATATCT 25661

LsatSalinas_CDS 25612 CAAGATCAAATCTGTTTATCAAAGTGCTTCTCCGATTGCTCAAGATATCT 25661

LserUS96UC23_CD 25612 CAAGATCAAATCTGTTTATCAAAGTGCTTCTCCGATTGCTCAAGATATCT 25661

LvirLAC006941_C 25627 CAAGATCAAATCTGTTTATCAAAGTGCTTCTCCGATTGCTCAAGATATCT 25676

LserLAC005780_C 25645 CAAGATCAAATCTGTTTATCAAAGTGCTTCTCCGATTGCTCAAGATATCT 25694

LsalLAC008020_C 25648 CAAGATCAAATCTGTTTATCAAAGTGCTTCTCCGATTGCTCAAGATATCT 25697

LsalCGN5271_CDS 25648 CAAGATCAAATCTGTTTATCAAAGTGCTTCTCCGATTGCTCAAGATATCT 25697

LsatLACWENDEL_C 25662 CTTTTCAACCGAGGAACAAAACAAGATCATTTCGCTCCATTTTTAGTCAA 25711

LsatSalinas_CDS 25662 CTTTTCAACCGAGGAACAAAACAAGATCATTTCGCTCCATTTTTAGTCAA 25711

LserUS96UC23_CD 25662 CTTTTCAACCGAGGAACAAAACAAGATCATTTCGCTCCATTTTTAGTCAA 25711

LvirLAC006941_C 25677 CTTTTCAACCGAGGAACAAAACAAGATCATTTCGCTCCATTTTTAGTCAA 25726

LserLAC005780_C 25695 CTTTTCAACCGAGGAACAAAACAAGATCATTTCGCTCCATTTTTAGTCAA 25744

LsalLAC008020_C 25698 CTTTTCAACCGAGGAACAAAACAAGATCATTTCGCTCCATTTTTAGTCAA 25747

LsalCGN5271_CDS 25698 CTTTTCAACCGAGGAACAAAACAAGATCATTTCGCTCCATTTTTAGTCAA 25747

LsatLACWENDEL_C 25712 ATAGTGAAGGATATTCGATTAGTAATGAAAAAAGGGGTGGAGGGGATCCG 25761

LsatSalinas_CDS 25712 ATAGTGAAGGATATTCGATTAGTAATGAAAAAAGGGGTGGAGGGGATCCG 25761

LserUS96UC23_CD 25712 ATAGTGAAGGATATTCGATTAGTAATGAAAAAAGGGGTGGAGGGGATCCG 25761

LvirLAC006941_C 25727 ATAGTGAAGGATATTCGATTAGTAATGAAAAAAGGGGTGGAGGGGATCCG 25776

LserLAC005780_C 25745 ATAGTGAAGGATATTCGATTAGTAATGAAAAAAGGGGTGGAGGGGATCCG 25794

LsalLAC008020_C 25748 ATAGTGAAGGATATTCGATTAGTAATGAAAAAAGGGGTGGAGGGGATCCG 25797

LsalCGN5271_CDS 25748 ATAGTGAAGGATATTCGATTAGTAATGAAAAAAGGGGTGGAGGGGATCCG 25797

LsatLACWENDEL_C 25762 TATATGTTGTTCAGGTCGATCAGAAGGTGCAGAAATAGCTAGAACTGAAT 25811

LsatSalinas_CDS 25762 TATATGTTGTTCAGGTCGATCAGAAGGTGCAGAAATAGCTAGAACTGAAT 25811

LserUS96UC23_CD 25762 TATATGTTGTTCAGGTCGATCAGAAGGTGCAGAAATAGCTAGAACTGAAT 25811

LvirLAC006941_C 25777 TATATGTTGTTCAGGTCGATCAGAAGGTGCAGAAATAGCTAGAACTGAAT 25826

LserLAC005780_C 25795 TATATGTTGTTCAGGTCGATCAGAAGGTGCAGAAATAGCTAGAACTGAAT 25844

LsalLAC008020_C 25798 TATATGTTGTTCAGGTCGATCAGAAGGTGCAGAAATAGCTAGAACTGAAT 25847

LsalCGN5271_CDS 25798 TATATGTTGTTCAGGTCGATCAGAAGGTGCAGAAATAGCTAGAACTGAAT 25847

LsatLACWENDEL_C 25812 GCGGAAAGTATGGAAAAACATCTAGTAATGTATTTAACCAGAAAATAGAT 25861

LsatSalinas_CDS 25812 GCGGAAAGTATGGAAAAACATCTAGTAATGTATTTAACCAGAAAATAGAT 25861

LserUS96UC23_CD 25812 GCGGAAAGTATGGAAAAACATCTAGTAATGTATTTAACCAGAAAATAGAT 25861

LvirLAC006941_C 25827 GCGGAAAGTATGGAAAAACATCTAGTAATGTATTTAACCAGAAAATAGAT 25876

LserLAC005780_C 25845 GCGGAAAGTATGGAAAAACATCTAGTAATGTATTTAACCAGAAAATAGAT 25894

LsalLAC008020_C 25848 GCGGAAAGTATGGAAAAACATCTAGTAATGTATTTAACCAGAAAATAGAT 25897

LsalCGN5271_CDS 25848 GCGGAAAGTATGGAAAAACATCTAGTAATGTATTTAACCAGAAAATAGAT 25897

LsatLACWENDEL_C 25862 TATGCTCCTGCGGAAGTATCTACTCGTTACGGAATCTCAGGTGTCAAAGT 25911

LsatSalinas_CDS 25862 TATGCTCCTGCGGAAGTATCTACTCGTTACGGAATCTCAGGTGTCAAAGT 25911

LserUS96UC23_CD 25862 TATGCTCCTGCGGAAGTATCTACTCGTTACGGAATCTCAGGTGTCAAAGT 25911

LvirLAC006941_C 25877 TATGCTCCTGCGGAAGTATCTACTCGTTACGGAATCTCAGGTGTCAAAGT 25926

LserLAC005780_C 25895 TATGCTCCTGCGGAAGTATCTACTCGTTACGGAATCTCAGGTGTCAAAGT 25944

LsalLAC008020_C 25898 TATGCTCCTGCGGAAGTATCTACTCGTTACGGAATCTCAGGTGTCAAAGT 25947

LsalCGN5271_CDS 25898 TATGCTCCTGCGGAAGTATCTACTCGTTACGGAATCTCAGGTGTCAAAGT 25947

LsatLACWENDEL_C 25912 GTGGATTTCATATAGTCAAAAAGAAAGGGGACGTGCTATATCCGAAACGT 25961

LsatSalinas_CDS 25912 GTGGATTTCATATAGTCAAAAAGAAAGGGGACGTGCTATATCCGAAACGT 25961

LserUS96UC23_CD 25912 GTGGATTTCATATAGTCAAAAAGAAAGGGGACGTGCTATATCCGAAACGT 25961

LvirLAC006941_C 25927 GTGGATTTCATATAGTCAAAAAGAAAGGGGACGTGCTATATCCGAAACGT 25976

LserLAC005780_C 25945 GTGGATTTCATATAGTCAAAAAGAAAGGGGACGTGCTATATCCGAAACGT 25994

LsalLAC008020_C 25948 GTGGATTTCATATAGTCAAAAAGAAAGGGGACGTGCTATATCCGAAACGT 25997

LsalCGN5271_CDS 25948 GTGGATTTCATATAGTCAAAAAGAAAGGGGACGTGCTATATCCGAAACGT 25997

LsatLACWENDEL_C 25962 ACGAAATATAGATGCCCGCATTAAGATTTAAAACTTGTCGTCTACTTGAA 26011

LsatSalinas_CDS 25962 ACGAAATATAGATGCCCGCATTAAGATTTAAAACTTGTCGTCTACTTGAA 26011

LserUS96UC23_CD 25962 ACGAAATATAGATGCCCGCATTAAGATTTAAAACTTGTCGTCTACTTGAA 26011

LvirLAC006941_C 25977 ACGAAATATAGATGCCCGCATTAAGATTTAAAACTTGTCGTCTACTTGAA 26026

LserLAC005780_C 25995 ACGAAATATAGATGCCCGCATTAAGATTTAAAACTTGTCGTCTACTTGAA 26044

LsalLAC008020_C 25998 ACGAAATATAGATGCCCGCATTAAGATTTAAAACTTGTCGTCTACTTGAA 26047

LsalCGN5271_CDS 25998 ACGAAATATAGATGCCCGCATTAAGATTTAAAACTTGTCGTCTACTTGAA 26047

LsatLACWENDEL_C 26012 GGAAATGTTTGGAACAGGAAACTTACAATAATACAACGCCGCATTCTTCG 26061

LsatSalinas_CDS 26012 GGAAATGTTTGGAACAGGAAACTTACAATAATACAACGCCGCATTCTTCG 26061

LserUS96UC23_CD 26012 GGAAATGTTTGGAACAGGAAACTTACAATAATACAACGCCGCATTCTTCG 26061

LvirLAC006941_C 26027 GGAAATGTTTGGAACAGGAAACTTACAATAATACAACGCCGCATTCTTCG 26076

LserLAC005780_C 26045 GGAAATGTTTGGAACAGGAAACTTACAATAATACAACGCCGCATTCTTCG 26094

LsalLAC008020_C 26048 GGAAATGTTTGGAACAGGAAACTTACAATAATACAACGCCGCATTCTTCG 26097

LsalCGN5271_CDS 26048 GGAAATGTTTGGAACAGGAAACTTACAATAATACAACGCCGCATTCTTCG 26097

LsatLACWENDEL_C 26062 AAGATTGAGGAACAAGACGAGATCTATTAAGAGAATGATTTATTCTCGAA 26111

LsatSalinas_CDS 26062 AAGATTGAGGAACAAGACGAGATCTATTAAGAGAATGATTTATTCTCGAA 26111

LserUS96UC23_CD 26062 AAGATTGAGGAACAAGACGAGATCTATTAAGAGAATGATTTATTCTCGAA 26111

LvirLAC006941_C 26077 AAGATTGAGGAACAAGACGAGATCTATTAAGAGAATGATTTATTCTCGAA 26126

LserLAC005780_C 26095 AAGATTGAGGAACAAGACGAGATCTATTAAGAGAATGATTTATTCTCGAA 26144

LsalLAC008020_C 26098 AAGATTGAGGAACAAGACGAGATCTATTAAGAGAATGATTTATTCTCGAA 26147

LsalCGN5271_CDS 26098 AAGATTGAGGAACAAGACGAGATCTATTAAGAGAATGATTTATTCTCGAA 26147

LsatLACWENDEL_C 26112 AAAATCTGAATAGTTACATCCAATTACAAACTACACGAAAGTTGCCCCTT 26161

LsatSalinas_CDS 26112 AAAATCTGAATAGTTACATCCAATTACAAACTACACGAAAGTTGCCCCTT 26161

LserUS96UC23_CD 26112 AAAATCTGAATAGTTACATCCAATTACAAACTACACGAAAGTTGCCCCTT 26161

LvirLAC006941_C 26127 AAAATCTGAATAGTTACATCCAATTACAAACTACACGAAAGTTGCCCCTT 26176

LserLAC005780_C 26145 AAAATCTGAATAGTTACATCCAATTACAAACTACACGAAAGTTGCCCCTT 26194

LsalLAC008020_C 26148 AAAATCTGAATAGTTACATCCAATTACAAACTACACGAAAGTTGCCCCTT 26197

LsalCGN5271_CDS 26148 AAAATCTGAATAGTTACATCCAATTACAAACTACACGAAAGTTGCCCCTT 26197

LsatLACWENDEL_C 26162 TTTCATGGAGATTTACCCATCACAGAGATGCATAGAGGAACAGAACGAAC 26211

LsatSalinas_CDS 26162 TTTCATGGAGATTTACCCATCACAGAGATGCATAGAGGAACAGAACGAAC 26211

LserUS96UC23_CD 26162 TTTCATGGAGATTTACCCATCACAGAGATGCATAGAGGAACAGAACGAAC 26211

LvirLAC006941_C 26177 TTTCATGGAGATTTACCCATCACAGAGATGCATAGAGGAACAGAACGAAC 26226

LserLAC005780_C 26195 TTTCATGGAGATTTACCCATCACAGAGATGCATAGAGGAACAGAACGAAC 26244

LsalLAC008020_C 26198 TTTCATGGAGATTTACCCATCACAGAGATGCATAGAGGAACAGAACGAAC 26247

LsalCGN5271_CDS 26198 TTTCATGGAGATTTACCCATCACAGAGATGCATAGAGGAACAGAACGAAC 26247

LsatLACWENDEL_C 26212 TTCATATATCCCTTTTCCACTCAATCCAGAAACAAGATCGGACGTTATTC 26261

LsatSalinas_CDS 26212 TTCATATATCCCTTTTCCACTCAATCCAGAAACAAGATCGGACGTTATTC 26261

LserUS96UC23_CD 26212 TTCATATATCCCTTTTCCACTCAATCCAGAAACAAGATCGGACGTTATTC 26261

LvirLAC006941_C 26227 TTCATATATCCCTTTTCCACTCAATCCAGAAACAAGATCGGACGTTATTC 26276

LserLAC005780_C 26245 TTCATATATCCCTTTTCCACTCAATCCAGAAACAAGATCGGACGTTATTC 26294

LsalLAC008020_C 26248 TTCATATATCCCTTTTCCACTCAATCCAGAAACAAGATCGGACGTTATTC 26297

LsalCGN5271_CDS 26248 TTCATATATCCCTTTTCCACTCAATCCAGAAACAAGATCGGACGTTATTC 26297

LsatLACWENDEL_C 26262 CGGTTCGTCTCCATTTTAGTGAAACTCTTCCTCAAGCAAGGCAGCCGATA 26311

LsatSalinas_CDS 26262 CGGTTCGTCTCCATTTTAGTGAAACTCTTCCTCAAGCAAGGCAGCCGATA 26311

LserUS96UC23_CD 26262 CGGTTCGTCTCCATTTTAGTGAAACTCTTCCTCAAGCAAGGCAGCCGATA 26311

LvirLAC006941_C 26277 CGGTTCGTCTCCATTTTAGTGAAACTCTTCCTCAAGCAAGGCAGCCGATA 26326

LserLAC005780_C 26295 CGGTTCGTCTCCATTTTAGTGAAACTCTTCCTCAAGCAAGGCAGCCGATA 26344

LsalLAC008020_C 26298 CGGTTCGTCTCCATTTTAGTGAAACTCTTCCTCAAGCAAGGCAGCCGATA 26347

LsalCGN5271_CDS 26298 CGGTTCGTCTCCATTTTAGTGAAACTCTTCCTCAAGCAAGGCAGCCGATA 26347

LsatLACWENDEL_C 26312 AGTCATCGAAGGCTTTTTGTTAATAATGTAATAGTCAGCATTACTTCTTT 26361

LsatSalinas_CDS 26312 AGTCATCGAAGGCTTTTTGTTAATAATGTAATAGTCAGCATTACTTCTTT 26361

LserUS96UC23_CD 26312 AGTCATCGAAGGCTTTTTGTTAATAATGTAATAGTCAGCATTACTTCTTT 26361

LvirLAC006941_C 26327 AGTCATCGAAGGCTTTTTGTTAATAATGTAATAGTCAGCATTACTTCTTT 26376

LserLAC005780_C 26345 AGTCATCGAAGGCTTTTTGTTAATAATGTAATAGTCAGCATTACTTCTTT 26394

LsalLAC008020_C 26348 AGTCATCGAAGGCTTTTTGTTAATAATGTAATAGTCAGCATTACTTCTTT 26397

LsalCGN5271_CDS 26348 AGTCATCGAAGGCTTTTTGTTAATAATGTAATAGTCAGCATTACTTCTTT 26397

LsatLACWENDEL_C 26362 TCAAGTTTCCCAAGGTGATTTCATATCTTTTAAAGAAAATGATACTCTAA 26411

LsatSalinas_CDS 26362 TCAAGTTTCCCAAGGTGATTTCATATCTTTTAAAGAAAATGATACTCTAA 26411

LserUS96UC23_CD 26362 TCAAGTTTCCCAAGGTGATTTCATATCTTTTAAAGAAAATGATACTCTAA 26411

LvirLAC006941_C 26377 TCAAGTTTCCCAAGGTGATTTCATATCTTTTAAAGAAAATGATACTCTAA 26426

LserLAC005780_C 26395 TCAAGTTTCCCAAGGTGATTTCATATCTTTTAAAGAAAATGATACTCTAA 26444

LsalLAC008020_C 26398 TCAAGTTTCCCAAGGTGATTTCATATCTTTTAAAGAAAATGATACTCTAA 26447

LsalCGN5271_CDS 26398 TCAAGTTTCCCAAGGTGATTTCATATCTTTTAAAGAAAATGATACTCTAA 26447

LsatLACWENDEL_C 26412 TCTATTCAGAAATAAGGAGATCCTTCTATATCGAAATTTCAGTTTCTAAA 26461

LsatSalinas_CDS 26412 TCTATTCAGAAATAAGGAGATCCTTCTATATCGAAATTTCAGTTTCTAAA 26461

LserUS96UC23_CD 26412 TCTATTCAGAAATAAGGAGATCCTTCTATATCGAAATTTCAGTTTCTAAA 26461

LvirLAC006941_C 26427 TCTATTCAGAAATAAGGAGATCCTTCTATATCGAAATTTCAGTTTCTAAA 26476

LserLAC005780_C 26445 TCTATTCAGAAATAAGGAGATCCTTCTATATCGAAATTTCAGTTTCTAAA 26494

LsalLAC008020_C 26448 TCTATTCAGAAATAAGGAGATCCTTCTATATCGAAATTTCAGTTTCTAAA 26497

LsalCGN5271_CDS 26448 TCTATTCAGAAATAAGGAGATCCTTCTATATCGAAATTTCAGTTTCTAAA 26497

LsatLACWENDEL_C 26462 ATCATAGGAAAATTCCTGGATAGCCCGGTAAGAATGTGGAGAAGAACCAA 26511

LsatSalinas_CDS 26462 ATCATAGGAAAATTCCTGGATAGCCCGGTAAGAATGTGGAGAAGAACCAA 26511

LserUS96UC23_CD 26462 ATCATAGGAAAATTCCTGGATAGCCCGGTAAGAATGTGGAGAAGAACCAA 26511

LvirLAC006941_C 26477 ATCATAGGAAAATTCCTGGATAGCCCGGTAAGAATGTGGAGAAGAACCAA 26526

LserLAC005780_C 26495 ATCATAGGAAAATTCCTGGATAGCCCGGTAAGAATGTGGAGAAGAACCAA 26544

LsalLAC008020_C 26498 ATCATAGGAAAATTCCTGGATAGCCCGGTAAGAATGTGGAGAAGAACCAA 26547

LsalCGN5271_CDS 26498 ATCATAGGAAAATTCCTGGATAGCCCGGTAAGAATGTGGAGAAGAACCAA 26547

LsatLACWENDEL_C 26512 AACGGAATGGTTCCGCTTACTTAAAACTAAGAGGGGATGCCGCCTACTAC 26561

LsatSalinas_CDS 26512 AACGGAATGGTTCCGCTTACTTAAAACTAAGAGGGGATGCCGCCTACTAC 26561

LserUS96UC23_CD 26512 AACGGAATGGTTCCGCTTACTTAAAACTAAGAGGGGATGCCGCCTACTAC 26561

LvirLAC006941_C 26527 AACGGAATGGTTCCGCTTACTTAAAACTAAGAGGGGATGCCGCCTACTAC 26576

LserLAC005780_C 26545 AACGGAATGGTTCCGCTTACTTAAAACTAAGAGGGGATGCCGCCTACTAC 26594

LsalLAC008020_C 26548 AACGGAATGGTTCCGCTTACTTAAAACTAAGAGGGGATGCCGCCTACTAC 26597

LsalCGN5271_CDS 26548 AACGGAATGGTTCCGCTTACTTAAAACTAAGAGGGGATGCCGCCTACTAC 26597

LsatLACWENDEL_C 26562 TGAAAGACCCGTTTTTGCAACAGTTGCGTTCTTCTATGCAAGACGAAGAC 26611

LsatSalinas_CDS 26562 TGAAAGACCCGTTTTTGCAACAGTTGCGTTCTTCTATGCAAGACGAAGAC 26611

LserUS96UC23_CD 26562 TGAAAGACCCGTTTTTGCAACAGTTGCGTTCTTCTATGCAAGACGAAGAC 26611

LvirLAC006941_C 26577 TGAAAGACCCGTTTTTGCAACAGTTGCGTTCTTCTATGCAAGACGAAGAC 26626

LserLAC005780_C 26595 TGAAAGACCCGTTTTTGCAACAGTTGCGTTCTTCTATGCAAGACGAAGAC 26644

LsalLAC008020_C 26598 TGAAAGACCCGTTTTTGCAACAGTTGCGTTCTTCTATGCAAGACGAAGAC 26647

LsalCGN5271_CDS 26598 TGAAAGACCCGTTTTTGCAACAGTTGCGTTCTTCTATGCAAGACGAAGAC 26647

LsatLACWENDEL_C 26612 TTAGAAAGAACAAAGAAGTTTGGATCCGAAAAAGTATGCTTAGGCAGTTC 26661

LsatSalinas_CDS 26612 TTAGAAAGAACAAAGAAGTTTGGATCCGAAAAAGTATGCTTAGGCAGTTC 26661

LserUS96UC23_CD 26612 TTAGAAAGAACAAAGAAGTTTGGATCCGAAAAAGTATGCTTAGGCAGTTC 26661

LvirLAC006941_C 26627 TTAGAAAGAACAAAGAAGTTTGGATCCGAAAAAGTATGCTTAGGCAGTTC 26676

LserLAC005780_C 26645 TTAGAAAGAACAAAGAAGTTTGGATCCGAAAAAGTATGCTTAGGCAGTTC 26694

LsalLAC008020_C 26648 TTAGAAAGAACAAAGAAGTTTGGATCCGAAAAAGTATGCTTAGGCAGTTC 26697

LsalCGN5271_CDS 26648 TTAGAAAGAACAAAGAAGTTTGGATCCGAAAAAGTATGCTTAGGCAGTTC 26697

LsatLACWENDEL_C 26662 TTTCGCTGAGCACAAGAGAATAAAGAGGCATTTTTATCATTTCAAATCGA 26711

LsatSalinas_CDS 26662 TTTCGCTGAGCACAAGAGAATAAAGAGGCATTTTTATCATTTCAAATCGA 26711

LserUS96UC23_CD 26662 TTTCGCTGAGCACAAGAGAATAAAGAGGCATTTTTATCATTTCAAATCGA 26711

LvirLAC006941_C 26677 TTTCGCTGAGCACAAGAGAATAAAGAGGCATTTTTATCATTTCAAATCGA 26726

LserLAC005780_C 26695 TTTCGCTGAGCACAAGAGAATAAAGAGGCATTTTTATCATTTCAAATCGA 26744

LsalLAC008020_C 26698 TTTCGCTGAGCACAAGAGAATAAAGAGGCATTTTTATCATTTCAAATCGA 26747

LsalCGN5271_CDS 26698 TTTCGCTGAGCACAAGAGAATAAAGAGGCATTTTTATCATTTCAAATCGA 26747

LsatLACWENDEL_C 26712 TATTCTTATCGAAGAGAAGGAACGAAAAAACCCTAAATCTTACTACTAGA 26761

LsatSalinas_CDS 26712 TATTCTTATCGAAGAGAAGGAACGAAAAAACCCTAAATCTTACTACTAGA 26761

LserUS96UC23_CD 26712 TATTCTTATCGAAGAGAAGGAACGAAAAAACCCTAAATCTTACTACTAGA 26761

LvirLAC006941_C 26727 TATTCTTATCGAAGAGAAGGAACGAAAAAACCCTAAATCTTACTACTAGA 26776

LserLAC005780_C 26745 TATTCTTATCGAAGAGAAGGAACGAAAAAACCCTAAATCTTACTACTAGA 26794

LsalLAC008020_C 26748 TATTCTTATCGAAGAGAAGGAACGAAAAAACCCTAAATCTTACTACTAGA 26797

LsalCGN5271_CDS 26748 TATTCTTATCGAAGAGAAGGAACGAAAAAACCCTAAATCTTACTACTAGA 26797

LsatLACWENDEL_C 26762 AAAAGAAGTCCTATAGTTTACAACTCTTCTTTCTATAGTAATTTGACCTC 26811

LsatSalinas_CDS 26762 AAAAGAAGTCCTATAGTTTACAACTCTTCTTTCTATAGTAATTTGACCTC 26811

LserUS96UC23_CD 26762 AAAAGAAGTCCTATAGTTTACAACTCTTCTTTCTATAGTAATTTGACCTC 26811

LvirLAC006941_C 26777 AAAAGAAGTCCTATAGTTTACAACTCTTCTTTCTATAGTAATTTGACCTC 26826

LserLAC005780_C 26795 AAAAGAAGTCCTATAGTTTACAACTCTTCTTTCTATAGTAATTTTACCTC 26844

LsalLAC008020_C 26798 AAAAGAAGTCCTATAGTTTACAACTCTTCTTTCTATAGTAATTTGACCTC 26847

LsalCGN5271_CDS 26798 AAAAGAAGTCCTATAGTTTACAACTCTTCTTTCTATAGTAATTTGACCTC 26847

LsatLACWENDEL_C 26812 TTGCTCCACCCATCAGTCTTCTATGAAGAGAAAAATAAAAAGCTCTTCCC 26861

LsatSalinas_CDS 26812 TTGCTCCACCCATCAGTCTTCTATGAAGAGAAAAATAAAAAGCTCTTCCC 26861

LserUS96UC23_CD 26812 TTGCTCCACCCATCAGTCTTCTATGAAGAGAAAAATAAAAAGCTCTTCCC 26861

LvirLAC006941_C 26827 TTGCTCCACCCATCAGTCTTCTATGAAGAGAAAAATAAAAAGCTCTTCCC 26876

LserLAC005780_C 26845 TTGCTCCACCCATCAGTCTTCTATGAAGAGAAAAATAAAAAGCTCTTCCC 26894

LsalLAC008020_C 26848 TTGCTCCACCCATCAGTCTTCTATGAAGAGAAAAATAAAAAGCTCTTCCC 26897

LsalCGN5271_CDS 26848 TTGCTCCACCCATCAGTCTTCTATGAAGAGAAAAATAAAAAGCTCTTCCC 26897

LsatLACWENDEL_C 26862 TATCTACTCATTATTCGGAGGTGAATCATAGAACACTAAAAGCTGTGCTA 26911

LsatSalinas_CDS 26862 TATCTACTCATTATTCGGAGGTGAATCATAGAACACTAAAAGCTGTGCTA 26911

LserUS96UC23_CD 26862 TATCTACTCATTATTCGGAGGTGAATCATAGAACACTAAAAGCTGTGCTA 26911

LvirLAC006941_C 26877 TATCTACTCATTATTCGGAGGTGAATCATAGAACACTAAAAGCTGTGCTA 26926

LserLAC005780_C 26895 TATCTACTCATTATTCGGAGGTGAATCATAGAACACTAAAAGCTGTGCTA 26944

LsalLAC008020_C 26898 TATCTACTCATTATTCGGAGGTGAATCATAGAACACTAAAAGCTGTGCTA 26947

LsalCGN5271_CDS 26898 TATCTACTCATTATTCGGAGGTGAATCATAGAACACTAAAAGCTGTGCTA 26947

LsatLACWENDEL_C 26912 TCTTATGGACCTAACATAGGTCACATCCCTCACGTGCGCCAAGAGCACAT 26961

LsatSalinas_CDS 26912 TCTTATGGACCTAACATAGGTCACATCCCTCACGTGCGCCAAGAGCACAT 26961

LserUS96UC23_CD 26912 TCTTATGGACCTAACATAGGTCACATCCCTCACGTGCGCCAAGAGCACAT 26961

LvirLAC006941_C 26927 TCTTATGGACCTAACATAGGTCACATCCCTCACGTGCGCCAAGAGCACAT 26976

LserLAC005780_C 26945 TCTTATGGACCTAACATAGGTCACATCCCTCACGTGCGCCAAGAGCACAT 26994

LsalLAC008020_C 26948 TCTTATGGACCTAACATAGGTCACATCCCTCACGTGCGCCAAGAGCACAT 26997

LsalCGN5271_CDS 26948 TCTTATGGACCTAACATAGGTCACATCCCTCACGTGCGCCAAGAGCACAT 26997

LsatLACWENDEL_C 26962 TCGATAG 26968

LsatSalinas_CDS 26962 TCGATAG 26968

LserUS96UC23_CD 26962 TCGATAG 26968

LvirLAC006941_C 26977 TCGATAG 26983

LserLAC005780_C 26995 TCGATAG 27001

LsalLAC008020_C 26998 TCGATAG 27004

LsalCGN5271_CDS 26998 TCGATAG 27004
